# Supplementary material for: Cancer immune control needs senescence induction by interferon-dependent cell cycle regulator pathways in tumours
Source: Nat Commun. 2020 Mar 12;11:1335. doi: 10.1038/s41467-020-14987-6 (PMC7067802; doi:10.1038/s41467-020-14987-6)
Supplement: Supplementary file 1 — Supplementary Information [file 41467_2020_14987_MOESM1_ESM.pdf]

Supplementary Information

Cancer immune control needs senescence induction by interferon-dependent cell cycle regulator pathways in tumours

Brenner et al.

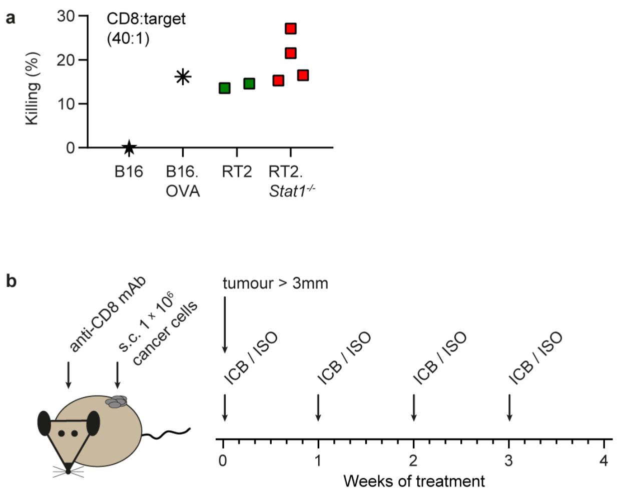

Supplementary Figure 1 Treatment protocol depicting the immune therapy of transplanted RT2-cancers and the ability for CD8 T cell-mediated killing of RT2 and of RT2.*Stat1*$^{-/-}$-cancer cells *in vitro*. **a** B16-F10 (*n*=1) or OVA-expressing B16 melanoma cells (*n*=1) (C57BL/6), or RT2 (*n*=2) or *Stat1*$^{-/-}$ RT2-cancer cells (*n*=4) (C3HeB/FeJ) were cultured with OVA-reactive C57BL/6 CD8^{+} cytotoxic T cells (what recognize the allogeneic RT2-cancers on the C3HeB/FeJ background) and specific chromium release was determined after 1.5 h. **b**, C3HeB/FeJ mice were CD8^{+} T cell depleted with rat anti-mouse-CD8 mAb[40]. After three days 1 × 10^{6} RT2-, RT2.*Stat1*$^{-/-}$-, RT2.*Cdkn2a*$^{-/-}$- or RT2.CRISPR-*Cdkn2a*-cancer cells were subcutaneously (s.c.) injected. Treatment was started with either isotype control mAbs (Ctr) or immune checkpoint inhibitors (ICB; anti-PD-L1 and anti-LAG-3) once per week was started when tumours were > 3 mm in diameter. CD8 T cells did not recover during the time of the experiment. Cancer size was measured 2 times per week.

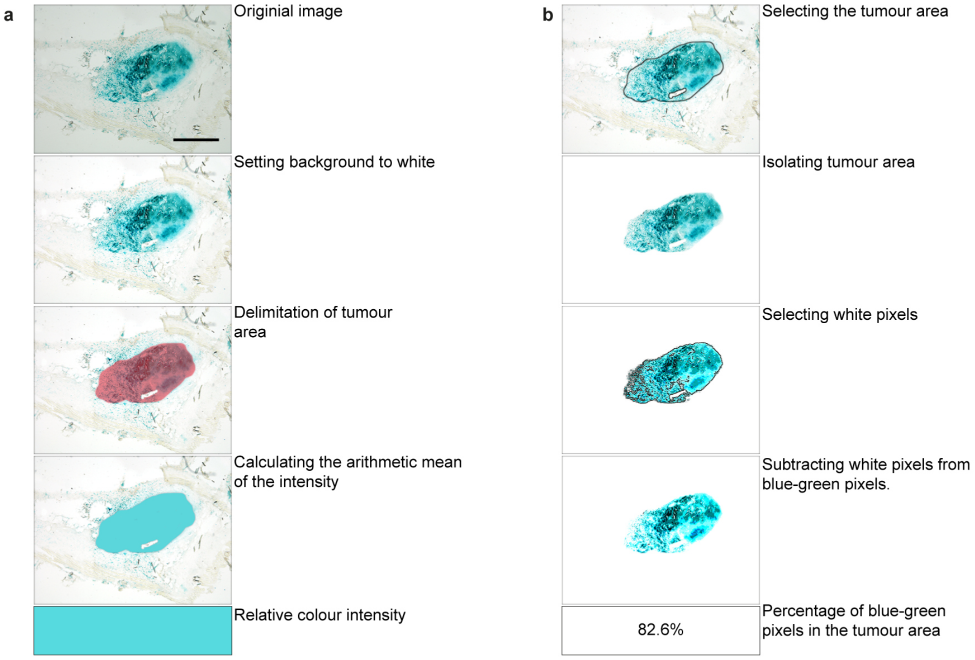

Supplementary Figure 2 Illustration of the digital image-processing, and of the calculation of the percentage of SA-β-gal positive tumour cells within a tumour area. **a**, **b** Image analysis of the field of an ICB/AT treated RT2-cancer stained with SA-β-galactosidase at pH 5.5. **a** Calculation of the SA-β-gal intensity within a given tumour area. **b** Calculation of the percentage of SA-β-gal positive cells within the tumour area. The technique is described in the section Methods. Scale bar 1000 μm.

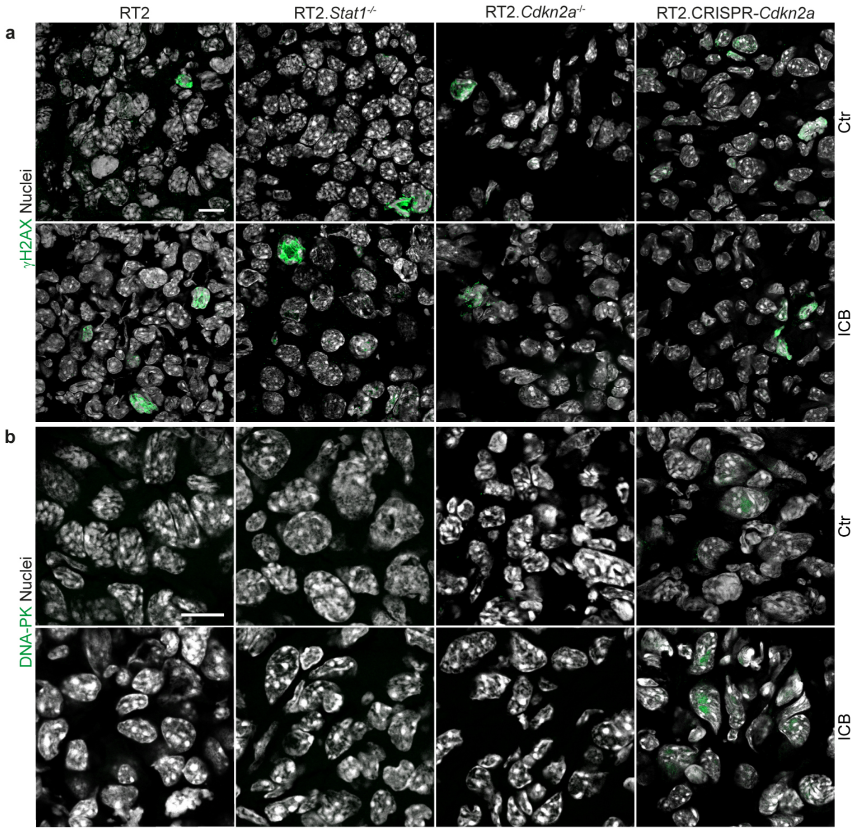

Supplementary Figure 3 Absence of ICB-induced DNA double-strand break-associated γH2AX or DNA-PK expression. **a**, **b** Representative immune fluorescence microscopic images of transplanted pancreatic islet cancers from either RT2-, RT2.*Stat1*$^{-/-}$- or RT2.*Cdkn2a*$^{-/-}$- or from RT2.CRISPR-*Cdkn2a*-cancer cells. Mice were treated with isotype control mAbs (Ctr) or with immune checkpoint blockade (ICB; anti-PD-L1 and anti-LAG-3). **a** Staining for the DNA double-strand break marker γH2AX (green) and for nuclei (white). **b** Staining for DNA damage response kinase DNA-PK (green) and for nuclei (white). Scale bars 10 µm. Histology was performed in one to three representative tumours from Fig. 1c.

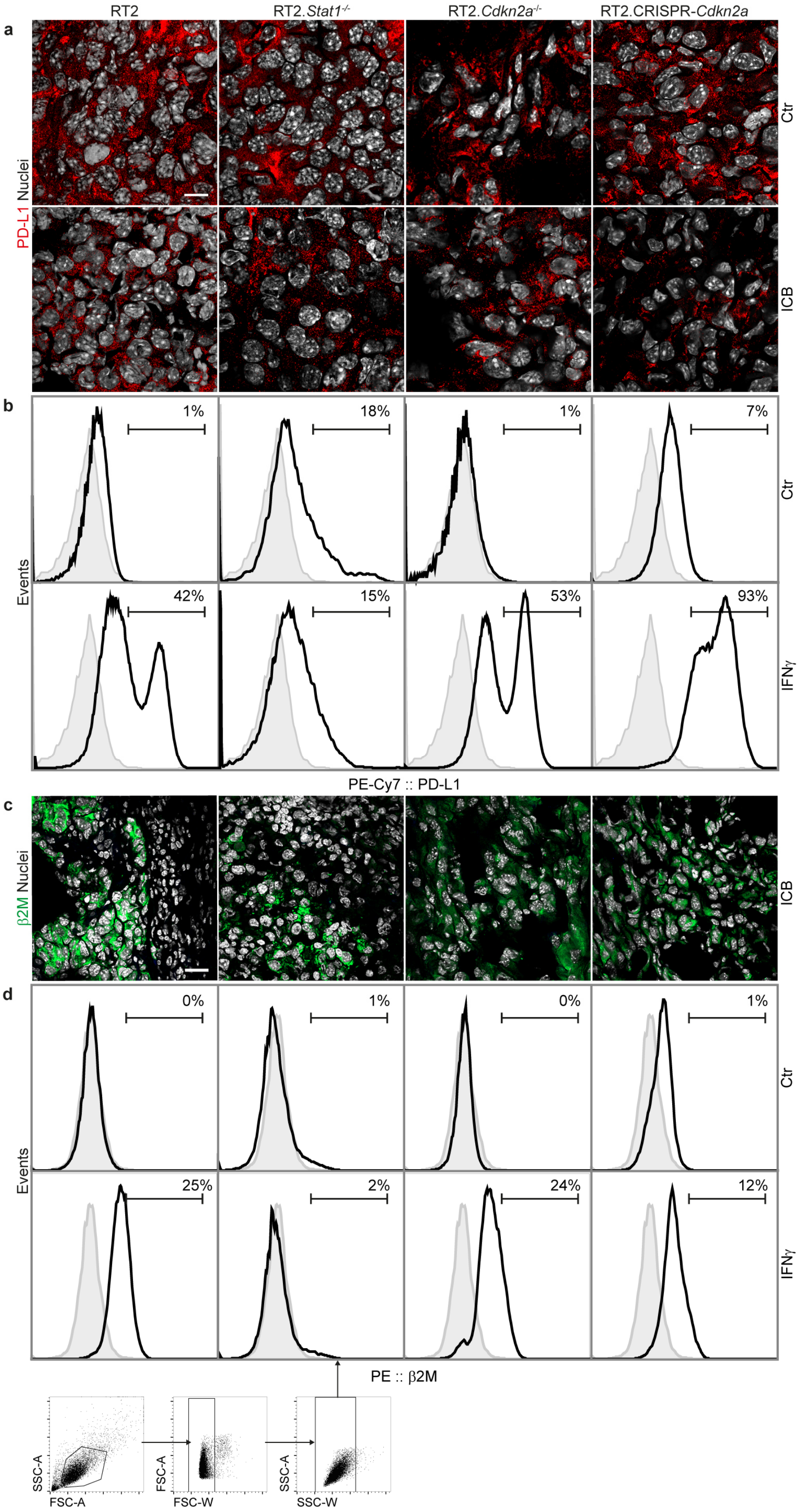

Supplementary Figure 4 PD-L1 or β2-microglobulin protein expression**. a**, **c** Representative immune fluorescence microscopic images of cancers derived from either transplanted RT2-cancer cells, or from transplanted RT2.*Stat1*$^{-/-}$- or RT2.*Cdkn2a*$^{-/-}$- or RT2.CRISPER-*Cdkn2a*-cancer cells. Mice were treated with isotype control mAbs (Ctr) or with immune checkpoint blockade (ICB; anti-PD-L1 and anti-LAG-3). Staining of tumour tissue for the inhibitory

membrane protein PD-L1 (red) and for nuclei (white) (**a**) and for β2-microglobulin (β2M, green) and for nuclei (white) (**c**). To detect the PD-L1 molecules also on cancers of mice treated *in vivo* with mAb PD-L1 (clone 10F.9G2), sections were stained with another unlabelled anti-PD-L1 mAb clone MIH6. To detect both unlabelled anti-PD-L1 mAbs, sections were counterstained with a fluorescence labelled anti-rat antibody. Scale bars 20 µm (**a**), 10 µm (**c**). **b**, **d** FACS analysis of surface expression of PD-L1 (**b**) or of β2-microglobulin (β2M) (**d**) on RT2-cancer cells, RT2.*Stat1*$^{-/-}$- or RT2.*Cdkn2a*$^{-/-}$- or from RT2.CRISPR-*Cdkn2a*-cancer cells, cultured with medium or with medium containing 100 ng ml^{-1} IFN-γ (including gating strategy).

a

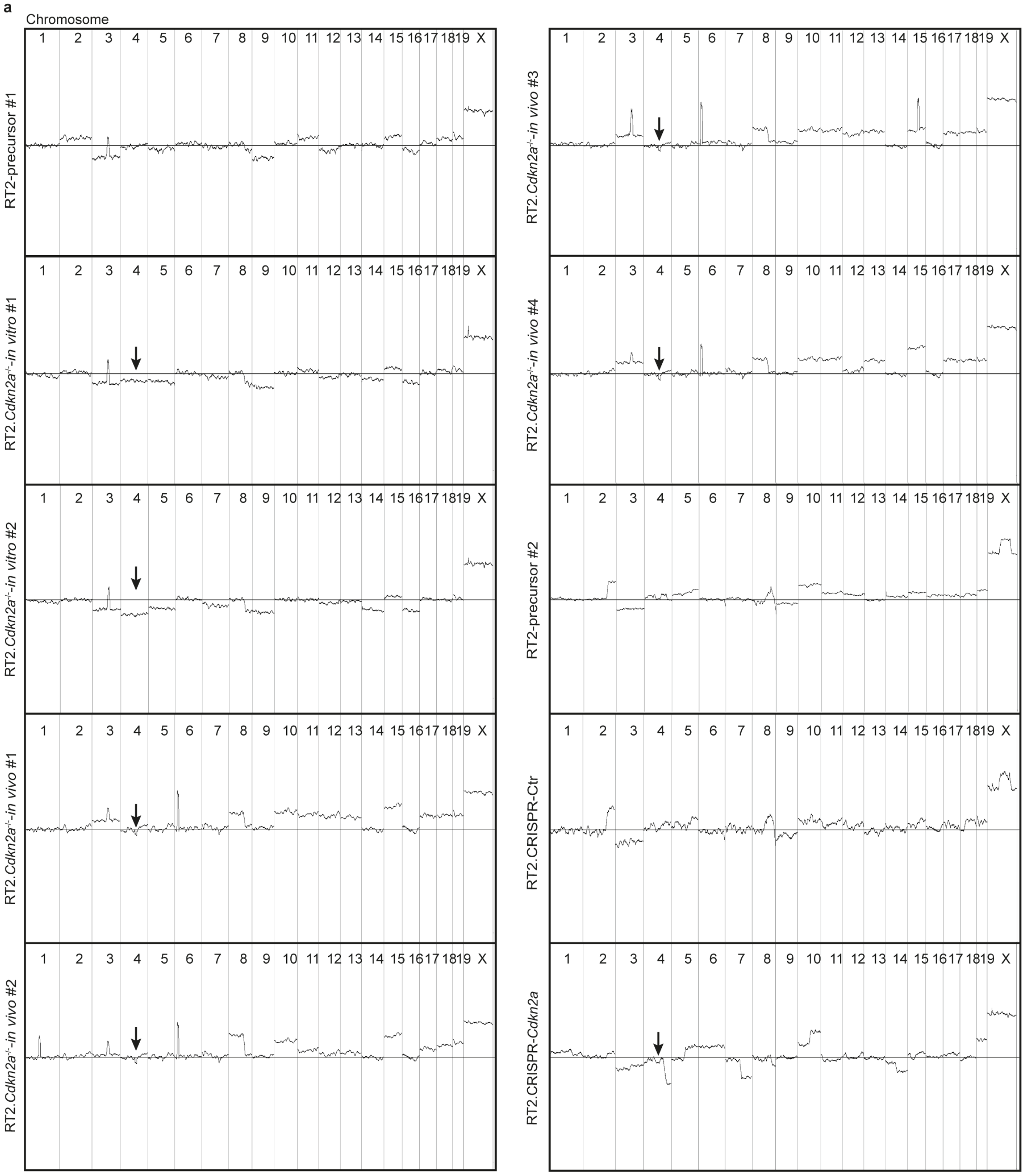

b

Relative expression of *Cdkn2a*			
RT2	RT2.*Cdkn2a*$^{-/-}$	RT2.CRISPR-Ctr	RT2.CRISPR-*Cdkn2a*
1,00	-7,50	1,12	-21,60

c

Engraftment of s.c. transplanted primary RT2-cells			
Type of RT2-cancer cells	s.c. implanted cancers	cancer Ø ≥ 3 mm	engraftment (%)
RT2.CRISPR-Ctr	20	0	0%
RT2.CRISPR-*Cdkn2a*	14	11	79%

Supplementary Figure 5 Genomic profiles of *Cdkn2a*-deficient variants of RT2-cancer cells and their parental RT2-cancer cells, expression of *Cdkn2a* mRNA and their engraftment *in vivo*. **a** Comparative genomic hybridization (CGH) arrays of RT2-cancer cells compared with the chromosome ideogram of wildtype C3HeB/FeJ mice, genome of interest from female mice (XX) compared to reference DNA of male mice (XY). The *Cdkn2a*-loss variants on

chromosome 4, qC4.A were generated by selection of cells resistant to immune therapy (by *in vitro* or *in vivo* selection) from the RT2-precursor cancer line#1. The RT2.CRISPR-Ctr or RT2.CRISPR-*Cdkn2a* were generated from the RT2-precursor cancer line#2, using a published construct[33] that targets $p16^{Ink4a}p19^{Arf}$. Arrows point towards the *Cdkn2a* locus. **b** Table showing the relative expression levels of *Cdkn2a* in RT2-cancers (set as 1), RT2.$Stat1^{-/-}$-, RT2.$Cdkn2a^{-/-}$-, RT2.CRISPR-Ctr- or RT2.CRISPR-*Cdkn2a*-cancer cells. Gene expression of *Cdkn2a* was analysed using *Actb* and *Eef1a1* as references. **c** Number of mice that were subcutaneously (s.c.) engrafted with 1×10^6 cancer cells and engraftment efficacy of either RT2.CRISPR-Ctr- or RT2.CRISPR-*Cdkn2a*-cancer cells. All RT2-cancer lines expressed *SV40-Tag*, see also Fig. 5.

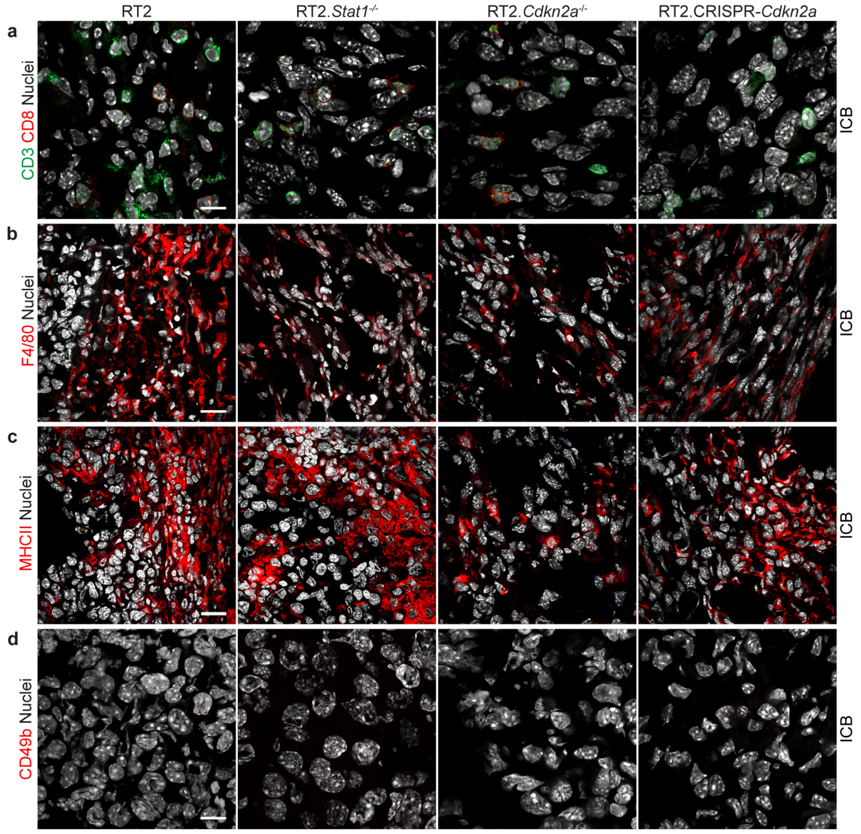

Supplementary Figure 6 Infiltration of $CD3^{+}CD8^{-}$ T cells and of $F4/80^{+}$ macrophages but absence of $CD49b^{+}$ NK cells in transplanted tumours after immune checkpoint blockade therapy. Representative immune fluorescence microscopic images from cancers growing after transplantation of RT2-, from RT2.*Stat1*$^{-/-}$-, from RT2.*Cdkn2a*$^{-/-}$- or from RT2.CRISPR-*Cdkn2a*-cancer cells into CD8-depleted C3H mice. Mice were treated with immune checkpoint blockade (ICB) as shown in Supplementary Figure 1. **a** Staining for CD8 (red) and CD3 (green) T cells and for nuclei (white), scale bar 10 µm. **b** Staining for F4/80 macrophages (red) and for nuclei (white), **c** for MCH class II (red) and for nuclei (white), and **d** for CD49b (red) and for nuclei (white), scale bar 20 µm. Immune histology was performed in one to two representative tumours from Fig. 1c.

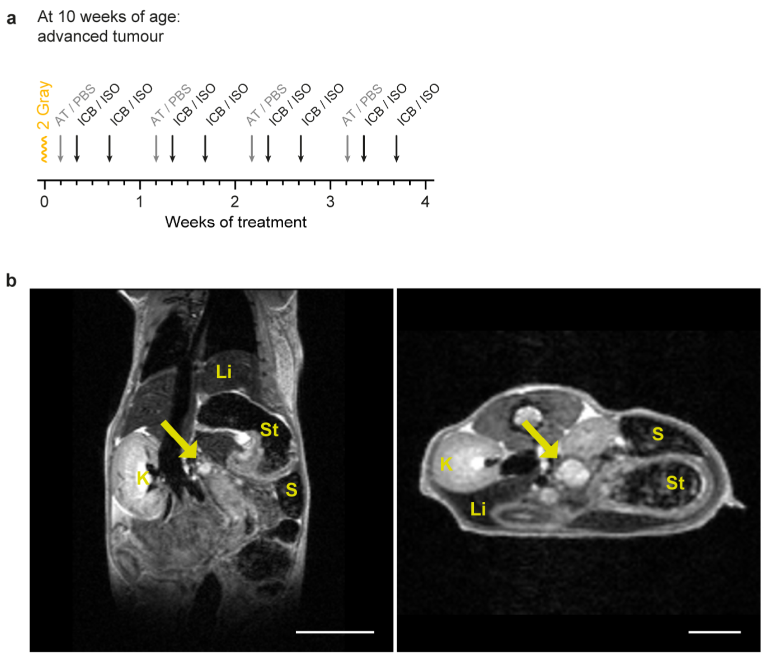

Supplementary Figure 7 Treatment protocol depicting the immune therapy of either RT2 mice or RT2.*Stat1*$^{-/-}$ mice with advanced RT2-cancers. **a** At 10 weeks of age four weeks prior to the expected death RT2 mice or RT2.*Stat1*$^{-/-}$ mice were irradiated with 2 Gy one day before the first i.p. transfer of 1×10^7 T antigen-specific T_H1 cells. T_H1 cells were prepared as described in Methods. Cell transfer was applied once weekly. ICB (anti-PD-L1/ anti-LAG-3) were i.p. injected twice per week. Ctr mice received isotype control mAbs. Blood glucose was measured twice per week. **b** Representative magnetic resonance images of a RT2 mouse at 10 weeks of age. Coronal view (left), transversal view (right). Arrows point towards RT2-cancers. Spleen (S), stomach (St), kidney (K), liver (L), scale bars 10 mm.

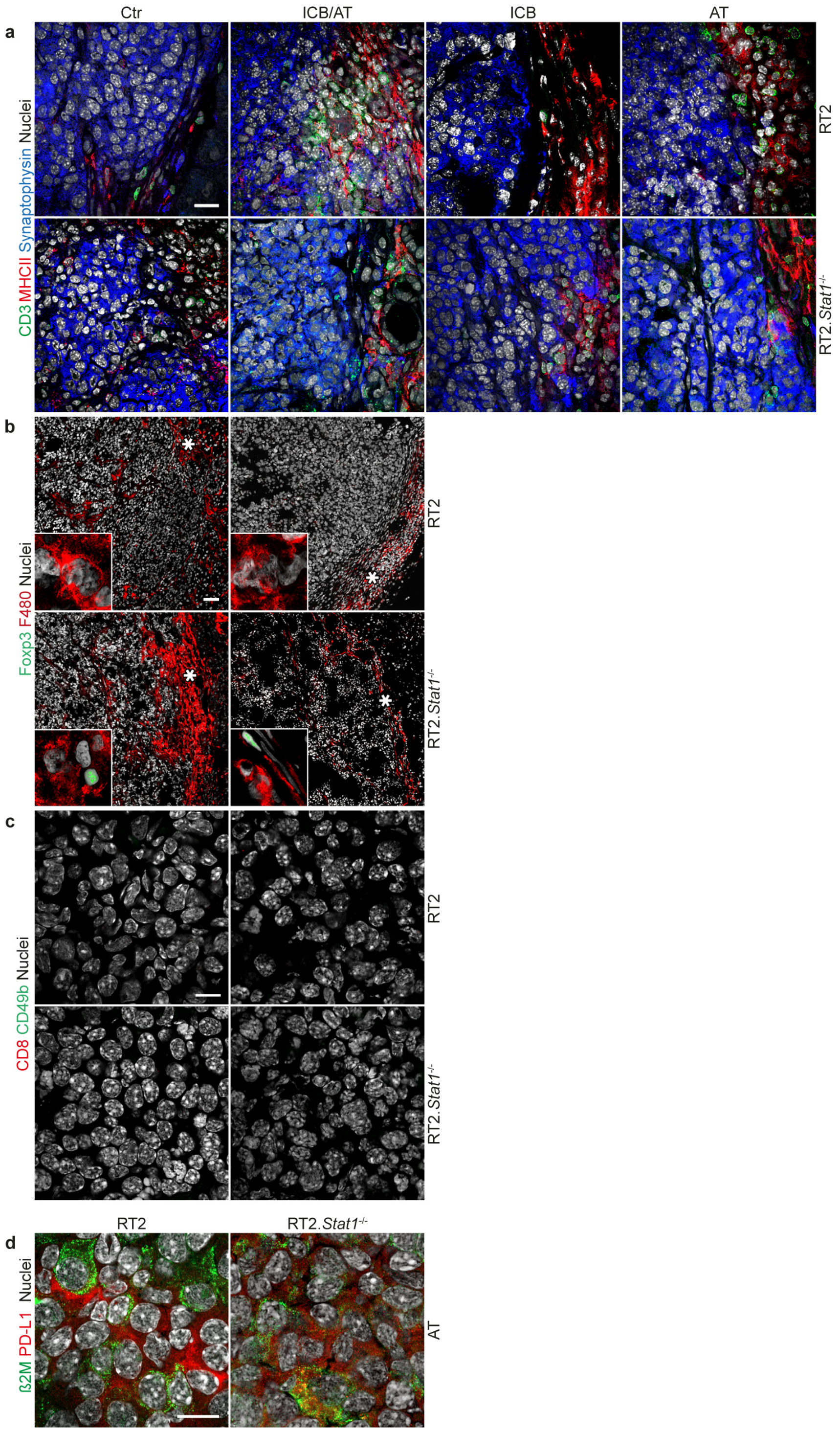

Supplementary Figure 8 Immune infiltration and PD-L1 expression in cancers from RT2 mice or RT2.*Stat1*$^{-/-}$ mice. **a** MHC class II^{+} antigen-presenting cells and CD3^{+} cells in the microenvironment of RT2-cancers of either RT2 mice or RT2.*Stat1*$^{-/-}$ mice after treatment with either isotype control mAbs (Ctr) or with immune checkpoint blockade and adoptive T cell transfer (ICB/AT) or with either ICB or AT. Staining for MHC class II^{+} APCs (in red) and the CD3ε chain on T cells (in green) and for the tumour specific neuroendocrine marker synaptophysin (in blue) and for nuclei (in white), **b** Staining for F480^{+} on macrophages (red) or Foxp3^{+} regulatory T cells (green) and for nuclei (white), **c** for CD8 (red) and CD49b

(green) and for nuclei (white). **d** Expression of the ICB target PD-L1 and of the β2-microglobulin (β2M) in the microenvironment of *Stat1*$^{+/+}$ and *Stat1*$^{-/-}$ RT2-cancers after AT with *Stat1*$^{+/+}$ T_H1 cells. Staining for PD-L1 (red) and β2M (green) and for nuclei (white). **a-d** Immune histology was performed in one to two representative tumours from Fig. 4e. Scale bars 20 µm (**a**), 50 µm (**b**) 10 µm (**c**, **d**).

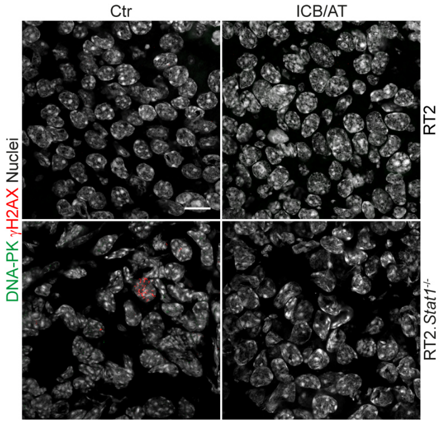

Supplementary Figure 9 Absence of DNA double-strand break-associated γH2AX or DNA-PK expression following immune therapy of RT2-cancers. Representative immune fluorescence microscopic images of pancreatic islet cancers from RT2 or RT2.*Stat1*$^{-/-}$ mice treated either with isotype control mAbs (Ctr) or with immune checkpoint blockade and adoptive T cell transfer (ICB/AT)**.** Remaining tumour cells were stained for the DNA double-strand break marker γH2AX (red), the DNA damage response kinase DNA-PK (green) and for nuclei (white). Immune histology was performed in one to two representative tumours from Fig. 4e. Scale bar 10 µm.

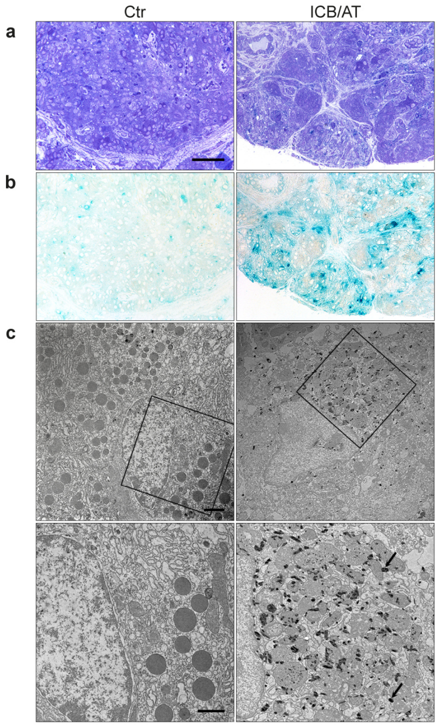

Supplementary Figure 10 Cytoplasmic localisation of SA-β-gal in ultrathin sections. **a** Overview image of RT2-cancers from mice treated either with isotype control mAbs (Ctr) or immune therapy (ICB/AT). Tissues were stained with toluidin blue. **b** Corresponding SA-β-gal-staining of RT2-cancers at pH 5.5. **c** Corresponding electron microscopy (EM) of ultra-thin sections of SA-β-gal-stained RT2-cancers, overview of upper panel detail lower panel. Note the black electron-dense dots in the cytoplasm (arrows). Scale bars 100 µm (**a**), 2 µm (**c** upper panel), 1 µm (**c** lower panel).

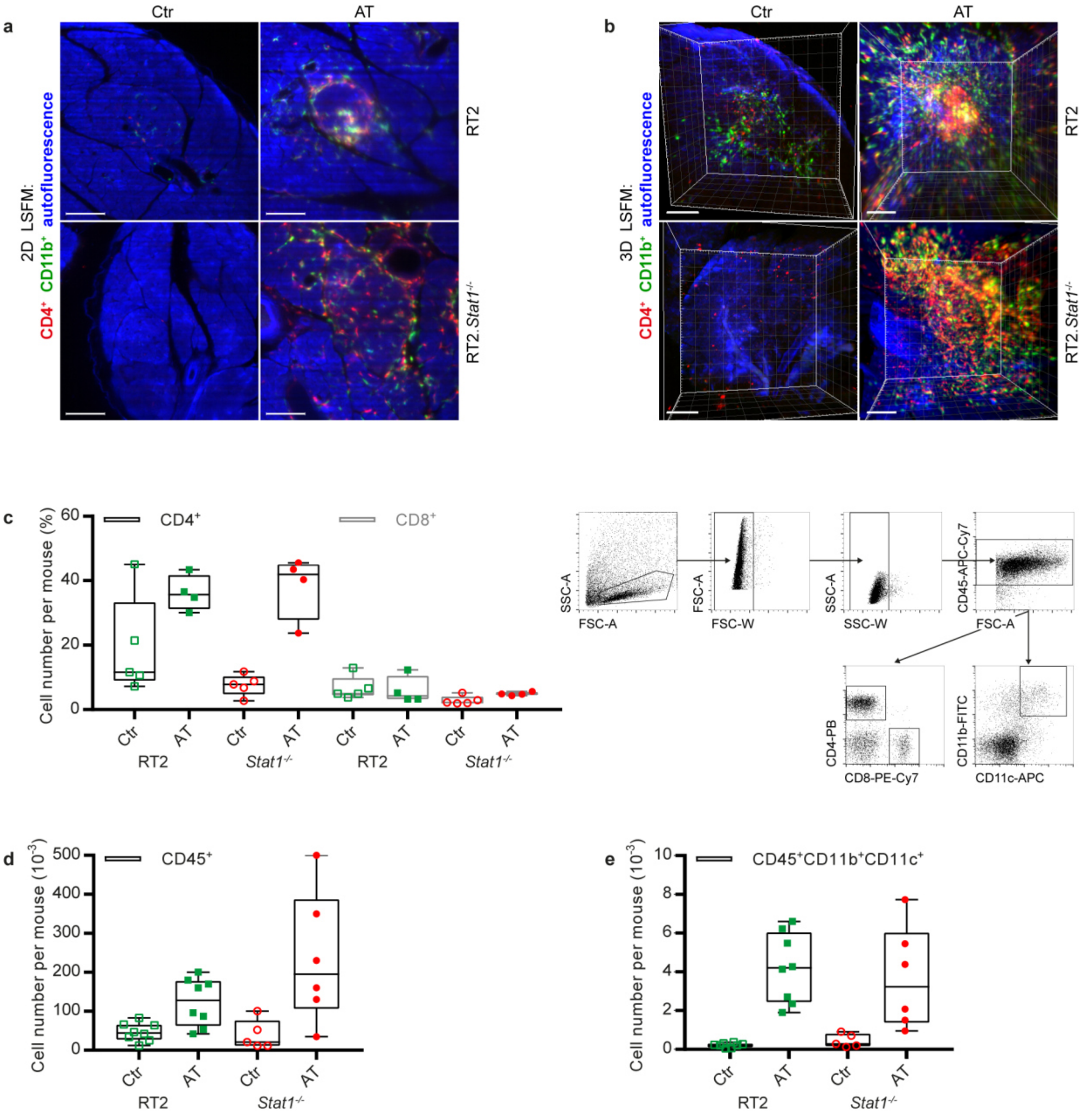

Supplementary Figure 11 Presence of CD4$^+$ T cells, CD11c$^+$- and CD11b$^+$ leukocytes in the tumour microenvironment of RT2-cancers from either RT2 mice or RT2.*Stat1*$^{-/-}$ mice following the injection of Tag-specific T$_H$1 cells (AT). **a** *In vivo* 2D light sheet fluorescence microscopy images (LSFM); **b** 3D LSFM, CD4$^+$ (red), CD11b$^+$ (green), autofluorescence (blue). Labelled mAbs were injected 2 h prior before organs were harvested. The pancreas from either RT2 mice or from RT2.*Stat1*$^{-/-}$ mice was isolated 2 days after the second treatment with either NaCl control (Ctr) or adoptive T cell transfer (AT), scale bars 100 µm. **c-e** Flow cytometry analysis of tumour-infiltrating mononuclear leucocytes in the pancreas of RT2 or RT2.*Stat1*$^{-/-}$mice 2 days after the second AT; percentage of CD4$^+$ and CD8$^+$ T cells (including gating strategy) (**c**); number of CD45$^+$ immune cells (**d**); number of CD45$^+$CD11b$^+$CD11c$^+$ dendritic cells (**e**). Each data point represents one mouse (Ctr, RT2 *N*=5, RT2.*Stat1*$^{-/-}$ *N*=5; AT, RT2 *N*=4, RT2.*Stat1*$^{-/-}$ *N*=4), box plots show the median, and whiskers indicate the 25th and 75th percentiles.

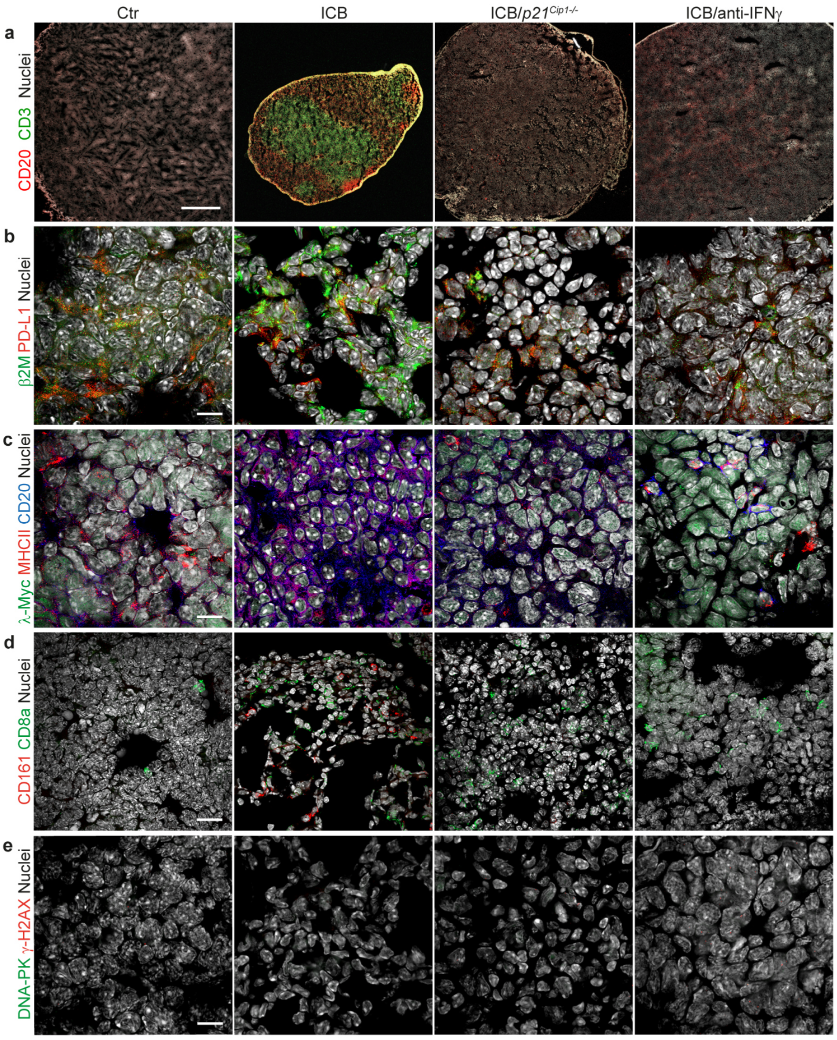

Supplementary Figure 12 Immune therapy with anti-CTLA-4 mAb and anti-PD-1 mAb of λ-*MYC* mice preserves the normal lymph node structure without inducing DNA-double strand breaks. **a**-**e** Representative lymph nodes of λ-*MYC* or λ-*MYC.p21*$^{Cip1-/-}$ mice. λ-*MYC* mice were controls (Ctr) or mice treated with anti-CTLA-4 and anti-PD-1 mAbs (ICB) or anti-CTLA-4, anti-PD-1 and anti-IFN-γ mAbs (ICB/anti-IFN-γ). λ-*MYC.p21*$^{Cip1-/-}$ mice were treated with ICB (group labelled ICB/*p21*$^{Cip1-/-}$). **a**. CD20 (red), CD3 (green), nuclei (white). **b** Staining for PD-L1 (red), β2-microglobulin (β2M, green) and for nuclei (white). **c** Staining for CD20 B-cells (blue; λ-*MYC* lymphoma cells are CD20^{-}), MHC class II (red), λ-MYC (green) and for nuclei (white). **d** Staining for CD161 NK cells (red) and CD8a T cells (green) and for nuclei (white). **e** Staining for the DNA double-strand break marker γH2AX (red), the DNA damage response kinase DNA-PK (green) and for nuclei (white). Immune histology was performed in one to two representative tumours from Fig. 8c. Scale bars, 500 µm (**a**), 10 µm (**b**-**e**).

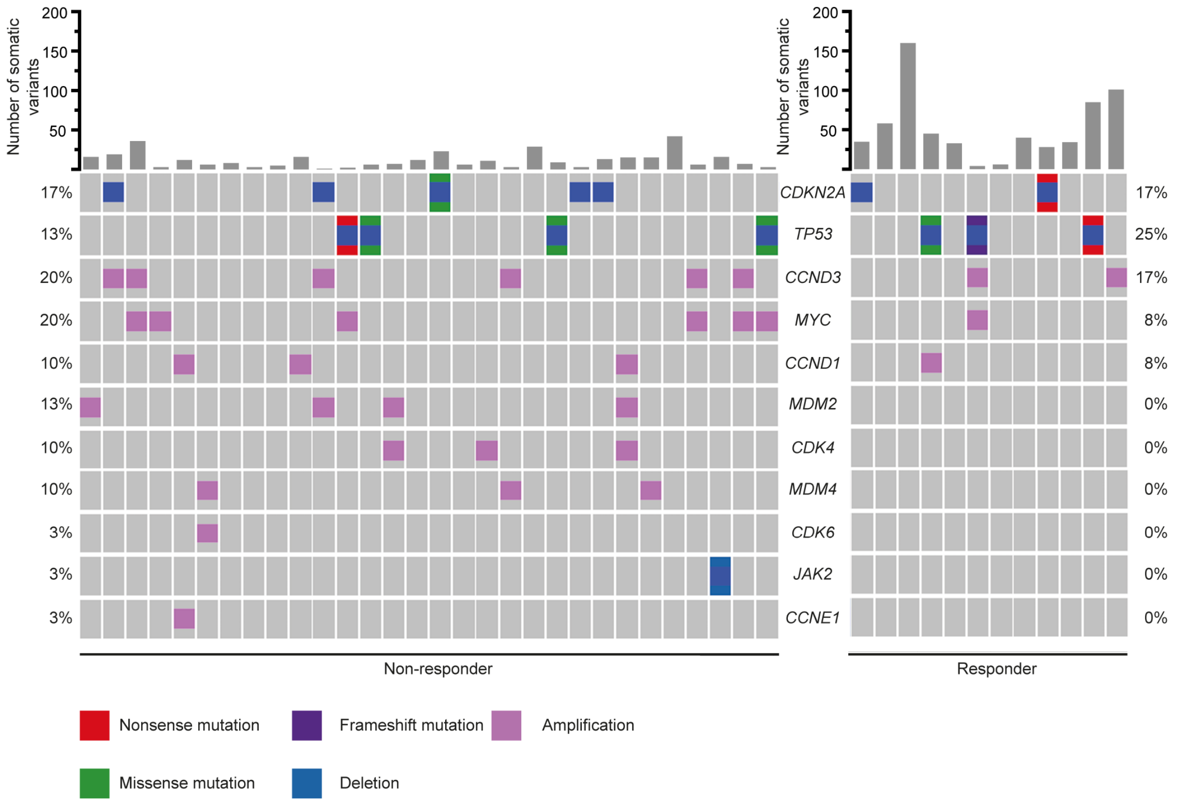

Supplementary Figure 13 Function-determining aberrations of IFN-γ-dependent cell cycle regulator genes are increased in melanoma metastases not responding to ICB therapy. Mutation Oncoplot with loss of function and gain of function alterations of cell cycle control genes leading to severe, function altering mutations. The mutation frequency alternates for each gene in metastases of the non-responder patients (progression within 3 months of ICB) is shown on the left and for each gene in metastases of the responder patients (melanoma regression ≥ 1 year) on the right. No changes in *CCND2, CDKN2B, CDKN2C, CDKN1a, CDKN1B, RB1, JAK1* or *JAK3* were detected.

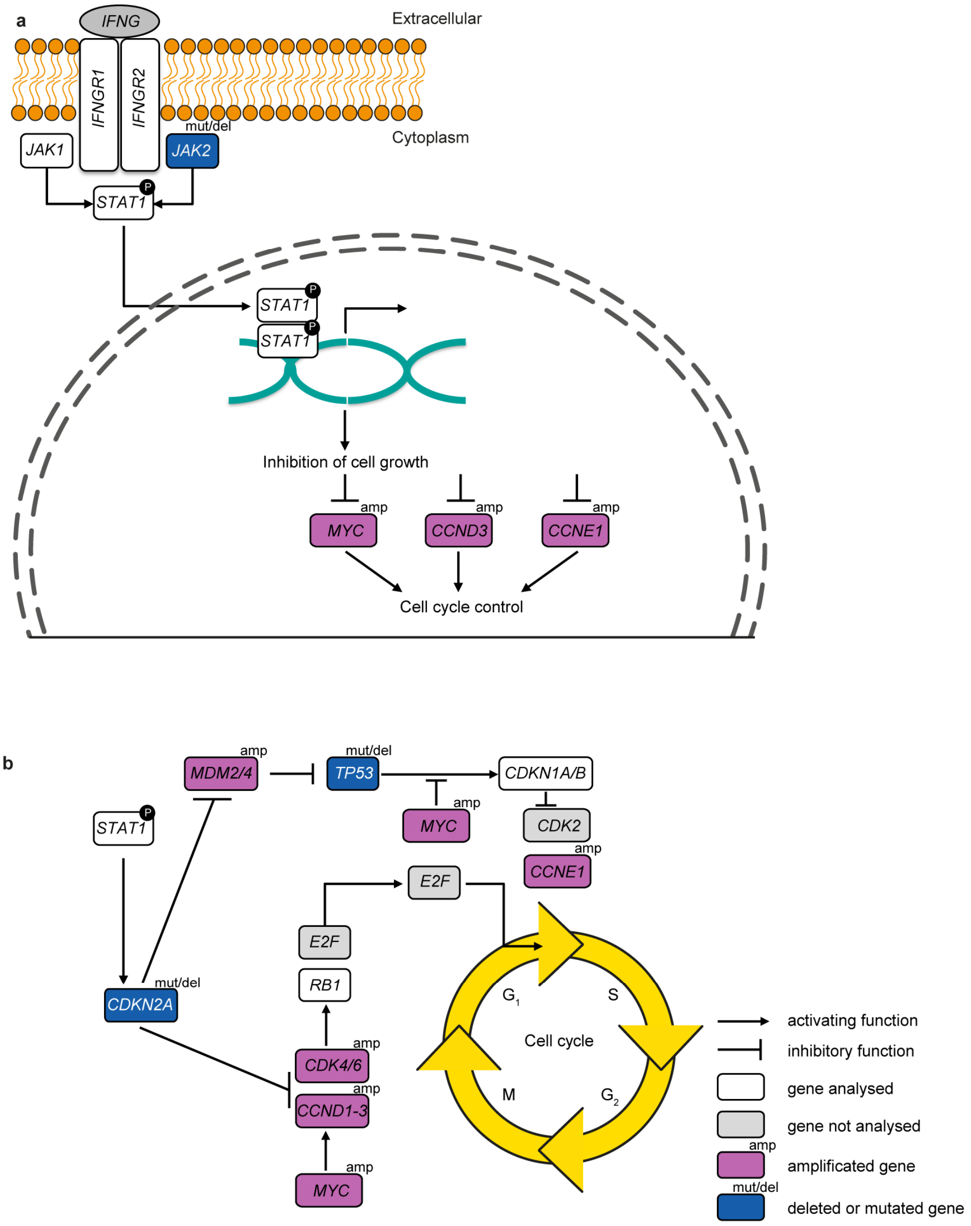

Supplementary Figure 14 Schematic overview of the cell cycle control genes and their functional gains and losses in metastases of non-responder patients. **a** IFN-receptor signalling. **b** Senescence regulating cell cycle control genes. Abbreviation: IFN-γ, interferon gamma; *IFNGR1/2*, interferon gamma receptor 1/2; *JAK1/2/3*; Janus kinase 1/2/3; *STAT1*, signal transducer and activator of transcription protein family 1; *MYC*, MYC proto-oncogene; *CCND1-3*, cyclin-D1-3; *CCNE1*, cyclin-E1; *MDM2*, MDM2 proto-oncogene; *MDM4*, MDM4, p53 regulator; *TP53*, tumour protein p53; *CDKN1A/B*, cyclin dependent kinase inhibitor 1A/B (p21^{Cip1}/p27^{Kip1}); *CDKN2A*, cyclin-dependent kinase inhibitor 2A/2B/2C (p16^{Ink4a}/p14^{Arf}/ p18^{Ink4c}); *CDK2/4/6*, cyclin dependent kinase 2/4/6; *E2F*, transcription factor E2F; *RB1*, retinoblastoma protein 1. Cell cycle phases: G1/G2, Gap1/2 checkpoint control; S, synthesis; M, Mitosis.
