# Supplementary material for: Cancer immune control needs senescence induction by interferon-dependent cell cycle regulator pathways in tumours
Source: Nat Commun. 2020 Mar 12;11:1335. doi: 10.1038/s41467-020-14987-6 (PMC7067802; doi:10.1038/s41467-020-14987-6)
Supplement: Supplementary file 3 — Description of Additional Supplementary Files [file 41467_2020_14987_MOESM3_ESM.pdf]

Description of Additional Supplementary Files

File Name: Supplementary Data 1
Description: Custom gene panel ssSCv2 designed to detect somatic mutations (SNVs), small insertions and deletions (INDELs), copy number alterations (CNAs) and selected structural rearrangements. List of target regions, total size: 1.256 Mbp.

File Name: Supplementary Data 2
Description: Custom gene panel ssSCv3 designed to detect somatic mutations (SNVs), small insertions and deletions (INDELs), copy number alterations (CNAs) and selected structural rearrangements. List of target regions in 7 probe groups (7 promotor-regions, IKP SNPs, QC SNPs and regions, selected fusions, 678 selected genes, CNV SNPs, 279 hotspots). Total size: 3.868 Mbp.

File Name: Supplementary Data 3
Description: Custom gene panel ssSCv4 designed to detect somatic mutations (SNVs), small insertions and deletions (INDELs), copy number alterations (CNAs) and selected structural rearrangements. List of target regions in 7 probe groups (7 promotor-regions, IKP SNPs, QC SNPs and regions, selected fusions, 693 selected genes, CNV SNPs, 279 hotspots). Total size: 3.929 Mbp.
