# Supplementary material for: Cancer immune control needs senescence induction by interferon-dependent cell cycle regulator pathways in tumours
Source: Nat Commun. 2020 Mar 12;11:1335. doi: 10.1038/s41467-020-14987-6 (PMC7067802; doi:10.1038/s41467-020-14987-6)
Supplement: Supplementary file 4 — Supplementary Data 1 [file 41467_2020_14987_MOESM4_ESM.pdf]

Supplementary Data 1 Custom gene panel ssSCv2

Chr	start	end	Gene
chr1	8073244	8074481	ERRFI1
chr1	8075342	8075469	ERRFI1
chr1	8075529	8075704	ERRFI1
chr1	11166636	11166864	MTOR
chr1	11166971	11167206	MTOR
chr1	11167516	11167582	MTOR
chr1	11168212	11168368	MTOR
chr1	11169321	11169452	MTOR
chr1	11169680	11169811	MTOR
chr1	11171159	11171213	MTOR
chr1	11172883	11172999	MTOR
chr1	11174349	11174535	MTOR
chr1	11174844	11174969	MTOR
chr1	11175427	11175550	MTOR
chr1	11177035	11177168	MTOR
chr1	11181277	11181450	MTOR
chr1	11182010	11182208	MTOR
chr1	11184529	11184715	MTOR
chr1	11186653	11186878	MTOR
chr1	11187041	11187226	MTOR
chr1	11187655	11187888	MTOR
chr1	11188035	11188208	MTOR
chr1	11188485	11188634	MTOR
chr1	11188886	11189033	MTOR
chr1	11189769	11189920	MTOR
chr1	11190560	11190859	MTOR
chr1	11193111	11193279	MTOR
chr1	11194382	11194548	MTOR
chr1	11199335	11199517	MTOR
chr1	11199564	11199740	MTOR
chr1	11204679	11204837	MTOR, MTOR-AS1
chr1	11204999	11205127	MTOR, MTOR-AS1
chr1	11206707	11206873	MTOR, MTOR-AS1
chr1	11210157	11210308	MTOR
chr1	11217183	11217373	MTOR
chr1	11227473	11227599	MTOR
chr1	11259289	11259485	MTOR
chr1	11259572	11259785	MTOR
chr1	11264592	11264785	MTOR
chr1	11269343	11269540	MTOR
chr1	11270845	11270988	MTOR
chr1	11272343	11272556	MTOR
chr1	11272827	11272990	MTOR
chr1	11273430	11273648	MTOR
chr1	11276179	11276316	MTOR
chr1	11288699	11289000	MTOR
chr1	11290956	11291136	MTOR
chr1	11291331	11291516	MTOR
chr1	11292467	11292610	MTOR
chr1	11293429	11293569	MTOR
chr1	11294174	11294347	MTOR
chr1	11297874	11298130	MTOR
chr1	11298433	11298699	MTOR
chr1	11300334	11300629	MTOR
chr1	11301584	11301763	MTOR
chr1	11303145	11303382	MTOR
chr1	11307656	11307815	MTOR
chr1	11307850	11308176	MTOR
chr1	11313870	11314055	MTOR
chr1	11316023	11316274	MTOR
chr1	11316964	11317247	MTOR
chr1	11318516	11318675	MTOR
chr1	11319279	11319491	MTOR
chr1	11850711	11850980	MTHFR
chr1	11851238	11851408	MTHFR
chr1	11852309	11852461	MTHFR
chr1	11853938	11854171	MTHFR
chr1	11854389	11854620	MTHFR
chr1	11854760	11854945	MTHFR
chr1	11855129	11855430	MTHFR
chr1	11856237	11856481	MTHFR
chr1	11860243	11860404	MTHFR
chr1	11861192	11861481	MTHFR
chr1	11862912	11863211	MTHFR
chr1	11863436	11863596	MTHFR
chr1	27022869	27024056	ARID1A
chr1	27056116	27056379	ARID1A
chr1	27057617	27058120	ARID1A
chr1	27059141	27059308	ARID1A
chr1	27087321	27087612	ARID1A
chr1	27087849	27087989	ARID1A
chr1	27088617	27088835	ARID1A
chr1	27089438	27089801	ARID1A
chr1	27092686	27092882	ARID1A
chr1	27092922	27093082	ARID1A
chr1	27094255	27094515	ARID1A
chr1	27097584	27097842	ARID1A
chr1	27098965	27099148	ARID1A
chr1	27099277	27099503	ARID1A
chr1	27099811	27100012	ARID1A

chr1	27100045	27100233	ARID1A
chr1	27100267	27100414	ARID1A
chr1	27100794	27101736	ARID1A
chr1	27102042	27102223	ARID1A
chr1	27105488	27107272	ARID1A
chr1	36931671	36931826	CSF3R
chr1	36931932	36932534	CSF3R
chr1	36932805	36932937	CSF3R
chr1	36933133	36933277	CSF3R
chr1	36933397	36933588	CSF3R
chr1	36933650	36933847	CSF3R
chr1	36934731	36934883	CSF3R
chr1	36935227	36935466	CSF3R
chr1	36937008	36937272	CSF3R
chr1	36937641	36937765	CSF3R
chr1	36937813	36938017	CSF3R
chr1	36938092	36938312	CSF3R
chr1	36939010	36939248	CSF3R
chr1	36939339	36939513	CSF3R
chr1	36940952	36941299	CSF3R
chr1	36945008	36945122	CSF3R
chr1	40363018	40363667	MYCL
chr1	40366460	40367140	MYCL
chr1	40367454	40367585	MYCL
chr1	43803494	43803623	MPL
chr1	43803744	43803927	MPL
chr1	43804187	43804416	MPL
chr1	43804916	43805265	MPL
chr1	43805609	43805822	MPL
chr1	43806032	43806209	MPL
chr1	43812090	43812325	MPL
chr1	43812437	43812630	MPL
chr1	43814488	43814698	MPL
chr1	43814908	43815140	MPL
chr1	43817861	43817999	MPL
chr1	43818163	43818468	MPL
chr1	45286335	45286401	PTCH2
chr1	45288061	45288366	PTCH2
chr1	45288715	45288865	PTCH2
chr1	45288889	45289082	PTCH2
chr1	45291896	45292084	PTCH2
chr1	45292134	45292465	PTCH2
chr1	45292548	45292779	PTCH2
chr1	45292813	45293006	PTCH2
chr1	45293048	45293411	PTCH2
chr1	45293489	45293888	PTCH2
chr1	45293942	45294111	PTCH2
chr1	45294152	45294328	PTCH2
chr1	45294628	45294771	PTCH2
chr1	45294803	45295009	PTCH2
chr1	45295048	45295230	PTCH2
chr1	45295260	45295458	PTCH2
chr1	45295555	45295727	PTCH2
chr1	45296494	45296740	PTCH2
chr1	45297352	45297494	PTCH2
chr1	45297621	45297741	PTCH2
chr1	45297798	45298038	PTCH2
chr1	45307493	45307736	PTCH2
chr1	45308507	45308629	PTCH2
chr1	45794952	45795134	MUTYH
chr1	45796162	45796254	MUTYH
chr1	45796828	45797031	MUTYH
chr1	45797066	45797253	MUTYH
chr1	45797307	45797546	MUTYH
chr1	45797669	45797783	MUTYH
chr1	45797812	45798007	MUTYH
chr1	45798037	45798185	MUTYH
chr1	45798220	45798384	MUTYH
chr1	45798409	45798531	MUTYH
chr1	45798564	45798656	MUTYH
chr1	45798743	45798867	MUTYH
chr1	45798931	45799021	MUTYH
chr1	45799059	45799300	MUTYH
chr1	45800037	45800208	MUTYH
chr1	45805865	45805951	MUTYH, TOE1
chr1	51436015	51436194	CDKN2C
chr1	51439539	51439967	CDKN2C
chr1	59247721	59248767	JUN
chr1	60359297	60359526	CYP2J2
chr1	60366611	60366800	CYP2J2
chr1	60370517	60370755	CYP2J2
chr1	60373432	60373624	CYP2J2
chr1	60375430	60375657	CYP2J2
chr1	60377254	60377465	CYP2J2
chr1	60377808	60378008	CYP2J2
chr1	60381584	60381797	CYP2J2
chr1	60392183	60392443	CYP2J2
chr1	65300219	65300365	JAK1
chr1	65301053	65301214	JAK1
chr1	65301755	65301923	JAK1
chr1	65303589	65303812	JAK1
chr1	65304122	65304297	JAK1
chr1	65305260	65305503	JAK1
chr1	65306902	65307047	JAK1
chr1	65307108	65307309	JAK1
chr1	65309721	65309923	JAK1

chr1	65310411	65310597	JAK1
chr1	65311170	65311348	JAK1
chr1	65312306	65312444	JAK1
chr1	65313189	65313383	JAK1
chr1	65316461	65316618	JAK1
chr1	65321166	65321406	JAK1
chr1	65323313	65323487	JAK1
chr1	65325762	65325970	JAK1
chr1	65330444	65330680	JAK1
chr1	65332523	65332916	JAK1
chr1	65334968	65335182	JAK1
chr1	65339027	65339231	JAK1
chr1	65344682	65344856	JAK1
chr1	65348934	65349183	JAK1
chr1	65351916	65351972	JAK1
chr1	78413173	78413262	
chr1	78414425	78414484	FUBP1
chr1	78414532	78414624	FUBP1
chr1	78414814	78415010	FUBP1
chr1	78420914	78421042	FUBP1
chr1	78422231	78422410	FUBP1
chr1	78425843	78425973	FUBP1
chr1	78426003	78426205	FUBP1
chr1	78428429	78428640	FUBP1
chr1	78429233	78429425	FUBP1
chr1	78429721	78429872	FUBP1
chr1	78429913	78430066	FUBP1
chr1	78430305	78430457	FUBP1
chr1	78430529	78430678	FUBP1
chr1	78430727	78430940	FUBP1
chr1	78432352	78432460	FUBP1
chr1	78432542	78432664	FUBP1
chr1	78432707	78432810	FUBP1
chr1	78433285	78433375	FUBP1
chr1	78433823	78433912	FUBP1
chr1	78434680	78434793	FUBP1
chr1	78435583	78435724	FUBP1
chr1	78444543	78444713	FUBP1
chr1	97544506	97544727	DPYD
chr1	97547860	97548051	DPYD
chr1	97564019	97564213	DPYD, DPYD-AS1
chr1	97658599	97658829	DPYD, DPYD-AS1
chr1	97700382	97700575	DPYD, DPYD-AS1
chr1	97770789	97770959	DPYD, DPYD-AS1
chr1	97771707	97771878	DPYD, DPYD-AS1
chr1	97839091	97839225	DPYD
chr1	97847923	97848042	DPYD
chr1	97915589	97915804	DPYD
chr1	97981256	97981522	DPYD
chr1	98015090	98015325	DPYD
chr1	98039290	98039551	DPYD
chr1	98058748	98058968	DPYD
chr1	98060589	98060747	DPYD
chr1	98144625	98144763	DPYD
chr1	98157247	98157379	DPYD
chr1	98164881	98165128	DPYD
chr1	98165734	98165883	DPYD
chr1	98186418	98186507	DPYD
chr1	98187040	98187252	DPYD
chr1	98205922	98206060	DPYD
chr1	98293644	98293777	DPYD
chr1	98348794	98348955	DPYD
chr1	98386414	98386503	DPYD
chr1	115251130	115251300	NRAS
chr1	115252164	115252374	NRAS
chr1	115256395	115256624	NRAS
chr1	115258645	115258806	NRAS
chr1	118165465	118166691	FAM46C
chr1	120457903	120459342	NOTCH2
chr1	120460262	120460410	NOTCH2
chr1	120461003	120461201	NOTCH2
chr1	120461909	120462261	NOTCH2
chr1	120462826	120463045	NOTCH2
chr1	120464310	120464457	NOTCH2
chr1	120464833	120465094	NOTCH2
chr1	120465233	120465426	NOTCH2
chr1	120466234	120466632	NOTCH2
chr1	120467902	120468458	NOTCH2
chr1	120469096	120469259	NOTCH2
chr1	120471573	120471860	NOTCH2
chr1	120478016	120478252	NOTCH2
chr1	120479879	120480114	NOTCH2
chr1	120480454	120480658	NOTCH2
chr1	120483152	120483404	NOTCH2
chr1	120484123	120484402	NOTCH2
chr1	120491011	120491214	NOTCH2
chr1	120491604	120491774	NOTCH2
chr1	120493091	120493485	NOTCH2
chr1	120496140	120496336	NOTCH2
chr1	120497637	120497880	NOTCH2
chr1	120501989	120502150	NOTCH2
chr1	120506171	120506455	NOTCH2
chr1	120508050	120508214	NOTCH2
chr1	120508973	120509137	NOTCH2
chr1	120510030	120510269	NOTCH2
chr1	120510674	120510880	NOTCH2

chr1	120512108	120512392	NOTCH2
chr1	120529557	120529730	NOTCH2
chr1	120534059	120534130	NOTCH2
chr1	120534206	120534281	NOTCH2
chr1	120539263	120539394	NOTCH2
chr1	120539398	120539451	NOTCH2
chr1	120539594	120539980	NOTCH2
chr1	120547926	120548236	NOTCH2
chr1	120572503	120572635	NOTCH2
chr1	120611371	120612100	NOTCH2
chr1	150549814	150549992	MCL1
chr1	150550694	150550992	MCL1
chr1	150551293	150552031	MCL1
chr1	155158585	155158710	MUC1
chr1	155159282	155159392	MUC1
chr1	155159675	155159875	MUC1
chr1	155159905	155160077	MUC1
chr1	155160172	155160359	MUC1
chr1	155160458	155160564	MUC1
chr1	155160613	155161453	MUC1
chr1	155161476	155161555	MUC1
chr1	155161673	155162126	MUC1
chr1	155162551	155162659	MUC1
chr1	156785596	156785655	NTRK1, SH2D2A
chr1	156811847	156812010	INSRR, NTRK1
chr1	156830701	156830963	NTRK1
chr1	156834120	156834245	NTRK1
chr1	156834494	156834616	NTRK1
chr1	156836676	156836795	NTRK1
chr1	156837870	156838066	NTRK1
chr1	156838271	156838464	NTRK1
chr1	156841389	156841572	NTRK1
chr1	156843399	156843776	NTRK1
chr1	156844149	156844217	NTRK1
chr1	156844337	156844443	NTRK1
chr1	156844672	156844825	NTRK1
chr1	156845286	156845483	NTRK1
chr1	156845837	156846027	NTRK1
chr1	156846166	156846389	NTRK1
chr1	156848888	156849179	NTRK1
chr1	156849765	156849974	NTRK1
chr1	156851223	156851459	NTRK1
chr1	162688828	162688960	DDR2
chr1	162722859	162723012	DDR2
chr1	162724388	162724670	DDR2
chr1	162724920	162725118	DDR2
chr1	162725428	162725584	DDR2
chr1	162729560	162729794	DDR2
chr1	162730975	162731269	DDR2
chr1	162735765	162735878	DDR2
chr1	162736993	162737174	DDR2
chr1	162740066	162740327	DDR2
chr1	162741788	162742062	DDR2
chr1	162743233	162743411	DDR2
chr1	162745416	162745658	DDR2
chr1	162745900	162746185	DDR2
chr1	162748344	162748544	DDR2
chr1	162749876	162750061	DDR2
chr1	169483525	169483722	F5
chr1	169484656	169484889	F5
chr1	169487624	169487826	F5
chr1	169489732	169489927	F5
chr1	169492409	169492615	F5
chr1	169493013	169493167	F5
chr1	169494049	169494171	F5
chr1	169495113	169495280	F5
chr1	169497127	169497357	F5
chr1	169498820	169499081	F5
chr1	169499998	169500285	F5
chr1	169505718	169505943	F5
chr1	169509506	169512377	F5
chr1	169513508	169513786	F5
chr1	169515654	169515855	F5
chr1	169519013	169519278	F5
chr1	169519852	169520002	F5
chr1	169521769	169521997	F5
chr1	169524394	169524610	F5
chr1	169525858	169526130	F5
chr1	169528365	169528559	F5
chr1	169529766	169530029	F5
chr1	169541433	169541606	F5
chr1	169551643	169551785	F5
chr1	169555441	169555649	F5
chr1	179076827	179078601	ABL2
chr1	179079391	179079615	ABL2
chr1	179079914	179080005	ABL2
chr1	179081418	179081558	ABL2
chr1	179083987	179084190	ABL2
chr1	179086373	179086676	ABL2
chr1	179087696	179087924	ABL2
chr1	179089299	179089434	ABL2
chr1	179090704	179091027	ABL2
chr1	179095486	179095832	ABL2
chr1	179100420	179100641	ABL2
chr1	179102421	179102534	ABL2
chr1	179112042	179112204	ABL2

chr1	179198350	179198557	ABL2
chr1	183711267	183711449	RGL1
chr1	183774406	183774483	RGL1
chr1	183775483	183775644	RGL1
chr1	183816674	183816933	RGL1
chr1	183835104	183835232	RGL1
chr1	183849724	183849959	RGL1
chr1	183852894	183853069	RGL1
chr1	183853831	183854097	RGL1
chr1	183857582	183857736	RGL1
chr1	183861185	183861320	RGL1
chr1	183866916	183867056	RGL1
chr1	183869258	183869395	RGL1
chr1	183871676	183871759	RGL1
chr1	183873958	183874130	RGL1
chr1	183876120	183876260	RGL1
chr1	183881190	183881427	RGL1
chr1	183885553	183885860	RGL1
chr1	183891330	183891495	RGL1
chr1	183895213	183895451	RGL1
chr1	186643459	186643919	PTGS2
chr1	186644355	186644553	PTGS2
chr1	186645004	186645341	PTGS2
chr1	186645573	186645870	PTGS2
chr1	186645939	186646073	PTGS2
chr1	186646755	186646987	PTGS2
chr1	186647367	186647561	PTGS2
chr1	186648164	186648358	PTGS2
chr1	186648428	186648595	PTGS2
chr1	186649345	186649447	PTGS2
chr1	193091305	193091486	CDC73
chr1	193094216	193094372	CDC73
chr1	193099278	193099398	CDC73
chr1	193104495	193104608	CDC73
chr1	193104641	193104744	CDC73
chr1	193107189	193107328	CDC73
chr1	193110954	193111221	CDC73
chr1	193116971	193117120	CDC73
chr1	193119408	193119537	CDC73
chr1	193121484	193121599	CDC73
chr1	193172899	193173007	CDC73
chr1	193181169	193181255	CDC73
chr1	193181494	193181632	CDC73
chr1	193202097	193202309	CDC73
chr1	193205360	193205511	CDC73
chr1	193218834	193219026	CDC73
chr1	193219780	193219867	CDC73
chr1	204494621	204494749	MDM4
chr1	204495462	204495587	MDM4
chr1	204499786	204499970	MDM4
chr1	204501293	204501399	MDM4
chr1	204501871	204501972	MDM4
chr1	204506020	204506072	MDM4
chr1	204506532	204506650	MDM4
chr1	204507311	204507461	MDM4
chr1	204511886	204512097	MDM4
chr1	204513637	204513837	MDM4
chr1	204515899	204516030	MDM4
chr1	204518215	204518835	MDM4
chr1	204527743	204527834	MDM4
chr1	206646545	206646682	IKBKE
chr1	206647648	206647839	IKBKE
chr1	206648182	206648362	IKBKE
chr1	206649498	206649730	IKBKE
chr1	206649995	206650206	IKBKE
chr1	206651066	206651227	IKBKE
chr1	206651477	206651707	IKBKE
chr1	206652260	206652501	IKBKE
chr1	206653187	206653302	IKBKE
chr1	206653339	206653481	IKBKE
chr1	206653764	206653901	IKBKE
chr1	206658308	206658434	IKBKE
chr1	206658505	206658668	IKBKE
chr1	206661225	206661352	IKBKE
chr1	206664126	206664216	IKBKE
chr1	206664955	206665107	C1ORF147, IKBKE
chr1	206666330	206666477	C1ORF147, IKBKE
chr1	206666573	206666736	C1ORF147, IKBKE
chr1	206667227	206667349	C1ORF147, IKBKE
chr1	206669419	206669503	C1ORF147, IKBKE
chr1	226252027	226252205	H3F3A
chr1	226253331	226253625	H3F3A
chr1	226259026	226259205	H3F3A
chr1	243663019	243663113	AKT3, SDCCAG8
chr1	243668525	243668661	AKT3
chr1	243675600	243675753	AKT3
chr1	243708786	243708924	AKT3
chr1	243716005	243716270	AKT3
chr1	243726996	243727175	AKT3
chr1	243736202	243736375	AKT3
chr1	243776947	243777066	AKT3
chr1	243778372	243778488	AKT3
chr1	243800887	243801069	AKT3
chr1	243809169	243809364	AKT3
chr1	243828048	243828210	AKT3
chr1	243858867	243859043	AKT3

chr1	244006401	244006497	AKT3
chr2	16082161	16083001	MYCN, MYCNOS
chr2	16085589	16086244	MYCN
chr2	25457122	25457314	DNMT3A
chr2	25458550	25458719	DNMT3A
chr2	25459779	25459899	DNMT3A
chr2	25461973	25462109	DNMT3A
chr2	25463145	25463344	DNMT3A
chr2	25463483	25463624	DNMT3A
chr2	25464405	25464601	DNMT3A
chr2	25466741	25466876	DNMT3A
chr2	25466998	25467232	DNMT3A
chr2	25467383	25467546	DNMT3A
chr2	25468096	25468226	DNMT3A
chr2	25468863	25468958	DNMT3A
chr2	25469003	25469203	DNMT3A
chr2	25469463	25469670	DNMT3A
chr2	25469894	25470052	DNMT3A
chr2	25470434	25470643	DNMT3A
chr2	25470880	25471146	DNMT3A
chr2	25472500	25472618	DNMT3A
chr2	25475037	25475091	DNMT3A
chr2	25497784	25497981	DNMT3A
chr2	25498343	25498437	DNMT3A
chr2	25505231	25505605	DNMT3A
chr2	25522982	25523137	DNMT3A
chr2	25536756	25536878	DNMT3A
chr2	29416064	29416813	ALK
chr2	29419610	29419751	ALK
chr2	29420382	29420567	ALK
chr2	29430011	29430163	ALK
chr2	29430885	29430936	ALK
chr2	29432626	29432769	ALK
chr2	29436824	29436972	ALK
chr2	29443546	29443726	ALK
chr2	29445184	29445299	ALK
chr2	29445357	29445498	ALK
chr2	29446182	29446419	ALK
chr2	29448301	29448456	ALK
chr2	29449762	29449965	ALK
chr2	29450414	29450563	ALK
chr2	29451724	29451957	ALK
chr2	29455144	29455339	ALK
chr2	29456405	29456587	ALK
chr2	29462520	29462721	ALK
chr2	29473945	29474158	ALK
chr2	29497939	29498118	ALK
chr2	29498242	29498387	ALK
chr2	29519728	29519948	ALK
chr2	29541144	29541295	ALK
chr2	29543591	29543773	ALK
chr2	29551190	29551372	ALK
chr2	29606572	29606750	ALK
chr2	29754755	29755007	ALK
chr2	29917690	29917905	ALK
chr2	29940414	29940588	ALK
chr2	30142833	30143550	ALK
chr2	47630305	47630566	MSH2
chr2	47635514	47635719	MSH2
chr2	47637207	47637536	MSH2
chr2	47639527	47639724	MSH2
chr2	47641382	47641582	MSH2
chr2	47643409	47643593	MSH2
chr2	47656855	47657105	MSH2
chr2	47672661	47672821	MSH2
chr2	47690144	47690318	MSH2
chr2	47691393	47691490	MSH2
chr2	47693771	47693972	MSH2
chr2	47698078	47698226	MSH2
chr2	47702138	47702434	MSH2
chr2	47703480	47703735	MSH2
chr2	47705385	47705683	MSH2
chr2	47707809	47708035	MSH2
chr2	47709892	47710113	MSH2
chr2	47739416	47739598	MSH2
chr2	48010347	48010657	MSH6
chr2	48018040	48018287	FBXO11, MSH6
chr2	48023007	48023227	FBXO11, MSH6
chr2	48025724	48028354	FBXO11, MSH6
chr2	48030533	48030849	FBXO11, MSH6
chr2	48032023	48032191	FBXO11, MSH6
chr2	48032731	48032871	FBXO11, MSH6
chr2	48033317	48033522	FBXO11, MSH6
chr2	48033565	48033815	FBXO11, MSH6
chr2	48033892	48034024	FBXO11, MSH6
chr2	58386874	58386960	FANCL, VRK2
chr2	58387217	58387339	FANCL
chr2	58388631	58388798	FANCL
chr2	58389975	58390107	FANCL
chr2	58390138	58390234	FANCL
chr2	58390543	58390677	FANCL
chr2	58392833	58393034	FANCL
chr2	58425688	58425822	FANCL
chr2	58431239	58431386	FANCL
chr2	58449051	58449202	FANCL
chr2	58453837	58453944	FANCL

chr2	58456923	58457034	FANCL
chr2	58459163	58459272	FANCL
chr2	58468327	58468473	FANCL
chr2	140990729	140990920	LRP1B
chr2	140992329	140992478	LRP1B
chr2	140995695	140995890	LRP1B
chr2	140996985	140997126	LRP1B
chr2	141004629	141004756	LRP1B
chr2	141027785	141027940	LRP1B
chr2	141031967	141032192	LRP1B
chr2	141055351	141055563	LRP1B
chr2	141072478	141072693	LRP1B
chr2	141079506	141079682	LRP1B
chr2	141081436	141081660	LRP1B
chr2	141083305	141083472	LRP1B
chr2	141091996	141092153	LRP1B
chr2	141093158	141093432	LRP1B
chr2	141108340	141108632	LRP1B
chr2	141110496	141110666	LRP1B
chr2	141113885	141114070	LRP1B
chr2	141115522	141115710	LRP1B
chr2	141116364	141116540	LRP1B
chr2	141122204	141122377	LRP1B
chr2	141128253	141128436	LRP1B
chr2	141128722	141128879	LRP1B
chr2	141130551	141130731	LRP1B
chr2	141135723	141135880	LRP1B
chr2	141143436	141143603	LRP1B
chr2	141200047	141200217	LRP1B
chr2	141201873	141202043	LRP1B
chr2	141202106	141202273	LRP1B
chr2	141208111	141208255	LRP1B
chr2	141213998	141214197	LRP1B
chr2	141215006	141215245	LRP1B
chr2	141232681	141232931	LRP1B
chr2	141242886	141243118	LRP1B
chr2	141245160	141245333	LRP1B
chr2	141250151	141250287	LRP1B
chr2	141253108	141253342	LRP1B
chr2	141259230	141259468	LRP1B
chr2	141260506	141260697	LRP1B
chr2	141264339	141264512	LRP1B
chr2	141267471	141267650	LRP1B
chr2	141272196	141272366	LRP1B
chr2	141274432	141274605	LRP1B
chr2	141283387	141283587	LRP1B
chr2	141283780	141283944	LRP1B
chr2	141291564	141291734	LRP1B
chr2	141294124	141294303	LRP1B
chr2	141298516	141298692	LRP1B
chr2	141299322	141299565	LRP1B
chr2	141356174	141356429	LRP1B
chr2	141358993	141359233	LRP1B
chr2	141457793	141458215	LRP1B
chr2	141459264	141459439	LRP1B
chr2	141459684	141459886	LRP1B
chr2	141459970	141460147	LRP1B
chr2	141473516	141473696	LRP1B
chr2	141474225	141474410	LRP1B
chr2	141526756	141526938	LRP1B
chr2	141528424	141528600	LRP1B
chr2	141533641	141533832	LRP1B
chr2	141571200	141571400	LRP1B
chr2	141597534	141597679	LRP1B
chr2	141598461	141598686	LRP1B
chr2	141607645	141607923	LRP1B
chr2	141609195	141609377	LRP1B
chr2	141625133	141625428	LRP1B
chr2	141625642	141625857	LRP1B
chr2	141641360	141641615	LRP1B
chr2	141643681	141643929	LRP1B
chr2	141660463	141660759	LRP1B
chr2	141665420	141665671	LRP1B
chr2	141680508	141680741	LRP1B
chr2	141707778	141707996	LRP1B
chr2	141709403	141709534	LRP1B
chr2	141739703	141739870	LRP1B
chr2	141747075	141747251	LRP1B
chr2	141751538	141751729	LRP1B
chr2	141762878	141763051	LRP1B
chr2	141771099	141771339	LRP1B
chr2	141773239	141773509	LRP1B
chr2	141777465	141777696	LRP1B
chr2	141806529	141806816	LRP1B
chr2	141812659	141812853	LRP1B
chr2	141816426	141816648	LRP1B
chr2	141819594	141819867	LRP1B
chr2	141945964	141946177	LRP1B
chr2	141986726	141987034	LRP1B
chr2	142004769	142004948	LRP1B
chr2	142012065	142012235	LRP1B
chr2	142044303	142044376	LRP1B
chr2	142237908	142238144	LRP1B
chr2	142567822	142567995	LRP1B
chr2	142888191	142888323	LRP1B
chr2	178095487	178096761	NFE2L2

chr2	178097094	178097336	NFE2L2
chr2	178097880	178098092	NFE2L2
chr2	178098707	178099024	NFE2L2
chr2	178129234	178129329	NFE2L2
chr2	198257001	198257210	SF3B1
chr2	198257670	198257937	SF3B1
chr2	198260754	198261077	SF3B1
chr2	198262683	198262865	SF3B1
chr2	198263159	198263330	SF3B1
chr2	198264753	198264915	SF3B1
chr2	198264950	198265183	SF3B1
chr2	198265413	198265685	SF3B1
chr2	198266098	198266274	SF3B1
chr2	198266440	198266637	SF3B1
chr2	198266683	198266879	SF3B1
chr2	198267254	198267575	SF3B1
chr2	198267647	198267784	SF3B1
chr2	198268283	198268513	SF3B1
chr2	198269774	198269926	SF3B1
chr2	198269973	198270221	SF3B1
chr2	198272696	198272868	SF3B1
chr2	198273067	198273330	SF3B1
chr2	198274468	198274756	SF3B1
chr2	198281439	198281660	SF3B1
chr2	198283207	198283337	SF3B1
chr2	198283630	198283700	SF3B1
chr2	198285076	198285291	SF3B1
chr2	198285727	198285882	SF3B1
chr2	198288506	198288723	SF3B1
chr2	198299670	198299748	SF3B1
chr2	209101777	209101918	IDH1
chr2	209103769	209103982	IDH1
chr2	209104561	209104752	IDH1
chr2	209106692	209106894	IDH1
chr2	209108125	209108353	IDH1
chr2	209110017	209110173	IDH1
chr2	209113067	209113409	IDH1
chr2	209116128	209116300	IDH1
chr2	212248314	212248810	ERBB4
chr2	212251552	212251900	ERBB4
chr2	212252644	212252742	ERBB4
chr2	212285140	212285361	ERBB4
chr2	212286706	212286854	ERBB4
chr2	212288854	212289051	ERBB4
chr2	212293107	212293233	ERBB4
chr2	212295644	212295850	ERBB4
chr2	212426602	212426838	ERBB4
chr2	212483876	212484025	ERBB4
chr2	212488621	212488794	ERBB4
chr2	212495161	212495344	ERBB4
chr2	212522453	212522578	ERBB4
chr2	212522649	212522744	ERBB4
chr2	212530022	212530227	ERBB4
chr2	212537863	212538007	ERBB4
chr2	212543751	212543934	ERBB4
chr2	212566666	212566916	ERBB4
chr2	212568803	212568944	ERBB4
chr2	212570017	212570141	ERBB4
chr2	212576749	212576926	ERBB4
chr2	212578234	212578398	ERBB4
chr2	212587092	212587284	ERBB4
chr2	212589775	212589944	ERBB4
chr2	212615338	212615454	ERBB4
chr2	212652724	212652909	ERBB4
chr2	212812129	212812366	ERBB4
chr2	212989451	212989653	ERBB4
chr2	213403147	213403279	ERBB4
chr2	215593374	215593757	BARD1
chr2	215595109	215595257	BARD1
chr2	215595606	215595672	BARD1
chr2	215609765	215609908	BARD1
chr2	215610420	215610603	BARD1
chr2	215617145	215617304	BARD1
chr2	215632180	215632403	BARD1
chr2	215633930	215634061	BARD1
chr2	215645258	215646258	BARD1
chr2	215656995	215657194	BARD1
chr2	215661759	215661866	BARD1
chr2	215674110	215674318	BARD1
chr2	234668908	234669822	UGT1A1, UGT1A10, UGT1A3, UGT1A4, UGT1A5, UGT1A6, UGT1A7, UGT1A8, UGT1A9
chr2	234675654	234675836	UGT1A1, UGT1A10, UGT1A3, UGT1A4, UGT1A5, UGT1A6, UGT1A7, UGT1A8, UGT1A9
chr2	234676469	234676607	UGT1A1, UGT1A10, UGT1A3, UGT1A4, UGT1A5, UGT1A6, UGT1A7, UGT1A8, UGT1A9
chr2	234676840	234677110	UGT1A1, UGT1A10, UGT1A3, UGT1A4, UGT1A5, UGT1A6, UGT1A7, UGT1A8, UGT1A9
chr2	234678151	234678232	UGT1A1, UGT1A10, UGT1A3, UGT1A4, UGT1A5, UGT1A6, UGT1A7, UGT1A8, UGT1A9
chr2	234680882	234681230	UGT1A1, UGT1A10, UGT1A3, UGT1A4, UGT1A5, UGT1A6, UGT1A7, UGT1A8, UGT1A9
chr3	10070316	10070430	FANCD2
chr3	10074490	10074681	FANCD2
chr3	10076127	10076245	FANCD2
chr3	10076353	10076507	FANCD2
chr3	10076831	10076942	FANCD2
chr3	10077945	10078048	FANCD2
chr3	10080937	10081066	FANCD2
chr3	10081379	10081585	FANCD2
chr3	10083281	10083419	FANCD2
chr3	10084217	10084372	FANCD2
chr3	10084708	10084859	FANCD2

chr3	10085142	10085301	FANCD2
chr3	10085487	10085573	FANCD2
chr3	10088238	10088432	FANCD2
chr3	10089575	10089760	FANCD2
chr3	10091032	10091214	FANCD2
chr3	10094045	10094206	FANCD2
chr3	10101952	10102112	FANCD2
chr3	10103809	10103920	FANCD2
chr3	10105450	10105620	FANCD2
chr3	10106014	10106138	FANCD2
chr3	10106387	10106584	FANCD2
chr3	10107052	10107203	FANCD2
chr3	10107522	10107688	FANCD2
chr3	10108867	10109026	FANCD2
chr3	10114529	10114690	FANCD2
chr3	10114911	10115071	FANCD2
chr3	10116188	10116382	FANCD2
chr3	10119739	10119906	FANCD2
chr3	10122758	10122937	FANCD2
chr3	10123004	10123173	FANCD2, FANCD2OS
chr3	10127470	10127631	FANCD2, FANCD2OS
chr3	10128792	10128973	FANCD2, FANCD2OS
chr3	10130107	10130251	FANCD2, FANCD2OS
chr3	10130486	10130659	FANCD2, FANCD2OS
chr3	10131950	10132094	FANCD2, FANCD2OS
chr3	10133839	10133961	FANCD2, FANCD2OS
chr3	10134943	10135032	FANCD2, FANCD2OS
chr3	10135947	10136072	FANCD2, FANCD2OS
chr3	10136858	10136983	FANCD2, FANCD2OS
chr3	10137984	10138181	FANCD2, FANCD2OS
chr3	10140378	10140659	FANCD2, FANCD2OS
chr3	10142846	10142971	FANCD2, FANCD2OS
chr3	10183506	10183896	VHL
chr3	10188172	10188345	VHL
chr3	10191445	10191674	VHL
chr3	12625987	12626181	RAF1
chr3	12626320	12626505	RAF1
chr3	12626595	12626777	RAF1
chr3	12627154	12627323	RAF1
chr3	12629064	12629161	RAF1
chr3	12632271	12632498	RAF1
chr3	12633181	12633316	RAF1
chr3	12641164	12641332	RAF1
chr3	12641625	12641803	RAF1
chr3	12641861	12641939	RAF1
chr3	12644951	12645061	RAF1
chr3	12645609	12645813	RAF1
chr3	12647674	12647823	RAF1
chr3	12650239	12650447	RAF1
chr3	12650706	12650859	RAF1
chr3	12653423	12653586	RAF1
chr3	12659988	12660245	RAF1
chr3	30648350	30648494	TGFBR2
chr3	30664665	30664790	TGFBR2
chr3	30686213	30686432	TGFBR2
chr3	30691736	30691977	TGFBR2
chr3	30702998	30703156	TGFBR2
chr3	30713104	30713954	TGFBR2
chr3	30715571	30715763	TGFBR2
chr3	30729850	30730028	TGFBR2
chr3	30732886	30733116	TGFBR2
chr3	37035013	37035179	MLH1
chr3	37038084	37038225	MLH1
chr3	37042420	37042569	MLH1
chr3	37045866	37045990	MLH1
chr3	37048456	37048579	MLH1
chr3	37050279	37050421	MLH1
chr3	37053285	37053378	MLH1
chr3	37053476	37053615	MLH1
chr3	37055897	37056060	MLH1
chr3	37058971	37059115	MLH1
chr3	37061775	37061979	MLH1
chr3	37067102	37067523	MLH1
chr3	37070249	37070448	MLH1
chr3	37081651	37081810	MLH1
chr3	37083727	37083847	MLH1
chr3	37088984	37089199	MLH1
chr3	37089982	37090125	MLH1
chr3	37090369	37090533	MLH1
chr3	37091951	37092169	MLH1
chr3	37107082	37107135	LRRFIP2, MLH1
chr3	38180127	38180544	MYD88
chr3	38181329	38181514	MYD88
chr3	38181853	38182108	MYD88
chr3	38182222	38182364	MYD88
chr3	38182597	38182802	MYD88
chr3	38591786	38593074	SCN5A
chr3	38595744	38596065	SCN5A
chr3	38597121	38597276	SCN5A
chr3	38597906	38598094	SCN5A
chr3	38598696	38598800	SCN5A
chr3	38601612	38601944	SCN5A
chr3	38603880	38604053	SCN5A
chr3	38607874	38608098	SCN5A
chr3	38616762	38616967	SCN5A
chr3	38618126	38618297	SCN5A

chr3	38620799	38621011	SCN5A
chr3	38622396	38622887	SCN5A
chr3	38627156	38627557	SCN5A
chr3	38628865	38629089	SCN5A
chr3	38639194	38639483	SCN5A
chr3	38640383	38640566	SCN5A
chr3	38645177	38645599	SCN5A
chr3	38646194	38646424	SCN5A
chr3	38647416	38647664	SCN5A
chr3	38648134	38648326	SCN5A
chr3	38649616	38649730	SCN5A
chr3	38651199	38651480	SCN5A
chr3	38655208	38655350	SCN5A
chr3	38655440	38655582	SCN5A
chr3	38662308	38662487	SCN5A
chr3	38663865	38664005	SCN5A
chr3	38671776	38671945	SCN5A
chr3	38674500	38674823	SCN5A
chr3	41265534	41265597	CTNNB1
chr3	41265991	41266269	CTNNB1
chr3	41266419	41266723	CTNNB1
chr3	41266799	41267088	CTNNB1
chr3	41267125	41267377	CTNNB1
chr3	41268673	41268868	CTNNB1
chr3	41274806	41274960	CTNNB1
chr3	41274994	41275383	CTNNB1
chr3	41275604	41275813	CTNNB1
chr3	41277189	41277359	CTNNB1
chr3	41277814	41278015	CTNNB1
chr3	41278053	41278225	CTNNB1
chr3	41279481	41279592	CTNNB1
chr3	41280599	41280858	CTNNB1
chr3	47058557	47058769	SETD2
chr3	47059102	47059254	SETD2
chr3	47061224	47061355	SETD2
chr3	47079130	47079292	SETD2
chr3	47084025	47084215	SETD2
chr3	47087951	47088136	SETD2
chr3	47098285	47099005	SETD2
chr3	47103627	47103861	SETD2
chr3	47108534	47108633	SETD2
chr3	47125184	47125897	SETD2
chr3	47127659	47127829	SETD2
chr3	47129577	47129762	SETD2
chr3	47139419	47139596	SETD2
chr3	47142922	47143070	SETD2
chr3	47144810	47144938	SETD2
chr3	47147461	47147635	SETD2
chr3	47155340	47155519	SETD2
chr3	47158087	47158269	SETD2
chr3	47161646	47166063	SETD2
chr3	47168112	47168178	SETD2
chr3	47169023	47169078	SETD2
chr3	47179739	47179800	SETD2
chr3	47186973	47187032	SETD2
chr3	47199022	47199079	SETD2
chr3	47205318	47205439	SETD2
chr3	52436278	52436462	BAP1
chr3	52436592	52436715	BAP1
chr3	52436769	52436912	BAP1
chr3	52437128	52437339	BAP1
chr3	52437406	52437935	BAP1
chr3	52438443	52438627	BAP1
chr3	52439100	52439335	BAP1
chr3	52439755	52439953	BAP1
chr3	52440243	52440417	BAP1
chr3	52440819	52440948	BAP1
chr3	52441164	52441357	BAP1
chr3	52441389	52441501	BAP1
chr3	52441948	52442118	BAP1
chr3	52442464	52442647	BAP1
chr3	52443544	52443649	BAP1
chr3	52443704	52443784	BAP1
chr3	52443832	52443919	BAP1
chr3	52582053	52582276	PBRM1, SMIM4
chr3	52584411	52584678	PBRM1, SMIM4
chr3	52584737	52584858	PBRM1, SMIM4
chr3	52588714	52588920	PBRM1, SMIM4
chr3	52592239	52592454	PBRM1, SMIM4
chr3	52595757	52596009	PBRM1, SMIM4
chr3	52597273	52597534	PBRM1, SMIM4
chr3	52598040	52598274	PBRM1, SMIM4
chr3	52610531	52610739	PBRM1, SMIM4
chr3	52613044	52613240	PBRM1, SMIM4
chr3	52620415	52620729	PBRM1
chr3	52621343	52621551	PBRM1
chr3	52623060	52623296	PBRM1
chr3	52637511	52637773	PBRM1
chr3	52643303	52643996	PBRM1
chr3	52649341	52649497	PBRM1
chr3	52651252	52651579	PBRM1
chr3	52658887	52658982	PBRM1
chr3	52661263	52661411	PBRM1
chr3	52662884	52663076	PBRM1
chr3	52668592	52668856	PBRM1
chr3	52675944	52676086	PBRM1

chr3	52677238	52677384	PBRM1
chr3	52678694	52678830	PBRM1
chr3	52682334	52682483	PBRM1
chr3	52685732	52685851	PBRM1
chr3	52692189	52692356	PBRM1
chr3	52696123	52696317	PBRM1
chr3	52702488	52702686	PBRM1
chr3	52712490	52712638	PBRM1
chr3	52713564	52713752	PBRM1
chr3	69788723	69788877	MITF
chr3	69812967	69813118	MITF
chr3	69915416	69915522	MITF
chr3	69928230	69928559	MITF
chr3	69985848	69985931	MITF
chr3	69986947	69987225	MITF
chr3	69987477	69987542	MITF
chr3	69988223	69988357	MITF
chr3	69990361	69990507	MITF
chr3	69998176	69998344	MITF
chr3	70000937	70001062	MITF
chr3	70005580	70005706	MITF
chr3	70008398	70008596	MITF
chr3	70013972	70014424	MITF
chr3	71007392	71007497	FOXP1
chr3	71008372	71008567	FOXP1
chr3	71015015	71015232	FOXP1
chr3	71019861	71019981	FOXP1
chr3	71021136	71021356	FOXP1
chr3	71021680	71021852	FOXP1
chr3	71026066	71026218	FOXP1
chr3	71026768	71026898	FOXP1
chr3	71026953	71027205	FOXP1
chr3	71037119	71037253	FOXP1
chr3	71050097	71050235	FOXP1
chr3	71064674	71064829	FOXP1
chr3	71090453	71090708	FOXP1
chr3	71096067	71096271	FOXP1
chr3	71101662	71101802	FOXP1
chr3	71102761	71102949	FOXP1
chr3	71161661	71161813	FOXP1
chr3	71179623	71179859	FOXP1
chr3	71247029	71247244	FOXP1
chr3	71247327	71247557	FOXP1
chr3	89156873	89157011	EPHA3
chr3	89176333	89176448	EPHA3
chr3	89258984	89259695	EPHA3
chr3	89390040	89390246	EPHA3
chr3	89390879	89391265	EPHA3
chr3	89444961	89445136	EPHA3
chr3	89448442	89448681	EPHA3
chr3	89456393	89456546	EPHA3
chr3	89457191	89457306	EPHA3
chr3	89462265	89462441	EPHA3
chr3	89468329	89468565	EPHA3
chr3	89478230	89478342	EPHA3
chr3	89480274	89480534	EPHA3
chr3	89498349	89498549	EPHA3
chr3	89499301	89499545	EPHA3
chr3	89521588	89521794	EPHA3
chr3	89528521	89528677	EPHA3
chr3	96533442	96533877	EPHA6
chr3	96585643	96585758	EPHA6
chr3	96706148	96706862	EPHA6
chr3	96728822	96728898	EPHA6
chr3	96945082	96945288	EPHA6
chr3	96962770	96963194	EPHA6
chr3	97123968	97124143	EPHA6
chr3	97167386	97167599	EPHA6
chr3	97185237	97185351	EPHA6
chr3	97194170	97194329	EPHA6
chr3	97198112	97198233	EPHA6
chr3	97202752	97202928	EPHA6
chr3	97251176	97251412	EPHA6
chr3	97311430	97311606	EPHA6
chr3	97329611	97329723	EPHA6
chr3	97331196	97331300	EPHA6
chr3	97356691	97356951	EPHA6
chr3	97364937	97365179	EPHA6
chr3	97367104	97367199	EPHA6
chr3	97439079	97439279	EPHA6
chr3	97454743	97454987	EPHA6
chr3	97466241	97466441	EPHA6
chr3	97467405	97467570	EPHA6
chr3	119501579	119501724	NR1I2
chr3	119526050	119526319	NR1I2
chr3	119528882	119529066	NR1I2
chr3	119530360	119530598	NR1I2
chr3	119531507	119531832	NR1I2
chr3	119533800	119533993	NR1I2
chr3	119534144	119534311	NR1I2
chr3	119534547	119534703	NR1I2
chr3	119535889	119536084	NR1I2
chr3	128199836	128200186	GATA2
chr3	128200636	128200812	GATA2
chr3	128202677	128202873	GATA2
chr3	128204544	128205236	GATA2

chr3	128205620	128205899	GATA2
chr3	134514448	134514556	EPHB1
chr3	134644632	134644747	EPHB1
chr3	134670187	134670919	EPHB1
chr3	134825264	134825470	EPHB1
chr3	134851530	134851916	EPHB1
chr3	134872968	134873143	EPHB1
chr3	134880834	134881047	EPHB1
chr3	134884784	134884943	EPHB1
chr3	134885758	134885873	EPHB1
chr3	134898676	134898849	EPHB1
chr3	134911392	134911690	EPHB1
chr3	134920290	134920556	EPHB1
chr3	134959964	134960164	EPHB1
chr3	134967132	134967376	EPHB1
chr3	134968152	134968358	EPHB1
chr3	134977828	134977987	EPHB1
chr3	138664408	138665589	FOXL2
chr3	142168245	142168469	ATR
chr3	142171944	142172100	ATR
chr3	142176420	142176622	ATR
chr3	142177774	142177978	ATR
chr3	142178043	142178250	ATR
chr3	142180756	142180957	ATR
chr3	142183913	142184107	ATR
chr3	142185140	142185400	ATR
chr3	142186750	142186935	ATR
chr3	142188153	142188436	ATR
chr3	142188902	142189050	ATR
chr3	142203956	142204149	ATR
chr3	142211948	142212178	ATR
chr3	142215177	142215387	ATR
chr3	142215829	142216059	ATR
chr3	142217413	142217641	ATR
chr3	142218443	142218585	ATR
chr3	142222178	142222320	ATR
chr3	142223955	142224170	ATR
chr3	142226747	142226976	ATR
chr3	142231076	142231337	ATR
chr3	142232317	142232505	ATR
chr3	142234211	142234382	ATR
chr3	142238485	142238651	ATR
chr3	142241544	142241708	ATR
chr3	142242809	142243066	ATR
chr3	142253896	142254072	ATR
chr3	142254924	142255068	ATR
chr3	142257298	142257492	ATR
chr3	142259720	142259901	ATR
chr3	142261481	142261624	ATR
chr3	142266541	142266777	ATR
chr3	142268295	142268540	ATR
chr3	142268948	142269169	ATR
chr3	142272043	142272265	ATR
chr3	142272456	142272607	ATR
chr3	142272641	142272882	ATR
chr3	142274693	142275006	ATR
chr3	142275199	142275442	ATR
chr3	142277440	142277643	ATR
chr3	142278067	142278308	ATR
chr3	142279079	142279321	ATR
chr3	142280059	142280288	ATR
chr3	142281048	142281976	ATR
chr3	142284937	142285128	ATR
chr3	142286879	142287021	ATR
chr3	142297462	142297571	ATR
chr3	151055579	151056658	MED12L, P2RY12
chr3	152553546	152554718	P2RY1
chr3	178916588	178916990	PIK3CA
chr3	178917452	178917712	PIK3CA
chr3	178919052	178919353	PIK3CA
chr3	178921306	178921602	PIK3CA
chr3	178922265	178922401	PIK3CA
chr3	178927357	178927513	PIK3CA
chr3	178927948	178928151	PIK3CA
chr3	178928193	178928378	PIK3CA
chr3	178935972	178936147	PIK3CA
chr3	178936958	178937090	PIK3CA
chr3	178937333	178937548	PIK3CA
chr3	178937711	178937865	PIK3CA
chr3	178938748	178938970	PIK3CA
chr3	178941843	178942000	PIK3CA
chr3	178942462	178942634	PIK3CA
chr3	178943724	178943853	PIK3CA
chr3	178947034	178947255	PIK3CA
chr3	178947766	178947934	PIK3CA
chr3	178947987	178948189	PIK3CA
chr3	178951856	178952177	PIK3CA
chr3	181430123	181431127	SOX2
chr3	187440220	187440414	BCL6
chr3	187442703	187442891	BCL6
chr3	187443261	187443442	BCL6
chr3	187444493	187444711	BCL6
chr3	187446122	187446357	BCL6
chr3	187446812	187447834	BCL6
chr3	187449471	187449743	BCL6
chr3	187451295	187451506	BCL6

chr4	1795636	1795795	FGFR3
chr4	1800955	1801275	FGFR3
chr4	1801448	1801564	FGFR3
chr4	1803068	1803288	FGFR3
chr4	1803321	1803495	FGFR3
chr4	1803536	1803777	FGFR3
chr4	1804615	1804816	FGFR3
chr4	1805393	1805588	FGFR3
chr4	1806031	1806272	FGFR3
chr4	1806522	1806721	FGFR3
chr4	1807056	1807228	FGFR3
chr4	1807260	1807421	FGFR3
chr4	1807451	1807692	FGFR3
chr4	1807752	1807925	FGFR3
chr4	1807958	1808079	FGFR3
chr4	1808247	1808435	FGFR3
chr4	1808530	1808686	FGFR3
chr4	1808817	1809439	FGFR3
chr4	15780012	15780295	CD38
chr4	15818108	15818288	CD38
chr4	15818318	15818374	CD38
chr4	15826478	15826664	CD38
chr4	15835814	15835950	CD38
chr4	15839689	15839813	CD38
chr4	15841623	15841766	CD38
chr4	15842049	15842186	CD38
chr4	15850136	15850250	CD38
chr4	55124910	55125009	FIP1L1, PDGFRA
chr4	55127236	55127604	FIP1L1, PDGFRA
chr4	55129808	55130148	FIP1L1, PDGFRA
chr4	55131060	55131241	FIP1L1, PDGFRA
chr4	55133430	55133652	FIP1L1, PDGFRA
chr4	55133693	55133933	FIP1L1, PDGFRA
chr4	55136774	55136940	FIP1L1, PDGFRA
chr4	55138535	55138712	FIP1L1, PDGFRA
chr4	55139678	55139922	FIP1L1, PDGFRA
chr4	55140672	55140817	FIP1L1, PDGFRA
chr4	55140982	55141165	FIP1L1, PDGFRA
chr4	55143529	55143684	FIP1L1, PDGFRA
chr4	55144037	55144198	FIP1L1, PDGFRA
chr4	55144503	55144707	FIP1L1, PDGFRA
chr4	55146457	55146674	FIP1L1, PDGFRA
chr4	55151512	55151678	FIP1L1, PDGFRA
chr4	55151982	55152155	FIP1L1, PDGFRA
chr4	55153571	55153733	FIP1L1, PDGFRA
chr4	55154940	55155090	FIP1L1, PDGFRA
chr4	55155150	55155306	FIP1L1, PDGFRA
chr4	55156454	55156746	FIP1L1, PDGFRA
chr4	55161266	55161464	FIP1L1, PDGFRA
chr4	55524156	55524273	KIT
chr4	55561652	55561972	KIT
chr4	55564424	55564756	KIT
chr4	55565770	55565957	KIT
chr4	55569864	55570083	KIT
chr4	55573238	55573478	KIT
chr4	55575564	55575778	KIT
chr4	55589724	55589889	KIT
chr4	55591997	55592241	KIT
chr4	55593358	55593515	KIT
chr4	55593556	55593733	KIT
chr4	55593963	55594118	KIT
chr4	55594151	55594312	KIT
chr4	55595475	55595676	KIT
chr4	55597468	55597610	KIT
chr4	55598011	55598189	KIT
chr4	55599210	55599383	KIT
chr4	55602638	55602800	KIT
chr4	55602861	55603011	KIT
chr4	55603315	55603471	KIT
chr4	55604569	55604748	KIT
chr4	55946082	55946355	KDR
chr4	55948097	55948233	KDR
chr4	55948677	55948827	KDR
chr4	55953748	55953950	KDR
chr4	55955009	55955165	KDR
chr4	55955515	55955665	KDR
chr4	55955832	55955994	KDR
chr4	55956097	55956270	KDR
chr4	55958758	55958906	KDR
chr4	55960943	55961147	KDR
chr4	55961718	55961857	KDR
chr4	55962370	55962534	KDR
chr4	55963803	55963958	KDR
chr4	55964278	55964464	KDR
chr4	55964838	55964995	KDR
chr4	55968038	55968220	KDR
chr4	55968503	55968700	KDR
chr4	55970784	55971176	KDR
chr4	55971973	55972132	KDR
chr4	55972828	55973002	KDR
chr4	55973878	55974085	KDR
chr4	55976544	55976758	KDR
chr4	55976795	55976960	KDR
chr4	55979445	55979673	KDR
chr4	55980267	55980457	KDR
chr4	55981015	55981234	KDR

chr4	55981422	55981603	KDR
chr4	55984745	55984992	KDR
chr4	55987238	55987382	KDR
chr4	55991368	55991485	KDR
chr4	66189806	66189962	EPHA5
chr4	66197661	66197871	EPHA5
chr4	66201624	66201868	EPHA5
chr4	66213746	66213946	EPHA5
chr4	66217081	66217341	EPHA5
chr4	66218734	66218846	EPHA5
chr4	66230709	66230945	EPHA5
chr4	66231624	66231800	EPHA5
chr4	66233049	66233167	EPHA5
chr4	66242690	66242806	EPHA5
chr4	66270066	66270222	EPHA5
chr4	66279976	66280186	EPHA5
chr4	66286133	66286308	EPHA5
chr4	66356069	66356455	EPHA5
chr4	66361080	66361286	EPHA5
chr4	66467333	66468047	EPHA5
chr4	66509055	66509170	EPHA5
chr4	66535254	66535485	EPHA5
chr4	100197755	100197830	ADH1A
chr4	100200557	100200746	ADH1A
chr4	100201275	100201461	ADH1A
chr4	100203477	100203788	ADH1A
chr4	100205530	100205800	ADH1A
chr4	100205847	100205985	ADH1A
chr4	100207981	100208170	ADH1A
chr4	100208695	100208847	ADH1A
chr4	100212028	100212096	ADH1A
chr4	100228971	100229046	ADH1B
chr4	100231896	100232085	ADH1B
chr4	100232652	100232838	ADH1B
chr4	100234952	100235263	ADH1B
chr4	100237029	100237299	ADH1B
chr4	100237346	100237484	ADH1B
chr4	100239177	100239366	ADH1B
chr4	100239916	100240068	ADH1B
chr4	100242445	100242513	ADH1B
chr4	100257883	100257958	ADH1C
chr4	100260708	100260897	ADH1C
chr4	100261706	100261892	ADH1C
chr4	100263926	100264237	ADH1C
chr4	100265993	100266263	ADH1C
chr4	100266310	100266448	ADH1C
chr4	100268137	100268326	ADH1C
chr4	100268876	100269028	ADH1C
chr4	100273789	100273857	ADH1C
chr4	106111601	106111668	TET2
chr4	106155028	106158622	TET2
chr4	106162470	106162615	TET2
chr4	106163965	106164109	TET2
chr4	106164701	106164960	TET2
chr4	106180750	106180951	TET2
chr4	106182890	106183030	TET2
chr4	106190741	106190929	TET2
chr4	106193695	106194100	TET2
chr4	106196179	106197701	TET2
chr4	153244007	153244326	FBXW7
chr4	153245310	153245571	FBXW7
chr4	153247132	153247408	FBXW7
chr4	153249334	153249566	FBXW7
chr4	153250798	153250962	FBXW7
chr4	153251858	153252045	FBXW7
chr4	153253722	153253896	FBXW7
chr4	153258928	153259113	FBXW7
chr4	153268056	153268248	FBXW7
chr4	153269800	153269906	FBXW7
chr4	153271168	153271301	FBXW7
chr4	153273596	153273907	FBXW7
chr4	153303315	153303512	FBXW7
chr4	153332399	153332980	FBXW7
chr5	1253817	1253971	TERT
chr5	1254457	1254645	TERT
chr5	1255376	1255551	TERT
chr5	1258687	1258799	TERT
chr5	1260563	1260740	TERT
chr5	1264493	1264732	TERT
chr5	1266553	1266675	TERT
chr5	1268609	1268773	TERT
chr5	1271208	1271344	TERT
chr5	1272274	1272420	TERT
chr5	1278730	1278936	TERT
chr5	1279380	1279610	TERT
chr5	1280247	1280478	TERT
chr5	1282518	1282764	TERT
chr5	1293402	1294806	TERT
chr5	1294860	1295129	TERT
chr5	35857054	35857186	IL7R
chr5	35860928	35861117	IL7R
chr5	35867382	35867664	IL7R
chr5	35871132	35871340	IL7R
chr5	35873556	35873775	IL7R
chr5	35874525	35874669	IL7R
chr5	35875588	35875718	IL7R

chr5	35876059	35876613	IL7R
chr5	38942380	38942505	RICTOR
chr5	38942909	38943098	RICTOR
chr5	38944522	38944696	RICTOR
chr5	38944989	38945195	RICTOR
chr5	38945567	38945851	RICTOR
chr5	38946544	38946679	RICTOR
chr5	38947340	38947568	RICTOR
chr5	38949432	38949554	RICTOR
chr5	38949788	38950847	RICTOR
chr5	38952272	38952552	RICTOR
chr5	38953061	38953218	RICTOR
chr5	38953537	38953680	RICTOR
chr5	38954850	38954988	RICTOR
chr5	38955671	38955831	RICTOR
chr5	38957728	38957857	RICTOR
chr5	38958519	38958646	RICTOR
chr5	38958743	38958958	RICTOR
chr5	38959271	38959448	RICTOR
chr5	38959855	38960105	RICTOR
chr5	38960474	38960660	RICTOR
chr5	38962391	38962488	RICTOR
chr5	38962561	38962713	RICTOR
chr5	38962952	38963168	RICTOR
chr5	38964868	38965019	RICTOR
chr5	38966717	38966848	RICTOR
chr5	38967237	38967354	RICTOR
chr5	38967413	38967554	RICTOR
chr5	38968019	38968157	RICTOR
chr5	38971953	38972086	RICTOR
chr5	38975613	38975731	RICTOR
chr5	38978659	38978777	RICTOR
chr5	38981943	38982163	RICTOR
chr5	38991025	38991202	RICTOR
chr5	38996895	38997009	RICTOR
chr5	39002611	39002793	RICTOR
chr5	39003634	39003749	RICTOR
chr5	39021115	39021263	RICTOR
chr5	39074187	39074285	RICTOR
chr5	39074405	39074504	RICTOR
chr5	56111375	56111907	MAP3K1
chr5	56152401	56152602	MAP3K1
chr5	56155516	56155767	MAP3K1
chr5	56160535	56160786	MAP3K1
chr5	56161141	56161308	MAP3K1
chr5	56161630	56161829	MAP3K1
chr5	56167711	56167883	MAP3K1
chr5	56168442	56168574	MAP3K1
chr5	56168626	56168857	MAP3K1
chr5	56170833	56171162	MAP3K1
chr5	56174781	56174953	MAP3K1
chr5	56176512	56176654	MAP3K1
chr5	56176884	56177124	MAP3K1
chr5	56177371	56178718	MAP3K1
chr5	56179328	56179531	MAP3K1
chr5	56180465	56180678	MAP3K1
chr5	56181733	56181915	MAP3K1
chr5	56183179	56183372	MAP3K1
chr5	56184027	56184209	MAP3K1
chr5	56189332	56189532	MAP3K1
chr5	67522478	67522862	PIK3R1
chr5	67569192	67569335	PIK3R1
chr5	67569741	67569866	PIK3R1
chr5	67575404	67575586	PIK3R1
chr5	67576330	67576582	PIK3R1
chr5	67576729	67576859	PIK3R1
chr5	67584538	67584604	PIK3R1
chr5	67586531	67586687	PIK3R1
chr5	67588061	67588214	PIK3R1
chr5	67588903	67589052	PIK3R1
chr5	67589105	67589336	PIK3R1
chr5	67589511	67589687	PIK3R1
chr5	67590338	67590531	PIK3R1
chr5	67590950	67591177	PIK3R1
chr5	67591222	67591341	PIK3R1
chr5	67591949	67592194	PIK3R1
chr5	67593214	67593454	PIK3R1
chr5	74638405	74638620	HMGCR
chr5	74638663	74638715	HMGCR
chr5	74639652	74639814	HMGCR
chr5	74640044	74640182	HMGCR
chr5	74641373	74641508	HMGCR
chr5	74643003	74643159	HMGCR
chr5	74645841	74645998	HMGCR
chr5	74646057	74646224	HMGCR
chr5	74646588	74646799	HMGCR
chr5	74646867	74647165	HMGCR
chr5	74647223	74647452	HMGCR
chr5	74650302	74650547	HMGCR
chr5	74650855	74651064	HMGCR
chr5	74651164	74651372	HMGCR
chr5	74652142	74652298	HMGCR
chr5	74654456	74654677	HMGCR
chr5	74654969	74655160	HMGCR
chr5	74655197	74655406	HMGCR
chr5	74655784	74655989	HMGCR

chr5	74656087	74656192	HMGCR
chr5	112043389	112043604	APC
chr5	112090562	112090747	APC
chr5	112101997	112102132	APC
chr5	112102860	112103112	APC
chr5	112111300	112111459	APC
chr5	112116461	112116625	APC
chr5	112128117	112128251	APC
chr5	112136950	112137105	APC
chr5	112151166	112151315	APC
chr5	112154637	112155066	APC
chr5	112157567	112157713	APC
chr5	112162779	112162969	APC
chr5	112163600	112163728	APC
chr5	112164527	112164694	APC
chr5	112170622	112170887	APC
chr5	112173224	112179848	APC
chr5	138117588	138117743	CTNNA1
chr5	138118840	138119086	CTNNA1
chr5	138145701	138145918	CTNNA1
chr5	138147846	138148016	CTNNA1
chr5	138160193	138160513	CTNNA1
chr5	138163178	138163432	CTNNA1
chr5	138221875	138222006	CTNNA1
chr5	138223153	138223356	CTNNA1
chr5	138240012	138240155	CTNNA1
chr5	138253405	138253612	CTNNA1
chr5	138260173	138260424	CTNNA1
chr5	138260919	138261121	CTNNA1
chr5	138264909	138265070	CTNNA1
chr5	138266136	138266368	CTNNA1
chr5	138266493	138266649	CTNNA1
chr5	138268241	138268426	CTNNA1
chr5	138268551	138268672	CTNNA1
chr5	138269465	138269803	CTNNA1
chr5	148206369	148207661	ADRB2
chr5	149433606	149433818	CSF1R
chr5	149433859	149434018	CSF1R
chr5	149434774	149434924	CSF1R
chr5	149435563	149435725	CSF1R
chr5	149435756	149435929	CSF1R
chr5	149436824	149436972	CSF1R
chr5	149437041	149437180	CSF1R
chr5	149439237	149439450	CSF1R
chr5	149440399	149440560	CSF1R
chr5	149441028	149441183	CSF1R
chr5	149441260	149441437	CSF1R
chr5	149447752	149447918	CSF1R
chr5	149449410	149449651	CSF1R
chr5	149449719	149449890	CSF1R
chr5	149449993	149450159	CSF1R
chr5	149452838	149453081	CSF1R
chr5	149456813	149457023	CSF1R
chr5	149457649	149457836	CSF1R
chr5	149459589	149459924	CSF1R
chr5	149460304	149460612	CSF1R
chr5	149465916	149466015	CSF1R
chr5	149495300	149495534	PDGFRB
chr5	149497155	149497438	PDGFRB
chr5	149498284	149498440	PDGFRB
chr5	149499004	149499154	PDGFRB
chr5	149499549	149499711	PDGFRB
chr5	149500425	149500598	PDGFRB
chr5	149500741	149500910	PDGFRB
chr5	149501417	149501628	PDGFRB
chr5	149502579	149502789	PDGFRB
chr5	149503787	149503948	PDGFRB
chr5	149504264	149504419	PDGFRB
chr5	149504982	149505165	PDGFRB
chr5	149506057	149506202	PDGFRB
chr5	149509294	149509556	PDGFRB
chr5	149510076	149510250	PDGFRB
chr5	149511516	149511682	PDGFRB
chr5	149512287	149512530	PDGFRB
chr5	149513123	149513348	PDGFRB
chr5	149513418	149513596	PDGFRB
chr5	149514287	149514604	PDGFRB
chr5	149515092	149515466	PDGFRB
chr5	149516545	149516635	PDGFRB
chr5	161112970	161113058	GABRA6
chr5	161113210	161113379	GABRA6
chr5	161113916	161114034	GABRA6
chr5	161115929	161116200	GABRA6
chr5	161116234	161116367	GABRA6
chr5	161116616	161116810	GABRA6
chr5	161117181	161117384	GABRA6
chr5	161118921	161119231	GABRA6
chr5	161128478	161128804	GABRA6
chr5	170814927	170815035	NPM1
chr5	170817029	170817159	NPM1
chr5	170818283	170818453	NPM1
chr5	170818684	170818828	NPM1
chr5	170819688	170819845	NPM1
chr5	170819892	170820007	NPM1
chr5	170827131	170827239	NPM1
chr5	170827817	170827954	NPM1

chr5	170832280	170832432	NPM1
chr5	170833375	170833434	NPM1
chr5	170834678	170834803	NPM1
chr5	170837505	170837594	NPM1
chr5	176516578	176516719	FGFR4
chr5	176517044	176517679	FGFR4
chr5	176517720	176518130	FGFR4
chr5	176518660	176518834	FGFR4
chr5	176519296	176519537	FGFR4
chr5	176519621	176520577	FGFR4
chr5	176520629	176520801	FGFR4
chr5	176522305	176522466	FGFR4
chr5	176522508	176522749	FGFR4
chr5	176523032	176523205	FGFR4
chr5	176523262	176523383	FGFR4
chr5	176523579	176523767	FGFR4
chr5	176524267	176524423	FGFR4
chr5	176524502	176524702	FGFR4
chr5	176562079	176563056	NSD1
chr5	176563848	176563982	NSD1
chr5	176618859	176619045	NSD1
chr5	176631095	176631318	NSD1
chr5	176636611	176639221	NSD1
chr5	176647993	176648051	NSD1
chr5	176662796	176662971	NSD1
chr5	176665212	176665533	NSD1
chr5	176666731	176666891	NSD1
chr5	176671170	176671296	NSD1
chr5	176673653	176673822	NSD1
chr5	176675156	176675350	NSD1
chr5	176678705	176678879	NSD1
chr5	176683926	176684177	NSD1
chr5	176686964	176687194	NSD1
chr5	176694537	176694744	NSD1
chr5	176696577	176696833	NSD1
chr5	176700647	176700810	NSD1
chr5	176707540	176707860	NSD1
chr5	176709440	176709607	NSD1
chr5	176710762	176710954	NSD1
chr5	176715794	176715951	NSD1
chr5	176718929	176719184	NSD1
chr5	176720807	176722485	NSD1
chr5	180030166	180030415	FLT4
chr5	180035106	180035184	FLT4
chr5	180035255	180035309	FLT4
chr5	180035942	180036078	FLT4
chr5	180036879	180037050	FLT4
chr5	180038305	180038504	FLT4
chr5	180039480	180039636	FLT4
chr5	180039985	180040135	FLT4
chr5	180041042	180041204	FLT4
chr5	180043341	180043514	FLT4
chr5	180043874	180044019	FLT4
chr5	180045744	180045945	FLT4
chr5	180045995	180046134	FLT4
chr5	180046227	180046391	FLT4
chr5	180046428	180046794	FLT4
chr5	180047147	180047333	FLT4
chr5	180047583	180047740	FLT4
chr5	180047850	180048032	FLT4
chr5	180048080	180048277	FLT4
chr5	180048516	180048929	FLT4
chr5	180049705	180049864	FLT4
chr5	180050909	180051086	FLT4
chr5	180052843	180053056	FLT4
chr5	180053085	180053290	FLT4
chr5	180055856	180056024	FLT4
chr5	180056233	180056452	FLT4
chr5	180056670	180056860	FLT4
chr5	180056917	180057130	FLT4
chr5	180057199	180057362	FLT4
chr5	180057529	180057824	FLT4
chr5	180058656	180058803	FLT4
chr5	180076462	180076570	FLT4
chr6	18130873	18131036	TPMT
chr6	18132338	18132433	TPMT
chr6	18134009	18134145	TPMT
chr6	18139168	18139293	TPMT
chr6	18139870	18139973	TPMT
chr6	18143801	18143984	TPMT
chr6	18148028	18148171	TPMT
chr6	18149193	18149383	TPMT
chr6	32163188	32163952	GPSM3, NOTCH4
chr6	32164075	32164223	NOTCH4
chr6	32164676	32164874	NOTCH4
chr6	32165050	32165396	NOTCH4
chr6	32166172	32166361	NOTCH4
chr6	32166400	32166532	NOTCH4
chr6	32166677	32166947	NOTCH4
chr6	32168582	32168808	NOTCH4
chr6	32168868	32169302	NOTCH4
chr6	32169827	32170401	NOTCH4
chr6	32171521	32171684	NOTCH4
chr6	32171888	32172191	NOTCH4
chr6	32178503	32178738	NOTCH4
chr6	32180225	32180429	NOTCH4

chr6	32180575	32180713	NOTCH4
chr6	32180886	32181054	NOTCH4
chr6	32181439	32181642	NOTCH4
chr6	32181861	32182057	NOTCH4
chr6	32182977	32183187	NOTCH4
chr6	32184696	32184869	NOTCH4
chr6	32184904	32185068	NOTCH4
chr6	32185746	32185910	NOTCH4
chr6	32187343	32187588	NOTCH4
chr6	32187880	32188086	NOTCH4
chr6	32188156	32188443	NOTCH4
chr6	32188507	32188680	NOTCH4
chr6	32188729	32189127	NOTCH4
chr6	32190262	32190608	NOTCH4
chr6	32190756	32190888	NOTCH4
chr6	32191607	32191730	NOTCH4
chr6	33259798	33260115	PFDN6, RGL2
chr6	33260181	33260346	PFDN6, RGL2
chr6	33260767	33261108	PFDN6, RGL2
chr6	33261164	33261326	PFDN6, RGL2
chr6	33261355	33261501	PFDN6, RGL2
chr6	33261542	33261868	PFDN6, RGL2
chr6	33262439	33262564	PFDN6, RGL2
chr6	33262728	33262847	PFDN6, RGL2
chr6	33262878	33263013	PFDN6, RGL2
chr6	33263070	33263224	PFDN6, RGL2
chr6	33263259	33263561	PFDN6, RGL2
chr6	33263779	33264127	PFDN6, RGL2
chr6	33264165	33264266	PFDN6, RGL2
chr6	33264349	33264578	PFDN6, RGL2
chr6	33264783	33264917	PFDN6, RGL2
chr6	33266206	33266412	RGL2
chr6	33286494	33286604	DAXX
chr6	33286748	33287021	DAXX
chr6	33287131	33287656	DAXX
chr6	33287762	33288026	DAXX
chr6	33288131	33288393	DAXX
chr6	33288487	33289369	DAXX
chr6	33289470	33289727	DAXX
chr6	33290613	33290716	DAXX
chr6	35420297	35420595	FANCE
chr6	35423498	35424155	FANCE
chr6	35425307	35425402	FANCE
chr6	35425667	35425786	FANCE
chr6	35426048	35426242	FANCE
chr6	35427082	35427256	FANCE
chr6	35427433	35427562	FANCE
chr6	35428303	35428420	FANCE
chr6	35430540	35430716	FANCE
chr6	35433995	35434147	FANCE
chr6	36645588	36645735	CDKN1A
chr6	36651848	36652348	CDKN1A
chr6	36653502	36653602	CDKN1A
chr6	41533473	41533727	FOXP4
chr6	41545698	41545844	FOXP4
chr6	41552481	41552654	FOXP4
chr6	41553143	41553280	FOXP4
chr6	41554721	41554919	FOXP4
chr6	41555011	41555275	FOXP4
chr6	41555505	41555660	FOXP4
chr6	41556356	41556494	FOXP4
chr6	41557483	41557617	FOXP4
chr6	41557675	41557933	FOXP4
chr6	41557983	41558110	FOXP4
chr6	41558933	41559085	FOXP4
chr6	41562582	41562754	FOXP4
chr6	41564888	41565005	FOXP4
chr6	41565493	41565713	FOXP4
chr6	41566501	41566699	FOXP4, MIR4641
chr6	41903563	41903870	CCND3
chr6	41904271	41904458	CCND3
chr6	41904947	41905157	CCND3
chr6	41908082	41908348	CCND3
chr6	41908664	41908762	CCND3
chr6	41909164	41909412	CCND3
chr6	43738418	43739074	VEGFA
chr6	43742052	43742154	VEGFA
chr6	43745180	43745427	VEGFA
chr6	43746171	43746298	VEGFA
chr6	43746600	43746680	VEGFA
chr6	43748443	43748616	VEGFA
chr6	43749667	43749849	VEGFA
chr6	43752252	43752390	VEGFA
chr6	51483853	51484343	PKHD1
chr6	51491769	51491939	PKHD1
chr6	51497337	51497546	PKHD1
chr6	51503621	51503779	PKHD1
chr6	51512803	51512941	PKHD1
chr6	51513857	51514043	PKHD1
chr6	51523724	51524792	PKHD1
chr6	51586758	51586843	PKHD1
chr6	51604766	51604842	PKHD1
chr6	51605924	51605980	PKHD1
chr6	51609157	51609365	PKHD1
chr6	51611493	51611712	PKHD1
chr6	51612559	51613488	PKHD1

chr6	51617973	51618176	PKHD1
chr6	51619556	51619761	PKHD1
chr6	51637474	51637612	PKHD1
chr6	51640580	51640744	PKHD1
chr6	51656008	51656196	PKHD1
chr6	51695633	51695812	PKHD1
chr6	51701176	51701292	PKHD1
chr6	51712547	51712793	PKHD1
chr6	51720665	51720893	PKHD1
chr6	51732635	51732932	PKHD1
chr6	51735276	51735462	PKHD1
chr6	51747865	51748050	PKHD1
chr6	51750639	51750795	PKHD1
chr6	51751905	51752068	PKHD1
chr6	51768369	51768550	PKHD1
chr6	51768758	51768865	PKHD1
chr6	51770987	51771163	PKHD1
chr6	51774055	51774297	PKHD1
chr6	51776571	51776779	PKHD1
chr6	51777138	51777399	PKHD1
chr6	51798882	51799145	PKHD1
chr6	51824642	51824849	PKHD1
chr6	51875081	51875282	PKHD1
chr6	51882182	51882452	PKHD1
chr6	51887573	51887767	PKHD1
chr6	51889346	51891004	PKHD1
chr6	51892601	51892719	PKHD1
chr6	51892928	51893174	PKHD1
chr6	51897802	51897988	PKHD1
chr6	51900363	51900544	PKHD1
chr6	51907631	51907957	PKHD1
chr6	51908397	51908553	PKHD1
chr6	51909738	51909911	PKHD1
chr6	51910776	51911011	PKHD1
chr6	51913264	51913442	PKHD1
chr6	51914929	51915118	PKHD1
chr6	51917848	51918074	PKHD1
chr6	51918810	51918988	PKHD1
chr6	51920359	51920552	PKHD1
chr6	51921470	51921611	PKHD1
chr6	51921662	51921802	PKHD1
chr6	51923095	51923424	PKHD1
chr6	51924700	51924865	PKHD1
chr6	51927291	51927483	PKHD1
chr6	51929727	51929873	PKHD1
chr6	51930748	51930900	PKHD1
chr6	51934229	51934350	PKHD1
chr6	51935178	51935268	PKHD1
chr6	51935778	51935893	PKHD1
chr6	51936887	51937012	PKHD1
chr6	51938235	51938364	PKHD1
chr6	51941048	51941156	PKHD1
chr6	51944672	51944831	PKHD1
chr6	51947164	51947365	PKHD1
chr6	51947950	51948078	PKHD1
chr6	51949654	51949756	PKHD1
chr6	93953118	93953283	EPHA7
chr6	93954990	93955196	EPHA7
chr6	93956484	93956728	EPHA7
chr6	93964339	93964539	EPHA7
chr6	93965520	93965780	EPHA7
chr6	93967154	93967266	EPHA7
chr6	93967791	93968027	EPHA7
chr6	93969046	93969222	EPHA7
chr6	93973552	93973658	EPHA7
chr6	93974286	93974445	EPHA7
chr6	93979169	93979403	EPHA7
chr6	93981990	93982165	EPHA7
chr6	94066409	94066795	EPHA7
chr6	94067948	94068154	EPHA7
chr6	94120185	94120913	EPHA7
chr6	94124395	94124510	EPHA7
chr6	94128937	94129084	EPHA7
chr6	106534403	106534495	PRDM1
chr6	106536050	106536349	PRDM1
chr6	106543464	106543634	PRDM1
chr6	106546935	106546994	PRDM1
chr6	106547149	106547452	PRDM1
chr6	106552674	106553833	PRDM1
chr6	106554220	106554399	PRDM1
chr6	106554760	106555386	PRDM1
chr6	117609629	117609990	ROS1
chr6	117622111	117622325	ROS1
chr6	117629931	117630116	ROS1
chr6	117631218	117631469	ROS1
chr6	117632157	117632305	ROS1
chr6	117638280	117638460	ROS1
chr6	117639325	117639440	ROS1
chr6	117641005	117641218	ROS1
chr6	117642396	117642582	ROS1
chr6	117645469	117645603	ROS1
chr6	117647361	117647602	ROS1
chr6	117650466	117650634	ROS1
chr6	117658309	117658528	ROS1
chr6	117662272	117662499	ROS1
chr6	117662537	117662820	ROS1

chr6	117663537	117663732	ROS1
chr6	117665197	117665450	ROS1
chr6	117674127	117674357	ROS1
chr6	117677766	117678103	ROS1
chr6	117678941	117679197	ROS1
chr6	117680946	117681199	ROS1
chr6	117681479	117681593	ROS1
chr6	117683740	117684053	ROS1
chr6	117686197	117686392	ROS1
chr6	117686718	117686929	ROS1
chr6	117687213	117687478	ROS1
chr6	117700196	117700347	ROS1
chr6	117704454	117704696	ROS1
chr6	117706820	117707049	ROS1
chr6	117708026	117708187	ROS1
chr6	117708917	117709222	ROS1
chr6	117710487	117711034	ROS1
chr6	117714361	117714509	ROS1
chr6	117715299	117715534	ROS1
chr6	117715753	117715926	ROS1
chr6	117717325	117717452	ROS1
chr6	117718052	117718304	ROS1
chr6	117724276	117724465	ROS1
chr6	117725417	117725616	ROS1
chr6	117730719	117730830	ROS1
chr6	117735656	117735733	ROS1
chr6	117737395	117737505	ROS1
chr6	117739599	117739694	ROS1
chr6	117746671	117746844	ROS1
chr6	135502626	135502699	MYB
chr6	135507015	135507183	MYB
chr6	135508946	135509068	MYB
chr6	135510903	135511046	MYB
chr6	135511239	135511510	MYB
chr6	135513436	135513721	MYB
chr6	135514950	135515081	MYB
chr6	135515468	135515647	MYB
chr6	135516860	135517165	MYB, MYB-AS1
chr6	135518073	135518486	MYB
chr6	135520020	135520213	MYB
chr6	135521197	135521362	MYB
chr6	135521402	135521578	MYB
chr6	135522751	135522912	MYB
chr6	135523526	135523832	MYB
chr6	135524060	135524112	MYB
chr6	135524329	135524517	MYB
chr6	135524828	135524911	MYB
chr6	135538976	135539143	MYB
chr6	138192339	138192684	TNFAIP3
chr6	138195956	138196197	TNFAIP3
chr6	138196799	138196997	TNFAIP3
chr6	138197107	138197328	TNFAIP3
chr6	138197387	138197442	TNFAIP3
chr6	138198187	138198418	TNFAIP3
chr6	138199543	138200513	TNFAIP3
chr6	138201182	138201414	TNFAIP3
chr6	138202146	138202481	TNFAIP3
chr6	152129022	152129524	ESR1
chr6	152163706	152163947	ESR1
chr6	152201764	152201931	ESR1
chr6	152265282	152265668	ESR1
chr6	152332765	152332954	ESR1
chr6	152382100	152382284	ESR1
chr6	152415494	152415728	ESR1
chr6	152419841	152420126	ESR1
chr6	152446375	152446508	ESR1, SYNE1
chr6	157099038	157100630	ARID1B
chr6	157150335	157150580	ARID1B
chr6	157192722	157192811	ARID1B
chr6	157222484	157222684	ARID1B
chr6	157256574	157256735	ARID1B
chr6	157357943	157358035	ARID1B
chr6	157405770	157406064	ARID1B
chr6	157431580	157431720	ARID1B
chr6	157454136	157454366	ARID1B
chr6	157469732	157470171	ARID1B
chr6	157488148	157488344	ARID1B
chr6	157495116	157495276	ARID1B
chr6	157495955	157496164	ARID1B
chr6	157502077	157502337	ARID1B
chr6	157505339	157505594	ARID1B
chr6	157507463	157507764	ARID1B
chr6	157510750	157510939	ARID1B
chr6	157511146	157511369	ARID1B
chr6	157517273	157517474	ARID1B
chr6	157519919	157520066	ARID1B
chr6	157521813	157522647	ARID1B
chr6	157524974	157525155	ARID1B
chr6	157527275	157529050	ARID1B
chr6	160390253	160390452	IGF2R
chr6	160412190	160412380	IGF2R
chr6	160430016	160430191	IGF2R
chr6	160431693	160431842	IGF2R
chr6	160445578	160445761	IGF2R
chr6	160448191	160448371	IGF2R
chr6	160450556	160450712	IGF2R

chr6	160453557	160453770	IGF2R
chr6	160453948	160454164	IGF2R
chr6	160455425	160455579	IGF2R
chr6	160461566	160461781	IGF2R
chr6	160464154	160464345	IGF2R
chr6	160465520	160465714	IGF2R
chr6	160466751	160466939	IGF2R
chr6	160467504	160467702	IGF2R
chr6	160468165	160468393	IGF2R
chr6	160468798	160468964	IGF2R
chr6	160469381	160469600	IGF2R
chr6	160471479	160471709	IGF2R
chr6	160477430	160477582	IGF2R
chr6	160479029	160479181	IGF2R
chr6	160479912	160480155	IGF2R
chr6	160481553	160481774	IGF2R
chr6	160482509	160482703	IGF2R
chr6	160482759	160482985	IGF2R
chr6	160483538	160483676	IGF2R
chr6	160484421	160484687	IGF2R
chr6	160485407	160485588	IGF2R
chr6	160485810	160485958	IGF2R
chr6	160489255	160489442	IGF2R
chr6	160490874	160491115	IGF2R
chr6	160492916	160493093	IGF2R
chr6	160493771	160493941	IGF2R
chr6	160494219	160494526	IGF2R
chr6	160494763	160495032	IGF2R
chr6	160496853	160497053	IGF2R
chr6	160499207	160499419	IGF2R
chr6	160500586	160500844	IGF2R
chr6	160501135	160501332	IGF2R
chr6	160504956	160505241	IGF2R
chr6	160506001	160506188	IGF2R
chr6	160509039	160509204	IGF2R
chr6	160510113	160510310	IGF2R
chr6	160510922	160511160	IGF2R
chr6	160517445	160517682	IGF2R
chr6	160523525	160523728	IGF2R
chr6	160524752	160524872	IGF2R
chr6	160525680	160526141	IGF2R
chr6	161771105	161771268	PRKN
chr6	161781094	161781262	PRKN
chr6	161790979	161791044	PRKN
chr6	161807800	161807934	PRKN
chr6	161966420	161966494	PRKN
chr6	161969860	161970060	PRKN
chr6	161990361	161990473	PRKN
chr6	162204672	162204742	PRKN
chr6	162206778	162206965	PRKN
chr6	162394308	162394474	PRKN
chr6	162475097	162475231	PRKN
chr6	162622137	162622309	PRKN
chr6	162683467	162683822	PRKN
chr6	162864316	162864534	PRKN
chr6	163148668	163148725	PACRG, PRKN
chr6	170591936	170591992	DLL1
chr6	170592050	170592218	DLL1
chr6	170592293	170593142	DLL1
chr6	170593981	170594248	DLL1
chr6	170594316	170594536	DLL1
chr6	170594631	170594812	DLL1
chr6	170595300	170595411	DLL1
chr6	170597301	170597609	DLL1
chr6	170597791	170597902	DLL1
chr6	170598574	170598921	DLL1
chr6	170599148	170599252	DLL1
chr7	2946246	2946501	CARD11
chr7	2949658	2949824	CARD11
chr7	2951780	2951955	CARD11
chr7	2952895	2953125	CARD11
chr7	2954845	2955031	CARD11
chr7	2956898	2957044	CARD11
chr7	2958099	2958246	CARD11
chr7	2958980	2959271	CARD11
chr7	2962242	2962419	CARD11
chr7	2962740	2962992	CARD11
chr7	2963841	2964024	CARD11
chr7	2966347	2966450	CARD11
chr7	2968206	2968357	CARD11
chr7	2969600	2969733	CARD11
chr7	2972143	2972245	CARD11
chr7	2974061	2974288	CARD11
chr7	2976645	2976893	CARD11
chr7	2977515	2977691	CARD11
chr7	2978287	2978490	CARD11
chr7	2979357	2979587	CARD11
chr7	2983820	2984196	CARD11
chr7	2985427	2985615	CARD11
chr7	2987183	2987446	CARD11
chr7	2998108	2998165	CARD11
chr7	6013004	6013198	PMS2
chr7	6017193	6017413	PMS2
chr7	6018201	6018352	PMS2
chr7	6022429	6022647	PMS2
chr7	6026364	6027276	PMS2

chr7	6029405	6029611	PMS2
chr7	6031578	6031713	PMS2
chr7	6035139	6035289	PMS2
chr7	6036931	6037079	PMS2
chr7	6038713	6038931	PMS2
chr7	6042058	6042292	PMS2
chr7	6043295	6043448	PMS2
chr7	6043577	6043714	PMS2
chr7	6045497	6045687	PMS2
chr7	6048602	6048675	PMS2
chr7	6414341	6414426	RAC1
chr7	6426817	6426939	RAC1
chr7	6431529	6431697	RAC1
chr7	6438267	6438374	RAC1
chr7	6439731	6439844	RAC1
chr7	6441473	6441683	RAC1
chr7	6441921	6442102	RAC1
chr7	17338863	17338978	AHR
chr7	17349534	17349772	AHR
chr7	17362099	17362256	AHR
chr7	17367357	17367497	AHR
chr7	17369550	17369724	AHR
chr7	17370354	17370535	AHR
chr7	17373510	17373763	AHR
chr7	17374485	17374645	AHR
chr7	17375243	17375435	AHR
chr7	17378584	17379877	AHR
chr7	17382519	17382713	AHR
chr7	27147508	27148364	HOXA-AS2, HOXA3
chr7	27149708	27150284	HOXA-AS2, HOXA3
chr7	41729222	41730165	INHBA
chr7	41739559	41739997	INHBA, INHBA-AS1
chr7	50358632	50358722	IKZF1
chr7	50367208	50367378	IKZF1
chr7	50435678	50436093	IKZF1
chr7	50444205	50444516	IKZF1
chr7	50450212	50450430	IKZF1
chr7	50455017	50455193	IKZF1
chr7	50459401	50459586	IKZF1
chr7	50467590	50468350	IKZF1
chr7	55086945	55087083	EGFR
chr7	55209953	55210155	EGFR
chr7	55210972	55211206	EGFR
chr7	55214273	55214458	EGFR
chr7	55218961	55219080	EGFR
chr7	55220004	55220064	EGFR
chr7	55220213	55220382	EGFR
chr7	55221678	55221870	EGFR
chr7	55223497	55223664	EGFR
chr7	55224200	55224377	EGFR
chr7	55224426	55224561	EGFR
chr7	55225330	55225471	EGFR
chr7	55227806	55228056	EGFR
chr7	55229166	55229349	EGFR
chr7	55231400	55231541	EGFR
chr7	55232947	55233155	EGFR
chr7	55236190	55236247	EGFR
chr7	55237974	55238262	EGFR
chr7	55238842	55238931	EGFR
chr7	55240513	55240618	EGFR
chr7	55240650	55240842	EGFR
chr7	55241588	55241761	EGFR
chr7	55242389	55242538	EGFR
chr7	55248960	55249196	EGFR, EGFR-AS1
chr7	55259386	55259592	EGFR
chr7	55260433	55260559	EGFR
chr7	55266384	55266581	EGFR
chr7	55267983	55268131	EGFR
chr7	55268855	55269073	EGFR
chr7	55269402	55269500	EGFR
chr7	55270184	55270483	EGFR
chr7	55272923	55273335	EGFR
chr7	77648532	77649318	MAGI2
chr7	77694865	77695004	MAGI2
chr7	77708238	77708427	MAGI2
chr7	77754985	77755179	MAGI2
chr7	77756488	77756758	MAGI2
chr7	77762180	77762402	MAGI2
chr7	77764312	77764548	MAGI2
chr7	77789316	77789615	MAGI2
chr7	77797207	77797450	MAGI2
chr7	77807302	77807444	MAGI2
chr7	77814920	77815012	MAGI2
chr7	77824165	77824405	MAGI2
chr7	77830453	77830535	MAGI2
chr7	77885124	77885923	MAGI2
chr7	77973069	77973302	MAGI2
chr7	77975213	77975385	MAGI2
chr7	77998447	77998555	MAGI2
chr7	78119052	78119182	MAGI2
chr7	78130868	78131129	MAGI2
chr7	78150721	78150987	MAGI2
chr7	78212712	78212796	MAGI2
chr7	78256410	78256580	MAGI2
chr7	78636380	78636547	MAGI2
chr7	79082310	79082661	MAGI2, MAGI2-AS3

chr7	81331871	81332098	HGF
chr7	81334680	81334876	HGF
chr7	81334937	81335094	HGF
chr7	81335577	81335768	HGF
chr7	81336580	81336705	HGF
chr7	81339437	81339584	HGF
chr7	81340771	81340860	HGF
chr7	81346522	81346706	HGF
chr7	81350035	81350188	HGF
chr7	81355180	81355358	HGF
chr7	81358895	81359120	HGF
chr7	81372215	81372291	HGF
chr7	81372643	81372812	HGF
chr7	81374290	81374461	HGF
chr7	81381402	81381603	HGF
chr7	81385281	81385404	HGF
chr7	81386475	81386644	HGF
chr7	81387982	81388145	HGF
chr7	81391997	81392213	HGF
chr7	81399174	81399312	HGF
chr7	87133533	87133790	ABCB1
chr7	87135187	87135384	ABCB1
chr7	87138565	87138822	ABCB1
chr7	87144521	87144769	ABCB1
chr7	87145799	87146006	ABCB1
chr7	87148616	87148807	ABCB1
chr7	87150066	87150217	ABCB1
chr7	87160584	87160838	ABCB1
chr7	87165748	87165882	ABCB1
chr7	87168558	87168686	ABCB1
chr7	87170647	87170805	ABCB1
chr7	87173419	87173616	ABCB1
chr7	87174113	87174340	ABCB1
chr7	87175153	87175365	ABCB1
chr7	87178638	87178859	ABCB1
chr7	87179141	87179395	ABCB1
chr7	87179461	87179637	ABCB1
chr7	87179758	87179919	ABCB1
chr7	87180015	87180179	ABCB1
chr7	87183051	87183273	ABCB1
chr7	87190553	87190728	ABCB1
chr7	87192659	87192723	ABCB1
chr7	87193429	87193493	ABCB1
chr7	87194756	87194823	ABCB1
chr7	87195360	87195582	ABCB1
chr7	87196075	87196317	ABCB1
chr7	87199462	87199564	ABCB1
chr7	87214802	87215021	ABCB1
chr7	87225056	87225155	ABCB1
chr7	87229407	87229525	ABCB1
chr7	92244428	92244625	CDK6
chr7	92247360	92247546	CDK6
chr7	92252324	92252425	CDK6
chr7	92300714	92300874	CDK6
chr7	92354914	92355132	CDK6
chr7	92403984	92404170	CDK6
chr7	92462379	92462662	CDK6
chr7	99245902	99246048	CYP3A5
chr7	99247670	99247880	CYP3A5
chr7	99250150	99250427	CYP3A5
chr7	99258096	99258307	CYP3A5
chr7	99260413	99260530	CYP3A5
chr7	99261565	99261743	CYP3A5
chr7	99262763	99262962	CYP3A5
chr7	99264198	99264337	CYP3A5
chr7	99264549	99264713	CYP3A5
chr7	99269371	99269526	CYP3A5
chr7	99270177	99270327	CYP3A5
chr7	99272130	99272233	CYP3A5
chr7	99273712	99273856	CYP3A5
chr7	99273914	99274005	CYP3A5
chr7	99274125	99274224	CYP3A5
chr7	99277423	99277544	CYP3A5
chr7	99355730	99355876	CYP3A4
chr7	99358416	99358629	CYP3A4
chr7	99359638	99359915	CYP3A4
chr7	99361452	99361663	CYP3A4
chr7	99363974	99364091	CYP3A4
chr7	99364728	99364906	CYP3A4
chr7	99365951	99366150	CYP3A4
chr7	99367365	99367504	CYP3A4
chr7	99367719	99367883	CYP3A4
chr7	99370187	99370337	CYP3A4
chr7	99375625	99375728	CYP3A4
chr7	99377589	99377733	CYP3A4
chr7	99381608	99381729	CYP3A4
chr7	100401057	100401237	EPHB4
chr7	100402762	100402968	EPHB4
chr7	100403097	100403341	EPHB4
chr7	100404016	100404216	EPHB4
chr7	100404961	100405227	EPHB4
chr7	100410343	100410641	EPHB4
chr7	100410691	100410855	EPHB4
chr7	100411248	100411363	EPHB4
chr7	100411515	100411668	EPHB4
chr7	100414788	100415004	EPHB4

chr7	100416116	100416291	EPHB4
chr7	100417153	100417536	EPHB4
chr7	100417737	100417943	EPHB4
chr7	100419867	100420314	EPHB4
chr7	100421240	100421578	EPHB4
chr7	100421799	100421920	EPHB4
chr7	100424575	100424677	EPHB4
chr7	101459285	101459398	CUX1
chr7	101460894	101460974	CUX1
chr7	101559369	101559530	CUX1
chr7	101671352	101671450	CUX1
chr7	101713593	101713722	CUX1
chr7	101740618	101740806	CUX1
chr7	101747590	101747764	CUX1
chr7	101754952	101755079	CUX1
chr7	101758461	101758578	CUX1
chr7	101801814	101801913	CUX1
chr7	101813700	101813855	CUX1
chr7	101821723	101821962	CUX1
chr7	101833067	101833176	CUX1
chr7	101837096	101837195	CUX1
chr7	101838761	101838908	CUX1
chr7	101839888	101840610	CUX1
chr7	101842056	101842172	CUX1
chr7	101843325	101843477	CUX1
chr7	101844614	101845509	CUX1
chr7	101847645	101847861	CUX1
chr7	101848368	101848475	CUX1
chr7	101870621	101870974	CUX1
chr7	101877306	101877545	CUX1
chr7	101882574	101882889	CUX1
chr7	101891666	101892347	CUX1
chr7	101916611	101916789	CUX1
chr7	101917489	101917606	CUX1
chr7	101918492	101918655	CUX1
chr7	101921194	101921361	CUX1
chr7	101923303	101923437	CUX1
chr7	101924070	101924177	CUX1
chr7	101925106	101925237	CUX1
chr7	101925978	101926093	CUX1
chr7	101926287	101926407	CUX1
chr7	106507981	106510026	PIK3CG
chr7	106512956	106513072	PIK3CG
chr7	106513132	106513408	PIK3CG
chr7	106515119	106515273	PIK3CG
chr7	106519938	106520135	PIK3CG
chr7	106522536	106522677	PIK3CG
chr7	106523452	106523633	PIK3CG
chr7	106524574	106524736	PIK3CG
chr7	106526554	106526762	PIK3CG
chr7	106545528	106545857	PIK3CG
chr7	116339113	116340363	MET
chr7	116371696	116371938	MET
chr7	116379978	116380163	MET
chr7	116380880	116381104	MET
chr7	116395383	116395594	MET
chr7	116397465	116397618	MET
chr7	116397666	116397853	MET
chr7	116398487	116398699	MET
chr7	116399365	116399569	MET
chr7	116403078	116403347	MET
chr7	116409673	116409870	MET
chr7	116411526	116411733	MET
chr7	116411877	116412068	MET
chr7	116414909	116415190	MET
chr7	116417417	116417548	MET
chr7	116418804	116419036	MET
chr7	116422016	116422176	MET
chr7	116423332	116423548	MET
chr7	116435683	116435870	MET
chr7	116435915	116436203	MET
chr7	128828967	128829348	SMO
chr7	128843199	128843455	SMO
chr7	128845018	128845278	SMO
chr7	128845425	128845648	SMO
chr7	128845965	128846235	SMO
chr7	128846279	128846453	SMO
chr7	128848574	128848717	SMO
chr7	128849104	128849263	SMO
chr7	128850178	128850414	SMO
chr7	128850780	128850979	SMO
chr7	128851451	128851636	SMO
chr7	128851839	128852317	SMO
chr7	140434371	140434595	BRAF
chr7	140439586	140439771	BRAF
chr7	140449061	140449243	BRAF
chr7	140453049	140453218	BRAF
chr7	140453961	140454058	BRAF
chr7	140476686	140476913	BRAF
chr7	140477765	140477900	BRAF
chr7	140481350	140481518	BRAF
chr7	140482795	140482982	BRAF
chr7	140487322	140487409	BRAF
chr7	140494082	140494292	BRAF
chr7	140500136	140500306	BRAF
chr7	140501186	140501385	BRAF

chr7	140507734	140507887	BRAF
chr7	140508666	140508820	BRAF
chr7	140534383	140534697	BRAF
chr7	140549885	140550037	BRAF
chr7	140624340	140624528	BRAF
chr7	142560960	142561110	EPHB6
chr7	142561363	142561478	EPHB6
chr7	142561698	142562529	EPHB6
chr7	142563204	142563410	EPHB6
chr7	142563689	142564096	EPHB6
chr7	142564210	142564385	EPHB6
chr7	142564635	142564848	EPHB6
chr7	142565337	142565502	EPHB6
chr7	142565726	142565829	EPHB6
chr7	142565970	142566140	EPHB6
chr7	142566221	142566519	EPHB6
chr7	142566701	142566925	EPHB6
chr7	142567544	142567744	EPHB6
chr7	142567941	142568185	EPHB6
chr7	142568257	142568463	EPHB6
chr7	142568523	142568682	EPHB6
chr7	148504712	148504823	EZH2
chr7	148506137	148506272	EZH2
chr7	148506376	148506507	EZH2
chr7	148507399	148507531	EZH2
chr7	148508691	148508837	EZH2
chr7	148511025	148511254	EZH2
chr7	148511980	148512156	EZH2
chr7	148512572	148512663	EZH2
chr7	148513750	148513895	EZH2
chr7	148514288	148514508	EZH2
chr7	148514943	148515234	EZH2
chr7	148516105	148516176	EZH2
chr7	148516662	148516804	EZH2
chr7	148523520	148523749	EZH2
chr7	148524230	148524383	EZH2
chr7	148525806	148525997	EZH2
chr7	148526794	148526965	EZH2
chr7	148529700	148529867	EZH2
chr7	148543536	148543715	EZH2
chr7	148544248	148544415	EZH2
chr7	150642427	150642627	KCNH2
chr7	150643939	150644167	KCNH2
chr7	150644390	150644627	KCNH2
chr7	150644668	150644991	KCNH2
chr7	150645506	150645656	KCNH2
chr7	150645918	150646162	KCNH2
chr7	150646961	150647533	KCNH2
chr7	150647983	150648233	KCNH2
chr7	150648510	150648948	KCNH2
chr7	150649487	150649966	KCNH2
chr7	150652458	150652616	KCNH2
chr7	150654353	150654615	KCNH2
chr7	150655121	150655615	KCNH2
chr7	150656634	150656868	KCNH2
chr7	150671773	150672054	KCNH2
chr7	150674900	150675026	KCNH2
chr7	151833891	151834034	KMT2C
chr7	151835855	151836014	KMT2C
chr7	151836245	151836369	KMT2C
chr7	151836734	151836901	KMT2C
chr7	151841772	151841991	KMT2C
chr7	151842212	151842405	KMT2C
chr7	151843658	151843845	KMT2C
chr7	151845092	151846262	KMT2C
chr7	151847959	151848117	KMT2C
chr7	151848501	151848691	KMT2C
chr7	151849764	151850064	KMT2C
chr7	151851069	151851256	KMT2C
chr7	151851326	151851555	KMT2C
chr7	151852969	151853167	KMT2C
chr7	151853264	151853456	KMT2C
chr7	151854820	151855035	KMT2C
chr7	151855916	151856182	KMT2C
chr7	151859176	151860936	KMT2C
chr7	151864205	151864488	KMT2C
chr7	151866245	151866359	KMT2C
chr7	151868323	151868452	KMT2C
chr7	151871190	151871352	KMT2C
chr7	151873250	151875120	KMT2C
chr7	151876893	151877236	KMT2C
chr7	151877770	151879704	KMT2C
chr7	151880033	151880266	KMT2C
chr7	151882617	151882741	KMT2C
chr7	151884321	151884586	KMT2C
chr7	151884774	151884957	KMT2C
chr7	151891068	151891238	KMT2C
chr7	151891288	151891371	KMT2C
chr7	151891499	151891678	KMT2C
chr7	151892966	151893121	KMT2C
chr7	151896338	151896569	KMT2C
chr7	151899993	151900174	KMT2C
chr7	151902165	151902335	KMT2C
chr7	151904359	151904538	KMT2C
chr7	151917582	151917845	KMT2C
chr7	151919060	151919176	KMT2C

chr7	151919632	151919792	KMT2C
chr7	151921074	151921289	KMT2C
chr7	151921494	151921726	KMT2C
chr7	151926982	151927137	KMT2C
chr7	151927279	151927431	KMT2C
chr7	151932876	151933043	KMT2C
chr7	151935766	151935936	KMT2C
chr7	151944961	151945730	KMT2C
chr7	151946935	151947063	KMT2C
chr7	151947912	151948076	KMT2C
chr7	151948998	151949200	KMT2C
chr7	151949605	151949825	KMT2C
chr7	151960075	151960240	KMT2C
chr7	151962097	151962319	KMT2C
chr7	151970764	151970977	KMT2C
chr7	152007025	152007185	KMT2C
chr7	152008857	152009056	KMT2C
chr7	152012197	152012448	KMT2C
chr7	152027660	152027849	KMT2C
chr7	152055646	152055785	KMT2C
chr7	152132685	152132896	KMT2C
chr8	9413424	9414206	TNKS
chr8	9437643	9437918	TNKS
chr8	9473066	9473212	TNKS
chr8	9537435	9537522	TNKS
chr8	9538209	9538335	TNKS
chr8	9562147	9562292	TNKS
chr8	9563671	9563788	TNKS
chr8	9564295	9564532	TNKS
chr8	9565855	9566027	TNKS
chr8	9567439	9567581	TNKS
chr8	9567626	9567860	TNKS
chr8	9577858	9578080	TNKS
chr8	9584125	9584255	TNKS
chr8	9588374	9588570	TNKS
chr8	9590763	9590979	TNKS
chr8	9592349	9592619	TNKS
chr8	9592861	9593021	TNKS
chr8	9605508	9605747	TNKS
chr8	9609093	9609381	TNKS
chr8	9610028	9610161	TNKS
chr8	9619000	9619171	TNKS
chr8	9620631	9620779	TNKS
chr8	9622200	9622325	TNKS
chr8	9623176	9623332	TNKS
chr8	9623723	9623960	TNKS
chr8	9627590	9627797	TNKS
chr8	9634134	9634271	TNKS
chr8	18257488	18258411	NAT2
chr8	37654761	37655077	ADGRA2
chr8	37672388	37672510	ADGRA2
chr8	37686380	37686502	ADGRA2
chr8	37686753	37686875	ADGRA2
chr8	37686980	37687102	ADGRA2
chr8	37687343	37687557	ADGRA2
chr8	37688202	37688466	ADGRA2
chr8	37688915	37689130	ADGRA2
chr8	37690502	37690751	ADGRA2
chr8	37691180	37691380	ADGRA2
chr8	37691459	37691671	ADGRA2
chr8	37692666	37692941	ADGRA2
chr8	37693046	37693313	ADGRA2
chr8	37695223	37695482	ADGRA2
chr8	37696448	37696626	ADGRA2
chr8	37696991	37697165	ADGRA2
chr8	37697613	37697809	ADGRA2
chr8	37698252	37698392	ADGRA2
chr8	37698578	37699898	ADGRA2
chr8	38271120	38271347	FGFR1
chr8	38271410	38271566	FGFR1
chr8	38271644	38271832	FGFR1
chr8	38272051	38272172	FGFR1
chr8	38272271	38272444	FGFR1
chr8	38273337	38273603	FGFR1
chr8	38274798	38274959	FGFR1
chr8	38275362	38275534	FGFR1
chr8	38275720	38275916	FGFR1
chr8	38277025	38277278	FGFR1
chr8	38279289	38279484	FGFR1
chr8	38280517	38280718	FGFR1
chr8	38282001	38282242	FGFR1
chr8	38283614	38283788	FGFR1
chr8	38285413	38285636	FGFR1
chr8	38285838	38285987	FGFR1
chr8	38287174	38287491	FGFR1
chr8	38297798	38297915	FGFR1
chr8	38314848	38315077	FGFR1
chr8	38318588	38318649	FGFR1
chr8	48685749	48685836	PRKDC
chr8	48686708	48686963	PRKDC
chr8	48689379	48689569	PRKDC
chr8	48690221	48690460	PRKDC
chr8	48690994	48691246	PRKDC
chr8	48691263	48691385	PRKDC
chr8	48691539	48691679	PRKDC
chr8	48694697	48694840	PRKDC

chr8	48694913	48695184	PRKDC
chr8	48696277	48696395	PRKDC
chr8	48697648	48697903	PRKDC
chr8	48701441	48701635	PRKDC
chr8	48701686	48701824	PRKDC
chr8	48706825	48707087	PRKDC
chr8	48710772	48710983	PRKDC
chr8	48711745	48711976	PRKDC
chr8	48713328	48713572	PRKDC
chr8	48715841	48716066	PRKDC
chr8	48719672	48719912	PRKDC
chr8	48729985	48730147	PRKDC
chr8	48731937	48732096	PRKDC
chr8	48733254	48733529	PRKDC
chr8	48734139	48734378	PRKDC
chr8	48736393	48736582	PRKDC
chr8	48739191	48739447	PRKDC
chr8	48740703	48740933	PRKDC
chr8	48743140	48743322	PRKDC
chr8	48744349	48744512	PRKDC
chr8	48746731	48746982	PRKDC
chr8	48748873	48749113	PRKDC
chr8	48749747	48750005	PRKDC
chr8	48751683	48751832	PRKDC
chr8	48752551	48752775	PRKDC
chr8	48761689	48761889	PRKDC
chr8	48761914	48762089	PRKDC
chr8	48765208	48765370	PRKDC
chr8	48766618	48766800	PRKDC
chr8	48767757	48767959	PRKDC
chr8	48769691	48769885	PRKDC
chr8	48771051	48771221	PRKDC
chr8	48771384	48771572	PRKDC
chr8	48772146	48772345	PRKDC
chr8	48773434	48773557	PRKDC
chr8	48774597	48774713	PRKDC
chr8	48774908	48775127	PRKDC
chr8	48775934	48776163	PRKDC
chr8	48777091	48777349	PRKDC
chr8	48790259	48790437	PRKDC
chr8	48792026	48792244	PRKDC
chr8	48793951	48794106	PRKDC
chr8	48794447	48794683	PRKDC
chr8	48798479	48798733	PRKDC
chr8	48800082	48800291	PRKDC
chr8	48801053	48801236	PRKDC
chr8	48801549	48801808	PRKDC
chr8	48802792	48803066	PRKDC
chr8	48805674	48805972	PRKDC
chr8	48809695	48809879	PRKDC
chr8	48811004	48811154	PRKDC
chr8	48812907	48813052	PRKDC
chr8	48815103	48815380	PRKDC
chr8	48817403	48817561	PRKDC
chr8	48824944	48825147	PRKDC
chr8	48826435	48826649	PRKDC
chr8	48827862	48828003	PRKDC
chr8	48830811	48830968	PRKDC
chr8	48839728	48839938	PRKDC
chr8	48840305	48840475	PRKDC
chr8	48841626	48841763	PRKDC
chr8	48842387	48842597	PRKDC
chr8	48843206	48843372	PRKDC
chr8	48845554	48845757	PRKDC
chr8	48846499	48846675	PRKDC
chr8	48847543	48847643	PRKDC
chr8	48848266	48848485	PRKDC
chr8	48848887	48849102	PRKDC
chr8	48852085	48852282	PRKDC
chr8	48855743	48855951	PRKDC
chr8	48856387	48856468	PRKDC
chr8	48856508	48856614	PRKDC
chr8	48866154	48866304	PRKDC
chr8	48866341	48866504	PRKDC
chr8	48866872	48867031	PRKDC
chr8	48868408	48868533	PRKDC
chr8	48869705	48869848	PRKDC
chr8	48869889	48870016	PRKDC
chr8	48872507	48872711	PRKDC
chr8	56854393	56854575	LYN
chr8	56858981	56859077	LYN
chr8	56860151	56860307	LYN
chr8	56862992	56863141	LYN
chr8	56863214	56863368	LYN
chr8	56864499	56864699	LYN
chr8	56866365	56866568	LYN
chr8	56879248	56879481	LYN
chr8	56882250	56882377	LYN
chr8	56910879	56911083	LYN
chr8	56911951	56912133	LYN
chr8	56922441	56922694	LYN
chr8	92972444	92972770	RUNX1T1
chr8	92982860	92983101	RUNX1T1
chr8	92988107	92988226	RUNX1T1
chr8	92998326	92998578	RUNX1T1
chr8	92999089	92999225	RUNX1T1

chr8	93003841	93004142	RUNX1T1
chr8	93017314	93017550	RUNX1T1
chr8	93023204	93023344	RUNX1T1
chr8	93026781	93027073	RUNX1T1
chr8	93029428	93029616	RUNX1T1
chr8	93074748	93074805	RUNX1T1
chr8	93088167	93088305	RUNX1T1
chr8	93107230	93107424	RUNX1T1
chr8	93107549	93107720	RUNX1T1
chr8	93115059	93115137	RUNX1T1
chr8	128748814	128748894	MYC
chr8	128750468	128751290	MYC
chr8	128752616	128753229	MYC
chr9	5021962	5022238	JAK2
chr9	5029757	5029931	JAK2
chr9	5044377	5044545	JAK2
chr9	5050660	5050856	JAK2
chr9	5054537	5054909	JAK2
chr9	5055643	5055813	JAK2
chr9	5064857	5065065	JAK2
chr9	5066652	5066814	JAK2
chr9	5068996	5069233	JAK2
chr9	5069899	5070077	JAK2
chr9	5072466	5072651	JAK2
chr9	5073672	5073810	JAK2
chr9	5077427	5077605	JAK2
chr9	5078280	5078469	JAK2
chr9	5080203	5080405	JAK2
chr9	5080507	5080708	JAK2
chr9	5081699	5081886	JAK2
chr9	5089648	5089888	JAK2
chr9	5090420	5090595	JAK2
chr9	5090713	5090936	JAK2
chr9	5122978	5123146	JAK2
chr9	5126307	5126471	JAK2
chr9	5126658	5126816	JAK2
chr9	8317848	8317967	PTPRD
chr9	8319805	8319991	PTPRD
chr9	8331556	8331761	PTPRD
chr9	8338896	8339072	PTPRD
chr9	8340317	8340494	PTPRD
chr9	8341064	8341293	PTPRD
chr9	8341667	8342003	PTPRD
chr9	8375910	8376115	PTPRD
chr9	8376581	8376751	PTPRD
chr9	8389206	8389432	PTPRD
chr9	8404511	8404685	PTPRD
chr9	8436566	8436714	PTPRD
chr9	8437172	8437264	PTPRD
chr9	8449699	8449870	PTPRD
chr9	8454554	8454619	PTPRD
chr9	8460251	8460305	PTPRD
chr9	8460385	8460596	PTPRD
chr9	8465440	8465700	PTPRD
chr9	8470969	8471110	PTPRD
chr9	8484093	8484403	PTPRD
chr9	8485201	8485352	PTPRD
chr9	8485736	8486374	PTPRD
chr9	8486430	8486484	PTPRD
chr9	8492836	8493004	PTPRD
chr9	8497216	8497293	PTPRD
chr9	8499621	8499865	PTPRD
chr9	8500728	8501084	PTPRD
chr9	8504235	8504430	PTPRD
chr9	8507275	8507459	PTPRD
chr9	8517822	8518454	PTPRD
chr9	8521251	8521571	PTPRD
chr9	8523487	8523549	PTPRD
chr9	8524753	8524815	PTPRD
chr9	8524899	8525071	PTPRD
chr9	8525142	8525211	PTPRD
chr9	8526601	8526669	PTPRD
chr9	8527319	8527378	PTPRD
chr9	8528399	8528517	PTPRD
chr9	8528555	8528804	PTPRD
chr9	8633291	8633483	PTPRD
chr9	8636673	8636869	PTPRD
chr9	8733754	8733868	PTPRD
chr9	21968202	21968266	CDKN2A
chr9	21968698	21968795	CDKN2A
chr9	21970875	21971232	CDKN2A
chr9	21974450	21974851	CDKN2A
chr9	21994112	21994478	CDKN2A
chr9	22005960	22006271	CDKN2B, CDKN2B-AS1
chr9	22008690	22008977	CDKN2B, CDKN2B-AS1
chr9	35074079	35074238	FANCG
chr9	35074342	35074516	FANCG
chr9	35074898	35075104	FANCG
chr9	35075250	35075347	FANCG
chr9	35075436	35075776	FANCG
chr9	35075933	35076050	FANCG
chr9	35076403	35076605	FANCG
chr9	35076695	35076892	FANCG
chr9	35076942	35077123	FANCG
chr9	35077235	35077421	FANCG
chr9	35078112	35078365	FANCG

chr9	35078576	35078758	FANCG
chr9	35079122	35079263	FANCG
chr9	35079412	35079546	FANCG
chr9	36840500	36840658	PAX5
chr9	36846814	36846951	PAX5
chr9	36881975	36882127	PAX5
chr9	36923326	36923506	PAX5
chr9	36930859	36930966	PAX5
chr9	36966520	36966746	PAX5
chr9	37002619	37002798	PAX5
chr9	37006444	37006559	PAX5
chr9	37014968	37015216	PAX5
chr9	37020607	37020823	PAX5
chr9	37033957	37034053	PAX5
chr9	75516098	75516221	ALDH1A1
chr9	75520848	75520973	ALDH1A1
chr9	75524492	75524700	ALDH1A1
chr9	75526848	75527063	ALDH1A1
chr9	75531810	75532045	ALDH1A1
chr9	75533610	75533763	ALDH1A1
chr9	75538909	75539073	ALDH1A1
chr9	75540374	75540553	ALDH1A1
chr9	75542006	75542118	ALDH1A1
chr9	75543782	75543962	ALDH1A1
chr9	75545769	75545960	ALDH1A1
chr9	75555038	75555193	ALDH1A1
chr9	75567825	75567983	ALDH1A1
chr9	80336213	80336454	GNAQ
chr9	80343404	80343608	GNAQ
chr9	80409353	80409533	GNAQ
chr9	80412410	80412589	GNAQ
chr9	80430506	80430711	GNAQ
chr9	80537051	80537286	GNAQ
chr9	80645990	80646176	GNAQ
chr9	87285638	87285900	NTRK2
chr9	87317048	87317173	NTRK2
chr9	87317237	87317359	NTRK2
chr9	87322733	87322852	NTRK2
chr9	87325526	87325731	NTRK2
chr9	87338462	87338649	NTRK2
chr9	87339113	87339296	NTRK2
chr9	87342543	87342899	NTRK2
chr9	87356781	87356867	NTRK2
chr9	87359862	87360013	NTRK2
chr9	87366875	87367025	NTRK2
chr9	87425431	87425519	NTRK2
chr9	87475929	87476027	NTRK2
chr9	87482132	87482371	NTRK2
chr9	87486678	87486757	NTRK2
chr9	87549051	87549232	NTRK2
chr9	87563351	87563574	NTRK2
chr9	87570172	87570457	NTRK2
chr9	87635095	87635304	NTRK2
chr9	87636141	87636377	NTRK2
chr9	93606155	93606622	SYK
chr9	93607690	93607901	SYK
chr9	93624462	93624651	SYK
chr9	93626845	93626974	SYK
chr9	93627304	93627404	SYK
chr9	93629387	93629506	SYK
chr9	93636460	93636598	SYK
chr9	93636928	93637156	SYK
chr9	93639827	93640087	SYK
chr9	93641020	93641260	SYK
chr9	93650005	93650196	SYK
chr9	93650771	93650934	SYK
chr9	93657784	93657907	SYK
chr9	97863963	97864157	FANCC
chr9	97869322	97869576	FANCC
chr9	97873452	97873652	FANCC
chr9	97873719	97873944	FANCC
chr9	97876885	97877017	FANCC
chr9	97879571	97879697	FANCC
chr9	97887342	97887492	FANCC
chr9	97888785	97888888	FANCC
chr9	97897602	97897809	FANCC
chr9	97912179	97912394	FANCC
chr9	97933335	97933450	FANCC
chr9	97934293	97934454	FANCC
chr9	98002905	98003050	FANCC
chr9	98009688	98009823	FANCC
chr9	98011383	98011598	FANCC
chr9	98208639	98208698	PTCH1
chr9	98209167	98209758	PTCH1
chr9	98211325	98211630	PTCH1
chr9	98212097	98212247	PTCH1
chr9	98215734	98215927	PTCH1
chr9	98218532	98218720	PTCH1
chr9	98220269	98220600	PTCH1
chr9	98221856	98222090	PTCH1
chr9	98224112	98224305	PTCH1
chr9	98229372	98229732	PTCH1
chr9	98231007	98231460	PTCH1
chr9	98232069	98232238	PTCH1
chr9	98236268	98236354	PTCH1
chr9	98238290	98238466	PTCH1

chr9	98239015	98239164	PTCH1
chr9	98239803	98240009	PTCH1
chr9	98240311	98240493	PTCH1
chr9	98241256	98241454	PTCH1
chr9	98242225	98242397	PTCH1
chr9	98242646	98242895	PTCH1
chr9	98244205	98244347	PTCH1
chr9	98244390	98244510	PTCH1
chr9	98247941	98248181	PTCH1
chr9	98268663	98268906	PTCH1
chr9	98270417	98270668	PTCH1
chr9	98278725	98278778	PTCH1
chr9	98278879	98279127	PTCH1
chr9	133589681	133589867	
chr9	133709394	133709456	
chr9	133710235	133710294	
chr9	133710674	133710937	ABL1
chr9	133729425	133729649	ABL1
chr9	133730162	133730508	ABL1
chr9	133738124	133738447	ABL1
chr9	133747490	133747625	ABL1
chr9	133748221	133748449	ABL1
chr9	133750229	133750464	ABL1
chr9	133753776	133753979	ABL1
chr9	133755429	133755569	ABL1
chr9	133755861	133756076	ABL1
chr9	133759330	133761095	ABL1
chr9	135771596	135772166	TSC1
chr9	135772545	135772757	TSC1
chr9	135772784	135773022	TSC1
chr9	135776076	135776249	TSC1
chr9	135776950	135777111	TSC1
chr9	135777966	135778199	TSC1
chr9	135779012	135779229	TSC1
chr9	135779772	135779866	TSC1
chr9	135780942	135781551	TSC1
chr9	135782092	135782247	TSC1
chr9	135782662	135782782	TSC1
chr9	135785932	135786104	TSC1
chr9	135786363	135786525	TSC1
chr9	135786742	135786980	TSC1
chr9	135787643	135787869	TSC1
chr9	135796724	135796848	TSC1
chr9	135797180	135797385	TSC1
chr9	135798709	135798904	TSC1
chr9	135800948	135801151	TSC1
chr9	135802562	135802716	TSC1
chr9	135804128	135804284	TSC1
chr9	139390497	139392035	NOTCH1
chr9	139393325	139393473	NOTCH1
chr9	139393538	139393736	NOTCH1
chr9	139394978	139395324	NOTCH1
chr9	139396174	139396390	NOTCH1
chr9	139396427	139396565	NOTCH1
chr9	139396698	139396965	NOTCH1
chr9	139397608	139397807	NOTCH1
chr9	139399099	139399581	NOTCH1
chr9	139399736	139400358	NOTCH1
chr9	139400953	139401116	NOTCH1
chr9	139401142	139401450	NOTCH1
chr9	139401731	139401914	NOTCH1
chr9	139402381	139402616	NOTCH1
chr9	139402658	139402862	NOTCH1
chr9	139403296	139403548	NOTCH1
chr9	139404159	139404438	NOTCH1
chr9	139405079	139405282	NOTCH1
chr9	139405578	139405748	NOTCH1
chr9	139407447	139407611	NOTCH1
chr9	139407818	139408014	NOTCH1
chr9	139408936	139409179	NOTCH1
chr9	139409716	139409877	NOTCH1
chr9	139409909	139410193	NOTCH1
chr9	139410407	139410571	NOTCH1
chr9	139411698	139411862	NOTCH1
chr9	139412178	139412414	NOTCH1
chr9	139412563	139412769	NOTCH1
chr9	139413017	139413301	NOTCH1
chr9	139413869	139414042	MIR4673, NOTCH1
chr9	139417276	139417665	NOTCH1
chr9	139418143	139418456	NOTCH1
chr9	139438450	139438579	NOTCH1
chr9	139440152	139440263	NOTCH1
chr10	8097593	8097884	GATA3
chr10	8100242	8100829	GATA3
chr10	8105930	8106126	GATA3
chr10	8111410	8111588	GATA3
chr10	8115676	8116011	GATA3
chr10	43572681	43572804	RET
chr10	43595881	43596195	RET
chr10	43597764	43598102	RET
chr10	43600374	43600666	RET
chr10	43601798	43602044	RET
chr10	43604453	43604703	RET
chr10	43606629	43606938	RET
chr10	43607521	43607697	RET
chr10	43608275	43608436	RET

chr10	43608978	43609148	RET
chr10	43609902	43610209	RET
chr10	43612006	43612204	RET
chr10	43613795	43613953	RET
chr10	43614953	43615218	RET
chr10	43615503	43615676	RET
chr10	43617368	43617489	RET
chr10	43619093	43619281	RET
chr10	43620305	43620455	RET
chr10	43621997	43622227	RET
chr10	43623534	43623742	RET
chr10	45869702	45869902	ALOX5
chr10	45877905	45878154	ALOX5
chr10	45891277	45891409	ALOX5
chr10	45907613	45907786	ALOX5
chr10	45919463	45919620	ALOX5
chr10	45920382	45920605	ALOX5
chr10	45924040	45924237	ALOX5
chr10	45935852	45936106	ALOX5
chr10	45936766	45936903	ALOX5
chr10	45938460	45938689	ALOX5
chr10	45938838	45939010	ALOX5
chr10	45939150	45939301	ALOX5
chr10	45939538	45939759	ALOX5
chr10	45940930	45941160	ALOX5
chr10	89624201	89624330	PTEN
chr10	89653756	89653891	PTEN
chr10	89685244	89685339	PTEN
chr10	89690777	89690871	PTEN
chr10	89692744	89693033	PTEN
chr10	89711849	89712041	PTEN
chr10	89717584	89717801	PTEN
chr10	89720625	89720900	PTEN
chr10	89725018	89725254	PTEN
chr10	93558422	93558671	TNKS2
chr10	93572714	93572989	TNKS2
chr10	93576865	93577011	TNKS2
chr10	93579001	93579088	TNKS2
chr10	93579213	93579339	TNKS2
chr10	93579670	93579815	TNKS2
chr10	93582027	93582144	TNKS2
chr10	93586748	93586985	TNKS2
chr10	93588016	93588188	TNKS2
chr10	93590654	93590796	TNKS2
chr10	93590834	93590963	TNKS2
chr10	93593584	93593806	TNKS2
chr10	93596650	93596780	TNKS2
chr10	93600292	93600488	TNKS2
chr10	93601014	93601230	TNKS2
chr10	93601903	93602173	TNKS2
chr10	93604650	93604810	TNKS2
chr10	93605483	93605722	TNKS2
chr10	93608114	93608417	TNKS2
chr10	93609243	93609376	TNKS2
chr10	93610947	93611118	TNKS2
chr10	93614760	93614908	TNKS2
chr10	93615345	93615470	TNKS2
chr10	93617156	93617312	TNKS2
chr10	93619193	93619430	TNKS2
chr10	93621730	93621937	TNKS2
chr10	93622668	93622781	TNKS2
chr10	96522437	96522655	CYP2C19
chr10	96534789	96535002	CYP2C19
chr10	96535121	96535321	CYP2C19
chr10	96540230	96540441	CYP2C19
chr10	96541552	96541779	CYP2C19
chr10	96580227	96580419	CYP2C19
chr10	96602568	96602806	CYP2C19
chr10	96609648	96609840	CYP2C19
chr10	96612464	96612696	CYP2C19
chr10	96698414	96698632	CYP2C9
chr10	96701589	96701802	CYP2C9
chr10	96701923	96702123	CYP2C9
chr10	96707510	96707721	CYP2C9
chr10	96708839	96709066	CYP2C9
chr10	96731835	96732027	CYP2C9
chr10	96740914	96741152	CYP2C9
chr10	96745764	96745956	CYP2C9
chr10	96748578	96748810	CYP2C9
chr10	96749200	96749335	
chr10	96796859	96797091	CYP2C8
chr10	96798628	96798820	CYP2C8
chr10	96800659	96800742	CYP2C8
chr10	96802621	96802859	CYP2C8
chr10	96805541	96805733	CYP2C8
chr10	96818066	96818293	CYP2C8
chr10	96824531	96824742	CYP2C8
chr10	96826939	96827473	CYP2C8
chr10	96828928	96829184	CYP2C8
chr10	104263884	104264116	SUFU
chr10	104268900	104269085	SUFU
chr10	104309701	104309888	SUFU
chr10	104352313	104352506	SUFU
chr10	104353367	104353503	SUFU
chr10	104353724	104353847	SUFU
chr10	104356871	104357075	SUFU

chr10	104359164	104359326	SUFU
chr10	104374999	104375184	SUFU
chr10	104377021	104377210	SUFU
chr10	104378797	104378853	SUFU
chr10	104386906	104387025	SUFU
chr10	104389797	104389937	SUFU
chr10	104849403	104849690	CNNM2, NT5C2
chr10	104850342	104850569	NT5C2
chr10	104850667	104850778	NT5C2
chr10	104851295	104851397	NT5C2
chr10	104852870	104853091	NT5C2
chr10	104853703	104853820	NT5C2
chr10	104854079	104854237	NT5C2
chr10	104855670	104855762	NT5C2
chr10	104857022	104857156	NT5C2
chr10	104857775	104857870	NT5C2
chr10	104858662	104858766	NT5C2
chr10	104859657	104859801	NT5C2
chr10	104860394	104860524	NT5C2
chr10	104860776	104860884	NT5C2
chr10	104860966	104861108	NT5C2
chr10	104865089	104865163	NT5C2
chr10	104865437	104865583	NT5C2
chr10	104866320	104866488	NT5C2
chr10	104899137	104899261	NT5C2
chr10	104913022	104913086	NT5C2
chr10	104934589	104934740	NT5C2
chr10	115803866	115805350	ADRB1
chr10	123239069	123239209	FGFR2
chr10	123239345	123239652	FGFR2
chr10	123241660	123241716	FGFR2
chr10	123243186	123243342	FGFR2
chr10	123244883	123245071	FGFR2
chr10	123246842	123246963	FGFR2
chr10	123247479	123247652	FGFR2
chr10	123256020	123256261	FGFR2
chr10	123257983	123258144	FGFR2
chr10	123260314	123260486	FGFR2
chr10	123263278	123263480	FGFR2
chr10	123274605	123274858	FGFR2
chr10	123276807	123277002	FGFR2
chr10	123277535	123277594	FGFR2
chr10	123278170	123278368	FGFR2
chr10	123279467	123279708	FGFR2
chr10	123297882	123297949	FGFR2
chr10	123298080	123298254	FGFR2
chr10	123310778	123310998	FGFR2
chr10	123323990	123324118	FGFR2
chr10	123324926	123325243	FGFR2
chr10	123353197	123353356	FGFR2
chr10	135340874	135341101	CYP2E1, SPRN
chr10	135341959	135342169	CYP2E1, SPRN
chr10	135345063	135345263	CYP2E1, SPRN
chr10	135345602	135345813	CYP2E1, SPRN
chr10	135346170	135346397	CYP2E1, SPRN
chr10	135347234	135347426	CYP2E1, SPRN
chr10	135350541	135350779	CYP2E1, SPRN
chr10	135351229	135351421	CYP2E1, SPRN
chr10	135352258	135352493	CYP2E1, SPRN
chr11	532610	532780	HRAS
chr11	533270	533383	HRAS
chr11	533427	533637	HRAS
chr11	533740	533969	HRAS
chr11	534186	534347	HRAS
chr11	8246137	8246293	LMO1
chr11	8248496	8248672	LMO1
chr11	8251812	8252076	LMO1
chr11	8284859	8284934	LMO1
chr11	8289948	8290020	LMO1
chr11	17408440	17409663	KCNJ11
chr11	22646206	22647381	FANCF
chr11	32410578	32410750	WT1
chr11	32413492	32413635	WT1
chr11	32414186	32414326	WT1
chr11	32417777	32417978	WT1
chr11	32421468	32421615	WT1
chr11	32438010	32438111	WT1
chr11	32439097	32439225	WT1
chr11	32449476	32449629	WT1
chr11	32450017	32450190	WT1
chr11	32452050	32452110	WT1
chr11	32456220	32456916	WT1
chr11	60229822	60230031	MS4A1
chr11	60230449	60230619	MS4A1
chr11	60231735	60231842	MS4A1
chr11	60233368	60233655	MS4A1
chr11	60234406	60234558	MS4A1
chr11	60235697	60235966	MS4A1
chr11	64571780	64572313	MEN1
chr11	64572480	64572695	MEN1
chr11	64573081	64573267	MEN1
chr11	64573678	64573865	MEN1
chr11	64574457	64574595	MEN1
chr11	64574625	64574716	MEN1
chr11	64574998	64575177	MEN1
chr11	64575337	64575596	MEN1

chr11	64577096	64577606	MEN1
chr11	67351289	67351340	GSTP1
chr11	67351579	67351665	GSTP1
chr11	67351909	67352066	GSTP1
chr11	67352130	67352268	GSTP1
chr11	67352583	67352737	GSTP1
chr11	67353549	67353707	GSTP1
chr11	67353834	67354073	GSTP1
chr11	69456056	69456304	CCND1
chr11	69457773	69458039	CCND1
chr11	69458574	69458784	CCND1
chr11	69462736	69462935	CCND1
chr11	69465860	69466075	CCND1
chr11	94153265	94153372	MRE11
chr11	94163051	94163177	MRE11
chr11	94168972	94169090	MRE11
chr11	94170317	94170426	MRE11
chr11	94178950	94179084	MRE11
chr11	94180359	94180629	MRE11
chr11	94189416	94189529	MRE11
chr11	94192548	94192772	MRE11
chr11	94194076	94194227	MRE11
chr11	94197253	94197430	MRE11
chr11	94200953	94201084	MRE11
chr11	94203611	94203833	MRE11
chr11	94204714	94204950	MRE11
chr11	94208753	94208810	MRE11
chr11	94209429	94209594	MRE11
chr11	94211875	94212067	MRE11
chr11	94212814	94212952	MRE11
chr11	94219064	94219275	MRE11
chr11	94223973	94224156	MRE11
chr11	94225782	94225861	MRE11
chr11	94225922	94225992	MRE11
chr11	100909821	100910027	PGR
chr11	100912650	100912858	PGR
chr11	100920634	100920815	PGR
chr11	100921654	100921705	PGR
chr11	100922129	100922324	PGR
chr11	100933152	100933508	PGR
chr11	100962465	100962632	PGR
chr11	100996712	100996914	PGR
chr11	100998139	100999826	PGR
chr11	106558249	106558507	GUCY1A2
chr11	106579212	106579417	GUCY1A2
chr11	106617251	106617394	GUCY1A2
chr11	106647139	106647333	GUCY1A2
chr11	106680693	106681229	GUCY1A2
chr11	106807350	106807463	GUCY1A2
chr11	106810160	106810929	GUCY1A2
chr11	106849319	106849491	GUCY1A2
chr11	106856770	106856882	GUCY1A2
chr11	106888453	106888806	GUCY1A2
chr11	108098326	108098448	ATM
chr11	108098477	108098640	ATM
chr11	108099879	108100075	ATM
chr11	108106371	108106586	ATM
chr11	108114654	108114870	ATM
chr11	108115489	108115778	ATM
chr11	108117665	108117879	ATM
chr11	108119634	108119854	ATM
chr11	108121402	108121824	ATM
chr11	108122538	108122783	ATM
chr11	108123518	108123664	ATM
chr11	108124515	108124791	ATM
chr11	108126916	108127092	ATM
chr11	108128182	108128358	ATM
chr11	108129687	108129827	ATM
chr11	108137872	108138094	ATM
chr11	108139111	108139361	ATM
chr11	108141765	108141898	ATM
chr11	108141952	108142158	ATM
chr11	108143233	108143359	ATM
chr11	108143423	108143604	ATM
chr11	108150192	108150360	ATM
chr11	108151696	108151920	ATM
chr11	108153411	108153631	ATM
chr11	108154928	108155225	ATM
chr11	108158301	108158467	ATM
chr11	108159678	108159855	ATM
chr11	108160303	108160553	ATM
chr11	108163320	108163545	ATM
chr11	108164014	108164229	ATM
chr11	108165628	108165811	ATM
chr11	108167988	108168134	ATM
chr11	108170415	108170637	ATM
chr11	108172349	108172541	ATM
chr11	108173554	108173781	ATM
chr11	108175376	108175604	ATM
chr11	108178598	108178736	ATM
chr11	108180861	108181067	ATM, C11ORF65
chr11	108183112	108183250	ATM, C11ORF65
chr11	108186524	108186663	ATM, C11ORF65
chr11	108186712	108186865	ATM, C11ORF65
chr11	108188074	108188273	ATM, C11ORF65
chr11	108190655	108190810	ATM, C11ORF65

chr11	108192002	108192172	ATM, C11ORF65
chr11	108196011	108196296	ATM, C11ORF65
chr11	108196759	108196977	ATM, C11ORF65
chr11	108198346	108198510	ATM, C11ORF65
chr11	108199722	108199990	ATM, C11ORF65
chr11	108200915	108201173	ATM, C11ORF65
chr11	108202145	108202309	ATM, C11ORF65
chr11	108202580	108202789	ATM, C11ORF65
chr11	108203463	108203652	ATM, C11ORF65
chr11	108204587	108204720	ATM, C11ORF65
chr11	108205670	108205861	ATM, C11ORF65
chr11	108206546	108206713	ATM, C11ORF65
chr11	108213923	108214123	ATM, C11ORF65
chr11	108216444	108216660	ATM, C11ORF65
chr11	108217980	108218117	ATM, C11ORF65
chr11	108224467	108224632	ATM, C11ORF65
chr11	108225512	108225626	ATM, C11ORF65
chr11	108235783	108235970	ATM, C11ORF65
chr11	108236026	108236260	ATM, C11ORF65
chr11	113281423	113281667	DRD2
chr11	113283252	113283630	DRD2
chr11	113284031	113284087	DRD2
chr11	113285071	113285208	DRD2
chr11	113286117	113286358	DRD2
chr11	113287559	113287746	DRD2
chr11	113288723	113288883	DRD2
chr11	113295059	113295398	DRD2
chr11	118307202	118307684	KMT2A
chr11	118309713	118309862	KMT2A
chr11	118339435	118339584	KMT2A
chr11	118342351	118345055	KMT2A
chr11	118347494	118347722	KMT2A
chr11	118348656	118348941	KMT2A
chr11	118350863	118350978	KMT2A
chr11	118352404	118352832	KMT2A
chr11	118353111	118353235	KMT2A
chr11	118354872	118355087	KMT2A
chr11	118355551	118355715	KMT2A
chr11	118359183	118359239	KMT2A
chr11	118359303	118359500	KMT2A
chr11	118360481	118360627	KMT2A
chr11	118360818	118360989	KMT2A
chr11	118361885	118362058	KMT2A
chr11	118362433	118362668	KMT2A
chr11	118363746	118363970	KMT2A
chr11	118364977	118365138	KMT2A
chr11	118365383	118365507	KMT2A
chr11	118366389	118366633	KMT2A
chr11	118366950	118367107	KMT2A
chr11	118368625	118368813	KMT2A
chr11	118369059	118369268	KMT2A
chr11	118369992	118370160	KMT2A
chr11	118370524	118370653	KMT2A
chr11	118371676	118371887	KMT2A
chr11	118372361	118372597	KMT2A
chr11	118373087	118377386	KMT2A
chr11	118378218	118378349	KMT2A
chr11	118379825	118379940	KMT2A
chr11	118380637	118380858	KMT2A
chr11	118382640	118382765	KMT2A
chr11	118390307	118390532	KMT2A
chr11	118390646	118390804	KMT2A
chr11	118391491	118391625	KMT2A
chr11	118391977	118392157	KMT2A
chr11	118392586	118392912	KMT2A
chr11	119077102	119077347	CBL
chr11	119103132	119103430	CBL
chr11	119142419	119142616	CBL
chr11	119144552	119144759	CBL
chr11	119145516	119145688	CBL
chr11	119146681	119146869	CBL
chr11	119148441	119148579	CBL
chr11	119148850	119149032	CBL
chr11	119149194	119149448	CBL
chr11	119155653	119155835	CBL
chr11	119155873	119156301	CBL
chr11	119158536	119158681	CBL
chr11	119167602	119167769	CBL
chr11	119168068	119168216	CBL
chr11	119169042	119169275	CBL
chr11	119170179	119170516	CBL
chr11	125495630	125495932	CHEK1
chr11	125496618	125496753	CHEK1
chr11	125497476	125497750	CHEK1
chr11	125499101	125499216	CHEK1
chr11	125499260	125499380	CHEK1
chr11	125503032	125503271	CHEK1
chr11	125505298	125505453	CHEK1
chr11	125507318	125507464	CHEK1
chr11	125513661	125513820	CHEK1
chr11	125513960	125514188	CHEK1
chr11	125514381	125514563	CHEK1
chr11	125523615	125523767	CHEK1
chr11	125525094	125525240	CHEK1
chr12	394596	394853	KDM5A
chr12	395303	395398	KDM5A

chr12	401899	402360	KDM5A
chr12	404713	404984	KDM5A
chr12	406181	406391	KDM5A
chr12	416086	416280	KDM5A
chr12	416594	417196	KDM5A
chr12	418943	419155	KDM5A
chr12	420025	420255	KDM5A
chr12	422196	422385	KDM5A
chr12	427246	427652	KDM5A
chr12	430135	430300	KDM5A
chr12	431557	431758	KDM5A
chr12	432222	432397	KDM5A
chr12	432740	432972	KDM5A
chr12	437975	438220	KDM5A
chr12	440959	441129	KDM5A
chr12	442627	442840	KDM5A
chr12	443381	443613	KDM5A
chr12	459761	459970	KDM5A
chr12	461345	461515	KDM5A
chr12	463216	463425	KDM5A
chr12	464298	464440	KDM5A
chr12	465572	465728	KDM5A
chr12	472103	472288	KDM5A
chr12	475074	475295	KDM5A
chr12	493171	493344	KDM5A
chr12	495037	495165	KDM5A
chr12	498067	498282	KDM5A
chr12	4383181	4383426	CCND2, CCND2-AS1
chr12	4385145	4385411	CCND2, CCND2-AS1
chr12	4387900	4388110	CCND2
chr12	4397982	4398181	CCND2
chr12	4409000	4409200	CCND2
chr12	6679816	6679884	CHD4
chr12	6680009	6680223	CHD4
chr12	6682214	6682460	CHD4
chr12	6686925	6687108	CHD4
chr12	6687169	6687328	CHD4
chr12	6687549	6687737	CHD4
chr12	6687986	6688108	CHD4
chr12	6690184	6690364	CHD4
chr12	6690431	6690579	CHD4
chr12	6690789	6691005	CHD4
chr12	6691277	6691472	CHD4
chr12	6691755	6691939	CHD4
chr12	6691988	6692127	CHD4
chr12	6692167	6692569	CHD4
chr12	6696524	6696750	CHD4
chr12	6696852	6697140	CHD4
chr12	6697438	6697613	CHD4
chr12	6700606	6700774	CHD4
chr12	6700834	6701016	CHD4
chr12	6701056	6701248	CHD4
chr12	6701533	6701757	CHD4
chr12	6701836	6702008	CHD4
chr12	6702231	6702419	CHD4
chr12	6702556	6702807	CHD4
chr12	6703599	6703841	CHD4
chr12	6704474	6704621	CHD4
chr12	6705146	6705328	CHD4
chr12	6707034	6707290	CHD4
chr12	6707362	6707616	CHD4
chr12	6708913	6709203	CHD4
chr12	6709357	6709586	CHD4
chr12	6709674	6709860	CHD4
chr12	6710066	6710244	CHD4
chr12	6710429	6710721	CHD4
chr12	6710788	6710957	CHD4
chr12	6711100	6711366	CHD4
chr12	6711516	6711688	CHD4
chr12	6715414	6715564	CHD4
chr12	12274034	12274379	BCL2L14, LRP6
chr12	12277473	12277621	BCL2L14, LRP6
chr12	12278204	12278391	BCL2L14, LRP6
chr12	12279599	12279880	BCL2L14, LRP6
chr12	12283691	12283852	BCL2L14, LRP6
chr12	12284729	12285016	BCL2L14, LRP6
chr12	12288083	12288259	BCL2L14, LRP6
chr12	12291233	12291493	BCL2L14, LRP6
chr12	12300274	12300515	LRP6
chr12	12301850	12302112	LRP6
chr12	12303744	12303997	LRP6
chr12	12311737	12312114	LRP6
chr12	12312688	12312923	LRP6
chr12	12315101	12315378	LRP6
chr12	12317181	12317521	LRP6
chr12	12317987	12318254	LRP6
chr12	12332718	12332940	LRP6
chr12	12333951	12334398	LRP6
chr12	12336888	12337070	LRP6
chr12	12339831	12340078	LRP6
chr12	12356111	12356359	LRP6
chr12	12397170	12397614	LRP6
chr12	12419589	12419694	LRP6
chr12	12870748	12871273	CDKN1B
chr12	12871733	12871905	CDKN1B
chr12	12873973	12874166	CDKN1B

chr12	21294483	21294617	SLCO1B1
chr12	21325558	21325750	SLCO1B1
chr12	21327485	21327668	SLCO1B1
chr12	21329684	21329856	SLCO1B1
chr12	21331484	21331681	SLCO1B1
chr12	21331830	21331979	SLCO1B1
chr12	21349854	21350147	SLCO1B1
chr12	21353416	21353631	SLCO1B1
chr12	21355399	21355645	SLCO1B1
chr12	21358776	21358992	SLCO1B1
chr12	21370027	21370262	SLCO1B1
chr12	21375208	21375323	SLCO1B1
chr12	21377630	21377798	SLCO1B1
chr12	21391887	21392148	SLCO1B1
chr12	25362703	25362870	KRAS
chr12	25368349	25368519	KRAS
chr12	25378522	25378732	KRAS
chr12	25380142	25380371	KRAS
chr12	25398182	25398343	KRAS
chr12	46123594	46123736	ARID2
chr12	46123801	46123945	ARID2
chr12	46124974	46125122	ARID2
chr12	46205175	46205359	ARID2
chr12	46211427	46211696	ARID2
chr12	46215177	46215295	ARID2
chr12	46230346	46230463	ARID2
chr12	46230498	46230799	ARID2
chr12	46231078	46231225	ARID2
chr12	46231255	46231515	ARID2
chr12	46233086	46233304	ARID2
chr12	46240613	46240745	ARID2
chr12	46242593	46242778	ARID2
chr12	46243337	46243584	ARID2
chr12	46243793	46246704	ARID2
chr12	46254558	46254757	ARID2
chr12	46285537	46285726	ARID2
chr12	46285768	46285904	ARID2
chr12	46287177	46287351	ARID2
chr12	46287387	46287529	ARID2
chr12	46298691	46298886	ARID2
chr12	48238503	48238813	VDR
chr12	48240092	48240259	VDR
chr12	48240414	48240616	VDR
chr12	48249387	48249609	VDR
chr12	48250886	48251057	VDR
chr12	48251261	48251496	VDR
chr12	48258804	48258985	VDR
chr12	48272573	48272673	VDR
chr12	48272725	48272923	VDR
chr12	48276451	48276582	VDR
chr12	48298321	48298438	VDR
chr12	49415537	49415680	KMT2D
chr12	49415800	49415959	KMT2D
chr12	49416037	49416161	KMT2D
chr12	49416347	49416683	KMT2D
chr12	49418335	49418516	KMT2D
chr12	49418567	49418754	KMT2D
chr12	49419939	49421130	KMT2D
chr12	49421560	49421738	KMT2D
chr12	49421766	49421949	KMT2D
chr12	49422585	49422766	KMT2D
chr12	49422818	49423044	KMT2D
chr12	49423158	49423284	KMT2D
chr12	49424037	49424247	KMT2D
chr12	49424358	49424576	KMT2D
chr12	49424650	49424841	KMT2D
chr12	49424932	49427772	KMT2D
chr12	49427824	49428107	KMT2D
chr12	49428167	49428284	KMT2D
chr12	49428339	49428474	KMT2D
chr12	49428569	49428743	KMT2D
chr12	49430882	49432797	KMT2D
chr12	49432979	49433166	KMT2D
chr12	49433192	49433425	KMT2D
chr12	49433481	49435343	KMT2D
chr12	49435412	49435513	KMT2D
chr12	49435674	49435798	KMT2D
chr12	49435846	49436138	KMT2D
chr12	49436318	49436453	KMT2D
chr12	49436498	49436686	KMT2D
chr12	49436833	49436994	KMT2D
chr12	49437120	49437236	KMT2D
chr12	49437392	49437590	KMT2D
chr12	49437625	49437806	KMT2D
chr12	49437957	49438112	KMT2D
chr12	49438160	49438330	KMT2D
chr12	49438501	49438773	KMT2D
chr12	49439677	49439775	KMT2D
chr12	49439822	49439982	KMT2D
chr12	49440017	49440232	KMT2D
chr12	49440366	49440598	KMT2D
chr12	49441722	49441877	KMT2D
chr12	49442416	49442577	KMT2D
chr12	49442862	49443026	KMT2D
chr12	49443439	49444598	KMT2D
chr12	49444643	49446232	KMT2D

chr12	49446321	49446517	KMT2D
chr12	49446672	49446880	KMT2D
chr12	49446964	49447129	KMT2D
chr12	49447233	49447449	KMT2D
chr12	49447735	49447948	KMT2D
chr12	49448064	49448224	KMT2D
chr12	49448285	49448559	KMT2D
chr12	49448657	49448834	KMT2D
chr12	49449033	49449132	KMT2D
chr12	52345502	52345643	ACVR1B
chr12	52369023	52369313	ACVR1B
chr12	52370085	52370384	ACVR1B
chr12	52374727	52375008	ACVR1B
chr12	52376444	52376617	ACVR1B
chr12	52377757	52377975	ACVR1B
chr12	52378950	52379157	ACVR1B
chr12	52380576	52380954	ACVR1B
chr12	52385621	52385841	ACVR1B
chr12	52387743	52387919	ACVR1B
chr12	56474059	56474191	ERBB3
chr12	56477509	56477711	ERBB3
chr12	56478753	56479121	ERBB3
chr12	56480289	56480465	ERBB3
chr12	56481335	56481451	ERBB3
chr12	56481553	56481722	ERBB3
chr12	56481779	56481971	ERBB3
chr12	56482301	56482465	ERBB3
chr12	56482506	56482677	ERBB3
chr12	56482882	56482937	ERBB3
chr12	56486505	56486629	ERBB3
chr12	56486744	56486885	ERBB3
chr12	56487103	56487359	ERBB3
chr12	56487522	56487705	ERBB3
chr12	56487857	56487998	ERBB3
chr12	56488160	56488365	ERBB3
chr12	56489015	56489119	ERBB3
chr12	56489423	56489615	ERBB3
chr12	56490261	56490431	ERBB3
chr12	56490506	56490655	ERBB3
chr12	56490803	56491039	ERBB3
chr12	56491543	56491749	ERBB3
chr12	56492258	56492384	ERBB3
chr12	56492517	56492714	ERBB3
chr12	56493406	56493554	ERBB3
chr12	56493596	56493838	ERBB3
chr12	56493932	56494054	ERBB3
chr12	56494819	56495170	ERBB3
chr12	56495287	56495864	ERBB3
chr12	58142282	58142425	CDK4, TSPAN31
chr12	58142939	58143125	CDK4, TSPAN31
chr12	58143211	58143312	CDK4, TSPAN31
chr12	58144413	58144573	CDK4
chr12	58144680	58144898	CDK4
chr12	58144964	58145150	CDK4
chr12	58145257	58145525	CDK4
chr12	69202232	69202296	MDM2
chr12	69202962	69203103	MDM2
chr12	69207308	69207433	MDM2
chr12	69210566	69210750	MDM2
chr12	69214079	69214179	MDM2
chr12	69218117	69218235	MDM2
chr12	69218309	69218456	MDM2
chr12	69222525	69222736	MDM2
chr12	69229583	69229789	MDM2
chr12	69230352	69230417	MDM2
chr12	69230426	69230554	MDM2
chr12	69233028	69233654	MDM2
chr12	112856890	112856954	PTPN11
chr12	112884054	112884227	PTPN11
chr12	112888096	112888341	PTPN11
chr12	112890973	112891216	PTPN11
chr12	112892342	112892509	PTPN11
chr12	112892572	112892628	PTPN11
chr12	112893728	112893892	PTPN11
chr12	112910722	112910869	PTPN11
chr12	112915429	112915559	PTPN11
chr12	112915635	112915844	PTPN11
chr12	112919852	112920034	PTPN11
chr12	112924253	112924462	PTPN11
chr12	112926221	112926339	PTPN11
chr12	112926802	112927004	PTPN11
chr12	112939922	112940085	PTPN11
chr12	112942473	112942593	PTPN11
chr12	121416546	121416922	HNF1A, HNF1A-AS1
chr12	121426610	121426860	HNF1A
chr12	121431297	121431534	HNF1A
chr12	121431941	121432233	HNF1A
chr12	121434039	121434241	HNF1A
chr12	121434318	121434824	HNF1A
chr12	121435251	121435621	HNF1A
chr12	121437045	121437455	HNF1A
chr12	121438842	121439020	HNF1A
chr12	121439558	121439621	HNF1A
chr12	121439853	121439960	HNF1A
chr12	121440215	121440313	C12ORF43, HNF1A
chr12	133201257	133201421	POLE

chr12	133201465	133201605	POLE
chr12	133201812	133202027	POLE
chr12	133202205	133202381	POLE
chr12	133202677	133202928	POLE
chr12	133208875	133209119	POLE
chr12	133209224	133209406	POLE
chr12	133210746	133210989	POLE
chr12	133212452	133212635	POLE
chr12	133214574	133214750	POLE
chr12	133215685	133215909	POLE
chr12	133217578	133217636	POLE
chr12	133218207	133218462	POLE
chr12	133218737	133219008	POLE
chr12	133219066	133219340	POLE
chr12	133219380	133219607	POLE
chr12	133219784	133219941	POLE
chr12	133219967	133220171	POLE
chr12	133220397	133220588	POLE
chr12	133225489	133225683	POLE
chr12	133225866	133226126	POLE
chr12	133226237	133226500	POLE
chr12	133233696	133233869	POLE
chr12	133233909	133234040	POLE
chr12	133234428	133234581	POLE
chr12	133235855	133236120	POLE
chr12	133237529	133237775	POLE
chr12	133238087	133238295	POLE
chr12	133240564	133240759	POLE
chr12	133240930	133241073	POLE
chr12	133241862	133242061	POLE
chr12	133244063	133244259	POLE
chr12	133244916	133245113	POLE
chr12	133245195	133245348	POLE
chr12	133245371	133245550	POLE
chr12	133248775	133248933	POLE
chr12	133249187	133249450	POLE
chr12	133249724	133249888	POLE
chr12	133250135	133250318	POLE
chr12	133251958	133252128	POLE
chr12	133252295	133252431	POLE
chr12	133252654	133252815	POLE
chr12	133253106	133253264	POLE
chr12	133253923	133254054	POLE
chr12	133254138	133254330	POLE
chr12	133256057	133256262	POLE
chr12	133256514	133256657	POLE
chr12	133256738	133256833	POLE
chr12	133257167	133257298	POLE
chr12	133257698	133257890	POLE
chr12	133263814	133263926	POLE
chr12	133413267	133413412	CHFR
chr13	26828753	26828931	CDK8
chr13	26911678	26911804	CDK8
chr13	26923183	26923344	CDK8
chr13	26927851	26928042	CDK8
chr13	26956925	26957033	CDK8
chr13	26959322	26959504	CDK8
chr13	26967478	26967672	CDK8
chr13	26970396	26970516	CDK8
chr13	26971264	26971387	CDK8
chr13	26974564	26974712	CDK8
chr13	26975380	26975509	CDK8
chr13	26975577	26975786	CDK8
chr13	26978067	26978243	CDK8
chr13	28578163	28578336	FLT3
chr13	28588563	28588719	FLT3
chr13	28589268	28589418	FLT3
chr13	28589701	28589863	FLT3
chr13	28592569	28592751	FLT3
chr13	28597461	28597639	FLT3
chr13	28598972	28599105	FLT3
chr13	28601199	28601403	FLT3
chr13	28602289	28602450	FLT3
chr13	28607998	28608153	FLT3
chr13	28608193	28608376	FLT3
chr13	28608412	28608569	FLT3
chr13	28609606	28609835	FLT3
chr13	28610046	28610205	FLT3
chr13	28611296	28611450	FLT3
chr13	28622386	28622605	FLT3
chr13	28623495	28623699	FLT3
chr13	28623746	28623936	FLT3
chr13	28624206	28624384	FLT3
chr13	28626656	28626836	FLT3
chr13	28631458	28631624	FLT3
chr13	28635978	28636231	FLT3
chr13	28644602	28644774	FLT3
chr13	28674579	28674672	FLT3
chr13	28877278	28877530	FLT1
chr13	28880789	28880934	FLT1
chr13	28882954	28883089	FLT1
chr13	28885701	28885894	FLT1
chr13	28886104	28886260	FLT1
chr13	28891609	28891759	FLT1
chr13	28893534	28893696	FLT1
chr13	28895574	28895747	FLT1

chr13	28896373	28896521	FLT1
chr13	28896567	28896639	FLT1
chr13	28896901	28897108	FLT1
chr13	28901573	28901712	FLT1
chr13	28903726	28903890	FLT1
chr13	28908136	28908291	FLT1
chr13	28913279	28913462	FLT1
chr13	28919556	28919713	FLT1
chr13	28931665	28931847	FLT1
chr13	28942689	28942825	FLT1
chr13	28958996	28959193	FLT1
chr13	28963812	28964266	FLT1
chr13	28970917	28971011	FLT1
chr13	28971071	28971230	FLT1
chr13	28973155	28973280	FLT1
chr13	28979891	28980056	FLT1
chr13	29001270	29001480	FLT1
chr13	29001863	29002083	FLT1
chr13	29004161	29004329	FLT1
chr13	29005247	29005472	FLT1
chr13	29007930	29008117	FLT1
chr13	29008169	29008382	FLT1
chr13	29012332	29012507	FLT1
chr13	29041014	29041291	FLT1
chr13	29041632	29041779	FLT1
chr13	29068891	29069005	FLT1
chr13	32890572	32890689	BRCA2
chr13	32893188	32893487	BRCA2
chr13	32899187	32899346	BRCA2
chr13	32900212	32900312	BRCA2
chr13	32900353	32900444	BRCA2
chr13	32900610	32900775	BRCA2
chr13	32903554	32903654	BRCA2
chr13	32905030	32905192	BRCA2
chr13	32906383	32907549	BRCA2
chr13	32910376	32915358	BRCA2
chr13	32918669	32918815	BRCA2
chr13	32920938	32921058	BRCA2
chr13	32928972	32929450	BRCA2
chr13	32930539	32930771	BRCA2
chr13	32931853	32932091	BRCA2
chr13	32936634	32936855	BRCA2
chr13	32937290	32937695	BRCA2
chr13	32944513	32944719	BRCA2
chr13	32945067	32945262	BRCA2
chr13	32950781	32950953	BRCA2
chr13	32953428	32953677	BRCA2
chr13	32953861	32954075	BRCA2
chr13	32954118	32954307	BRCA2
chr13	32968800	32969095	BRCA2
chr13	32971009	32971206	BRCA2
chr13	32972273	32972932	BRCA2
chr13	48878023	48878210	RB1
chr13	48881390	48881567	RB1
chr13	48916709	48916875	RB1
chr13	48919190	48919360	RB1
chr13	48921935	48922024	RB1
chr13	48923066	48923184	RB1
chr13	48934127	48934288	RB1
chr13	48936925	48937118	RB1
chr13	48939004	48939132	RB1
chr13	48941604	48941764	RB1
chr13	48942637	48942765	RB1
chr13	48947515	48947653	RB1
chr13	48951028	48951195	RB1
chr13	48953704	48953811	RB1
chr13	48954163	48954245	RB1
chr13	48954275	48954402	RB1
chr13	48955357	48955604	RB1
chr13	49027103	49027272	RB1
chr13	49030314	49030510	RB1
chr13	49033798	49033994	RB1
chr13	49037841	49037996	RB1
chr13	49039108	49039272	RB1
chr13	49039315	49039529	RB1
chr13	49047470	49047551	RB1
chr13	49050811	49051004	RB1
chr13	49051465	49051565	RB1
chr13	49054108	49054232	RB1
chr13	110408625	110408680	IRS2
chr13	110434363	110438425	IRS2
chr14	23776951	23777433	BCL2L2, BCL2L2-PABPN1
chr14	23777999	23778199	BCL2L2, BCL2L2-PABPN1
chr14	36986457	36987250	NKX2-1
chr14	36988164	36988600	NKX2-1, NKX2-1-AS1
chr14	36989232	36989359	NKX2-1, NKX2-1-AS1
chr14	64551609	64551795	ESR2, SYNE2
chr14	64694225	64694357	ESR2
chr14	64694582	64694672	ESR2
chr14	64694719	64694788	ESR2
chr14	64699829	64700066	ESR2
chr14	64701649	64701893	ESR2
chr14	64716238	64716422	ESR2
chr14	64723918	64724107	ESR2
chr14	64726867	64726934	ESR2
chr14	64727141	64727491	ESR2

chr14	64735487	64735654	ESR2
chr14	64746673	64746896	ESR2
chr14	64749316	64749728	ESR2
chr14	81421999	81422219	TSHR
chr14	81528466	81528588	TSHR
chr14	81534572	81534697	TSHR
chr14	81554272	81554397	TSHR
chr14	81557387	81557512	TSHR
chr14	81558849	81558977	TSHR
chr14	81562957	81563076	TSHR
chr14	81574693	81574821	TSHR
chr14	81574867	81575050	TSHR
chr14	81605997	81606236	TSHR
chr14	81609258	81610722	TSHR
chr14	102548023	102548183	HSP90AA1
chr14	102548422	102548806	HSP90AA1
chr14	102549321	102549664	HSP90AA1
chr14	102549857	102550055	HSP90AA1
chr14	102550104	102550345	HSP90AA1
chr14	102550710	102550926	HSP90AA1
chr14	102550992	102551360	HSP90AA1
chr14	102551609	102551793	HSP90AA1
chr14	102552069	102552486	HSP90AA1
chr14	102552528	102552740	HSP90AA1
chr14	102568186	102568447	HSP90AA1
chr14	102605561	102605766	HSP90AA1
chr14	105236652	105236782	AKT1
chr14	105237056	105237209	AKT1
chr14	105238676	105238814	AKT1
chr14	105239189	105239454	AKT1
chr14	105239562	105239741	AKT1
chr14	105239766	105239942	AKT1
chr14	105240223	105240342	AKT1
chr14	105241249	105241365	AKT1
chr14	105241387	105241569	AKT1
chr14	105241963	105242161	AKT1
chr14	105242970	105243132	AKT1
chr14	105246399	105246578	AKT1
chr14	105258909	105259005	AKT1
chr14	105609006	105609532	JAG2
chr14	105609793	105610000	JAG2
chr14	105611241	105611423	JAG2
chr14	105612042	105612335	JAG2
chr14	105612696	105612862	JAG2
chr14	105612922	105613086	JAG2
chr14	105613637	105613773	JAG2
chr14	105613811	105613889	JAG2
chr14	105614083	105614250	JAG2
chr14	105614427	105614591	JAG2
chr14	105614637	105614801	JAG2
chr14	105615056	105615220	JAG2
chr14	105615248	105615451	JAG2
chr14	105615481	105615682	JAG2
chr14	105616915	105617139	JAG2
chr14	105617176	105617273	JAG2
chr14	105617302	105617466	JAG2
chr14	105617594	105617758	JAG2
chr14	105617937	105618101	JAG2
chr14	105618249	105618419	JAG2
chr14	105618472	105618653	JAG2
chr14	105621873	105621984	JAG2
chr14	105622049	105622351	JAG2
chr14	105624017	105624125	JAG2
chr14	105634068	105634469	JAG2
chr14	105634666	105634782	JAG2
chr15	41221841	41221957	DLL4
chr15	41222019	41222339	DLL4
chr15	41222797	41222905	DLL4
chr15	41223675	41223989	DLL4
chr15	41224343	41224454	DLL4
chr15	41224489	41224670	DLL4
chr15	41226720	41226940	DLL4
chr15	41227070	41227340	DLL4
chr15	41228400	41229153	DLL4
chr15	41229590	41229749	DLL4
chr15	41230201	41230257	DLL4
chr15	41795757	41795823	
chr15	41796168	41796467	LTK
chr15	41796514	41796655	LTK
chr15	41796678	41796863	LTK
chr15	41796945	41797097	LTK
chr15	41797144	41797287	LTK
chr15	41797380	41797528	LTK
chr15	41797573	41797753	LTK
chr15	41797885	41798000	LTK
chr15	41798087	41798228	LTK
chr15	41799267	41799513	LTK
chr15	41799661	41799880	LTK
chr15	41800241	41800444	LTK
chr15	41801203	41801352	LTK
chr15	41803336	41803569	LTK
chr15	41803594	41803801	LTK
chr15	41803989	41804186	LTK
chr15	41804287	41804488	LTK
chr15	41804879	41805101	LTK
chr15	41805149	41805343	LTK

chr15	41805839	41805932	LTK
chr15	66679660	66679790	MAP2K1
chr15	66727339	66727600	MAP2K1
chr15	66729058	66729255	MAP2K1
chr15	66735592	66735720	MAP2K1
chr15	66736968	66737070	MAP2K1
chr15	66774067	66774242	MAP2K1
chr15	66777302	66777554	MAP2K1
chr15	66779540	66779655	MAP2K1
chr15	66781527	66781639	MAP2K1
chr15	66782030	66782126	MAP2K1
chr15	66782814	66782978	MAP2K1, SNAPC5
chr15	67358467	67358723	SMAD3
chr15	67430339	67430463	SMAD3
chr15	67457207	67457451	SMAD3
chr15	67457565	67457747	SMAD3
chr15	67459091	67459216	SMAD3
chr15	67462866	67462967	SMAD3
chr15	67473553	67473816	SMAD3
chr15	67477039	67477227	SMAD3
chr15	67479677	67479872	SMAD3
chr15	67482725	67482899	SMAD3
chr15	75042054	75042935	CYP1A2
chr15	75043504	75043675	CYP1A2
chr15	75044080	75044220	CYP1A2
chr15	75044439	75044613	CYP1A2
chr15	75045499	75045636	CYP1A2
chr15	75047106	75047454	CYP1A2
chr15	80253383	80253541	BCL2A1
chr15	80259953	80260059	BCL2A1
chr15	80263016	80263486	BCL2A1
chr15	88420140	88420376	NTRK3
chr15	88423475	88423684	NTRK3
chr15	88428899	88428991	NTRK3
chr15	88459751	88459816	NTRK3
chr15	88472396	88472690	NTRK3
chr15	88474405	88474492	NTRK3
chr15	88476217	88476440	NTRK3
chr15	88483828	88484009	NTRK3
chr15	88509649	88509706	NTRK3
chr15	88522550	88522719	NTRK3
chr15	88524427	88524616	NTRK3
chr15	88535156	88535231	NTRK3
chr15	88576062	88576301	NTRK3
chr15	88669476	88669629	NTRK3
chr15	88670367	88670482	NTRK3
chr15	88671916	88671990	NTRK3
chr15	88678306	88678653	NTRK3
chr15	88679104	88679296	NTRK3
chr15	88679672	88679865	NTRK3
chr15	88680609	88680817	NTRK3
chr15	88690540	88690659	NTRK3
chr15	88726623	88726745	NTRK3
chr15	88727430	88727555	NTRK3
chr15	88799111	88799409	NTRK3, NTRK3-AS1
chr15	90627472	90627610	IDH2
chr15	90628022	90628165	IDH2
chr15	90628207	90628355	IDH2
chr15	90628481	90628644	IDH2
chr15	90630318	90630520	IDH2
chr15	90630645	90630832	IDH2
chr15	90631565	90631759	IDH2
chr15	90631793	90632004	IDH2
chr15	90633685	90633901	IDH2
chr15	90634759	90634901	IDH2
chr15	90645482	90645647	IDH2
chr15	93444442	93444554	CHD2
chr15	93448112	93448263	CHD2
chr15	93467525	93467807	CHD2
chr15	93470448	93470585	CHD2
chr15	93472234	93472346	CHD2
chr15	93480722	93480880	CHD2
chr15	93482782	93482973	CHD2
chr15	93485026	93485210	CHD2
chr15	93486047	93486323	CHD2
chr15	93487619	93487770	CHD2
chr15	93489025	93489120	CHD2
chr15	93489242	93489471	CHD2
chr15	93492156	93492335	CHD2
chr15	93496561	93496828	CHD2
chr15	93498627	93498767	CHD2
chr15	93499663	93499904	CHD2
chr15	93510529	93510768	CHD2
chr15	93514969	93515182	CHD2
chr15	93515469	93515672	CHD2
chr15	93518083	93518205	CHD2
chr15	93521438	93521638	CHD2
chr15	93522339	93522538	CHD2
chr15	93524019	93524166	CHD2
chr15	93524569	93524712	CHD2
chr15	93527534	93527755	CHD2
chr15	93528702	93528928	CHD2
chr15	93534680	93534772	CHD2
chr15	93536063	93536253	CHD2
chr15	93540161	93540350	CHD2
chr15	93540457	93540658	CHD2

chr15	93541703	93541876	CHD2
chr15	93543716	93543895	CHD2
chr15	93545381	93545572	CHD2
chr15	93547821	93548006	CHD2
chr15	93552349	93552578	CHD2
chr15	93555549	93555699	CHD2
chr15	93557900	93558164	CHD2
chr15	93563216	93563513	CHD2
chr15	93567576	93567960	CHD2
chr15	99192785	99192929	IGF1R
chr15	99250765	99251361	IGF1R
chr15	99434528	99434891	IGF1R
chr15	99439960	99440159	IGF1R
chr15	99442680	99442875	IGF1R
chr15	99451888	99452153	IGF1R
chr15	99454518	99454695	IGF1R
chr15	99456247	99456536	IGF1R
chr15	99459167	99459385	IGF1R
chr15	99459875	99460130	IGF1R
chr15	99465351	99465685	IGF1R
chr15	99467079	99467266	IGF1R
chr15	99467728	99467938	IGF1R
chr15	99472761	99472914	IGF1R
chr15	99473438	99473559	IGF1R
chr15	99478027	99478307	IGF1R
chr15	99478519	99478680	IGF1R
chr15	99482404	99482614	IGF1R
chr15	99486126	99486306	IGF1R
chr15	99491777	99491962	IGF1R
chr15	99500264	99500696	IGF1R
chr16	338096	338273	AXIN1
chr16	339414	339632	AXIN1
chr16	341164	341322	AXIN1
chr16	343462	343743	AXIN1
chr16	347030	347251	AXIN1
chr16	347696	348276	AXIN1
chr16	354278	354466	AXIN1
chr16	359947	360094	AXIN1
chr16	364517	364708	AXIN1
chr16	396122	397050	AXIN1
chr16	2098591	2098779	TSC2
chr16	2100375	2100512	TSC2
chr16	2103317	2103478	TSC2
chr16	2104271	2104466	TSC2
chr16	2105377	2105545	TSC2
chr16	2106171	2106270	TSC2
chr16	2106619	2106795	TSC2
chr16	2107080	2107204	TSC2
chr16	2108722	2108899	TSC2
chr16	2110645	2110839	TSC2
chr16	2111846	2112034	TSC2
chr16	2112472	2112626	TSC2
chr16	2112947	2113079	TSC2
chr16	2114247	2114453	TSC2
chr16	2115494	2115661	TSC2
chr16	2120431	2120604	TSC2
chr16	2121485	2121642	TSC2
chr16	2121759	2121960	TSC2
chr16	2122216	2122389	TSC2
chr16	2122824	2123009	TSC2
chr16	2124175	2124415	TSC2
chr16	2125774	2125918	TSC2
chr16	2126043	2126196	TSC2
chr16	2126466	2126611	TSC2
chr16	2127573	2127752	TSC2
chr16	2129007	2129222	TSC2
chr16	2129251	2129454	TSC2
chr16	2129532	2129695	TSC2
chr16	2130140	2130403	TSC2
chr16	2131570	2131824	TSC2
chr16	2132411	2132530	TSC2
chr16	2133670	2133842	TSC2
chr16	2134203	2134741	TSC2
chr16	2134926	2135052	TSC2
chr16	2135205	2135348	TSC2
chr16	2136168	2136405	TSC2
chr16	2136707	2136897	TSC2
chr16	2137838	2137967	TSC2
chr16	2138023	2138165	TSC2
chr16	2138202	2138351	TSC2
chr16	2138421	2138636	TSC2
chr16	3777693	3779900	CREBBP
chr16	3781167	3781499	CREBBP
chr16	3781751	3781963	CREBBP
chr16	3786011	3786229	CREBBP
chr16	3786625	3786841	CREBBP
chr16	3788534	3788698	CREBBP
chr16	3789553	3789750	CREBBP
chr16	3790374	3790575	CREBBP
chr16	3794869	3794987	CREBBP
chr16	3795252	3795380	CREBBP
chr16	3799602	3799709	CREBBP
chr16	3801701	3801832	CREBBP
chr16	3807263	3807402	CREBBP
chr16	3807784	3808074	CREBBP
chr16	3808829	3808998	CREBBP

chr16	3817695	3817935	CREBBP
chr16	3819149	3819379	CREBBP
chr16	3820545	3821012	CREBBP
chr16	3823726	3823956	CREBBP
chr16	3824544	3824719	CREBBP
chr16	3827588	3827683	CREBBP
chr16	3827986	3828208	CREBBP
chr16	3828675	3828843	CREBBP
chr16	3830707	3830904	CREBBP
chr16	3831179	3831332	CREBBP
chr16	3832659	3832952	CREBBP
chr16	3841956	3842120	CREBBP
chr16	3843361	3843652	CREBBP
chr16	3860578	3860805	CREBBP
chr16	3900272	3901035	CREBBP
chr16	3929807	3930146	CREBBP
chr16	11348674	11349360	RMI2, SOCS1
chr16	28617116	28617279	SULT1A1
chr16	28617351	28617582	SULT1A1
chr16	28618056	28618201	SULT1A1
chr16	28618246	28618423	SULT1A1
chr16	28619586	28619734	SULT1A1
chr16	28619773	28619949	SULT1A1
chr16	28620003	28620201	SULT1A1
chr16	28631358	28631479	SULT1A1
chr16	28634426	28634543	SULT1A1
chr16	31102429	31102688	VKORC1
chr16	31104078	31104210	VKORC1
chr16	31104607	31104828	VKORC1
chr16	31105530	31105847	VKORC1
chr16	31105852	31106075	VKORC1
chr16	31106684	31107004	VKORC1
chr16	50783584	50784138	CYLD
chr16	50785489	50785842	CYLD
chr16	50788204	50788401	CYLD
chr16	50809051	50809110	CYLD
chr16	50810055	50810213	CYLD
chr16	50811710	50811877	CYLD
chr16	50813550	50813980	CYLD
chr16	50815131	50815347	CYLD
chr16	50816210	50816402	CYLD
chr16	50818214	50818387	CYLD
chr16	50820740	50820882	CYLD
chr16	50821671	50821788	CYLD
chr16	50825443	50825626	CYLD
chr16	50826482	50826641	CYLD
chr16	50827431	50827600	CYLD
chr16	50828097	50828364	CYLD
chr16	50830209	50830444	CYLD
chr16	66413215	66413475	CDH5
chr16	66420686	66421025	CDH5
chr16	66422201	66422368	CDH5
chr16	66423235	66423450	CDH5
chr16	66424280	66424518	CDH5
chr16	66426013	66426311	CDH5
chr16	66429936	66430129	CDH5
chr16	66431859	66432034	CDH5
chr16	66432333	66432489	CDH5
chr16	66434648	66434944	CDH5
chr16	66436529	66437097	CDH5
chr16	67063285	67063413	CBFB
chr16	67063604	67063741	CBFB
chr16	67070516	67070683	CBFB
chr16	67100559	67100726	CBFB
chr16	67116090	67116267	CBFB
chr16	67132587	67132706	CBFB
chr16	67644710	67645541	CTCF
chr16	67645828	67646049	CTCF
chr16	67650622	67650806	CTCF
chr16	67654574	67654745	CTCF
chr16	67655319	67655519	CTCF
chr16	67660432	67660643	CTCF
chr16	67662247	67662480	CTCF
chr16	67663275	67663461	CTCF
chr16	67670567	67670779	CTCF
chr16	67671565	67671800	CTCF
chr16	68771293	68771391	CDH1
chr16	68772174	68772339	CDH1
chr16	68835547	68835821	CDH1
chr16	68842301	68842495	CDH1
chr16	68842570	68842776	CDH1
chr16	68844074	68844269	CDH1
chr16	68845561	68845787	CDH1
chr16	68846012	68846191	CDH1
chr16	68847190	68847490	CDH1
chr16	68849392	68849687	CDH1
chr16	68853157	68853353	CDH1
chr16	68855878	68856153	CDH1
chr16	68857276	68857554	CDH1
chr16	68862051	68862232	CDH1
chr16	68863531	68863725	CDH1
chr16	68867167	68867427	CDH1
chr16	68868080	68868209	CDH1
chr16	69744853	69745209	NQO1
chr16	69746905	69747057	NQO1
chr16	69748841	69749005	NQO1

chr16	69752000	69752181	NQO1
chr16	69752247	69752462	NQO1
chr16	69760310	69760367	NQO1
chr16	71674777	71674968	MARVELD3, PHLPP2
chr16	71678679	71678783	PHLPP2
chr16	71682767	71683972	PHLPP2
chr16	71686667	71686949	PHLPP2
chr16	71689117	71689362	PHLPP2
chr16	71690448	71690609	PHLPP2
chr16	71692110	71692291	PHLPP2
chr16	71692530	71692743	PHLPP2
chr16	71697776	71698027	PHLPP2
chr16	71701055	71701261	PHLPP2
chr16	71703152	71703298	PHLPP2
chr16	71706139	71706250	PHLPP2
chr16	71710324	71710577	PHLPP2
chr16	71712628	71712913	PHLPP2
chr16	71713266	71713463	PHLPP2
chr16	71715628	71715833	PHLPP2
chr16	71718353	71718529	PHLPP2
chr16	71724396	71724637	PHLPP2
chr16	71736475	71736659	PHLPP2
chr16	71748389	71748723	PHLPP2
chr16	89804983	89805141	FANCA, ZNF276
chr16	89805264	89805407	FANCA, ZNF276
chr16	89805515	89805722	FANCA, ZNF276
chr16	89805860	89805986	FANCA, ZNF276
chr16	89806272	89806532	FANCA, ZNF276
chr16	89807186	89807299	FANCA, ZNF276
chr16	89809182	89809371	FANCA
chr16	89811341	89811504	FANCA
chr16	89812966	89813121	FANCA
chr16	89813213	89813323	FANCA
chr16	89815041	89815200	FANCA
chr16	89816112	89816335	FANCA
chr16	89818520	89818655	FANCA
chr16	89824959	89825138	FANCA
chr16	89828331	89828455	FANCA
chr16	89831272	89831499	FANCA
chr16	89833523	89833670	FANCA
chr16	89836219	89836457	FANCA
chr16	89836548	89836692	FANCA
chr16	89836946	89837067	FANCA
chr16	89838060	89838247	FANCA
chr16	89839653	89839817	FANCA
chr16	89842124	89842248	FANCA
chr16	89845183	89845283	FANCA
chr16	89845325	89845436	FANCA
chr16	89846251	89846390	FANCA
chr16	89849241	89849351	FANCA
chr16	89849389	89849535	FANCA
chr16	89851236	89851397	FANCA
chr16	89857785	89857969	FANCA
chr16	89858309	89858501	FANCA
chr16	89858853	89858980	FANCA
chr16	89862288	89862451	FANCA
chr16	89865440	89865512	FANCA
chr16	89865548	89865665	FANCA
chr16	89865987	89866071	FANCA
chr16	89869641	89869774	FANCA
chr16	89871662	89871825	FANCA
chr16	89874676	89874800	FANCA
chr16	89877089	89877235	FANCA
chr16	89877311	89877504	FANCA
chr16	89880902	89881046	FANCA
chr16	89882259	89882419	FANCA
chr16	89882919	89883048	FANCA
chr17	7565231	7565357	TP53
chr17	7569498	7569587	TP53
chr17	7572901	7573033	TP53
chr17	7573901	7574058	TP53
chr17	7576511	7576682	TP53
chr17	7576827	7576951	TP53
chr17	7576993	7577180	TP53
chr17	7577473	7577633	TP53
chr17	7578112	7578314	TP53
chr17	7578345	7578579	TP53
chr17	7579286	7579615	TP53
chr17	7579674	7579746	TP53
chr17	7579813	7579937	TP53
chr17	8108163	8108387	AURKB
chr17	8108508	8108733	AURKB
chr17	8109783	8109982	AURKB
chr17	8110042	8110231	AURKB
chr17	8110468	8110713	AURKB
chr17	8110863	8110968	AURKB
chr17	8111030	8111183	AURKB
chr17	8113469	8113567	AURKB
chr17	11924178	11924343	MAP2K4, RPL21P122
chr17	11935557	11935640	MAP2K4
chr17	11958180	11958333	MAP2K4
chr17	11984647	11984872	MAP2K4
chr17	11998744	11998803	MAP2K4
chr17	11998866	11999036	MAP2K4
chr17	12011081	12011251	MAP2K4
chr17	12013666	12013768	MAP2K4

chr17	12016524	12016702	MAP2K4
chr17	12028585	12028713	MAP2K4
chr17	12032430	12032629	MAP2K4
chr17	12043130	12043226	MAP2K4
chr17	12044438	12044602	MAP2K4
chr17	17116943	17117195	FLCN
chr17	17118273	17118429	FLCN
chr17	17118473	17118655	FLCN
chr17	17119668	17119842	FLCN
chr17	17120357	17120521	FLCN
chr17	17122307	17122548	FLCN
chr17	17124667	17124967	FLCN
chr17	17125789	17126000	FLCN
chr17	17127210	17127482	FLCN
chr17	17129266	17129661	FLCN
chr17	17131177	17131476	FLCN
chr17	29422302	29422412	NF1
chr17	29482975	29483169	NF1
chr17	29486002	29486136	NF1
chr17	29490178	29490419	NF1
chr17	29496883	29497040	NF1
chr17	29508414	29508532	NF1
chr17	29508702	29508828	NF1
chr17	29509500	29509708	NF1
chr17	29527414	29527638	NF1
chr17	29528029	29528202	NF1
chr17	29528403	29528528	NF1
chr17	29533232	29533414	NF1
chr17	29541443	29541628	NF1
chr17	29545997	29546161	NF1
chr17	29548842	29549033	NF1
chr17	29550436	29550610	NF1
chr17	29552087	29552293	NF1
chr17	29553427	29553727	NF1
chr17	29554210	29554334	NF1
chr17	29554515	29554649	NF1
chr17	29556017	29556508	NF1
chr17	29556827	29557017	NF1
chr17	29557252	29557425	NF1
chr17	29557834	29557968	NF1
chr17	29559065	29559232	NF1
chr17	29559692	29559924	NF1
chr17	29559994	29560256	NF1
chr17	29562603	29562815	NF1
chr17	29562910	29563064	NF1
chr17	29575976	29576162	NF1
chr17	29579930	29580043	NF1
chr17	29585336	29585545	NF1
chr17	29586024	29586172	NF1
chr17	29587361	29587558	NF1
chr17	29588703	29588900	NF1
chr17	29592221	29592382	NF1
chr17	29652812	29653295	NF1
chr17	29654491	29654882	NF1
chr17	29657288	29657541	NF1
chr17	29661830	29662074	NF1
chr17	29663325	29663516	NF1
chr17	29663627	29663957	NF1
chr17	29664360	29664625	NF1
chr17	29664811	29664923	NF1
chr17	29664994	29665182	NF1
chr17	29665696	29665848	NF1
chr17	29667497	29667688	NF1
chr17	29670001	29670178	NF1
chr17	29676112	29676294	NF1
chr17	29677175	29677361	NF1
chr17	29679249	29679457	NF1
chr17	29683452	29683625	NF1
chr17	29683952	29684133	NF1
chr17	29684261	29684412	NF1
chr17	29685472	29685665	NF1
chr17	29685961	29686058	NF1
chr17	29687479	29687746	NF1
chr17	29701005	29701198	NF1
chr17	29705880	29705974	NF1
chr17	37618299	37619395	CDK12
chr17	37627106	37628041	CDK12
chr17	37646784	37647011	CDK12
chr17	37648978	37649168	CDK12
chr17	37650751	37650972	CDK12
chr17	37657477	37657717	CDK12
chr17	37665932	37666039	CDK12
chr17	37667756	37667908	CDK12
chr17	37671958	37672086	CDK12
chr17	37673667	37673834	CDK12
chr17	37676183	37676365	CDK12
chr17	37680901	37681163	CDK12
chr17	37682091	37682594	CDK12
chr17	37686831	37687594	CDK12
chr17	37855787	37855865	ERBB2
chr17	37856466	37856589	ERBB2
chr17	37863217	37863419	ERBB2
chr17	37864548	37864812	ERBB2
chr17	37865545	37865730	ERBB2
chr17	37866040	37866159	ERBB2
chr17	37866313	37866479	ERBB2

chr17	37866567	37866759	ERBB2
chr17	37868155	37868325	ERBB2
chr17	37868549	37868726	ERBB2
chr17	37871513	37871637	ERBB2
chr17	37871673	37871814	ERBB2
chr17	37871967	37872217	ERBB2
chr17	37872528	37872711	ERBB2
chr17	37872742	37872883	ERBB2
chr17	37873547	37873762	ERBB2
chr17	37876014	37876112	ERBB2
chr17	37879546	37879735	ERBB2
chr17	37879765	37879938	ERBB2
chr17	37880139	37880288	ERBB2
chr17	37880953	37881189	ERBB2
chr17	37881276	37881482	ERBB2
chr17	37881554	37881680	ERBB2
chr17	37881934	37882131	ERBB2
chr17	37882789	37882937	ERBB2, MIR4728
chr17	37883042	37883281	ERBB2
chr17	37883522	37883825	ERBB2
chr17	37883916	37884322	ERBB2
chr17	38487445	38487673	RARA
chr17	38497616	38497892	RARA
chr17	38498931	38499144	RARA
chr17	38504542	38504741	RARA
chr17	38506010	38506202	RARA
chr17	38508136	38508347	RARA
chr17	38508557	38508784	RARA
chr17	38510528	38510783	RARA
chr17	38511489	38511698	RARA
chr17	38512235	38513041	RARA
chr17	38545745	38545924	TOP2A
chr17	38546191	38546441	TOP2A
chr17	38547732	38547917	TOP2A
chr17	38548294	38548422	TOP2A
chr17	38548440	38548618	TOP2A
chr17	38548811	38549014	TOP2A
chr17	38551675	38551816	TOP2A
chr17	38552509	38552742	TOP2A
chr17	38554784	38554918	TOP2A
chr17	38554999	38555214	TOP2A
chr17	38555266	38555408	TOP2A
chr17	38556098	38556344	TOP2A
chr17	38556435	38556686	TOP2A
chr17	38556755	38556940	TOP2A
chr17	38557076	38557358	TOP2A
chr17	38559132	38559331	TOP2A
chr17	38560378	38560550	TOP2A
chr17	38560603	38560768	TOP2A
chr17	38561017	38561160	TOP2A
chr17	38562610	38562770	TOP2A
chr17	38562810	38562966	TOP2A
chr17	38563058	38563219	TOP2A
chr17	38563775	38563951	TOP2A
chr17	38564193	38564401	TOP2A
chr17	38564718	38565012	TOP2A
chr17	38565087	38565175	TOP2A
chr17	38565379	38565478	TOP2A
chr17	38565612	38565692	TOP2A
chr17	38565701	38565910	TOP2A
chr17	38566189	38566339	TOP2A
chr17	38567335	38567523	TOP2A
chr17	38567580	38567761	TOP2A
chr17	38567771	38568095	TOP2A
chr17	38568985	38569248	TOP2A
chr17	38569378	38569569	TOP2A
chr17	38569706	38569902	TOP2A
chr17	38572231	38572345	TOP2A
chr17	38572648	38572789	TOP2A
chr17	38572966	38573172	TOP2A
chr17	38573997	38574068	TOP2A
chr17	38574365	38574433	
chr17	40467737	40467843	STAT3
chr17	40468781	40468944	STAT3
chr17	40469174	40469267	STAT3
chr17	40474274	40474537	STAT3
chr17	40474996	40475186	STAT3
chr17	40475252	40475397	STAT3
chr17	40475565	40475668	STAT3
chr17	40476703	40476889	STAT3
chr17	40476955	40477104	STAT3
chr17	40478108	40478242	STAT3
chr17	40481402	40481500	STAT3
chr17	40481546	40481690	STAT3
chr17	40481739	40481819	STAT3
chr17	40483464	40483574	STAT3
chr17	40485665	40485808	STAT3
chr17	40485883	40486092	STAT3
chr17	40489427	40489629	STAT3
chr17	40489755	40489900	STAT3
chr17	40490723	40490855	STAT3
chr17	40491306	40491452	STAT3
chr17	40497551	40497700	STAT3
chr17	40498561	40498756	STAT3
chr17	40500381	40500559	STAT3
chr17	41197669	41197844	BRCA1

chr17	41199634	41199745	BRCA1
chr17	41201112	41201236	BRCA1
chr17	41203054	41203159	BRCA1
chr17	41209043	41209177	BRCA1
chr17	41215324	41215415	BRCA1
chr17	41215865	41215993	BRCA1
chr17	41219599	41219737	BRCA1
chr17	41222919	41223280	BRCA1
chr17	41226322	41226563	BRCA1
chr17	41228479	41228656	BRCA1
chr17	41231325	41231441	BRCA1, RPL21P4
chr17	41234395	41234617	BRCA1
chr17	41242935	41243074	BRCA1
chr17	41243426	41246902	BRCA1
chr17	41247837	41247964	BRCA1
chr17	41249235	41249331	BRCA1
chr17	41251766	41251922	BRCA1
chr17	41256113	41256303	BRCA1
chr17	41256859	41256998	BRCA1
chr17	41258447	41258575	BRCA1
chr17	41267717	41267821	BRCA1
chr17	41276008	41276138	BRCA1
chr17	47677714	47677909	SPOP
chr17	47679201	47679394	SPOP
chr17	47684586	47684759	SPOP
chr17	47685210	47685316	SPOP
chr17	47688616	47688844	SPOP
chr17	47696317	47696495	SPOP
chr17	47696570	47696772	SPOP
chr17	47699282	47699454	SPOP
chr17	47700069	47700197	SPOP
chr17	59760631	59761526	BRIP1
chr17	59763171	59763551	BRIP1
chr17	59770765	59770898	BRIP1
chr17	59793286	59793449	BRIP1
chr17	59820348	59820520	BRIP1
chr17	59821767	59821977	BRIP1
chr17	59853736	59853948	BRIP1
chr17	59857596	59857787	BRIP1
chr17	59858175	59858391	BRIP1
chr17	59861605	59861810	BRIP1
chr17	59870932	59871115	BRIP1
chr17	59876435	59876685	BRIP1
chr17	59878588	59878860	BRIP1
chr17	59885802	59886143	BRIP1
chr17	59924436	59924606	BRIP1
chr17	59926464	59926642	BRIP1
chr17	59934393	59934617	BRIP1
chr17	59937131	59937293	BRIP1
chr17	59938782	59938925	BRIP1
chr17	61554430	61554729	ACE
chr17	61555266	61555484	ACE
chr17	61556342	61556486	ACE
chr17	61557104	61557298	ACE
chr17	61557672	61557914	ACE
chr17	61558426	61558574	ACE
chr17	61558901	61559124	ACE
chr17	61559801	61560075	ACE
chr17	61560364	61560559	ACE
chr17	61560795	61560944	ACE
chr17	61561184	61561357	ACE
chr17	61561665	61561927	ACE
chr17	61562203	61562452	ACE
chr17	61562571	61562758	ACE
chr17	61563892	61564101	ACE
chr17	61564321	61564459	ACE
chr17	61565983	61566177	ACE
chr17	61566276	61566518	ACE
chr17	61568289	61568437	ACE
chr17	61568544	61568767	ACE
chr17	61570771	61571045	ACE
chr17	61571257	61571452	ACE
chr17	61571707	61571856	ACE
chr17	61573729	61573902	ACE
chr17	61574133	61574371	ACE
chr17	61574472	61574752	ACE
chr17	61574868	61574946	ACE
chr17	62006560	62006709	CD79B
chr17	62006768	62006860	CD79B
chr17	62007104	62007273	CD79B
chr17	62007408	62007770	CD79B
chr17	62008669	62008773	CD79B
chr17	62009529	62009646	CD79B
chr17	70117507	70117988	SOX9
chr17	70118834	70119138	SOX9
chr17	70119658	70120553	SOX9
chr17	74731914	74731982	MFSD11, SRSF2
chr17	74732210	74732571	MFSD11, MIR636, SRSF2
chr17	74732855	74733348	MFSD11, SRSF2
chr17	78519404	78519616	RPTOR
chr17	78599465	78599618	RPTOR
chr17	78617502	78617635	RPTOR
chr17	78681615	78681824	RPTOR
chr17	78704334	78704531	RPTOR
chr17	78727784	78728010	RPTOR
chr17	78765224	78765334	RPTOR

chr17	78795975	78796126	RPTOR
chr17	78796853	78797048	RPTOR
chr17	78811696	78811822	RPTOR
chr17	78820247	78820399	RPTOR
chr17	78829238	78829372	RPTOR
chr17	78831564	78831761	RPTOR
chr17	78854189	78854314	RPTOR
chr17	78857193	78857309	RPTOR
chr17	78857555	78857797	RPTOR
chr17	78858782	78858973	RPTOR
chr17	78865494	78865662	RPTOR
chr17	78866503	78866694	RPTOR
chr17	78867481	78867690	RPTOR
chr17	78882585	78882754	RPTOR
chr17	78896498	78896652	RPTOR
chr17	78897264	78897498	RPTOR
chr17	78899144	78899305	RPTOR
chr17	78914270	78914426	RPTOR
chr17	78919441	78919606	RPTOR
chr17	78921001	78921176	RPTOR
chr17	78923217	78923372	RPTOR
chr17	78931398	78931555	RPTOR
chr17	78933852	78934030	RPTOR
chr17	78935168	78935305	RPTOR
chr17	78936235	78936402	RPTOR
chr17	78936702	78936882	RPTOR
chr17	78938036	78938155	RPTOR
chr18	657717	657972	TYMS, TYMSOS
chr18	659615	659739	TYMS
chr18	662120	662345	TYMS
chr18	669046	669198	TYMS
chr18	670666	670892	TYMS
chr18	671354	671476	TYMS
chr18	672834	673022	ENOSF1, TYMS
chr18	19751080	19752265	GATA6
chr18	19756890	19757107	GATA6
chr18	19761388	19761564	GATA6
chr18	19762692	19762830	GATA6
chr18	19762875	19763029	GATA6
chr18	19780593	19780811	GATA6
chr18	25532091	25532348	CDH2
chr18	25543295	25543510	CDH2
chr18	25562882	25563072	CDH2
chr18	25564938	25565222	CDH2
chr18	25565466	25565750	CDH2
chr18	25568462	25568655	CDH2
chr18	25570035	25570339	CDH2
chr18	25572593	25572829	CDH2
chr18	25573438	25573626	CDH2
chr18	25582935	25583158	CDH2
chr18	25585787	25585982	CDH2
chr18	25589655	25589861	CDH2
chr18	25591784	25591981	CDH2
chr18	25593621	25593898	CDH2
chr18	25616408	25616537	CDH2
chr18	25727611	25727773	CDH2
chr18	25756901	25757011	CDH2
chr18	45368172	45368346	SMAD2
chr18	45371685	45371880	SMAD2
chr18	45372008	45372196	SMAD2
chr18	45374820	45375083	SMAD2
chr18	45377619	45377723	SMAD2
chr18	45391404	45391529	SMAD2
chr18	45394668	45394853	SMAD2
chr18	45395588	45395832	SMAD2
chr18	45396820	45396960	SMAD2
chr18	45422866	45423152	SMAD2
chr18	48573391	48573690	SMAD4
chr18	48575030	48575255	SMAD4
chr18	48575639	48575719	SMAD4
chr18	48577698	48577810	SMAD4
chr18	48578979	48579047	SMAD4
chr18	48581125	48581388	SMAD4
chr18	48584469	48584639	SMAD4
chr18	48584684	48584851	SMAD4
chr18	48586210	48586311	SMAD4
chr18	48591767	48592001	SMAD4
chr18	48593363	48593582	SMAD4
chr18	48602982	48603171	SMAD4
chr18	48604600	48604862	SMAD4
chr18	59157761	59158057	CDH20
chr18	59166393	59166738	CDH20
chr18	59167590	59167760	CDH20
chr18	59170160	59170378	CDH20
chr18	59174580	59174818	CDH20
chr18	59195174	59195478	CDH20
chr18	59203700	59203887	CDH20
chr18	59206231	59206403	CDH20
chr18	59212234	59212402	CDH20
chr18	59217185	59217487	CDH20
chr18	59221397	59221953	CDH20
chr18	60795832	60796017	BCL2
chr18	60985256	60985924	BCL2
chr19	1206887	1207227	STK11
chr19	1218390	1218524	STK11
chr19	1219297	1219437	STK11

chr19	1220346	1220529	STK11
chr19	1220554	1220741	STK11
chr19	1221186	1221364	STK11
chr19	1221922	1222030	STK11
chr19	1222958	1223196	STK11
chr19	1226427	1226671	STK11
chr19	2164158	2164289	DOT1L
chr19	2180686	2180780	DOT1L
chr19	2185828	2185953	DOT1L
chr19	2189705	2189819	DOT1L
chr19	2190985	2191264	DOT1L
chr19	2193662	2193807	DOT1L
chr19	2194488	2194601	DOT1L
chr19	2199857	2199963	DOT1L
chr19	2202673	2202803	DOT1L
chr19	2206702	2206821	DOT1L
chr19	2207547	2207704	DOT1L
chr19	2208908	2209000	DOT1L
chr19	2210373	2210534	DOT1L
chr19	2210594	2210879	DOT1L
chr19	2211072	2211236	DOT1L
chr19	2211724	2211866	DOT1L
chr19	2213512	2213664	DOT1L
chr19	2213822	2214010	DOT1L
chr19	2214444	2214620	DOT1L
chr19	2216254	2216789	DOT1L
chr19	2216928	2217114	DOT1L
chr19	2217745	2217942	DOT1L
chr19	2220081	2220246	DOT1L
chr19	2221949	2222583	DOT1L
chr19	2223254	2223510	DOT1L
chr19	2225361	2225476	DOT1L
chr19	2226156	2227151	DOT1L
chr19	2227190	2227246	DOT1L
chr19	2227710	2228371	DOT1L
chr19	2229758	2229816	DOT1L
chr19	3094624	3094810	GNA11
chr19	3110121	3110356	GNA11
chr19	3113302	3113507	GNA11
chr19	3114916	3115095	GNA11
chr19	3118896	3119076	GNA11
chr19	3119178	3119382	GNA11
chr19	3120961	3121202	GNA11
chr19	4090570	4090731	MAP2K2
chr19	4094425	4094521	MAP2K2
chr19	4095360	4095472	MAP2K2
chr19	4097251	4097366	MAP2K2
chr19	4099173	4099437	MAP2K2
chr19	4100991	4101166	MAP2K2
chr19	4101201	4101303	MAP2K2
chr19	4102348	4102476	MAP2K2
chr19	4110481	4110678	MAP2K2
chr19	4117391	4117652	MAP2K2
chr19	4123755	4123897	MAP2K2
chr19	7117041	7117446	INSR
chr19	7119434	7119619	INSR
chr19	7120605	7120785	INSR
chr19	7122599	7122809	INSR
chr19	7122864	7123025	INSR
chr19	7125268	7125563	INSR
chr19	7126569	7126687	INSR
chr19	7128837	7128990	INSR
chr19	7132143	7132353	INSR
chr19	7141662	7141852	INSR
chr19	7142801	7143126	INSR
chr19	7150482	7150568	INSR
chr19	7152711	7152963	INSR
chr19	7163017	7163235	INSR
chr19	7166139	7166440	INSR
chr19	7167953	7168130	INSR
chr19	7170522	7170787	INSR
chr19	7172275	7172470	INSR
chr19	7174568	7174767	INSR
chr19	7184301	7184673	INSR
chr19	7267330	7267932	INSR
chr19	7293777	7293927	INSR
chr19	8555047	8555135	PRAM1
chr19	8555196	8555281	PRAM1
chr19	8555334	8555418	PRAM1
chr19	8555452	8555665	PRAM1
chr19	8555745	8555904	PRAM1
chr19	8555958	8556066	PRAM1
chr19	8560913	8561014	PRAM1
chr19	8562622	8562749	PRAM1
chr19	8563081	8563198	PRAM1
chr19	8563234	8564689	PRAM1
chr19	8567423	8567500	PRAM1
chr19	10597302	10597519	KEAP1
chr19	10599842	10600069	KEAP1
chr19	10600298	10600554	KEAP1
chr19	10602227	10602963	KEAP1
chr19	10610045	10610734	KEAP1
chr19	11094802	11095074	SMARCA4
chr19	11095923	11096106	SMARCA4
chr19	11096839	11097294	SMARCA4
chr19	11097555	11097704	SMARCA4

chr19	11098316	11098625	SMARCA4
chr19	11099967	11100144	SMARCA4
chr19	11101800	11102024	SMARCA4
chr19	11105478	11105702	SMARCA4
chr19	11105767	11105914	SMARCA4
chr19	11105993	11106086	SMARCA4
chr19	11106154	11106244	SMARCA4
chr19	11106565	11106627	SMARCA4
chr19	11106863	11107081	SMARCA4
chr19	11107144	11107245	SMARCA4
chr19	11113679	11113860	SMARCA4
chr19	11113990	11114098	SMARCA4
chr19	11118552	11118724	SMARCA4
chr19	11121031	11121232	SMARCA4
chr19	11123599	11123813	SMARCA4
chr19	11129607	11129724	SMARCA4
chr19	11130241	11130402	SMARCA4
chr19	11132375	11132668	SMARCA4
chr19	11134168	11134332	SMARCA4
chr19	11134981	11135139	SMARCA4
chr19	11136072	11136209	SMARCA4
chr19	11136950	11137047	SMARCA4
chr19	11138434	11138651	SMARCA4
chr19	11141380	11141594	SMARCA4
chr19	11143940	11144218	SMARCA4
chr19	11144417	11144566	SMARCA4
chr19	11144773	11144901	SMARCA4
chr19	11145564	11145833	SMARCA4
chr19	11150108	11150254	SMARCA4
chr19	11151948	11152261	SMARCA4
chr19	11168905	11169064	SMARCA4
chr19	11169438	11169590	SMARCA4
chr19	11170403	11170586	SMARCA4
chr19	11170695	11170888	SMARCA4
chr19	11172434	11172517	SMARCA4
chr19	15271447	15272550	NOTCH3
chr19	15273250	15273398	NOTCH3
chr19	15276153	15276351	NOTCH3
chr19	15276572	15276927	NOTCH3
chr19	15278034	15278247	NOTCH3
chr19	15280871	15281006	NOTCH3
chr19	15281116	15281389	NOTCH3
chr19	15281456	15281661	NOTCH3
chr19	15284853	15285236	NOTCH3
chr19	15288310	15288926	NOTCH3
chr19	15289608	15289777	NOTCH3
chr19	15289810	15290118	NOTCH3
chr19	15290149	15290332	NOTCH3
chr19	15290857	15291092	NOTCH3
chr19	15291466	15291664	NOTCH3
chr19	15291746	15291998	NOTCH3
chr19	15292361	15292637	NOTCH3
chr19	15295080	15295286	NOTCH3
chr19	15295691	15295855	NOTCH3
chr19	15296042	15296244	NOTCH3
chr19	15296272	15296515	NOTCH3
chr19	15297663	15297824	NOTCH3
chr19	15297890	15298174	NOTCH3
chr19	15298666	15298830	NOTCH3
chr19	15299020	15299184	NOTCH3
chr19	15299774	15300010	NOTCH3
chr19	15300058	15300264	NOTCH3
chr19	15302209	15302493	NOTCH3
chr19	15302530	15302703	NOTCH3
chr19	15302745	15303134	NOTCH3
chr19	15303162	15303355	NOTCH3
chr19	15308285	15308414	NOTCH3
chr19	15311573	15311747	NOTCH3
chr19	15349162	15349281	BRD4
chr19	15349528	15349816	BRD4
chr19	15349844	15350100	BRD4
chr19	15350177	15350358	BRD4
chr19	15350444	15350657	BRD4
chr19	15350695	15350858	BRD4
chr19	15353685	15354323	BRD4
chr19	15355016	15355436	BRD4
chr19	15355495	15355598	BRD4
chr19	15360065	15360126	BRD4
chr19	15364342	15364619	BRD4
chr19	15364937	15365098	BRD4
chr19	15366082	15366428	BRD4
chr19	15366849	15367099	BRD4
chr19	15367749	15368009	BRD4
chr19	15374205	15374384	BRD4
chr19	15375189	15375602	BRD4
chr19	15376139	15376479	BRD4
chr19	15378201	15378387	BRD4
chr19	15379690	15379878	BRD4
chr19	15383600	15383935	BRD4
chr19	17937526	17937744	JAK3
chr19	17940813	17941052	JAK3
chr19	17941286	17941454	JAK3
chr19	17942011	17942234	JAK3
chr19	17942457	17942632	JAK3
chr19	17943302	17943542	JAK3
chr19	17943573	17943763	JAK3

chr19	17945354	17945555	JAK3
chr19	17945635	17945837	JAK3
chr19	17945866	17946049	JAK3
chr19	17946707	17946885	JAK3
chr19	17947838	17948047	JAK3
chr19	17948715	17948897	JAK3
chr19	17949046	17949224	JAK3
chr19	17950260	17950497	JAK3
chr19	17951013	17951175	JAK3
chr19	17952172	17952380	JAK3
chr19	17952423	17952596	JAK3
chr19	17953099	17953444	JAK3
chr19	17953810	17954006	JAK3
chr19	17954163	17954325	JAK3
chr19	17954560	17954734	JAK3
chr19	17955017	17955251	JAK3
chr19	30303437	30303510	CCNE1
chr19	30303570	30303708	CCNE1
chr19	30303850	30303969	CCNE1
chr19	30308018	30308214	CCNE1
chr19	30308287	30308491	CCNE1
chr19	30311583	30311780	CCNE1
chr19	30312603	30312749	CCNE1
chr19	30312877	30313062	CCNE1
chr19	30313121	30313283	CCNE1
chr19	30313327	30313535	CCNE1
chr19	30314536	30314709	CCNE1
chr19	33792218	33793345	CEBPA
chr19	39989589	39989708	DLL3
chr19	39989806	39990138	DLL3
chr19	39991229	39991337	DLL3
chr19	39993429	39993722	DLL3
chr19	39994685	39994953	DLL3
chr19	39995843	39996116	DLL3
chr19	39997653	39998283	DLL3
chr19	39998444	39998678	DLL3
chr19	39998866	39998922	DLL3
chr19	40739450	40739621	AKT2
chr19	40739753	40739883	AKT2
chr19	40740926	40741079	AKT2
chr19	40741144	40741282	AKT2
chr19	40741771	40742036	AKT2
chr19	40742138	40742317	AKT2
chr19	40743850	40744023	AKT2
chr19	40744786	40744905	AKT2
chr19	40745698	40746042	AKT2
chr19	40747819	40748001	AKT2
chr19	40748415	40748619	AKT2
chr19	40761039	40761201	AKT2
chr19	40762807	40762986	AKT2
chr19	40771099	40771199	AKT2
chr19	41349675	41349907	CYP2A6
chr19	41350510	41350702	CYP2A6
chr19	41351173	41351411	CYP2A6
chr19	41351835	41352027	CYP2A6
chr19	41352754	41352981	CYP2A6
chr19	41354098	41354309	CYP2A6
chr19	41354493	41354693	CYP2A6
chr19	41355697	41355910	CYP2A6
chr19	41356126	41356356	CYP2A6
chr19	41497185	41497406	CYP2B6
chr19	41509829	41510093	CYP2B6
chr19	41510176	41510376	CYP2B6
chr19	41512784	41512995	CYP2B6
chr19	41515098	41515325	CYP2B6
chr19	41515873	41516065	CYP2B6
chr19	41518177	41518415	CYP2B6
chr19	41518553	41518745	CYP2B6
chr19	41522525	41522757	CYP2B6
chr19	42381349	42381478	CD79A
chr19	42383034	42383384	CD79A
chr19	42383579	42383748	CD79A
chr19	42384711	42384830	CD79A
chr19	42384908	42385072	CD79A
chr19	42788831	42788948	CIC
chr19	42790897	42791097	CIC
chr19	42791132	42791417	CIC
chr19	42791446	42791626	CIC
chr19	42791671	42791904	CIC
chr19	42791936	42792152	CIC
chr19	42793014	42793267	CIC
chr19	42793307	42793583	CIC
chr19	42793974	42794128	CIC
chr19	42794359	42795643	CIC
chr19	42795684	42795922	CIC
chr19	42796212	42796384	CIC
chr19	42796426	42796643	CIC
chr19	42796692	42797036	CIC
chr19	42797082	42797458	CIC
chr19	42797718	42798013	CIC
chr19	42798061	42798266	CIC
chr19	42798299	42798481	CIC
chr19	42798730	42798912	CIC
chr19	42798950	42799368	CIC
chr19	45854861	45855004	ERCC2
chr19	45855441	45855635	ERCC2

chr19	45855738	45855932	ERCC2
chr19	45855978	45856099	ERCC2
chr19	45856315	45856438	ERCC2
chr19	45856474	45856617	ERCC2
chr19	45857962	45858134	ERCC2
chr19	45858897	45859011	ERCC2
chr19	45860502	45860654	ERCC2
chr19	45860706	45860826	ERCC2
chr19	45860862	45860982	ERCC2
chr19	45862092	45862195	ERCC2
chr19	45864756	45864925	ERCC2
chr19	45866975	45867194	ERCC2
chr19	45867218	45867402	ERCC2
chr19	45867467	45867614	ERCC2
chr19	45867656	45867830	ERCC2
chr19	45868070	45868237	ERCC2
chr19	45868274	45868441	ERCC2
chr19	45871862	45872026	ERCC2
chr19	45872162	45872275	ERCC2
chr19	45872302	45872430	ERCC2
chr19	45873365	45873515	ERCC2
chr19	45873768	45873823	ERCC2
chr19	50902080	50902335	POLD1
chr19	50902602	50902766	POLD1
chr19	50905009	50905206	POLD1
chr19	50905230	50905406	POLD1
chr19	50905436	50905655	POLD1
chr19	50905685	50905817	POLD1
chr19	50905843	50906023	POLD1
chr19	50906284	50906501	POLD1
chr19	50906724	50906879	POLD1
chr19	50909413	50909604	POLD1
chr19	50909638	50909799	POLD1
chr19	50910214	50910456	POLD1
chr19	50910558	50910697	POLD1
chr19	50912016	50912183	POLD1
chr19	50912353	50912517	POLD1
chr19	50912750	50912948	POLD1
chr19	50916657	50916803	POLD1
chr19	50916973	50917161	POLD1
chr19	50918046	50918272	POLD1
chr19	50918669	50918872	POLD1
chr19	50918955	50919108	POLD1
chr19	50919627	50919810	POLD1
chr19	50919841	50920005	POLD1
chr19	50920276	50920379	POLD1
chr19	50920403	50920551	POLD1
chr19	50921073	50921229	POLD1
chr19	52693324	52693452	PPP2R1A
chr19	52705171	52705312	PPP2R1A
chr19	52709190	52709341	PPP2R1A
chr19	52714487	52714770	PPP2R1A
chr19	52715913	52716111	PPP2R1A
chr19	52716182	52716388	PPP2R1A
chr19	52719006	52719171	PPP2R1A
chr19	52719231	52719352	PPP2R1A
chr19	52719756	52719941	PPP2R1A
chr19	52722918	52723142	PPP2R1A
chr19	52723416	52723527	PPP2R1A
chr19	52724206	52724411	PPP2R1A
chr19	52725326	52725519	PPP2R1A
chr19	52728944	52729086	PPP2R1A
chr19	52729192	52729259	PPP2R1A
chr20	10620120	10620628	JAG1
chr20	10621405	10621606	JAG1
chr20	10621735	10621917	JAG1
chr20	10622082	10622366	JAG1
chr20	10622405	10622565	JAG1
chr20	10623110	10623274	JAG1
chr20	10624400	10624536	JAG1
chr20	10624979	10625057	JAG1
chr20	10625485	10625652	JAG1
chr20	10625765	10625929	JAG1
chr20	10625978	10626142	JAG1
chr20	10626593	10626757	JAG1
chr20	10627561	10627776	JAG1
chr20	10628582	10628783	JAG1
chr20	10629171	10629395	JAG1
chr20	10629683	10629780	JAG1
chr20	10630144	10630308	JAG1
chr20	10630869	10631033	JAG1
chr20	10632203	10632367	JAG1
chr20	10632753	10632923	JAG1
chr20	10633090	10633271	JAG1
chr20	10637020	10637131	JAG1
chr20	10639090	10639395	JAG1
chr20	10644585	10644687	JAG1
chr20	10653323	10653679	JAG1
chr20	10654072	10654203	JAG1
chr20	30253726	30253914	BCL2L1
chr20	30309432	30310046	BCL2L1
chr20	30946553	30946660	ASXL1
chr20	30947085	30947162	ASXL1
chr20	30947524	30947619	ASXL1
chr20	30954161	30954295	ASXL1
chr20	30955504	30955557	ASXL1

chr20	30956789	30956951	ASXL1
chr20	30959555	30959611	ASXL1
chr20	30959941	30959997	ASXL1
chr20	31015905	31016076	ASXL1
chr20	31016102	31016250	ASXL1
chr20	31017115	31017259	ASXL1
chr20	31017678	31017881	ASXL1
chr20	31019098	31019312	ASXL1
chr20	31019360	31019507	ASXL1
chr20	31020657	31020813	ASXL1
chr20	31021061	31021745	ASXL1
chr20	31022209	31025166	ASXL1
chr20	36012531	36012831	SRC
chr20	36014452	36014602	SRC
chr20	36014837	36014905	SRC
chr20	36022272	36022421	SRC
chr20	36022551	36022705	SRC
chr20	36024539	36024739	SRC
chr20	36026076	36026282	SRC
chr20	36028492	36028722	SRC
chr20	36029979	36030106	SRC
chr20	36030812	36031016	SRC
chr20	36031126	36031308	SRC
chr20	36031548	36031807	SRC
chr20	39657682	39657765	TOP1
chr20	39658045	39658120	TOP1
chr20	39690008	39690155	TOP1
chr20	39704785	39704959	TOP1
chr20	39706196	39706302	TOP1
chr20	39708699	39708845	TOP1
chr20	39709779	39709905	TOP1
chr20	39713076	39713233	TOP1
chr20	39721086	39721252	TOP1
chr20	39725834	39726006	TOP1
chr20	39726829	39727002	TOP1
chr20	39728670	39728908	TOP1
chr20	39729823	39730018	TOP1
chr20	39741396	39741590	TOP1
chr20	39742584	39742820	TOP1
chr20	39743985	39744104	TOP1
chr20	39744892	39745057	TOP1
chr20	39746783	39746961	TOP1
chr20	39750310	39750455	TOP1
chr20	39750620	39750820	TOP1
chr20	39751809	39751962	TOP1
chr20	39766256	39766523	PLCG1
chr20	39788220	39788423	PLCG1
chr20	39788484	39788628	PLCG1
chr20	39788720	39788818	PLCG1
chr20	39791066	39791201	PLCG1
chr20	39791256	39791390	PLCG1
chr20	39791572	39791657	PLCG1
chr20	39791817	39791940	PLCG1
chr20	39791992	39792144	PLCG1
chr20	39792329	39792498	PLCG1
chr20	39792535	39792671	PLCG1
chr20	39792695	39792866	PLCG1
chr20	39793547	39793766	PLCG1
chr20	39793859	39794032	PLCG1
chr20	39794064	39794216	PLCG1
chr20	39794253	39794491	PLCG1
chr20	39794808	39795059	PLCG1
chr20	39795090	39795260	PLCG1
chr20	39795293	39795524	PLCG1
chr20	39796466	39796594	PLCG1
chr20	39797381	39797535	PLCG1
chr20	39797693	39797840	PLCG1
chr20	39798071	39798191	PLCG1
chr20	39798726	39798934	PLCG1
chr20	39800807	39800954	PLCG1
chr20	39801035	39801310	PLCG1
chr20	39801343	39801542	PLCG1
chr20	39802034	39802199	PLCG1
chr20	39802266	39802478	PLCG1
chr20	39802537	39802676	PLCG1
chr20	39802738	39802976	PLCG1
chr20	39803081	39803174	PLCG1
chr20	48124431	48124626	PTGIS
chr20	48127539	48127741	PTGIS
chr20	48129591	48129823	PTGIS
chr20	48130738	48130957	PTGIS
chr20	48140569	48140801	PTGIS
chr20	48156081	48156283	PTGIS
chr20	48160816	48161010	PTGIS
chr20	48164352	48164581	PTGIS
chr20	48166577	48166751	PTGIS
chr20	48184554	48184678	PTGIS
chr20	50400778	50401248	SALL4
chr20	50405374	50405705	SALL4
chr20	50406535	50408916	SALL4
chr20	50418792	50418972	SALL4
chr20	54945188	54945421	AURKA
chr20	54945500	54945740	AURKA
chr20	54948438	54948637	AURKA
chr20	54956463	54956652	AURKA
chr20	54958015	54958257	AURKA

chr20	54959300	54959405	AURKA
chr20	54961287	54961614	AURKA
chr20	54963186	54963278	AURKA
chr20	57415136	57415924	GNAS, GNAS-AS1
chr20	57428295	57430413	GNAS
chr20	57430583	57430728	GNAS
chr20	57466756	57466945	GNAS
chr20	57470641	57470764	GNAS
chr20	57473970	57474065	GNAS
chr20	57474978	57475035	GNAS
chr20	57478557	57478665	GNAS
chr20	57478701	57478871	GNAS
chr20	57480412	57480560	GNAS
chr20	57484191	57484296	GNAS
chr20	57484379	57484503	GNAS
chr20	57484550	57484659	GNAS
chr20	57484713	57484884	GNAS
chr20	57484980	57485161	GNAS
chr20	57485363	57485481	GNAS
chr20	57485712	57485909	GNAS
chr20	62331769	62331907	ARFRP1
chr20	62331924	62332079	ARFRP1
chr20	62333156	62333277	ARFRP1
chr20	62333462	62333594	ARFRP1
chr20	62336964	62337164	ARFRP1
chr20	62337683	62337816	ARFRP1
chr20	62337977	62338115	ARFRP1
chr20	62338325	62338468	ARFRP1
chr21	36164250	36164398	RUNX1
chr21	36164406	36164932	RUNX1
chr21	36171572	36171784	RUNX1
chr21	36193939	36194018	RUNX1
chr21	36206681	36206923	RUNX1
chr21	36228684	36228769	RUNX1
chr21	36231745	36231900	RUNX1
chr21	36252828	36253035	RUNX1
chr21	36259114	36259434	RUNX1
chr21	36261948	36262059	RUNX1
chr21	36265196	36265285	RUNX1
chr21	36421113	36421221	RUNX1
chr21	39739531	39739595	
chr21	39755299	39755870	ERG
chr21	39762891	39762989	ERG
chr21	39763555	39763662	ERG
chr21	39764213	39764391	ERG
chr21	39772470	39772592	ERG
chr21	39774453	39774584	ERG
chr21	39775402	39775656	ERG
chr21	39795306	39795508	ERG
chr21	39817301	39817569	ERG
chr21	39870261	39870329	ERG
chr21	39947560	39947649	ERG
chr21	44513186	44513384	U2AF1
chr21	44514555	44514698	U2AF1
chr21	44514739	44514923	U2AF1
chr21	44515522	44515671	U2AF1
chr21	44515778	44515878	U2AF1
chr21	44520537	44520654	U2AF1
chr21	44521450	44521567	U2AF1
chr21	44524399	44524537	U2AF1
chr21	44527535	44527629	U2AF1
chr21	46916223	46916318	COL18A1
chr21	46918234	46918551	COL18A1, SLC19A1
chr21	46934864	46935091	SLC19A1
chr21	46935546	46936079	SLC19A1
chr21	46945705	46945897	SLC19A1
chr21	46950658	46950910	SLC19A1
chr21	46951277	46952087	SLC19A1
chr21	46954426	46954545	SLC19A1
chr21	46957659	46957898	SLC19A1
chr22	19950024	19950363	COMT
chr22	19951063	19951307	COMT, MIR4761
chr22	19951665	19951940	COMT
chr22	19956033	19956284	COMT
chr22	21272197	21272558	CRKL
chr22	21288041	21288557	CRKL
chr22	21303973	21304158	CRKL
chr22	24129331	24129474	SMARCB1
chr22	24133917	24134106	SMARCB1
chr22	24135720	24135900	SMARCB1
chr22	24143105	24143347	SMARCB1
chr22	24145456	24145634	SMARCB1
chr22	24158931	24159148	SMARCB1
chr22	24167386	24167627	SMARCB1
chr22	24175733	24175915	SMARCB1
chr22	24176302	24176392	SMARCB1
chr22	24376397	24376642	GSTT1
chr22	24376796	24377023	GSTT1
chr22	24379335	24379536	GSTT1
chr22	24381674	24381812	GSTT1
chr22	24383860	24383912	GSTT1
chr22	24384094	24384256	GSTT1
chr22	29083859	29083999	CHEK2
chr22	29085097	29085279	CHEK2
chr22	29089994	29090130	CHEK2
chr22	29091089	29091255	CHEK2

chr22	29091672	29091886	CHEK2
chr22	29092863	29093000	CHEK2
chr22	29095800	29095950	CHEK2
chr22	29099467	29099579	CHEK2
chr22	29105968	29106072	CHEK2
chr22	29107871	29108030	CHEK2
chr22	29115357	29115498	CHEK2
chr22	29120939	29121137	CHEK2
chr22	29121205	29121380	CHEK2
chr22	29126382	29126561	CHEK2
chr22	29130365	29130734	CHEK2
chr22	29999962	30000126	NF2
chr22	30032714	30032890	NF2
chr22	30035053	30035226	NF2
chr22	30038165	30038299	NF2
chr22	30050620	30050739	NF2
chr22	30051557	30051690	NF2
chr22	30054152	30054278	NF2
chr22	30057168	30057353	NF2
chr22	30060953	30061078	NF2
chr22	30064296	30064460	NF2
chr22	30067789	30067962	NF2
chr22	30069232	30069500	NF2
chr22	30070799	30070955	NF2
chr22	30074159	30074337	NF2
chr22	30077402	30077615	NF2
chr22	30078942	30079078	NF2
chr22	30090715	30090823	NF2
chr22	38369476	38370230	POLR2F, SOX10
chr22	38373848	38374167	POLR2F, SOX10
chr22	38379338	38379816	POLR2F, SOX10
chr22	41488983	41489127	EP300
chr22	41513165	41513850	EP300
chr22	41521842	41522069	EP300
chr22	41523465	41523777	EP300
chr22	41525868	41526032	EP300
chr22	41527366	41527662	EP300
chr22	41531791	41531935	EP300
chr22	41533631	41533819	EP300
chr22	41536118	41536286	EP300
chr22	41537026	41537251	EP300
chr22	41542717	41542845	EP300
chr22	41543815	41543975	EP300
chr22	41545016	41545204	EP300
chr22	41545739	41546227	EP300
chr22	41547811	41548041	EP300
chr22	41548184	41548379	EP300
chr22	41550973	41551142	EP300
chr22	41553147	41553437	EP300
chr22	41554390	41554529	EP300
chr22	41556620	41556751	EP300
chr22	41558701	41558808	EP300
chr22	41560031	41560159	EP300
chr22	41562577	41562695	EP300
chr22	41564427	41564628	EP300
chr22	41564699	41564896	EP300
chr22	41565481	41565645	EP300
chr22	41566384	41566600	EP300
chr22	41568477	41568692	EP300
chr22	41569601	41569813	EP300
chr22	41572225	41572557	EP300
chr22	41572751	41574985	EP300
chr22	42522550	42522779	CYP2D6, NDUFA6-AS1
chr22	42522827	42523019	CYP2D6, NDUFA6-AS1
chr22	42523423	42523661	CYP2D6, NDUFA6-AS1
chr22	42523818	42524010	CYP2D6, NDUFA6-AS1
chr22	42524150	42524377	CYP2D6, NDUFA6-AS1
chr22	42524760	42524971	CYP2D6, NDUFA6-AS1
chr22	42525009	42525212	CYP2D6, NDUFA6-AS1
chr22	42525357	42525416	CYP2D6, NDUFA6-AS1
chr22	42525714	42525963	CYP2D6, NDUFA6-AS1
chr22	42526588	42526818	CYP2D6, NDUFA6-AS1
chr22	42540383	42540490	CYP2D7
chrX	1314861	1315039	CRLF2
chrX	1317393	1317606	CRLF2
chrX	1317764	1318033	CRLF2
chrX	1321246	1321430	CRLF2
chrX	1325300	1325517	CRLF2
chrX	1327673	1327826	CRLF2
chrX	1331423	1331552	CRLF2
chrX	39909143	39909269	BCOR
chrX	39911336	39911678	BCOR
chrX	39913113	39913320	BCOR
chrX	39913483	39913611	BCOR
chrX	39914595	39914791	BCOR
chrX	39916382	39916599	BCOR
chrX	39921366	39921671	BCOR
chrX	39921973	39922349	BCOR
chrX	39922835	39923230	BCOR
chrX	39923563	39923877	BCOR
chrX	39930200	39930437	BCOR
chrX	39930864	39930968	BCOR
chrX	39931558	39934458	BCOR
chrX	39935681	39935810	BCOR
chrX	39936169	39936240	BCOR
chrX	39937071	39937207	BCOR

chrX	40982856	40983002	USP9X
chrX	40988227	40988423	USP9X
chrX	40990684	40990814	USP9X
chrX	40993952	40994115	USP9X
chrX	40996031	40996300	USP9X
chrX	40999883	41000049	USP9X
chrX	41000193	41000495	USP9X
chrX	41000520	41000709	USP9X
chrX	41002518	41002721	USP9X
chrX	41003749	41003904	USP9X
chrX	41007596	41007853	USP9X
chrX	41010148	41010335	USP9X
chrX	41012175	41012359	USP9X
chrX	41022017	41022155	USP9X
chrX	41025099	41025492	USP9X
chrX	41026709	41026855	USP9X
chrX	41027234	41027496	USP9X
chrX	41029222	41029513	USP9X
chrX	41029697	41029897	USP9X
chrX	41031065	41031236	USP9X
chrX	41043225	41043406	USP9X
chrX	41043624	41043953	USP9X
chrX	41045744	41045920	USP9X
chrX	41047219	41047395	USP9X
chrX	41048536	41048753	USP9X
chrX	41055478	41055637	USP9X
chrX	41055819	41056016	USP9X
chrX	41056591	41056788	USP9X
chrX	41057755	41058028	USP9X
chrX	41060287	41060558	USP9X
chrX	41064530	41064771	USP9X
chrX	41069736	41069960	USP9X
chrX	41073795	41073987	USP9X
chrX	41075126	41075930	USP9X
chrX	41076447	41076621	USP9X
chrX	41077599	41077875	USP9X
chrX	41078329	41078509	USP9X
chrX	41082444	41082680	USP9X
chrX	41083969	41084240	USP9X
chrX	41084276	41084415	USP9X
chrX	41088480	41088687	USP9X
chrX	41088794	41089105	USP9X
chrX	41089728	41089874	USP9X
chrX	41091614	41091802	USP9X
chrX	44732772	44732983	KDM6A
chrX	44733144	44733258	KDM6A
chrX	44820503	44820662	KDM6A
chrX	44833885	44833985	KDM6A
chrX	44870180	44870289	KDM6A
chrX	44879829	44880000	KDM6A
chrX	44894150	44894255	KDM6A
chrX	44896874	44896959	KDM6A
chrX	44910928	44911072	KDM6A
chrX	44913048	44913225	KDM6A
chrX	44918225	44918374	KDM6A
chrX	44918466	44918736	KDM6A
chrX	44919241	44919426	KDM6A
chrX	44919828	44920034	KDM6A
chrX	44920543	44920689	KDM6A
chrX	44921866	44922018	KDM6A
chrX	44922641	44923087	KDM6A
chrX	44928798	44929627	KDM6A
chrX	44935916	44936096	KDM6A
chrX	44937619	44937775	KDM6A
chrX	44938365	44938621	KDM6A
chrX	44941795	44941910	KDM6A
chrX	44941934	44942059	KDM6A
chrX	44942679	44942878	KDM6A
chrX	44945084	44945249	KDM6A
chrX	44948962	44949200	KDM6A
chrX	44949942	44950134	KDM6A
chrX	44966629	44966806	KDM6A
chrX	44969298	44969519	KDM6A
chrX	44970601	44970681	KDM6A
chrX	47004820	47004940	NDUFB11, RBM10
chrX	47006730	47006922	RBM10
chrX	47028688	47028922	RBM10
chrX	47030401	47030682	RBM10
chrX	47032501	47032621	RBM10
chrX	47034392	47034516	RBM10
chrX	47035873	47036010	RBM10
chrX	47038476	47038587	RBM10
chrX	47038692	47038919	RBM10
chrX	47039253	47039464	RBM10
chrX	47039585	47039733	RBM10
chrX	47039792	47039930	RBM10
chrX	47040588	47040825	RBM10
chrX	47040880	47041070	RBM10
chrX	47041122	47041290	RBM10
chrX	47041324	47041466	RBM10
chrX	47041535	47041750	RBM10
chrX	47044428	47044628	RBM10
chrX	47044675	47044791	RBM10
chrX	47044815	47045054	RBM10
chrX	47045089	47045214	RBM10
chrX	47045438	47045595	RBM10

chrX	47045631	47045811	RBM10
chrX	47045847	47046023	RBM10
chrX	47422341	47422487	ARAF
chrX	47422599	47422753	ARAF
chrX	47424170	47424323	ARAF
chrX	47424358	47424563	ARAF
chrX	47424616	47424778	ARAF
chrX	47426012	47426204	ARAF
chrX	47426257	47426335	ARAF
chrX	47426359	47426555	ARAF
chrX	47426603	47426856	ARAF
chrX	47428091	47428318	ARAF
chrX	47428360	47428457	ARAF
chrX	47428912	47429081	ARAF
chrX	47429266	47429448	ARAF
chrX	47430251	47430436	ARAF
chrX	47430696	47430881	ARAF
chrX	48649491	48649761	GATA1
chrX	48650225	48650653	GATA1
chrX	48650704	48650900	GATA1
chrX	48651553	48651729	GATA1
chrX	48652174	48652700	GATA1
chrX	53221900	53222044	KDM5C
chrX	53222123	53222539	KDM5C
chrX	53222593	53222843	KDM5C
chrX	53222929	53223058	KDM5C
chrX	53223295	53223945	KDM5C
chrX	53224087	53224275	KDM5C
chrX	53224387	53224617	KDM5C
chrX	53225072	53225261	KDM5C
chrX	53225842	53226251	KDM5C
chrX	53226927	53227083	KDM5C
chrX	53227646	53227844	KDM5C
chrX	53227920	53228095	KDM5C
chrX	53228133	53228365	KDM5C
chrX	53230706	53230951	KDM5C
chrX	53231010	53231180	KDM5C
chrX	53239570	53239783	KDM5C
chrX	53239832	53240064	KDM5C
chrX	53240653	53240862	KDM5C
chrX	53240943	53241113	KDM5C
chrX	53243845	53244054	KDM5C, KDM5C-IT1
chrX	53244951	53245183	KDM5C
chrX	53245230	53245404	KDM5C
chrX	53246299	53246484	KDM5C
chrX	53246952	53247173	KDM5C
chrX	53247432	53247605	KDM5C
chrX	53249995	53250123	KDM5C
chrX	53253896	53254096	KDM5C
chrX	63409733	63413191	AMER1
chrX	66764963	66766633	AR
chrX	66788819	66788889	AR
chrX	66863072	66863274	AR
chrX	66905826	66905993	AR
chrX	66914489	66914589	AR
chrX	66931218	66931556	AR
chrX	66937294	66937489	AR
chrX	66941649	66941830	AR
chrX	66942619	66942851	AR
chrX	66943502	66943708	AR
chrX	70338579	70338728	MED12
chrX	70339197	70339352	MED12
chrX	70339510	70339752	MED12
chrX	70339838	70340045	MED12
chrX	70340795	70341027	MED12
chrX	70341151	70341312	MED12
chrX	70341386	70341691	MED12
chrX	70342024	70342221	MED12
chrX	70342332	70342482	MED12
chrX	70342562	70342749	MED12
chrX	70342919	70343101	MED12
chrX	70343418	70343595	MED12
chrX	70343983	70344263	MED12
chrX	70344588	70344719	MED12
chrX	70344800	70345021	MED12
chrX	70345175	70345370	MED12
chrX	70345487	70345588	MED12
chrX	70345860	70346029	MED12
chrX	70346165	70346359	MED12
chrX	70346793	70347007	MED12
chrX	70347160	70347342	MED12
chrX	70347717	70347995	MED12
chrX	70348120	70348315	MED12
chrX	70348422	70348593	MED12
chrX	70348938	70349090	MED12
chrX	70349140	70349304	MED12
chrX	70349504	70349730	MED12
chrX	70349859	70350089	MED12
chrX	70351374	70351496	MED12
chrX	70351897	70352081	MED12
chrX	70352201	70352413	MED12
chrX	70352669	70352831	MED12
chrX	70352947	70353087	MED12
chrX	70354181	70354341	MED12
chrX	70354537	70354723	MED12
chrX	70354916	70355128	MED12

chrX	70356105	70356530	MED12
chrX	70356703	70356904	MED12
chrX	70357011	70357267	MED12
chrX	70357382	70357510	MED12
chrX	70357550	70357818	MED12
chrX	70360459	70360732	MED12
chrX	70361054	70361245	MED12
chrX	70361707	70361839	MED12
chrX	70361999	70362093	MED12
chrX	76763803	76764132	ATRX
chrX	76776240	76776419	ATRX
chrX	76776855	76777001	ATRX
chrX	76777715	76777891	ATRX
chrX	76778704	76778904	ATRX
chrX	76812896	76813141	ATRX
chrX	76814114	76814342	ATRX
chrX	76829689	76829848	ATRX
chrX	76845278	76845435	ATRX
chrX	76849140	76849344	ATRX
chrX	76854854	76855074	ATRX
chrX	76855175	76855314	ATRX
chrX	76855877	76856058	ATRX
chrX	76872055	76872223	ATRX
chrX	76874248	76874474	ATRX
chrX	76875837	76876025	ATRX
chrX	76888669	76888897	ATRX
chrX	76889028	76889225	ATRX
chrX	76890059	76890219	ATRX
chrX	76891380	76891572	ATRX
chrX	76907578	76907868	ATRX
chrX	76909562	76909715	ATRX
chrX	76912024	76912168	ATRX
chrX	76918845	76919072	ATRX
chrX	76920108	76920292	ATRX
chrX	76931695	76931818	ATRX
chrX	76936986	76940110	ATRX
chrX	76940405	76940523	ATRX
chrX	76944285	76944445	ATRX
chrX	76949273	76949451	ATRX
chrX	76952039	76952217	ATRX
chrX	76953045	76953148	ATRX
chrX	76954036	76954144	ATRX
chrX	76954336	76954400	ATRX
chrX	76972582	76972745	ATRX
chrX	77041442	77041512	ATRX
chrX	79277743	79277968	TBX22
chrX	79278533	79278764	TBX22
chrX	79279536	79279688	TBX22
chrX	79281076	79281301	TBX22
chrX	79282177	79282392	TBX22
chrX	79282729	79282844	TBX22
chrX	79283464	79283600	TBX22
chrX	79285971	79286635	TBX22
chrX	100604847	100604969	BTK
chrX	100608156	100608364	BTK
chrX	100608832	100609001	BTK
chrX	100609592	100609707	BTK
chrX	100611014	100611281	BTK
chrX	100611746	100611968	BTK
chrX	100612471	100612596	BTK
chrX	100613121	100613173	BTK
chrX	100613268	100613450	BTK
chrX	100613579	100613709	BTK
chrX	100614255	100614360	BTK
chrX	100615050	100615163	BTK
chrX	100615530	100615768	BTK
chrX	100617135	100617253	BTK
chrX	100617523	100617702	BTK
chrX	100624960	100625092	BTK
chrX	100626595	100626714	BTK
chrX	100629498	100629647	BTK
chrX	100630106	100630297	BTK
chrX	110366306	110366531	PAK3
chrX	110385298	110385449	PAK3
chrX	110388062	110388175	PAK3
chrX	110389723	110389818	PAK3
chrX	110390939	110391143	PAK3
chrX	110395612	110395700	PAK3
chrX	110406117	110406299	PAK3
chrX	110406764	110406980	PAK3
chrX	110416220	110416334	PAK3
chrX	110435329	110435428	PAK3
chrX	110435708	110435871	PAK3
chrX	110437503	110437671	PAK3
chrX	110439044	110439194	PAK3
chrX	110439646	110439893	PAK3
chrX	110459623	110459811	PAK3
chrX	110463560	110463700	PAK3
chrX	123156452	123156546	STAG2
chrX	123159664	123159793	STAG2
chrX	123164785	123165000	STAG2
chrX	123171351	123171498	STAG2
chrX	123176393	123176520	STAG2
chrX	123178988	123179243	STAG2
chrX	123181178	123181380	STAG2
chrX	123182829	123182953	STAG2

chrX	123184010	123184184	STAG2
chrX	123184945	123185094	STAG2
chrX	123185139	123185269	STAG2
chrX	123189952	123190110	STAG2
chrX	123191690	123191852	STAG2
chrX	123195048	123195216	STAG2
chrX	123195595	123195749	STAG2
chrX	123196726	123196869	STAG2
chrX	123196940	123197080	STAG2
chrX	123197672	123197926	STAG2
chrX	123199700	123199821	STAG2
chrX	123199999	123200137	STAG2
chrX	123200180	123200311	STAG2
chrX	123202388	123202531	STAG2
chrX	123204973	123205198	STAG2
chrX	123210156	123210346	STAG2
chrX	123211781	123211933	STAG2
chrX	123215204	123215403	STAG2
chrX	123217245	123217424	STAG2
chrX	123220371	123220645	STAG2
chrX	123224399	123224639	STAG2
chrX	123224678	123224839	STAG2
chrX	123227842	123228019	STAG2
chrX	123229196	123229324	STAG2
chrX	123234398	123234472	STAG2
chrX	133511622	133511810	PHF6
chrX	133512009	133512161	PHF6
chrX	133527505	133527689	PHF6
chrX	133527913	133528007	PHF6
chrX	133547492	133547712	PHF6
chrX	133547827	133548021	PHF6
chrX	133549020	133549277	PHF6
chrX	133551173	133551361	PHF6
chrX	133559205	133559385	PHF6
chrX	153760189	153760330	G6PD
chrX	153760377	153760520	G6PD
chrX	153760575	153760702	G6PD
chrX	153760756	153761042	G6PD
chrX	153761131	153761368	G6PD
chrX	153761765	153762047	G6PD
chrX	153762224	153762400	G6PD
chrX	153762527	153762736	G6PD
chrX	153763357	153763625	G6PD
chrX	153764126	153764285	G6PD
chrX	153764330	153764418	G6PD
chrX	153774225	153774403	G6PD, IKBKG
chrX	153774978	153775110	G6PD, IKBKG
chrY	1264861	1265039	
chrY	1267393	1267606	
chrY	1267764	1268033	
chrY	1271246	1271430	
chrY	1275300	1275517	
chrY	1277673	1277826	
chrY	1281423	1281552	
