# Supplementary material for: Cancer immune control needs senescence induction by interferon-dependent cell cycle regulator pathways in tumours
Source: Nat Commun. 2020 Mar 12;11:1335. doi: 10.1038/s41467-020-14987-6 (PMC7067802; doi:10.1038/s41467-020-14987-6)
Supplement: Supplementary file 6 — Supplementary Data 3 [file 41467_2020_14987_MOESM6_ESM.pdf]

Supplementary Data 3 Custom gene panel ssSCv4

Chr	start	end	Gene	Probegroup
chr12	133263827	133264427	POLE	7 _promotor-regions
chr19	50887104	50887704	POLD1	7 _promotor-regions
chr19	50901589	50902189	POLD1	7 _promotor-regions
chr2	47629806	47630406	MSH2	7 _promotor-regions
chr2	47636848	47637448	MSH2	7 _promotor-regions
chr2	48009784	48010384	MSH6	7 _promotor-regions
chr3	37034478	37035078	MLH1	7 _promotor-regions
chr3	37034803	37035403	MLH1	7 _promotor-regions
chr3	37053024	37053624	MLH1	7 _promotor-regions
chr5	1294967	1295567	TERT	7 _promotor-regions
chr5	1295166	1295311	TERT	7 _promotor-regions
chr7	6048579	6049179	PMS2	7 _promotor-regions
chr7	99262809	99262860	rs10264272	IKP_SNPs
chr10	96741027	96741078	rs1057910	IKP_SNPs
chr6	29913272	29913323	rs1061235	IKP_SNPs
chr22	42526668	42526719	rs1065852	IKP_SNPs
chr6	18130892	18130943	rs1142345	IKP_SNPs
chr10	96521631	96521682	rs12248560	IKP_SNPs
chr6	29746843	29746894	rs1633021	IKP_SNPs
chr10	96535220	96535271	rs17884712	IKP_SNPs
chr10	96702021	96702072	rs1799853	IKP_SNPs
chr6	18139202	18139253	rs1800460	IKP_SNPs
chr6	18143929	18143980	rs1800462	IKP_SNPs
chr19	41515237	41515288	rs2279343	IKP_SNPs
chr6	31431754	31431805	rs2395029	IKP_SNPs
chr10	96740955	96741006	rs28371685	IKP_SNPs
chr10	96741032	96741083	rs28371686	IKP_SNPs
chr22	42525746	42525797	rs28371706	IKP_SNPs
chr22	42523779	42523830	rs28371725	IKP_SNPs
chr19	41518195	41518246	rs28399499	IKP_SNPs
chr10	96522437	96522488	rs28399504	IKP_SNPs
chr6	30946122	30946173	rs2844682	IKP_SNPs
chr2	234669593	234669644	rs35350960	IKP_SNPs
chr22	42524218	42524269	rs35742686	IKP_SNPs
chr19	41512815	41512866	rs3745274	IKP_SNPs
chr22	42524921	42524972	rs3892097	IKP_SNPs
chr6	30699358	30699409	rs3909184	IKP_SNPs
chr1	97915588	97915639	rs3918290	IKP_SNPs
chr10	96535147	96535198	rs41291556	IKP_SNPs
chr7	99250368	99250418	rs41303343	IKP_SNPs
chr2	234669118	234669169	rs4148323	IKP_SNPs
chr12	21331523	21331574	rs4149056	IKP_SNPs
chr10	96541590	96541641	rs4244285	IKP_SNPs
chr10	96540384	96540435	rs4986893	IKP_SNPs
chr22	42525060	42525111	rs5030655	IKP_SNPs
chr22	42524150	42524203	rs5030656	IKP_SNPs
chr22	42525009	42525060	rs5030865	IKP_SNPs
chr1	97981317	97981368	rs55886062	IKP_SNPs
chr1	98039393	98039444	rs56038477	IKP_SNPs
chr10	96612469	96612520	rs56337013	IKP_SNPs
chr1	169519023	169519074	rs6025	IKP_SNPs
chr10	96541589	96541640	rs6413438	IKP_SNPs
chr1	97547921	97547972	rs67376798	IKP_SNPs
chr7	99270513	99270564	rs776746	IKP_SNPs
chr2	234668855	234668907	rs8175347	IKP_SNPs
chr16	31104852	31104903	rs9934438	IKP_SNPs
chr18	19995535	19997774	CTAGE1	QC_SNPs_and_regions
chr1	152057441	152060019	TCHHL1	QC_SNPs_and_regions
chr9	5919682	5923309	KIAA2026	QC_SNPs_and_regions
chr9	13150505	13150556	rs10756457	QC_SNPs_and_regions
chr4	68780373	68780424	rs1371932	QC_SNPs_and_regions
chr15	27772650	27772701	rs140679	QC_SNPs_and_regions
chr4	85762359	85762410	rs2046402	QC_SNPs_and_regions
chr6	26091310	26091361	rs2071303	QC_SNPs_and_regions
chr6	49425495	49425546	rs2229384	QC_SNPs_and_regions
chr1	183542361	183542412	rs2274064	QC_SNPs_and_regions
chr1	45973902	45973953	rs2275276	QC_SNPs_and_regions
chr6	101166069	101166120	rs41288423	QC_SNPs_and_regions
chr12	72416209	72416260	rs4290270	QC_SNPs_and_regions
chr13	78475287	78475338	rs5351	QC_SNPs_and_regions
chr13	47469914	47469965	rs6313	QC_SNPs_and_regions
chr3	193209152	193209203	rs6788448	QC_SNPs_and_regions
chr6	79679551	79679602	rs7742431	QC_SNPs_and_regions
chr10	123239535	123243211	FGFR2	selected_fusions
chr10	43604678	43606654	RET	selected_fusions
chr10	43606913	43607546	RET	selected_fusions
chr10	43608411	43609003	RET	selected_fusions
chr10	43609123	43609927	RET	selected_fusions

chr10	43610184	43612031	RET	selected_fusions
chr12	12022903	12037378	ETV6	selected_fusions
chr15	34638236	34640169	C15orf55	selected_fusions
chr16	31195717	31196259	FUS	selected_fusions
chr16	31196500	31198122	FUS	selected_fusions
chr16	31198157	31199645	FUS	selected_fusions
chr16	31200547	31200985	FUS	selected_fusions
chr17	30264503	30267304	SUZ12	selected_fusions
chr17	34149837	34151081	TAF15	selected_fusions
chr17	38487648	38504567	RARA	selected_fusions
chr19	1615519	1615684	TCF3	selected_fusions
chr19	1615820	1619109	TCF3	selected_fusions
chr1	156843751	156844174	NTRK1	selected_fusions
chr1	156844192	156844362	NTRK1	selected_fusions
chr1	156844418	156844697	NTRK1	selected_fusions
chr21	42866505	42870045	TMPRSS2	selected_fusions
chr21	42870116	42879876	TMPRSS2	selected_fusions
chr22	23524426	23595985	BCR	selected_fusions
chr22	23631808	23632525	BCR	selected_fusions
chr22	23632600	23634727	BCR	selected_fusions
chr22	23654023	23655073	BCR	selected_fusions
chr22	29683123	29684594	EWSR1	selected_fusions
chr22	29684775	29687553	EWSR1	selected_fusions
chr22	29687588	29688125	EWSR1	selected_fusions
chr22	29688158	29688476	EWSR1	selected_fusions
chr22	29688595	29692228	EWSR1	selected_fusions
chr22	29692358	29693816	EWSR1	selected_fusions
chr22	29693939	29694722	EWSR1	selected_fusions
chr22	39631879	39639905	PDGFB	selected_fusions
chr2	113977755	113984731	PAX8	selected_fusions
chr2	113984833	113992970	PAX8	selected_fusions
chr2	113993159	113994177	PAX8	selected_fusions
chr2	113994298	113999127	PAX8	selected_fusions
chr2	223066909	223084858	PAX3	selected_fusions
chr2	223085073	223085940	PAX3	selected_fusions
chr2	29446394	29448326	ALK	selected_fusions
chr3	12641307	12641650	RAF1	selected_fusions
chr3	12641914	12645634	RAF1	selected_fusions
chr4	1808661	1808842	FGFR3	selected_fusions
chr6	117641193	117642421	ROS1	selected_fusions
chr6	117642557	117645494	ROS1	selected_fusions
chr6	117645578	117647386	ROS1	selected_fusions
chr6	117647577	117650491	ROS1	selected_fusions
chr6	117650609	117658334	ROS1	selected_fusions
chr6	135515598	135516885	MYB	selected_fusions
chr6	135521553	135522776	MYB	selected_fusions
chr6	135524462	135539001	MYB	selected_fusions
chr7	13978871	14017051	ETV1	selected_fusions
chr7	14027798	14028632	ETV1	selected_fusions
chr7	140481493	140482820	BRAF	selected_fusions
chr7	140482957	140487347	BRAF	selected_fusions
chr7	140487384	140494107	BRAF	selected_fusions
chr7	140494267	140500161	BRAF	selected_fusions
chr7	55221845	55223522	EGFR	selected_fusions
chr8	38271322	38271435	FGFR1	selected_fusions
chr9	87285875	87317073	NTRK2	selected_fusions
chrX	48121250	48123216	SSX1	selected_fusions
chrX	48891766	48895534	TFE3	selected_fusions
chrX	48895639	48895721	TFE3	selected_fusions
chrX	48895967	48896631	TFE3	selected_fusions
chr7	87133533	87133790	ABCB1	693_selected_genes
chr7	87135187	87135384	ABCB1	693_selected_genes
chr7	87138565	87138822	ABCB1	693_selected_genes
chr7	87144521	87144769	ABCB1	693_selected_genes
chr7	87145799	87146006	ABCB1	693_selected_genes
chr7	87148616	87148807	ABCB1	693_selected_genes
chr7	87150066	87150217	ABCB1	693_selected_genes
chr7	87160584	87160838	ABCB1	693_selected_genes
chr7	87165748	87165882	ABCB1	693_selected_genes
chr7	87168558	87168686	ABCB1	693_selected_genes
chr7	87170647	87170805	ABCB1	693_selected_genes
chr7	87173419	87173616	ABCB1	693_selected_genes
chr7	87174113	87174340	ABCB1	693_selected_genes
chr7	87175153	87175365	ABCB1	693_selected_genes
chr7	87178638	87178859	ABCB1	693_selected_genes
chr7	87179141	87179395	ABCB1	693_selected_genes
chr7	87179461	87179637	ABCB1	693_selected_genes
chr7	87179758	87179919	ABCB1	693_selected_genes
chr7	87180015	87180179	ABCB1	693_selected_genes
chr7	87183051	87183273	ABCB1	693_selected_genes
chr7	87190553	87190728	ABCB1	693_selected_genes
chr7	87195360	87195582	ABCB1	693_selected_genes
chr7	87196075	87196317	ABCB1	693_selected_genes
chr7	87199462	87199564	ABCB1	693_selected_genes

chr7	87214802	87215021	ABCB1	693_selected_genes
chr7	87225056	87225155	ABCB1	693_selected_genes
chr7	87229407	87229525	ABCB1	693_selected_genes
chr10	101542576	101542659	ABCC2	693_selected_genes
chr10	101544339	101544563	ABCC2	693_selected_genes
chr10	101551965	101552141	ABCC2	693_selected_genes
chr10	101553282	101553467	ABCC2	693_selected_genes
chr10	101553623	101553781	ABCC2	693_selected_genes
chr10	101554144	101554250	ABCC2	693_selected_genes
chr10	101556828	101557128	ABCC2	693_selected_genes
chr10	101558938	101559152	ABCC2	693_selected_genes
chr10	101560117	101560345	ABCC2	693_selected_genes
chr10	101563750	101564055	ABCC2	693_selected_genes
chr10	101565113	101565229	ABCC2	693_selected_genes
chr10	101567115	101567303	ABCC2	693_selected_genes
chr10	101567814	101568011	ABCC2	693_selected_genes
chr10	101569865	101570000	ABCC2	693_selected_genes
chr10	101571267	101571384	ABCC2	693_selected_genes
chr10	101572749	101572926	ABCC2	693_selected_genes
chr10	101577039	101577266	ABCC2	693_selected_genes
chr10	101578521	101578739	ABCC2	693_selected_genes
chr10	101578820	101579051	ABCC2	693_selected_genes
chr10	101590038	101590215	ABCC2	693_selected_genes
chr10	101590447	101590633	ABCC2	693_selected_genes
chr10	101591342	101591612	ABCC2	693_selected_genes
chr10	101591708	101591913	ABCC2	693_selected_genes
chr10	101594111	101594317	ABCC2	693_selected_genes
chr10	101595822	101596072	ABCC2	693_selected_genes
chr10	101601698	101601875	ABCC2	693_selected_genes
chr10	101603530	101603682	ABCC2	693_selected_genes
chr10	101604053	101604247	ABCC2	693_selected_genes
chr10	101605355	101605564	ABCC2	693_selected_genes
chr10	101606692	101606909	ABCC2	693_selected_genes
chr10	101610333	101610578	ABCC2	693_selected_genes
chr10	101611233	101611413	ABCC2	693_selected_genes
chr13	95673803	95673961	ABCC4	693_selected_genes
chr13	95686833	95687018	ABCC4	693_selected_genes
chr13	95695910	95696066	ABCC4	693_selected_genes
chr13	95696494	95696717	ABCC4	693_selected_genes
chr13	95705323	95705463	ABCC4	693_selected_genes
chr13	95714932	95715138	ABCC4	693_selected_genes
chr13	95723890	95724132	ABCC4	693_selected_genes
chr13	95725432	95725583	ABCC4	693_selected_genes
chr13	95726442	95726603	ABCC4	693_selected_genes
chr13	95727660	95727830	ABCC4	693_selected_genes
chr13	95735368	95735569	ABCC4	693_selected_genes
chr13	95748363	95748458	ABCC4	693_selected_genes
chr13	95768150	95768280	ABCC4	693_selected_genes
chr13	95813417	95813614	ABCC4	693_selected_genes
chr13	95815350	95815495	ABCC4	693_selected_genes
chr13	95815838	95815926	ABCC4	693_selected_genes
chr13	95816606	95816797	ABCC4	693_selected_genes
chr13	95818386	95818646	ABCC4	693_selected_genes
chr13	95822760	95822907	ABCC4	693_selected_genes
chr13	95829935	95830072	ABCC4	693_selected_genes
chr13	95830225	95830370	ABCC4	693_selected_genes
chr13	95838929	95839171	ABCC4	693_selected_genes
chr13	95840681	95840821	ABCC4	693_selected_genes
chr13	95847064	95847216	ABCC4	693_selected_genes
chr13	95858760	95859060	ABCC4	693_selected_genes
chr13	95860028	95860204	ABCC4	693_selected_genes
chr13	95861662	95861876	ABCC4	693_selected_genes
chr13	95862920	95863060	ABCC4	693_selected_genes
chr13	95886838	95887113	ABCC4	693_selected_genes
chr13	95899203	95899374	ABCC4	693_selected_genes
chr13	95899871	95900032	ABCC4	693_selected_genes
chr13	95921650	95921701	ABCC4	693_selected_genes
chr13	95953469	95953593	ABCC4	693_selected_genes
chr4	89013360	89013558	ABCG2	693_selected_genes
chr4	89015703	89015836	ABCG2	693_selected_genes
chr4	89016646	89016786	ABCG2	693_selected_genes
chr4	89018579	89018784	ABCG2	693_selected_genes
chr4	89020450	89020625	ABCG2	693_selected_genes
chr4	89022356	89022496	ABCG2	693_selected_genes
chr4	89028310	89028443	ABCG2	693_selected_genes
chr4	89034429	89034730	ABCG2	693_selected_genes
chr4	89036083	89036235	ABCG2	693_selected_genes
chr4	89039235	89039437	ABCG2	693_selected_genes
chr4	89042761	89042969	ABCG2	693_selected_genes
chr4	89052187	89052390	ABCG2	693_selected_genes
chr4	89052929	89053094	ABCG2	693_selected_genes
chr4	89053702	89053812	ABCG2	693_selected_genes
chr4	89060919	89061191	ABCG2	693_selected_genes
chr4	89079703	89079848	ABCG2	693_selected_genes

chr4	89080331	89080416	ABCG2	693_selected_genes
chr9	133589681	133589867	ABL1	693_selected_genes
chr9	133710808	133710937	ABL1	693_selected_genes
chr9	133729425	133729649	ABL1	693_selected_genes
chr9	133730162	133730508	ABL1	693_selected_genes
chr9	133738124	133738447	ABL1	693_selected_genes
chr9	133747490	133747625	ABL1	693_selected_genes
chr9	133748221	133748449	ABL1	693_selected_genes
chr9	133750229	133750464	ABL1	693_selected_genes
chr9	133753776	133753979	ABL1	693_selected_genes
chr9	133755429	133755569	ABL1	693_selected_genes
chr9	133755861	133756076	ABL1	693_selected_genes
chr9	133759330	133761095	ABL1	693_selected_genes
chr1	179076827	179078601	ABL2	693_selected_genes
chr1	179079391	179079615	ABL2	693_selected_genes
chr1	179079914	179080005	ABL2	693_selected_genes
chr1	179081418	179081558	ABL2	693_selected_genes
chr1	179083987	179084190	ABL2	693_selected_genes
chr1	179086373	179086676	ABL2	693_selected_genes
chr1	179087696	179087924	ABL2	693_selected_genes
chr1	179089299	179089434	ABL2	693_selected_genes
chr1	179090704	179091027	ABL2	693_selected_genes
chr1	179095486	179095832	ABL2	693_selected_genes
chr1	179100420	179100641	ABL2	693_selected_genes
chr1	179102421	179102534	ABL2	693_selected_genes
chr1	179112042	179112204	ABL2	693_selected_genes
chr1	179198350	179198557	ABL2	693_selected_genes
chr9	32405479	32405626	ACO1	693_selected_genes
chr9	32407233	32407452	ACO1	693_selected_genes
chr9	32408486	32408674	ACO1	693_selected_genes
chr9	32418100	32418220	ACO1	693_selected_genes
chr9	32418300	32418534	ACO1	693_selected_genes
chr9	32419010	32419200	ACO1	693_selected_genes
chr9	32420828	32421050	ACO1	693_selected_genes
chr9	32423291	32423442	ACO1	693_selected_genes
chr9	32424521	32424688	ACO1	693_selected_genes
chr9	32425810	32426020	ACO1	693_selected_genes
chr9	32427273	32427459	ACO1	693_selected_genes
chr9	32429391	32429526	ACO1	693_selected_genes
chr9	32430390	32430597	ACO1	693_selected_genes
chr9	32431691	32431866	ACO1	693_selected_genes
chr9	32433700	32433855	ACO1	693_selected_genes
chr9	32434531	32434724	ACO1	693_selected_genes
chr9	32436222	32436420	ACO1	693_selected_genes
chr9	32440437	32440610	ACO1	693_selected_genes
chr9	32448868	32449104	ACO1	693_selected_genes
chr9	32449970	32450134	ACO1	693_selected_genes
chr9	32450184	32450243	ACO1	693_selected_genes
chr7	5567353	5567547	ACTB	693_selected_genes
chr7	5567609	5567841	ACTB	693_selected_genes
chr7	5567886	5568375	ACTB	693_selected_genes
chr7	5568650	5568715	ACTB	693_selected_genes
chr7	5568766	5569056	ACTB	693_selected_genes
chr7	5569140	5569319	ACTB	693_selected_genes
chr7	5569411	5569464	ACTB	693_selected_genes
chr12	52345502	52345643	ACVR1B	693_selected_genes
chr12	52369023	52369313	ACVR1B	693_selected_genes
chr12	52370085	52370384	ACVR1B	693_selected_genes
chr12	52374727	52375008	ACVR1B	693_selected_genes
chr12	52376444	52376617	ACVR1B	693_selected_genes
chr12	52377757	52377975	ACVR1B	693_selected_genes
chr12	52378950	52379157	ACVR1B	693_selected_genes
chr12	52380576	52380954	ACVR1B	693_selected_genes
chr12	52385621	52385841	ACVR1B	693_selected_genes
chr12	52387743	52387919	ACVR1B	693_selected_genes
chr2	148602696	148602801	ACVR2A	693_selected_genes
chr2	148653844	148654102	ACVR2A	693_selected_genes
chr2	148657001	148657161	ACVR2A	693_selected_genes
chr2	148657287	148657492	ACVR2A	693_selected_genes
chr2	148672734	148672928	ACVR2A	693_selected_genes
chr2	148674826	148675020	ACVR2A	693_selected_genes
chr2	148675990	148676186	ACVR2A	693_selected_genes
chr2	148677773	148677938	ACVR2A	693_selected_genes
chr2	148680516	148680705	ACVR2A	693_selected_genes
chr2	148683574	148683755	ACVR2A	693_selected_genes
chr2	148684623	148684868	ACVR2A	693_selected_genes
chr15	58889630	58889865	ADAM10	693_selected_genes
chr15	58891771	58891948	ADAM10	693_selected_genes
chr15	58902470	58902741	ADAM10	693_selected_genes
chr15	58903172	58903331	ADAM10	693_selected_genes
chr15	58903981	58904215	ADAM10	693_selected_genes
chr15	58913644	58913845	ADAM10	693_selected_genes
chr15	58919873	58920107	ADAM10	693_selected_genes
chr15	58925369	58925583	ADAM10	693_selected_genes

chr15	58932950	58933184	ADAM10	693_selected_genes
chr15	58936059	58936202	ADAM10	693_selected_genes
chr15	58938228	58938428	ADAM10	693_selected_genes
chr15	58957270	58957421	ADAM10	693_selected_genes
chr15	58968450	58968517	ADAM10	693_selected_genes
chr15	58971297	58971506	ADAM10	693_selected_genes
chr15	58974369	58974538	ADAM10	693_selected_genes
chr15	58990453	58990628	ADAM10	693_selected_genes
chr15	59009750	59009951	ADAM10	693_selected_genes
chr15	59041653	59041758	ADAM10	693_selected_genes
chr7	45614117	45614806	ADCY1	693_selected_genes
chr7	45632332	45632532	ADCY1	693_selected_genes
chr7	45649952	45650121	ADCY1	693_selected_genes
chr7	45662205	45662367	ADCY1	693_selected_genes
chr7	45688243	45688421	ADCY1	693_selected_genes
chr7	45697300	45697509	ADCY1	693_selected_genes
chr7	45699615	45699807	ADCY1	693_selected_genes
chr7	45701632	45701838	ADCY1	693_selected_genes
chr7	45703862	45703999	ADCY1	693_selected_genes
chr7	45717442	45717687	ADCY1	693_selected_genes
chr7	45717739	45717887	ADCY1	693_selected_genes
chr7	45719282	45719417	ADCY1	693_selected_genes
chr7	45724552	45724692	ADCY1	693_selected_genes
chr7	45725535	45725839	ADCY1	693_selected_genes
chr7	45726120	45726297	ADCY1	693_selected_genes
chr7	45742949	45743116	ADCY1	693_selected_genes
chr7	45743173	45743370	ADCY1	693_selected_genes
chr7	45744091	45744240	ADCY1	693_selected_genes
chr7	45747923	45748088	ADCY1	693_selected_genes
chr7	45750101	45750276	ADCY1	693_selected_genes
chr7	45753266	45753619	ADCY1	693_selected_genes
chr8	37654761	37655077	ADGRA2	693_selected_genes
chr8	37672388	37672510	ADGRA2	693_selected_genes
chr8	37686380	37686502	ADGRA2	693_selected_genes
chr8	37686753	37686875	ADGRA2	693_selected_genes
chr8	37686980	37687102	ADGRA2	693_selected_genes
chr8	37687343	37687557	ADGRA2	693_selected_genes
chr8	37688202	37688466	ADGRA2	693_selected_genes
chr8	37688915	37689130	ADGRA2	693_selected_genes
chr8	37690502	37690751	ADGRA2	693_selected_genes
chr8	37691180	37691380	ADGRA2	693_selected_genes
chr8	37691459	37691671	ADGRA2	693_selected_genes
chr8	37692666	37692941	ADGRA2	693_selected_genes
chr8	37693046	37693313	ADGRA2	693_selected_genes
chr8	37695223	37695482	ADGRA2	693_selected_genes
chr8	37696448	37696626	ADGRA2	693_selected_genes
chr8	37696991	37697165	ADGRA2	693_selected_genes
chr8	37697613	37697809	ADGRA2	693_selected_genes
chr8	37698252	37698392	ADGRA2	693_selected_genes
chr8	37698578	37699898	ADGRA2	693_selected_genes
chr14	23442626	23442802	AJUBA	693_selected_genes
chr14	23443230	23443349	AJUBA	693_selected_genes
chr14	23444022	23444124	AJUBA	693_selected_genes
chr14	23444157	23444338	AJUBA	693_selected_genes
chr14	23445639	23445752	AJUBA	693_selected_genes
chr14	23445828	23445946	AJUBA	693_selected_genes
chr14	23447527	23447679	AJUBA	693_selected_genes
chr14	23448116	23448270	AJUBA	693_selected_genes
chr14	23450444	23451500	AJUBA	693_selected_genes
chr7	91570388	91570486	AKAP9	693_selected_genes
chr7	91599761	91599847	AKAP9	693_selected_genes
chr7	91602999	91603307	AKAP9	693_selected_genes
chr7	91609577	91609672	AKAP9	693_selected_genes
chr7	91621446	91621550	AKAP9	693_selected_genes
chr7	91622173	91622394	AKAP9	693_selected_genes
chr7	91623909	91624115	AKAP9	693_selected_genes
chr7	91624891	91625154	AKAP9	693_selected_genes
chr7	91630136	91632574	AKAP9	693_selected_genes
chr7	91641717	91641981	AKAP9	693_selected_genes
chr7	91643537	91643667	AKAP9	693_selected_genes
chr7	91645417	91645606	AKAP9	693_selected_genes
chr7	91646305	91646441	AKAP9	693_selected_genes
chr7	91651526	91651691	AKAP9	693_selected_genes
chr7	91652102	91652348	AKAP9	693_selected_genes
chr7	91659183	91659330	AKAP9	693_selected_genes
chr7	91660800	91660943	AKAP9	693_selected_genes
chr7	91667707	91668111	AKAP9	693_selected_genes
chr7	91669962	91670237	AKAP9	693_selected_genes
chr7	91671334	91671525	AKAP9	693_selected_genes
chr7	91671956	91672110	AKAP9	693_selected_genes
chr7	91674296	91674552	AKAP9	693_selected_genes
chr7	91682014	91682297	AKAP9	693_selected_genes
chr7	91690548	91690761	AKAP9	693_selected_genes
chr7	91691562	91691825	AKAP9	693_selected_genes

chr7	91694519	91694802	AKAP9	693_selected_genes
chr7	91695715	91695885	AKAP9	693_selected_genes
chr7	91699318	91699545	AKAP9	693_selected_genes
chr7	91700193	91700348	AKAP9	693_selected_genes
chr7	91706143	91706346	AKAP9	693_selected_genes
chr7	91706984	91707214	AKAP9	693_selected_genes
chr7	91708367	91709491	AKAP9	693_selected_genes
chr7	91711810	91712001	AKAP9	693_selected_genes
chr7	91712458	91713034	AKAP9	693_selected_genes
chr7	91713928	91714042	AKAP9	693_selected_genes
chr7	91714107	91714279	AKAP9	693_selected_genes
chr7	91714783	91715025	AKAP9	693_selected_genes
chr7	91715516	91715755	AKAP9	693_selected_genes
chr7	91718673	91719032	AKAP9	693_selected_genes
chr7	91722385	91722655	AKAP9	693_selected_genes
chr7	91724311	91724512	AKAP9	693_selected_genes
chr7	91725977	91726696	AKAP9	693_selected_genes
chr7	91726874	91727133	AKAP9	693_selected_genes
chr7	91727397	91727553	AKAP9	693_selected_genes
chr7	91728975	91729208	AKAP9	693_selected_genes
chr7	91730144	91730395	AKAP9	693_selected_genes
chr7	91731882	91732165	AKAP9	693_selected_genes
chr7	91734966	91735102	AKAP9	693_selected_genes
chr7	91736581	91736761	AKAP9	693_selected_genes
chr7	91737782	91738058	AKAP9	693_selected_genes
chr7	91739410	91739498	AKAP9	693_selected_genes
chr14	105235897	105235988	AKT1	693_selected_genes
chr14	105236652	105236782	AKT1	693_selected_genes
chr14	105237056	105237209	AKT1	693_selected_genes
chr14	105238676	105238814	AKT1	693_selected_genes
chr14	105239189	105239454	AKT1	693_selected_genes
chr14	105239562	105239741	AKT1	693_selected_genes
chr14	105239766	105239942	AKT1	693_selected_genes
chr14	105240223	105240342	AKT1	693_selected_genes
chr14	105241249	105241365	AKT1	693_selected_genes
chr14	105241387	105241569	AKT1	693_selected_genes
chr14	105241963	105242161	AKT1	693_selected_genes
chr14	105242970	105243132	AKT1	693_selected_genes
chr14	105246399	105246578	AKT1	693_selected_genes
chr14	105258909	105259005	AKT1	693_selected_genes
chr19	40739753	40739883	AKT2	693_selected_genes
chr19	40740361	40740503	AKT2	693_selected_genes
chr19	40740753	40741079	AKT2	693_selected_genes
chr19	40741144	40741282	AKT2	693_selected_genes
chr19	40741321	40741399	AKT2	693_selected_genes
chr19	40741771	40742036	AKT2	693_selected_genes
chr19	40742138	40742317	AKT2	693_selected_genes
chr19	40743850	40744023	AKT2	693_selected_genes
chr19	40744786	40744905	AKT2	693_selected_genes
chr19	40745926	40746053	AKT2	693_selected_genes
chr19	40746143	40746226	AKT2	693_selected_genes
chr19	40747819	40748001	AKT2	693_selected_genes
chr19	40748415	40748619	AKT2	693_selected_genes
chr19	40750292	40750346	AKT2	693_selected_genes
chr19	40761038	40761231	AKT2	693_selected_genes
chr19	40762807	40762986	AKT2	693_selected_genes
chr19	40771103	40771199	AKT2	693_selected_genes
chr1	243663019	243663113	AKT3	693_selected_genes
chr1	243668525	243668661	AKT3	693_selected_genes
chr1	243675600	243675753	AKT3	693_selected_genes
chr1	243708786	243708924	AKT3	693_selected_genes
chr1	243716005	243716270	AKT3	693_selected_genes
chr1	243726996	243727175	AKT3	693_selected_genes
chr1	243736202	243736375	AKT3	693_selected_genes
chr1	243776947	243777066	AKT3	693_selected_genes
chr1	243778372	243778488	AKT3	693_selected_genes
chr1	243800887	243801069	AKT3	693_selected_genes
chr1	243809169	243809364	AKT3	693_selected_genes
chr1	243828048	243828210	AKT3	693_selected_genes
chr1	243858867	243859043	AKT3	693_selected_genes
chr1	244006401	244006497	AKT3	693_selected_genes
chr2	29416064	29416813	ALK	693_selected_genes
chr2	29419610	29419751	ALK	693_selected_genes
chr2	29420382	29420567	ALK	693_selected_genes
chr2	29430011	29430163	ALK	693_selected_genes
chr2	29430885	29430936	ALK	693_selected_genes
chr2	29432626	29432769	ALK	693_selected_genes
chr2	29436824	29436972	ALK	693_selected_genes
chr2	29443546	29443726	ALK	693_selected_genes
chr2	29443828	29444128	ALK	693_selected_genes
chr2	29445184	29445299	ALK	693_selected_genes
chr2	29445357	29445498	ALK	693_selected_genes
chr2	29446182	29446419	ALK	693_selected_genes
chr2	29448301	29448456	ALK	693_selected_genes

chr2	29449762	29449965	ALK	693_selected_genes
chr2	29450414	29450563	ALK	693_selected_genes
chr2	29451724	29451957	ALK	693_selected_genes
chr2	29455144	29455339	ALK	693_selected_genes
chr2	29456405	29456587	ALK	693_selected_genes
chr2	29462520	29462721	ALK	693_selected_genes
chr2	29473945	29474158	ALK	693_selected_genes
chr2	29497939	29498118	ALK	693_selected_genes
chr2	29498242	29498387	ALK	693_selected_genes
chr2	29519728	29519948	ALK	693_selected_genes
chr2	29541144	29541295	ALK	693_selected_genes
chr2	29543591	29543773	ALK	693_selected_genes
chr2	29551190	29551372	ALK	693_selected_genes
chr2	29606572	29606750	ALK	693_selected_genes
chr2	29754755	29755007	ALK	693_selected_genes
chr2	29917690	29917905	ALK	693_selected_genes
chr2	29940414	29940588	ALK	693_selected_genes
chr2	30142833	30143550	ALK	693_selected_genes
chr17	7976063	7976293	ALOX12B	693_selected_genes
chr17	7976440	7976661	ALOX12B	693_selected_genes
chr17	7976949	7977100	ALOX12B	693_selected_genes
chr17	7978887	7979059	ALOX12B	693_selected_genes
chr17	7979467	7979687	ALOX12B	693_selected_genes
chr17	7979949	7980086	ALOX12B	693_selected_genes
chr17	7980282	7980536	ALOX12B	693_selected_genes
chr17	7982688	7982882	ALOX12B	693_selected_genes
chr17	7983061	7983284	ALOX12B	693_selected_genes
chr17	7983527	7983681	ALOX12B	693_selected_genes
chr17	7983950	7984123	ALOX12B	693_selected_genes
chr17	7984176	7984319	ALOX12B	693_selected_genes
chr17	7984398	7984530	ALOX12B	693_selected_genes
chr17	7989308	7989563	ALOX12B	693_selected_genes
chr17	7990588	7990785	ALOX12B	693_selected_genes
chrX	63405971	63406079	AMER1	693_selected_genes
chrX	63409733	63413191	AMER1	693_selected_genes
chr10	61802423	61802542	ANK3	693_selected_genes
chr10	61813409	61813510	ANK3	693_selected_genes
chr10	61815390	61815821	ANK3	693_selected_genes
chr10	61819072	61819213	ANK3	693_selected_genes
chr10	61819430	61819816	ANK3	693_selected_genes
chr10	61822843	61823037	ANK3	693_selected_genes
chr10	61823889	61824071	ANK3	693_selected_genes
chr10	61827667	61827792	ANK3	693_selected_genes
chr10	61828369	61836231	ANK3	693_selected_genes
chr10	61840269	61840403	ANK3	693_selected_genes
chr10	61841882	61841959	ANK3	693_selected_genes
chr10	61842347	61842520	ANK3	693_selected_genes
chr10	61843224	61843400	ANK3	693_selected_genes
chr10	61844334	61844613	ANK3	693_selected_genes
chr10	61844889	61845036	ANK3	693_selected_genes
chr10	61846409	61846667	ANK3	693_selected_genes
chr10	61847879	61848141	ANK3	693_selected_genes
chr10	61865637	61865842	ANK3	693_selected_genes
chr10	61867920	61868069	ANK3	693_selected_genes
chr10	61868562	61868837	ANK3	693_selected_genes
chr10	61873957	61874114	ANK3	693_selected_genes
chr10	61894003	61894156	ANK3	693_selected_genes
chr10	61898665	61898870	ANK3	693_selected_genes
chr10	61900084	61900150	ANK3	693_selected_genes
chr10	61905700	61905804	ANK3	693_selected_genes
chr10	61926323	61926436	ANK3	693_selected_genes
chr10	61926556	61926679	ANK3	693_selected_genes
chr10	61932040	61932186	ANK3	693_selected_genes
chr10	61932632	61932781	ANK3	693_selected_genes
chr10	61932820	61932969	ANK3	693_selected_genes
chr10	61941061	61941210	ANK3	693_selected_genes
chr10	61946447	61946695	ANK3	693_selected_genes
chr10	61955877	61956026	ANK3	693_selected_genes
chr10	61956259	61956408	ANK3	693_selected_genes
chr10	61958072	61958320	ANK3	693_selected_genes
chr10	61959861	61960046	ANK3	693_selected_genes
chr10	61962734	61962883	ANK3	693_selected_genes
chr10	61965524	61965673	ANK3	693_selected_genes
chr10	61967768	61968016	ANK3	693_selected_genes
chr10	61973144	61973293	ANK3	693_selected_genes
chr10	61994420	61994569	ANK3	693_selected_genes
chr10	62021591	62021740	ANK3	693_selected_genes
chr10	62023567	62023803	ANK3	693_selected_genes
chr10	62029863	62030012	ANK3	693_selected_genes
chr10	62038506	62038655	ANK3	693_selected_genes
chr10	62038782	62038931	ANK3	693_selected_genes
chr10	62039270	62039422	ANK3	693_selected_genes
chr10	62060051	62060137	ANK3	693_selected_genes
chr10	62149157	62149321	ANK3	693_selected_genes

chr10	62332197	62332310	ANK3	693_selected_genes
chr10	62374918	62375007	ANK3	693_selected_genes
chr10	62492995	62493102	ANK3	693_selected_genes
chr12	99042112	99042300	APAF1	693_selected_genes
chr12	99042378	99042683	APAF1	693_selected_genes
chr12	99043239	99043487	APAF1	693_selected_genes
chr12	99052912	99053146	APAF1	693_selected_genes
chr12	99056208	99056371	APAF1	693_selected_genes
chr12	99056427	99056609	APAF1	693_selected_genes
chr12	99059305	99059594	APAF1	693_selected_genes
chr12	99059942	99060160	APAF1	693_selected_genes
chr12	99061265	99061447	APAF1	693_selected_genes
chr12	99064725	99064889	APAF1	693_selected_genes
chr12	99065287	99065522	APAF1	693_selected_genes
chr12	99071177	99071354	APAF1	693_selected_genes
chr12	99074029	99074205	APAF1	693_selected_genes
chr12	99076895	99077077	APAF1	693_selected_genes
chr12	99080500	99080676	APAF1	693_selected_genes
chr12	99093160	99093372	APAF1	693_selected_genes
chr12	99097123	99097302	APAF1	693_selected_genes
chr12	99100237	99100413	APAF1	693_selected_genes
chr12	99102337	99102507	APAF1	693_selected_genes
chr12	99106071	99106238	APAF1	693_selected_genes
chr12	99109179	99109355	APAF1	693_selected_genes
chr12	99116945	99117115	APAF1	693_selected_genes
chr12	99117391	99117567	APAF1	693_selected_genes
chr12	99119167	99119343	APAF1	693_selected_genes
chr12	99120925	99121119	APAF1	693_selected_genes
chr12	99126172	99126369	APAF1	693_selected_genes
chr5	112043389	112043604	APC	693_selected_genes
chr5	112090562	112090747	APC	693_selected_genes
chr5	112101997	112102132	APC	693_selected_genes
chr5	112102860	112103112	APC	693_selected_genes
chr5	112111300	112111459	APC	693_selected_genes
chr5	112116461	112116630	APC	693_selected_genes
chr5	112128117	112128251	APC	693_selected_genes
chr5	112136950	112137105	APC	693_selected_genes
chr5	112145797	112145877	APC	693_selected_genes
chr5	112151166	112151315	APC	693_selected_genes
chr5	112154637	112155066	APC	693_selected_genes
chr5	112157567	112157713	APC	693_selected_genes
chr5	112158978	112159082	APC	693_selected_genes
chr5	112162779	112162969	APC	693_selected_genes
chr5	112163600	112163728	APC	693_selected_genes
chr5	112164527	112164694	APC	693_selected_genes
chr5	112170622	112170887	APC	693_selected_genes
chr5	112173224	112179848	APC	693_selected_genes
chr5	112192320	112192395	APC	693_selected_genes
chr5	112198179	112198258	APC	693_selected_genes
chr18	10454953	10455061	APCDD1	693_selected_genes
chr18	10468440	10468674	APCDD1	693_selected_genes
chr18	10471501	10472083	APCDD1	693_selected_genes
chr18	10474077	10474269	APCDD1	693_selected_genes
chr18	10475858	10476364	APCDD1	693_selected_genes
chr18	10485433	10485805	APCDD1	693_selected_genes
chr18	10487561	10488060	APCDD1	693_selected_genes
chrX	66764963	66766629	AR	693_selected_genes
chrX	66788819	66788889	AR	693_selected_genes
chrX	66863072	66863274	AR	693_selected_genes
chrX	66900536	66900621	AR	693_selected_genes
chrX	66905826	66905993	AR	693_selected_genes
chrX	66914489	66914589	AR	693_selected_genes
chrX	66931218	66931556	AR	693_selected_genes
chrX	66937294	66937489	AR	693_selected_genes
chrX	66941649	66941830	AR	693_selected_genes
chrX	66942643	66942851	AR	693_selected_genes
chrX	66943502	66943708	AR	693_selected_genes
chrX	47422341	47422487	ARAF	693_selected_genes
chrX	47422599	47422753	ARAF	693_selected_genes
chrX	47424170	47424323	ARAF	693_selected_genes
chrX	47424358	47424563	ARAF	693_selected_genes
chrX	47424616	47424778	ARAF	693_selected_genes
chrX	47426012	47426204	ARAF	693_selected_genes
chrX	47426257	47426335	ARAF	693_selected_genes
chrX	47426359	47426555	ARAF	693_selected_genes
chrX	47426603	47426856	ARAF	693_selected_genes
chrX	47428091	47428318	ARAF	693_selected_genes
chrX	47428360	47428457	ARAF	693_selected_genes
chrX	47428912	47429081	ARAF	693_selected_genes
chrX	47429266	47429448	ARAF	693_selected_genes
chrX	47430251	47430436	ARAF	693_selected_genes
chrX	47430696	47430881	ARAF	693_selected_genes
chr20	62331769	62331907	ARFRP1	693_selected_genes
chr20	62331924	62332079	ARFRP1	693_selected_genes

chr20	62332516	62332765	ARFRP1	693_selected_genes
chr20	62333156	62333277	ARFRP1	693_selected_genes
chr20	62333462	62333594	ARFRP1	693_selected_genes
chr20	62336964	62337164	ARFRP1	693_selected_genes
chr20	62337683	62337816	ARFRP1	693_selected_genes
chr20	62337977	62338115	ARFRP1	693_selected_genes
chr20	62338325	62338468	ARFRP1	693_selected_genes
chr19	47421907	47425638	ARHGAP35	693_selected_genes
chr19	47440495	47440690	ARHGAP35	693_selected_genes
chr19	47491220	47491348	ARHGAP35	693_selected_genes
chr19	47492775	47492957	ARHGAP35	693_selected_genes
chr19	47502535	47502691	ARHGAP35	693_selected_genes
chr19	47503562	47503970	ARHGAP35	693_selected_genes
chrX	135750162	135750353	ARHGEF6	693_selected_genes
chrX	135751615	135751720	ARHGEF6	693_selected_genes
chrX	135754153	135754303	ARHGEF6	693_selected_genes
chrX	135757140	135757280	ARHGEF6	693_selected_genes
chrX	135758757	135758901	ARHGEF6	693_selected_genes
chrX	135760069	135760140	ARHGEF6	693_selected_genes
chrX	135761668	135761844	ARHGEF6	693_selected_genes
chrX	135762864	135763060	ARHGEF6	693_selected_genes
chrX	135764023	135764152	ARHGEF6	693_selected_genes
chrX	135764891	135765028	ARHGEF6	693_selected_genes
chrX	135767810	135768007	ARHGEF6	693_selected_genes
chrX	135770065	135770175	ARHGEF6	693_selected_genes
chrX	135772743	135772932	ARHGEF6	693_selected_genes
chrX	135789041	135789214	ARHGEF6	693_selected_genes
chrX	135790808	135790954	ARHGEF6	693_selected_genes
chrX	135795409	135795554	ARHGEF6	693_selected_genes
chrX	135814235	135814356	ARHGEF6	693_selected_genes
chrX	135819517	135819648	ARHGEF6	693_selected_genes
chrX	135825718	135825970	ARHGEF6	693_selected_genes
chrX	135827356	135827531	ARHGEF6	693_selected_genes
chrX	135829641	135829776	ARHGEF6	693_selected_genes
chrX	135861547	135861681	ARHGEF6	693_selected_genes
chrX	135862851	135863066	ARHGEF6	693_selected_genes
chr1	27022869	27024056	ARID1A	693_selected_genes
chr1	27056116	27056379	ARID1A	693_selected_genes
chr1	27057617	27058120	ARID1A	693_selected_genes
chr1	27059141	27059308	ARID1A	693_selected_genes
chr1	27087321	27087612	ARID1A	693_selected_genes
chr1	27087849	27087989	ARID1A	693_selected_genes
chr1	27088617	27088835	ARID1A	693_selected_genes
chr1	27089438	27089801	ARID1A	693_selected_genes
chr1	27092686	27092882	ARID1A	693_selected_genes
chr1	27092922	27093082	ARID1A	693_selected_genes
chr1	27094255	27094517	ARID1A	693_selected_genes
chr1	27097584	27097842	ARID1A	693_selected_genes
chr1	27098965	27099148	ARID1A	693_selected_genes
chr1	27099277	27099503	ARID1A	693_selected_genes
chr1	27099811	27100012	ARID1A	693_selected_genes
chr1	27100045	27100233	ARID1A	693_selected_genes
chr1	27100267	27100414	ARID1A	693_selected_genes
chr1	27100794	27101736	ARID1A	693_selected_genes
chr1	27102042	27102223	ARID1A	693_selected_genes
chr1	27102282	27102416	ARID1A	693_selected_genes
chr1	27105488	27107272	ARID1A	693_selected_genes
chr6	157099038	157100630	ARID1B	693_selected_genes
chr6	157150335	157150580	ARID1B	693_selected_genes
chr6	157192722	157192811	ARID1B	693_selected_genes
chr6	157222481	157222684	ARID1B	693_selected_genes
chr6	157256574	157256735	ARID1B	693_selected_genes
chr6	157405770	157406064	ARID1B	693_selected_genes
chr6	157431580	157431720	ARID1B	693_selected_genes
chr6	157454136	157454366	ARID1B	693_selected_genes
chr6	157469732	157470110	ARID1B	693_selected_genes
chr6	157488148	157488344	ARID1B	693_selected_genes
chr6	157495116	157495276	ARID1B	693_selected_genes
chr6	157495955	157496164	ARID1B	693_selected_genes
chr6	157502077	157502337	ARID1B	693_selected_genes
chr6	157505339	157505704	ARID1B	693_selected_genes
chr6	157507463	157507764	ARID1B	693_selected_genes
chr6	157510750	157510939	ARID1B	693_selected_genes
chr6	157511146	157511369	ARID1B	693_selected_genes
chr6	157517273	157517474	ARID1B	693_selected_genes
chr6	157519919	157520066	ARID1B	693_selected_genes
chr6	157521813	157522647	ARID1B	693_selected_genes
chr6	157524974	157525155	ARID1B	693_selected_genes
chr6	157527275	157529050	ARID1B	693_selected_genes
chr12	46123594	46123736	ARID2	693_selected_genes
chr12	46123801	46123945	ARID2	693_selected_genes
chr12	46124974	46125122	ARID2	693_selected_genes
chr12	46149688	46149778	ARID2	693_selected_genes
chr12	46169838	46169904	ARID2	693_selected_genes

chr12	46205175	46205359	ARID2	693_selected_genes
chr12	46211427	46211696	ARID2	693_selected_genes
chr12	46215177	46215295	ARID2	693_selected_genes
chr12	46230346	46230463	ARID2	693_selected_genes
chr12	46230498	46230799	ARID2	693_selected_genes
chr12	46231078	46231225	ARID2	693_selected_genes
chr12	46231255	46231515	ARID2	693_selected_genes
chr12	46233086	46233304	ARID2	693_selected_genes
chr12	46240613	46240745	ARID2	693_selected_genes
chr12	46242593	46242778	ARID2	693_selected_genes
chr12	46243337	46243584	ARID2	693_selected_genes
chr12	46243793	46246704	ARID2	693_selected_genes
chr12	46254558	46254757	ARID2	693_selected_genes
chr12	46285537	46285726	ARID2	693_selected_genes
chr12	46285768	46285904	ARID2	693_selected_genes
chr12	46287177	46287351	ARID2	693_selected_genes
chr12	46287387	46287533	ARID2	693_selected_genes
chr12	46298099	46298168	ARID2	693_selected_genes
chr12	46298691	46298886	ARID2	693_selected_genes
chr10	63661443	63661514	ARID5B	693_selected_genes
chr10	63661892	63662197	ARID5B	693_selected_genes
chr10	63699916	63700192	ARID5B	693_selected_genes
chr10	63759824	63760105	ARID5B	693_selected_genes
chr10	63809213	63809267	ARID5B	693_selected_genes
chr10	63810621	63810784	ARID5B	693_selected_genes
chr10	63816850	63817102	ARID5B	693_selected_genes
chr10	63818976	63819079	ARID5B	693_selected_genes
chr10	63829433	63829581	ARID5B	693_selected_genes
chr10	63845435	63845684	ARID5B	693_selected_genes
chr10	63850595	63852814	ARID5B	693_selected_genes
chr20	30946553	30946660	ASXL1	693_selected_genes
chr20	30947524	30947619	ASXL1	693_selected_genes
chr20	30954161	30954294	ASXL1	693_selected_genes
chr20	30955504	30955557	ASXL1	693_selected_genes
chr20	30956789	30956951	ASXL1	693_selected_genes
chr20	30959555	30959611	ASXL1	693_selected_genes
chr20	30959941	30959997	ASXL1	693_selected_genes
chr20	31015905	31016076	ASXL1	693_selected_genes
chr20	31016102	31016250	ASXL1	693_selected_genes
chr20	31017115	31017259	ASXL1	693_selected_genes
chr20	31017678	31017881	ASXL1	693_selected_genes
chr20	31018920	31019004	ASXL1	693_selected_genes
chr20	31019098	31019312	ASXL1	693_selected_genes
chr20	31019360	31019507	ASXL1	693_selected_genes
chr20	31020657	31020813	ASXL1	693_selected_genes
chr20	31021061	31021745	ASXL1	693_selected_genes
chr20	31022209	31025166	ASXL1	693_selected_genes
chr2	25964872	25967370	ASXL2	693_selected_genes
chr2	25972539	25973307	ASXL2	693_selected_genes
chr2	25976377	25976533	ASXL2	693_selected_genes
chr2	25978861	25979008	ASXL2	693_selected_genes
chr2	25982325	25982539	ASXL2	693_selected_genes
chr2	25990426	25990620	ASXL2	693_selected_genes
chr2	25991585	25991762	ASXL2	693_selected_genes
chr2	25994283	25994434	ASXL2	693_selected_genes
chr2	26022228	26022429	ASXL2	693_selected_genes
chr2	26029072	26029234	ASXL2	693_selected_genes
chr2	26058381	26058434	ASXL2	693_selected_genes
chr2	26068324	26068457	ASXL2	693_selected_genes
chr2	26101009	26101116	ASXL2	693_selected_genes
chr11	108098326	108098448	ATM	693_selected_genes
chr11	108098477	108098640	ATM	693_selected_genes
chr11	108099879	108100075	ATM	693_selected_genes
chr11	108106371	108106586	ATM	693_selected_genes
chr11	108114654	108114870	ATM	693_selected_genes
chr11	108115489	108115778	ATM	693_selected_genes
chr11	108117665	108117879	ATM	693_selected_genes
chr11	108119634	108119854	ATM	693_selected_genes
chr11	108121402	108121824	ATM	693_selected_genes
chr11	108122538	108122783	ATM	693_selected_genes
chr11	108123518	108123664	ATM	693_selected_genes
chr11	108124515	108124791	ATM	693_selected_genes
chr11	108126916	108127092	ATM	693_selected_genes
chr11	108128182	108128358	ATM	693_selected_genes
chr11	108129687	108129827	ATM	693_selected_genes
chr11	108137872	108138094	ATM	693_selected_genes
chr11	108139111	108139361	ATM	693_selected_genes
chr11	108141765	108141898	ATM	693_selected_genes
chr11	108141952	108142158	ATM	693_selected_genes
chr11	108143233	108143359	ATM	693_selected_genes
chr11	108143423	108143604	ATM	693_selected_genes
chr11	108150192	108150360	ATM	693_selected_genes
chr11	108151696	108151920	ATM	693_selected_genes
chr11	108153411	108153631	ATM	693_selected_genes

chr11	108154928	108155225	ATM	693_selected_genes
chr11	108158301	108158467	ATM	693_selected_genes
chr11	108159678	108159855	ATM	693_selected_genes
chr11	108160303	108160553	ATM	693_selected_genes
chr11	108163320	108163545	ATM	693_selected_genes
chr11	108164014	108164229	ATM	693_selected_genes
chr11	108165628	108165811	ATM	693_selected_genes
chr11	108167988	108168134	ATM	693_selected_genes
chr11	108170415	108170637	ATM	693_selected_genes
chr11	108172349	108172541	ATM	693_selected_genes
chr11	108173554	108173781	ATM	693_selected_genes
chr11	108175376	108175604	ATM	693_selected_genes
chr11	108178598	108178736	ATM	693_selected_genes
chr11	108180861	108181067	ATM	693_selected_genes
chr11	108183112	108183250	ATM	693_selected_genes
chr11	108186524	108186663	ATM	693_selected_genes
chr11	108186712	108186865	ATM	693_selected_genes
chr11	108188074	108188273	ATM	693_selected_genes
chr11	108190655	108190810	ATM	693_selected_genes
chr11	108192002	108192172	ATM	693_selected_genes
chr11	108196011	108196296	ATM	693_selected_genes
chr11	108196759	108196977	ATM	693_selected_genes
chr11	108198346	108198510	ATM	693_selected_genes
chr11	108199722	108199990	ATM	693_selected_genes
chr11	108200915	108201173	ATM	693_selected_genes
chr11	108202145	108202309	ATM	693_selected_genes
chr11	108202580	108202789	ATM	693_selected_genes
chr11	108203463	108203652	ATM	693_selected_genes
chr11	108204587	108204720	ATM	693_selected_genes
chr11	108205670	108205861	ATM	693_selected_genes
chr11	108206546	108206713	ATM	693_selected_genes
chr11	108213923	108214123	ATM	693_selected_genes
chr11	108216444	108216660	ATM	693_selected_genes
chr11	108217980	108218117	ATM	693_selected_genes
chr11	108224467	108224632	ATM	693_selected_genes
chr11	108225512	108225626	ATM	693_selected_genes
chr11	108235783	108235970	ATM	693_selected_genes
chr11	108236026	108236260	ATM	693_selected_genes
chr1	116916108	116916170	ATP1A1	693_selected_genes
chr1	116916826	116916888	ATP1A1	693_selected_genes
chr1	116926610	116926771	ATP1A1	693_selected_genes
chr1	116927379	116927489	ATP1A1	693_selected_genes
chr1	116929884	116930138	ATP1A1	693_selected_genes
chr1	116930727	116930891	ATP1A1	693_selected_genes
chr1	116931234	116931419	ATP1A1	693_selected_genes
chr1	116931498	116931666	ATP1A1	693_selected_genes
chr1	116932035	116932354	ATP1A1	693_selected_genes
chr1	116932809	116933058	ATP1A1	693_selected_genes
chr1	116933378	116933538	ATP1A1	693_selected_genes
chr1	116935450	116935635	ATP1A1	693_selected_genes
chr1	116936127	116936370	ATP1A1	693_selected_genes
chr1	116937706	116937932	ATP1A1	693_selected_genes
chr1	116939194	116939381	ATP1A1	693_selected_genes
chr1	116940484	116940685	ATP1A1	693_selected_genes
chr1	116941217	116941436	ATP1A1	693_selected_genes
chr1	116941526	116941731	ATP1A1	693_selected_genes
chr1	116942016	116942190	ATP1A1	693_selected_genes
chr1	116943457	116943653	ATP1A1	693_selected_genes
chr1	116943726	116943907	ATP1A1	693_selected_genes
chr1	116944150	116944302	ATP1A1	693_selected_genes
chr1	116946480	116946622	ATP1A1	693_selected_genes
chr1	116947012	116947091	ATP1A1	693_selected_genes
chr1	116952731	116952803	ATP1A1	693_selected_genes
chr3	142168245	142168469	ATR	693_selected_genes
chr3	142169349	142169469	ATR	693_selected_genes
chr3	142171872	142172100	ATR	693_selected_genes
chr3	142176420	142176622	ATR	693_selected_genes
chr3	142177774	142177978	ATR	693_selected_genes
chr3	142178043	142178250	ATR	693_selected_genes
chr3	142180756	142180957	ATR	693_selected_genes
chr3	142183913	142184107	ATR	693_selected_genes
chr3	142185140	142185400	ATR	693_selected_genes
chr3	142186750	142186935	ATR	693_selected_genes
chr3	142188153	142188436	ATR	693_selected_genes
chr3	142188902	142189050	ATR	693_selected_genes
chr3	142203956	142204149	ATR	693_selected_genes
chr3	142211948	142212178	ATR	693_selected_genes
chr3	142215177	142215387	ATR	693_selected_genes
chr3	142215829	142216059	ATR	693_selected_genes
chr3	142217413	142217641	ATR	693_selected_genes
chr3	142217946	142218059	ATR	693_selected_genes
chr3	142218443	142218585	ATR	693_selected_genes
chr3	142222178	142222320	ATR	693_selected_genes
chr3	142223955	142224170	ATR	693_selected_genes

chr3	142226747	142226976	ATR	693_selected_genes
chr3	142231076	142231337	ATR	693_selected_genes
chr3	142232317	142232505	ATR	693_selected_genes
chr3	142234211	142234382	ATR	693_selected_genes
chr3	142238485	142238651	ATR	693_selected_genes
chr3	142241544	142241708	ATR	693_selected_genes
chr3	142242809	142243066	ATR	693_selected_genes
chr3	142253896	142254072	ATR	693_selected_genes
chr3	142254924	142255068	ATR	693_selected_genes
chr3	142257298	142257492	ATR	693_selected_genes
chr3	142259720	142259901	ATR	693_selected_genes
chr3	142261481	142261624	ATR	693_selected_genes
chr3	142266541	142266777	ATR	693_selected_genes
chr3	142268295	142268540	ATR	693_selected_genes
chr3	142268948	142269169	ATR	693_selected_genes
chr3	142272043	142272265	ATR	693_selected_genes
chr3	142272456	142272607	ATR	693_selected_genes
chr3	142272641	142272882	ATR	693_selected_genes
chr3	142274693	142275006	ATR	693_selected_genes
chr3	142275199	142275442	ATR	693_selected_genes
chr3	142277440	142277643	ATR	693_selected_genes
chr3	142278067	142278308	ATR	693_selected_genes
chr3	142279079	142279321	ATR	693_selected_genes
chr3	142280059	142280288	ATR	693_selected_genes
chr3	142281048	142281976	ATR	693_selected_genes
chr3	142284937	142285128	ATR	693_selected_genes
chr3	142286879	142287021	ATR	693_selected_genes
chr3	142297462	142297571	ATR	693_selected_genes
chrX	76763803	76764132	ATRX	693_selected_genes
chrX	76776240	76776419	ATRX	693_selected_genes
chrX	76776855	76777001	ATRX	693_selected_genes
chrX	76777715	76777891	ATRX	693_selected_genes
chrX	76778704	76778904	ATRX	693_selected_genes
chrX	76812896	76813141	ATRX	693_selected_genes
chrX	76814114	76814342	ATRX	693_selected_genes
chrX	76829689	76829848	ATRX	693_selected_genes
chrX	76845278	76845435	ATRX	693_selected_genes
chrX	76849140	76849344	ATRX	693_selected_genes
chrX	76854854	76855074	ATRX	693_selected_genes
chrX	76855175	76855314	ATRX	693_selected_genes
chrX	76855877	76856058	ATRX	693_selected_genes
chrX	76871904	76872004	ATRX	693_selected_genes
chrX	76872055	76872223	ATRX	693_selected_genes
chrX	76874248	76874474	ATRX	693_selected_genes
chrX	76875837	76876025	ATRX	693_selected_genes
chrX	76888669	76888897	ATRX	693_selected_genes
chrX	76889028	76889225	ATRX	693_selected_genes
chrX	76890059	76890219	ATRX	693_selected_genes
chrX	76891380	76891572	ATRX	693_selected_genes
chrX	76907578	76907868	ATRX	693_selected_genes
chrX	76909562	76909715	ATRX	693_selected_genes
chrX	76912024	76912168	ATRX	693_selected_genes
chrX	76918845	76919072	ATRX	693_selected_genes
chrX	76920108	76920292	ATRX	693_selected_genes
chrX	76931695	76931818	ATRX	693_selected_genes
chrX	76936986	76940110	ATRX	693_selected_genes
chrX	76940405	76940523	ATRX	693_selected_genes
chrX	76944285	76944445	ATRX	693_selected_genes
chrX	76949273	76949451	ATRX	693_selected_genes
chrX	76952039	76952217	ATRX	693_selected_genes
chrX	76953045	76953148	ATRX	693_selected_genes
chrX	76954036	76954142	ATRX	693_selected_genes
chrX	76972582	76972745	ATRX	693_selected_genes
chrX	77041442	77041512	ATRX	693_selected_genes
chr20	54945188	54945421	AURKA	693_selected_genes
chr20	54945500	54945740	AURKA	693_selected_genes
chr20	54948438	54948637	AURKA	693_selected_genes
chr20	54956463	54956652	AURKA	693_selected_genes
chr20	54958015	54958257	AURKA	693_selected_genes
chr20	54959300	54959405	AURKA	693_selected_genes
chr20	54961287	54961614	AURKA	693_selected_genes
chr20	54963186	54963278	AURKA	693_selected_genes
chr17	8108163	8108387	AURKB	693_selected_genes
chr17	8108508	8108733	AURKB	693_selected_genes
chr17	8109783	8109982	AURKB	693_selected_genes
chr17	8110032	8110277	AURKB	693_selected_genes
chr17	8110468	8110713	AURKB	693_selected_genes
chr17	8110863	8110968	AURKB	693_selected_genes
chr17	8111030	8111183	AURKB	693_selected_genes
chr17	8113469	8113567	AURKB	693_selected_genes
chr16	338096	338331	AXIN1	693_selected_genes
chr16	339414	339632	AXIN1	693_selected_genes
chr16	341164	341322	AXIN1	693_selected_genes
chr16	343462	343743	AXIN1	693_selected_genes

chr16	347030	347251	AXIN1	693_selected_genes
chr16	347696	348276	AXIN1	693_selected_genes
chr16	354278	354466	AXIN1	693_selected_genes
chr16	359947	360094	AXIN1	693_selected_genes
chr16	364517	364708	AXIN1	693_selected_genes
chr16	396122	397050	AXIN1	693_selected_genes
chr17	63526068	63526245	AXIN2	693_selected_genes
chr17	63530004	63530222	AXIN2	693_selected_genes
chr17	63531718	63531864	AXIN2	693_selected_genes
chr17	63532412	63532696	AXIN2	693_selected_genes
chr17	63532961	63533206	AXIN2	693_selected_genes
chr17	63533416	63533978	AXIN2	693_selected_genes
chr17	63534295	63534486	AXIN2	693_selected_genes
chr17	63537547	63537700	AXIN2	693_selected_genes
chr17	63545612	63545803	AXIN2	693_selected_genes
chr17	63553898	63554763	AXIN2	693_selected_genes
chr19	41725272	41725407	AXL	693_selected_genes
chr19	41726515	41726788	AXL	693_selected_genes
chr19	41727025	41727176	AXL	693_selected_genes
chr19	41727759	41727986	AXL	693_selected_genes
chr19	41736846	41736977	AXL	693_selected_genes
chr19	41737062	41737228	AXL	693_selected_genes
chr19	41743823	41744084	AXL	693_selected_genes
chr19	41744349	41744539	AXL	693_selected_genes
chr19	41745043	41745244	AXL	693_selected_genes
chr19	41745573	41745650	AXL	693_selected_genes
chr19	41748762	41748945	AXL	693_selected_genes
chr19	41749495	41749637	AXL	693_selected_genes
chr19	41754393	41754539	AXL	693_selected_genes
chr19	41754622	41754750	AXL	693_selected_genes
chr19	41758230	41758373	AXL	693_selected_genes
chr19	41758725	41758897	AXL	693_selected_genes
chr19	41759478	41759638	AXL	693_selected_genes
chr19	41762331	41762541	AXL	693_selected_genes
chr19	41763372	41763559	AXL	693_selected_genes
chr19	41765432	41765834	AXL	693_selected_genes
chr15	45003719	45003836	B2M	693_selected_genes
chr15	45007595	45007947	B2M	693_selected_genes
chr15	45008501	45008565	B2M	693_selected_genes
chr21	30693576	30693860	BACH1	693_selected_genes
chr21	30698354	30699739	BACH1	693_selected_genes
chr21	30701782	30702039	BACH1	693_selected_genes
chr21	30714694	30715179	BACH1	693_selected_genes
chr21	30954606	30954696	BACH1	693_selected_genes
chr21	30969864	30970039	BACH1	693_selected_genes
chr21	31002833	31002901	BACH1	693_selected_genes
chr3	52436278	52436462	BAP1	693_selected_genes
chr3	52436592	52436715	BAP1	693_selected_genes
chr3	52436769	52436981	BAP1	693_selected_genes
chr3	52437128	52437339	BAP1	693_selected_genes
chr3	52437406	52437935	BAP1	693_selected_genes
chr3	52438443	52438627	BAP1	693_selected_genes
chr3	52439100	52439335	BAP1	693_selected_genes
chr3	52439755	52439953	BAP1	693_selected_genes
chr3	52440243	52440417	BAP1	693_selected_genes
chr3	52440819	52440948	BAP1	693_selected_genes
chr3	52441164	52441357	BAP1	693_selected_genes
chr3	52441389	52441501	BAP1	693_selected_genes
chr3	52441948	52442118	BAP1	693_selected_genes
chr3	52442464	52442647	BAP1	693_selected_genes
chr3	52443544	52443649	BAP1	693_selected_genes
chr3	52443704	52443784	BAP1	693_selected_genes
chr3	52443832	52443919	BAP1	693_selected_genes
chr2	215593374	215593757	BARD1	693_selected_genes
chr2	215595109	215595257	BARD1	693_selected_genes
chr2	215595606	215595672	BARD1	693_selected_genes
chr2	215609765	215609908	BARD1	693_selected_genes
chr2	215610420	215610603	BARD1	693_selected_genes
chr2	215617145	215617304	BARD1	693_selected_genes
chr2	215632180	215632403	BARD1	693_selected_genes
chr2	215633930	215634061	BARD1	693_selected_genes
chr2	215645258	215646258	BARD1	693_selected_genes
chr2	215656995	215657194	BARD1	693_selected_genes
chr2	215661759	215661866	BARD1	693_selected_genes
chr2	215674110	215674318	BARD1	693_selected_genes
chr19	47724932	47725200	BBC3	693_selected_genes
chr19	47729795	47730036	BBC3	693_selected_genes
chr19	47731389	47731728	BBC3	693_selected_genes
chr19	47735746	47735884	BBC3	693_selected_genes
chr18	60795832	60796017	BCL2	693_selected_genes
chr18	60985256	60985924	BCL2	693_selected_genes
chr20	30253726	30253914	BCL2L1	693_selected_genes
chr20	30309432	30310046	BCL2L1	693_selected_genes
chr2	111881297	111881741	BCL2L11	693_selected_genes

chr2	111881869	111882023	BCL2L11	693_selected_genes
chr2	111886178	111886353	BCL2L11	693_selected_genes
chr2	111887683	111887837	BCL2L11	693_selected_genes
chr2	111899864	111899993	BCL2L11	693_selected_genes
chr2	111902039	111902103	BCL2L11	693_selected_genes
chr2	111907595	111907749	BCL2L11	693_selected_genes
chr2	111909373	111909453	BCL2L11	693_selected_genes
chr2	111911331	111911410	BCL2L11	693_selected_genes
chr2	111918970	111919041	BCL2L11	693_selected_genes
chr2	111921684	111921833	BCL2L11	693_selected_genes
chr14	23776951	23777433	BCL2L2	693_selected_genes
chr14	23777999	23778199	BCL2L2	693_selected_genes
chr14	23780156	23780332	BCL2L2	693_selected_genes
chr3	187440220	187440414	BCL6	693_selected_genes
chr3	187442703	187442891	BCL6	693_selected_genes
chr3	187443261	187443442	BCL6	693_selected_genes
chr3	187444493	187444711	BCL6	693_selected_genes
chr3	187446122	187446357	BCL6	693_selected_genes
chr3	187446812	187447834	BCL6	693_selected_genes
chr3	187449471	187449743	BCL6	693_selected_genes
chr3	187451295	187451506	BCL6	693_selected_genes
chr6	136582222	136582278	BCLAF1	693_selected_genes
chr6	136582377	136582640	BCLAF1	693_selected_genes
chr6	136588141	136588338	BCLAF1	693_selected_genes
chr6	136589274	136589502	BCLAF1	693_selected_genes
chr6	136590376	136590466	BCLAF1	693_selected_genes
chr6	136590549	136590775	BCLAF1	693_selected_genes
chr6	136593107	136593242	BCLAF1	693_selected_genes
chr6	136594194	136594350	BCLAF1	693_selected_genes
chr6	136595225	136595352	BCLAF1	693_selected_genes
chr6	136596644	136596864	BCLAF1	693_selected_genes
chr6	136596955	136597671	BCLAF1	693_selected_genes
chr6	136598977	136599939	BCLAF1	693_selected_genes
chr6	136600875	136601029	BCLAF1	693_selected_genes
chrX	39909143	39909269	BCOR	693_selected_genes
chrX	39911336	39911678	BCOR	693_selected_genes
chrX	39913109	39913320	BCOR	693_selected_genes
chrX	39913483	39913611	BCOR	693_selected_genes
chrX	39914595	39914791	BCOR	693_selected_genes
chrX	39916382	39916599	BCOR	693_selected_genes
chrX	39921366	39921671	BCOR	693_selected_genes
chrX	39921973	39922349	BCOR	693_selected_genes
chrX	39922835	39923230	BCOR	693_selected_genes
chrX	39923563	39923877	BCOR	693_selected_genes
chrX	39930200	39930437	BCOR	693_selected_genes
chrX	39930864	39930968	BCOR	693_selected_genes
chrX	39931576	39934458	BCOR	693_selected_genes
chrX	39935681	39935810	BCOR	693_selected_genes
chrX	39937071	39937207	BCOR	693_selected_genes
chrX	129139182	129139318	BCORL1	693_selected_genes
chrX	129144458	129144560	BCORL1	693_selected_genes
chrX	129146528	129146669	BCORL1	693_selected_genes
chrX	129146900	129150214	BCORL1	693_selected_genes
chrX	129154934	129155150	BCORL1	693_selected_genes
chrX	129156846	129156977	BCORL1	693_selected_genes
chrX	129158939	129159379	BCORL1	693_selected_genes
chrX	129162584	129162861	BCORL1	693_selected_genes
chrX	129168404	129168676	BCORL1	693_selected_genes
chrX	129171316	129171533	BCORL1	693_selected_genes
chrX	129173086	129173282	BCORL1	693_selected_genes
chrX	129184666	129184794	BCORL1	693_selected_genes
chrX	129185809	129186016	BCORL1	693_selected_genes
chrX	129189803	129190136	BCORL1	693_selected_genes
chr22	23523122	23524451	BCR	693_selected_genes
chr22	23584979	23585154	BCR	693_selected_genes
chr22	23595960	23596192	BCR	693_selected_genes
chr22	23603111	23603266	BCR	693_selected_genes
chr22	23603516	23603752	BCR	693_selected_genes
chr22	23610569	23610727	BCR	693_selected_genes
chr22	23613693	23613804	BCR	693_selected_genes
chr22	23615242	23615345	BCR	693_selected_genes
chr22	23615795	23615986	BCR	693_selected_genes
chr22	23626138	23626310	BCR	693_selected_genes
chr22	23627194	23627413	BCR	693_selected_genes
chr22	23629320	23629490	BCR	693_selected_genes
chr22	23630258	23630384	BCR	693_selected_genes
chr22	23631678	23631833	BCR	693_selected_genes
chr22	23632500	23632625	BCR	693_selected_genes
chr22	23634702	23634850	BCR	693_selected_genes
chr22	23637185	23637367	BCR	693_selected_genes
chr22	23651585	23651695	BCR	693_selected_genes
chr22	23652485	23652645	BCR	693_selected_genes
chr22	23653858	23654048	BCR	693_selected_genes
chr22	23655048	23655233	BCR	693_selected_genes

chr22	23656129	23656285	BCR	693_selected_genes
chr22	23656713	23656926	BCR	693_selected_genes
chr22	23657594	23657734	BCR	693_selected_genes
chr15	91290597	91290745	BLM	693_selected_genes
chr15	91292571	91293322	BLM	693_selected_genes
chr15	91294991	91295201	BLM	693_selected_genes
chr15	91298015	91298193	BLM	693_selected_genes
chr15	91303185	91303534	BLM	693_selected_genes
chr15	91303798	91304510	BLM	693_selected_genes
chr15	91306170	91306412	BLM	693_selected_genes
chr15	91308500	91308669	BLM	693_selected_genes
chr15	91310114	91310278	BLM	693_selected_genes
chr15	91312337	91312486	BLM	693_selected_genes
chr15	91312642	91312841	BLM	693_selected_genes
chr15	91326026	91326183	BLM	693_selected_genes
chr15	91328125	91328336	BLM	693_selected_genes
chr15	91333853	91334099	BLM	693_selected_genes
chr15	91337371	91337612	BLM	693_selected_genes
chr15	91341394	91341592	BLM	693_selected_genes
chr15	91346725	91346975	BLM	693_selected_genes
chr15	91347371	91347614	BLM	693_selected_genes
chr15	91352341	91352514	BLM	693_selected_genes
chr15	91354409	91354661	BLM	693_selected_genes
chr15	91358306	91358534	BLM	693_selected_genes
chr10	88635750	88635867	BMPR1A	693_selected_genes
chr10	88649793	88650006	BMPR1A	693_selected_genes
chr10	88651858	88652011	BMPR1A	693_selected_genes
chr10	88659525	88659672	BMPR1A	693_selected_genes
chr10	88659758	88659908	BMPR1A	693_selected_genes
chr10	88671971	88672166	BMPR1A	693_selected_genes
chr10	88676865	88677108	BMPR1A	693_selected_genes
chr10	88678903	88679251	BMPR1A	693_selected_genes
chr10	88681251	88681477	BMPR1A	693_selected_genes
chr10	88683107	88683288	BMPR1A	693_selected_genes
chr10	88683325	88683501	BMPR1A	693_selected_genes
chr7	140426268	140426341	BRAF	693_selected_genes
chr7	140434371	140434595	BRAF	693_selected_genes
chr7	140439586	140439771	BRAF	693_selected_genes
chr7	140447189	140447272	BRAF	693_selected_genes
chr7	140449061	140449243	BRAF	693_selected_genes
chr7	140453049	140453218	BRAF	693_selected_genes
chr7	140453961	140454058	BRAF	693_selected_genes
chr7	140476686	140476913	BRAF	693_selected_genes
chr7	140477765	140477900	BRAF	693_selected_genes
chr7	140481350	140481518	BRAF	693_selected_genes
chr7	140482795	140482982	BRAF	693_selected_genes
chr7	140487322	140487409	BRAF	693_selected_genes
chr7	140494082	140494292	BRAF	693_selected_genes
chr7	140500136	140500306	BRAF	693_selected_genes
chr7	140501186	140501385	BRAF	693_selected_genes
chr7	140507734	140507887	BRAF	693_selected_genes
chr7	140508069	140508141	BRAF	693_selected_genes
chr7	140508666	140508820	BRAF	693_selected_genes
chr7	140534383	140534697	BRAF	693_selected_genes
chr7	140549885	140550037	BRAF	693_selected_genes
chr7	140624340	140624528	BRAF	693_selected_genes
chr17	41197669	41197844	BRCA1	693_selected_genes
chr17	41199634	41199745	BRCA1	693_selected_genes
chr17	41201112	41201236	BRCA1	693_selected_genes
chr17	41202053	41202134	BRCA1	693_selected_genes
chr17	41203054	41203159	BRCA1	693_selected_genes
chr17	41209043	41209177	BRCA1	693_selected_genes
chr17	41215324	41215415	BRCA1	693_selected_genes
chr17	41215865	41215993	BRCA1	693_selected_genes
chr17	41219599	41219737	BRCA1	693_selected_genes
chr17	41222919	41223280	BRCA1	693_selected_genes
chr17	41226322	41226563	BRCA1	693_selected_genes
chr17	41228479	41228656	BRCA1	693_selected_genes
chr17	41231325	41231441	BRCA1	693_selected_genes
chr17	41234395	41234617	BRCA1	693_selected_genes
chr17	41242935	41243074	BRCA1	693_selected_genes
chr17	41243426	41246902	BRCA1	693_selected_genes
chr17	41247837	41247964	BRCA1	693_selected_genes
chr17	41249235	41249331	BRCA1	693_selected_genes
chr17	41251766	41251922	BRCA1	693_selected_genes
chr17	41256113	41256303	BRCA1	693_selected_genes
chr17	41256859	41256998	BRCA1	693_selected_genes
chr17	41258447	41258575	BRCA1	693_selected_genes
chr17	41262526	41262622	BRCA1	693_selected_genes
chr17	41267717	41267821	BRCA1	693_selected_genes
chr17	41276008	41276138	BRCA1	693_selected_genes
chr17	41277173	41277227	BRCA1	693_selected_genes
chr13	32890572	32890689	BRCA2	693_selected_genes
chr13	32893188	32893487	BRCA2	693_selected_genes

chr13	32899187	32899346	BRCA2	693_selected_genes
chr13	32900212	32900312	BRCA2	693_selected_genes
chr13	32900353	32900444	BRCA2	693_selected_genes
chr13	32900610	32900775	BRCA2	693_selected_genes
chr13	32903554	32903654	BRCA2	693_selected_genes
chr13	32905030	32905192	BRCA2	693_selected_genes
chr13	32906383	32907549	BRCA2	693_selected_genes
chr13	32910376	32915358	BRCA2	693_selected_genes
chr13	32918669	32918815	BRCA2	693_selected_genes
chr13	32920938	32921058	BRCA2	693_selected_genes
chr13	32928972	32929450	BRCA2	693_selected_genes
chr13	32930539	32930771	BRCA2	693_selected_genes
chr13	32931853	32932091	BRCA2	693_selected_genes
chr13	32936634	32936855	BRCA2	693_selected_genes
chr13	32937290	32937695	BRCA2	693_selected_genes
chr13	32944513	32944719	BRCA2	693_selected_genes
chr13	32945067	32945262	BRCA2	693_selected_genes
chr13	32949454	32949568	BRCA2	693_selected_genes
chr13	32950781	32950953	BRCA2	693_selected_genes
chr13	32953428	32953677	BRCA2	693_selected_genes
chr13	32953861	32954075	BRCA2	693_selected_genes
chr13	32954118	32954307	BRCA2	693_selected_genes
chr13	32968800	32969095	BRCA2	693_selected_genes
chr13	32970102	32970254	BRCA2	693_selected_genes
chr13	32971009	32971206	BRCA2	693_selected_genes
chr13	32972273	32972932	BRCA2	693_selected_genes
chr19	15349162	15349281	BRD4	693_selected_genes
chr19	15349528	15349816	BRD4	693_selected_genes
chr19	15349844	15350100	BRD4	693_selected_genes
chr19	15350177	15350358	BRD4	693_selected_genes
chr19	15350444	15350657	BRD4	693_selected_genes
chr19	15350695	15350858	BRD4	693_selected_genes
chr19	15353685	15354323	BRD4	693_selected_genes
chr19	15355016	15355436	BRD4	693_selected_genes
chr19	15355495	15355598	BRD4	693_selected_genes
chr19	15360065	15360126	BRD4	693_selected_genes
chr19	15364342	15364619	BRD4	693_selected_genes
chr19	15364937	15365098	BRD4	693_selected_genes
chr19	15366082	15366428	BRD4	693_selected_genes
chr19	15366849	15367099	BRD4	693_selected_genes
chr19	15367749	15368009	BRD4	693_selected_genes
chr19	15374205	15374384	BRD4	693_selected_genes
chr19	15375189	15375602	BRD4	693_selected_genes
chr19	15376139	15376479	BRD4	693_selected_genes
chr19	15378201	15378387	BRD4	693_selected_genes
chr19	15379049	15379117	BRD4	693_selected_genes
chr19	15379690	15379878	BRD4	693_selected_genes
chr19	15383600	15383935	BRD4	693_selected_genes
chr17	59760631	59761526	BRIP1	693_selected_genes
chr17	59763091	59763551	BRIP1	693_selected_genes
chr17	59770765	59770898	BRIP1	693_selected_genes
chr17	59793286	59793449	BRIP1	693_selected_genes
chr17	59816566	59816796	BRIP1	693_selected_genes
chr17	59820348	59820520	BRIP1	693_selected_genes
chr17	59821767	59821977	BRIP1	693_selected_genes
chr17	59853736	59853948	BRIP1	693_selected_genes
chr17	59857596	59857787	BRIP1	693_selected_genes
chr17	59858175	59858391	BRIP1	693_selected_genes
chr17	59861221	59861293	BRIP1	693_selected_genes
chr17	59861605	59861810	BRIP1	693_selected_genes
chr17	59870932	59871115	BRIP1	693_selected_genes
chr17	59876435	59876685	BRIP1	693_selected_genes
chr17	59878588	59878860	BRIP1	693_selected_genes
chr17	59885802	59886143	BRIP1	693_selected_genes
chr17	59924436	59924606	BRIP1	693_selected_genes
chr17	59926464	59926642	BRIP1	693_selected_genes
chr17	59934393	59934617	BRIP1	693_selected_genes
chr17	59937131	59937293	BRIP1	693_selected_genes
chr17	59938782	59938925	BRIP1	693_selected_genes
chr12	92537830	92538248	BTG1	693_selected_genes
chr12	92539138	92539336	BTG1	693_selected_genes
chrX	100604847	100604969	BTK	693_selected_genes
chrX	100608156	100608364	BTK	693_selected_genes
chrX	100608832	100609001	BTK	693_selected_genes
chrX	100609592	100609707	BTK	693_selected_genes
chrX	100611014	100611281	BTK	693_selected_genes
chrX	100611746	100611968	BTK	693_selected_genes
chrX	100612471	100612596	BTK	693_selected_genes
chrX	100613272	100613450	BTK	693_selected_genes
chrX	100613579	100613709	BTK	693_selected_genes
chrX	100614255	100614360	BTK	693_selected_genes
chrX	100615050	100615163	BTK	693_selected_genes
chrX	100615530	100615768	BTK	693_selected_genes
chrX	100617135	100617253	BTK	693_selected_genes

chrX	100617523	100617702	BTK	693_selected_genes
chrX	100624960	100625092	BTK	693_selected_genes
chrX	100626595	100626714	BTK	693_selected_genes
chrX	100629498	100629647	BTK	693_selected_genes
chrX	100630106	100630327	BTK	693_selected_genes
chrX	100645440	100645562	BTK	693_selected_genes
chr15	40453396	40453481	BUB1B	693_selected_genes
chr15	40457228	40457422	BUB1B	693_selected_genes
chr15	40458534	40458696	BUB1B	693_selected_genes
chr15	40462237	40462389	BUB1B	693_selected_genes
chr15	40462712	40462907	BUB1B	693_selected_genes
chr15	40468652	40468899	BUB1B	693_selected_genes
chr15	40475889	40476109	BUB1B	693_selected_genes
chr15	40477340	40477605	BUB1B	693_selected_genes
chr15	40477726	40477868	BUB1B	693_selected_genes
chr15	40488720	40489000	BUB1B	693_selected_genes
chr15	40491790	40491953	BUB1B	693_selected_genes
chr15	40492419	40492585	BUB1B	693_selected_genes
chr15	40493106	40493206	BUB1B	693_selected_genes
chr15	40494580	40494691	BUB1B	693_selected_genes
chr15	40494764	40494920	BUB1B	693_selected_genes
chr15	40498359	40498684	BUB1B	693_selected_genes
chr15	40500812	40500996	BUB1B	693_selected_genes
chr15	40501810	40502001	BUB1B	693_selected_genes
chr15	40502285	40502436	BUB1B	693_selected_genes
chr15	40504674	40504874	BUB1B	693_selected_genes
chr15	40505507	40505700	BUB1B	693_selected_genes
chr15	40509671	40509893	BUB1B	693_selected_genes
chr15	40510631	40510788	BUB1B	693_selected_genes
chr15	40512739	40512985	BUB1B	693_selected_genes
chr2	27440394	27440526	CAD	693_selected_genes
chr2	27440719	27440909	CAD	693_selected_genes
chr2	27444060	27444240	CAD	693_selected_genes
chr2	27445036	27445229	CAD	693_selected_genes
chr2	27445361	27445553	CAD	693_selected_genes
chr2	27445708	27445930	CAD	693_selected_genes
chr2	27446405	27446641	CAD	693_selected_genes
chr2	27446759	27446922	CAD	693_selected_genes
chr2	27447187	27447383	CAD	693_selected_genes
chr2	27447587	27447769	CAD	693_selected_genes
chr2	27447852	27448136	CAD	693_selected_genes
chr2	27448547	27448819	CAD	693_selected_genes
chr2	27448973	27449212	CAD	693_selected_genes
chr2	27449367	27449542	CAD	693_selected_genes
chr2	27449674	27449855	CAD	693_selected_genes
chr2	27454310	27454473	CAD	693_selected_genes
chr2	27454822	27455117	CAD	693_selected_genes
chr2	27455290	27455587	CAD	693_selected_genes
chr2	27455884	27456033	CAD	693_selected_genes
chr2	27456154	27456429	CAD	693_selected_genes
chr2	27456468	27456701	CAD	693_selected_genes
chr2	27456850	27457119	CAD	693_selected_genes
chr2	27457360	27457578	CAD	693_selected_genes
chr2	27458087	27458320	CAD	693_selected_genes
chr2	27458378	27458533	CAD	693_selected_genes
chr2	27459126	27459416	CAD	693_selected_genes
chr2	27459570	27459723	CAD	693_selected_genes
chr2	27460221	27460438	CAD	693_selected_genes
chr2	27460560	27460775	CAD	693_selected_genes
chr2	27460898	27461080	CAD	693_selected_genes
chr2	27461273	27461525	CAD	693_selected_genes
chr2	27461884	27462125	CAD	693_selected_genes
chr2	27462173	27462364	CAD	693_selected_genes
chr2	27462539	27462691	CAD	693_selected_genes
chr2	27463107	27463254	CAD	693_selected_genes
chr2	27463409	27463510	CAD	693_selected_genes
chr2	27463753	27463848	CAD	693_selected_genes
chr2	27463900	27464120	CAD	693_selected_genes
chr2	27464164	27464289	CAD	693_selected_genes
chr2	27464753	27465016	CAD	693_selected_genes
chr2	27465144	27465320	CAD	693_selected_genes
chr2	27465462	27465866	CAD	693_selected_genes
chr2	27466040	27466185	CAD	693_selected_genes
chr2	27466258	27466411	CAD	693_selected_genes
chr7	2946246	2946501	CARD11	693_selected_genes
chr7	2949658	2949824	CARD11	693_selected_genes
chr7	2951780	2951955	CARD11	693_selected_genes
chr7	2952895	2953125	CARD11	693_selected_genes
chr7	2954845	2955031	CARD11	693_selected_genes
chr7	2956898	2957044	CARD11	693_selected_genes
chr7	2958099	2958246	CARD11	693_selected_genes
chr7	2958980	2959271	CARD11	693_selected_genes
chr7	2962242	2962419	CARD11	693_selected_genes
chr7	2962740	2962992	CARD11	693_selected_genes

chr7	2963841	2964024	CARD11	693_selected_genes
chr7	2966347	2966450	CARD11	693_selected_genes
chr7	2968206	2968357	CARD11	693_selected_genes
chr7	2969600	2969733	CARD11	693_selected_genes
chr7	2972143	2972245	CARD11	693_selected_genes
chr7	2974061	2974288	CARD11	693_selected_genes
chr7	2976645	2976893	CARD11	693_selected_genes
chr7	2977515	2977691	CARD11	693_selected_genes
chr7	2978287	2978490	CARD11	693_selected_genes
chr7	2979357	2979587	CARD11	693_selected_genes
chr7	2983820	2984196	CARD11	693_selected_genes
chr7	2985427	2985615	CARD11	693_selected_genes
chr7	2987183	2987446	CARD11	693_selected_genes
chr7	2998108	2998165	CARD11	693_selected_genes
chr19	10982353	10982623	CARM1	693_selected_genes
chr19	11015601	11015777	CARM1	693_selected_genes
chr19	11018689	11018846	CARM1	693_selected_genes
chr19	11019200	11019283	CARM1	693_selected_genes
chr19	11019753	11019908	CARM1	693_selected_genes
chr19	11022834	11022995	CARM1	693_selected_genes
chr19	11024527	11024755	CARM1	693_selected_genes
chr19	11027057	11027198	CARM1	693_selected_genes
chr19	11027346	11027478	CARM1	693_selected_genes
chr19	11030245	11030381	CARM1	693_selected_genes
chr19	11030483	11030667	CARM1	693_selected_genes
chr19	11031086	11031274	CARM1	693_selected_genes
chr19	11031309	11031449	CARM1	693_selected_genes
chr19	11031484	11031647	CARM1	693_selected_genes
chr19	11031700	11031828	CARM1	693_selected_genes
chr19	11032025	11032144	CARM1	693_selected_genes
chr19	11032265	11032458	CARM1	693_selected_genes
chr2	202122929	202123130	CASP8	693_selected_genes
chr2	202131158	202131539	CASP8	693_selected_genes
chr2	202134207	202134353	CASP8	693_selected_genes
chr2	202136199	202136369	CASP8	693_selected_genes
chr2	202137334	202137524	CASP8	693_selected_genes
chr2	202137595	202137690	CASP8	693_selected_genes
chr2	202139586	202139701	CASP8	693_selected_genes
chr2	202140220	202140297	CASP8	693_selected_genes
chr2	202141524	202141716	CASP8	693_selected_genes
chr2	202142419	202142553	CASP8	693_selected_genes
chr2	202149513	202150065	CASP8	693_selected_genes
chr2	202151156	202151342	CASP8	693_selected_genes
chr2	202152123	202152222	CASP8	693_selected_genes
chr5	95865499	95865609	CAST	693_selected_genes
chr5	95998101	95998226	CAST	693_selected_genes
chr5	95998815	95998895	CAST	693_selected_genes
chr5	96011217	96011330	CAST	693_selected_genes
chr5	96031514	96031636	CAST	693_selected_genes
chr5	96058317	96058427	CAST	693_selected_genes
chr5	96062472	96062588	CAST	693_selected_genes
chr5	96063167	96063259	CAST	693_selected_genes
chr5	96064831	96064938	CAST	693_selected_genes
chr5	96065268	96065454	CAST	693_selected_genes
chr5	96066458	96066589	CAST	693_selected_genes
chr5	96071850	96071969	CAST	693_selected_genes
chr5	96073527	96073676	CAST	693_selected_genes
chr5	96075716	96075847	CAST	693_selected_genes
chr5	96076423	96076512	CAST	693_selected_genes
chr5	96076944	96077087	CAST	693_selected_genes
chr5	96077172	96077314	CAST	693_selected_genes
chr5	96078333	96078485	CAST	693_selected_genes
chr5	96079247	96079475	CAST	693_selected_genes
chr5	96082020	96082154	CAST	693_selected_genes
chr5	96083023	96083128	CAST	693_selected_genes
chr5	96084196	96084342	CAST	693_selected_genes
chr5	96086265	96086411	CAST	693_selected_genes
chr5	96089738	96089890	CAST	693_selected_genes
chr5	96090336	96090470	CAST	693_selected_genes
chr5	96093122	96093223	CAST	693_selected_genes
chr5	96093261	96093383	CAST	693_selected_genes
chr5	96097952	96098101	CAST	693_selected_genes
chr5	96100899	96101054	CAST	693_selected_genes
chr5	96101731	96101874	CAST	693_selected_genes
chr5	96103116	96103211	CAST	693_selected_genes
chr5	96103585	96103728	CAST	693_selected_genes
chr5	96106209	96106331	CAST	693_selected_genes
chr5	96107322	96107408	CAST	693_selected_genes
chr16	67063285	67063413	CBFB	693_selected_genes
chr16	67063604	67063741	CBFB	693_selected_genes
chr16	67070516	67070683	CBFB	693_selected_genes
chr16	67100559	67100726	CBFB	693_selected_genes
chr16	67116090	67116267	CBFB	693_selected_genes
chr16	67132587	67132706	CBFB	693_selected_genes

chr11	119077102	119077347	CBL	693_selected_genes
chr11	119103132	119103430	CBL	693_selected_genes
chr11	119142419	119142616	CBL	693_selected_genes
chr11	119144552	119144759	CBL	693_selected_genes
chr11	119145516	119145688	CBL	693_selected_genes
chr11	119146681	119146869	CBL	693_selected_genes
chr11	119148441	119148579	CBL	693_selected_genes
chr11	119148850	119149032	CBL	693_selected_genes
chr11	119149194	119149448	CBL	693_selected_genes
chr11	119155653	119155835	CBL	693_selected_genes
chr11	119155873	119156301	CBL	693_selected_genes
chr11	119158536	119158681	CBL	693_selected_genes
chr11	119167602	119167769	CBL	693_selected_genes
chr11	119168068	119168216	CBL	693_selected_genes
chr11	119169042	119169275	CBL	693_selected_genes
chr11	119170179	119170516	CBL	693_selected_genes
chr3	105377788	105378098	CBLB	693_selected_genes
chr3	105389051	105389221	CBLB	693_selected_genes
chr3	105397249	105397440	CBLB	693_selected_genes
chr3	105400292	105400479	CBLB	693_selected_genes
chr3	105400525	105400687	CBLB	693_selected_genes
chr3	105404138	105404335	CBLB	693_selected_genes
chr3	105412312	105412457	CBLB	693_selected_genes
chr3	105420912	105421328	CBLB	693_selected_genes
chr3	105422806	105423042	CBLB	693_selected_genes
chr3	105438865	105439119	CBLB	693_selected_genes
chr3	105452827	105453009	CBLB	693_selected_genes
chr3	105455989	105456127	CBLB	693_selected_genes
chr3	105459312	105459500	CBLB	693_selected_genes
chr3	105464735	105464907	CBLB	693_selected_genes
chr3	105468542	105468634	CBLB	693_selected_genes
chr3	105470280	105470487	CBLB	693_selected_genes
chr3	105495214	105495411	CBLB	693_selected_genes
chr3	105572232	105572533	CBLB	693_selected_genes
chr3	105586228	105586460	CBLB	693_selected_genes
chr3	105588155	105588257	CBLB	693_selected_genes
chr19	45281163	45281566	CBLC	693_selected_genes
chr19	45284136	45284333	CBLC	693_selected_genes
chr19	45284438	45284645	CBLC	693_selected_genes
chr19	45285601	45285773	CBLC	693_selected_genes
chr19	45287495	45287683	CBLC	693_selected_genes
chr19	45293235	45293373	CBLC	693_selected_genes
chr19	45295614	45295796	CBLC	693_selected_genes
chr19	45296705	45296902	CBLC	693_selected_genes
chr19	45297435	45297563	CBLC	693_selected_genes
chr19	45303612	45303725	CBLC	693_selected_genes
chr10	70481268	70481445	CCAR1	693_selected_genes
chr10	70482186	70482359	CCAR1	693_selected_genes
chr10	70496607	70496830	CCAR1	693_selected_genes
chr10	70497576	70497671	CCAR1	693_selected_genes
chr10	70500360	70500443	CCAR1	693_selected_genes
chr10	70502107	70502351	CCAR1	693_selected_genes
chr10	70506892	70507057	CCAR1	693_selected_genes
chr10	70507105	70507348	CCAR1	693_selected_genes
chr10	70508867	70509047	CCAR1	693_selected_genes
chr10	70509255	70509467	CCAR1	693_selected_genes
chr10	70513583	70513859	CCAR1	693_selected_genes
chr10	70514445	70514609	CCAR1	693_selected_genes
chr10	70515101	70515318	CCAR1	693_selected_genes
chr10	70516004	70516392	CCAR1	693_selected_genes
chr10	70517025	70517159	CCAR1	693_selected_genes
chr10	70520738	70520974	CCAR1	693_selected_genes
chr10	70525619	70525861	CCAR1	693_selected_genes
chr10	70530937	70531227	CCAR1	693_selected_genes
chr10	70532719	70532881	CCAR1	693_selected_genes
chr10	70545867	70546000	CCAR1	693_selected_genes
chr10	70546277	70546474	CCAR1	693_selected_genes
chr10	70547658	70547829	CCAR1	693_selected_genes
chr10	70547874	70548110	CCAR1	693_selected_genes
chr10	70549441	70549697	CCAR1	693_selected_genes
chr10	70550938	70551048	CCAR1	693_selected_genes
chr11	69456056	69456304	CCND1	693_selected_genes
chr11	69457773	69458039	CCND1	693_selected_genes
chr11	69458574	69458784	CCND1	693_selected_genes
chr11	69462736	69462935	CCND1	693_selected_genes
chr11	69465860	69466075	CCND1	693_selected_genes
chr12	4383181	4383426	CCND2	693_selected_genes
chr12	4385145	4385411	CCND2	693_selected_genes
chr12	4387900	4388110	CCND2	693_selected_genes
chr12	4397982	4398181	CCND2	693_selected_genes
chr12	4406897	4407141	CCND2	693_selected_genes
chr12	4409000	4409200	CCND2	693_selected_genes
chr6	41903563	41903870	CCND3	693_selected_genes
chr6	41904256	41904458	CCND3	693_selected_genes

chr6	41904947	41905157	CCND3	693_selected_genes
chr6	41908082	41908348	CCND3	693_selected_genes
chr6	41908664	41908762	CCND3	693_selected_genes
chr6	41909164	41909412	CCND3	693_selected_genes
chr19	30303437	30303510	CCNE1	693_selected_genes
chr19	30303570	30303708	CCNE1	693_selected_genes
chr19	30303850	30303969	CCNE1	693_selected_genes
chr19	30308018	30308214	CCNE1	693_selected_genes
chr19	30308287	30308491	CCNE1	693_selected_genes
chr19	30311583	30311780	CCNE1	693_selected_genes
chr19	30312603	30312749	CCNE1	693_selected_genes
chr19	30312877	30313062	CCNE1	693_selected_genes
chr19	30313121	30313283	CCNE1	693_selected_genes
chr19	30313327	30313535	CCNE1	693_selected_genes
chr19	30314536	30314709	CCNE1	693_selected_genes
chr1	158150866	158150977	CD1D	693_selected_genes
chr1	158151219	158151536	CD1D	693_selected_genes
chr1	158151796	158152125	CD1D	693_selected_genes
chr1	158152642	158152971	CD1D	693_selected_genes
chr1	158153700	158153850	CD1D	693_selected_genes
chr1	158153893	158153965	CD1D	693_selected_genes
chr9	5456088	5456190	CD274	693_selected_genes
chr9	5457053	5457445	CD274	693_selected_genes
chr9	5462808	5463202	CD274	693_selected_genes
chr9	5465473	5465631	CD274	693_selected_genes
chr9	5466744	5466854	CD274	693_selected_genes
chr9	5467814	5467887	CD274	693_selected_genes
chr15	73983029	73983142	CD276	693_selected_genes
chr15	73991901	73992084	CD276	693_selected_genes
chr15	73994570	73994959	CD276	693_selected_genes
chr15	73995087	73995452	CD276	693_selected_genes
chr15	73995974	73996363	CD276	693_selected_genes
chr15	73996491	73996838	CD276	693_selected_genes
chr15	74000654	74000839	CD276	693_selected_genes
chr15	74001963	74002055	CD276	693_selected_genes
chr15	74003450	74003556	CD276	693_selected_genes
chr15	74005249	74005322	CD276	693_selected_genes
chr19	6583285	6583514	CD70	693_selected_genes
chr19	6586005	6586441	CD70	693_selected_genes
chr19	6590088	6590172	CD70	693_selected_genes
chr19	6590826	6591038	CD70	693_selected_genes
chr19	42381349	42381478	CD79A	693_selected_genes
chr19	42383034	42383748	CD79A	693_selected_genes
chr19	42384711	42384830	CD79A	693_selected_genes
chr19	42384908	42385072	CD79A	693_selected_genes
chr17	62006560	62006709	CD79B	693_selected_genes
chr17	62006768	62006860	CD79B	693_selected_genes
chr17	62007104	62007273	CD79B	693_selected_genes
chr17	62007408	62007770	CD79B	693_selected_genes
chr17	62008669	62008773	CD79B	693_selected_genes
chr17	62009529	62009646	CD79B	693_selected_genes
chr17	45198275	45198408	CDC27	693_selected_genes
chr17	45199784	45200010	CDC27	693_selected_genes
chr17	45201226	45201351	CDC27	693_selected_genes
chr17	45206733	45206912	CDC27	693_selected_genes
chr17	45209597	45209765	CDC27	693_selected_genes
chr17	45214492	45214751	CDC27	693_selected_genes
chr17	45216079	45216282	CDC27	693_selected_genes
chr17	45217296	45217364	CDC27	693_selected_genes
chr17	45219193	45219416	CDC27	693_selected_genes
chr17	45219569	45219827	CDC27	693_selected_genes
chr17	45221223	45221373	CDC27	693_selected_genes
chr17	45229146	45229327	CDC27	693_selected_genes
chr17	45232012	45232255	CDC27	693_selected_genes
chr17	45234253	45234515	CDC27	693_selected_genes
chr17	45234570	45234775	CDC27	693_selected_genes
chr17	45235546	45235694	CDC27	693_selected_genes
chr17	45247257	45247433	CDC27	693_selected_genes
chr17	45249257	45249455	CDC27	693_selected_genes
chr17	45258902	45259028	CDC27	693_selected_genes
chr17	45266486	45266563	CDC27	693_selected_genes
chr1	193091305	193091486	CDC73	693_selected_genes
chr1	193094216	193094372	CDC73	693_selected_genes
chr1	193099278	193099398	CDC73	693_selected_genes
chr1	193104495	193104608	CDC73	693_selected_genes
chr1	193104641	193104744	CDC73	693_selected_genes
chr1	193107189	193107328	CDC73	693_selected_genes
chr1	193110954	193111221	CDC73	693_selected_genes
chr1	193116971	193117120	CDC73	693_selected_genes
chr1	193119408	193119537	CDC73	693_selected_genes
chr1	193121484	193121599	CDC73	693_selected_genes
chr1	193172899	193173007	CDC73	693_selected_genes
chr1	193181169	193181255	CDC73	693_selected_genes
chr1	193181494	193181632	CDC73	693_selected_genes

chr1	193202097	193202309	CDC73	693_selected_genes
chr1	193205360	193205511	CDC73	693_selected_genes
chr1	193218834	193219026	CDC73	693_selected_genes
chr1	193219780	193219867	CDC73	693_selected_genes
chr16	68771293	68771391	CDH1	693_selected_genes
chr16	68772174	68772339	CDH1	693_selected_genes
chr16	68835547	68835821	CDH1	693_selected_genes
chr16	68842301	68842495	CDH1	693_selected_genes
chr16	68842570	68842776	CDH1	693_selected_genes
chr16	68844074	68844269	CDH1	693_selected_genes
chr16	68845561	68845787	CDH1	693_selected_genes
chr16	68846012	68846191	CDH1	693_selected_genes
chr16	68847190	68847490	CDH1	693_selected_genes
chr16	68849392	68849687	CDH1	693_selected_genes
chr16	68853157	68853353	CDH1	693_selected_genes
chr16	68855878	68856153	CDH1	693_selected_genes
chr16	68857276	68857554	CDH1	693_selected_genes
chr16	68862051	68862232	CDH1	693_selected_genes
chr16	68863531	68863725	CDH1	693_selected_genes
chr16	68867167	68867427	CDH1	693_selected_genes
chr18	59157761	59158057	CDH20	693_selected_genes
chr18	59166393	59166738	CDH20	693_selected_genes
chr18	59167590	59167760	CDH20	693_selected_genes
chr18	59170160	59170378	CDH20	693_selected_genes
chr18	59174580	59174818	CDH20	693_selected_genes
chr18	59195174	59195478	CDH20	693_selected_genes
chr18	59203700	59203887	CDH20	693_selected_genes
chr18	59206231	59206403	CDH20	693_selected_genes
chr18	59212234	59212402	CDH20	693_selected_genes
chr18	59217185	59217487	CDH20	693_selected_genes
chr18	59221397	59221953	CDH20	693_selected_genes
chr17	37618299	37619395	CDK12	693_selected_genes
chr17	37627106	37628041	CDK12	693_selected_genes
chr17	37646784	37647011	CDK12	693_selected_genes
chr17	37648978	37649168	CDK12	693_selected_genes
chr17	37650751	37650972	CDK12	693_selected_genes
chr17	37657477	37657717	CDK12	693_selected_genes
chr17	37665932	37666039	CDK12	693_selected_genes
chr17	37667756	37667908	CDK12	693_selected_genes
chr17	37671958	37672086	CDK12	693_selected_genes
chr17	37673667	37673834	CDK12	693_selected_genes
chr17	37676183	37676365	CDK12	693_selected_genes
chr17	37680901	37681163	CDK12	693_selected_genes
chr17	37682091	37682594	CDK12	693_selected_genes
chr17	37686831	37687594	CDK12	693_selected_genes
chr17	37700476	37700555	CDK12	693_selected_genes
chr12	56360767	56360937	CDK2	693_selected_genes
chr12	56361615	56361743	CDK2	693_selected_genes
chr12	56361807	56361978	CDK2	693_selected_genes
chr12	56362536	56362757	CDK2	693_selected_genes
chr12	56363233	56363385	CDK2	693_selected_genes
chr12	56364356	56364550	CDK2	693_selected_genes
chr12	56364802	56365056	CDK2	693_selected_genes
chr12	56365279	56365434	CDK2	693_selected_genes
chr12	58142282	58142425	CDK4	693_selected_genes
chr12	58142939	58143125	CDK4	693_selected_genes
chr12	58143211	58143312	CDK4	693_selected_genes
chr12	58144237	58144371	CDK4	693_selected_genes
chr12	58144413	58144573	CDK4	693_selected_genes
chr12	58144680	58144898	CDK4	693_selected_genes
chr12	58144964	58145150	CDK4	693_selected_genes
chr12	58145257	58145525	CDK4	693_selected_genes
chr7	92244428	92244625	CDK6	693_selected_genes
chr7	92247360	92247546	CDK6	693_selected_genes
chr7	92252324	92252425	CDK6	693_selected_genes
chr7	92300714	92300874	CDK6	693_selected_genes
chr7	92354914	92355132	CDK6	693_selected_genes
chr7	92403984	92404170	CDK6	693_selected_genes
chr7	92462379	92462662	CDK6	693_selected_genes
chr13	26828753	26828931	CDK8	693_selected_genes
chr13	26911678	26911804	CDK8	693_selected_genes
chr13	26923183	26923344	CDK8	693_selected_genes
chr13	26927851	26928042	CDK8	693_selected_genes
chr13	26956925	26957033	CDK8	693_selected_genes
chr13	26959322	26959504	CDK8	693_selected_genes
chr13	26967478	26967672	CDK8	693_selected_genes
chr13	26970396	26970516	CDK8	693_selected_genes
chr13	26971264	26971387	CDK8	693_selected_genes
chr13	26974564	26974712	CDK8	693_selected_genes
chr13	26975380	26975509	CDK8	693_selected_genes
chr13	26975577	26975786	CDK8	693_selected_genes
chr13	26978067	26978243	CDK8	693_selected_genes
chr6	36645588	36645735	CDKN1A	693_selected_genes
chr6	36651848	36652348	CDKN1A	693_selected_genes

chr6	36653502	36653602	CDKN1A	693_selected_genes
chr12	12870748	12871273	CDKN1B	693_selected_genes
chr12	12871733	12871905	CDKN1B	693_selected_genes
chr12	12873973	12874166	CDKN1B	693_selected_genes
chr9	21968182	21968266	CDKN2A	693_selected_genes
chr9	21968698	21968795	CDKN2A	693_selected_genes
chr9	21969705	21969817	CDKN2A	693_selected_genes
chr9	21970864	21971232	CDKN2A	693_selected_genes
chr9	21974450	21974851	CDKN2A	693_selected_genes
chr9	21994112	21994478	CDKN2A	693_selected_genes
chr9	22005960	22006271	CDKN2B	693_selected_genes
chr9	22008690	22008977	CDKN2B	693_selected_genes
chr1	51436015	51436194	CDKN2C	693_selected_genes
chr1	51439539	51439967	CDKN2C	693_selected_genes
chr19	33792218	33793450	CEBPA	693_selected_genes
chr17	7788099	7788426	CHD3	693_selected_genes
chr17	7792293	7792443	CHD3	693_selected_genes
chr17	7792956	7793119	CHD3	693_selected_genes
chr17	7793863	7794084	CHD3	693_selected_genes
chr17	7794232	7794407	CHD3	693_selected_genes
chr17	7796578	7796912	CHD3	693_selected_genes
chr17	7797097	7797278	CHD3	693_selected_genes
chr17	7797407	7797608	CHD3	693_selected_genes
chr17	7797707	7797951	CHD3	693_selected_genes
chr17	7798209	7798493	CHD3	693_selected_genes
chr17	7798631	7798885	CHD3	693_selected_genes
chr17	7800375	7800637	CHD3	693_selected_genes
chr17	7801263	7801445	CHD3	693_selected_genes
chr17	7801788	7801938	CHD3	693_selected_genes
chr17	7802303	7802545	CHD3	693_selected_genes
chr17	7802635	7802886	CHD3	693_selected_genes
chr17	7803188	7803376	CHD3	693_selected_genes
chr17	7803582	7803754	CHD3	693_selected_genes
chr17	7803850	7804074	CHD3	693_selected_genes
chr17	7804144	7804336	CHD3	693_selected_genes
chr17	7804536	7804718	CHD3	693_selected_genes
chr17	7805902	7806070	CHD3	693_selected_genes
chr17	7806229	7806404	CHD3	693_selected_genes
chr17	7806564	7806846	CHD3	693_selected_genes
chr17	7807117	7807334	CHD3	693_selected_genes
chr17	7807421	7807519	CHD3	693_selected_genes
chr17	7807734	7807962	CHD3	693_selected_genes
chr17	7808392	7808508	CHD3	693_selected_genes
chr17	7808913	7809049	CHD3	693_selected_genes
chr17	7809148	7809332	CHD3	693_selected_genes
chr17	7809845	7810040	CHD3	693_selected_genes
chr17	7810161	7810374	CHD3	693_selected_genes
chr17	7810418	7810590	CHD3	693_selected_genes
chr17	7810645	7810831	CHD3	693_selected_genes
chr17	7810893	7811045	CHD3	693_selected_genes
chr17	7811183	7811362	CHD3	693_selected_genes
chr17	7811694	7811853	CHD3	693_selected_genes
chr17	7811989	7812172	CHD3	693_selected_genes
chr17	7812435	7812727	CHD3	693_selected_genes
chr17	7813720	7813934	CHD3	693_selected_genes
chr17	7814139	7814316	CHD3	693_selected_genes
chr17	7814756	7815047	CHD3	693_selected_genes
chr12	6679816	6679884	CHD4	693_selected_genes
chr12	6680009	6680223	CHD4	693_selected_genes
chr12	6682214	6682460	CHD4	693_selected_genes
chr12	6686925	6687108	CHD4	693_selected_genes
chr12	6687169	6687328	CHD4	693_selected_genes
chr12	6687549	6687737	CHD4	693_selected_genes
chr12	6687986	6688108	CHD4	693_selected_genes
chr12	6690184	6690364	CHD4	693_selected_genes
chr12	6690431	6690579	CHD4	693_selected_genes
chr12	6690789	6691005	CHD4	693_selected_genes
chr12	6691277	6691472	CHD4	693_selected_genes
chr12	6691755	6691939	CHD4	693_selected_genes
chr12	6691988	6692127	CHD4	693_selected_genes
chr12	6692167	6692569	CHD4	693_selected_genes
chr12	6696524	6696750	CHD4	693_selected_genes
chr12	6696852	6697140	CHD4	693_selected_genes
chr12	6697438	6697613	CHD4	693_selected_genes
chr12	6700606	6700774	CHD4	693_selected_genes
chr12	6700834	6701016	CHD4	693_selected_genes
chr12	6701056	6701248	CHD4	693_selected_genes
chr12	6701533	6701757	CHD4	693_selected_genes
chr12	6701836	6702008	CHD4	693_selected_genes
chr12	6702231	6702419	CHD4	693_selected_genes
chr12	6702556	6702807	CHD4	693_selected_genes
chr12	6703599	6703841	CHD4	693_selected_genes
chr12	6704474	6704621	CHD4	693_selected_genes
chr12	6705146	6705328	CHD4	693_selected_genes

chr12	6707034	6707290	CHD4	693_selected_genes
chr12	6707362	6707616	CHD4	693_selected_genes
chr12	6708913	6709203	CHD4	693_selected_genes
chr12	6709357	6709586	CHD4	693_selected_genes
chr12	6709674	6709860	CHD4	693_selected_genes
chr12	6710066	6710244	CHD4	693_selected_genes
chr12	6710429	6710721	CHD4	693_selected_genes
chr12	6710788	6710957	CHD4	693_selected_genes
chr12	6711100	6711366	CHD4	693_selected_genes
chr12	6711516	6711688	CHD4	693_selected_genes
chr12	6715414	6715564	CHD4	693_selected_genes
chr14	21853746	21854360	CHD8	693_selected_genes
chr14	21859080	21859247	CHD8	693_selected_genes
chr14	21859596	21859826	CHD8	693_selected_genes
chr14	21859966	21860130	CHD8	693_selected_genes
chr14	21860640	21860993	CHD8	693_selected_genes
chr14	21861239	21861438	CHD8	693_selected_genes
chr14	21861609	21862379	CHD8	693_selected_genes
chr14	21862410	21862669	CHD8	693_selected_genes
chr14	21863045	21863303	CHD8	693_selected_genes
chr14	21863431	21863536	CHD8	693_selected_genes
chr14	21863950	21864076	CHD8	693_selected_genes
chr14	21865956	21866136	CHD8	693_selected_genes
chr14	21867735	21867889	CHD8	693_selected_genes
chr14	21868114	21868254	CHD8	693_selected_genes
chr14	21868284	21868515	CHD8	693_selected_genes
chr14	21868546	21868796	CHD8	693_selected_genes
chr14	21869008	21869255	CHD8	693_selected_genes
chr14	21869536	21869697	CHD8	693_selected_genes
chr14	21870090	21870320	CHD8	693_selected_genes
chr14	21870469	21870687	CHD8	693_selected_genes
chr14	21871150	21871396	CHD8	693_selected_genes
chr14	21871586	21871847	CHD8	693_selected_genes
chr14	21873342	21873648	CHD8	693_selected_genes
chr14	21873854	21874048	CHD8	693_selected_genes
chr14	21874989	21875216	CHD8	693_selected_genes
chr14	21876445	21876739	CHD8	693_selected_genes
chr14	21876837	21877009	CHD8	693_selected_genes
chr14	21877984	21878172	CHD8	693_selected_genes
chr14	21881046	21881180	CHD8	693_selected_genes
chr14	21882434	21882602	CHD8	693_selected_genes
chr14	21883071	21883177	CHD8	693_selected_genes
chr14	21883707	21883826	CHD8	693_selected_genes
chr14	21883858	21884091	CHD8	693_selected_genes
chr14	21894261	21894426	CHD8	693_selected_genes
chr14	21896002	21896438	CHD8	693_selected_genes
chr14	21897097	21897519	CHD8	693_selected_genes
chr14	21898934	21899827	CHD8	693_selected_genes
chr14	21899944	21900000	CHD8	693_selected_genes
chr14	21905034	21905180	CHD8	693_selected_genes
chr11	125495630	125495932	CHEK1	693_selected_genes
chr11	125496618	125496753	CHEK1	693_selected_genes
chr11	125497476	125497750	CHEK1	693_selected_genes
chr11	125499101	125499216	CHEK1	693_selected_genes
chr11	125499260	125499380	CHEK1	693_selected_genes
chr11	125503032	125503271	CHEK1	693_selected_genes
chr11	125505298	125505453	CHEK1	693_selected_genes
chr11	125507318	125507465	CHEK1	693_selected_genes
chr11	125513661	125513820	CHEK1	693_selected_genes
chr11	125513960	125514188	CHEK1	693_selected_genes
chr11	125514381	125514563	CHEK1	693_selected_genes
chr11	125523615	125523767	CHEK1	693_selected_genes
chr11	125525094	125525240	CHEK1	693_selected_genes
chr22	29083859	29083999	CHEK2	693_selected_genes
chr22	29085097	29085279	CHEK2	693_selected_genes
chr22	29089994	29090130	CHEK2	693_selected_genes
chr22	29091089	29091255	CHEK2	693_selected_genes
chr22	29091672	29091886	CHEK2	693_selected_genes
chr22	29092860	29093000	CHEK2	693_selected_genes
chr22	29095800	29095950	CHEK2	693_selected_genes
chr22	29099467	29099579	CHEK2	693_selected_genes
chr22	29105968	29106072	CHEK2	693_selected_genes
chr22	29107871	29108030	CHEK2	693_selected_genes
chr22	29115357	29115498	CHEK2	693_selected_genes
chr22	29117562	29117644	CHEK2	693_selected_genes
chr22	29120844	29120936	CHEK2	693_selected_genes
chr22	29120939	29121137	CHEK2	693_selected_genes
chr22	29121205	29121380	CHEK2	693_selected_genes
chr22	29125283	29125389	CHEK2	693_selected_genes
chr22	29126382	29126561	CHEK2	693_selected_genes
chr22	29130365	29130740	CHEK2	693_selected_genes
chr22	29133222	29133296	CHEK2	693_selected_genes
chr10	101949329	101949409	CHUK	693_selected_genes
chr10	101950600	101950750	CHUK	693_selected_genes

chr10	101953029	101953213	CHUK	693_selected_genes
chr10	101953715	101953913	CHUK	693_selected_genes
chr10	101954156	101954303	CHUK	693_selected_genes
chr10	101959702	101959802	CHUK	693_selected_genes
chr10	101960402	101960562	CHUK	693_selected_genes
chr10	101961819	101961931	CHUK	693_selected_genes
chr10	101964237	101964439	CHUK	693_selected_genes
chr10	101964807	101964981	CHUK	693_selected_genes
chr10	101966961	101967114	CHUK	693_selected_genes
chr10	101969326	101969571	CHUK	693_selected_genes
chr10	101977726	101977912	CHUK	693_selected_genes
chr10	101978449	101978607	CHUK	693_selected_genes
chr10	101978739	101978914	CHUK	693_selected_genes
chr10	101979001	101979141	CHUK	693_selected_genes
chr10	101980319	101980458	CHUK	693_selected_genes
chr10	101981843	101981963	CHUK	693_selected_genes
chr10	101982597	101982762	CHUK	693_selected_genes
chr10	101985654	101985799	CHUK	693_selected_genes
chr10	101989159	101989314	CHUK	693_selected_genes
chr19	42775910	42778754	CIC	693_selected_genes
chr19	42788831	42788948	CIC	693_selected_genes
chr19	42790897	42791097	CIC	693_selected_genes
chr19	42791132	42791417	CIC	693_selected_genes
chr19	42791446	42791626	CIC	693_selected_genes
chr19	42791671	42791904	CIC	693_selected_genes
chr19	42791936	42792152	CIC	693_selected_genes
chr19	42793014	42793267	CIC	693_selected_genes
chr19	42793307	42793583	CIC	693_selected_genes
chr19	42793974	42794128	CIC	693_selected_genes
chr19	42794359	42795643	CIC	693_selected_genes
chr19	42795684	42795922	CIC	693_selected_genes
chr19	42796212	42796384	CIC	693_selected_genes
chr19	42796426	42796643	CIC	693_selected_genes
chr19	42796692	42797036	CIC	693_selected_genes
chr19	42797082	42797458	CIC	693_selected_genes
chr19	42797718	42798013	CIC	693_selected_genes
chr19	42798061	42798266	CIC	693_selected_genes
chr19	42798299	42798481	CIC	693_selected_genes
chr19	42798701	42798912	CIC	693_selected_genes
chr19	42798950	42799368	CIC	693_selected_genes
chr17	57697467	57697559	CLTC	693_selected_genes
chr17	57721611	57721881	CLTC	693_selected_genes
chr17	57724733	57725052	CLTC	693_selected_genes
chr17	57725575	57725787	CLTC	693_selected_genes
chr17	57728538	57728702	CLTC	693_selected_genes
chr17	57733189	57733413	CLTC	693_selected_genes
chr17	57735997	57736053	CLTC	693_selected_genes
chr17	57737726	57737974	CLTC	693_selected_genes
chr17	57738778	57739029	CLTC	693_selected_genes
chr17	57741177	57741380	CLTC	693_selected_genes
chr17	57742122	57742295	CLTC	693_selected_genes
chr17	57743438	57743626	CLTC	693_selected_genes
chr17	57743815	57744030	CLTC	693_selected_genes
chr17	57744132	57744363	CLTC	693_selected_genes
chr17	57746112	57746326	CLTC	693_selected_genes
chr17	57750982	57751158	CLTC	693_selected_genes
chr17	57752036	57752229	CLTC	693_selected_genes
chr17	57754289	57754574	CLTC	693_selected_genes
chr17	57756732	57756905	CLTC	693_selected_genes
chr17	57758247	57758443	CLTC	693_selected_genes
chr17	57758630	57758864	CLTC	693_selected_genes
chr17	57758982	57759225	CLTC	693_selected_genes
chr17	57759606	57759814	CLTC	693_selected_genes
chr17	57759964	57760179	CLTC	693_selected_genes
chr17	57760242	57760400	CLTC	693_selected_genes
chr17	57760430	57760648	CLTC	693_selected_genes
chr17	57760722	57760922	CLTC	693_selected_genes
chr17	57760960	57761142	CLTC	693_selected_genes
chr17	57761210	57761371	CLTC	693_selected_genes
chr17	57762391	57762612	CLTC	693_selected_genes
chr17	57762922	57763194	CLTC	693_selected_genes
chr17	57764336	57764449	CLTC	693_selected_genes
chr17	57767971	57768114	CLTC	693_selected_genes
chr17	57771063	57771238	CLTC	693_selected_genes
chr16	58554836	58554965	CNOT1	693_selected_genes
chr16	58555061	58555246	CNOT1	693_selected_genes
chr16	58557248	58557431	CNOT1	693_selected_genes
chr16	58559057	58559288	CNOT1	693_selected_genes
chr16	58559867	58560067	CNOT1	693_selected_genes
chr16	58561969	58562034	CNOT1	693_selected_genes
chr16	58562353	58562577	CNOT1	693_selected_genes
chr16	58564124	58564276	CNOT1	693_selected_genes
chr16	58565836	58566004	CNOT1	693_selected_genes
chr16	58566110	58566324	CNOT1	693_selected_genes

chr16	58568025	58568324	CNOT1	693_selected_genes
chr16	58570846	58571149	CNOT1	693_selected_genes
chr16	58571769	58571989	CNOT1	693_selected_genes
chr16	58572036	58572195	CNOT1	693_selected_genes
chr16	58572650	58572843	CNOT1	693_selected_genes
chr16	58573646	58573888	CNOT1	693_selected_genes
chr16	58575379	58575549	CNOT1	693_selected_genes
chr16	58576109	58576264	CNOT1	693_selected_genes
chr16	58576306	58576497	CNOT1	693_selected_genes
chr16	58577162	58577221	CNOT1	693_selected_genes
chr16	58577263	58577832	CNOT1	693_selected_genes
chr16	58579239	58579420	CNOT1	693_selected_genes
chr16	58580199	58580427	CNOT1	693_selected_genes
chr16	58580550	58580678	CNOT1	693_selected_genes
chr16	58581064	58581225	CNOT1	693_selected_genes
chr16	58581444	58581611	CNOT1	693_selected_genes
chr16	58583597	58583827	CNOT1	693_selected_genes
chr16	58585010	58585201	CNOT1	693_selected_genes
chr16	58585467	58585748	CNOT1	693_selected_genes
chr16	58587660	58587789	CNOT1	693_selected_genes
chr16	58589129	58589466	CNOT1	693_selected_genes
chr16	58589662	58589837	CNOT1	693_selected_genes
chr16	58590725	58590922	CNOT1	693_selected_genes
chr16	58592351	58592603	CNOT1	693_selected_genes
chr16	58594090	58594291	CNOT1	693_selected_genes
chr16	58608487	58608689	CNOT1	693_selected_genes
chr16	58608885	58609058	CNOT1	693_selected_genes
chr16	58610341	58610511	CNOT1	693_selected_genes
chr16	58612577	58612868	CNOT1	693_selected_genes
chr16	58614511	58614689	CNOT1	693_selected_genes
chr16	58615223	58615444	CNOT1	693_selected_genes
chr16	58616671	58616832	CNOT1	693_selected_genes
chr16	58616934	58617111	CNOT1	693_selected_genes
chr16	58619216	58619435	CNOT1	693_selected_genes
chr16	58620423	58620677	CNOT1	693_selected_genes
chr16	58621079	58621184	CNOT1	693_selected_genes
chr16	58621223	58621342	CNOT1	693_selected_genes
chr16	58621658	58621807	CNOT1	693_selected_genes
chr16	58622677	58622835	CNOT1	693_selected_genes
chr16	58633114	58633266	CNOT1	693_selected_genes
chr17	40834822	40834939	CNTNAP1	693_selected_genes
chr17	40835813	40835965	CNTNAP1	693_selected_genes
chr17	40836028	40836272	CNTNAP1	693_selected_genes
chr17	40836983	40837181	CNTNAP1	693_selected_genes
chr17	40837209	40837463	CNTNAP1	693_selected_genes
chr17	40837949	40838184	CNTNAP1	693_selected_genes
chr17	40838895	40839089	CNTNAP1	693_selected_genes
chr17	40839712	40840024	CNTNAP1	693_selected_genes
chr17	40840454	40840654	CNTNAP1	693_selected_genes
chr17	40840868	40841090	CNTNAP1	693_selected_genes
chr17	40841513	40841670	CNTNAP1	693_selected_genes
chr17	40842080	40842250	CNTNAP1	693_selected_genes
chr17	40842731	40842985	CNTNAP1	693_selected_genes
chr17	40843129	40843336	CNTNAP1	693_selected_genes
chr17	40843376	40843554	CNTNAP1	693_selected_genes
chr17	40843798	40844034	CNTNAP1	693_selected_genes
chr17	40844491	40844763	CNTNAP1	693_selected_genes
chr17	40845289	40845579	CNTNAP1	693_selected_genes
chr17	40847513	40847917	CNTNAP1	693_selected_genes
chr17	40848017	40848195	CNTNAP1	693_selected_genes
chr17	40849266	40849410	CNTNAP1	693_selected_genes
chr17	40849546	40849842	CNTNAP1	693_selected_genes
chr17	40849895	40849993	CNTNAP1	693_selected_genes
chr17	40850610	40850953	CNTNAP1	693_selected_genes
chr17	48262837	48263034	COL1A1	693_selected_genes
chr17	48263113	48263406	COL1A1	693_selected_genes
chr17	48263652	48263893	COL1A1	693_selected_genes
chr17	48263975	48264308	COL1A1	693_selected_genes
chr17	48264350	48264508	COL1A1	693_selected_genes
chr17	48264819	48264923	COL1A1	693_selected_genes
chr17	48265211	48265369	COL1A1	693_selected_genes
chr17	48265431	48265535	COL1A1	693_selected_genes
chr17	48265865	48266023	COL1A1	693_selected_genes
chr17	48266077	48266181	COL1A1	693_selected_genes
chr17	48266238	48266396	COL1A1	693_selected_genes
chr17	48266503	48266661	COL1A1	693_selected_genes
chr17	48266712	48266924	COL1A1	693_selected_genes
chr17	48267014	48267118	COL1A1	693_selected_genes
chr17	48267194	48267298	COL1A1	693_selected_genes
chr17	48267336	48267494	COL1A1	693_selected_genes
chr17	48267662	48267766	COL1A1	693_selected_genes
chr17	48267878	48267982	COL1A1	693_selected_genes
chr17	48268152	48268310	COL1A1	693_selected_genes
chr17	48268718	48268876	COL1A1	693_selected_genes

chr17	48269123	48269272	COL1A1	693_selected_genes
chr17	48269315	48269410	COL1A1	693_selected_genes
chr17	48269810	48269914	COL1A1	693_selected_genes
chr17	48269975	48270079	COL1A1	693_selected_genes
chr17	48270132	48270236	COL1A1	693_selected_genes
chr17	48270329	48270433	COL1A1	693_selected_genes
chr17	48271278	48271427	COL1A1	693_selected_genes
chr17	48271465	48271569	COL1A1	693_selected_genes
chr17	48271684	48271833	COL1A1	693_selected_genes
chr17	48271908	48272012	COL1A1	693_selected_genes
chr17	48272056	48272214	COL1A1	693_selected_genes
chr17	48272382	48272486	COL1A1	693_selected_genes
chr17	48272567	48272716	COL1A1	693_selected_genes
chr17	48272769	48272864	COL1A1	693_selected_genes
chr17	48272902	48273051	COL1A1	693_selected_genes
chr17	48273258	48273362	COL1A1	693_selected_genes
chr17	48273490	48273585	COL1A1	693_selected_genes
chr17	48273649	48273753	COL1A1	693_selected_genes
chr17	48273819	48273914	COL1A1	693_selected_genes
chr17	48273952	48274056	COL1A1	693_selected_genes
chr17	48274345	48274449	COL1A1	693_selected_genes
chr17	48274515	48274619	COL1A1	693_selected_genes
chr17	48275067	48275171	COL1A1	693_selected_genes
chr17	48275284	48275388	COL1A1	693_selected_genes
chr17	48275496	48275591	COL1A1	693_selected_genes
chr17	48275768	48275890	COL1A1	693_selected_genes
chr17	48276561	48276713	COL1A1	693_selected_genes
chr17	48276753	48276839	COL1A1	693_selected_genes
chr17	48276891	48276976	COL1A1	693_selected_genes
chr17	48277088	48277490	COL1A1	693_selected_genes
chr17	48278746	48278899	COL1A1	693_selected_genes
chr2	189839190	189839319	COL3A1	693_selected_genes
chr2	189849460	189849713	COL3A1	693_selected_genes
chr2	189849897	189849998	COL3A1	693_selected_genes
chr2	189850365	189850529	COL3A1	693_selected_genes
chr2	189851759	189851890	COL3A1	693_selected_genes
chr2	189852781	189852885	COL3A1	693_selected_genes
chr2	189853290	189853394	COL3A1	693_selected_genes
chr2	189854096	189854200	COL3A1	693_selected_genes
chr2	189854796	189854900	COL3A1	693_selected_genes
chr2	189855007	189855111	COL3A1	693_selected_genes
chr2	189855704	189855808	COL3A1	693_selected_genes
chr2	189856187	189856282	COL3A1	693_selected_genes
chr2	189856369	189856473	COL3A1	693_selected_genes
chr2	189856884	189856979	COL3A1	693_selected_genes
chr2	189857587	189857691	COL3A1	693_selected_genes
chr2	189858061	189858210	COL3A1	693_selected_genes
chr2	189858738	189858833	COL3A1	693_selected_genes
chr2	189858934	189859083	COL3A1	693_selected_genes
chr2	189859241	189859345	COL3A1	693_selected_genes
chr2	189859424	189859582	COL3A1	693_selected_genes
chr2	189859746	189859850	COL3A1	693_selected_genes
chr2	189860392	189860541	COL3A1	693_selected_genes
chr2	189860825	189860929	COL3A1	693_selected_genes
chr2	189861098	189861247	COL3A1	693_selected_genes
chr2	189861865	189861969	COL3A1	693_selected_genes
chr2	189862036	189862140	COL3A1	693_selected_genes
chr2	189862400	189862504	COL3A1	693_selected_genes
chr2	189862966	189863070	COL3A1	693_selected_genes
chr2	189863374	189863469	COL3A1	693_selected_genes
chr2	189863985	189864134	COL3A1	693_selected_genes
chr2	189864170	189864328	COL3A1	693_selected_genes
chr2	189864542	189864646	COL3A1	693_selected_genes
chr2	189866097	189866201	COL3A1	693_selected_genes
chr2	189866236	189866340	COL3A1	693_selected_genes
chr2	189866998	189867102	COL3A1	693_selected_genes
chr2	189867655	189867813	COL3A1	693_selected_genes
chr2	189868111	189868215	COL3A1	693_selected_genes
chr2	189868434	189868538	COL3A1	693_selected_genes
chr2	189868682	189868894	COL3A1	693_selected_genes
chr2	189868957	189869115	COL3A1	693_selected_genes
chr2	189870050	189870208	COL3A1	693_selected_genes
chr2	189870325	189870380	COL3A1	693_selected_genes
chr2	189870906	189871010	COL3A1	693_selected_genes
chr2	189871045	189871203	COL3A1	693_selected_genes
chr2	189871637	189871741	COL3A1	693_selected_genes
chr2	189872200	189872358	COL3A1	693_selected_genes
chr2	189872585	189872689	COL3A1	693_selected_genes
chr2	189872735	189872893	COL3A1	693_selected_genes
chr2	189873624	189873972	COL3A1	693_selected_genes
chr2	189874878	189875116	COL3A1	693_selected_genes
chr2	189875348	189875641	COL3A1	693_selected_genes
chr2	189876328	189876525	COL3A1	693_selected_genes
chr22	19950024	19950363	COMT	693_selected_genes

chr22	19951063	19951307	COMT	693_selected_genes
chr22	19951607	19951940	COMT	693_selected_genes
chr22	19954661	19954975	COMT	693_selected_genes
chr22	19956033	19956284	COMT	693_selected_genes
chr3	3192523	3192754	CRBN	693_selected_genes
chr3	3194114	3194296	CRBN	693_selected_genes
chr3	3195082	3195197	CRBN	693_selected_genes
chr3	3195618	3195784	CRBN	693_selected_genes
chr3	3196405	3196540	CRBN	693_selected_genes
chr3	3197877	3197990	CRBN	693_selected_genes
chr3	3209280	3209502	CRBN	693_selected_genes
chr3	3214434	3214634	CRBN	693_selected_genes
chr3	3215717	3215970	CRBN	693_selected_genes
chr3	3216821	3216978	CRBN	693_selected_genes
chr3	3221279	3221396	CRBN	693_selected_genes
chr2	208420334	208420498	CREB1	693_selected_genes
chr2	208424924	208425121	CREB1	693_selected_genes
chr2	208425817	208425909	CREB1	693_selected_genes
chr2	208432161	208432312	CREB1	693_selected_genes
chr2	208434877	208435070	CREB1	693_selected_genes
chr2	208436432	208436517	CREB1	693_selected_genes
chr2	208439970	208440203	CREB1	693_selected_genes
chr2	208441329	208441396	CREB1	693_selected_genes
chr2	208442203	208442507	CREB1	693_selected_genes
chr2	208461612	208461807	CREB1	693_selected_genes
chr16	3777693	3779900	CREBBP	693_selected_genes
chr16	3781167	3781499	CREBBP	693_selected_genes
chr16	3781751	3781963	CREBBP	693_selected_genes
chr16	3786011	3786229	CREBBP	693_selected_genes
chr16	3786625	3786841	CREBBP	693_selected_genes
chr16	3788534	3788698	CREBBP	693_selected_genes
chr16	3789549	3789750	CREBBP	693_selected_genes
chr16	3790374	3790575	CREBBP	693_selected_genes
chr16	3794869	3794987	CREBBP	693_selected_genes
chr16	3795252	3795380	CREBBP	693_selected_genes
chr16	3799602	3799709	CREBBP	693_selected_genes
chr16	3801701	3801834	CREBBP	693_selected_genes
chr16	3807263	3807402	CREBBP	693_selected_genes
chr16	3807784	3808074	CREBBP	693_selected_genes
chr16	3808829	3808998	CREBBP	693_selected_genes
chr16	3817695	3817935	CREBBP	693_selected_genes
chr16	3819149	3819379	CREBBP	693_selected_genes
chr16	3820545	3821012	CREBBP	693_selected_genes
chr16	3823693	3823956	CREBBP	693_selected_genes
chr16	3824544	3824719	CREBBP	693_selected_genes
chr16	3827588	3827683	CREBBP	693_selected_genes
chr16	3827986	3828208	CREBBP	693_selected_genes
chr16	3828675	3828843	CREBBP	693_selected_genes
chr16	3830707	3830904	CREBBP	693_selected_genes
chr16	3831179	3831332	CREBBP	693_selected_genes
chr16	3832659	3832952	CREBBP	693_selected_genes
chr16	3841956	3842120	CREBBP	693_selected_genes
chr16	3843361	3843652	CREBBP	693_selected_genes
chr16	3860578	3860805	CREBBP	693_selected_genes
chr16	3900272	3901035	CREBBP	693_selected_genes
chr16	3929807	3929942	CREBBP	693_selected_genes
chr22	21272197	21272558	CRKL	693_selected_genes
chr22	21288041	21288557	CRKL	693_selected_genes
chr22	21303973	21304158	CRKL	693_selected_genes
chrY	1264864	1265039	CRLF2	693_selected_genes
chrY	1267393	1267606	CRLF2	693_selected_genes
chrY	1267764	1268033	CRLF2	693_selected_genes
chrY	1271246	1271430	CRLF2	693_selected_genes
chrY	1275300	1275517	CRLF2	693_selected_genes
chrY	1277673	1277826	CRLF2	693_selected_genes
chrY	1281423	1281552	CRLF2	693_selected_genes
chrX	1314864	1315039	CRLF2	693_selected_genes
chrX	1317393	1317606	CRLF2	693_selected_genes
chrX	1317764	1318033	CRLF2	693_selected_genes
chrX	1321246	1321430	CRLF2	693_selected_genes
chrX	1325300	1325517	CRLF2	693_selected_genes
chrX	1327673	1327826	CRLF2	693_selected_genes
chrX	1331423	1331552	CRLF2	693_selected_genes
chr19	18794487	18794663	CRTC1	693_selected_genes
chr19	18846425	18846478	CRTC1	693_selected_genes
chr19	18853694	18853861	CRTC1	693_selected_genes
chr19	18854892	18854990	CRTC1	693_selected_genes
chr19	18856607	18856795	CRTC1	693_selected_genes
chr19	18857837	18857949	CRTC1	693_selected_genes
chr19	18860565	18860710	CRTC1	693_selected_genes
chr19	18864284	18864420	CRTC1	693_selected_genes
chr19	18870335	18870426	CRTC1	693_selected_genes
chr19	18870792	18871063	CRTC1	693_selected_genes
chr19	18876188	18876363	CRTC1	693_selected_genes

chr19	18879269	18879628	CRTC1	693_selected_genes
chr19	18882226	18882381	CRTC1	693_selected_genes
chr19	18885684	18885821	CRTC1	693_selected_genes
chr19	18886425	18886656	CRTC1	693_selected_genes
chr19	18887955	18888217	CRTC1	693_selected_genes
chr15	91073278	91073460	CRTC3	693_selected_genes
chr15	91083245	91083394	CRTC3	693_selected_genes
chr15	91136842	91137012	CRTC3	693_selected_genes
chr15	91141656	91141751	CRTC3	693_selected_genes
chr15	91145530	91145642	CRTC3	693_selected_genes
chr15	91147591	91147704	CRTC3	693_selected_genes
chr15	91150584	91150735	CRTC3	693_selected_genes
chr15	91157659	91157745	CRTC3	693_selected_genes
chr15	91161089	91161225	CRTC3	693_selected_genes
chr15	91162947	91163047	CRTC3	693_selected_genes
chr15	91168982	91169250	CRTC3	693_selected_genes
chr15	91172440	91172789	CRTC3	693_selected_genes
chr15	91181652	91181903	CRTC3	693_selected_genes
chr15	91181941	91182072	CRTC3	693_selected_genes
chr15	91184303	91184456	CRTC3	693_selected_genes
chr15	91185138	91185397	CRTC3	693_selected_genes
chr1	115260764	115260862	CSDE1	693_selected_genes
chr1	115261208	115261391	CSDE1	693_selected_genes
chr1	115262174	115262388	CSDE1	693_selected_genes
chr1	115263134	115263363	CSDE1	693_selected_genes
chr1	115266478	115266648	CSDE1	693_selected_genes
chr1	115267816	115267979	CSDE1	693_selected_genes
chr1	115268806	115269032	CSDE1	693_selected_genes
chr1	115269578	115269736	CSDE1	693_selected_genes
chr1	115272853	115273068	CSDE1	693_selected_genes
chr1	115273103	115273294	CSDE1	693_selected_genes
chr1	115275199	115275462	CSDE1	693_selected_genes
chr1	115276327	115276503	CSDE1	693_selected_genes
chr1	115276584	115276763	CSDE1	693_selected_genes
chr1	115277037	115277169	CSDE1	693_selected_genes
chr1	115279353	115279501	CSDE1	693_selected_genes
chr1	115280066	115280209	CSDE1	693_selected_genes
chr1	115280558	115280718	CSDE1	693_selected_genes
chr1	115282287	115282536	CSDE1	693_selected_genes
chr1	115284122	115284310	CSDE1	693_selected_genes
chr5	149433606	149433812	CSF1R	693_selected_genes
chr5	149433859	149434018	CSF1R	693_selected_genes
chr5	149434774	149434924	CSF1R	693_selected_genes
chr5	149435563	149435725	CSF1R	693_selected_genes
chr5	149435756	149435929	CSF1R	693_selected_genes
chr5	149436824	149436972	CSF1R	693_selected_genes
chr5	149437041	149437180	CSF1R	693_selected_genes
chr5	149437798	149437859	CSF1R	693_selected_genes
chr5	149439237	149439450	CSF1R	693_selected_genes
chr5	149440399	149440560	CSF1R	693_selected_genes
chr5	149441028	149441256	CSF1R	693_selected_genes
chr5	149441260	149441437	CSF1R	693_selected_genes
chr5	149447752	149447918	CSF1R	693_selected_genes
chr5	149449410	149449651	CSF1R	693_selected_genes
chr5	149449719	149449890	CSF1R	693_selected_genes
chr5	149449993	149450159	CSF1R	693_selected_genes
chr5	149452838	149453081	CSF1R	693_selected_genes
chr5	149456813	149457023	CSF1R	693_selected_genes
chr5	149457649	149457836	CSF1R	693_selected_genes
chr5	149459589	149459924	CSF1R	693_selected_genes
chr5	149460304	149460612	CSF1R	693_selected_genes
chr5	149465916	149466015	CSF1R	693_selected_genes
chr16	67644710	67645541	CTCF	693_selected_genes
chr16	67645828	67646049	CTCF	693_selected_genes
chr16	67650622	67650806	CTCF	693_selected_genes
chr16	67654574	67654745	CTCF	693_selected_genes
chr16	67655319	67655519	CTCF	693_selected_genes
chr16	67660432	67660643	CTCF	693_selected_genes
chr16	67662247	67662480	CTCF	693_selected_genes
chr16	67663275	67663461	CTCF	693_selected_genes
chr16	67670567	67670779	CTCF	693_selected_genes
chr16	67671565	67671800	CTCF	693_selected_genes
chr2	204732640	204732799	CTLA4	693_selected_genes
chr2	204735283	204735681	CTLA4	693_selected_genes
chr2	204736075	204736235	CTLA4	693_selected_genes
chr2	204737405	204737560	CTLA4	693_selected_genes
chr5	138117588	138117743	CTNNA1	693_selected_genes
chr5	138117953	138118066	CTNNA1	693_selected_genes
chr5	138118840	138119088	CTNNA1	693_selected_genes
chr5	138145701	138145918	CTNNA1	693_selected_genes
chr5	138147846	138148016	CTNNA1	693_selected_genes
chr5	138160193	138160513	CTNNA1	693_selected_genes
chr5	138163178	138163432	CTNNA1	693_selected_genes
chr5	138216705	138216818	CTNNA1	693_selected_genes

chr5	138221875	138222006	CTNNA1	693_selected_genes
chr5	138223153	138223356	CTNNA1	693_selected_genes
chr5	138240012	138240155	CTNNA1	693_selected_genes
chr5	138253405	138253612	CTNNA1	693_selected_genes
chr5	138260173	138260424	CTNNA1	693_selected_genes
chr5	138260919	138261121	CTNNA1	693_selected_genes
chr5	138264909	138265070	CTNNA1	693_selected_genes
chr5	138266136	138266368	CTNNA1	693_selected_genes
chr5	138266493	138266649	CTNNA1	693_selected_genes
chr5	138268241	138268426	CTNNA1	693_selected_genes
chr5	138268551	138268672	CTNNA1	693_selected_genes
chr5	138269465	138269803	CTNNA1	693_selected_genes
chr3	41265534	41265597	CTNNB1	693_selected_genes
chr3	41265991	41266269	CTNNB1	693_selected_genes
chr3	41266419	41266723	CTNNB1	693_selected_genes
chr3	41266799	41267088	CTNNB1	693_selected_genes
chr3	41267125	41267377	CTNNB1	693_selected_genes
chr3	41268673	41268868	CTNNB1	693_selected_genes
chr3	41274806	41274960	CTNNB1	693_selected_genes
chr3	41274994	41275383	CTNNB1	693_selected_genes
chr3	41275604	41275813	CTNNB1	693_selected_genes
chr3	41277189	41277359	CTNNB1	693_selected_genes
chr3	41277814	41278015	CTNNB1	693_selected_genes
chr3	41278053	41278225	CTNNB1	693_selected_genes
chr3	41279481	41279592	CTNNB1	693_selected_genes
chr3	41280599	41280858	CTNNB1	693_selected_genes
chr11	57558925	57559170	CTNND1	693_selected_genes
chr11	57561456	57561578	CTNND1	693_selected_genes
chr11	57563023	57563226	CTNND1	693_selected_genes
chr11	57563903	57564489	CTNND1	693_selected_genes
chr11	57569179	57569693	CTNND1	693_selected_genes
chr11	57571067	57571301	CTNND1	693_selected_genes
chr11	57572109	57572277	CTNND1	693_selected_genes
chr11	57573328	57573532	CTNND1	693_selected_genes
chr11	57573907	57573975	CTNND1	693_selected_genes
chr11	57574361	57574480	CTNND1	693_selected_genes
chr11	57575611	57575789	CTNND1	693_selected_genes
chr11	57575836	57576037	CTNND1	693_selected_genes
chr11	57576720	57576963	CTNND1	693_selected_genes
chr11	57577555	57577720	CTNND1	693_selected_genes
chr11	57578845	57578983	CTNND1	693_selected_genes
chr11	57581757	57581870	CTNND1	693_selected_genes
chr11	57582840	57583030	CTNND1	693_selected_genes
chr11	57583361	57583498	CTNND1	693_selected_genes
chr11	57583743	57583805	CTNND1	693_selected_genes
chr11	70253378	70253515	CTTN	693_selected_genes
chr11	70253598	70253722	CTTN	693_selected_genes
chr11	70255911	70256091	CTTN	693_selected_genes
chr11	70260622	70260783	CTTN	693_selected_genes
chr11	70261743	70261848	CTTN	693_selected_genes
chr11	70263093	70263254	CTTN	693_selected_genes
chr11	70265826	70266034	CTTN	693_selected_genes
chr11	70266480	70266641	CTTN	693_selected_genes
chr11	70267550	70267711	CTTN	693_selected_genes
chr11	70268583	70268723	CTTN	693_selected_genes
chr11	70269020	70269126	CTTN	693_selected_genes
chr11	70271412	70271532	CTTN	693_selected_genes
chr11	70275131	70275330	CTTN	693_selected_genes
chr11	70277271	70277411	CTTN	693_selected_genes
chr11	70279181	70279409	CTTN	693_selected_genes
chr11	70279705	70279849	CTTN	693_selected_genes
chr11	70281106	70281293	CTTN	693_selected_genes
chr11	70281546	70281879	CTTN	693_selected_genes
chr11	70282362	70282539	CTTN	693_selected_genes
chr7	148427189	148427379	CUL1	693_selected_genes
chr7	148451042	148451267	CUL1	693_selected_genes
chr7	148454049	148454267	CUL1	693_selected_genes
chr7	148456370	148456471	CUL1	693_selected_genes
chr7	148456614	148456755	CUL1	693_selected_genes
chr7	148457399	148457613	CUL1	693_selected_genes
chr7	148463627	148463840	CUL1	693_selected_genes
chr7	148464685	148464866	CUL1	693_selected_genes
chr7	148480849	148481007	CUL1	693_selected_genes
chr7	148481037	148481194	CUL1	693_selected_genes
chr7	148483617	148483716	CUL1	693_selected_genes
chr7	148484055	148484237	CUL1	693_selected_genes
chr7	148485623	148485791	CUL1	693_selected_genes
chr7	148486816	148486943	CUL1	693_selected_genes
chr7	148487376	148487558	CUL1	693_selected_genes
chr7	148489792	148489935	CUL1	693_selected_genes
chr7	148494878	148494976	CUL1	693_selected_genes
chr7	148495003	148495136	CUL1	693_selected_genes
chr7	148495638	148495794	CUL1	693_selected_genes
chr7	148496341	148496505	CUL1	693_selected_genes

chr7	148497568	148497699	CUL1	693_selected_genes
chr13	113863916	113864114	CUL4A	693_selected_genes
chr13	113864261	113864427	CUL4A	693_selected_genes
chr13	113873233	113873387	CUL4A	693_selected_genes
chr13	113882264	113882384	CUL4A	693_selected_genes
chr13	113883734	113883858	CUL4A	693_selected_genes
chr13	113887441	113887678	CUL4A	693_selected_genes
chr13	113888185	113888325	CUL4A	693_selected_genes
chr13	113889351	113889484	CUL4A	693_selected_genes
chr13	113891111	113891229	CUL4A	693_selected_genes
chr13	113893721	113893890	CUL4A	693_selected_genes
chr13	113897256	113897499	CUL4A	693_selected_genes
chr13	113898698	113898853	CUL4A	693_selected_genes
chr13	113899237	113899398	CUL4A	693_selected_genes
chr13	113899440	113899576	CUL4A	693_selected_genes
chr13	113900244	113900402	CUL4A	693_selected_genes
chr13	113907370	113907534	CUL4A	693_selected_genes
chr13	113908981	113909137	CUL4A	693_selected_genes
chr13	113909241	113909464	CUL4A	693_selected_genes
chr13	113914895	113915098	CUL4A	693_selected_genes
chr13	113917775	113917921	CUL4A	693_selected_genes
chrX	119660590	119660736	CUL4B	693_selected_genes
chrX	119663931	119664134	CUL4B	693_selected_genes
chrX	119666251	119666474	CUL4B	693_selected_genes
chrX	119668310	119668466	CUL4B	693_selected_genes
chrX	119669659	119669823	CUL4B	693_selected_genes
chrX	119670756	119670914	CUL4B	693_selected_genes
chrX	119671953	119672089	CUL4B	693_selected_genes
chrX	119672489	119672650	CUL4B	693_selected_genes
chrX	119673097	119673252	CUL4B	693_selected_genes
chrX	119674199	119674442	CUL4B	693_selected_genes
chrX	119675431	119675600	CUL4B	693_selected_genes
chrX	119676795	119676913	CUL4B	693_selected_genes
chrX	119677556	119677689	CUL4B	693_selected_genes
chrX	119677943	119678083	CUL4B	693_selected_genes
chrX	119678310	119678523	CUL4B	693_selected_genes
chrX	119679273	119679397	CUL4B	693_selected_genes
chrX	119680376	119680496	CUL4B	693_selected_genes
chrX	119680965	119681119	CUL4B	693_selected_genes
chrX	119691753	119691919	CUL4B	693_selected_genes
chrX	119693608	119693680	CUL4B	693_selected_genes
chrX	119693912	119694518	CUL4B	693_selected_genes
chrX	119695201	119695279	CUL4B	693_selected_genes
chrX	119708380	119708497	CUL4B	693_selected_genes
chr7	101459285	101459398	CUX1	693_selected_genes
chr7	101460894	101460974	CUX1	693_selected_genes
chr7	101559369	101559530	CUX1	693_selected_genes
chr7	101671352	101671450	CUX1	693_selected_genes
chr7	101713593	101713722	CUX1	693_selected_genes
chr7	101740618	101740806	CUX1	693_selected_genes
chr7	101747590	101747764	CUX1	693_selected_genes
chr7	101754952	101755079	CUX1	693_selected_genes
chr7	101758461	101758578	CUX1	693_selected_genes
chr7	101801814	101801913	CUX1	693_selected_genes
chr7	101813700	101813855	CUX1	693_selected_genes
chr7	101821723	101821962	CUX1	693_selected_genes
chr7	101833067	101833176	CUX1	693_selected_genes
chr7	101837096	101837195	CUX1	693_selected_genes
chr7	101838761	101838908	CUX1	693_selected_genes
chr7	101839888	101840610	CUX1	693_selected_genes
chr7	101842056	101842172	CUX1	693_selected_genes
chr7	101843325	101843477	CUX1	693_selected_genes
chr7	101844614	101845509	CUX1	693_selected_genes
chr7	101847645	101847861	CUX1	693_selected_genes
chr7	101848368	101848475	CUX1	693_selected_genes
chr7	101870621	101870974	CUX1	693_selected_genes
chr7	101877306	101877545	CUX1	693_selected_genes
chr7	101882574	101882889	CUX1	693_selected_genes
chr7	101891666	101892347	CUX1	693_selected_genes
chr7	101916611	101916789	CUX1	693_selected_genes
chr7	101917419	101917606	CUX1	693_selected_genes
chr7	101918492	101918655	CUX1	693_selected_genes
chr7	101921194	101921361	CUX1	693_selected_genes
chr7	101923303	101923437	CUX1	693_selected_genes
chr7	101924070	101924177	CUX1	693_selected_genes
chr7	101925106	101925237	CUX1	693_selected_genes
chr7	101925978	101926093	CUX1	693_selected_genes
chr7	101926287	101926407	CUX1	693_selected_genes
chr16	50783584	50784138	CYLD	693_selected_genes
chr16	50785489	50785842	CYLD	693_selected_genes
chr16	50788204	50788401	CYLD	693_selected_genes
chr16	50809051	50809110	CYLD	693_selected_genes
chr16	50810055	50810213	CYLD	693_selected_genes
chr16	50811710	50811877	CYLD	693_selected_genes

chr16	50813550	50813980	CYLD	693_selected_genes
chr16	50815131	50815347	CYLD	693_selected_genes
chr16	50816210	50816402	CYLD	693_selected_genes
chr16	50818214	50818387	CYLD	693_selected_genes
chr16	50820740	50820882	CYLD	693_selected_genes
chr16	50821671	50821788	CYLD	693_selected_genes
chr16	50825443	50825626	CYLD	693_selected_genes
chr16	50826482	50826641	CYLD	693_selected_genes
chr16	50827431	50827600	CYLD	693_selected_genes
chr16	50828097	50828414	CYLD	693_selected_genes
chr16	50829433	50829539	CYLD	693_selected_genes
chr16	50830209	50830444	CYLD	693_selected_genes
chr16	50834705	50834778	CYLD	693_selected_genes
chr10	104590433	104590767	CYP17A1	693_selected_genes
chr10	104591239	104591393	CYP17A1	693_selected_genes
chr10	104592242	104592462	CYP17A1	693_selected_genes
chr10	104592724	104592990	CYP17A1	693_selected_genes
chr10	104593767	104593904	CYP17A1	693_selected_genes
chr10	104594516	104594796	CYP17A1	693_selected_genes
chr10	104594985	104595174	CYP17A1	693_selected_genes
chr10	104596796	104597143	CYP17A1	693_selected_genes
chr2	38297839	38298478	CYP1B1	693_selected_genes
chr2	38301463	38302556	CYP1B1	693_selected_genes
chr10	96522437	96522655	CYP2C19	693_selected_genes
chr10	96534789	96535002	CYP2C19	693_selected_genes
chr10	96535121	96535321	CYP2C19	693_selected_genes
chr10	96540230	96540441	CYP2C19	693_selected_genes
chr10	96541552	96541779	CYP2C19	693_selected_genes
chr10	96580227	96580419	CYP2C19	693_selected_genes
chr10	96602568	96602806	CYP2C19	693_selected_genes
chr10	96609648	96609840	CYP2C19	693_selected_genes
chr10	96612464	96612696	CYP2C19	693_selected_genes
chr10	96796859	96797091	CYP2C8	693_selected_genes
chr10	96798628	96798820	CYP2C8	693_selected_genes
chr10	96800659	96800742	CYP2C8	693_selected_genes
chr10	96802621	96802859	CYP2C8	693_selected_genes
chr10	96805541	96805733	CYP2C8	693_selected_genes
chr10	96818066	96818293	CYP2C8	693_selected_genes
chr10	96824531	96824742	CYP2C8	693_selected_genes
chr10	96826939	96827212	CYP2C8	693_selected_genes
chr10	96827260	96827473	CYP2C8	693_selected_genes
chr10	96828928	96829184	CYP2C8	693_selected_genes
chr10	96698414	96698632	CYP2C9	693_selected_genes
chr10	96701589	96701802	CYP2C9	693_selected_genes
chr10	96701923	96702123	CYP2C9	693_selected_genes
chr10	96707510	96707721	CYP2C9	693_selected_genes
chr10	96708839	96709066	CYP2C9	693_selected_genes
chr10	96731835	96732027	CYP2C9	693_selected_genes
chr10	96740914	96741152	CYP2C9	693_selected_genes
chr10	96745764	96745956	CYP2C9	693_selected_genes
chr10	96748578	96748810	CYP2C9	693_selected_genes
chr22	42522550	42522779	CYP2D6	693_selected_genes
chr22	42522827	42523019	CYP2D6	693_selected_genes
chr22	42523423	42523661	CYP2D6	693_selected_genes
chr22	42523818	42524010	CYP2D6	693_selected_genes
chr22	42524150	42524377	CYP2D6	693_selected_genes
chr22	42524760	42524971	CYP2D6	693_selected_genes
chr22	42525009	42525212	CYP2D6	693_selected_genes
chr22	42525357	42525416	CYP2D6	693_selected_genes
chr22	42525713	42525939	CYP2D6	693_selected_genes
chr22	42526588	42526818	CYP2D6	693_selected_genes
chr7	99355730	99355876	CYP3A4	693_selected_genes
chr7	99358416	99358629	CYP3A4	693_selected_genes
chr7	99359638	99359915	CYP3A4	693_selected_genes
chr7	99361452	99361663	CYP3A4	693_selected_genes
chr7	99363974	99364091	CYP3A4	693_selected_genes
chr7	99364728	99364906	CYP3A4	693_selected_genes
chr7	99365951	99366150	CYP3A4	693_selected_genes
chr7	99367365	99367504	CYP3A4	693_selected_genes
chr7	99367719	99367883	CYP3A4	693_selected_genes
chr7	99370187	99370337	CYP3A4	693_selected_genes
chr7	99375625	99375728	CYP3A4	693_selected_genes
chr7	99377589	99377772	CYP3A4	693_selected_genes
chr7	99381608	99381729	CYP3A4	693_selected_genes
chr7	99245902	99246048	CYP3A5	693_selected_genes
chr7	99247670	99247880	CYP3A5	693_selected_genes
chr7	99250150	99250427	CYP3A5	693_selected_genes
chr7	99258096	99258307	CYP3A5	693_selected_genes
chr7	99260413	99260530	CYP3A5	693_selected_genes
chr7	99261565	99261743	CYP3A5	693_selected_genes
chr7	99262763	99262962	CYP3A5	693_selected_genes
chr7	99264198	99264337	CYP3A5	693_selected_genes
chr7	99264549	99264713	CYP3A5	693_selected_genes
chr7	99269371	99269526	CYP3A5	693_selected_genes

chr7	99270177	99270327	CYP3A5	693_selected_genes
chr7	99272130	99272233	CYP3A5	693_selected_genes
chr7	99273712	99273856	CYP3A5	693_selected_genes
chr7	99273914	99274005	CYP3A5	693_selected_genes
chr7	99274125	99274224	CYP3A5	693_selected_genes
chr7	99277423	99277544	CYP3A5	693_selected_genes
chr6	33286494	33286604	DAXX	693_selected_genes
chr6	33286748	33287021	DAXX	693_selected_genes
chr6	33287131	33287656	DAXX	693_selected_genes
chr6	33287762	33288026	DAXX	693_selected_genes
chr6	33288131	33288393	DAXX	693_selected_genes
chr6	33288487	33289369	DAXX	693_selected_genes
chr6	33289470	33289727	DAXX	693_selected_genes
chr6	33290613	33290716	DAXX	693_selected_genes
chr3	182662856	182662986	DCUN1D1	693_selected_genes
chr3	182665000	182665147	DCUN1D1	693_selected_genes
chr3	182665312	182665445	DCUN1D1	693_selected_genes
chr3	182678988	182679169	DCUN1D1	693_selected_genes
chr3	182681643	182681862	DCUN1D1	693_selected_genes
chr3	182683299	182683566	DCUN1D1	693_selected_genes
chr3	182698249	182698302	DCUN1D1	693_selected_genes
chr11	47236662	47236839	DDB2	693_selected_genes
chr11	47237861	47238048	DDB2	693_selected_genes
chr11	47238383	47238625	DDB2	693_selected_genes
chr11	47254339	47254535	DDB2	693_selected_genes
chr11	47256098	47256248	DDB2	693_selected_genes
chr11	47256282	47256510	DDB2	693_selected_genes
chr11	47256795	47256988	DDB2	693_selected_genes
chr11	47259362	47259577	DDB2	693_selected_genes
chr11	47259663	47259759	DDB2	693_selected_genes
chr11	47260325	47260425	DDB2	693_selected_genes
chr6	30852971	30853033	DDR1	693_selected_genes
chr6	30853527	30853613	DDR1	693_selected_genes
chr6	30856439	30856616	DDR1	693_selected_genes
chr6	30856659	30856812	DDR1	693_selected_genes
chr6	30856953	30857232	DDR1	693_selected_genes
chr6	30858724	30858922	DDR1	693_selected_genes
chr6	30859131	30859281	DDR1	693_selected_genes
chr6	30859753	30859990	DDR1	693_selected_genes
chr6	30860047	30860344	DDR1	693_selected_genes
chr6	30860819	30860965	DDR1	693_selected_genes
chr6	30861023	30861225	DDR1	693_selected_genes
chr6	30862257	30862473	DDR1	693_selected_genes
chr6	30863155	30863316	DDR1	693_selected_genes
chr6	30864372	30864667	DDR1	693_selected_genes
chr6	30864765	30864943	DDR1	693_selected_genes
chr6	30865112	30865399	DDR1	693_selected_genes
chr6	30865824	30866109	DDR1	693_selected_genes
chr6	30866639	30867098	DDR1	693_selected_genes
chr6	30867799	30867884	DDR1	693_selected_genes
chr1	162688828	162688960	DDR2	693_selected_genes
chr1	162722859	162723012	DDR2	693_selected_genes
chr1	162724388	162724670	DDR2	693_selected_genes
chr1	162724920	162725118	DDR2	693_selected_genes
chr1	162725428	162725584	DDR2	693_selected_genes
chr1	162729560	162729794	DDR2	693_selected_genes
chr1	162730975	162731269	DDR2	693_selected_genes
chr1	162735765	162735878	DDR2	693_selected_genes
chr1	162736993	162737174	DDR2	693_selected_genes
chr1	162740066	162740494	DDR2	693_selected_genes
chr1	162741788	162742071	DDR2	693_selected_genes
chr1	162743233	162743411	DDR2	693_selected_genes
chr1	162745416	162745658	DDR2	693_selected_genes
chr1	162745900	162746185	DDR2	693_selected_genes
chr1	162748344	162748544	DDR2	693_selected_genes
chr1	162749876	162750061	DDR2	693_selected_genes
chrX	41193480	41193575	DDX3X	693_selected_genes
chrX	41193820	41194047	DDX3X	693_selected_genes
chrX	41196635	41196743	DDX3X	693_selected_genes
chrX	41198263	41198361	DDX3X	693_selected_genes
chrX	41200711	41200894	DDX3X	693_selected_genes
chrX	41201722	41201931	DDX3X	693_selected_genes
chrX	41201964	41202114	DDX3X	693_selected_genes
chrX	41202443	41202629	DDX3X	693_selected_genes
chrX	41202964	41203100	DDX3X	693_selected_genes
chrX	41203257	41203406	DDX3X	693_selected_genes
chrX	41203466	41203677	DDX3X	693_selected_genes
chrX	41204407	41204602	DDX3X	693_selected_genes
chrX	41204631	41204826	DDX3X	693_selected_genes
chrX	41205456	41205688	DDX3X	693_selected_genes
chrX	41205732	41205900	DDX3X	693_selected_genes
chrX	41206086	41206290	DDX3X	693_selected_genes
chrX	41206539	41206729	DDX3X	693_selected_genes
chrX	41206867	41206997	DDX3X	693_selected_genes

chr17	62496015	62496469	DDX5	693_selected_genes
chr17	62496641	62496916	DDX5	693_selected_genes
chr17	62498102	62498212	DDX5	693_selected_genes
chr17	62498254	62498366	DDX5	693_selected_genes
chr17	62498521	62498692	DDX5	693_selected_genes
chr17	62499014	62499241	DDX5	693_selected_genes
chr17	62499280	62499491	DDX5	693_selected_genes
chr17	62499522	62499714	DDX5	693_selected_genes
chr17	62499895	62500011	DDX5	693_selected_genes
chr17	62500075	62500259	DDX5	693_selected_genes
chr17	62500314	62500461	DDX5	693_selected_genes
chr17	62500769	62500985	DDX5	693_selected_genes
chr17	62501838	62501932	DDX5	693_selected_genes
chr17	62502168	62502262	DDX5	693_selected_genes
chr5	140896392	140896600	DIAPH1	693_selected_genes
chr5	140898485	140898588	DIAPH1	693_selected_genes
chr5	140903684	140903821	DIAPH1	693_selected_genes
chr5	140905579	140905765	DIAPH1	693_selected_genes
chr5	140905838	140906053	DIAPH1	693_selected_genes
chr5	140907112	140907289	DIAPH1	693_selected_genes
chr5	140907994	140908174	DIAPH1	693_selected_genes
chr5	140908243	140908533	DIAPH1	693_selected_genes
chr5	140908713	140908865	DIAPH1	693_selected_genes
chr5	140909144	140909289	DIAPH1	693_selected_genes
chr5	140913876	140914025	DIAPH1	693_selected_genes
chr5	140915595	140915654	DIAPH1	693_selected_genes
chr5	140940432	140940494	DIAPH1	693_selected_genes
chr5	140950969	140951028	DIAPH1	693_selected_genes
chr5	140951463	140951632	DIAPH1	693_selected_genes
chr5	140953033	140953800	DIAPH1	693_selected_genes
chr5	140954508	140954738	DIAPH1	693_selected_genes
chr5	140955771	140955886	DIAPH1	693_selected_genes
chr5	140956297	140956463	DIAPH1	693_selected_genes
chr5	140957016	140957183	DIAPH1	693_selected_genes
chr5	140957766	140957935	DIAPH1	693_selected_genes
chr5	140958056	140958217	DIAPH1	693_selected_genes
chr5	140958629	140958788	DIAPH1	693_selected_genes
chr5	140960285	140960475	DIAPH1	693_selected_genes
chr5	140961853	140961967	DIAPH1	693_selected_genes
chr5	140962747	140962884	DIAPH1	693_selected_genes
chr5	140963026	140963207	DIAPH1	693_selected_genes
chr5	140963665	140963817	DIAPH1	693_selected_genes
chr5	140966583	140966789	DIAPH1	693_selected_genes
chr5	140967765	140967842	DIAPH1	693_selected_genes
chr5	140998339	140998506	DIAPH1	693_selected_genes
chr14	95556809	95557025	DICER1	693_selected_genes
chr14	95557345	95557471	DICER1	693_selected_genes
chr14	95557514	95557727	DICER1	693_selected_genes
chr14	95560199	95560518	DICER1	693_selected_genes
chr14	95562083	95563075	DICER1	693_selected_genes
chr14	95566091	95566297	DICER1	693_selected_genes
chr14	95569657	95570488	DICER1	693_selected_genes
chr14	95571382	95571608	DICER1	693_selected_genes
chr14	95571989	95572145	DICER1	693_selected_genes
chr14	95572352	95572585	DICER1	693_selected_genes
chr14	95573919	95574123	DICER1	693_selected_genes
chr14	95574191	95574455	DICER1	693_selected_genes
chr14	95574635	95574865	DICER1	693_selected_genes
chr14	95577628	95577818	DICER1	693_selected_genes
chr14	95578483	95578609	DICER1	693_selected_genes
chr14	95579403	95579586	DICER1	693_selected_genes
chr14	95581978	95582183	DICER1	693_selected_genes
chr14	95582764	95583057	DICER1	693_selected_genes
chr14	95583933	95584116	DICER1	693_selected_genes
chr14	95590507	95591030	DICER1	693_selected_genes
chr14	95592891	95593110	DICER1	693_selected_genes
chr14	95595783	95595994	DICER1	693_selected_genes
chr14	95596369	95596554	DICER1	693_selected_genes
chr14	95597820	95598001	DICER1	693_selected_genes
chr14	95598826	95599039	DICER1	693_selected_genes
chr14	95599626	95599820	DICER1	693_selected_genes
chr20	61510559	61513791	DIDO1	693_selected_genes
chr20	61522257	61522532	DIDO1	693_selected_genes
chr20	61523313	61523453	DIDO1	693_selected_genes
chr20	61524135	61524339	DIDO1	693_selected_genes
chr20	61524992	61525571	DIDO1	693_selected_genes
chr20	61525739	61525925	DIDO1	693_selected_genes
chr20	61526136	61526291	DIDO1	693_selected_genes
chr20	61526375	61526542	DIDO1	693_selected_genes
chr20	61527559	61527769	DIDO1	693_selected_genes
chr20	61527857	61528373	DIDO1	693_selected_genes
chr20	61536881	61536978	DIDO1	693_selected_genes
chr20	61537112	61537477	DIDO1	693_selected_genes
chr20	61538473	61538736	DIDO1	693_selected_genes

chr20	61541025	61541397	DIDO1	693_selected_genes
chr20	61542100	61542989	DIDO1	693_selected_genes
chr13	73333907	73334041	DIS3	693_selected_genes
chr13	73334641	73334814	DIS3	693_selected_genes
chr13	73335475	73335684	DIS3	693_selected_genes
chr13	73335758	73335977	DIS3	693_selected_genes
chr13	73336035	73336300	DIS3	693_selected_genes
chr13	73337563	73337770	DIS3	693_selected_genes
chr13	73340084	73340221	DIS3	693_selected_genes
chr13	73342897	73343075	DIS3	693_selected_genes
chr13	73345016	73345151	DIS3	693_selected_genes
chr13	73345193	73345308	DIS3	693_selected_genes
chr13	73345907	73346059	DIS3	693_selected_genes
chr13	73346271	73346438	DIS3	693_selected_genes
chr13	73346805	73347002	DIS3	693_selected_genes
chr13	73347796	73347984	DIS3	693_selected_genes
chr13	73348058	73348222	DIS3	693_selected_genes
chr13	73349323	73349538	DIS3	693_selected_genes
chr13	73350037	73350255	DIS3	693_selected_genes
chr13	73351532	73351656	DIS3	693_selected_genes
chr13	73352299	73352543	DIS3	693_selected_genes
chr13	73354958	73355166	DIS3	693_selected_genes
chr13	73355401	73355519	DIS3	693_selected_genes
chr13	73355717	73355995	DIS3	693_selected_genes
chrX	31139924	31140072	DMD	693_selected_genes
chrX	31144733	31144845	DMD	693_selected_genes
chrX	31152193	31152336	DMD	693_selected_genes
chrX	31164382	31164556	DMD	693_selected_genes
chrX	31165366	31165660	DMD	693_selected_genes
chrX	31187534	31187743	DMD	693_selected_genes
chrX	31190439	31190555	DMD	693_selected_genes
chrX	31191630	31191746	DMD	693_selected_genes
chrX	31196023	31196112	DMD	693_selected_genes
chrX	31196756	31196947	DMD	693_selected_genes
chrX	31198461	31198623	DMD	693_selected_genes
chrX	31200829	31201046	DMD	693_selected_genes
chrX	31222052	31222260	DMD	693_selected_genes
chrX	31224673	31224809	DMD	693_selected_genes
chrX	31227589	31227841	DMD	693_selected_genes
chrX	31241138	31241263	DMD	693_selected_genes
chrX	31279046	31279158	DMD	693_selected_genes
chrX	31284901	31284971	DMD	693_selected_genes
chrX	31341689	31341800	DMD	693_selected_genes
chrX	31366647	31366776	DMD	693_selected_genes
chrX	31462572	31462769	DMD	693_selected_genes
chrX	31496197	31496516	DMD	693_selected_genes
chrX	31497074	31497245	DMD	693_selected_genes
chrX	31514879	31515086	DMD	693_selected_genes
chrX	31525372	31525595	DMD	693_selected_genes
chrX	31526299	31526379	DMD	693_selected_genes
chrX	31645764	31646004	DMD	693_selected_genes
chrX	31676081	31676286	DMD	693_selected_genes
chrX	31697466	31697728	DMD	693_selected_genes
chrX	31747722	31747890	DMD	693_selected_genes
chrX	31792051	31792334	DMD	693_selected_genes
chrX	31838066	31838225	DMD	693_selected_genes
chrX	31854809	31854964	DMD	693_selected_genes
chrX	31893279	31893515	DMD	693_selected_genes
chrX	31947687	31947887	DMD	693_selected_genes
chrX	31950171	31950369	DMD	693_selected_genes
chrX	31986430	31986656	DMD	693_selected_genes
chrX	32235007	32235205	DMD	693_selected_genes
chrX	32305620	32305843	DMD	693_selected_genes
chrX	32328173	32328418	DMD	693_selected_genes
chrX	32360191	32360424	DMD	693_selected_genes
chrX	32360933	32360992	DMD	693_selected_genes
chrX	32361225	32361428	DMD	693_selected_genes
chrX	32364034	32364222	DMD	693_selected_genes
chrX	32366497	32366670	DMD	693_selected_genes
chrX	32380879	32381100	DMD	693_selected_genes
chrX	32382673	32382852	DMD	693_selected_genes
chrX	32383111	32383341	DMD	693_selected_genes
chrX	32398601	32398822	DMD	693_selected_genes
chrX	32404401	32404607	DMD	693_selected_genes
chrX	32407592	32407816	DMD	693_selected_genes
chrX	32408162	32408323	DMD	693_selected_genes
chrX	32429843	32430055	DMD	693_selected_genes
chrX	32430110	32430199	DMD	693_selected_genes
chrX	32430253	32430351	DMD	693_selected_genes
chrX	32456332	32456532	DMD	693_selected_genes
chrX	32459271	32459456	DMD	693_selected_genes
chrX	32466547	32466780	DMD	693_selected_genes
chrX	32472753	32472974	DMD	693_selected_genes
chrX	32481530	32481736	DMD	693_selected_genes

chrX	32482677	32482841	DMD	693_selected_genes
chrX	32486589	32486852	DMD	693_selected_genes
chrX	32490255	32490451	DMD	693_selected_genes
chrX	32503010	32503241	DMD	693_selected_genes
chrX	32509368	32509660	DMD	693_selected_genes
chrX	32519846	32519984	DMD	693_selected_genes
chrX	32536048	32536273	DMD	693_selected_genes
chrX	32563250	32563476	DMD	693_selected_genes
chrX	32583793	32584023	DMD	693_selected_genes
chrX	32591621	32591779	DMD	693_selected_genes
chrX	32591836	32591988	DMD	693_selected_genes
chrX	32613848	32614018	DMD	693_selected_genes
chrX	32632394	32632595	DMD	693_selected_genes
chrX	32662223	32662455	DMD	693_selected_genes
chrX	32663055	32663294	DMD	693_selected_genes
chrX	32715961	32716140	DMD	693_selected_genes
chrX	32717203	32717435	DMD	693_selected_genes
chrX	32827584	32827753	DMD	693_selected_genes
chrX	32834559	32834782	DMD	693_selected_genes
chrX	32841386	32841529	DMD	693_selected_genes
chrX	32862874	32863002	DMD	693_selected_genes
chrX	32867819	32867962	DMD	693_selected_genes
chrX	33038230	33038342	DMD	693_selected_genes
chrX	33146238	33146307	DMD	693_selected_genes
chrX	33229373	33229454	DMD	693_selected_genes
chrX	33357350	33357407	DMD	693_selected_genes
chr19	10244317	10244402	DNMT1	693_selected_genes
chr19	10244867	10245008	DNMT1	693_selected_genes
chr19	10246269	10246553	DNMT1	693_selected_genes
chr19	10246771	10246988	DNMT1	693_selected_genes
chr19	10247726	10247981	DNMT1	693_selected_genes
chr19	10248482	10248710	DNMT1	693_selected_genes
chr19	10249089	10249306	DNMT1	693_selected_genes
chr19	10250326	10250518	DNMT1	693_selected_genes
chr19	10250696	10251029	DNMT1	693_selected_genes
chr19	10251431	10251610	DNMT1	693_selected_genes
chr19	10251755	10251890	DNMT1	693_selected_genes
chr19	10252678	10252921	DNMT1	693_selected_genes
chr19	10254416	10254688	DNMT1	693_selected_genes
chr19	10257001	10257225	DNMT1	693_selected_genes
chr19	10259534	10259718	DNMT1	693_selected_genes
chr19	10260103	10260358	DNMT1	693_selected_genes
chr19	10260503	10260669	DNMT1	693_selected_genes
chr19	10262048	10262246	DNMT1	693_selected_genes
chr19	10262400	10262548	DNMT1	693_selected_genes
chr19	10264943	10265180	DNMT1	693_selected_genes
chr19	10265236	10265474	DNMT1	693_selected_genes
chr19	10265555	10265757	DNMT1	693_selected_genes
chr19	10266503	10266646	DNMT1	693_selected_genes
chr19	10267041	10267210	DNMT1	693_selected_genes
chr19	10270308	10270468	DNMT1	693_selected_genes
chr19	10270492	10270623	DNMT1	693_selected_genes
chr19	10270668	10270764	DNMT1	693_selected_genes
chr19	10271034	10271119	DNMT1	693_selected_genes
chr19	10273317	10273449	DNMT1	693_selected_genes
chr19	10273976	10274061	DNMT1	693_selected_genes
chr19	10277248	10277386	DNMT1	693_selected_genes
chr19	10278980	10279065	DNMT1	693_selected_genes
chr19	10283740	10283875	DNMT1	693_selected_genes
chr19	10284521	10284606	DNMT1	693_selected_genes
chr19	10286190	10286319	DNMT1	693_selected_genes
chr19	10287942	10288068	DNMT1	693_selected_genes
chr19	10290531	10290683	DNMT1	693_selected_genes
chr19	10290837	10290935	DNMT1	693_selected_genes
chr19	10291000	10291270	DNMT1	693_selected_genes
chr19	10291428	10291586	DNMT1	693_selected_genes
chr19	10292691	10292778	DNMT1	693_selected_genes
chr19	10305470	10305755	DNMT1	693_selected_genes
chr19	10311524	10311584	DNMT1	693_selected_genes
chr2	25457122	25457314	DNMT3A	693_selected_genes
chr2	25458550	25458719	DNMT3A	693_selected_genes
chr2	25459779	25459899	DNMT3A	693_selected_genes
chr2	25461973	25462109	DNMT3A	693_selected_genes
chr2	25462333	25462407	DNMT3A	693_selected_genes
chr2	25463145	25463344	DNMT3A	693_selected_genes
chr2	25463483	25463624	DNMT3A	693_selected_genes
chr2	25464405	25464601	DNMT3A	693_selected_genes
chr2	25466741	25466876	DNMT3A	693_selected_genes
chr2	25466998	25467232	DNMT3A	693_selected_genes
chr2	25467383	25467546	DNMT3A	693_selected_genes
chr2	25468096	25468226	DNMT3A	693_selected_genes
chr2	25468863	25468958	DNMT3A	693_selected_genes
chr2	25469003	25469203	DNMT3A	693_selected_genes
chr2	25469463	25469670	DNMT3A	693_selected_genes

chr2	25469894	25470052	DNMT3A	693_selected_genes
chr2	25470434	25470643	DNMT3A	693_selected_genes
chr2	25470880	25471146	DNMT3A	693_selected_genes
chr2	25472500	25472618	DNMT3A	693_selected_genes
chr2	25475037	25475091	DNMT3A	693_selected_genes
chr2	25497784	25497981	DNMT3A	693_selected_genes
chr2	25498343	25498437	DNMT3A	693_selected_genes
chr2	25505231	25505605	DNMT3A	693_selected_genes
chr2	25522982	25523137	DNMT3A	693_selected_genes
chr2	25536756	25536878	DNMT3A	693_selected_genes
chr20	31367896	31367976	DNMT3B	693_selected_genes
chr20	31368098	31368296	DNMT3B	693_selected_genes
chr20	31369133	31369245	DNMT3B	693_selected_genes
chr20	31372538	31372690	DNMT3B	693_selected_genes
chr20	31374282	31374458	DNMT3B	693_selected_genes
chr20	31375010	31375282	DNMT3B	693_selected_genes
chr20	31376634	31376843	DNMT3B	693_selected_genes
chr20	31379381	31379539	DNMT3B	693_selected_genes
chr20	31380406	31380601	DNMT3B	693_selected_genes
chr20	31381316	31381426	DNMT3B	693_selected_genes
chr20	31383189	31383365	DNMT3B	693_selected_genes
chr20	31383430	31383525	DNMT3B	693_selected_genes
chr20	31384570	31384700	DNMT3B	693_selected_genes
chr20	31384967	31385130	DNMT3B	693_selected_genes
chr20	31386240	31386474	DNMT3B	693_selected_genes
chr20	31387024	31387159	DNMT3B	693_selected_genes
chr20	31387933	31388129	DNMT3B	693_selected_genes
chr20	31388615	31388756	DNMT3B	693_selected_genes
chr20	31389058	31389257	DNMT3B	693_selected_genes
chr20	31390165	31390301	DNMT3B	693_selected_genes
chr20	31393118	31393238	DNMT3B	693_selected_genes
chr20	31393989	31394158	DNMT3B	693_selected_genes
chr20	31395542	31395734	DNMT3B	693_selected_genes
chr19	2164158	2164289	DOT1L	693_selected_genes
chr19	2180686	2180780	DOT1L	693_selected_genes
chr19	2185828	2185953	DOT1L	693_selected_genes
chr19	2189705	2189819	DOT1L	693_selected_genes
chr19	2190985	2191264	DOT1L	693_selected_genes
chr19	2193662	2193807	DOT1L	693_selected_genes
chr19	2194488	2194601	DOT1L	693_selected_genes
chr19	2199857	2199963	DOT1L	693_selected_genes
chr19	2202673	2202803	DOT1L	693_selected_genes
chr19	2206702	2206821	DOT1L	693_selected_genes
chr19	2207547	2207704	DOT1L	693_selected_genes
chr19	2208908	2209000	DOT1L	693_selected_genes
chr19	2210373	2210534	DOT1L	693_selected_genes
chr19	2210594	2210879	DOT1L	693_selected_genes
chr19	2211072	2211236	DOT1L	693_selected_genes
chr19	2211724	2211866	DOT1L	693_selected_genes
chr19	2213512	2213664	DOT1L	693_selected_genes
chr19	2213822	2214010	DOT1L	693_selected_genes
chr19	2214444	2214620	DOT1L	693_selected_genes
chr19	2215431	2215595	DOT1L	693_selected_genes
chr19	2216254	2216789	DOT1L	693_selected_genes
chr19	2216928	2217114	DOT1L	693_selected_genes
chr19	2217745	2217942	DOT1L	693_selected_genes
chr19	2220081	2220246	DOT1L	693_selected_genes
chr19	2221949	2222583	DOT1L	693_selected_genes
chr19	2223254	2223510	DOT1L	693_selected_genes
chr19	2225361	2225476	DOT1L	693_selected_genes
chr19	2226156	2227151	DOT1L	693_selected_genes
chr19	2227336	2227491	DOT1L	693_selected_genes
chr19	2227710	2228371	DOT1L	693_selected_genes
chr19	2229758	2229816	DOT1L	693_selected_genes
chr1	97544506	97544727	DPYD	693_selected_genes
chr1	97547860	97548051	DPYD	693_selected_genes
chr1	97564019	97564213	DPYD	693_selected_genes
chr1	97658599	97658829	DPYD	693_selected_genes
chr1	97700382	97700575	DPYD	693_selected_genes
chr1	97770789	97770959	DPYD	693_selected_genes
chr1	97771707	97771878	DPYD	693_selected_genes
chr1	97839091	97839225	DPYD	693_selected_genes
chr1	97847923	97848042	DPYD	693_selected_genes
chr1	97915589	97915804	DPYD	693_selected_genes
chr1	97981256	97981522	DPYD	693_selected_genes
chr1	98015090	98015325	DPYD	693_selected_genes
chr1	98039290	98039551	DPYD	693_selected_genes
chr1	98058748	98058968	DPYD	693_selected_genes
chr1	98060589	98060747	DPYD	693_selected_genes
chr1	98144625	98144763	DPYD	693_selected_genes
chr1	98157247	98157379	DPYD	693_selected_genes
chr1	98164881	98165128	DPYD	693_selected_genes
chr1	98165734	98165883	DPYD	693_selected_genes
chr1	98186418	98186507	DPYD	693_selected_genes

chr1	98187040	98187252	DPYD	693_selected_genes
chr1	98205922	98206060	DPYD	693_selected_genes
chr1	98293644	98293777	DPYD	693_selected_genes
chr1	98348794	98348955	DPYD	693_selected_genes
chr1	98386414	98386503	DPYD	693_selected_genes
chr6	20402438	20402881	E2F3	693_selected_genes
chr6	20404020	20404088	E2F3	693_selected_genes
chr6	20480051	20480213	E2F3	693_selected_genes
chr6	20481411	20481681	E2F3	693_selected_genes
chr6	20482967	20483176	E2F3	693_selected_genes
chr6	20486894	20487059	E2F3	693_selected_genes
chr6	20488318	20488504	E2F3	693_selected_genes
chr6	20490373	20490686	E2F3	693_selected_genes
chr11	85956246	85956410	EED	693_selected_genes
chr11	85961312	85961515	EED	693_selected_genes
chr11	85963164	85963307	EED	693_selected_genes
chr11	85966238	85966354	EED	693_selected_genes
chr11	85967403	85967579	EED	693_selected_genes
chr11	85968531	85968663	EED	693_selected_genes
chr11	85975188	85975330	EED	693_selected_genes
chr11	85977099	85977283	EED	693_selected_genes
chr11	85979472	85979628	EED	693_selected_genes
chr11	85981099	85981224	EED	693_selected_genes
chr11	85987996	85988205	EED	693_selected_genes
chr11	85988934	85989062	EED	693_selected_genes
chr11	85989415	85989592	EED	693_selected_genes
chr6	74227507	74227682	EEF1A1	693_selected_genes
chr6	74227727	74228012	EEF1A1	693_selected_genes
chr6	74228051	74228358	EEF1A1	693_selected_genes
chr6	74228395	74228596	EEF1A1	693_selected_genes
chr6	74228629	74228976	EEF1A1	693_selected_genes
chr6	74229034	74229264	EEF1A1	693_selected_genes
chr6	74229580	74229774	EEF1A1	693_selected_genes
chr9	139562709	139562839	EGFL7	693_selected_genes
chr9	139562983	139563150	EGFL7	693_selected_genes
chr9	139564032	139564198	EGFL7	693_selected_genes
chr9	139564340	139564486	EGFL7	693_selected_genes
chr9	139564595	139564807	EGFL7	693_selected_genes
chr9	139565376	139565491	EGFL7	693_selected_genes
chr9	139566352	139566565	EGFL7	693_selected_genes
chr9	139566690	139566763	EGFL7	693_selected_genes
chr7	55086945	55087083	EGFR	693_selected_genes
chr7	55209953	55210155	EGFR	693_selected_genes
chr7	55210972	55211206	EGFR	693_selected_genes
chr7	55214273	55214458	EGFR	693_selected_genes
chr7	55218961	55219080	EGFR	693_selected_genes
chr7	55220213	55220382	EGFR	693_selected_genes
chr7	55221678	55221870	EGFR	693_selected_genes
chr7	55223497	55223664	EGFR	693_selected_genes
chr7	55224200	55224377	EGFR	693_selected_genes
chr7	55224426	55224561	EGFR	693_selected_genes
chr7	55225330	55225471	EGFR	693_selected_genes
chr7	55227806	55228056	EGFR	693_selected_genes
chr7	55229166	55229349	EGFR	693_selected_genes
chr7	55231400	55231541	EGFR	693_selected_genes
chr7	55232947	55233155	EGFR	693_selected_genes
chr7	55236190	55236247	EGFR	693_selected_genes
chr7	55237974	55238262	EGFR	693_selected_genes
chr7	55238842	55238931	EGFR	693_selected_genes
chr7	55240513	55240618	EGFR	693_selected_genes
chr7	55240650	55240842	EGFR	693_selected_genes
chr7	55241588	55241761	EGFR	693_selected_genes
chr7	55242389	55242538	EGFR	693_selected_genes
chr7	55248960	55249196	EGFR	693_selected_genes
chr7	55259386	55259592	EGFR	693_selected_genes
chr7	55260433	55260559	EGFR	693_selected_genes
chr7	55266384	55266581	EGFR	693_selected_genes
chr7	55267983	55268131	EGFR	693_selected_genes
chr7	55268855	55269073	EGFR	693_selected_genes
chr7	55269402	55269500	EGFR	693_selected_genes
chr7	55270184	55270483	EGFR	693_selected_genes
chr7	55272923	55273335	EGFR	693_selected_genes
chr8	22547960	22549020	EGR3	693_selected_genes
chr8	22549789	22549892	EGR3	693_selected_genes
chr8	22550278	22550482	EGR3	693_selected_genes
chrX	20146398	20146454	EIF1AX	693_selected_genes
chrX	20148608	20148750	EIF1AX	693_selected_genes
chrX	20150274	20150406	EIF1AX	693_selected_genes
chrX	20152049	20152150	EIF1AX	693_selected_genes
chrX	20153830	20153984	EIF1AX	693_selected_genes
chrX	20156631	20156765	EIF1AX	693_selected_genes
chrX	20159717	20159783	EIF1AX	693_selected_genes
chr3	186501374	186501453	EIF4A2	693_selected_genes
chr3	186502192	186502291	EIF4A2	693_selected_genes

chr3	186502327	186502510	EIF4A2	693_selected_genes
chr3	186502725	186502915	EIF4A2	693_selected_genes
chr3	186503646	186503865	EIF4A2	693_selected_genes
chr3	186503927	186504087	EIF4A2	693_selected_genes
chr3	186504265	186504459	EIF4A2	693_selected_genes
chr3	186504890	186505078	EIF4A2	693_selected_genes
chr3	186505258	186505398	EIF4A2	693_selected_genes
chr3	186505566	186505696	EIF4A2	693_selected_genes
chr3	186506073	186506133	EIF4A2	693_selected_genes
chr3	186506888	186507083	EIF4A2	693_selected_genes
chr1	201980239	201980452	ELF3	693_selected_genes
chr1	201981059	201981331	ELF3	693_selected_genes
chr1	201981446	201981589	ELF3	693_selected_genes
chr1	201981742	201981912	ELF3	693_selected_genes
chr1	201982049	201982189	ELF3	693_selected_genes
chr1	201982284	201982451	ELF3	693_selected_genes
chr1	201982931	201983177	ELF3	693_selected_genes
chr1	201984311	201984476	ELF3	693_selected_genes
chr2	42396726	42396801	EML4	693_selected_genes
chr2	42472619	42472852	EML4	693_selected_genes
chr2	42483615	42483795	EML4	693_selected_genes
chr2	42488235	42488466	EML4	693_selected_genes
chr2	42490292	42490471	EML4	693_selected_genes
chr2	42491820	42491896	EML4	693_selected_genes
chr2	42492033	42492116	EML4	693_selected_genes
chr2	42507964	42508138	EML4	693_selected_genes
chr2	42509937	42510137	EML4	693_selected_genes
chr2	42511748	42511868	EML4	693_selected_genes
chr2	42513305	42513544	EML4	693_selected_genes
chr2	42515341	42515487	EML4	693_selected_genes
chr2	42522239	42522424	EML4	693_selected_genes
chr2	42522495	42522681	EML4	693_selected_genes
chr2	42528355	42528557	EML4	693_selected_genes
chr2	42530218	42530394	EML4	693_selected_genes
chr2	42530429	42530611	EML4	693_selected_genes
chr2	42531598	42531716	EML4	693_selected_genes
chr2	42543076	42543215	EML4	693_selected_genes
chr2	42544541	42544689	EML4	693_selected_genes
chr2	42552581	42552719	EML4	693_selected_genes
chr2	42553204	42553417	EML4	693_selected_genes
chr2	42556000	42556181	EML4	693_selected_genes
chr2	42556848	42557372	EML4	693_selected_genes
chr22	41488983	41489127	EP300	693_selected_genes
chr22	41513165	41513850	EP300	693_selected_genes
chr22	41521842	41522069	EP300	693_selected_genes
chr22	41523465	41523777	EP300	693_selected_genes
chr22	41525868	41526032	EP300	693_selected_genes
chr22	41527366	41527662	EP300	693_selected_genes
chr22	41531791	41531935	EP300	693_selected_genes
chr22	41533631	41533819	EP300	693_selected_genes
chr22	41536118	41536286	EP300	693_selected_genes
chr22	41537026	41537251	EP300	693_selected_genes
chr22	41542717	41542845	EP300	693_selected_genes
chr22	41543815	41543975	EP300	693_selected_genes
chr22	41545016	41545204	EP300	693_selected_genes
chr22	41545739	41546227	EP300	693_selected_genes
chr22	41547811	41548041	EP300	693_selected_genes
chr22	41548184	41548379	EP300	693_selected_genes
chr22	41550973	41551142	EP300	693_selected_genes
chr22	41553147	41553437	EP300	693_selected_genes
chr22	41554390	41554529	EP300	693_selected_genes
chr22	41556620	41556751	EP300	693_selected_genes
chr22	41558701	41558808	EP300	693_selected_genes
chr22	41560031	41560159	EP300	693_selected_genes
chr22	41562577	41562695	EP300	693_selected_genes
chr22	41564427	41564628	EP300	693_selected_genes
chr22	41564699	41564896	EP300	693_selected_genes
chr22	41565481	41565645	EP300	693_selected_genes
chr22	41566384	41566600	EP300	693_selected_genes
chr22	41568477	41568692	EP300	693_selected_genes
chr22	41569601	41569813	EP300	693_selected_genes
chr22	41572225	41572557	EP300	693_selected_genes
chr22	41572751	41574985	EP300	693_selected_genes
chr2	47596189	47596399	EPCAM	693_selected_genes
chr2	47596619	47596973	EPCAM	693_selected_genes
chr2	47600576	47600734	EPCAM	693_selected_genes
chr2	47600921	47601212	EPCAM	693_selected_genes
chr2	47602347	47602463	EPCAM	693_selected_genes
chr2	47604127	47604241	EPCAM	693_selected_genes
chr2	47606066	47606218	EPCAM	693_selected_genes
chr2	47606882	47607133	EPCAM	693_selected_genes
chr2	47612279	47612374	EPCAM	693_selected_genes
chr2	47613685	47613777	EPCAM	693_selected_genes
chr1	16451684	16451840	EPHA2	693_selected_genes

chr1	16455903	16456109	EPHA2	693_selected_genes
chr1	16456695	16456939	EPHA2	693_selected_genes
chr1	16458190	16458390	EPHA2	693_selected_genes
chr1	16458533	16458793	EPHA2	693_selected_genes
chr1	16458847	16458959	EPHA2	693_selected_genes
chr1	16459649	16459888	EPHA2	693_selected_genes
chr1	16459950	16460126	EPHA2	693_selected_genes
chr1	16460329	16460435	EPHA2	693_selected_genes
chr1	16460937	16461087	EPHA2	693_selected_genes
chr1	16461505	16461709	EPHA2	693_selected_genes
chr1	16462124	16462290	EPHA2	693_selected_genes
chr1	16464322	16464705	EPHA2	693_selected_genes
chr1	16464744	16464950	EPHA2	693_selected_genes
chr1	16474847	16475567	EPHA2	693_selected_genes
chr1	16477365	16477483	EPHA2	693_selected_genes
chr1	16482317	16482452	EPHA2	693_selected_genes
chr3	89156873	89157011	EPHA3	693_selected_genes
chr3	89176333	89176448	EPHA3	693_selected_genes
chr3	89258984	89259695	EPHA3	693_selected_genes
chr3	89390040	89390246	EPHA3	693_selected_genes
chr3	89390879	89391265	EPHA3	693_selected_genes
chr3	89444961	89445136	EPHA3	693_selected_genes
chr3	89448442	89448681	EPHA3	693_selected_genes
chr3	89456393	89456546	EPHA3	693_selected_genes
chr3	89457191	89457306	EPHA3	693_selected_genes
chr3	89462265	89462441	EPHA3	693_selected_genes
chr3	89468329	89468565	EPHA3	693_selected_genes
chr3	89478230	89478342	EPHA3	693_selected_genes
chr3	89480274	89480534	EPHA3	693_selected_genes
chr3	89498349	89498549	EPHA3	693_selected_genes
chr3	89499301	89499545	EPHA3	693_selected_genes
chr3	89521588	89521794	EPHA3	693_selected_genes
chr3	89528521	89528677	EPHA3	693_selected_genes
chr4	66189806	66189962	EPHA5	693_selected_genes
chr4	66197661	66197871	EPHA5	693_selected_genes
chr4	66201624	66201868	EPHA5	693_selected_genes
chr4	66213746	66213946	EPHA5	693_selected_genes
chr4	66217081	66217341	EPHA5	693_selected_genes
chr4	66218734	66218846	EPHA5	693_selected_genes
chr4	66230709	66230945	EPHA5	693_selected_genes
chr4	66231624	66231800	EPHA5	693_selected_genes
chr4	66233049	66233167	EPHA5	693_selected_genes
chr4	66242690	66242806	EPHA5	693_selected_genes
chr4	66270066	66270222	EPHA5	693_selected_genes
chr4	66279976	66280186	EPHA5	693_selected_genes
chr4	66286133	66286308	EPHA5	693_selected_genes
chr4	66356069	66356455	EPHA5	693_selected_genes
chr4	66361080	66361286	EPHA5	693_selected_genes
chr4	66467333	66468047	EPHA5	693_selected_genes
chr4	66509055	66509170	EPHA5	693_selected_genes
chr4	66535254	66535485	EPHA5	693_selected_genes
chr3	96533442	96533877	EPHA6	693_selected_genes
chr3	96585643	96585758	EPHA6	693_selected_genes
chr3	96706148	96706862	EPHA6	693_selected_genes
chr3	96728822	96728898	EPHA6	693_selected_genes
chr3	96945082	96945288	EPHA6	693_selected_genes
chr3	96962770	96963194	EPHA6	693_selected_genes
chr3	97123968	97124143	EPHA6	693_selected_genes
chr3	97167386	97167599	EPHA6	693_selected_genes
chr3	97185237	97185351	EPHA6	693_selected_genes
chr3	97194170	97194329	EPHA6	693_selected_genes
chr3	97198112	97198233	EPHA6	693_selected_genes
chr3	97202752	97202928	EPHA6	693_selected_genes
chr3	97251176	97251412	EPHA6	693_selected_genes
chr3	97278409	97278524	EPHA6	693_selected_genes
chr3	97311430	97311606	EPHA6	693_selected_genes
chr3	97329611	97329723	EPHA6	693_selected_genes
chr3	97331196	97331300	EPHA6	693_selected_genes
chr3	97356691	97356951	EPHA6	693_selected_genes
chr3	97364937	97365179	EPHA6	693_selected_genes
chr3	97367104	97367199	EPHA6	693_selected_genes
chr3	97439079	97439279	EPHA6	693_selected_genes
chr3	97454743	97454987	EPHA6	693_selected_genes
chr3	97464177	97464249	EPHA6	693_selected_genes
chr3	97466241	97466441	EPHA6	693_selected_genes
chr3	97467405	97467570	EPHA6	693_selected_genes
chr6	93953118	93953283	EPHA7	693_selected_genes
chr6	93954990	93955196	EPHA7	693_selected_genes
chr6	93956484	93956728	EPHA7	693_selected_genes
chr6	93964339	93964539	EPHA7	693_selected_genes
chr6	93965520	93965780	EPHA7	693_selected_genes
chr6	93967154	93967266	EPHA7	693_selected_genes
chr6	93967791	93968027	EPHA7	693_selected_genes
chr6	93969046	93969222	EPHA7	693_selected_genes

chr6	93973552	93973658	EPHA7	693_selected_genes
chr6	93974286	93974445	EPHA7	693_selected_genes
chr6	93979169	93979403	EPHA7	693_selected_genes
chr6	93981990	93982165	EPHA7	693_selected_genes
chr6	94066409	94066795	EPHA7	693_selected_genes
chr6	94067948	94068154	EPHA7	693_selected_genes
chr6	94120185	94120913	EPHA7	693_selected_genes
chr6	94124395	94124510	EPHA7	693_selected_genes
chr6	94128937	94129084	EPHA7	693_selected_genes
chr3	134514448	134514556	EPHB1	693_selected_genes
chr3	134644632	134644747	EPHB1	693_selected_genes
chr3	134670187	134670919	EPHB1	693_selected_genes
chr3	134696782	134696873	EPHB1	693_selected_genes
chr3	134825264	134825470	EPHB1	693_selected_genes
chr3	134851530	134851916	EPHB1	693_selected_genes
chr3	134872968	134873143	EPHB1	693_selected_genes
chr3	134880834	134881047	EPHB1	693_selected_genes
chr3	134884784	134884943	EPHB1	693_selected_genes
chr3	134885758	134885873	EPHB1	693_selected_genes
chr3	134898676	134898849	EPHB1	693_selected_genes
chr3	134911392	134911690	EPHB1	693_selected_genes
chr3	134920290	134920556	EPHB1	693_selected_genes
chr3	134959964	134960164	EPHB1	693_selected_genes
chr3	134967132	134967376	EPHB1	693_selected_genes
chr3	134968152	134968358	EPHB1	693_selected_genes
chr3	134977828	134977987	EPHB1	693_selected_genes
chr1	23037450	23037561	EPHB2	693_selected_genes
chr1	23101669	23101762	EPHB2	693_selected_genes
chr1	23107888	23108003	EPHB2	693_selected_genes
chr1	23110859	23111594	EPHB2	693_selected_genes
chr1	23189504	23189710	EPHB2	693_selected_genes
chr1	23191344	23191730	EPHB2	693_selected_genes
chr1	23208826	23209001	EPHB2	693_selected_genes
chr1	23217596	23217667	EPHB2	693_selected_genes
chr1	23219348	23219564	EPHB2	693_selected_genes
chr1	23221939	23222098	EPHB2	693_selected_genes
chr1	23222878	23222996	EPHB2	693_selected_genes
chr1	23232454	23232627	EPHB2	693_selected_genes
chr1	23233177	23233475	EPHB2	693_selected_genes
chr1	23234420	23234686	EPHB2	693_selected_genes
chr1	23235489	23235689	EPHB2	693_selected_genes
chr1	23236849	23237093	EPHB2	693_selected_genes
chr1	23238911	23239121	EPHB2	693_selected_genes
chr1	23239929	23240088	EPHB2	693_selected_genes
chr1	23240125	23240388	EPHB2	693_selected_genes
chr7	142560960	142561110	EPHB6	693_selected_genes
chr7	142561363	142561478	EPHB6	693_selected_genes
chr7	142561698	142562529	EPHB6	693_selected_genes
chr7	142563204	142563410	EPHB6	693_selected_genes
chr7	142563689	142564096	EPHB6	693_selected_genes
chr7	142564210	142564385	EPHB6	693_selected_genes
chr7	142564635	142564848	EPHB6	693_selected_genes
chr7	142565337	142565502	EPHB6	693_selected_genes
chr7	142565726	142565829	EPHB6	693_selected_genes
chr7	142565970	142566140	EPHB6	693_selected_genes
chr7	142566221	142566519	EPHB6	693_selected_genes
chr7	142566701	142566925	EPHB6	693_selected_genes
chr7	142567544	142567744	EPHB6	693_selected_genes
chr7	142567941	142568185	EPHB6	693_selected_genes
chr7	142568257	142568463	EPHB6	693_selected_genes
chr7	142568523	142568682	EPHB6	693_selected_genes
chr8	144940133	144947446	EPPK1	693_selected_genes
chr17	37855787	37855865	ERBB2	693_selected_genes
chr17	37856466	37856589	ERBB2	693_selected_genes
chr17	37863217	37863467	ERBB2	693_selected_genes
chr17	37864548	37864812	ERBB2	693_selected_genes
chr17	37865545	37865730	ERBB2	693_selected_genes
chr17	37866040	37866159	ERBB2	693_selected_genes
chr17	37866313	37866479	ERBB2	693_selected_genes
chr17	37866567	37866759	ERBB2	693_selected_genes
chr17	37868155	37868325	ERBB2	693_selected_genes
chr17	37868549	37868726	ERBB2	693_selected_genes
chr17	37869380	37869547	ERBB2	693_selected_genes
chr17	37871513	37871637	ERBB2	693_selected_genes
chr17	37871673	37871814	ERBB2	693_selected_genes
chr17	37871967	37872217	ERBB2	693_selected_genes
chr17	37872528	37872711	ERBB2	693_selected_genes
chr17	37872742	37872883	ERBB2	693_selected_genes
chr17	37873547	37873762	ERBB2	693_selected_genes
chr17	37876014	37876112	ERBB2	693_selected_genes
chr17	37879546	37879735	ERBB2	693_selected_genes
chr17	37879765	37879938	ERBB2	693_selected_genes
chr17	37880139	37880288	ERBB2	693_selected_genes
chr17	37880953	37881189	ERBB2	693_selected_genes

chr17	37881276	37881482	ERBB2	693_selected_genes
chr17	37881554	37881680	ERBB2	693_selected_genes
chr17	37881934	37882131	ERBB2	693_selected_genes
chr17	37882789	37882937	ERBB2	693_selected_genes
chr17	37883042	37883281	ERBB2	693_selected_genes
chr17	37883522	37883825	ERBB2	693_selected_genes
chr17	37883916	37884322	ERBB2	693_selected_genes
chr12	56474059	56474191	ERBB3	693_selected_genes
chr12	56477509	56477711	ERBB3	693_selected_genes
chr12	56478753	56479121	ERBB3	693_selected_genes
chr12	56480289	56480465	ERBB3	693_selected_genes
chr12	56481335	56481451	ERBB3	693_selected_genes
chr12	56481553	56481722	ERBB3	693_selected_genes
chr12	56481779	56481971	ERBB3	693_selected_genes
chr12	56482301	56482465	ERBB3	693_selected_genes
chr12	56482506	56482677	ERBB3	693_selected_genes
chr12	56486505	56486629	ERBB3	693_selected_genes
chr12	56486744	56486885	ERBB3	693_selected_genes
chr12	56487103	56487359	ERBB3	693_selected_genes
chr12	56487522	56487705	ERBB3	693_selected_genes
chr12	56487857	56487998	ERBB3	693_selected_genes
chr12	56488160	56488365	ERBB3	693_selected_genes
chr12	56489015	56489119	ERBB3	693_selected_genes
chr12	56489423	56489615	ERBB3	693_selected_genes
chr12	56490261	56490431	ERBB3	693_selected_genes
chr12	56490506	56490655	ERBB3	693_selected_genes
chr12	56490803	56491039	ERBB3	693_selected_genes
chr12	56491543	56491749	ERBB3	693_selected_genes
chr12	56492258	56492458	ERBB3	693_selected_genes
chr12	56492465	56492714	ERBB3	693_selected_genes
chr12	56493406	56493554	ERBB3	693_selected_genes
chr12	56493596	56493838	ERBB3	693_selected_genes
chr12	56493932	56494054	ERBB3	693_selected_genes
chr12	56494819	56495170	ERBB3	693_selected_genes
chr12	56495287	56495864	ERBB3	693_selected_genes
chr2	212248314	212248810	ERBB4	693_selected_genes
chr2	212251552	212251900	ERBB4	693_selected_genes
chr2	212252644	212252742	ERBB4	693_selected_genes
chr2	212285140	212285361	ERBB4	693_selected_genes
chr2	212286706	212286854	ERBB4	693_selected_genes
chr2	212288854	212289051	ERBB4	693_selected_genes
chr2	212293107	212293233	ERBB4	693_selected_genes
chr2	212295644	212295850	ERBB4	693_selected_genes
chr2	212426602	212426838	ERBB4	693_selected_genes
chr2	212483876	212484025	ERBB4	693_selected_genes
chr2	212488621	212488794	ERBB4	693_selected_genes
chr2	212495161	212495344	ERBB4	693_selected_genes
chr2	212522453	212522578	ERBB4	693_selected_genes
chr2	212522649	212522744	ERBB4	693_selected_genes
chr2	212530022	212530227	ERBB4	693_selected_genes
chr2	212537863	212538007	ERBB4	693_selected_genes
chr2	212543751	212543934	ERBB4	693_selected_genes
chr2	212566666	212566916	ERBB4	693_selected_genes
chr2	212568803	212568944	ERBB4	693_selected_genes
chr2	212570017	212570141	ERBB4	693_selected_genes
chr2	212576749	212576926	ERBB4	693_selected_genes
chr2	212578234	212578398	ERBB4	693_selected_genes
chr2	212587092	212587284	ERBB4	693_selected_genes
chr2	212589775	212589944	ERBB4	693_selected_genes
chr2	212615338	212615454	ERBB4	693_selected_genes
chr2	212652724	212652909	ERBB4	693_selected_genes
chr2	212812129	212812366	ERBB4	693_selected_genes
chr2	212989451	212989653	ERBB4	693_selected_genes
chr2	213403147	213403279	ERBB4	693_selected_genes
chr19	45912907	45913008	ERCC1	693_selected_genes
chr19	45916780	45917028	ERCC1	693_selected_genes
chr19	45917195	45917317	ERCC1	693_selected_genes
chr19	45918093	45918243	ERCC1	693_selected_genes
chr19	45920053	45920180	ERCC1	693_selected_genes
chr19	45922330	45922480	ERCC1	693_selected_genes
chr19	45922643	45922772	ERCC1	693_selected_genes
chr19	45923556	45923710	ERCC1	693_selected_genes
chr19	45924410	45924676	ERCC1	693_selected_genes
chr19	45926502	45926657	ERCC1	693_selected_genes
chr19	45854861	45855004	ERCC2	693_selected_genes
chr19	45855441	45855635	ERCC2	693_selected_genes
chr19	45855738	45855932	ERCC2	693_selected_genes
chr19	45855978	45856099	ERCC2	693_selected_genes
chr19	45856315	45856438	ERCC2	693_selected_genes
chr19	45856474	45856617	ERCC2	693_selected_genes
chr19	45857962	45858134	ERCC2	693_selected_genes
chr19	45858897	45859011	ERCC2	693_selected_genes
chr19	45860502	45860654	ERCC2	693_selected_genes
chr19	45860706	45860826	ERCC2	693_selected_genes

chr19	45860862	45860982	ERCC2	693_selected_genes
chr19	45862092	45862195	ERCC2	693_selected_genes
chr19	45864756	45864925	ERCC2	693_selected_genes
chr19	45866975	45867194	ERCC2	693_selected_genes
chr19	45867218	45867402	ERCC2	693_selected_genes
chr19	45867467	45867614	ERCC2	693_selected_genes
chr19	45867656	45867830	ERCC2	693_selected_genes
chr19	45868070	45868237	ERCC2	693_selected_genes
chr19	45868274	45868441	ERCC2	693_selected_genes
chr19	45871862	45872026	ERCC2	693_selected_genes
chr19	45872162	45872275	ERCC2	693_selected_genes
chr19	45872302	45872430	ERCC2	693_selected_genes
chr19	45873365	45873515	ERCC2	693_selected_genes
chr19	45873768	45873823	ERCC2	693_selected_genes
chr2	128015146	128015328	ERCC3	693_selected_genes
chr2	128016846	128017049	ERCC3	693_selected_genes
chr2	128018778	128018947	ERCC3	693_selected_genes
chr2	128028886	128029054	ERCC3	693_selected_genes
chr2	128030415	128030562	ERCC3	693_selected_genes
chr2	128036723	128036976	ERCC3	693_selected_genes
chr2	128037997	128038232	ERCC3	693_selected_genes
chr2	128044253	128044618	ERCC3	693_selected_genes
chr2	128046210	128046465	ERCC3	693_selected_genes
chr2	128046887	128047120	ERCC3	693_selected_genes
chr2	128047239	128047425	ERCC3	693_selected_genes
chr2	128047774	128047876	ERCC3	693_selected_genes
chr2	128050160	128050447	ERCC3	693_selected_genes
chr2	128051063	128051319	ERCC3	693_selected_genes
chr2	128051416	128051682	ERCC3	693_selected_genes
chr16	14013997	14014254	ERCC4	693_selected_genes
chr16	14015862	14016093	ERCC4	693_selected_genes
chr16	14020383	14020638	ERCC4	693_selected_genes
chr16	14021859	14022117	ERCC4	693_selected_genes
chr16	14024541	14024772	ERCC4	693_selected_genes
chr16	14025988	14026184	ERCC4	693_selected_genes
chr16	14028023	14028184	ERCC4	693_selected_genes
chr16	14028977	14029626	ERCC4	693_selected_genes
chr16	14031595	14031740	ERCC4	693_selected_genes
chr16	14037805	14037868	ERCC4	693_selected_genes
chr16	14038554	14038717	ERCC4	693_selected_genes
chr16	14041445	14042229	ERCC4	693_selected_genes
chr13	103498591	103498729	ERCC5	693_selected_genes
chr13	103501442	103501569	ERCC5	693_selected_genes
chr13	103504442	103504668	ERCC5	693_selected_genes
chr13	103506081	103506296	ERCC5	693_selected_genes
chr13	103506612	103506749	ERCC5	693_selected_genes
chr13	103508376	103508487	ERCC5	693_selected_genes
chr13	103510599	103510793	ERCC5	693_selected_genes
chr13	103511235	103511312	ERCC5	693_selected_genes
chr13	103513831	103514089	ERCC5	693_selected_genes
chr13	103514354	103515478	ERCC5	693_selected_genes
chr13	103517991	103518286	ERCC5	693_selected_genes
chr13	103518586	103518756	ERCC5	693_selected_genes
chr13	103518956	103519220	ERCC5	693_selected_genes
chr13	103520437	103520632	ERCC5	693_selected_genes
chr13	103524522	103524773	ERCC5	693_selected_genes
chr13	103525583	103525718	ERCC5	693_selected_genes
chr13	103527631	103528278	ERCC5	693_selected_genes
chr21	39739531	39739595	ERG	693_selected_genes
chr21	39755299	39755870	ERG	693_selected_genes
chr21	39762891	39762989	ERG	693_selected_genes
chr21	39763555	39763662	ERG	693_selected_genes
chr21	39764213	39764391	ERG	693_selected_genes
chr21	39772258	39772592	ERG	693_selected_genes
chr21	39774453	39774584	ERG	693_selected_genes
chr21	39775402	39775656	ERG	693_selected_genes
chr21	39795306	39795508	ERG	693_selected_genes
chr21	39817301	39817569	ERG	693_selected_genes
chr21	39870261	39870329	ERG	693_selected_genes
chr21	39947560	39947649	ERG	693_selected_genes
chr6	152129022	152129524	ESR1	693_selected_genes
chr6	152130216	152130432	ESR1	693_selected_genes
chr6	152163706	152163953	ESR1	693_selected_genes
chr6	152201764	152201931	ESR1	693_selected_genes
chr6	152265282	152265668	ESR1	693_selected_genes
chr6	152332765	152332954	ESR1	693_selected_genes
chr6	152382100	152382284	ESR1	693_selected_genes
chr6	152415494	152415728	ESR1	693_selected_genes
chr6	152419841	152420126	ESR1	693_selected_genes
chr6	152446375	152446508	ESR1	693_selected_genes
chr14	64551609	64551795	ESR2	693_selected_genes
chr14	64694225	64694357	ESR2	693_selected_genes
chr14	64694582	64694672	ESR2	693_selected_genes
chr14	64694719	64694788	ESR2	693_selected_genes

chr14	64699829	64700066	ESR2	693_selected_genes
chr14	64701649	64701893	ESR2	693_selected_genes
chr14	64716238	64716422	ESR2	693_selected_genes
chr14	64723918	64724107	ESR2	693_selected_genes
chr14	64727141	64727491	ESR2	693_selected_genes
chr14	64735487	64735654	ESR2	693_selected_genes
chr14	64746673	64746896	ESR2	693_selected_genes
chr14	64749316	64749728	ESR2	693_selected_genes
chr7	13935465	13935737	ETV1	693_selected_genes
chr7	13940337	13940489	ETV1	693_selected_genes
chr7	13946029	13946249	ETV1	693_selected_genes
chr7	13947392	13947506	ETV1	693_selected_genes
chr7	13949231	13949350	ETV1	693_selected_genes
chr7	13950838	13950957	ETV1	693_selected_genes
chr7	13971101	13971399	ETV1	693_selected_genes
chr7	13975307	13975546	ETV1	693_selected_genes
chr7	13978716	13978896	ETV1	693_selected_genes
chr7	14017026	14017130	ETV1	693_selected_genes
chr7	14025715	14025826	ETV1	693_selected_genes
chr7	14026232	14026335	ETV1	693_selected_genes
chr7	14027685	14027823	ETV1	693_selected_genes
chr7	14028279	14028416	ETV1	693_selected_genes
chr7	14028607	14028702	ETV1	693_selected_genes
chr17	41605861	41606136	ETV4	693_selected_genes
chr17	41606477	41606629	ETV4	693_selected_genes
chr17	41606846	41607069	ETV4	693_selected_genes
chr17	41607226	41607345	ETV4	693_selected_genes
chr17	41607449	41607574	ETV4	693_selected_genes
chr17	41610016	41610332	ETV4	693_selected_genes
chr17	41610529	41610741	ETV4	693_selected_genes
chr17	41611201	41611378	ETV4	693_selected_genes
chr17	41613768	41613872	ETV4	693_selected_genes
chr17	41622317	41622415	ETV4	693_selected_genes
chr17	41622616	41622760	ETV4	693_selected_genes
chr17	41622900	41623010	ETV4	693_selected_genes
chr3	185766402	185766674	ETV5	693_selected_genes
chr3	185769793	185769945	ETV5	693_selected_genes
chr3	185774838	185775058	ETV5	693_selected_genes
chr3	185775186	185775305	ETV5	693_selected_genes
chr3	185782180	185782290	ETV5	693_selected_genes
chr3	185783576	185783886	ETV5	693_selected_genes
chr3	185797580	185797918	ETV5	693_selected_genes
chr3	185798809	185798989	ETV5	693_selected_genes
chr3	185801932	185802094	ETV5	693_selected_genes
chr3	185823068	185823169	ETV5	693_selected_genes
chr3	185823212	185823310	ETV5	693_selected_genes
chr3	185823388	185823526	ETV5	693_selected_genes
chr3	185823587	185823756	ETV5	693_selected_genes
chr3	185826220	185826322	ETV5	693_selected_genes
chr12	11803036	11803119	ETV6	693_selected_genes
chr12	11905358	11905538	ETV6	693_selected_genes
chr12	11992048	11992263	ETV6	693_selected_genes
chr12	11993369	11993442	ETV6	693_selected_genes
chr12	12006335	12006520	ETV6	693_selected_genes
chr12	12022332	12022928	ETV6	693_selected_genes
chr12	12037353	12037546	ETV6	693_selected_genes
chr12	12038834	12038985	ETV6	693_selected_genes
chr12	12043849	12044005	ETV6	693_selected_genes
chr12	12044449	12044560	ETV6	693_selected_genes
chr22	29664300	29664363	EWSR1	693_selected_genes
chr22	29668179	29668266	EWSR1	693_selected_genes
chr22	29668343	29668445	EWSR1	693_selected_genes
chr22	29669701	29669878	EWSR1	693_selected_genes
chr22	29670228	29670296	EWSR1	693_selected_genes
chr22	29673993	29674230	EWSR1	693_selected_genes
chr22	29678353	29678571	EWSR1	693_selected_genes
chr22	29682885	29683148	EWSR1	693_selected_genes
chr22	29684569	29684800	EWSR1	693_selected_genes
chr22	29686376	29686517	EWSR1	693_selected_genes
chr22	29687525	29687613	EWSR1	693_selected_genes
chr22	29688100	29688183	EWSR1	693_selected_genes
chr22	29688451	29688620	EWSR1	693_selected_genes
chr22	29692203	29692383	EWSR1	693_selected_genes
chr22	29693791	29693964	EWSR1	693_selected_genes
chr22	29694697	29694910	EWSR1	693_selected_genes
chr22	29695159	29695346	EWSR1	693_selected_genes
chr22	29695563	29695866	EWSR1	693_selected_genes
chr22	29696086	29696176	EWSR1	693_selected_genes
chr8	118811925	118812161	EXT1	693_selected_genes
chr8	118816935	118817157	EXT1	693_selected_genes
chr8	118819430	118819641	EXT1	693_selected_genes
chr8	118825085	118825225	EXT1	693_selected_genes
chr8	118830648	118830794	EXT1	693_selected_genes
chr8	118831889	118832058	EXT1	693_selected_genes

chr8	118834678	118834861	EXT1	693_selected_genes
chr8	118842443	118842613	EXT1	693_selected_genes
chr8	118843812	118843917	EXT1	693_selected_genes
chr8	118847657	118847815	EXT1	693_selected_genes
chr8	118849321	118849465	EXT1	693_selected_genes
chr8	119122298	119123310	EXT1	693_selected_genes
chr11	44117777	44117896	EXT2	693_selected_genes
chr11	44129207	44129823	EXT2	693_selected_genes
chr11	44130718	44130858	EXT2	693_selected_genes
chr11	44135709	44135876	EXT2	693_selected_genes
chr11	44146313	44146559	EXT2	693_selected_genes
chr11	44148340	44148530	EXT2	693_selected_genes
chr11	44151569	44151713	EXT2	693_selected_genes
chr11	44165771	44165914	EXT2	693_selected_genes
chr11	44193135	44193317	EXT2	693_selected_genes
chr11	44219353	44219593	EXT2	693_selected_genes
chr11	44228317	44228534	EXT2	693_selected_genes
chr11	44253877	44254071	EXT2	693_selected_genes
chr11	44255639	44255818	EXT2	693_selected_genes
chr11	44257817	44257950	EXT2	693_selected_genes
chr11	44265673	44265862	EXT2	693_selected_genes
chr17	40854524	40854635	EZH1	693_selected_genes
chr17	40854869	40855004	EZH1	693_selected_genes
chr17	40855732	40855863	EZH1	693_selected_genes
chr17	40856594	40856726	EZH1	693_selected_genes
chr17	40857080	40857226	EZH1	693_selected_genes
chr17	40857999	40858228	EZH1	693_selected_genes
chr17	40859950	40860126	EZH1	693_selected_genes
chr17	40860868	40860959	EZH1	693_selected_genes
chr17	40861838	40861980	EZH1	693_selected_genes
chr17	40864281	40864528	EZH1	693_selected_genes
chr17	40865201	40865432	EZH1	693_selected_genes
chr17	40869968	40870110	EZH1	693_selected_genes
chr17	40870446	40870660	EZH1	693_selected_genes
chr17	40871097	40871250	EZH1	693_selected_genes
chr17	40872265	40872492	EZH1	693_selected_genes
chr17	40874787	40874958	EZH1	693_selected_genes
chr17	40876297	40876467	EZH1	693_selected_genes
chr17	40879627	40879806	EZH1	693_selected_genes
chr17	40880817	40880984	EZH1	693_selected_genes
chr7	148504712	148504823	EZH2	693_selected_genes
chr7	148506137	148506272	EZH2	693_selected_genes
chr7	148506376	148506507	EZH2	693_selected_genes
chr7	148507399	148507531	EZH2	693_selected_genes
chr7	148508691	148508837	EZH2	693_selected_genes
chr7	148511025	148511254	EZH2	693_selected_genes
chr7	148511980	148512156	EZH2	693_selected_genes
chr7	148512572	148512663	EZH2	693_selected_genes
chr7	148513750	148513895	EZH2	693_selected_genes
chr7	148514288	148514508	EZH2	693_selected_genes
chr7	148514943	148515234	EZH2	693_selected_genes
chr7	148516105	148516176	EZH2	693_selected_genes
chr7	148516662	148516804	EZH2	693_selected_genes
chr7	148523520	148523749	EZH2	693_selected_genes
chr7	148524230	148524383	EZH2	693_selected_genes
chr7	148525806	148525997	EZH2	693_selected_genes
chr7	148526794	148526965	EZH2	693_selected_genes
chr7	148529700	148529867	EZH2	693_selected_genes
chr7	148533888	148534117	EZH2	693_selected_genes
chr7	148543536	148543715	EZH2	693_selected_genes
chr7	148544248	148544415	EZH2	693_selected_genes
chr4	84383596	84384080	FAM175A	693_selected_genes
chr4	84384621	84384786	FAM175A	693_selected_genes
chr4	84388581	84388716	FAM175A	693_selected_genes
chr4	84390159	84390329	FAM175A	693_selected_genes
chr4	84391330	84391574	FAM175A	693_selected_genes
chr4	84393349	84393466	FAM175A	693_selected_genes
chr4	84397770	84397857	FAM175A	693_selected_genes
chr4	84401274	84401546	FAM175A	693_selected_genes
chr4	84403281	84403422	FAM175A	693_selected_genes
chr4	84405267	84405344	FAM175A	693_selected_genes
chr4	84406113	84406250	FAM175A	693_selected_genes
chr1	118165465	118166691	FAM46C	693_selected_genes
chr16	89804983	89805722	FANCA	693_selected_genes
chr16	89805860	89805986	FANCA	693_selected_genes
chr16	89806376	89806532	FANCA	693_selected_genes
chr16	89807186	89807299	FANCA	693_selected_genes
chr16	89809182	89809371	FANCA	693_selected_genes
chr16	89811341	89811504	FANCA	693_selected_genes
chr16	89812966	89813121	FANCA	693_selected_genes
chr16	89813213	89813323	FANCA	693_selected_genes
chr16	89815041	89815200	FANCA	693_selected_genes
chr16	89816112	89816335	FANCA	693_selected_genes
chr16	89818520	89818655	FANCA	693_selected_genes

chr16	89824959	89825138	FANCA	693_selected_genes
chr16	89828331	89828455	FANCA	693_selected_genes
chr16	89829128	89829226	FANCA	693_selected_genes
chr16	89831189	89831499	FANCA	693_selected_genes
chr16	89833523	89833670	FANCA	693_selected_genes
chr16	89836215	89836457	FANCA	693_selected_genes
chr16	89836548	89836692	FANCA	693_selected_genes
chr16	89836946	89837067	FANCA	693_selected_genes
chr16	89838060	89838247	FANCA	693_selected_genes
chr16	89839653	89839817	FANCA	693_selected_genes
chr16	89842124	89842248	FANCA	693_selected_genes
chr16	89844739	89844895	FANCA	693_selected_genes
chr16	89845183	89845283	FANCA	693_selected_genes
chr16	89845325	89845436	FANCA	693_selected_genes
chr16	89846251	89846390	FANCA	693_selected_genes
chr16	89849241	89849351	FANCA	693_selected_genes
chr16	89849389	89849535	FANCA	693_selected_genes
chr16	89851236	89851397	FANCA	693_selected_genes
chr16	89857785	89857969	FANCA	693_selected_genes
chr16	89858309	89858501	FANCA	693_selected_genes
chr16	89858853	89858980	FANCA	693_selected_genes
chr16	89862288	89862451	FANCA	693_selected_genes
chr16	89865440	89865665	FANCA	693_selected_genes
chr16	89865987	89866071	FANCA	693_selected_genes
chr16	89869641	89869774	FANCA	693_selected_genes
chr16	89871662	89871825	FANCA	693_selected_genes
chr16	89874676	89874800	FANCA	693_selected_genes
chr16	89877089	89877235	FANCA	693_selected_genes
chr16	89877311	89877504	FANCA	693_selected_genes
chr16	89880902	89881046	FANCA	693_selected_genes
chr16	89882259	89882419	FANCA	693_selected_genes
chr16	89882521	89883048	FANCA	693_selected_genes
chr9	97863963	97864157	FANCC	693_selected_genes
chr9	97869322	97869576	FANCC	693_selected_genes
chr9	97873452	97873652	FANCC	693_selected_genes
chr9	97873719	97873944	FANCC	693_selected_genes
chr9	97876885	97877017	FANCC	693_selected_genes
chr9	97879571	97879697	FANCC	693_selected_genes
chr9	97887342	97887492	FANCC	693_selected_genes
chr9	97888785	97888888	FANCC	693_selected_genes
chr9	97897602	97897809	FANCC	693_selected_genes
chr9	97912179	97912394	FANCC	693_selected_genes
chr9	97933335	97933450	FANCC	693_selected_genes
chr9	97934293	97934454	FANCC	693_selected_genes
chr9	98002905	98003050	FANCC	693_selected_genes
chr9	98009688	98009823	FANCC	693_selected_genes
chr9	98011383	98011598	FANCC	693_selected_genes
chr3	10070316	10070430	FANCD2	693_selected_genes
chr3	10073957	10074060	FANCD2	693_selected_genes
chr3	10074490	10074681	FANCD2	693_selected_genes
chr3	10076127	10076245	FANCD2	693_selected_genes
chr3	10076353	10076507	FANCD2	693_selected_genes
chr3	10076831	10076942	FANCD2	693_selected_genes
chr3	10077945	10078048	FANCD2	693_selected_genes
chr3	10080937	10081066	FANCD2	693_selected_genes
chr3	10081379	10081585	FANCD2	693_selected_genes
chr3	10082195	10082249	FANCD2	693_selected_genes
chr3	10083281	10083419	FANCD2	693_selected_genes
chr3	10084217	10084372	FANCD2	693_selected_genes
chr3	10084708	10084859	FANCD2	693_selected_genes
chr3	10085142	10085301	FANCD2	693_selected_genes
chr3	10085487	10085573	FANCD2	693_selected_genes
chr3	10088238	10088432	FANCD2	693_selected_genes
chr3	10089575	10089760	FANCD2	693_selected_genes
chr3	10091032	10091214	FANCD2	693_selected_genes
chr3	10094045	10094206	FANCD2	693_selected_genes
chr3	10101952	10102112	FANCD2	693_selected_genes
chr3	10103809	10103920	FANCD2	693_selected_genes
chr3	10105450	10105620	FANCD2	693_selected_genes
chr3	10106014	10106138	FANCD2	693_selected_genes
chr3	10106387	10106584	FANCD2	693_selected_genes
chr3	10107052	10107203	FANCD2	693_selected_genes
chr3	10107522	10107688	FANCD2	693_selected_genes
chr3	10108867	10109026	FANCD2	693_selected_genes
chr3	10114529	10114690	FANCD2	693_selected_genes
chr3	10114911	10115071	FANCD2	693_selected_genes
chr3	10116188	10116382	FANCD2	693_selected_genes
chr3	10119739	10119906	FANCD2	693_selected_genes
chr3	10122758	10122937	FANCD2	693_selected_genes
chr3	10123004	10123173	FANCD2	693_selected_genes
chr3	10127470	10127631	FANCD2	693_selected_genes
chr3	10128792	10128973	FANCD2	693_selected_genes
chr3	10130107	10130251	FANCD2	693_selected_genes
chr3	10130486	10130659	FANCD2	693_selected_genes

chr3	10131950	10132094	FANCD2	693_selected_genes
chr3	10133839	10133961	FANCD2	693_selected_genes
chr3	10134943	10135032	FANCD2	693_selected_genes
chr3	10135947	10136072	FANCD2	693_selected_genes
chr3	10136858	10136983	FANCD2	693_selected_genes
chr3	10137984	10138181	FANCD2	693_selected_genes
chr3	10140378	10140659	FANCD2	693_selected_genes
chr3	10142846	10142971	FANCD2	693_selected_genes
chr6	35420297	35420595	FANCE	693_selected_genes
chr6	35423498	35424155	FANCE	693_selected_genes
chr6	35425307	35425402	FANCE	693_selected_genes
chr6	35425667	35425786	FANCE	693_selected_genes
chr6	35426048	35426242	FANCE	693_selected_genes
chr6	35427082	35427256	FANCE	693_selected_genes
chr6	35427433	35427562	FANCE	693_selected_genes
chr6	35428303	35428420	FANCE	693_selected_genes
chr6	35430540	35430716	FANCE	693_selected_genes
chr6	35433995	35434147	FANCE	693_selected_genes
chr11	22646206	22647381	FANCF	693_selected_genes
chr9	35074079	35074238	FANCG	693_selected_genes
chr9	35074342	35074516	FANCG	693_selected_genes
chr9	35074898	35075104	FANCG	693_selected_genes
chr9	35075250	35075347	FANCG	693_selected_genes
chr9	35075436	35075776	FANCG	693_selected_genes
chr9	35075933	35076050	FANCG	693_selected_genes
chr9	35076403	35076605	FANCG	693_selected_genes
chr9	35076695	35076892	FANCG	693_selected_genes
chr9	35076942	35077123	FANCG	693_selected_genes
chr9	35077235	35077421	FANCG	693_selected_genes
chr9	35078112	35078365	FANCG	693_selected_genes
chr9	35078576	35078758	FANCG	693_selected_genes
chr9	35079122	35079263	FANCG	693_selected_genes
chr9	35079412	35079546	FANCG	693_selected_genes
chr15	89790853	89790987	FANCI	693_selected_genes
chr15	89801909	89802032	FANCI	693_selected_genes
chr15	89803918	89804099	FANCI	693_selected_genes
chr15	89804790	89804997	FANCI	693_selected_genes
chr15	89805026	89805134	FANCI	693_selected_genes
chr15	89806624	89806716	FANCI	693_selected_genes
chr15	89807108	89807293	FANCI	693_selected_genes
chr15	89807727	89807867	FANCI	693_selected_genes
chr15	89811604	89811799	FANCI	693_selected_genes
chr15	89816582	89816725	FANCI	693_selected_genes
chr15	89817373	89817560	FANCI	693_selected_genes
chr15	89819916	89820147	FANCI	693_selected_genes
chr15	89821892	89822030	FANCI	693_selected_genes
chr15	89824375	89824556	FANCI	693_selected_genes
chr15	89824970	89825091	FANCI	693_selected_genes
chr15	89826341	89826506	FANCI	693_selected_genes
chr15	89828301	89828474	FANCI	693_selected_genes
chr15	89833418	89833537	FANCI	693_selected_genes
chr15	89834818	89834970	FANCI	693_selected_genes
chr15	89835893	89836120	FANCI	693_selected_genes
chr15	89836147	89836319	FANCI	693_selected_genes
chr15	89837038	89837253	FANCI	693_selected_genes
chr15	89838120	89838350	FANCI	693_selected_genes
chr15	89843005	89843222	FANCI	693_selected_genes
chr15	89843505	89843641	FANCI	693_selected_genes
chr15	89844531	89844698	FANCI	693_selected_genes
chr15	89847069	89847171	FANCI	693_selected_genes
chr15	89848320	89848498	FANCI	693_selected_genes
chr15	89848546	89848665	FANCI	693_selected_genes
chr15	89848810	89848954	FANCI	693_selected_genes
chr15	89849212	89849450	FANCI	693_selected_genes
chr15	89850681	89850785	FANCI	693_selected_genes
chr15	89850818	89850928	FANCI	693_selected_genes
chr15	89856109	89856228	FANCI	693_selected_genes
chr15	89857817	89857963	FANCI	693_selected_genes
chr15	89858487	89858645	FANCI	693_selected_genes
chr15	89859602	89859715	FANCI	693_selected_genes
chr2	58386874	58386960	FANCL	693_selected_genes
chr2	58387217	58387339	FANCL	693_selected_genes
chr2	58388631	58388798	FANCL	693_selected_genes
chr2	58389975	58390107	FANCL	693_selected_genes
chr2	58390134	58390234	FANCL	693_selected_genes
chr2	58390543	58390677	FANCL	693_selected_genes
chr2	58392833	58393034	FANCL	693_selected_genes
chr2	58421338	58421433	FANCL	693_selected_genes
chr2	58425688	58425822	FANCL	693_selected_genes
chr2	58431239	58431386	FANCL	693_selected_genes
chr2	58449051	58449202	FANCL	693_selected_genes
chr2	58453837	58453944	FANCL	693_selected_genes
chr2	58456923	58457034	FANCL	693_selected_genes
chr2	58459163	58459272	FANCL	693_selected_genes

chr2	58468327	58468473	FANCL	693_selected_genes
chr14	45605209	45605767	FANCM	693_selected_genes
chr14	45606246	45606469	FANCM	693_selected_genes
chr14	45609809	45609937	FANCM	693_selected_genes
chr14	45618014	45618223	FANCM	693_selected_genes
chr14	45620574	45620756	FANCM	693_selected_genes
chr14	45623097	45623280	FANCM	693_selected_genes
chr14	45623874	45624050	FANCM	693_selected_genes
chr14	45624550	45624687	FANCM	693_selected_genes
chr14	45628273	45628508	FANCM	693_selected_genes
chr14	45633536	45633793	FANCM	693_selected_genes
chr14	45636127	45636399	FANCM	693_selected_genes
chr14	45639766	45639974	FANCM	693_selected_genes
chr14	45642232	45642438	FANCM	693_selected_genes
chr14	45644248	45646204	FANCM	693_selected_genes
chr14	45650607	45650752	FANCM	693_selected_genes
chr14	45650814	45650933	FANCM	693_selected_genes
chr14	45652951	45653130	FANCM	693_selected_genes
chr14	45654394	45654601	FANCM	693_selected_genes
chr14	45656958	45657115	FANCM	693_selected_genes
chr14	45657979	45658590	FANCM	693_selected_genes
chr14	45664658	45664813	FANCM	693_selected_genes
chr14	45665349	45665775	FANCM	693_selected_genes
chr14	45667821	45668163	FANCM	693_selected_genes
chr14	45669047	45669236	FANCM	693_selected_genes
chr10	90750608	90750688	FAS	693_selected_genes
chr10	90762760	90762976	FAS	693_selected_genes
chr10	90767431	90767619	FAS	693_selected_genes
chr10	90768620	90768779	FAS	693_selected_genes
chr10	90770270	90770382	FAS	693_selected_genes
chr10	90770484	90770597	FAS	693_selected_genes
chr10	90771730	90771863	FAS	693_selected_genes
chr10	90773074	90773149	FAS	693_selected_genes
chr10	90773831	90774232	FAS	693_selected_genes
chr4	187509720	187510399	FAT1	693_selected_genes
chr4	187511496	187511582	FAT1	693_selected_genes
chr4	187511674	187511874	FAT1	693_selected_genes
chr4	187513821	187513931	FAT1	693_selected_genes
chr4	187516817	187517005	FAT1	693_selected_genes
chr4	187517668	187518350	FAT1	693_selected_genes
chr4	187518810	187518971	FAT1	693_selected_genes
chr4	187519100	187519304	FAT1	693_selected_genes
chr4	187521026	187521539	FAT1	693_selected_genes
chr4	187522397	187522605	FAT1	693_selected_genes
chr4	187524031	187524213	FAT1	693_selected_genes
chr4	187524304	187525156	FAT1	693_selected_genes
chr4	187525505	187525753	FAT1	693_selected_genes
chr4	187527198	187527392	FAT1	693_selected_genes
chr4	187530311	187530499	FAT1	693_selected_genes
chr4	187530929	187531194	FAT1	693_selected_genes
chr4	187532514	187532954	FAT1	693_selected_genes
chr4	187534237	187534521	FAT1	693_selected_genes
chr4	187535319	187535523	FAT1	693_selected_genes
chr4	187538133	187538380	FAT1	693_selected_genes
chr4	187538836	187542954	FAT1	693_selected_genes
chr4	187549282	187549543	FAT1	693_selected_genes
chr4	187549616	187549942	FAT1	693_selected_genes
chr4	187554812	187555002	FAT1	693_selected_genes
chr4	187557153	187557414	FAT1	693_selected_genes
chr4	187557713	187558093	FAT1	693_selected_genes
chr4	187560850	187560962	FAT1	693_selected_genes
chr4	187584427	187584792	FAT1	693_selected_genes
chr4	187627691	187631006	FAT1	693_selected_genes
chr11	92085253	92088595	FAT3	693_selected_genes
chr11	92257774	92258139	FAT3	693_selected_genes
chr11	92292550	92292758	FAT3	693_selected_genes
chr11	92430524	92430636	FAT3	693_selected_genes
chr11	92494996	92495361	FAT3	693_selected_genes
chr11	92498019	92498280	FAT3	693_selected_genes
chr11	92507181	92507371	FAT3	693_selected_genes
chr11	92523083	92523409	FAT3	693_selected_genes
chr11	92525907	92526168	FAT3	693_selected_genes
chr11	92530976	92535100	FAT3	693_selected_genes
chr11	92538293	92538540	FAT3	693_selected_genes
chr11	92539502	92539706	FAT3	693_selected_genes
chr11	92542983	92543267	FAT3	693_selected_genes
chr11	92564762	92565202	FAT3	693_selected_genes
chr11	92568010	92568275	FAT3	693_selected_genes
chr11	92569706	92569894	FAT3	693_selected_genes
chr11	92570803	92570997	FAT3	693_selected_genes
chr11	92573702	92573950	FAT3	693_selected_genes
chr11	92577074	92577923	FAT3	693_selected_genes
chr11	92590354	92590539	FAT3	693_selected_genes
chr11	92592305	92592513	FAT3	693_selected_genes

chr11	92599881	92600400	FAT3	693_selected_genes
chr11	92613871	92614075	FAT3	693_selected_genes
chr11	92615878	92616584	FAT3	693_selected_genes
chr11	92620140	92620304	FAT3	693_selected_genes
chr11	92622329	92622439	FAT3	693_selected_genes
chr11	92622996	92623082	FAT3	693_selected_genes
chr11	92623631	92624304	FAT3	693_selected_genes
chr15	48703161	48703601	FBN1	693_selected_genes
chr15	48704740	48704965	FBN1	693_selected_genes
chr15	48707707	48707989	FBN1	693_selected_genes
chr15	48712858	48713028	FBN1	693_selected_genes
chr15	48713729	48713908	FBN1	693_selected_genes
chr15	48714123	48714290	FBN1	693_selected_genes
chr15	48717540	48717713	FBN1	693_selected_genes
chr15	48717910	48718086	FBN1	693_selected_genes
chr15	48719738	48719995	FBN1	693_selected_genes
chr15	48720053	48720165	FBN1	693_selected_genes
chr15	48720517	48720693	FBN1	693_selected_genes
chr15	48722842	48723024	FBN1	693_selected_genes
chr15	48725037	48725210	FBN1	693_selected_genes
chr15	48726765	48726935	FBN1	693_selected_genes
chr15	48729132	48729299	FBN1	693_selected_genes
chr15	48729493	48729609	FBN1	693_selected_genes
chr15	48729939	48730139	FBN1	693_selected_genes
chr15	48733892	48734068	FBN1	693_selected_genes
chr15	48736712	48736882	FBN1	693_selected_genes
chr15	48737547	48737726	FBN1	693_selected_genes
chr15	48738877	48739044	FBN1	693_selected_genes
chr15	48740939	48741115	FBN1	693_selected_genes
chr15	48744733	48744906	FBN1	693_selected_genes
chr15	48748808	48748984	FBN1	693_selected_genes
chr15	48752417	48752539	FBN1	693_selected_genes
chr15	48755253	48755462	FBN1	693_selected_genes
chr15	48756070	48756243	FBN1	693_selected_genes
chr15	48757739	48757915	FBN1	693_selected_genes
chr15	48757961	48758080	FBN1	693_selected_genes
chr15	48760109	48760324	FBN1	693_selected_genes
chr15	48760583	48760756	FBN1	693_selected_genes
chr15	48762805	48762978	FBN1	693_selected_genes
chr15	48764722	48764898	FBN1	693_selected_genes
chr15	48766426	48766599	FBN1	693_selected_genes
chr15	48766699	48766872	FBN1	693_selected_genes
chr15	48773826	48774002	FBN1	693_selected_genes
chr15	48775989	48776165	FBN1	693_selected_genes
chr15	48777545	48777718	FBN1	693_selected_genes
chr15	48779246	48779422	FBN1	693_selected_genes
chr15	48779483	48779659	FBN1	693_selected_genes
chr15	48780284	48780463	FBN1	693_selected_genes
chr15	48780539	48780715	FBN1	693_selected_genes
chr15	48782022	48782300	FBN1	693_selected_genes
chr15	48784632	48784808	FBN1	693_selected_genes
chr15	48786375	48786476	FBN1	693_selected_genes
chr15	48787294	48787482	FBN1	693_selected_genes
chr15	48787640	48787810	FBN1	693_selected_genes
chr15	48788271	48788447	FBN1	693_selected_genes
chr15	48789437	48789613	FBN1	693_selected_genes
chr15	48791156	48791260	FBN1	693_selected_genes
chr15	48795958	48796161	FBN1	693_selected_genes
chr15	48797196	48797369	FBN1	693_selected_genes
chr15	48800753	48800926	FBN1	693_selected_genes
chr15	48802215	48802391	FBN1	693_selected_genes
chr15	48805720	48805890	FBN1	693_selected_genes
chr15	48807558	48807749	FBN1	693_selected_genes
chr15	48808354	48808584	FBN1	693_selected_genes
chr15	48812830	48813039	FBN1	693_selected_genes
chr15	48818301	48818477	FBN1	693_selected_genes
chr15	48826251	48826427	FBN1	693_selected_genes
chr15	48829782	48830030	FBN1	693_selected_genes
chr15	48888454	48888600	FBN1	693_selected_genes
chr15	48892310	48892456	FBN1	693_selected_genes
chr15	48902899	48903048	FBN1	693_selected_genes
chr15	48905181	48905314	FBN1	693_selected_genes
chr15	48936773	48936991	FBN1	693_selected_genes
chr2	48016549	48016604	FBXO11	693_selected_genes
chr2	48035231	48035411	FBXO11	693_selected_genes
chr2	48035442	48035591	FBXO11	693_selected_genes
chr2	48036271	48036430	FBXO11	693_selected_genes
chr2	48036713	48036871	FBXO11	693_selected_genes
chr2	48037429	48037590	FBXO11	693_selected_genes
chr2	48040261	48040541	FBXO11	693_selected_genes
chr2	48040904	48041031	FBXO11	693_selected_genes
chr2	48045892	48046028	FBXO11	693_selected_genes
chr2	48046069	48046242	FBXO11	693_selected_genes
chr2	48047475	48047620	FBXO11	693_selected_genes

chr2	48049331	48049467	FBXO11	693_selected_genes
chr2	48050256	48050524	FBXO11	693_selected_genes
chr2	48059462	48059650	FBXO11	693_selected_genes
chr2	48059685	48059842	FBXO11	693_selected_genes
chr2	48059882	48060044	FBXO11	693_selected_genes
chr2	48060077	48060234	FBXO11	693_selected_genes
chr2	48061692	48061875	FBXO11	693_selected_genes
chr2	48061901	48062035	FBXO11	693_selected_genes
chr2	48062985	48063165	FBXO11	693_selected_genes
chr2	48064101	48064200	FBXO11	693_selected_genes
chr2	48065972	48066167	FBXO11	693_selected_genes
chr2	48066532	48066664	FBXO11	693_selected_genes
chr2	48066755	48066933	FBXO11	693_selected_genes
chr2	48132602	48132884	FBXO11	693_selected_genes
chr4	153244007	153244326	FBXW7	693_selected_genes
chr4	153245310	153245571	FBXW7	693_selected_genes
chr4	153247132	153247408	FBXW7	693_selected_genes
chr4	153249334	153249566	FBXW7	693_selected_genes
chr4	153250798	153250962	FBXW7	693_selected_genes
chr4	153251858	153252045	FBXW7	693_selected_genes
chr4	153253722	153253896	FBXW7	693_selected_genes
chr4	153258928	153259113	FBXW7	693_selected_genes
chr4	153268056	153268248	FBXW7	693_selected_genes
chr4	153269800	153269906	FBXW7	693_selected_genes
chr4	153271168	153271301	FBXW7	693_selected_genes
chr4	153273596	153273907	FBXW7	693_selected_genes
chr4	153303315	153303512	FBXW7	693_selected_genes
chr4	153332399	153332980	FBXW7	693_selected_genes
chr15	91428250	91428840	FES	693_selected_genes
chr15	91430165	91430312	FES	693_selected_genes
chr15	91430391	91430625	FES	693_selected_genes
chr15	91432510	91432698	FES	693_selected_genes
chr15	91432721	91432891	FES	693_selected_genes
chr15	91433044	91433217	FES	693_selected_genes
chr15	91433296	91433533	FES	693_selected_genes
chr15	91433570	91433739	FES	693_selected_genes
chr15	91434186	91434446	FES	693_selected_genes
chr15	91434704	91434931	FES	693_selected_genes
chr15	91435262	91435366	FES	693_selected_genes
chr15	91435911	91436080	FES	693_selected_genes
chr15	91436304	91436449	FES	693_selected_genes
chr15	91436476	91436669	FES	693_selected_genes
chr15	91436858	91437066	FES	693_selected_genes
chr15	91437140	91437313	FES	693_selected_genes
chr15	91438620	91438813	FES	693_selected_genes
chr5	44305071	44305319	FGF10	693_selected_genes
chr5	44310503	44310657	FGF10	693_selected_genes
chr5	44388434	44388809	FGF10	693_selected_genes
chr3	191861772	191861941	FGF12	693_selected_genes
chr3	191888221	191888470	FGF12	693_selected_genes
chr3	191986414	191986563	FGF12	693_selected_genes
chr3	192053124	192053278	FGF12	693_selected_genes
chr3	192078191	192078352	FGF12	693_selected_genes
chr3	192125788	192126037	FGF12	693_selected_genes
chr3	192444944	192445007	FGF12	693_selected_genes
chr13	102375155	102375342	FGF14	693_selected_genes
chr13	102378936	102379185	FGF14	693_selected_genes
chr13	102521049	102521203	FGF14	693_selected_genes
chr13	102527510	102527671	FGF14	693_selected_genes
chr13	102568777	102569020	FGF14	693_selected_genes
chr13	103053795	103054053	FGF14	693_selected_genes
chr11	69514004	69514369	FGF19	693_selected_genes
chr11	69518003	69518157	FGF19	693_selected_genes
chr11	69518387	69518669	FGF19	693_selected_genes
chr12	4479483	4479974	FGF23	693_selected_genes
chr12	4481734	4481888	FGF23	693_selected_genes
chr12	4488512	4488773	FGF23	693_selected_genes
chr11	69625047	69625493	FGF3	693_selected_genes
chr11	69631062	69631216	FGF3	693_selected_genes
chr11	69633456	69633726	FGF3	693_selected_genes
chr11	69588051	69588278	FGF4	693_selected_genes
chr11	69588766	69588920	FGF4	693_selected_genes
chr11	69589487	69589877	FGF4	693_selected_genes
chr12	4537535	4537594	FGF6	693_selected_genes
chr12	4543355	4543582	FGF6	693_selected_genes
chr12	4553273	4553427	FGF6	693_selected_genes
chr12	4554365	4554761	FGF6	693_selected_genes
chr15	49716469	49716813	FGF7	693_selected_genes
chr15	49775322	49775476	FGF7	693_selected_genes
chr15	49776481	49776726	FGF7	693_selected_genes
chr4	15937525	15938280	FGFBP1	693_selected_genes
chr8	38271120	38271347	FGFR1	693_selected_genes
chr8	38271410	38271577	FGFR1	693_selected_genes
chr8	38271644	38271832	FGFR1	693_selected_genes

chr8	38272051	38272172	FGFR1	693_selected_genes
chr8	38272271	38272444	FGFR1	693_selected_genes
chr8	38273362	38273603	FGFR1	693_selected_genes
chr8	38274798	38274959	FGFR1	693_selected_genes
chr8	38275362	38275534	FGFR1	693_selected_genes
chr8	38275720	38275916	FGFR1	693_selected_genes
chr8	38277025	38277278	FGFR1	693_selected_genes
chr8	38279289	38279484	FGFR1	693_selected_genes
chr8	38280517	38280718	FGFR1	693_selected_genes
chr8	38282001	38282242	FGFR1	693_selected_genes
chr8	38283614	38283788	FGFR1	693_selected_genes
chr8	38285413	38285636	FGFR1	693_selected_genes
chr8	38285838	38285987	FGFR1	693_selected_genes
chr8	38286788	38286933	FGFR1	693_selected_genes
chr8	38287174	38287491	FGFR1	693_selected_genes
chr8	38297798	38297915	FGFR1	693_selected_genes
chr8	38314848	38315077	FGFR1	693_selected_genes
chr8	38318588	38318649	FGFR1	693_selected_genes
chr10	123239069	123239209	FGFR2	693_selected_genes
chr10	123239345	123239652	FGFR2	693_selected_genes
chr10	123241660	123241716	FGFR2	693_selected_genes
chr10	123243186	123243342	FGFR2	693_selected_genes
chr10	123244883	123245071	FGFR2	693_selected_genes
chr10	123246842	123246963	FGFR2	693_selected_genes
chr10	123247479	123247652	FGFR2	693_selected_genes
chr10	123256020	123256261	FGFR2	693_selected_genes
chr10	123257983	123258144	FGFR2	693_selected_genes
chr10	123260314	123260486	FGFR2	693_selected_genes
chr10	123263278	123263480	FGFR2	693_selected_genes
chr10	123274605	123274858	FGFR2	693_selected_genes
chr10	123276807	123277002	FGFR2	693_selected_genes
chr10	123278170	123278368	FGFR2	693_selected_genes
chr10	123279467	123279708	FGFR2	693_selected_genes
chr10	123285643	123285733	FGFR2	693_selected_genes
chr10	123286217	123286291	FGFR2	693_selected_genes
chr10	123297882	123297949	FGFR2	693_selected_genes
chr10	123298080	123298254	FGFR2	693_selected_genes
chr10	123310778	123310998	FGFR2	693_selected_genes
chr10	123323990	123324118	FGFR2	693_selected_genes
chr10	123324926	123325243	FGFR2	693_selected_genes
chr10	123353197	123353356	FGFR2	693_selected_genes
chr4	1795636	1795795	FGFR3	693_selected_genes
chr4	1800955	1801275	FGFR3	693_selected_genes
chr4	1801448	1801564	FGFR3	693_selected_genes
chr4	1803068	1803288	FGFR3	693_selected_genes
chr4	1803321	1803495	FGFR3	693_selected_genes
chr4	1803536	1803777	FGFR3	693_selected_genes
chr4	1804615	1804816	FGFR3	693_selected_genes
chr4	1805393	1805588	FGFR3	693_selected_genes
chr4	1806031	1806272	FGFR3	693_selected_genes
chr4	1806522	1806721	FGFR3	693_selected_genes
chr4	1807056	1807228	FGFR3	693_selected_genes
chr4	1807260	1807421	FGFR3	693_selected_genes
chr4	1807451	1807692	FGFR3	693_selected_genes
chr4	1807752	1807925	FGFR3	693_selected_genes
chr4	1807958	1808079	FGFR3	693_selected_genes
chr4	1808247	1808435	FGFR3	693_selected_genes
chr4	1808530	1808686	FGFR3	693_selected_genes
chr4	1808817	1809040	FGFR3	693_selected_genes
chr5	176516578	176516719	FGFR4	693_selected_genes
chr5	176516833	176516966	FGFR4	693_selected_genes
chr5	176517365	176517679	FGFR4	693_selected_genes
chr5	176517720	176517851	FGFR4	693_selected_genes
chr5	176517913	176518130	FGFR4	693_selected_genes
chr5	176518660	176518834	FGFR4	693_selected_genes
chr5	176519296	176519537	FGFR4	693_selected_genes
chr5	176519621	176519810	FGFR4	693_selected_genes
chr5	176520113	176520577	FGFR4	693_selected_genes
chr5	176520629	176520801	FGFR4	693_selected_genes
chr5	176522305	176522466	FGFR4	693_selected_genes
chr5	176522508	176522749	FGFR4	693_selected_genes
chr5	176523032	176523205	FGFR4	693_selected_genes
chr5	176523262	176523383	FGFR4	693_selected_genes
chr5	176523579	176523767	FGFR4	693_selected_genes
chr5	176524267	176524423	FGFR4	693_selected_genes
chr5	176524502	176524702	FGFR4	693_selected_genes
chr1	241661102	241661295	FH	693_selected_genes
chr1	241663711	241663915	FH	693_selected_genes
chr1	241665717	241665895	FH	693_selected_genes
chr1	241667316	241667570	FH	693_selected_genes
chr1	241669277	241669493	FH	693_selected_genes
chr1	241671877	241672110	FH	693_selected_genes
chr1	241675241	241675468	FH	693_selected_genes
chr1	241676877	241677038	FH	693_selected_genes

chr1	241680456	241680641	FH	693_selected_genes
chr1	241682865	241683047	FH	693_selected_genes
chr17	17116943	17117195	FLCN	693_selected_genes
chr17	17118273	17118429	FLCN	693_selected_genes
chr17	17118473	17118655	FLCN	693_selected_genes
chr17	17119668	17119842	FLCN	693_selected_genes
chr17	17120357	17120521	FLCN	693_selected_genes
chr17	17122307	17122548	FLCN	693_selected_genes
chr17	17124667	17124967	FLCN	693_selected_genes
chr17	17125789	17126000	FLCN	693_selected_genes
chr17	17127210	17127482	FLCN	693_selected_genes
chr17	17129266	17129661	FLCN	693_selected_genes
chr17	17131177	17131476	FLCN	693_selected_genes
chr13	28877278	28877530	FLT1	693_selected_genes
chr13	28880789	28880934	FLT1	693_selected_genes
chr13	28882954	28883089	FLT1	693_selected_genes
chr13	28885701	28885894	FLT1	693_selected_genes
chr13	28886104	28886260	FLT1	693_selected_genes
chr13	28891609	28891759	FLT1	693_selected_genes
chr13	28893534	28893696	FLT1	693_selected_genes
chr13	28895574	28895747	FLT1	693_selected_genes
chr13	28896373	28896521	FLT1	693_selected_genes
chr13	28896567	28896639	FLT1	693_selected_genes
chr13	28896901	28897108	FLT1	693_selected_genes
chr13	28901573	28901712	FLT1	693_selected_genes
chr13	28903726	28903890	FLT1	693_selected_genes
chr13	28908136	28908291	FLT1	693_selected_genes
chr13	28913279	28913462	FLT1	693_selected_genes
chr13	28919556	28919713	FLT1	693_selected_genes
chr13	28931665	28931847	FLT1	693_selected_genes
chr13	28942689	28942825	FLT1	693_selected_genes
chr13	28958996	28959193	FLT1	693_selected_genes
chr13	28963812	28964266	FLT1	693_selected_genes
chr13	28971071	28971230	FLT1	693_selected_genes
chr13	28973155	28973280	FLT1	693_selected_genes
chr13	28979891	28980056	FLT1	693_selected_genes
chr13	29001270	29001480	FLT1	693_selected_genes
chr13	29001863	29002083	FLT1	693_selected_genes
chr13	29004161	29004329	FLT1	693_selected_genes
chr13	29005247	29005472	FLT1	693_selected_genes
chr13	29007930	29008117	FLT1	693_selected_genes
chr13	29008169	29008382	FLT1	693_selected_genes
chr13	29012332	29012507	FLT1	693_selected_genes
chr13	29041014	29041291	FLT1	693_selected_genes
chr13	29041632	29041779	FLT1	693_selected_genes
chr13	29068891	29069005	FLT1	693_selected_genes
chr13	28578163	28578336	FLT3	693_selected_genes
chr13	28588563	28588719	FLT3	693_selected_genes
chr13	28589268	28589418	FLT3	693_selected_genes
chr13	28589701	28589863	FLT3	693_selected_genes
chr13	28592569	28592751	FLT3	693_selected_genes
chr13	28597461	28597639	FLT3	693_selected_genes
chr13	28598972	28599105	FLT3	693_selected_genes
chr13	28599514	28599577	FLT3	693_selected_genes
chr13	28601199	28601403	FLT3	693_selected_genes
chr13	28602289	28602450	FLT3	693_selected_genes
chr13	28607998	28608153	FLT3	693_selected_genes
chr13	28608193	28608376	FLT3	693_selected_genes
chr13	28608412	28608569	FLT3	693_selected_genes
chr13	28609606	28609835	FLT3	693_selected_genes
chr13	28610046	28610205	FLT3	693_selected_genes
chr13	28611296	28611450	FLT3	693_selected_genes
chr13	28622386	28622605	FLT3	693_selected_genes
chr13	28623495	28623699	FLT3	693_selected_genes
chr13	28623746	28623936	FLT3	693_selected_genes
chr13	28624206	28624384	FLT3	693_selected_genes
chr13	28626656	28626836	FLT3	693_selected_genes
chr13	28631458	28631624	FLT3	693_selected_genes
chr13	28635978	28636231	FLT3	693_selected_genes
chr13	28644602	28644774	FLT3	693_selected_genes
chr13	28674579	28674672	FLT3	693_selected_genes
chr5	180030166	180030415	FLT4	693_selected_genes
chr5	180035106	180035184	FLT4	693_selected_genes
chr5	180035255	180035309	FLT4	693_selected_genes
chr5	180035942	180036078	FLT4	693_selected_genes
chr5	180036879	180037050	FLT4	693_selected_genes
chr5	180038305	180038504	FLT4	693_selected_genes
chr5	180039480	180039636	FLT4	693_selected_genes
chr5	180039985	180040135	FLT4	693_selected_genes
chr5	180041042	180041204	FLT4	693_selected_genes
chr5	180043341	180043514	FLT4	693_selected_genes
chr5	180043874	180044019	FLT4	693_selected_genes
chr5	180045744	180045945	FLT4	693_selected_genes
chr5	180045995	180046134	FLT4	693_selected_genes

chr5	180046227	180046391	FLT4	693_selected_genes
chr5	180046639	180046794	FLT4	693_selected_genes
chr5	180047147	180047333	FLT4	693_selected_genes
chr5	180047583	180047740	FLT4	693_selected_genes
chr5	180047850	180048032	FLT4	693_selected_genes
chr5	180048080	180048277	FLT4	693_selected_genes
chr5	180048516	180048929	FLT4	693_selected_genes
chr5	180049705	180049864	FLT4	693_selected_genes
chr5	180050909	180051086	FLT4	693_selected_genes
chr5	180052843	180053056	FLT4	693_selected_genes
chr5	180053085	180053290	FLT4	693_selected_genes
chr5	180055856	180056024	FLT4	693_selected_genes
chr5	180056233	180056452	FLT4	693_selected_genes
chr5	180056670	180056860	FLT4	693_selected_genes
chr5	180056917	180057130	FLT4	693_selected_genes
chr5	180057199	180057362	FLT4	693_selected_genes
chr5	180057529	180057824	FLT4	693_selected_genes
chr5	180058656	180058803	FLT4	693_selected_genes
chr5	180076462	180076570	FLT4	693_selected_genes
chr2	216226252	216226374	FN1	693_selected_genes
chr2	216226666	216226827	FN1	693_selected_genes
chr2	216229576	216229733	FN1	693_selected_genes
chr2	216230202	216230378	FN1	693_selected_genes
chr2	216232560	216232775	FN1	693_selected_genes
chr2	216234991	216235180	FN1	693_selected_genes
chr2	216236606	216237123	FN1	693_selected_genes
chr2	216238019	216238159	FN1	693_selected_genes
chr2	216239911	216240141	FN1	693_selected_genes
chr2	216240326	216240466	FN1	693_selected_genes
chr2	216241195	216241422	FN1	693_selected_genes
chr2	216242872	216243010	FN1	693_selected_genes
chr2	216243827	216244065	FN1	693_selected_genes
chr2	216245508	216245828	FN1	693_selected_genes
chr2	216246909	216247073	FN1	693_selected_genes
chr2	216248025	216248231	FN1	693_selected_genes
chr2	216248717	216248932	FN1	693_selected_genes
chr2	216249557	216249724	FN1	693_selected_genes
chr2	216251386	216251706	FN1	693_selected_genes
chr2	216252909	216253049	FN1	693_selected_genes
chr2	216256329	216256562	FN1	693_selected_genes
chr2	216257628	216257951	FN1	693_selected_genes
chr2	216259225	216259467	FN1	693_selected_genes
chr2	216261834	216261971	FN1	693_selected_genes
chr2	216262377	216262596	FN1	693_selected_genes
chr2	216263954	216264099	FN1	693_selected_genes
chr2	216269086	216269403	FN1	693_selected_genes
chr2	216270935	216271258	FN1	693_selected_genes
chr2	216271824	216272069	FN1	693_selected_genes
chr2	216272805	216272945	FN1	693_selected_genes
chr2	216272995	216273174	FN1	693_selected_genes
chr2	216274260	216274487	FN1	693_selected_genes
chr2	216274631	216274862	FN1	693_selected_genes
chr2	216279501	216279706	FN1	693_selected_genes
chr2	216283939	216284133	FN1	693_selected_genes
chr2	216285370	216285549	FN1	693_selected_genes
chr2	216286788	216286991	FN1	693_selected_genes
chr2	216288047	216288274	FN1	693_selected_genes
chr2	216288843	216289073	FN1	693_selected_genes
chr2	216289791	216290033	FN1	693_selected_genes
chr2	216292877	216293086	FN1	693_selected_genes
chr2	216295412	216295600	FN1	693_selected_genes
chr2	216296530	216296712	FN1	693_selected_genes
chr2	216298021	216298209	FN1	693_selected_genes
chr2	216299393	216299572	FN1	693_selected_genes
chr2	216300352	216300550	FN1	693_selected_genes
chr14	38060544	38061941	FOXA1	693_selected_genes
chr14	38063317	38063406	FOXA1	693_selected_genes
chr14	38064080	38064202	FOXA1	693_selected_genes
chr20	22562462	22563817	FOXA2	693_selected_genes
chr20	22564804	22564941	FOXA2	693_selected_genes
chr9	100616171	100617343	FOXE1	693_selected_genes
chr3	138664408	138665589	FOXL2	693_selected_genes
chr3	71008372	71008567	FOXP1	693_selected_genes
chr3	71015015	71015232	FOXP1	693_selected_genes
chr3	71019861	71019981	FOXP1	693_selected_genes
chr3	71021136	71021356	FOXP1	693_selected_genes
chr3	71021680	71021852	FOXP1	693_selected_genes
chr3	71026066	71026218	FOXP1	693_selected_genes
chr3	71026768	71026898	FOXP1	693_selected_genes
chr3	71026953	71027205	FOXP1	693_selected_genes
chr3	71037119	71037253	FOXP1	693_selected_genes
chr3	71050097	71050235	FOXP1	693_selected_genes
chr3	71064674	71064829	FOXP1	693_selected_genes
chr3	71090453	71090708	FOXP1	693_selected_genes

chr3	71096062	71096271	FOXP1	693_selected_genes
chr3	71101662	71101802	FOXP1	693_selected_genes
chr3	71102761	71102949	FOXP1	693_selected_genes
chr3	71161661	71161813	FOXP1	693_selected_genes
chr3	71179623	71179859	FOXP1	693_selected_genes
chr3	71247029	71247244	FOXP1	693_selected_genes
chr3	71247327	71247557	FOXP1	693_selected_genes
chr6	1312914	1314176	FOXQ1	693_selected_genes
chr1	78413173	78413262	FUBP1	693_selected_genes
chr1	78414425	78414484	FUBP1	693_selected_genes
chr1	78414532	78414624	FUBP1	693_selected_genes
chr1	78414814	78415010	FUBP1	693_selected_genes
chr1	78420914	78421039	FUBP1	693_selected_genes
chr1	78422231	78422410	FUBP1	693_selected_genes
chr1	78425843	78425973	FUBP1	693_selected_genes
chr1	78426003	78426205	FUBP1	693_selected_genes
chr1	78428429	78428640	FUBP1	693_selected_genes
chr1	78429233	78429425	FUBP1	693_selected_genes
chr1	78429721	78429872	FUBP1	693_selected_genes
chr1	78429913	78430066	FUBP1	693_selected_genes
chr1	78430305	78430457	FUBP1	693_selected_genes
chr1	78430529	78430678	FUBP1	693_selected_genes
chr1	78430727	78430940	FUBP1	693_selected_genes
chr1	78432352	78432460	FUBP1	693_selected_genes
chr1	78432542	78432664	FUBP1	693_selected_genes
chr1	78432707	78432810	FUBP1	693_selected_genes
chr1	78433285	78433375	FUBP1	693_selected_genes
chr1	78433823	78433912	FUBP1	693_selected_genes
chr1	78434680	78434793	FUBP1	693_selected_genes
chr1	78435583	78435724	FUBP1	693_selected_genes
chr1	78444543	78444713	FUBP1	693_selected_genes
chr5	161112970	161113058	GABRA6	693_selected_genes
chr5	161113210	161113379	GABRA6	693_selected_genes
chr5	161113916	161114034	GABRA6	693_selected_genes
chr5	161114460	161114592	GABRA6	693_selected_genes
chr5	161115929	161116200	GABRA6	693_selected_genes
chr5	161116234	161116367	GABRA6	693_selected_genes
chr5	161116541	161116810	GABRA6	693_selected_genes
chr5	161117181	161117384	GABRA6	693_selected_genes
chr5	161118921	161119231	GABRA6	693_selected_genes
chr5	161128478	161128804	GABRA6	693_selected_genes
chrX	48649491	48649761	GATA1	693_selected_genes
chrX	48650225	48650653	GATA1	693_selected_genes
chrX	48650704	48650900	GATA1	693_selected_genes
chrX	48651553	48651729	GATA1	693_selected_genes
chrX	48652174	48652700	GATA1	693_selected_genes
chr3	128199836	128200186	GATA2	693_selected_genes
chr3	128200636	128200812	GATA2	693_selected_genes
chr3	128202677	128202873	GATA2	693_selected_genes
chr3	128204544	128205236	GATA2	693_selected_genes
chr3	128205620	128205899	GATA2	693_selected_genes
chr10	8097593	8097884	GATA3	693_selected_genes
chr10	8100242	8100829	GATA3	693_selected_genes
chr10	8105930	8106126	GATA3	693_selected_genes
chr10	8111410	8111586	GATA3	693_selected_genes
chr10	8115676	8116011	GATA3	693_selected_genes
chr17	17942753	17943241	GID4	693_selected_genes
chr17	17948435	17948545	GID4	693_selected_genes
chr17	17957415	17957573	GID4	693_selected_genes
chr17	17959991	17960365	GID4	693_selected_genes
chr17	17962156	17962308	GID4	693_selected_genes
chr17	17965133	17965318	GID4	693_selected_genes
chr17	17968468	17968582	GID4	693_selected_genes
chrX	100652771	100653112	GLA	693_selected_genes
chrX	100653332	100653580	GLA	693_selected_genes
chrX	100653747	100653959	GLA	693_selected_genes
chrX	100654733	100654813	GLA	693_selected_genes
chrX	100655628	100655770	GLA	693_selected_genes
chrX	100656594	100656822	GLA	693_selected_genes
chrX	100658773	100658998	GLA	693_selected_genes
chrX	100662672	100662916	GLA	693_selected_genes
chr19	3094624	3094810	GNA11	693_selected_genes
chr19	3110121	3110356	GNA11	693_selected_genes
chr19	3113302	3113507	GNA11	693_selected_genes
chr19	3114916	3115095	GNA11	693_selected_genes
chr19	3118896	3119076	GNA11	693_selected_genes
chr19	3119178	3119382	GNA11	693_selected_genes
chr19	3120961	3121202	GNA11	693_selected_genes
chr19	3121317	3121457	GNA11	693_selected_genes
chr17	63010349	63010972	GNA13	693_selected_genes
chr17	63014345	63014446	GNA13	693_selected_genes
chr17	63049594	63049871	GNA13	693_selected_genes
chr17	63052403	63052736	GNA13	693_selected_genes
chr7	79764451	79764619	GNAI1	693_selected_genes

chr7	79818241	79818334	GNAI1	693_selected_genes
chr7	79818380	79818572	GNAI1	693_selected_genes
chr7	79828515	79828723	GNAI1	693_selected_genes
chr7	79832994	79833173	GNAI1	693_selected_genes
chr7	79840259	79840439	GNAI1	693_selected_genes
chr7	79842006	79842210	GNAI1	693_selected_genes
chr7	79846590	79846834	GNAI1	693_selected_genes
chr9	80336213	80336454	GNAQ	693_selected_genes
chr9	80343404	80343608	GNAQ	693_selected_genes
chr9	80409353	80409533	GNAQ	693_selected_genes
chr9	80412410	80412589	GNAQ	693_selected_genes
chr9	80430506	80430711	GNAQ	693_selected_genes
chr9	80537051	80537286	GNAQ	693_selected_genes
chr9	80645405	80645504	GNAQ	693_selected_genes
chr9	80645990	80646176	GNAQ	693_selected_genes
chr20	57415136	57415924	GNAS	693_selected_genes
chr20	57428295	57430413	GNAS	693_selected_genes
chr20	57430583	57430728	GNAS	693_selected_genes
chr20	57466756	57466945	GNAS	693_selected_genes
chr20	57470641	57470764	GNAS	693_selected_genes
chr20	57473970	57474065	GNAS	693_selected_genes
chr20	57474978	57475035	GNAS	693_selected_genes
chr20	57478557	57478665	GNAS	693_selected_genes
chr20	57478701	57478871	GNAS	693_selected_genes
chr20	57480412	57480560	GNAS	693_selected_genes
chr20	57484191	57484296	GNAS	693_selected_genes
chr20	57484379	57484503	GNAS	693_selected_genes
chr20	57484550	57484659	GNAS	693_selected_genes
chr20	57484713	57484884	GNAS	693_selected_genes
chr20	57484980	57485161	GNAS	693_selected_genes
chr20	57485363	57485481	GNAS	693_selected_genes
chr20	57485712	57485909	GNAS	693_selected_genes
chr14	93263757	93264351	GOLGA5	693_selected_genes
chr14	93273055	93273333	GOLGA5	693_selected_genes
chr14	93275619	93275889	GOLGA5	693_selected_genes
chr14	93276573	93276747	GOLGA5	693_selected_genes
chr14	93277870	93278124	GOLGA5	693_selected_genes
chr14	93282570	93282791	GOLGA5	693_selected_genes
chr14	93286027	93286206	GOLGA5	693_selected_genes
chr14	93290865	93291014	GOLGA5	693_selected_genes
chr14	93299441	93299717	GOLGA5	693_selected_genes
chr14	93301878	93302034	GOLGA5	693_selected_genes
chr14	93303705	93303819	GOLGA5	693_selected_genes
chr14	93305685	93305816	GOLGA5	693_selected_genes
chr10	101157278	101157468	GOT1	693_selected_genes
chr10	101162311	101162504	GOT1	693_selected_genes
chr10	101163200	101163416	GOT1	693_selected_genes
chr10	101163455	101163656	GOT1	693_selected_genes
chr10	101165487	101165642	GOT1	693_selected_genes
chr10	101165868	101166031	GOT1	693_selected_genes
chr10	101166457	101166631	GOT1	693_selected_genes
chr10	101180355	101180587	GOT1	693_selected_genes
chr10	101189953	101190058	GOT1	693_selected_genes
chr10	101190179	101190347	GOT1	693_selected_genes
chr17	7214872	7214934	GPS2	693_selected_genes
chr17	7216049	7216183	GPS2	693_selected_genes
chr17	7216256	7216468	GPS2	693_selected_genes
chr17	7216505	7216635	GPS2	693_selected_genes
chr17	7216673	7216813	GPS2	693_selected_genes
chr17	7216861	7217065	GPS2	693_selected_genes
chr17	7217199	7217332	GPS2	693_selected_genes
chr17	7217373	7217503	GPS2	693_selected_genes
chr17	7217584	7217747	GPS2	693_selected_genes
chr17	7217781	7217941	GPS2	693_selected_genes
chr17	7218252	7218467	GPS2	693_selected_genes
chr3	49394795	49395205	GPX1	693_selected_genes
chr3	49395389	49395736	GPX1	693_selected_genes
chr15	33022866	33023471	GREM1	693_selected_genes
chr16	9856980	9858830	GRIN2A	693_selected_genes
chr16	9862682	9862971	GRIN2A	693_selected_genes
chr16	9892108	9892346	GRIN2A	693_selected_genes
chr16	9916095	9916306	GRIN2A	693_selected_genes
chr16	9923254	9923534	GRIN2A	693_selected_genes
chr16	9927936	9928112	GRIN2A	693_selected_genes
chr16	9934478	9934682	GRIN2A	693_selected_genes
chr16	9934767	9934986	GRIN2A	693_selected_genes
chr16	9943587	9943843	GRIN2A	693_selected_genes
chr16	9984817	9984982	GRIN2A	693_selected_genes
chr16	10031790	10032433	GRIN2A	693_selected_genes
chr16	10273829	10274293	GRIN2A	693_selected_genes
chr7	86274063	86274213	GRM3	693_selected_genes
chr7	86394436	86394954	GRM3	693_selected_genes
chr7	86415551	86416457	GRM3	693_selected_genes
chr7	86468129	86469246	GRM3	693_selected_genes

chr7	86479660	86479885	GRM3	693_selected_genes
chr7	86493572	86493737	GRM3	693_selected_genes
chr3	119545609	119545727	GSK3B	693_selected_genes
chr3	119562076	119562225	GSK3B	693_selected_genes
chr3	119582240	119582477	GSK3B	693_selected_genes
chr3	119585411	119585500	GSK3B	693_selected_genes
chr3	119595234	119595380	GSK3B	693_selected_genes
chr3	119624576	119624724	GSK3B	693_selected_genes
chr3	119631525	119631682	GSK3B	693_selected_genes
chr3	119634865	119635046	GSK3B	693_selected_genes
chr3	119642194	119642355	GSK3B	693_selected_genes
chr3	119666089	119666223	GSK3B	693_selected_genes
chr3	119720867	119721111	GSK3B	693_selected_genes
chr3	119812168	119812306	GSK3B	693_selected_genes
chr11	67351289	67351340	GSTP1	693_selected_genes
chr11	67351579	67351665	GSTP1	693_selected_genes
chr11	67351909	67352066	GSTP1	693_selected_genes
chr11	67352130	67352268	GSTP1	693_selected_genes
chr11	67352583	67352737	GSTP1	693_selected_genes
chr11	67353549	67353707	GSTP1	693_selected_genes
chr11	67353834	67354073	GSTP1	693_selected_genes
chr11	106558249	106558507	GUCY1A2	693_selected_genes
chr11	106579212	106579417	GUCY1A2	693_selected_genes
chr11	106617251	106617394	GUCY1A2	693_selected_genes
chr11	106647139	106647333	GUCY1A2	693_selected_genes
chr11	106680693	106681229	GUCY1A2	693_selected_genes
chr11	106807350	106807463	GUCY1A2	693_selected_genes
chr11	106810160	106810929	GUCY1A2	693_selected_genes
chr11	106849319	106849491	GUCY1A2	693_selected_genes
chr11	106856770	106856882	GUCY1A2	693_selected_genes
chr11	106888453	106888806	GUCY1A2	693_selected_genes
chr7	65425858	65426075	GUSB	693_selected_genes
chr7	65429284	65429470	GUSB	693_selected_genes
chr7	65432692	65432919	GUSB	693_selected_genes
chr7	65435243	65435378	GUSB	693_selected_genes
chr7	65439256	65439453	GUSB	693_selected_genes
chr7	65439487	65439716	GUSB	693_selected_genes
chr7	65439880	65440083	GUSB	693_selected_genes
chr7	65440976	65441214	GUSB	693_selected_genes
chr7	65444360	65444553	GUSB	693_selected_genes
chr7	65444688	65444923	GUSB	693_selected_genes
chr7	65445185	65445421	GUSB	693_selected_genes
chr7	65446935	65447195	GUSB	693_selected_genes
chr1	226252027	226252205	H3F3A	693_selected_genes
chr1	226253331	226253625	H3F3A	693_selected_genes
chr1	226259026	226259205	H3F3A	693_selected_genes
chr12	31944667	31945125	H3F3C	693_selected_genes
chrX	153214772	153214862	HCFC1	693_selected_genes
chrX	153214978	153215092	HCFC1	693_selected_genes
chrX	153215668	153216019	HCFC1	693_selected_genes
chrX	153216238	153216474	HCFC1	693_selected_genes
chrX	153216775	153216963	HCFC1	693_selected_genes
chrX	153217014	153217183	HCFC1	693_selected_genes
chrX	153217266	153217637	HCFC1	693_selected_genes
chrX	153217939	153218566	HCFC1	693_selected_genes
chrX	153219032	153219246	HCFC1	693_selected_genes
chrX	153219491	153221018	HCFC1	693_selected_genes
chrX	153221616	153221887	HCFC1	693_selected_genes
chrX	153222050	153222239	HCFC1	693_selected_genes
chrX	153222343	153222536	HCFC1	693_selected_genes
chrX	153222739	153223009	HCFC1	693_selected_genes
chrX	153223207	153223362	HCFC1	693_selected_genes
chrX	153223450	153223725	HCFC1	693_selected_genes
chrX	153223994	153224242	HCFC1	693_selected_genes
chrX	153224756	153224967	HCFC1	693_selected_genes
chrX	153225227	153225637	HCFC1	693_selected_genes
chrX	153225660	153225890	HCFC1	693_selected_genes
chrX	153226967	153227124	HCFC1	693_selected_genes
chrX	153227647	153227782	HCFC1	693_selected_genes
chrX	153228650	153228909	HCFC1	693_selected_genes
chrX	153229549	153229760	HCFC1	693_selected_genes
chrX	153230003	153230202	HCFC1	693_selected_genes
chrX	153236073	153236316	HCFC1	693_selected_genes
chr15	63901254	63901490	HERC1	693_selected_genes
chr15	63904424	63904780	HERC1	693_selected_genes
chr15	63907941	63908144	HERC1	693_selected_genes
chr15	63908603	63908906	HERC1	693_selected_genes
chr15	63914988	63915115	HERC1	693_selected_genes
chr15	63915898	63916114	HERC1	693_selected_genes
chr15	63916331	63916551	HERC1	693_selected_genes
chr15	63918115	63918378	HERC1	693_selected_genes
chr15	63920850	63921039	HERC1	693_selected_genes
chr15	63922639	63922859	HERC1	693_selected_genes
chr15	63924768	63924829	HERC1	693_selected_genes

chr15	63924882	63925035	HERC1	693_selected_genes
chr15	63926021	63926194	HERC1	693_selected_genes
chr15	63926906	63927112	HERC1	693_selected_genes
chr15	63928134	63928366	HERC1	693_selected_genes
chr15	63929273	63929358	HERC1	693_selected_genes
chr15	63929678	63929867	HERC1	693_selected_genes
chr15	63930584	63930760	HERC1	693_selected_genes
chr15	63930884	63931000	HERC1	693_selected_genes
chr15	63932325	63932669	HERC1	693_selected_genes
chr15	63933643	63933867	HERC1	693_selected_genes
chr15	63935130	63935282	HERC1	693_selected_genes
chr15	63935577	63935774	HERC1	693_selected_genes
chr15	63937165	63937321	HERC1	693_selected_genes
chr15	63937656	63937906	HERC1	693_selected_genes
chr15	63940242	63940423	HERC1	693_selected_genes
chr15	63941898	63942149	HERC1	693_selected_genes
chr15	63943426	63943604	HERC1	693_selected_genes
chr15	63944587	63944765	HERC1	693_selected_genes
chr15	63946292	63946548	HERC1	693_selected_genes
chr15	63947915	63948179	HERC1	693_selected_genes
chr15	63948261	63948582	HERC1	693_selected_genes
chr15	63950717	63950942	HERC1	693_selected_genes
chr15	63951909	63952160	HERC1	693_selected_genes
chr15	63953146	63953249	HERC1	693_selected_genes
chr15	63953926	63954245	HERC1	693_selected_genes
chr15	63955157	63955428	HERC1	693_selected_genes
chr15	63956643	63956818	HERC1	693_selected_genes
chr15	63958092	63958374	HERC1	693_selected_genes
chr15	63958529	63958696	HERC1	693_selected_genes
chr15	63961711	63961922	HERC1	693_selected_genes
chr15	63964669	63964918	HERC1	693_selected_genes
chr15	63966515	63967341	HERC1	693_selected_genes
chr15	63970018	63970589	HERC1	693_selected_genes
chr15	63972250	63972384	HERC1	693_selected_genes
chr15	63972710	63973000	HERC1	693_selected_genes
chr15	63978532	63978759	HERC1	693_selected_genes
chr15	63981762	63981923	HERC1	693_selected_genes
chr15	63982714	63982871	HERC1	693_selected_genes
chr15	63984584	63984790	HERC1	693_selected_genes
chr15	63986137	63986381	HERC1	693_selected_genes
chr15	63986485	63986773	HERC1	693_selected_genes
chr15	63986947	63987118	HERC1	693_selected_genes
chr15	63988297	63988563	HERC1	693_selected_genes
chr15	63990901	63991220	HERC1	693_selected_genes
chr15	63998953	63999055	HERC1	693_selected_genes
chr15	64004948	64005119	HERC1	693_selected_genes
chr15	64005526	64005889	HERC1	693_selected_genes
chr15	64008475	64008697	HERC1	693_selected_genes
chr15	64010747	64010918	HERC1	693_selected_genes
chr15	64010956	64011121	HERC1	693_selected_genes
chr15	64015355	64015579	HERC1	693_selected_genes
chr15	64017465	64017737	HERC1	693_selected_genes
chr15	64019820	64020062	HERC1	693_selected_genes
chr15	64021409	64021592	HERC1	693_selected_genes
chr15	64021670	64021873	HERC1	693_selected_genes
chr15	64025097	64025369	HERC1	693_selected_genes
chr15	64026897	64027073	HERC1	693_selected_genes
chr15	64039091	64039307	HERC1	693_selected_genes
chr15	64039897	64040082	HERC1	693_selected_genes
chr15	64041540	64041762	HERC1	693_selected_genes
chr15	64041820	64042015	HERC1	693_selected_genes
chr15	64045131	64045309	HERC1	693_selected_genes
chr15	64046678	64046872	HERC1	693_selected_genes
chr15	64047402	64047549	HERC1	693_selected_genes
chr15	64048610	64048972	HERC1	693_selected_genes
chr15	64050348	64050593	HERC1	693_selected_genes
chr15	64056269	64056415	HERC1	693_selected_genes
chr15	64066867	64067847	HERC1	693_selected_genes
chr7	81331871	81332098	HGF	693_selected_genes
chr7	81334680	81334876	HGF	693_selected_genes
chr7	81334937	81335094	HGF	693_selected_genes
chr7	81335577	81335768	HGF	693_selected_genes
chr7	81336580	81336705	HGF	693_selected_genes
chr7	81339437	81339584	HGF	693_selected_genes
chr7	81340771	81340860	HGF	693_selected_genes
chr7	81346522	81346706	HGF	693_selected_genes
chr7	81350035	81350188	HGF	693_selected_genes
chr7	81355180	81355358	HGF	693_selected_genes
chr7	81358895	81359120	HGF	693_selected_genes
chr7	81372232	81372290	HGF	693_selected_genes
chr7	81372643	81372812	HGF	693_selected_genes
chr7	81374290	81374461	HGF	693_selected_genes
chr7	81381402	81381603	HGF	693_selected_genes
chr7	81385281	81385404	HGF	693_selected_genes

chr7	81386475	81386644	HGF	693_selected_genes
chr7	81387982	81388145	HGF	693_selected_genes
chr7	81391997	81392213	HGF	693_selected_genes
chr7	81399174	81399312	HGF	693_selected_genes
chr14	62162497	62162582	HIF1A	693_selected_genes
chr14	62164543	62164697	HIF1A	693_selected_genes
chr14	62187071	62187315	HIF1A	693_selected_genes
chr14	62188201	62188397	HIF1A	693_selected_genes
chr14	62188431	62188566	HIF1A	693_selected_genes
chr14	62193398	62193561	HIF1A	693_selected_genes
chr14	62194145	62194398	HIF1A	693_selected_genes
chr14	62199110	62199267	HIF1A	693_selected_genes
chr14	62200830	62201028	HIF1A	693_selected_genes
chr14	62203581	62203852	HIF1A	693_selected_genes
chr14	62204779	62205116	HIF1A	693_selected_genes
chr14	62207197	62207370	HIF1A	693_selected_genes
chr14	62207447	62207931	HIF1A	693_selected_genes
chr14	62211397	62211556	HIF1A	693_selected_genes
chr14	62212383	62212560	HIF1A	693_selected_genes
chr14	62213626	62213828	HIF1A	693_selected_genes
chr6	26055989	26056681	HIST1H1C	693_selected_genes
chr6	26158372	26158803	HIST1H2BD	693_selected_genes
chr6	26031852	26032313	HIST1H3B	693_selected_genes
chr6	29910305	29910428	HLA-A	693_selected_genes
chr6	29910508	29910828	HLA-A	693_selected_genes
chr6	29911019	29911345	HLA-A	693_selected_genes
chr6	29911873	29912199	HLA-A	693_selected_genes
chr6	29912251	29912436	HLA-A	693_selected_genes
chr6	29912810	29912893	HLA-A	693_selected_genes
chr6	29912985	29913083	HLA-A	693_selected_genes
chr6	29913202	29913257	HLA-A	693_selected_genes
chr6	31322234	31322328	HLA-B	693_selected_genes
chr6	31322384	31322467	HLA-B	693_selected_genes
chr6	31322858	31323025	HLA-B	693_selected_genes
chr6	31323068	31323394	HLA-B	693_selected_genes
chr6	31323502	31323608	HLA-B	693_selected_genes
chr6	31323918	31324244	HLA-B	693_selected_genes
chr6	31324439	31324962	HLA-B	693_selected_genes
chr12	121416403	121416922	HNF1A	693_selected_genes
chr12	121426610	121426860	HNF1A	693_selected_genes
chr12	121431297	121431534	HNF1A	693_selected_genes
chr12	121431941	121432233	HNF1A	693_selected_genes
chr12	121434039	121434241	HNF1A	693_selected_genes
chr12	121434318	121434824	HNF1A	693_selected_genes
chr12	121435251	121435621	HNF1A	693_selected_genes
chr12	121437045	121437455	HNF1A	693_selected_genes
chr12	121438842	121439020	HNF1A	693_selected_genes
chr11	532610	532780	HRAS	693_selected_genes
chr11	533251	533383	HRAS	693_selected_genes
chr11	533427	533637	HRAS	693_selected_genes
chr11	533740	533969	HRAS	693_selected_genes
chr11	534186	534347	HRAS	693_selected_genes
chr1	120050068	120050269	HSD3B1	693_selected_genes
chr1	120054100	120054315	HSD3B1	693_selected_genes
chr1	120056431	120057293	HSD3B1	693_selected_genes
chr6	44216341	44216538	HSP90AB1	693_selected_genes
chr6	44217088	44217345	HSP90AB1	693_selected_genes
chr6	44217386	44217596	HSP90AB1	693_selected_genes
chr6	44217732	44217916	HSP90AB1	693_selected_genes
chr6	44218002	44218361	HSP90AB1	693_selected_genes
chr6	44218759	44218975	HSP90AB1	693_selected_genes
chr6	44219129	44219370	HSP90AB1	693_selected_genes
chr6	44219448	44219646	HSP90AB1	693_selected_genes
chr6	44219710	44220029	HSP90AB1	693_selected_genes
chr6	44220756	44221140	HSP90AB1	693_selected_genes
chr6	44221200	44221360	HSP90AB1	693_selected_genes
chr6	44221381	44221624	HSP90AB1	693_selected_genes
chr11	122928416	122928652	HSPA8	693_selected_genes
chr11	122928934	122929217	HSPA8	693_selected_genes
chr11	122929314	122929563	HSPA8	693_selected_genes
chr11	122929741	122929994	HSPA8	693_selected_genes
chr11	122930155	122930761	HSPA8	693_selected_genes
chr11	122930798	122931001	HSPA8	693_selected_genes
chr11	122931275	122931531	HSPA8	693_selected_genes
chr11	122931802	122932057	HSPA8	693_selected_genes
chr21	45642904	45642986	ICOSLG	693_selected_genes
chr21	45648891	45648952	ICOSLG	693_selected_genes
chr21	45649387	45649997	ICOSLG	693_selected_genes
chr21	45651137	45651352	ICOSLG	693_selected_genes
chr21	45655129	45655470	ICOSLG	693_selected_genes
chr21	45656724	45657125	ICOSLG	693_selected_genes
chr21	45658305	45658396	ICOSLG	693_selected_genes
chr21	45660661	45660725	ICOSLG	693_selected_genes
chr2	209101777	209101918	IDH1	693_selected_genes

chr2	209103769	209103982	IDH1	693_selected_genes
chr2	209104561	209104752	IDH1	693_selected_genes
chr2	209106692	209106894	IDH1	693_selected_genes
chr2	209108125	209108353	IDH1	693_selected_genes
chr2	209110017	209110173	IDH1	693_selected_genes
chr2	209113064	209113409	IDH1	693_selected_genes
chr2	209116128	209116300	IDH1	693_selected_genes
chr15	90627472	90627610	IDH2	693_selected_genes
chr15	90628022	90628165	IDH2	693_selected_genes
chr15	90628207	90628355	IDH2	693_selected_genes
chr15	90628481	90628644	IDH2	693_selected_genes
chr15	90630318	90630520	IDH2	693_selected_genes
chr15	90630645	90630832	IDH2	693_selected_genes
chr15	90631565	90631759	IDH2	693_selected_genes
chr15	90631793	90632004	IDH2	693_selected_genes
chr15	90633685	90633901	IDH2	693_selected_genes
chr15	90634759	90634901	IDH2	693_selected_genes
chr15	90645482	90645647	IDH2	693_selected_genes
chr6	137519142	137519801	IFNGR1	693_selected_genes
chr6	137521992	137522170	IFNGR1	693_selected_genes
chr6	137524610	137524892	IFNGR1	693_selected_genes
chr6	137525443	137525666	IFNGR1	693_selected_genes
chr6	137527247	137527470	IFNGR1	693_selected_genes
chr6	137528074	137528239	IFNGR1	693_selected_genes
chr6	137536378	137536483	IFNGR1	693_selected_genes
chr6	137539562	137539613	IFNGR1	693_selected_genes
chr6	137540354	137540489	IFNGR1	693_selected_genes
chr12	102796259	102796369	IGF1	693_selected_genes
chr12	102811570	102811806	IGF1	693_selected_genes
chr12	102813261	102813493	IGF1	693_selected_genes
chr12	102869395	102869602	IGF1	693_selected_genes
chr12	102872239	102872304	IGF1	693_selected_genes
chr12	102874071	102874184	IGF1	693_selected_genes
chr15	99192785	99192929	IGF1R	693_selected_genes
chr15	99250765	99251361	IGF1R	693_selected_genes
chr15	99434528	99434891	IGF1R	693_selected_genes
chr15	99439960	99440159	IGF1R	693_selected_genes
chr15	99442680	99442875	IGF1R	693_selected_genes
chr15	99451888	99452153	IGF1R	693_selected_genes
chr15	99454518	99454695	IGF1R	693_selected_genes
chr15	99456247	99456536	IGF1R	693_selected_genes
chr15	99459167	99459385	IGF1R	693_selected_genes
chr15	99459875	99460130	IGF1R	693_selected_genes
chr15	99465351	99465685	IGF1R	693_selected_genes
chr15	99467079	99467266	IGF1R	693_selected_genes
chr15	99467728	99467938	IGF1R	693_selected_genes
chr15	99472761	99472914	IGF1R	693_selected_genes
chr15	99473438	99473559	IGF1R	693_selected_genes
chr15	99478027	99478307	IGF1R	693_selected_genes
chr15	99478519	99478680	IGF1R	693_selected_genes
chr15	99482404	99482614	IGF1R	693_selected_genes
chr15	99486126	99486306	IGF1R	693_selected_genes
chr15	99491777	99491962	IGF1R	693_selected_genes
chr15	99500264	99500696	IGF1R	693_selected_genes
chr11	2154191	2154478	IGF2	693_selected_genes
chr11	2154721	2154929	IGF2	693_selected_genes
chr11	2156571	2156784	IGF2	693_selected_genes
chr11	2161339	2161551	IGF2	693_selected_genes
chr6	160390253	160390452	IGF2R	693_selected_genes
chr6	160412190	160412380	IGF2R	693_selected_genes
chr6	160430016	160430191	IGF2R	693_selected_genes
chr6	160431693	160431842	IGF2R	693_selected_genes
chr6	160445578	160445761	IGF2R	693_selected_genes
chr6	160448191	160448371	IGF2R	693_selected_genes
chr6	160450556	160450712	IGF2R	693_selected_genes
chr6	160453557	160453770	IGF2R	693_selected_genes
chr6	160453948	160454164	IGF2R	693_selected_genes
chr6	160455425	160455579	IGF2R	693_selected_genes
chr6	160461566	160461781	IGF2R	693_selected_genes
chr6	160464154	160464345	IGF2R	693_selected_genes
chr6	160465520	160465714	IGF2R	693_selected_genes
chr6	160466751	160466939	IGF2R	693_selected_genes
chr6	160467504	160467702	IGF2R	693_selected_genes
chr6	160468165	160468393	IGF2R	693_selected_genes
chr6	160468798	160468964	IGF2R	693_selected_genes
chr6	160469381	160469600	IGF2R	693_selected_genes
chr6	160471479	160471709	IGF2R	693_selected_genes
chr6	160477430	160477582	IGF2R	693_selected_genes
chr6	160479029	160479181	IGF2R	693_selected_genes
chr6	160479912	160480155	IGF2R	693_selected_genes
chr6	160481553	160481774	IGF2R	693_selected_genes
chr6	160482509	160482703	IGF2R	693_selected_genes
chr6	160482759	160482985	IGF2R	693_selected_genes
chr6	160483538	160483676	IGF2R	693_selected_genes

chr6	160484421	160484687	IGF2R	693_selected_genes
chr6	160485407	160485588	IGF2R	693_selected_genes
chr6	160485810	160485958	IGF2R	693_selected_genes
chr6	160489255	160489442	IGF2R	693_selected_genes
chr6	160490874	160491115	IGF2R	693_selected_genes
chr6	160492916	160493093	IGF2R	693_selected_genes
chr6	160493771	160493941	IGF2R	693_selected_genes
chr6	160494219	160494526	IGF2R	693_selected_genes
chr6	160494763	160495032	IGF2R	693_selected_genes
chr6	160496853	160497053	IGF2R	693_selected_genes
chr6	160499207	160499419	IGF2R	693_selected_genes
chr6	160500586	160500844	IGF2R	693_selected_genes
chr6	160501135	160501332	IGF2R	693_selected_genes
chr6	160504956	160505241	IGF2R	693_selected_genes
chr6	160506001	160506188	IGF2R	693_selected_genes
chr6	160509039	160509204	IGF2R	693_selected_genes
chr6	160510113	160510310	IGF2R	693_selected_genes
chr6	160510922	160511195	IGF2R	693_selected_genes
chr6	160517445	160517682	IGF2R	693_selected_genes
chr6	160523525	160523728	IGF2R	693_selected_genes
chr6	160524752	160524872	IGF2R	693_selected_genes
chr6	160525680	160526141	IGF2R	693_selected_genes
chr1	206646545	206646682	IKBKE	693_selected_genes
chr1	206647648	206647839	IKBKE	693_selected_genes
chr1	206648182	206648362	IKBKE	693_selected_genes
chr1	206649498	206649730	IKBKE	693_selected_genes
chr1	206649995	206650206	IKBKE	693_selected_genes
chr1	206651066	206651227	IKBKE	693_selected_genes
chr1	206651477	206651707	IKBKE	693_selected_genes
chr1	206652260	206652501	IKBKE	693_selected_genes
chr1	206653187	206653302	IKBKE	693_selected_genes
chr1	206653339	206653481	IKBKE	693_selected_genes
chr1	206653764	206653901	IKBKE	693_selected_genes
chr1	206658308	206658434	IKBKE	693_selected_genes
chr1	206658505	206658668	IKBKE	693_selected_genes
chr1	206661225	206661352	IKBKE	693_selected_genes
chr1	206664126	206664216	IKBKE	693_selected_genes
chr1	206664955	206665107	IKBKE	693_selected_genes
chr1	206666330	206666477	IKBKE	693_selected_genes
chr1	206666573	206666736	IKBKE	693_selected_genes
chr1	206667227	206667349	IKBKE	693_selected_genes
chr1	206669419	206669503	IKBKE	693_selected_genes
chr7	50358632	50358722	IKZF1	693_selected_genes
chr7	50367208	50367378	IKZF1	693_selected_genes
chr7	50435678	50436093	IKZF1	693_selected_genes
chr7	50437194	50437258	IKZF1	693_selected_genes
chr7	50444205	50444516	IKZF1	693_selected_genes
chr7	50450212	50450430	IKZF1	693_selected_genes
chr7	50455017	50455193	IKZF1	693_selected_genes
chr7	50459401	50459586	IKZF1	693_selected_genes
chr7	50467590	50468350	IKZF1	693_selected_genes
chr1	206941955	206942098	IL10	693_selected_genes
chr1	206943148	206943264	IL10	693_selected_genes
chr1	206944226	206944429	IL10	693_selected_genes
chr1	206944675	206944785	IL10	693_selected_genes
chr1	206945590	206945805	IL10	693_selected_genes
chr5	35857054	35857186	IL7R	693_selected_genes
chr5	35860928	35861117	IL7R	693_selected_genes
chr5	35867382	35867664	IL7R	693_selected_genes
chr5	35871132	35871340	IL7R	693_selected_genes
chr5	35873556	35873775	IL7R	693_selected_genes
chr5	35874525	35874669	IL7R	693_selected_genes
chr5	35875490	35875718	IL7R	693_selected_genes
chr5	35876059	35876613	IL7R	693_selected_genes
chr13	111365295	111365349	ING1	693_selected_genes
chr13	111366471	111366657	ING1	693_selected_genes
chr13	111367765	111368380	ING1	693_selected_genes
chr13	111371550	111372304	ING1	693_selected_genes
chr7	41729222	41730165	INHBA	693_selected_genes
chr7	41739559	41739997	INHBA	693_selected_genes
chr4	142949909	142950092	INPP4B	693_selected_genes
chr4	142951255	142951480	INPP4B	693_selected_genes
chr4	143003158	143003363	INPP4B	693_selected_genes
chr4	143007271	143007434	INPP4B	693_selected_genes
chr4	143029220	143029368	INPP4B	693_selected_genes
chr4	143033669	143033860	INPP4B	693_selected_genes
chr4	143043255	143043423	INPP4B	693_selected_genes
chr4	143044419	143044593	INPP4B	693_selected_genes
chr4	143045715	143045938	INPP4B	693_selected_genes
chr4	143066967	143067174	INPP4B	693_selected_genes
chr4	143081485	143081739	INPP4B	693_selected_genes
chr4	143094759	143094987	INPP4B	693_selected_genes
chr4	143114214	143114373	INPP4B	693_selected_genes
chr4	143129552	143129707	INPP4B	693_selected_genes

chr4	143130023	143130204	INPP4B	693_selected_genes
chr4	143158991	143159189	INPP4B	693_selected_genes
chr4	143181619	143181742	INPP4B	693_selected_genes
chr4	143191790	143191952	INPP4B	693_selected_genes
chr4	143226585	143226715	INPP4B	693_selected_genes
chr4	143226827	143226913	INPP4B	693_selected_genes
chr4	143235839	143235940	INPP4B	693_selected_genes
chr4	143268654	143268734	INPP4B	693_selected_genes
chr4	143305220	143305294	INPP4B	693_selected_genes
chr4	143324065	143324232	INPP4B	693_selected_genes
chr4	143326333	143326502	INPP4B	693_selected_genes
chr4	143344937	143344995	INPP4B	693_selected_genes
chr4	143348579	143348655	INPP4B	693_selected_genes
chr4	143350300	143350395	INPP4B	693_selected_genes
chr4	143352296	143352437	INPP4B	693_selected_genes
chr11	71936003	71936235	INPPL1	693_selected_genes
chr11	71939208	71939322	INPPL1	693_selected_genes
chr11	71939366	71939567	INPPL1	693_selected_genes
chr11	71939745	71939916	INPPL1	693_selected_genes
chr11	71940108	71940299	INPPL1	693_selected_genes
chr11	71940483	71940627	INPPL1	693_selected_genes
chr11	71940681	71940821	INPPL1	693_selected_genes
chr11	71940942	71941088	INPPL1	693_selected_genes
chr11	71941139	71941340	INPPL1	693_selected_genes
chr11	71941380	71941537	INPPL1	693_selected_genes
chr11	71941814	71941967	INPPL1	693_selected_genes
chr11	71942011	71942258	INPPL1	693_selected_genes
chr11	71942516	71942684	INPPL1	693_selected_genes
chr11	71943258	71943405	INPPL1	693_selected_genes
chr11	71943644	71943833	INPPL1	693_selected_genes
chr11	71943893	71944043	INPPL1	693_selected_genes
chr11	71944093	71944232	INPPL1	693_selected_genes
chr11	71944459	71944591	INPPL1	693_selected_genes
chr11	71944673	71944813	INPPL1	693_selected_genes
chr11	71945299	71945463	INPPL1	693_selected_genes
chr11	71945545	71945684	INPPL1	693_selected_genes
chr11	71946134	71946272	INPPL1	693_selected_genes
chr11	71946314	71946520	INPPL1	693_selected_genes
chr11	71946693	71946822	INPPL1	693_selected_genes
chr11	71946864	71947055	INPPL1	693_selected_genes
chr11	71948142	71948865	INPPL1	693_selected_genes
chr11	71949060	71949422	INPPL1	693_selected_genes
chr19	7117041	7117446	INSR	693_selected_genes
chr19	7119434	7119619	INSR	693_selected_genes
chr19	7120605	7120785	INSR	693_selected_genes
chr19	7122599	7122809	INSR	693_selected_genes
chr19	7122864	7123025	INSR	693_selected_genes
chr19	7125268	7125563	INSR	693_selected_genes
chr19	7126569	7126687	INSR	693_selected_genes
chr19	7128837	7128990	INSR	693_selected_genes
chr19	7132143	7132353	INSR	693_selected_genes
chr19	7141662	7141852	INSR	693_selected_genes
chr19	7142801	7143126	INSR	693_selected_genes
chr19	7150482	7150568	INSR	693_selected_genes
chr19	7152711	7152963	INSR	693_selected_genes
chr19	7159602	7159681	INSR	693_selected_genes
chr19	7163017	7163235	INSR	693_selected_genes
chr19	7166139	7166440	INSR	693_selected_genes
chr19	7167953	7168130	INSR	693_selected_genes
chr19	7170522	7170787	INSR	693_selected_genes
chr19	7172275	7172470	INSR	693_selected_genes
chr19	7174568	7174767	INSR	693_selected_genes
chr19	7184301	7184673	INSR	693_selected_genes
chr19	7267330	7267932	INSR	693_selected_genes
chr19	7293777	7293927	INSR	693_selected_genes
chr6	393127	393393	IRF4	693_selected_genes
chr6	394795	395032	IRF4	693_selected_genes
chr6	395821	395960	IRF4	693_selected_genes
chr6	397082	397277	IRF4	693_selected_genes
chr6	398802	398960	IRF4	693_selected_genes
chr6	401398	401802	IRF4	693_selected_genes
chr6	404992	405155	IRF4	693_selected_genes
chr6	406740	406811	IRF4	693_selected_genes
chr6	407429	407623	IRF4	693_selected_genes
chr2	227659700	227663479	IRS1	693_selected_genes
chr13	110408625	110408680	IRS2	693_selected_genes
chr13	110434363	110438425	IRS2	693_selected_genes
chr20	10620120	10620628	JAG1	693_selected_genes
chr20	10621405	10621606	JAG1	693_selected_genes
chr20	10621735	10621917	JAG1	693_selected_genes
chr20	10622082	10622366	JAG1	693_selected_genes
chr20	10622405	10622565	JAG1	693_selected_genes
chr20	10623110	10623274	JAG1	693_selected_genes
chr20	10624400	10624536	JAG1	693_selected_genes

chr20	10624979	10625057	JAG1	693_selected_genes
chr20	10625485	10625652	JAG1	693_selected_genes
chr20	10625765	10625929	JAG1	693_selected_genes
chr20	10625978	10626142	JAG1	693_selected_genes
chr20	10626593	10626757	JAG1	693_selected_genes
chr20	10627561	10627776	JAG1	693_selected_genes
chr20	10628582	10628783	JAG1	693_selected_genes
chr20	10629171	10629395	JAG1	693_selected_genes
chr20	10629683	10629780	JAG1	693_selected_genes
chr20	10630144	10630308	JAG1	693_selected_genes
chr20	10630869	10631033	JAG1	693_selected_genes
chr20	10632203	10632367	JAG1	693_selected_genes
chr20	10632753	10632923	JAG1	693_selected_genes
chr20	10633090	10633271	JAG1	693_selected_genes
chr20	10637020	10637131	JAG1	693_selected_genes
chr20	10639090	10639395	JAG1	693_selected_genes
chr20	10644585	10644687	JAG1	693_selected_genes
chr20	10653323	10653679	JAG1	693_selected_genes
chr20	10654072	10654203	JAG1	693_selected_genes
chr14	105609006	105609532	JAG2	693_selected_genes
chr14	105609793	105610000	JAG2	693_selected_genes
chr14	105611241	105611423	JAG2	693_selected_genes
chr14	105612042	105612335	JAG2	693_selected_genes
chr14	105612696	105612862	JAG2	693_selected_genes
chr14	105612922	105613086	JAG2	693_selected_genes
chr14	105613637	105613773	JAG2	693_selected_genes
chr14	105613811	105613889	JAG2	693_selected_genes
chr14	105614083	105614250	JAG2	693_selected_genes
chr14	105614427	105614591	JAG2	693_selected_genes
chr14	105614637	105614801	JAG2	693_selected_genes
chr14	105615056	105615220	JAG2	693_selected_genes
chr14	105615248	105615451	JAG2	693_selected_genes
chr14	105615481	105615682	JAG2	693_selected_genes
chr14	105616915	105617139	JAG2	693_selected_genes
chr14	105617176	105617273	JAG2	693_selected_genes
chr14	105617302	105617466	JAG2	693_selected_genes
chr14	105617594	105617758	JAG2	693_selected_genes
chr14	105617937	105618101	JAG2	693_selected_genes
chr14	105618249	105618419	JAG2	693_selected_genes
chr14	105618472	105618653	JAG2	693_selected_genes
chr14	105621873	105621984	JAG2	693_selected_genes
chr14	105622049	105622351	JAG2	693_selected_genes
chr14	105624017	105624125	JAG2	693_selected_genes
chr14	105634068	105634469	JAG2	693_selected_genes
chr14	105634666	105634782	JAG2	693_selected_genes
chr1	65300219	65300365	JAK1	693_selected_genes
chr1	65301053	65301214	JAK1	693_selected_genes
chr1	65301755	65301923	JAK1	693_selected_genes
chr1	65303589	65303812	JAK1	693_selected_genes
chr1	65304122	65304297	JAK1	693_selected_genes
chr1	65305260	65305503	JAK1	693_selected_genes
chr1	65306902	65307047	JAK1	693_selected_genes
chr1	65307108	65307309	JAK1	693_selected_genes
chr1	65309721	65309923	JAK1	693_selected_genes
chr1	65310411	65310597	JAK1	693_selected_genes
chr1	65311170	65311348	JAK1	693_selected_genes
chr1	65312306	65312444	JAK1	693_selected_genes
chr1	65313189	65313383	JAK1	693_selected_genes
chr1	65316461	65316618	JAK1	693_selected_genes
chr1	65321166	65321406	JAK1	693_selected_genes
chr1	65323313	65323487	JAK1	693_selected_genes
chr1	65325762	65325970	JAK1	693_selected_genes
chr1	65330444	65330680	JAK1	693_selected_genes
chr1	65332523	65332916	JAK1	693_selected_genes
chr1	65334968	65335182	JAK1	693_selected_genes
chr1	65339027	65339231	JAK1	693_selected_genes
chr1	65344682	65344856	JAK1	693_selected_genes
chr1	65348934	65349183	JAK1	693_selected_genes
chr1	65351916	65351972	JAK1	693_selected_genes
chr9	5021962	5022238	JAK2	693_selected_genes
chr9	5029757	5029931	JAK2	693_selected_genes
chr9	5044377	5044545	JAK2	693_selected_genes
chr9	5050660	5050856	JAK2	693_selected_genes
chr9	5054537	5054909	JAK2	693_selected_genes
chr9	5055643	5055813	JAK2	693_selected_genes
chr9	5064857	5065065	JAK2	693_selected_genes
chr9	5066652	5066814	JAK2	693_selected_genes
chr9	5068996	5069233	JAK2	693_selected_genes
chr9	5069899	5070077	JAK2	693_selected_genes
chr9	5072466	5072651	JAK2	693_selected_genes
chr9	5073672	5073810	JAK2	693_selected_genes
chr9	5077427	5077605	JAK2	693_selected_genes
chr9	5078280	5078469	JAK2	693_selected_genes
chr9	5080203	5080405	JAK2	693_selected_genes

chr9	5080507	5080708	JAK2	693_selected_genes
chr9	5081699	5081886	JAK2	693_selected_genes
chr9	5089648	5089888	JAK2	693_selected_genes
chr9	5090420	5090595	JAK2	693_selected_genes
chr9	5090713	5090936	JAK2	693_selected_genes
chr9	5122978	5123146	JAK2	693_selected_genes
chr9	5126307	5126471	JAK2	693_selected_genes
chr9	5126658	5126816	JAK2	693_selected_genes
chr19	17937526	17937744	JAK3	693_selected_genes
chr19	17940813	17941052	JAK3	693_selected_genes
chr19	17941286	17941454	JAK3	693_selected_genes
chr19	17942011	17942234	JAK3	693_selected_genes
chr19	17942457	17942632	JAK3	693_selected_genes
chr19	17943302	17943542	JAK3	693_selected_genes
chr19	17943573	17943763	JAK3	693_selected_genes
chr19	17945354	17945555	JAK3	693_selected_genes
chr19	17945635	17945837	JAK3	693_selected_genes
chr19	17945866	17946049	JAK3	693_selected_genes
chr19	17946707	17946885	JAK3	693_selected_genes
chr19	17947912	17948047	JAK3	693_selected_genes
chr19	17948715	17948897	JAK3	693_selected_genes
chr19	17949046	17949224	JAK3	693_selected_genes
chr19	17950260	17950497	JAK3	693_selected_genes
chr19	17951013	17951175	JAK3	693_selected_genes
chr19	17952172	17952380	JAK3	693_selected_genes
chr19	17952423	17952596	JAK3	693_selected_genes
chr19	17953099	17953444	JAK3	693_selected_genes
chr19	17953810	17954006	JAK3	693_selected_genes
chr19	17954163	17954325	JAK3	693_selected_genes
chr19	17954560	17954734	JAK3	693_selected_genes
chr19	17955017	17955251	JAK3	693_selected_genes
chr1	59247721	59248767	JUN	693_selected_genes
chr3	123798844	123798964	KALRN	693_selected_genes
chr3	123801074	123801204	KALRN	693_selected_genes
chr3	123813659	123813776	KALRN	693_selected_genes
chr3	123946811	123946936	KALRN	693_selected_genes
chr3	123953650	123953815	KALRN	693_selected_genes
chr3	123983319	123983562	KALRN	693_selected_genes
chr3	123987564	123988127	KALRN	693_selected_genes
chr3	124017612	124017785	KALRN	693_selected_genes
chr3	124044801	124045043	KALRN	693_selected_genes
chr3	124048682	124048864	KALRN	693_selected_genes
chr3	124053086	124053367	KALRN	693_selected_genes
chr3	124065964	124066137	KALRN	693_selected_genes
chr3	124103666	124103908	KALRN	693_selected_genes
chr3	124113956	124114270	KALRN	693_selected_genes
chr3	124117518	124117743	KALRN	693_selected_genes
chr3	124132291	124132537	KALRN	693_selected_genes
chr3	124141633	124141850	KALRN	693_selected_genes
chr3	124149477	124149647	KALRN	693_selected_genes
chr3	124153128	124153397	KALRN	693_selected_genes
chr3	124157709	124157909	KALRN	693_selected_genes
chr3	124160766	124160931	KALRN	693_selected_genes
chr3	124164982	124165148	KALRN	693_selected_genes
chr3	124165584	124165757	KALRN	693_selected_genes
chr3	124173998	124174231	KALRN	693_selected_genes
chr3	124175431	124175600	KALRN	693_selected_genes
chr3	124180711	124180828	KALRN	693_selected_genes
chr3	124181345	124181505	KALRN	693_selected_genes
chr3	124193484	124193604	KALRN	693_selected_genes
chr3	124196066	124196206	KALRN	693_selected_genes
chr3	124201629	124201772	KALRN	693_selected_genes
chr3	124207025	124207187	KALRN	693_selected_genes
chr3	124209515	124209756	KALRN	693_selected_genes
chr3	124210144	124210296	KALRN	693_selected_genes
chr3	124211561	124211754	KALRN	693_selected_genes
chr3	124215132	124215285	KALRN	693_selected_genes
chr3	124223724	124223822	KALRN	693_selected_genes
chr3	124237221	124237334	KALRN	693_selected_genes
chr3	124281664	124281961	KALRN	693_selected_genes
chr3	124303643	124303778	KALRN	693_selected_genes
chr3	124351241	124351575	KALRN	693_selected_genes
chr3	124352670	124352825	KALRN	693_selected_genes
chr3	124356029	124356175	KALRN	693_selected_genes
chr3	124369629	124369810	KALRN	693_selected_genes
chr3	124374422	124374539	KALRN	693_selected_genes
chr3	124376269	124376423	KALRN	693_selected_genes
chr3	124376555	124376675	KALRN	693_selected_genes
chr3	124377252	124377389	KALRN	693_selected_genes
chr3	124378186	124378329	KALRN	693_selected_genes
chr3	124379744	124379845	KALRN	693_selected_genes
chr3	124380672	124380800	KALRN	693_selected_genes
chr3	124385270	124385506	KALRN	693_selected_genes
chr3	124385833	124386055	KALRN	693_selected_genes

chr3	124390481	124390770	KALRN	693_selected_genes
chr3	124393182	124393490	KALRN	693_selected_genes
chr3	124396385	124396479	KALRN	693_selected_genes
chr3	124397011	124397185	KALRN	693_selected_genes
chr3	124398279	124398533	KALRN	693_selected_genes
chr3	124412625	124412703	KALRN	693_selected_genes
chr3	124413153	124413375	KALRN	693_selected_genes
chr3	124414955	124415127	KALRN	693_selected_genes
chr3	124416414	124416596	KALRN	693_selected_genes
chr3	124418690	124418905	KALRN	693_selected_genes
chr3	124420859	124420988	KALRN	693_selected_genes
chr3	124431756	124432007	KALRN	693_selected_genes
chr3	124436068	124436257	KALRN	693_selected_genes
chr3	124437746	124438342	KALRN	693_selected_genes
chr8	41789697	41792410	KAT6A	693_selected_genes
chr8	41794748	41795111	KAT6A	693_selected_genes
chr8	41798334	41798987	KAT6A	693_selected_genes
chr8	41800273	41800543	KAT6A	693_selected_genes
chr8	41801240	41801522	KAT6A	693_selected_genes
chr8	41804083	41804227	KAT6A	693_selected_genes
chr8	41805243	41805455	KAT6A	693_selected_genes
chr8	41806714	41806906	KAT6A	693_selected_genes
chr8	41812788	41812954	KAT6A	693_selected_genes
chr8	41814541	41814813	KAT6A	693_selected_genes
chr8	41832196	41832365	KAT6A	693_selected_genes
chr8	41834500	41834870	KAT6A	693_selected_genes
chr8	41836134	41836320	KAT6A	693_selected_genes
chr8	41838338	41838470	KAT6A	693_selected_genes
chr8	41839331	41839497	KAT6A	693_selected_genes
chr8	41844947	41845106	KAT6A	693_selected_genes
chr8	41905870	41906520	KAT6A	693_selected_genes
chr7	150642427	150642627	KCNH2	693_selected_genes
chr7	150643939	150644167	KCNH2	693_selected_genes
chr7	150644390	150644627	KCNH2	693_selected_genes
chr7	150644668	150644991	KCNH2	693_selected_genes
chr7	150645506	150645656	KCNH2	693_selected_genes
chr7	150645918	150646162	KCNH2	693_selected_genes
chr7	150646961	150647533	KCNH2	693_selected_genes
chr7	150647983	150648233	KCNH2	693_selected_genes
chr7	150648510	150648948	KCNH2	693_selected_genes
chr7	150649487	150649966	KCNH2	693_selected_genes
chr7	150652458	150652616	KCNH2	693_selected_genes
chr7	150654353	150654615	KCNH2	693_selected_genes
chr7	150655121	150655615	KCNH2	693_selected_genes
chr7	150656634	150656868	KCNH2	693_selected_genes
chr7	150671773	150672054	KCNH2	693_selected_genes
chr7	150674900	150675026	KCNH2	693_selected_genes
chr11	2465890	2465963	KCNQ1	693_selected_genes
chr11	2466303	2466739	KCNQ1	693_selected_genes
chr11	2482914	2482969	KCNQ1	693_selected_genes
chr11	2483098	2483232	KCNQ1	693_selected_genes
chr11	2542683	2542825	KCNQ1	693_selected_genes
chr11	2549132	2549273	KCNQ1	693_selected_genes
chr11	2591832	2592009	KCNQ1	693_selected_genes
chr11	2592529	2592658	KCNQ1	693_selected_genes
chr11	2593217	2593364	KCNQ1	693_selected_genes
chr11	2594050	2594241	KCNQ1	693_selected_genes
chr11	2604639	2604800	KCNQ1	693_selected_genes
chr11	2606416	2606562	KCNQ1	693_selected_genes
chr11	2608774	2608947	KCNQ1	693_selected_genes
chr11	2609917	2610109	KCNQ1	693_selected_genes
chr11	2683165	2683336	KCNQ1	693_selected_genes
chr11	2790048	2790174	KCNQ1	693_selected_genes
chr11	2797164	2797309	KCNQ1	693_selected_genes
chr11	2798190	2798287	KCNQ1	693_selected_genes
chr11	2799180	2799292	KCNQ1	693_selected_genes
chr11	2868971	2869258	KCNQ1	693_selected_genes
chr12	394596	394853	KDM5A	693_selected_genes
chr12	395303	395398	KDM5A	693_selected_genes
chr12	401899	402360	KDM5A	693_selected_genes
chr12	404713	404984	KDM5A	693_selected_genes
chr12	406181	406391	KDM5A	693_selected_genes
chr12	416086	416280	KDM5A	693_selected_genes
chr12	416594	417196	KDM5A	693_selected_genes
chr12	418943	419155	KDM5A	693_selected_genes
chr12	420025	420255	KDM5A	693_selected_genes
chr12	422196	422385	KDM5A	693_selected_genes
chr12	427246	427652	KDM5A	693_selected_genes
chr12	430135	430300	KDM5A	693_selected_genes
chr12	431557	431758	KDM5A	693_selected_genes
chr12	432222	432397	KDM5A	693_selected_genes
chr12	432740	432972	KDM5A	693_selected_genes
chr12	437975	438220	KDM5A	693_selected_genes
chr12	440959	441129	KDM5A	693_selected_genes

chr12	442627	442840	KDM5A	693_selected_genes
chr12	443381	443613	KDM5A	693_selected_genes
chr12	459761	459970	KDM5A	693_selected_genes
chr12	461345	461515	KDM5A	693_selected_genes
chr12	463216	463425	KDM5A	693_selected_genes
chr12	464298	464440	KDM5A	693_selected_genes
chr12	465572	465728	KDM5A	693_selected_genes
chr12	472103	472288	KDM5A	693_selected_genes
chr12	475074	475295	KDM5A	693_selected_genes
chr12	493171	493344	KDM5A	693_selected_genes
chr12	495037	495165	KDM5A	693_selected_genes
chr12	496388	496465	KDM5A	693_selected_genes
chr12	497373	497531	KDM5A	693_selected_genes
chr12	498067	498282	KDM5A	693_selected_genes
chrX	53221900	53222044	KDM5C	693_selected_genes
chrX	53222123	53222539	KDM5C	693_selected_genes
chrX	53222593	53222843	KDM5C	693_selected_genes
chrX	53222929	53223058	KDM5C	693_selected_genes
chrX	53223295	53223945	KDM5C	693_selected_genes
chrX	53224087	53224275	KDM5C	693_selected_genes
chrX	53224387	53224617	KDM5C	693_selected_genes
chrX	53225072	53225261	KDM5C	693_selected_genes
chrX	53225842	53226251	KDM5C	693_selected_genes
chrX	53226927	53227083	KDM5C	693_selected_genes
chrX	53227646	53227844	KDM5C	693_selected_genes
chrX	53227920	53228095	KDM5C	693_selected_genes
chrX	53228133	53228365	KDM5C	693_selected_genes
chrX	53230706	53230951	KDM5C	693_selected_genes
chrX	53231010	53231180	KDM5C	693_selected_genes
chrX	53239570	53239783	KDM5C	693_selected_genes
chrX	53239832	53240064	KDM5C	693_selected_genes
chrX	53240653	53240862	KDM5C	693_selected_genes
chrX	53240943	53241113	KDM5C	693_selected_genes
chrX	53243845	53244054	KDM5C	693_selected_genes
chrX	53244951	53245183	KDM5C	693_selected_genes
chrX	53245230	53245404	KDM5C	693_selected_genes
chrX	53246299	53246484	KDM5C	693_selected_genes
chrX	53246952	53247173	KDM5C	693_selected_genes
chrX	53247432	53247605	KDM5C	693_selected_genes
chrX	53247797	53247919	KDM5C	693_selected_genes
chrX	53249995	53250123	KDM5C	693_selected_genes
chrX	53250875	53251000	KDM5C	693_selected_genes
chrX	53253896	53254096	KDM5C	693_selected_genes
chrX	44732772	44732983	KDM6A	693_selected_genes
chrX	44733144	44733258	KDM6A	693_selected_genes
chrX	44820503	44820662	KDM6A	693_selected_genes
chrX	44833885	44833985	KDM6A	693_selected_genes
chrX	44870180	44870289	KDM6A	693_selected_genes
chrX	44879829	44880000	KDM6A	693_selected_genes
chrX	44894150	44894255	KDM6A	693_selected_genes
chrX	44896874	44896959	KDM6A	693_selected_genes
chrX	44910928	44911072	KDM6A	693_selected_genes
chrX	44913048	44913225	KDM6A	693_selected_genes
chrX	44918225	44918374	KDM6A	693_selected_genes
chrX	44918466	44918736	KDM6A	693_selected_genes
chrX	44919241	44919426	KDM6A	693_selected_genes
chrX	44919828	44920034	KDM6A	693_selected_genes
chrX	44920543	44920689	KDM6A	693_selected_genes
chrX	44921866	44922018	KDM6A	693_selected_genes
chrX	44922641	44923087	KDM6A	693_selected_genes
chrX	44928798	44929627	KDM6A	693_selected_genes
chrX	44935916	44936096	KDM6A	693_selected_genes
chrX	44937619	44937775	KDM6A	693_selected_genes
chrX	44938365	44938621	KDM6A	693_selected_genes
chrX	44941795	44941910	KDM6A	693_selected_genes
chrX	44941934	44942059	KDM6A	693_selected_genes
chrX	44942679	44942878	KDM6A	693_selected_genes
chrX	44945084	44945249	KDM6A	693_selected_genes
chrX	44948962	44949200	KDM6A	693_selected_genes
chrX	44949942	44950134	KDM6A	693_selected_genes
chrX	44965761	44965919	KDM6A	693_selected_genes
chrX	44966629	44966806	KDM6A	693_selected_genes
chrX	44969298	44969519	KDM6A	693_selected_genes
chrX	44970601	44970681	KDM6A	693_selected_genes
chr4	55946082	55946355	KDR	693_selected_genes
chr4	55948097	55948233	KDR	693_selected_genes
chr4	55948677	55948827	KDR	693_selected_genes
chr4	55953748	55953950	KDR	693_selected_genes
chr4	55955009	55955165	KDR	693_selected_genes
chr4	55955515	55955665	KDR	693_selected_genes
chr4	55955832	55955994	KDR	693_selected_genes
chr4	55956097	55956270	KDR	693_selected_genes
chr4	55958758	55958906	KDR	693_selected_genes
chr4	55960943	55961147	KDR	693_selected_genes

chr4	55961718	55961857	KDR	693_selected_genes
chr4	55962370	55962534	KDR	693_selected_genes
chr4	55963803	55963958	KDR	693_selected_genes
chr4	55964278	55964464	KDR	693_selected_genes
chr4	55964838	55964995	KDR	693_selected_genes
chr4	55968038	55968220	KDR	693_selected_genes
chr4	55968503	55968700	KDR	693_selected_genes
chr4	55970784	55971176	KDR	693_selected_genes
chr4	55971973	55972132	KDR	693_selected_genes
chr4	55972828	55973002	KDR	693_selected_genes
chr4	55973878	55974085	KDR	693_selected_genes
chr4	55976544	55976758	KDR	693_selected_genes
chr4	55976795	55976960	KDR	693_selected_genes
chr4	55979445	55979673	KDR	693_selected_genes
chr4	55980267	55980457	KDR	693_selected_genes
chr4	55981015	55981234	KDR	693_selected_genes
chr4	55981422	55981603	KDR	693_selected_genes
chr4	55984745	55984992	KDR	693_selected_genes
chr4	55987238	55987382	KDR	693_selected_genes
chr4	55991368	55991485	KDR	693_selected_genes
chr19	10597302	10597519	KEAP1	693_selected_genes
chr19	10599842	10600069	KEAP1	693_selected_genes
chr19	10600298	10600554	KEAP1	693_selected_genes
chr19	10602227	10602963	KEAP1	693_selected_genes
chr19	10610045	10610734	KEAP1	693_selected_genes
chr19	10613891	10614259	KEAP1	693_selected_genes
chr7	142638313	142638525	KEL	693_selected_genes
chr7	142639495	142639641	KEL	693_selected_genes
chr7	142639936	142640156	KEL	693_selected_genes
chr7	142640337	142640455	KEL	693_selected_genes
chr7	142640547	142640708	KEL	693_selected_genes
chr7	142640844	142640995	KEL	693_selected_genes
chr7	142641384	142641512	KEL	693_selected_genes
chr7	142641704	142641853	KEL	693_selected_genes
chr7	142643268	142643429	KEL	693_selected_genes
chr7	142649570	142649750	KEL	693_selected_genes
chr7	142650869	142651068	KEL	693_selected_genes
chr7	142651245	142651484	KEL	693_selected_genes
chr7	142651526	142651639	KEL	693_selected_genes
chr7	142654888	142655085	KEL	693_selected_genes
chr7	142655365	142655540	KEL	693_selected_genes
chr7	142657989	142658216	KEL	693_selected_genes
chr7	142658421	142658613	KEL	693_selected_genes
chr7	142658856	142658984	KEL	693_selected_genes
chr7	142659176	142659260	KEL	693_selected_genes
chr7	142659265	142659318	KEL	693_selected_genes
chr4	55524156	55524273	KIT	693_selected_genes
chr4	55561652	55561972	KIT	693_selected_genes
chr4	55564424	55564756	KIT	693_selected_genes
chr4	55565770	55565957	KIT	693_selected_genes
chr4	55569864	55570083	KIT	693_selected_genes
chr4	55573238	55573478	KIT	693_selected_genes
chr4	55575564	55575730	KIT	693_selected_genes
chr4	55589724	55589889	KIT	693_selected_genes
chr4	55591997	55592241	KIT	693_selected_genes
chr4	55593358	55593515	KIT	693_selected_genes
chr4	55593556	55593733	KIT	693_selected_genes
chr4	55593963	55594118	KIT	693_selected_genes
chr4	55594151	55594312	KIT	693_selected_genes
chr4	55595475	55595676	KIT	693_selected_genes
chr4	55597468	55597610	KIT	693_selected_genes
chr4	55598011	55598189	KIT	693_selected_genes
chr4	55599210	55599383	KIT	693_selected_genes
chr4	55602638	55602800	KIT	693_selected_genes
chr4	55602861	55603011	KIT	693_selected_genes
chr4	55603315	55603471	KIT	693_selected_genes
chr4	55604569	55604748	KIT	693_selected_genes
chr9	110248006	110248232	KLF4	693_selected_genes
chr9	110249283	110250573	KLF4	693_selected_genes
chr9	110251185	110251356	KLF4	693_selected_genes
chr9	110251423	110251478	KLF4	693_selected_genes
chr13	73633440	73633751	KLF5	693_selected_genes
chr13	73635973	73636897	KLF5	693_selected_genes
chr13	73637936	73638046	KLF5	693_selected_genes
chr13	73649820	73650049	KLF5	693_selected_genes
chr3	183209689	183210041	KLHL6	693_selected_genes
chr3	183210256	183210520	KLHL6	693_selected_genes
chr3	183211841	183212094	KLHL6	693_selected_genes
chr3	183217352	183217640	KLHL6	693_selected_genes
chr3	183225821	183226321	KLHL6	693_selected_genes
chr3	183245607	183245823	KLHL6	693_selected_genes
chr3	183273123	183273466	KLHL6	693_selected_genes
chr11	118307202	118307684	KMT2A	693_selected_genes
chr11	118309713	118309862	KMT2A	693_selected_genes

chr11	118318314	118318427	KMT2A	693_selected_genes
chr11	118339464	118339584	KMT2A	693_selected_genes
chr11	118342351	118345055	KMT2A	693_selected_genes
chr11	118347494	118347722	KMT2A	693_selected_genes
chr11	118348656	118348942	KMT2A	693_selected_genes
chr11	118350863	118350978	KMT2A	693_selected_genes
chr11	118352404	118352832	KMT2A	693_selected_genes
chr11	118353111	118353235	KMT2A	693_selected_genes
chr11	118354872	118355054	KMT2A	693_selected_genes
chr11	118355551	118355715	KMT2A	693_selected_genes
chr11	118359303	118359500	KMT2A	693_selected_genes
chr11	118360481	118360627	KMT2A	693_selected_genes
chr11	118360818	118360989	KMT2A	693_selected_genes
chr11	118361885	118362058	KMT2A	693_selected_genes
chr11	118362433	118362668	KMT2A	693_selected_genes
chr11	118363746	118363970	KMT2A	693_selected_genes
chr11	118364977	118365138	KMT2A	693_selected_genes
chr11	118365383	118365507	KMT2A	693_selected_genes
chr11	118366389	118366633	KMT2A	693_selected_genes
chr11	118366950	118367107	KMT2A	693_selected_genes
chr11	118368625	118368813	KMT2A	693_selected_genes
chr11	118369059	118369268	KMT2A	693_selected_genes
chr11	118369992	118370160	KMT2A	693_selected_genes
chr11	118370524	118370653	KMT2A	693_selected_genes
chr11	118371676	118371887	KMT2A	693_selected_genes
chr11	118372361	118372597	KMT2A	693_selected_genes
chr11	118373087	118377386	KMT2A	693_selected_genes
chr11	118378218	118378349	KMT2A	693_selected_genes
chr11	118379825	118379940	KMT2A	693_selected_genes
chr11	118380637	118380858	KMT2A	693_selected_genes
chr11	118382640	118382765	KMT2A	693_selected_genes
chr11	118390307	118390532	KMT2A	693_selected_genes
chr11	118390646	118390804	KMT2A	693_selected_genes
chr11	118391491	118391625	KMT2A	693_selected_genes
chr11	118391977	118392157	KMT2A	693_selected_genes
chr11	118392586	118392912	KMT2A	693_selected_genes
chr19	36208895	36209308	KMT2B	693_selected_genes
chr19	36210345	36210468	KMT2B	693_selected_genes
chr19	36210660	36212731	KMT2B	693_selected_genes
chr19	36213235	36213399	KMT2B	693_selected_genes
chr19	36213444	36213645	KMT2B	693_selected_genes
chr19	36213871	36214201	KMT2B	693_selected_genes
chr19	36214323	36214430	KMT2B	693_selected_genes
chr19	36214607	36214933	KMT2B	693_selected_genes
chr19	36215512	36215657	KMT2B	693_selected_genes
chr19	36215864	36216013	KMT2B	693_selected_genes
chr19	36216095	36216259	KMT2B	693_selected_genes
chr19	36216354	36216551	KMT2B	693_selected_genes
chr19	36216598	36216744	KMT2B	693_selected_genes
chr19	36217111	36217279	KMT2B	693_selected_genes
chr19	36218031	36218195	KMT2B	693_selected_genes
chr19	36218313	36218548	KMT2B	693_selected_genes
chr19	36218573	36218713	KMT2B	693_selected_genes
chr19	36218756	36218911	KMT2B	693_selected_genes
chr19	36218973	36219097	KMT2B	693_selected_genes
chr19	36219649	36219815	KMT2B	693_selected_genes
chr19	36219860	36220002	KMT2B	693_selected_genes
chr19	36220034	36220222	KMT2B	693_selected_genes
chr19	36220842	36221051	KMT2B	693_selected_genes
chr19	36221217	36221388	KMT2B	693_selected_genes
chr19	36221413	36221542	KMT2B	693_selected_genes
chr19	36221582	36221793	KMT2B	693_selected_genes
chr19	36222783	36223061	KMT2B	693_selected_genes
chr19	36223090	36224434	KMT2B	693_selected_genes
chr19	36224472	36224612	KMT2B	693_selected_genes
chr19	36224638	36224798	KMT2B	693_selected_genes
chr19	36227565	36227753	KMT2B	693_selected_genes
chr19	36227787	36227912	KMT2B	693_selected_genes
chr19	36227961	36228189	KMT2B	693_selected_genes
chr19	36228511	36228669	KMT2B	693_selected_genes
chr19	36228734	36228868	KMT2B	693_selected_genes
chr19	36228937	36229117	KMT2B	693_selected_genes
chr19	36229157	36229483	KMT2B	693_selected_genes
chr7	151833891	151834034	KMT2C	693_selected_genes
chr7	151835843	151836014	KMT2C	693_selected_genes
chr7	151836245	151836369	KMT2C	693_selected_genes
chr7	151836734	151836901	KMT2C	693_selected_genes
chr7	151841772	151841991	KMT2C	693_selected_genes
chr7	151842212	151842405	KMT2C	693_selected_genes
chr7	151843658	151843845	KMT2C	693_selected_genes
chr7	151845092	151846262	KMT2C	693_selected_genes
chr7	151847959	151848117	KMT2C	693_selected_genes
chr7	151848501	151848691	KMT2C	693_selected_genes
chr7	151849764	151850064	KMT2C	693_selected_genes

chr7	151851069	151851256	KMT2C	693_selected_genes
chr7	151851326	151851555	KMT2C	693_selected_genes
chr7	151852969	151853167	KMT2C	693_selected_genes
chr7	151853264	151853456	KMT2C	693_selected_genes
chr7	151854820	151855035	KMT2C	693_selected_genes
chr7	151855916	151856182	KMT2C	693_selected_genes
chr7	151859176	151860936	KMT2C	693_selected_genes
chr7	151864205	151864488	KMT2C	693_selected_genes
chr7	151866245	151866359	KMT2C	693_selected_genes
chr7	151868323	151868452	KMT2C	693_selected_genes
chr7	151871190	151871352	KMT2C	693_selected_genes
chr7	151873250	151875120	KMT2C	693_selected_genes
chr7	151876893	151877236	KMT2C	693_selected_genes
chr7	151877770	151879704	KMT2C	693_selected_genes
chr7	151880033	151880266	KMT2C	693_selected_genes
chr7	151882617	151882741	KMT2C	693_selected_genes
chr7	151884321	151884586	KMT2C	693_selected_genes
chr7	151884774	151884957	KMT2C	693_selected_genes
chr7	151891068	151891238	KMT2C	693_selected_genes
chr7	151891288	151891371	KMT2C	693_selected_genes
chr7	151891499	151891678	KMT2C	693_selected_genes
chr7	151892966	151893121	KMT2C	693_selected_genes
chr7	151896338	151896569	KMT2C	693_selected_genes
chr7	151899993	151900174	KMT2C	693_selected_genes
chr7	151902165	151902335	KMT2C	693_selected_genes
chr7	151904359	151904538	KMT2C	693_selected_genes
chr7	151917582	151917845	KMT2C	693_selected_genes
chr7	151919060	151919176	KMT2C	693_selected_genes
chr7	151919632	151919792	KMT2C	693_selected_genes
chr7	151921074	151921289	KMT2C	693_selected_genes
chr7	151921494	151921726	KMT2C	693_selected_genes
chr7	151926982	151927137	KMT2C	693_selected_genes
chr7	151927279	151927431	KMT2C	693_selected_genes
chr7	151932876	151933043	KMT2C	693_selected_genes
chr7	151935766	151935936	KMT2C	693_selected_genes
chr7	151944961	151945730	KMT2C	693_selected_genes
chr7	151946935	151947063	KMT2C	693_selected_genes
chr7	151947912	151948076	KMT2C	693_selected_genes
chr7	151948998	151949200	KMT2C	693_selected_genes
chr7	151949605	151949825	KMT2C	693_selected_genes
chr7	151960075	151960240	KMT2C	693_selected_genes
chr7	151962097	151962319	KMT2C	693_selected_genes
chr7	151970764	151970977	KMT2C	693_selected_genes
chr7	152007025	152007185	KMT2C	693_selected_genes
chr7	152008857	152009056	KMT2C	693_selected_genes
chr7	152012197	152012448	KMT2C	693_selected_genes
chr7	152027660	152027852	KMT2C	693_selected_genes
chr7	152055646	152055785	KMT2C	693_selected_genes
chr7	152132685	152132896	KMT2C	693_selected_genes
chr12	49415537	49415680	KMT2D	693_selected_genes
chr12	49415800	49415959	KMT2D	693_selected_genes
chr12	49416037	49416161	KMT2D	693_selected_genes
chr12	49416347	49416683	KMT2D	693_selected_genes
chr12	49417810	49417908	KMT2D	693_selected_genes
chr12	49418335	49418516	KMT2D	693_selected_genes
chr12	49418567	49418754	KMT2D	693_selected_genes
chr12	49419939	49421130	KMT2D	693_selected_genes
chr12	49421560	49421738	KMT2D	693_selected_genes
chr12	49421766	49421949	KMT2D	693_selected_genes
chr12	49422585	49422766	KMT2D	693_selected_genes
chr12	49422818	49423044	KMT2D	693_selected_genes
chr12	49423158	49423284	KMT2D	693_selected_genes
chr12	49424037	49424247	KMT2D	693_selected_genes
chr12	49424358	49424576	KMT2D	693_selected_genes
chr12	49424650	49424841	KMT2D	693_selected_genes
chr12	49424932	49427772	KMT2D	693_selected_genes
chr12	49427824	49428107	KMT2D	693_selected_genes
chr12	49428167	49428284	KMT2D	693_selected_genes
chr12	49428339	49428474	KMT2D	693_selected_genes
chr12	49428569	49428743	KMT2D	693_selected_genes
chr12	49430882	49432797	KMT2D	693_selected_genes
chr12	49432979	49433166	KMT2D	693_selected_genes
chr12	49433192	49433425	KMT2D	693_selected_genes
chr12	49433481	49435343	KMT2D	693_selected_genes
chr12	49435412	49435513	KMT2D	693_selected_genes
chr12	49435674	49435798	KMT2D	693_selected_genes
chr12	49435846	49436138	KMT2D	693_selected_genes
chr12	49436318	49436453	KMT2D	693_selected_genes
chr12	49436498	49436686	KMT2D	693_selected_genes
chr12	49436833	49436994	KMT2D	693_selected_genes
chr12	49437120	49437236	KMT2D	693_selected_genes
chr12	49437392	49437590	KMT2D	693_selected_genes
chr12	49437625	49437806	KMT2D	693_selected_genes
chr12	49437957	49438112	KMT2D	693_selected_genes

chr12	49438160	49438330	KMT2D	693_selected_genes
chr12	49438501	49438773	KMT2D	693_selected_genes
chr12	49439677	49439775	KMT2D	693_selected_genes
chr12	49439822	49439982	KMT2D	693_selected_genes
chr12	49440017	49440232	KMT2D	693_selected_genes
chr12	49440366	49440598	KMT2D	693_selected_genes
chr12	49441722	49441877	KMT2D	693_selected_genes
chr12	49442416	49442577	KMT2D	693_selected_genes
chr12	49442862	49443026	KMT2D	693_selected_genes
chr12	49443439	49444598	KMT2D	693_selected_genes
chr12	49444643	49446232	KMT2D	693_selected_genes
chr12	49446321	49446517	KMT2D	693_selected_genes
chr12	49446672	49446880	KMT2D	693_selected_genes
chr12	49446964	49447129	KMT2D	693_selected_genes
chr12	49447233	49447449	KMT2D	693_selected_genes
chr12	49447735	49447948	KMT2D	693_selected_genes
chr12	49448064	49448224	KMT2D	693_selected_genes
chr12	49448285	49448559	KMT2D	693_selected_genes
chr12	49448657	49448834	KMT2D	693_selected_genes
chr12	49449033	49449132	KMT2D	693_selected_genes
chr12	25362703	25362870	KRAS	693_selected_genes
chr12	25368349	25368519	KRAS	693_selected_genes
chr12	25378522	25378732	KRAS	693_selected_genes
chr12	25380142	25380371	KRAS	693_selected_genes
chr12	25388114	25388185	KRAS	693_selected_genes
chr12	25398182	25398343	KRAS	693_selected_genes
chr6	129204365	129204527	LAMA2	693_selected_genes
chr6	129371037	129371258	LAMA2	693_selected_genes
chr6	129380903	129381066	LAMA2	693_selected_genes
chr6	129419292	129419585	LAMA2	693_selected_genes
chr6	129465020	129465250	LAMA2	693_selected_genes
chr6	129468078	129468218	LAMA2	693_selected_genes
chr6	129470098	129470266	LAMA2	693_selected_genes
chr6	129475624	129475853	LAMA2	693_selected_genes
chr6	129486695	129486845	LAMA2	693_selected_genes
chr6	129498825	129499036	LAMA2	693_selected_genes
chr6	129511324	129511515	LAMA2	693_selected_genes
chr6	129513799	129514023	LAMA2	693_selected_genes
chr6	129571231	129571383	LAMA2	693_selected_genes
chr6	129573203	129573465	LAMA2	693_selected_genes
chr6	129581830	129581992	LAMA2	693_selected_genes
chr6	129588225	129588389	LAMA2	693_selected_genes
chr6	129591743	129591921	LAMA2	693_selected_genes
chr6	129601180	129601317	LAMA2	693_selected_genes
chr6	129608966	129609228	LAMA2	693_selected_genes
chr6	129612733	129612890	LAMA2	693_selected_genes
chr6	129618804	129619035	LAMA2	693_selected_genes
chr6	129621855	129622042	LAMA2	693_selected_genes
chr6	129633980	129634267	LAMA2	693_selected_genes
chr6	129635774	129635968	LAMA2	693_selected_genes
chr6	129636595	129636825	LAMA2	693_selected_genes
chr6	129636881	129637120	LAMA2	693_selected_genes
chr6	129637157	129637341	LAMA2	693_selected_genes
chr6	129641657	129641825	LAMA2	693_selected_genes
chr6	129649397	129649582	LAMA2	693_selected_genes
chr6	129663462	129663637	LAMA2	693_selected_genes
chr6	129670417	129670554	LAMA2	693_selected_genes
chr6	129674283	129674527	LAMA2	693_selected_genes
chr6	129687338	129687531	LAMA2	693_selected_genes
chr6	129691011	129691160	LAMA2	693_selected_genes
chr6	129704241	129704403	LAMA2	693_selected_genes
chr6	129712610	129712823	LAMA2	693_selected_genes
chr6	129714164	129714425	LAMA2	693_selected_genes
chr6	129722343	129722510	LAMA2	693_selected_genes
chr6	129723443	129723657	LAMA2	693_selected_genes
chr6	129724940	129725129	LAMA2	693_selected_genes
chr6	129748871	129749024	LAMA2	693_selected_genes
chr6	129759765	129759932	LAMA2	693_selected_genes
chr6	129761935	129762168	LAMA2	693_selected_genes
chr6	129763341	129763397	LAMA2	693_selected_genes
chr6	129764182	129764238	LAMA2	693_selected_genes
chr6	129766786	129766991	LAMA2	693_selected_genes
chr6	129774107	129774301	LAMA2	693_selected_genes
chr6	129775274	129775458	LAMA2	693_selected_genes
chr6	129777454	129777664	LAMA2	693_selected_genes
chr6	129781319	129781494	LAMA2	693_selected_genes
chr6	129785409	129785622	LAMA2	693_selected_genes
chr6	129786264	129786459	LAMA2	693_selected_genes
chr6	129794333	129794522	LAMA2	693_selected_genes
chr6	129796509	129796571	LAMA2	693_selected_genes
chr6	129799812	129799983	LAMA2	693_selected_genes
chr6	129802382	129802609	LAMA2	693_selected_genes
chr6	129807593	129807792	LAMA2	693_selected_genes
chr6	129813020	129813247	LAMA2	693_selected_genes

chr6	129813434	129813653	LAMA2	693_selected_genes
chr6	129823778	129823941	LAMA2	693_selected_genes
chr6	129824210	129824450	LAMA2	693_selected_genes
chr6	129826319	129826525	LAMA2	693_selected_genes
chr6	129828608	129828812	LAMA2	693_selected_genes
chr6	129833482	129833663	LAMA2	693_selected_genes
chr6	129835492	129835765	LAMA2	693_selected_genes
chr6	129837309	129837517	LAMA2	693_selected_genes
chr6	149982839	149983399	LATS1	693_selected_genes
chr6	149997370	149997527	LATS1	693_selected_genes
chr6	149997665	149997898	LATS1	693_selected_genes
chr6	150000985	150001618	LATS1	693_selected_genes
chr6	150004126	150005753	LATS1	693_selected_genes
chr6	150016184	150016382	LATS1	693_selected_genes
chr6	150018237	150018347	LATS1	693_selected_genes
chr6	150022889	150023287	LATS1	693_selected_genes
chr6	150038959	150039195	LATS1	693_selected_genes
chr13	21548983	21549528	LATS2	693_selected_genes
chr13	21553804	21553961	LATS2	693_selected_genes
chr13	21555579	21555812	LATS2	693_selected_genes
chr13	21557337	21557970	LATS2	693_selected_genes
chr13	21561994	21563468	LATS2	693_selected_genes
chr13	21565385	21565568	LATS2	693_selected_genes
chr13	21619798	21620190	LATS2	693_selected_genes
chr13	46701700	46701883	LCP1	693_selected_genes
chr13	46704923	46705098	LCP1	693_selected_genes
chr13	46708236	46708410	LCP1	693_selected_genes
chr13	46716401	46716585	LCP1	693_selected_genes
chr13	46716835	46716960	LCP1	693_selected_genes
chr13	46717399	46717564	LCP1	693_selected_genes
chr13	46718551	46718680	LCP1	693_selected_genes
chr13	46721017	46721263	LCP1	693_selected_genes
chr13	46722461	46722607	LCP1	693_selected_genes
chr13	46725045	46725238	LCP1	693_selected_genes
chr13	46726889	46727105	LCP1	693_selected_genes
chr13	46728914	46729046	LCP1	693_selected_genes
chr13	46730547	46730730	LCP1	693_selected_genes
chr13	46732631	46732811	LCP1	693_selected_genes
chr13	46732935	46733149	LCP1	693_selected_genes
chr13	46733708	46733822	LCP1	693_selected_genes
chr19	11200199	11200316	LDLR	693_selected_genes
chr19	11201249	11201620	LDLR	693_selected_genes
chr19	11210873	11211046	LDLR	693_selected_genes
chr19	11213314	11213487	LDLR	693_selected_genes
chr19	11215870	11216301	LDLR	693_selected_genes
chr19	11217215	11217388	LDLR	693_selected_genes
chr19	11218042	11218215	LDLR	693_selected_genes
chr19	11221302	11221472	LDLR	693_selected_genes
chr19	11222164	11222340	LDLR	693_selected_genes
chr19	11223928	11224150	LDLR	693_selected_genes
chr19	11224185	11224463	LDLR	693_selected_genes
chr19	11226744	11226913	LDLR	693_selected_genes
chr19	11227509	11227699	LDLR	693_selected_genes
chr19	11230742	11230934	LDLR	693_selected_genes
chr19	11231020	11231223	LDLR	693_selected_genes
chr19	11233824	11234045	LDLR	693_selected_genes
chr19	11238658	11238786	LDLR	693_selected_genes
chr19	11240101	11240371	LDLR	693_selected_genes
chr19	11241931	11242219	LDLR	693_selected_genes
chr5	38481671	38482345	LIFR	693_selected_genes
chr5	38482665	38482794	LIFR	693_selected_genes
chr5	38484851	38484995	LIFR	693_selected_genes
chr5	38485895	38486107	LIFR	693_selected_genes
chr5	38489154	38489372	LIFR	693_selected_genes
chr5	38490266	38490418	LIFR	693_selected_genes
chr5	38493682	38493912	LIFR	693_selected_genes
chr5	38496458	38496722	LIFR	693_selected_genes
chr5	38499589	38499710	LIFR	693_selected_genes
chr5	38502713	38502926	LIFR	693_selected_genes
chr5	38504052	38504248	LIFR	693_selected_genes
chr5	38505981	38506201	LIFR	693_selected_genes
chr5	38506579	38506759	LIFR	693_selected_genes
chr5	38510540	38510845	LIFR	693_selected_genes
chr5	38511866	38512091	LIFR	693_selected_genes
chr5	38523495	38523709	LIFR	693_selected_genes
chr5	38527231	38527421	LIFR	693_selected_genes
chr5	38528802	38528967	LIFR	693_selected_genes
chr5	38530582	38530774	LIFR	693_selected_genes
chr1	156084684	156085090	LMNA	693_selected_genes
chr1	156095969	156096039	LMNA	693_selected_genes
chr1	156096310	156096467	LMNA	693_selected_genes
chr1	156096568	156096735	LMNA	693_selected_genes
chr1	156099615	156099724	LMNA	693_selected_genes
chr1	156100382	156100589	LMNA	693_selected_genes

chr1	156104168	156104344	LMNA	693_selected_genes
chr1	156104570	156104791	LMNA	693_selected_genes
chr1	156104952	156105128	LMNA	693_selected_genes
chr1	156105666	156105937	LMNA	693_selected_genes
chr1	156105979	156106252	LMNA	693_selected_genes
chr1	156106686	156106844	LMNA	693_selected_genes
chr1	156106878	156107048	LMNA	693_selected_genes
chr1	156107419	156107580	LMNA	693_selected_genes
chr1	156108253	156108573	LMNA	693_selected_genes
chr1	156108845	156108922	LMNA	693_selected_genes
chr1	156109535	156109655	LMNA	693_selected_genes
chr11	8246137	8246293	LMO1	693_selected_genes
chr11	8248496	8248672	LMO1	693_selected_genes
chr11	8251812	8252076	LMO1	693_selected_genes
chr11	8284859	8284934	LMO1	693_selected_genes
chr11	8289948	8290020	LMO1	693_selected_genes
chr3	188123883	188124126	LPP	693_selected_genes
chr3	188202354	188202517	LPP	693_selected_genes
chr3	188242427	188242600	LPP	693_selected_genes
chr3	188326923	188327657	LPP	693_selected_genes
chr3	188426029	188426253	LPP	693_selected_genes
chr3	188464250	188464308	LPP	693_selected_genes
chr3	188477875	188478095	LPP	693_selected_genes
chr3	188583962	188584191	LPP	693_selected_genes
chr3	188590405	188590576	LPP	693_selected_genes
chr3	188592113	188592292	LPP	693_selected_genes
chr2	140990729	140990920	LRP1B	693_selected_genes
chr2	140992329	140992478	LRP1B	693_selected_genes
chr2	140995695	140995890	LRP1B	693_selected_genes
chr2	140996985	140997126	LRP1B	693_selected_genes
chr2	141004629	141004756	LRP1B	693_selected_genes
chr2	141026811	141026975	LRP1B	693_selected_genes
chr2	141027785	141027940	LRP1B	693_selected_genes
chr2	141031967	141032192	LRP1B	693_selected_genes
chr2	141055351	141055563	LRP1B	693_selected_genes
chr2	141072478	141072693	LRP1B	693_selected_genes
chr2	141079506	141079682	LRP1B	693_selected_genes
chr2	141081436	141081660	LRP1B	693_selected_genes
chr2	141083305	141083472	LRP1B	693_selected_genes
chr2	141091996	141092153	LRP1B	693_selected_genes
chr2	141093158	141093432	LRP1B	693_selected_genes
chr2	141108340	141108632	LRP1B	693_selected_genes
chr2	141110496	141110666	LRP1B	693_selected_genes
chr2	141113885	141114070	LRP1B	693_selected_genes
chr2	141115522	141115710	LRP1B	693_selected_genes
chr2	141116364	141116540	LRP1B	693_selected_genes
chr2	141122204	141122377	LRP1B	693_selected_genes
chr2	141128253	141128436	LRP1B	693_selected_genes
chr2	141128722	141128879	LRP1B	693_selected_genes
chr2	141130551	141130731	LRP1B	693_selected_genes
chr2	141135723	141135880	LRP1B	693_selected_genes
chr2	141143436	141143603	LRP1B	693_selected_genes
chr2	141200047	141200217	LRP1B	693_selected_genes
chr2	141201873	141202043	LRP1B	693_selected_genes
chr2	141202106	141202273	LRP1B	693_selected_genes
chr2	141208111	141208255	LRP1B	693_selected_genes
chr2	141213998	141214197	LRP1B	693_selected_genes
chr2	141215006	141215245	LRP1B	693_selected_genes
chr2	141232681	141232931	LRP1B	693_selected_genes
chr2	141242886	141243118	LRP1B	693_selected_genes
chr2	141245160	141245333	LRP1B	693_selected_genes
chr2	141250151	141250287	LRP1B	693_selected_genes
chr2	141253108	141253342	LRP1B	693_selected_genes
chr2	141259230	141259468	LRP1B	693_selected_genes
chr2	141260506	141260697	LRP1B	693_selected_genes
chr2	141264339	141264512	LRP1B	693_selected_genes
chr2	141267471	141267650	LRP1B	693_selected_genes
chr2	141272196	141272366	LRP1B	693_selected_genes
chr2	141274432	141274605	LRP1B	693_selected_genes
chr2	141283387	141283587	LRP1B	693_selected_genes
chr2	141283780	141283944	LRP1B	693_selected_genes
chr2	141291564	141291734	LRP1B	693_selected_genes
chr2	141294124	141294303	LRP1B	693_selected_genes
chr2	141298516	141298692	LRP1B	693_selected_genes
chr2	141299322	141299565	LRP1B	693_selected_genes
chr2	141356174	141356429	LRP1B	693_selected_genes
chr2	141358993	141359233	LRP1B	693_selected_genes
chr2	141457793	141458215	LRP1B	693_selected_genes
chr2	141459264	141459439	LRP1B	693_selected_genes
chr2	141459684	141459886	LRP1B	693_selected_genes
chr2	141459970	141460147	LRP1B	693_selected_genes
chr2	141473516	141473696	LRP1B	693_selected_genes
chr2	141474225	141474410	LRP1B	693_selected_genes
chr2	141526756	141526938	LRP1B	693_selected_genes

chr2	141528424	141528600	LRP1B	693_selected_genes
chr2	141533641	141533832	LRP1B	693_selected_genes
chr2	141571200	141571400	LRP1B	693_selected_genes
chr2	141597534	141597679	LRP1B	693_selected_genes
chr2	141598461	141598686	LRP1B	693_selected_genes
chr2	141607645	141607923	LRP1B	693_selected_genes
chr2	141609195	141609377	LRP1B	693_selected_genes
chr2	141625133	141625428	LRP1B	693_selected_genes
chr2	141625642	141625857	LRP1B	693_selected_genes
chr2	141641360	141641615	LRP1B	693_selected_genes
chr2	141643681	141643929	LRP1B	693_selected_genes
chr2	141660463	141660759	LRP1B	693_selected_genes
chr2	141665420	141665671	LRP1B	693_selected_genes
chr2	141680508	141680741	LRP1B	693_selected_genes
chr2	141707778	141707996	LRP1B	693_selected_genes
chr2	141709403	141709534	LRP1B	693_selected_genes
chr2	141739703	141739870	LRP1B	693_selected_genes
chr2	141747075	141747251	LRP1B	693_selected_genes
chr2	141751538	141751729	LRP1B	693_selected_genes
chr2	141762878	141763051	LRP1B	693_selected_genes
chr2	141771099	141771339	LRP1B	693_selected_genes
chr2	141773239	141773509	LRP1B	693_selected_genes
chr2	141777465	141777696	LRP1B	693_selected_genes
chr2	141806529	141806816	LRP1B	693_selected_genes
chr2	141812659	141812853	LRP1B	693_selected_genes
chr2	141816426	141816648	LRP1B	693_selected_genes
chr2	141819594	141819867	LRP1B	693_selected_genes
chr2	141945964	141946177	LRP1B	693_selected_genes
chr2	141986726	141987034	LRP1B	693_selected_genes
chr2	142004769	142004948	LRP1B	693_selected_genes
chr2	142012065	142012235	LRP1B	693_selected_genes
chr2	142237939	142238127	LRP1B	693_selected_genes
chr2	142567822	142567995	LRP1B	693_selected_genes
chr2	142888191	142888323	LRP1B	693_selected_genes
chr12	40618908	40619109	LRRK2	693_selected_genes
chr12	40619331	40619467	LRRK2	693_selected_genes
chr12	40626050	40626210	LRRK2	693_selected_genes
chr12	40629402	40629541	LRRK2	693_selected_genes
chr12	40631745	40631930	LRRK2	693_selected_genes
chr12	40634259	40634444	LRRK2	693_selected_genes
chr12	40637326	40637508	LRRK2	693_selected_genes
chr12	40643602	40643772	LRRK2	693_selected_genes
chr12	40645008	40645201	LRRK2	693_selected_genes
chr12	40645241	40645371	LRRK2	693_selected_genes
chr12	40646686	40646843	LRRK2	693_selected_genes
chr12	40651024	40651204	LRRK2	693_selected_genes
chr12	40653256	40653431	LRRK2	693_selected_genes
chr12	40657565	40657728	LRRK2	693_selected_genes
chr12	40668359	40668554	LRRK2	693_selected_genes
chr12	40668630	40668820	LRRK2	693_selected_genes
chr12	40671664	40671843	LRRK2	693_selected_genes
chr12	40671867	40672088	LRRK2	693_selected_genes
chr12	40677651	40677960	LRRK2	693_selected_genes
chr12	40681127	40681366	LRRK2	693_selected_genes
chr12	40687321	40687490	LRRK2	693_selected_genes
chr12	40688621	40688741	LRRK2	693_selected_genes
chr12	40689203	40689471	LRRK2	693_selected_genes
chr12	40692019	40692320	LRRK2	693_selected_genes
chr12	40692885	40693084	LRRK2	693_selected_genes
chr12	40694589	40694722	LRRK2	693_selected_genes
chr12	40696565	40696709	LRRK2	693_selected_genes
chr12	40697724	40698000	LRRK2	693_selected_genes
chr12	40699561	40699793	LRRK2	693_selected_genes
chr12	40702243	40702523	LRRK2	693_selected_genes
chr12	40702882	40703060	LRRK2	693_selected_genes
chr12	40704207	40704476	LRRK2	693_selected_genes
chr12	40707748	40708000	LRRK2	693_selected_genes
chr12	40708988	40709127	LRRK2	693_selected_genes
chr12	40713764	40714002	LRRK2	693_selected_genes
chr12	40714810	40715015	LRRK2	693_selected_genes
chr12	40715811	40716008	LRRK2	693_selected_genes
chr12	40716095	40716337	LRRK2	693_selected_genes
chr12	40716936	40717133	LRRK2	693_selected_genes
chr12	40722136	40722287	LRRK2	693_selected_genes
chr12	40728743	40728984	LRRK2	693_selected_genes
chr12	40734070	40734281	LRRK2	693_selected_genes
chr12	40740529	40740750	LRRK2	693_selected_genes
chr12	40742185	40742336	LRRK2	693_selected_genes
chr12	40745315	40745560	LRRK2	693_selected_genes
chr12	40748075	40748319	LRRK2	693_selected_genes
chr12	40749891	40750014	LRRK2	693_selected_genes
chr12	40753036	40753271	LRRK2	693_selected_genes
chr12	40757178	40757381	LRRK2	693_selected_genes
chr12	40758618	40758877	LRRK2	693_selected_genes

chr12	40760782	40760904	LRRK2	693_selected_genes
chr12	40761420	40761592	LRRK2	693_selected_genes
chr8	56854393	56854575	LYN	693_selected_genes
chr8	56858981	56859077	LYN	693_selected_genes
chr8	56860151	56860307	LYN	693_selected_genes
chr8	56862992	56863141	LYN	693_selected_genes
chr8	56863214	56863368	LYN	693_selected_genes
chr8	56864499	56864699	LYN	693_selected_genes
chr8	56866365	56866568	LYN	693_selected_genes
chr8	56879248	56879481	LYN	693_selected_genes
chr8	56882250	56882377	LYN	693_selected_genes
chr8	56910879	56911083	LYN	693_selected_genes
chr8	56911951	56912133	LYN	693_selected_genes
chr8	56922441	56922694	LYN	693_selected_genes
chr22	21336635	21336890	LZTR1	693_selected_genes
chr22	21337290	21337403	LZTR1	693_selected_genes
chr22	21340104	21340211	LZTR1	693_selected_genes
chr22	21341767	21341897	LZTR1	693_selected_genes
chr22	21342273	21342432	LZTR1	693_selected_genes
chr22	21343052	21343186	LZTR1	693_selected_genes
chr22	21343888	21343996	LZTR1	693_selected_genes
chr22	21344649	21344839	LZTR1	693_selected_genes
chr22	21345891	21346143	LZTR1	693_selected_genes
chr22	21346477	21346683	LZTR1	693_selected_genes
chr22	21347057	21347218	LZTR1	693_selected_genes
chr22	21347925	21348068	LZTR1	693_selected_genes
chr22	21348187	21348333	LZTR1	693_selected_genes
chr22	21348367	21348583	LZTR1	693_selected_genes
chr22	21348821	21349041	LZTR1	693_selected_genes
chr22	21349133	21349340	LZTR1	693_selected_genes
chr22	21349631	21349791	LZTR1	693_selected_genes
chr22	21350009	21350186	LZTR1	693_selected_genes
chr22	21350226	21350426	LZTR1	693_selected_genes
chr22	21350959	21351115	LZTR1	693_selected_genes
chr22	21351149	21351451	LZTR1	693_selected_genes
chr22	21351495	21351662	LZTR1	693_selected_genes
chr11	95712086	95713152	MAML2	693_selected_genes
chr11	95718669	95718831	MAML2	693_selected_genes
chr11	95724658	95724912	MAML2	693_selected_genes
chr11	95825030	95826706	MAML2	693_selected_genes
chr11	96074521	96075084	MAML2	693_selected_genes
chr15	66679660	66679790	MAP2K1	693_selected_genes
chr15	66727339	66727600	MAP2K1	693_selected_genes
chr15	66729058	66729255	MAP2K1	693_selected_genes
chr15	66735592	66735720	MAP2K1	693_selected_genes
chr15	66736968	66737070	MAP2K1	693_selected_genes
chr15	66745816	66745906	MAP2K1	693_selected_genes
chr15	66774067	66774242	MAP2K1	693_selected_genes
chr15	66777302	66777554	MAP2K1	693_selected_genes
chr15	66779540	66779655	MAP2K1	693_selected_genes
chr15	66781527	66781639	MAP2K1	693_selected_genes
chr15	66782030	66782126	MAP2K1	693_selected_genes
chr15	66782814	66782978	MAP2K1	693_selected_genes
chr19	4090570	4090731	MAP2K2	693_selected_genes
chr19	4094425	4094521	MAP2K2	693_selected_genes
chr19	4095360	4095472	MAP2K2	693_selected_genes
chr19	4097251	4097366	MAP2K2	693_selected_genes
chr19	4099173	4099437	MAP2K2	693_selected_genes
chr19	4100991	4101166	MAP2K2	693_selected_genes
chr19	4101201	4101303	MAP2K2	693_selected_genes
chr19	4102348	4102476	MAP2K2	693_selected_genes
chr19	4110481	4110678	MAP2K2	693_selected_genes
chr19	4117391	4117652	MAP2K2	693_selected_genes
chr19	4123755	4123897	MAP2K2	693_selected_genes
chr17	11924178	11924343	MAP2K4	693_selected_genes
chr17	11935557	11935640	MAP2K4	693_selected_genes
chr17	11958180	11958333	MAP2K4	693_selected_genes
chr17	11984647	11984872	MAP2K4	693_selected_genes
chr17	11998866	11999036	MAP2K4	693_selected_genes
chr17	12011081	12011251	MAP2K4	693_selected_genes
chr17	12013666	12013768	MAP2K4	693_selected_genes
chr17	12016524	12016702	MAP2K4	693_selected_genes
chr17	12028585	12028713	MAP2K4	693_selected_genes
chr17	12032430	12032629	MAP2K4	693_selected_genes
chr17	12043130	12043226	MAP2K4	693_selected_genes
chr17	12044438	12044602	MAP2K4	693_selected_genes
chr19	7968804	7968978	MAP2K7	693_selected_genes
chr19	7970667	7970765	MAP2K7	693_selected_genes
chr19	7974614	7974806	MAP2K7	693_selected_genes
chr19	7974922	7975283	MAP2K7	693_selected_genes
chr19	7975312	7975482	MAP2K7	693_selected_genes
chr19	7975555	7975713	MAP2K7	693_selected_genes
chr19	7975839	7976069	MAP2K7	693_selected_genes
chr19	7976109	7976240	MAP2K7	693_selected_genes

chr19	7976274	7976488	MAP2K7	693_selected_genes
chr19	7977008	7977104	MAP2K7	693_selected_genes
chr19	7977156	7977341	MAP2K7	693_selected_genes
chr5	56111375	56111907	MAP3K1	693_selected_genes
chr5	56152401	56152602	MAP3K1	693_selected_genes
chr5	56155516	56155767	MAP3K1	693_selected_genes
chr5	56160535	56160786	MAP3K1	693_selected_genes
chr5	56161141	56161308	MAP3K1	693_selected_genes
chr5	56161630	56161829	MAP3K1	693_selected_genes
chr5	56167711	56167883	MAP3K1	693_selected_genes
chr5	56168442	56168574	MAP3K1	693_selected_genes
chr5	56168626	56168857	MAP3K1	693_selected_genes
chr5	56170833	56171162	MAP3K1	693_selected_genes
chr5	56174781	56174953	MAP3K1	693_selected_genes
chr5	56176512	56176654	MAP3K1	693_selected_genes
chr5	56176884	56177124	MAP3K1	693_selected_genes
chr5	56177371	56178718	MAP3K1	693_selected_genes
chr5	56179328	56179531	MAP3K1	693_selected_genes
chr5	56180465	56180678	MAP3K1	693_selected_genes
chr5	56181733	56181915	MAP3K1	693_selected_genes
chr5	56183179	56183372	MAP3K1	693_selected_genes
chr5	56184027	56184209	MAP3K1	693_selected_genes
chr5	56189332	56189532	MAP3K1	693_selected_genes
chr3	185003368	185003456	MAP3K13	693_selected_genes
chr3	185009375	185009510	MAP3K13	693_selected_genes
chr3	185046969	185047062	MAP3K13	693_selected_genes
chr3	185146344	185146869	MAP3K13	693_selected_genes
chr3	185155209	185155443	MAP3K13	693_selected_genes
chr3	185161207	185161449	MAP3K13	693_selected_genes
chr3	185165551	185165760	MAP3K13	693_selected_genes
chr3	185167662	185167871	MAP3K13	693_selected_genes
chr3	185169049	185169208	MAP3K13	693_selected_genes
chr3	185181312	185181472	MAP3K13	693_selected_genes
chr3	185183509	185183676	MAP3K13	693_selected_genes
chr3	185184588	185184776	MAP3K13	693_selected_genes
chr3	185190737	185191574	MAP3K13	693_selected_genes
chr3	185195088	185195209	MAP3K13	693_selected_genes
chr3	185197994	185198342	MAP3K13	693_selected_genes
chr3	185200117	185200269	MAP3K13	693_selected_genes
chr17	61700094	61700148	MAP3K3	693_selected_genes
chr17	61710015	61710187	MAP3K3	693_selected_genes
chr17	61712043	61712186	MAP3K3	693_selected_genes
chr17	61723368	61723459	MAP3K3	693_selected_genes
chr17	61729891	61730041	MAP3K3	693_selected_genes
chr17	61735128	61735292	MAP3K3	693_selected_genes
chr17	61744274	61744445	MAP3K3	693_selected_genes
chr17	61745480	61745550	MAP3K3	693_selected_genes
chr17	61759100	61759284	MAP3K3	693_selected_genes
chr17	61762851	61762975	MAP3K3	693_selected_genes
chr17	61765861	61765979	MAP3K3	693_selected_genes
chr17	61766123	61766266	MAP3K3	693_selected_genes
chr17	61766878	61767120	MAP3K3	693_selected_genes
chr17	61767598	61767797	MAP3K3	693_selected_genes
chr17	61768327	61768422	MAP3K3	693_selected_genes
chr17	61768436	61768618	MAP3K3	693_selected_genes
chr17	61769067	61769247	MAP3K3	693_selected_genes
chr17	61769576	61769804	MAP3K3	693_selected_genes
chr17	61770883	61771162	MAP3K3	693_selected_genes
chr6	161412938	161413140	MAP3K4	693_selected_genes
chr6	161455265	161455506	MAP3K4	693_selected_genes
chr6	161469252	161469346	MAP3K4	693_selected_genes
chr6	161469622	161471036	MAP3K4	693_selected_genes
chr6	161488205	161488353	MAP3K4	693_selected_genes
chr6	161491614	161491907	MAP3K4	693_selected_genes
chr6	161494472	161494669	MAP3K4	693_selected_genes
chr6	161501887	161502095	MAP3K4	693_selected_genes
chr6	161505507	161505674	MAP3K4	693_selected_genes
chr6	161507385	161507535	MAP3K4	693_selected_genes
chr6	161507590	161507724	MAP3K4	693_selected_genes
chr6	161508694	161509011	MAP3K4	693_selected_genes
chr6	161510328	161510528	MAP3K4	693_selected_genes
chr6	161512385	161512597	MAP3K4	693_selected_genes
chr6	161513016	161513200	MAP3K4	693_selected_genes
chr6	161513984	161514113	MAP3K4	693_selected_genes
chr6	161514779	161514908	MAP3K4	693_selected_genes
chr6	161518086	161518233	MAP3K4	693_selected_genes
chr6	161519284	161519484	MAP3K4	693_selected_genes
chr6	161522898	161523049	MAP3K4	693_selected_genes
chr6	161523705	161523836	MAP3K4	693_selected_genes
chr6	161527520	161527762	MAP3K4	693_selected_genes
chr6	161528905	161529026	MAP3K4	693_selected_genes
chr6	161529749	161529916	MAP3K4	693_selected_genes
chr6	161529957	161530098	MAP3K4	693_selected_genes
chr6	161530761	161530971	MAP3K4	693_selected_genes

chr6	161532842	161533015	MAP3K4	693_selected_genes
chr6	161533674	161533831	MAP3K4	693_selected_genes
chr6	161536129	161536359	MAP3K4	693_selected_genes
chr6	161537856	161537927	MAP3K4	693_selected_genes
chr19	39078362	39078482	MAP4K1	693_selected_genes
chr19	39079792	39079940	MAP4K1	693_selected_genes
chr19	39083896	39084002	MAP4K1	693_selected_genes
chr19	39086099	39086220	MAP4K1	693_selected_genes
chr19	39086254	39086394	MAP4K1	693_selected_genes
chr19	39086553	39086666	MAP4K1	693_selected_genes
chr19	39086926	39087151	MAP4K1	693_selected_genes
chr19	39087648	39087802	MAP4K1	693_selected_genes
chr19	39087940	39088049	MAP4K1	693_selected_genes
chr19	39088083	39088259	MAP4K1	693_selected_genes
chr19	39090539	39090650	MAP4K1	693_selected_genes
chr19	39090691	39090818	MAP4K1	693_selected_genes
chr19	39092055	39092190	MAP4K1	693_selected_genes
chr19	39096023	39096156	MAP4K1	693_selected_genes
chr19	39096182	39096395	MAP4K1	693_selected_genes
chr19	39096787	39096880	MAP4K1	693_selected_genes
chr19	39098478	39098698	MAP4K1	693_selected_genes
chr19	39098726	39098835	MAP4K1	693_selected_genes
chr19	39100210	39100339	MAP4K1	693_selected_genes
chr19	39100523	39100690	MAP4K1	693_selected_genes
chr19	39101665	39101797	MAP4K1	693_selected_genes
chr19	39101857	39101970	MAP4K1	693_selected_genes
chr19	39103225	39103407	MAP4K1	693_selected_genes
chr19	39104494	39104617	MAP4K1	693_selected_genes
chr19	39104657	39104750	MAP4K1	693_selected_genes
chr19	39104859	39104957	MAP4K1	693_selected_genes
chr19	39105004	39105110	MAP4K1	693_selected_genes
chr19	39106809	39106924	MAP4K1	693_selected_genes
chr19	39107968	39108109	MAP4K1	693_selected_genes
chr19	39108182	39108290	MAP4K1	693_selected_genes
chr19	39108411	39108560	MAP4K1	693_selected_genes
chr19	39109379	39109449	MAP4K1	693_selected_genes
chr2	39477733	39477871	MAP4K3	693_selected_genes
chr2	39478945	39479051	MAP4K3	693_selected_genes
chr2	39481565	39481686	MAP4K3	693_selected_genes
chr2	39485463	39485606	MAP4K3	693_selected_genes
chr2	39485634	39485753	MAP4K3	693_selected_genes
chr2	39487721	39487943	MAP4K3	693_selected_genes
chr2	39492318	39492472	MAP4K3	693_selected_genes
chr2	39494304	39494413	MAP4K3	693_selected_genes
chr2	39499398	39499566	MAP4K3	693_selected_genes
chr2	39499597	39499708	MAP4K3	693_selected_genes
chr2	39505522	39505652	MAP4K3	693_selected_genes
chr2	39507387	39507522	MAP4K3	693_selected_genes
chr2	39509628	39509720	MAP4K3	693_selected_genes
chr2	39513967	39514130	MAP4K3	693_selected_genes
chr2	39515236	39515446	MAP4K3	693_selected_genes
chr2	39517407	39517500	MAP4K3	693_selected_genes
chr2	39519888	39519992	MAP4K3	693_selected_genes
chr2	39520345	39520434	MAP4K3	693_selected_genes
chr2	39526858	39526967	MAP4K3	693_selected_genes
chr2	39530230	39530283	MAP4K3	693_selected_genes
chr2	39535058	39535171	MAP4K3	693_selected_genes
chr2	39536576	39536685	MAP4K3	693_selected_genes
chr2	39542425	39542554	MAP4K3	693_selected_genes
chr2	39552633	39552794	MAP4K3	693_selected_genes
chr2	39552845	39552977	MAP4K3	693_selected_genes
chr2	39553014	39553127	MAP4K3	693_selected_genes
chr2	39553261	39553443	MAP4K3	693_selected_genes
chr2	39559032	39559155	MAP4K3	693_selected_genes
chr2	39560647	39560740	MAP4K3	693_selected_genes
chr2	39564035	39564133	MAP4K3	693_selected_genes
chr2	39564641	39564747	MAP4K3	693_selected_genes
chr2	39570503	39570618	MAP4K3	693_selected_genes
chr2	39583330	39583505	MAP4K3	693_selected_genes
chr2	39605181	39605289	MAP4K3	693_selected_genes
chr2	39606867	39606975	MAP4K3	693_selected_genes
chr2	39664007	39664153	MAP4K3	693_selected_genes
chr22	22123467	22123634	MAPK1	693_selected_genes
chr22	22127136	22127296	MAPK1	693_selected_genes
chr22	22142520	22142702	MAPK1	693_selected_genes
chr22	22142957	22143122	MAPK1	693_selected_genes
chr22	22153275	22153442	MAPK1	693_selected_genes
chr22	22160113	22160353	MAPK1	693_selected_genes
chr22	22161927	22162160	MAPK1	693_selected_genes
chr22	22221586	22221755	MAPK1	693_selected_genes
chr11	45907346	45907497	MAPK8IP1	693_selected_genes
chr11	45918405	45918526	MAPK8IP1	693_selected_genes
chr11	45919610	45919766	MAPK8IP1	693_selected_genes
chr11	45921663	45922028	MAPK8IP1	693_selected_genes

chr11	45923505	45923637	MAPK8IP1	693_selected_genes
chr11	45923897	45924760	MAPK8IP1	693_selected_genes
chr11	45924890	45925016	MAPK8IP1	693_selected_genes
chr11	45925514	45925737	MAPK8IP1	693_selected_genes
chr11	45925980	45926140	MAPK8IP1	693_selected_genes
chr11	45926243	45926410	MAPK8IP1	693_selected_genes
chr11	45926496	45926617	MAPK8IP1	693_selected_genes
chr11	45926676	45926825	MAPK8IP1	693_selected_genes
chr11	45927174	45927297	MAPK8IP1	693_selected_genes
chr14	65472896	65473027	MAX	693_selected_genes
chr14	65543143	65543406	MAX	693_selected_genes
chr14	65544036	65544171	MAX	693_selected_genes
chr14	65544255	65544386	MAX	693_selected_genes
chr14	65544495	65544779	MAX	693_selected_genes
chr14	65550872	65551042	MAX	693_selected_genes
chr14	65560400	65560558	MAX	693_selected_genes
chr14	65568238	65568315	MAX	693_selected_genes
chr14	65568996	65569082	MAX	693_selected_genes
chr18	47793932	47794058	MBD1	693_selected_genes
chr18	47795973	47796213	MBD1	693_selected_genes
chr18	47796355	47796480	MBD1	693_selected_genes
chr18	47797845	47797935	MBD1	693_selected_genes
chr18	47799021	47799133	MBD1	693_selected_genes
chr18	47799168	47799350	MBD1	693_selected_genes
chr18	47799678	47799866	MBD1	693_selected_genes
chr18	47799908	47800258	MBD1	693_selected_genes
chr18	47800530	47800748	MBD1	693_selected_genes
chr18	47801321	47801440	MBD1	693_selected_genes
chr18	47801473	47801640	MBD1	693_selected_genes
chr18	47801714	47801839	MBD1	693_selected_genes
chr18	47801944	47802123	MBD1	693_selected_genes
chr18	47802179	47802376	MBD1	693_selected_genes
chr18	47802722	47802813	MBD1	693_selected_genes
chr18	47803006	47803139	MBD1	693_selected_genes
chr18	47803176	47803393	MBD1	693_selected_genes
chr18	47803434	47803599	MBD1	693_selected_genes
chr18	47806227	47806412	MBD1	693_selected_genes
chr18	47807508	47807611	MBD1	693_selected_genes
chr16	89985641	89986645	MC1R	693_selected_genes
chr16	89986972	89987221	MC1R	693_selected_genes
chr1	150549814	150549992	MCL1	693_selected_genes
chr1	150550694	150550992	MCL1	693_selected_genes
chr1	150551293	150552031	MCL1	693_selected_genes
chr6	30668216	30668434	MDC1	693_selected_genes
chr6	30670304	30670465	MDC1	693_selected_genes
chr6	30670503	30670679	MDC1	693_selected_genes
chr6	30670855	30671088	MDC1	693_selected_genes
chr6	30671169	30671339	MDC1	693_selected_genes
chr6	30671372	30673900	MDC1	693_selected_genes
chr6	30675135	30675256	MDC1	693_selected_genes
chr6	30675317	30676159	MDC1	693_selected_genes
chr6	30679163	30679306	MDC1	693_selected_genes
chr6	30679418	30679528	MDC1	693_selected_genes
chr6	30679625	30681156	MDC1	693_selected_genes
chr6	30681399	30681519	MDC1	693_selected_genes
chr6	30681554	30681985	MDC1	693_selected_genes
chr6	30682791	30682977	MDC1	693_selected_genes
chr12	69202232	69202296	MDM2	693_selected_genes
chr12	69202962	69203103	MDM2	693_selected_genes
chr12	69207308	69207433	MDM2	693_selected_genes
chr12	69208357	69208464	MDM2	693_selected_genes
chr12	69209382	69209525	MDM2	693_selected_genes
chr12	69210566	69210750	MDM2	693_selected_genes
chr12	69214079	69214179	MDM2	693_selected_genes
chr12	69218117	69218235	MDM2	693_selected_genes
chr12	69218309	69218456	MDM2	693_selected_genes
chr12	69222525	69222736	MDM2	693_selected_genes
chr12	69229583	69229789	MDM2	693_selected_genes
chr12	69230426	69230554	MDM2	693_selected_genes
chr12	69233028	69233654	MDM2	693_selected_genes
chr1	204494621	204494749	MDM4	693_selected_genes
chr1	204495462	204495587	MDM4	693_selected_genes
chr1	204499786	204499970	MDM4	693_selected_genes
chr1	204501293	204501399	MDM4	693_selected_genes
chr1	204501871	204501972	MDM4	693_selected_genes
chr1	204506020	204506072	MDM4	693_selected_genes
chr1	204506532	204506650	MDM4	693_selected_genes
chr1	204507016	204507144	MDM4	693_selected_genes
chr1	204507311	204507461	MDM4	693_selected_genes
chr1	204511886	204512097	MDM4	693_selected_genes
chr1	204513637	204513837	MDM4	693_selected_genes
chr1	204515899	204516030	MDM4	693_selected_genes
chr1	204518215	204518835	MDM4	693_selected_genes
chr1	204527743	204527834	MDM4	693_selected_genes

chr3	168802671	168802856	MECOM	693_selected_genes
chr3	168804310	168804378	MECOM	693_selected_genes
chr3	168806762	168806996	MECOM	693_selected_genes
chr3	168807762	168808049	MECOM	693_selected_genes
chr3	168810720	168810915	MECOM	693_selected_genes
chr3	168812838	168813058	MECOM	693_selected_genes
chr3	168818647	168818775	MECOM	693_selected_genes
chr3	168819822	168820039	MECOM	693_selected_genes
chr3	168825688	168825765	MECOM	693_selected_genes
chr3	168830549	168830687	MECOM	693_selected_genes
chr3	168833145	168834552	MECOM	693_selected_genes
chr3	168838818	168839025	MECOM	693_selected_genes
chr3	168840342	168840540	MECOM	693_selected_genes
chr3	168845606	168845873	MECOM	693_selected_genes
chr3	168849191	168849344	MECOM	693_selected_genes
chr3	168861460	168861645	MECOM	693_selected_genes
chr3	168862759	168862812	MECOM	693_selected_genes
chr3	169098949	169099337	MECOM	693_selected_genes
chr3	169283731	169283853	MECOM	693_selected_genes
chr3	169381098	169381185	MECOM	693_selected_genes
chr17	37561505	37561586	MED1	693_selected_genes
chr17	37563702	37566999	MED1	693_selected_genes
chr17	37571253	37571409	MED1	693_selected_genes
chr17	37571471	37571617	MED1	693_selected_genes
chr17	37575944	37576196	MED1	693_selected_genes
chr17	37579554	37579723	MED1	693_selected_genes
chr17	37579924	37580099	MED1	693_selected_genes
chr17	37580854	37581016	MED1	693_selected_genes
chr17	37583928	37584068	MED1	693_selected_genes
chr17	37587342	37587466	MED1	693_selected_genes
chr17	37588169	37588294	MED1	693_selected_genes
chr17	37590476	37590598	MED1	693_selected_genes
chr17	37595392	37595471	MED1	693_selected_genes
chr17	37596613	37596796	MED1	693_selected_genes
chr17	37596850	37596955	MED1	693_selected_genes
chr17	37599777	37599906	MED1	693_selected_genes
chr17	37601689	37601847	MED1	693_selected_genes
chr17	37604025	37604182	MED1	693_selected_genes
chr17	37607265	37607340	MED1	693_selected_genes
chrX	70338579	70338728	MED12	693_selected_genes
chrX	70339163	70339352	MED12	693_selected_genes
chrX	70339510	70339752	MED12	693_selected_genes
chrX	70339838	70340045	MED12	693_selected_genes
chrX	70340795	70341027	MED12	693_selected_genes
chrX	70341151	70341312	MED12	693_selected_genes
chrX	70341386	70341691	MED12	693_selected_genes
chrX	70342024	70342221	MED12	693_selected_genes
chrX	70342332	70342482	MED12	693_selected_genes
chrX	70342562	70342749	MED12	693_selected_genes
chrX	70342919	70343101	MED12	693_selected_genes
chrX	70343418	70343595	MED12	693_selected_genes
chrX	70343983	70344263	MED12	693_selected_genes
chrX	70344588	70344719	MED12	693_selected_genes
chrX	70344800	70345021	MED12	693_selected_genes
chrX	70345175	70345370	MED12	693_selected_genes
chrX	70345487	70345588	MED12	693_selected_genes
chrX	70345860	70346029	MED12	693_selected_genes
chrX	70346165	70346359	MED12	693_selected_genes
chrX	70346793	70347007	MED12	693_selected_genes
chrX	70347160	70347342	MED12	693_selected_genes
chrX	70347717	70347995	MED12	693_selected_genes
chrX	70348120	70348315	MED12	693_selected_genes
chrX	70348422	70348593	MED12	693_selected_genes
chrX	70348938	70349090	MED12	693_selected_genes
chrX	70349140	70349304	MED12	693_selected_genes
chrX	70349504	70349730	MED12	693_selected_genes
chrX	70349859	70350089	MED12	693_selected_genes
chrX	70351374	70351496	MED12	693_selected_genes
chrX	70351897	70352081	MED12	693_selected_genes
chrX	70352201	70352413	MED12	693_selected_genes
chrX	70352669	70352831	MED12	693_selected_genes
chrX	70352947	70353087	MED12	693_selected_genes
chrX	70354181	70354341	MED12	693_selected_genes
chrX	70354537	70354723	MED12	693_selected_genes
chrX	70354916	70355128	MED12	693_selected_genes
chrX	70356105	70356530	MED12	693_selected_genes
chrX	70356703	70356904	MED12	693_selected_genes
chrX	70357011	70357267	MED12	693_selected_genes
chrX	70357382	70357510	MED12	693_selected_genes
chrX	70357529	70357818	MED12	693_selected_genes
chrX	70360459	70360732	MED12	693_selected_genes
chrX	70361054	70361245	MED12	693_selected_genes
chrX	70361707	70361839	MED12	693_selected_genes
chrX	70361999	70362093	MED12	693_selected_genes

chr11	93517654	93517954	MED17	693_selected_genes
chr11	93521141	93521379	MED17	693_selected_genes
chr11	93523400	93523534	MED17	693_selected_genes
chr11	93523714	93523984	MED17	693_selected_genes
chr11	93526868	93527082	MED17	693_selected_genes
chr11	93527091	93527226	MED17	693_selected_genes
chr11	93528048	93528251	MED17	693_selected_genes
chr11	93529550	93529731	MED17	693_selected_genes
chr11	93530675	93530910	MED17	693_selected_genes
chr11	93534975	93535163	MED17	693_selected_genes
chr11	93540658	93540826	MED17	693_selected_genes
chr11	93542857	93543067	MED17	693_selected_genes
chr11	93544993	93545255	MED17	693_selected_genes
chr6	131895407	131895460	MED23	693_selected_genes
chr6	131908034	131908102	MED23	693_selected_genes
chr6	131908793	131909011	MED23	693_selected_genes
chr6	131910575	131910761	MED23	693_selected_genes
chr6	131911436	131911607	MED23	693_selected_genes
chr6	131912427	131912692	MED23	693_selected_genes
chr6	131913502	131913625	MED23	693_selected_genes
chr6	131914120	131914336	MED23	693_selected_genes
chr6	131915213	131915500	MED23	693_selected_genes
chr6	131917061	131917328	MED23	693_selected_genes
chr6	131917632	131917853	MED23	693_selected_genes
chr6	131919365	131919632	MED23	693_selected_genes
chr6	131919670	131919926	MED23	693_selected_genes
chr6	131921152	131921327	MED23	693_selected_genes
chr6	131923332	131923546	MED23	693_selected_genes
chr6	131924144	131924369	MED23	693_selected_genes
chr6	131925292	131925485	MED23	693_selected_genes
chr6	131926354	131926650	MED23	693_selected_genes
chr6	131927593	131927789	MED23	693_selected_genes
chr6	131929042	131929236	MED23	693_selected_genes
chr6	131931160	131931429	MED23	693_selected_genes
chr6	131936438	131936506	MED23	693_selected_genes
chr6	131936814	131936877	MED23	693_selected_genes
chr6	131937016	131937167	MED23	693_selected_genes
chr6	131939521	131939684	MED23	693_selected_genes
chr6	131940941	131941061	MED23	693_selected_genes
chr6	131941742	131941894	MED23	693_selected_genes
chr6	131942995	131943144	MED23	693_selected_genes
chr6	131944465	131944627	MED23	693_selected_genes
chr6	131945979	131946154	MED23	693_selected_genes
chr6	131948510	131948648	MED23	693_selected_genes
chr6	131948755	131948837	MED23	693_selected_genes
chr6	131949125	131949214	MED23	693_selected_genes
chr15	100173299	100173403	MEF2A	693_selected_genes
chr15	100185740	100185994	MEF2A	693_selected_genes
chr15	100211502	100211684	MEF2A	693_selected_genes
chr15	100211699	100211887	MEF2A	693_selected_genes
chr15	100214572	100214842	MEF2A	693_selected_genes
chr15	100215578	100215688	MEF2A	693_selected_genes
chr15	100230420	100230658	MEF2A	693_selected_genes
chr15	100230745	100230810	MEF2A	693_selected_genes
chr15	100230824	100230886	MEF2A	693_selected_genes
chr15	100243541	100243615	MEF2A	693_selected_genes
chr15	100246908	100247085	MEF2A	693_selected_genes
chr15	100250813	100250990	MEF2A	693_selected_genes
chr15	100252569	100253001	MEF2A	693_selected_genes
chr19	19256477	19256856	MEF2B	693_selected_genes
chr19	19257052	19257239	MEF2B	693_selected_genes
chr19	19257338	19257482	MEF2B	693_selected_genes
chr19	19257525	19257709	MEF2B	693_selected_genes
chr19	19257819	19258017	MEF2B	693_selected_genes
chr19	19258481	19258666	MEF2B	693_selected_genes
chr19	19260000	19260263	MEF2B	693_selected_genes
chr19	19261465	19261598	MEF2B	693_selected_genes
chr19	19267808	19267970	MEF2B	693_selected_genes
chr11	64571780	64572313	MEN1	693_selected_genes
chr11	64572480	64572695	MEN1	693_selected_genes
chr11	64573081	64573267	MEN1	693_selected_genes
chr11	64573678	64573865	MEN1	693_selected_genes
chr11	64574457	64574595	MEN1	693_selected_genes
chr11	64574625	64574716	MEN1	693_selected_genes
chr11	64574998	64575177	MEN1	693_selected_genes
chr11	64575337	64575596	MEN1	693_selected_genes
chr11	64577096	64577606	MEN1	693_selected_genes
chr7	116335785	116335878	MET	693_selected_genes
chr7	116339099	116340363	MET	693_selected_genes
chr7	116371696	116371938	MET	693_selected_genes
chr7	116379978	116380163	MET	693_selected_genes
chr7	116380880	116381104	MET	693_selected_genes
chr7	116395383	116395594	MET	693_selected_genes
chr7	116397465	116397618	MET	693_selected_genes

chr7	116397666	116397853	MET	693_selected_genes
chr7	116398487	116398699	MET	693_selected_genes
chr7	116399365	116399569	MET	693_selected_genes
chr7	116403078	116403347	MET	693_selected_genes
chr7	116409673	116409945	MET	693_selected_genes
chr7	116411526	116411733	MET	693_selected_genes
chr7	116411877	116412068	MET	693_selected_genes
chr7	116414909	116415190	MET	693_selected_genes
chr7	116417417	116417548	MET	693_selected_genes
chr7	116418804	116419036	MET	693_selected_genes
chr7	116422016	116422176	MET	693_selected_genes
chr7	116423332	116423548	MET	693_selected_genes
chr7	116435683	116435870	MET	693_selected_genes
chr7	116435915	116436203	MET	693_selected_genes
chr15	41961067	41962181	MGA	693_selected_genes
chr15	41976566	41976632	MGA	693_selected_genes
chr15	41988247	41989246	MGA	693_selected_genes
chr15	41991035	41991164	MGA	693_selected_genes
chr15	41991236	41991382	MGA	693_selected_genes
chr15	41999900	42000082	MGA	693_selected_genes
chr15	42000276	42000431	MGA	693_selected_genes
chr15	42002863	42003572	MGA	693_selected_genes
chr15	42005323	42005719	MGA	693_selected_genes
chr15	42019352	42019629	MGA	693_selected_genes
chr15	42021336	42021572	MGA	693_selected_genes
chr15	42026694	42026817	MGA	693_selected_genes
chr15	42028353	42028921	MGA	693_selected_genes
chr15	42032078	42032426	MGA	693_selected_genes
chr15	42034718	42035395	MGA	693_selected_genes
chr15	42040809	42041150	MGA	693_selected_genes
chr15	42041283	42042838	MGA	693_selected_genes
chr15	42046609	42046790	MGA	693_selected_genes
chr15	42049960	42050062	MGA	693_selected_genes
chr15	42052495	42052752	MGA	693_selected_genes
chr15	42053911	42054073	MGA	693_selected_genes
chr15	42054301	42054585	MGA	693_selected_genes
chr15	42057058	42057285	MGA	693_selected_genes
chr15	42058176	42059503	MGA	693_selected_genes
chr3	69788723	69788877	MITF	693_selected_genes
chr3	69812967	69813118	MITF	693_selected_genes
chr3	69915416	69915522	MITF	693_selected_genes
chr3	69928230	69928559	MITF	693_selected_genes
chr3	69985848	69985931	MITF	693_selected_genes
chr3	69986947	69987225	MITF	693_selected_genes
chr3	69987477	69987542	MITF	693_selected_genes
chr3	69988223	69988357	MITF	693_selected_genes
chr3	69990361	69990507	MITF	693_selected_genes
chr3	69998176	69998344	MITF	693_selected_genes
chr3	70000937	70001062	MITF	693_selected_genes
chr3	70005580	70005706	MITF	693_selected_genes
chr3	70008398	70008596	MITF	693_selected_genes
chr3	70013972	70014424	MITF	693_selected_genes
chr3	37035013	37035210	MLH1	693_selected_genes
chr3	37038084	37038225	MLH1	693_selected_genes
chr3	37042420	37042569	MLH1	693_selected_genes
chr3	37045866	37045990	MLH1	693_selected_genes
chr3	37048456	37048579	MLH1	693_selected_genes
chr3	37050279	37050421	MLH1	693_selected_genes
chr3	37053285	37053378	MLH1	693_selected_genes
chr3	37053476	37053615	MLH1	693_selected_genes
chr3	37055897	37056060	MLH1	693_selected_genes
chr3	37058971	37059115	MLH1	693_selected_genes
chr3	37061775	37061979	MLH1	693_selected_genes
chr3	37067102	37067523	MLH1	693_selected_genes
chr3	37070249	37070448	MLH1	693_selected_genes
chr3	37081651	37081810	MLH1	693_selected_genes
chr3	37083733	37083847	MLH1	693_selected_genes
chr3	37088984	37089199	MLH1	693_selected_genes
chr3	37089982	37090125	MLH1	693_selected_genes
chr3	37090369	37090533	MLH1	693_selected_genes
chr3	37091951	37092169	MLH1	693_selected_genes
chr3	37107082	37107135	MLH1	693_selected_genes
chr14	75483759	75483929	MLH3	693_selected_genes
chr14	75485506	75485708	MLH3	693_selected_genes
chr14	75489491	75489620	MLH3	693_selected_genes
chr14	75489672	75489746	MLH3	693_selected_genes
chr14	75497220	75497443	MLH3	693_selected_genes
chr14	75498745	75498907	MLH3	693_selected_genes
chr14	75500096	75500218	MLH3	693_selected_genes
chr14	75503346	75503470	MLH3	693_selected_genes
chr14	75505017	75505140	MLH3	693_selected_genes
chr14	75506588	75506743	MLH3	693_selected_genes
chr14	75508292	75508428	MLH3	693_selected_genes
chr14	75509056	75509205	MLH3	693_selected_genes

chr14	75513053	75516383	MLH3	693_selected_genes
chr10	21823548	21823758	MLLT10	693_selected_genes
chr10	21827736	21827866	MLLT10	693_selected_genes
chr10	21840719	21840875	MLLT10	693_selected_genes
chr10	21845546	21845896	MLLT10	693_selected_genes
chr10	21875197	21875302	MLLT10	693_selected_genes
chr10	21884234	21884394	MLLT10	693_selected_genes
chr10	21901251	21901405	MLLT10	693_selected_genes
chr10	21903734	21903878	MLLT10	693_selected_genes
chr10	21906015	21906161	MLLT10	693_selected_genes
chr10	21940576	21940722	MLLT10	693_selected_genes
chr10	21959352	21959658	MLLT10	693_selected_genes
chr10	21962253	21962873	MLLT10	693_selected_genes
chr10	21970235	21970330	MLLT10	693_selected_genes
chr10	21971128	21971211	MLLT10	693_selected_genes
chr10	21977394	21977492	MLLT10	693_selected_genes
chr10	22002675	22002904	MLLT10	693_selected_genes
chr10	22015147	22015309	MLLT10	693_selected_genes
chr10	22016759	22016882	MLLT10	693_selected_genes
chr10	22019803	22020008	MLLT10	693_selected_genes
chr10	22021802	22022041	MLLT10	693_selected_genes
chr10	22022407	22022546	MLLT10	693_selected_genes
chr10	22022671	22023083	MLLT10	693_selected_genes
chr10	22024042	22024189	MLLT10	693_selected_genes
chr10	22028933	22029190	MLLT10	693_selected_genes
chr10	22030842	22030937	MLLT10	693_selected_genes
chr9	20346415	20346597	MLLT3	693_selected_genes
chr9	20353497	20353619	MLLT3	693_selected_genes
chr9	20354780	20354902	MLLT3	693_selected_genes
chr9	20360714	20360864	MLLT3	693_selected_genes
chr9	20363448	20363628	MLLT3	693_selected_genes
chr9	20365641	20365767	MLLT3	693_selected_genes
chr9	20413693	20414448	MLLT3	693_selected_genes
chr9	20448095	20448289	MLLT3	693_selected_genes
chr9	20456676	20456809	MLLT3	693_selected_genes
chr9	20620627	20620858	MLLT3	693_selected_genes
chr9	20621746	20621799	MLLT3	693_selected_genes
chr9	20622218	20622280	MLLT3	693_selected_genes
chr16	55513366	55513569	MMP2	693_selected_genes
chr16	55515762	55515815	MMP2	693_selected_genes
chr16	55516795	55517072	MMP2	693_selected_genes
chr16	55517902	55518101	MMP2	693_selected_genes
chr16	55519185	55519364	MMP2	693_selected_genes
chr16	55519490	55519714	MMP2	693_selected_genes
chr16	55522429	55522653	MMP2	693_selected_genes
chr16	55523537	55523761	MMP2	693_selected_genes
chr16	55525687	55525893	MMP2	693_selected_genes
chr16	55527044	55527230	MMP2	693_selected_genes
chr16	55530812	55530999	MMP2	693_selected_genes
chr16	55532175	55532385	MMP2	693_selected_genes
chr16	55536665	55536826	MMP2	693_selected_genes
chr16	55539225	55539379	MMP2	693_selected_genes
chr22	28146877	28147109	MN1	693_selected_genes
chr22	28192725	28196556	MN1	693_selected_genes
chr1	158811918	158812233	MNDA	693_selected_genes
chr1	158813043	158813230	MNDA	693_selected_genes
chr1	158813719	158813937	MNDA	693_selected_genes
chr1	158815351	158815818	MNDA	693_selected_genes
chr1	158817492	158817731	MNDA	693_selected_genes
chr1	158818954	158819052	MNDA	693_selected_genes
chr1	43803494	43803623	MPL	693_selected_genes
chr1	43803744	43803927	MPL	693_selected_genes
chr1	43804187	43804416	MPL	693_selected_genes
chr1	43804916	43805265	MPL	693_selected_genes
chr1	43805609	43805822	MPL	693_selected_genes
chr1	43806032	43806209	MPL	693_selected_genes
chr1	43812090	43812325	MPL	693_selected_genes
chr1	43812437	43812630	MPL	693_selected_genes
chr1	43814488	43814698	MPL	693_selected_genes
chr1	43814908	43815140	MPL	693_selected_genes
chr1	43817861	43817999	MPL	693_selected_genes
chr1	43818163	43818468	MPL	693_selected_genes
chr17	56347991	56348249	MPO	693_selected_genes
chr17	56348990	56349278	MPO	693_selected_genes
chr17	56350083	56350304	MPO	693_selected_genes
chr17	56350749	56351055	MPO	693_selected_genes
chr17	56352877	56353088	MPO	693_selected_genes
chr17	56355162	56355531	MPO	693_selected_genes
chr17	56356343	56356600	MPO	693_selected_genes
chr17	56356632	56357032	MPO	693_selected_genes
chr17	56357174	56357400	MPO	693_selected_genes
chr17	56357701	56357845	MPO	693_selected_genes
chr17	56357940	56358144	MPO	693_selected_genes
chr11	94153265	94153372	MRE11A	693_selected_genes

chr11	94163051	94163177	MRE11A	693_selected_genes
chr11	94168972	94169090	MRE11A	693_selected_genes
chr11	94170317	94170426	MRE11A	693_selected_genes
chr11	94178950	94179084	MRE11A	693_selected_genes
chr11	94180359	94180629	MRE11A	693_selected_genes
chr11	94189416	94189529	MRE11A	693_selected_genes
chr11	94192548	94192772	MRE11A	693_selected_genes
chr11	94194076	94194227	MRE11A	693_selected_genes
chr11	94197253	94197430	MRE11A	693_selected_genes
chr11	94200953	94201084	MRE11A	693_selected_genes
chr11	94203611	94203833	MRE11A	693_selected_genes
chr11	94204714	94204950	MRE11A	693_selected_genes
chr11	94208753	94208810	MRE11A	693_selected_genes
chr11	94209429	94209594	MRE11A	693_selected_genes
chr11	94211875	94212067	MRE11A	693_selected_genes
chr11	94212814	94212952	MRE11A	693_selected_genes
chr11	94219064	94219275	MRE11A	693_selected_genes
chr11	94223973	94224156	MRE11A	693_selected_genes
chr11	94225782	94225861	MRE11A	693_selected_genes
chr11	94225922	94225992	MRE11A	693_selected_genes
chr2	47630305	47630566	MSH2	693_selected_genes
chr2	47635514	47635719	MSH2	693_selected_genes
chr2	47637207	47637536	MSH2	693_selected_genes
chr2	47639527	47639724	MSH2	693_selected_genes
chr2	47641382	47641582	MSH2	693_selected_genes
chr2	47643409	47643593	MSH2	693_selected_genes
chr2	47656855	47657105	MSH2	693_selected_genes
chr2	47672661	47672821	MSH2	693_selected_genes
chr2	47690144	47690318	MSH2	693_selected_genes
chr2	47693771	47693972	MSH2	693_selected_genes
chr2	47698078	47698226	MSH2	693_selected_genes
chr2	47702138	47702434	MSH2	693_selected_genes
chr2	47703480	47703735	MSH2	693_selected_genes
chr2	47705385	47705683	MSH2	693_selected_genes
chr2	47707809	47708035	MSH2	693_selected_genes
chr2	47709892	47710113	MSH2	693_selected_genes
chr2	47739416	47739598	MSH2	693_selected_genes
chr2	48010347	48010657	MSH6	693_selected_genes
chr2	48018040	48018287	MSH6	693_selected_genes
chr2	48023007	48023227	MSH6	693_selected_genes
chr2	48025724	48028319	MSH6	693_selected_genes
chr2	48030533	48030849	MSH6	693_selected_genes
chr2	48032023	48032191	MSH6	693_selected_genes
chr2	48032731	48032871	MSH6	693_selected_genes
chr2	48033317	48033522	MSH6	693_selected_genes
chr2	48033565	48033815	MSH6	693_selected_genes
chr2	48033892	48034024	MSH6	693_selected_genes
chr1	11850711	11850980	MTHFR	693_selected_genes
chr1	11851238	11851408	MTHFR	693_selected_genes
chr1	11852309	11852461	MTHFR	693_selected_genes
chr1	11853938	11854171	MTHFR	693_selected_genes
chr1	11854389	11854620	MTHFR	693_selected_genes
chr1	11854760	11854945	MTHFR	693_selected_genes
chr1	11855129	11855430	MTHFR	693_selected_genes
chr1	11856237	11856481	MTHFR	693_selected_genes
chr1	11860243	11860404	MTHFR	693_selected_genes
chr1	11861192	11861481	MTHFR	693_selected_genes
chr1	11862912	11863267	MTHFR	693_selected_genes
chr1	11863436	11863596	MTHFR	693_selected_genes
chr1	11167516	11167582	MTOR	693_selected_genes
chr1	11168212	11168368	MTOR	693_selected_genes
chr1	11169321	11169452	MTOR	693_selected_genes
chr1	11169680	11169811	MTOR	693_selected_genes
chr1	11172883	11172999	MTOR	693_selected_genes
chr1	11174349	11174535	MTOR	693_selected_genes
chr1	11174844	11174969	MTOR	693_selected_genes
chr1	11175427	11175550	MTOR	693_selected_genes
chr1	11177035	11177168	MTOR	693_selected_genes
chr1	11181277	11181450	MTOR	693_selected_genes
chr1	11182010	11182208	MTOR	693_selected_genes
chr1	11184529	11184715	MTOR	693_selected_genes
chr1	11186653	11186878	MTOR	693_selected_genes
chr1	11187041	11187226	MTOR	693_selected_genes
chr1	11187655	11187888	MTOR	693_selected_genes
chr1	11188035	11188208	MTOR	693_selected_genes
chr1	11188485	11188634	MTOR	693_selected_genes
chr1	11188886	11189033	MTOR	693_selected_genes
chr1	11189769	11189920	MTOR	693_selected_genes
chr1	11190560	11190859	MTOR	693_selected_genes
chr1	11193111	11193279	MTOR	693_selected_genes
chr1	11194382	11194548	MTOR	693_selected_genes
chr1	11199335	11199517	MTOR	693_selected_genes
chr1	11199564	11199740	MTOR	693_selected_genes
chr1	11204679	11204837	MTOR	693_selected_genes

chr1	11204999	11205127	MTOR	693_selected_genes
chr1	11206707	11206873	MTOR	693_selected_genes
chr1	11210157	11210308	MTOR	693_selected_genes
chr1	11217183	11217373	MTOR	693_selected_genes
chr1	11227473	11227599	MTOR	693_selected_genes
chr1	11259289	11259485	MTOR	693_selected_genes
chr1	11259572	11259785	MTOR	693_selected_genes
chr1	11264592	11264785	MTOR	693_selected_genes
chr1	11269343	11269540	MTOR	693_selected_genes
chr1	11270845	11270988	MTOR	693_selected_genes
chr1	11272343	11272556	MTOR	693_selected_genes
chr1	11272827	11272990	MTOR	693_selected_genes
chr1	11273430	11273648	MTOR	693_selected_genes
chr1	11276179	11276316	MTOR	693_selected_genes
chr1	11288699	11289000	MTOR	693_selected_genes
chr1	11290956	11291136	MTOR	693_selected_genes
chr1	11291331	11291516	MTOR	693_selected_genes
chr1	11292467	11292610	MTOR	693_selected_genes
chr1	11293429	11293569	MTOR	693_selected_genes
chr1	11294174	11294347	MTOR	693_selected_genes
chr1	11297874	11298130	MTOR	693_selected_genes
chr1	11298433	11298699	MTOR	693_selected_genes
chr1	11300334	11300629	MTOR	693_selected_genes
chr1	11301584	11301763	MTOR	693_selected_genes
chr1	11303145	11303382	MTOR	693_selected_genes
chr1	11307656	11307815	MTOR	693_selected_genes
chr1	11307850	11308176	MTOR	693_selected_genes
chr1	11313870	11314055	MTOR	693_selected_genes
chr1	11316023	11316274	MTOR	693_selected_genes
chr1	11316964	11317247	MTOR	693_selected_genes
chr1	11318516	11318675	MTOR	693_selected_genes
chr1	11319279	11319491	MTOR	693_selected_genes
chr1	45794952	45795134	MUTYH	693_selected_genes
chr1	45795712	45795765	MUTYH	693_selected_genes
chr1	45796162	45796254	MUTYH	693_selected_genes
chr1	45796828	45797031	MUTYH	693_selected_genes
chr1	45797066	45797253	MUTYH	693_selected_genes
chr1	45797307	45797569	MUTYH	693_selected_genes
chr1	45797669	45797783	MUTYH	693_selected_genes
chr1	45797812	45798007	MUTYH	693_selected_genes
chr1	45798037	45798185	MUTYH	693_selected_genes
chr1	45798220	45798384	MUTYH	693_selected_genes
chr1	45798409	45798531	MUTYH	693_selected_genes
chr1	45798564	45798656	MUTYH	693_selected_genes
chr1	45798743	45798867	MUTYH	693_selected_genes
chr1	45798931	45799021	MUTYH	693_selected_genes
chr1	45799059	45799300	MUTYH	693_selected_genes
chr1	45800037	45800208	MUTYH	693_selected_genes
chr1	45803832	45803894	MUTYH	693_selected_genes
chr1	45805545	45805651	MUTYH	693_selected_genes
chr1	45805849	45805951	MUTYH	693_selected_genes
chr6	135502626	135502699	MYB	693_selected_genes
chr6	135507015	135507183	MYB	693_selected_genes
chr6	135508944	135509068	MYB	693_selected_genes
chr6	135510903	135511046	MYB	693_selected_genes
chr6	135511239	135511510	MYB	693_selected_genes
chr6	135513436	135513721	MYB	693_selected_genes
chr6	135514950	135515081	MYB	693_selected_genes
chr6	135515468	135515647	MYB	693_selected_genes
chr6	135516072	135516227	MYB	693_selected_genes
chr6	135516860	135517165	MYB	693_selected_genes
chr6	135517838	135517937	MYB	693_selected_genes
chr6	135518073	135518486	MYB	693_selected_genes
chr6	135520020	135520213	MYB	693_selected_genes
chr6	135520638	135520692	MYB	693_selected_genes
chr6	135521197	135521362	MYB	693_selected_genes
chr6	135521402	135521737	MYB	693_selected_genes
chr6	135522751	135522912	MYB	693_selected_genes
chr6	135523526	135523832	MYB	693_selected_genes
chr6	135524060	135524112	MYB	693_selected_genes
chr6	135524329	135524517	MYB	693_selected_genes
chr6	135538976	135539143	MYB	693_selected_genes
chr11	47353396	47353457	MYBPC3	693_selected_genes
chr11	47353597	47353834	MYBPC3	693_selected_genes
chr11	47354091	47354278	MYBPC3	693_selected_genes
chr11	47354339	47354549	MYBPC3	693_selected_genes
chr11	47354719	47354909	MYBPC3	693_selected_genes
chr11	47355082	47355328	MYBPC3	693_selected_genes
chr11	47355447	47355586	MYBPC3	693_selected_genes
chr11	47356567	47356785	MYBPC3	693_selected_genes
chr11	47357402	47357587	MYBPC3	693_selected_genes
chr11	47358916	47359155	MYBPC3	693_selected_genes
chr11	47359215	47359370	MYBPC3	693_selected_genes
chr11	47360045	47360255	MYBPC3	693_selected_genes

chr11	47360849	47360980	MYBPC3	693_selected_genes
chr11	47361176	47361366	MYBPC3	693_selected_genes
chr11	47362528	47362608	MYBPC3	693_selected_genes
chr11	47362663	47362820	MYBPC3	693_selected_genes
chr11	47363516	47363732	MYBPC3	693_selected_genes
chr11	47364103	47364320	MYBPC3	693_selected_genes
chr11	47364355	47364511	MYBPC3	693_selected_genes
chr11	47364546	47364724	MYBPC3	693_selected_genes
chr11	47364785	47364838	MYBPC3	693_selected_genes
chr11	47365017	47365200	MYBPC3	693_selected_genes
chr11	47367732	47367946	MYBPC3	693_selected_genes
chr11	47368152	47368223	MYBPC3	693_selected_genes
chr11	47368552	47368605	MYBPC3	693_selected_genes
chr11	47368947	47369055	MYBPC3	693_selected_genes
chr11	47369176	47369256	MYBPC3	693_selected_genes
chr11	47369382	47369481	MYBPC3	693_selected_genes
chr11	47369949	47370117	MYBPC3	693_selected_genes
chr11	47371299	47371498	MYBPC3	693_selected_genes
chr11	47371539	47371688	MYBPC3	693_selected_genes
chr11	47372027	47372191	MYBPC3	693_selected_genes
chr11	47372764	47373081	MYBPC3	693_selected_genes
chr11	47374148	47374223	MYBPC3	693_selected_genes
chr8	128748814	128748894	MYC	693_selected_genes
chr8	128750468	128751290	MYC	693_selected_genes
chr8	128752616	128753229	MYC	693_selected_genes
chr1	40363018	40363667	MYCL	693_selected_genes
chr1	40366460	40367140	MYCL	693_selected_genes
chr1	40367454	40367585	MYCL	693_selected_genes
chr2	16080683	16080890	MYCN	693_selected_genes
chr2	16082044	16083001	MYCN	693_selected_genes
chr2	16085589	16086244	MYCN	693_selected_genes
chr3	38180127	38180544	MYD88	693_selected_genes
chr3	38181329	38181514	MYD88	693_selected_genes
chr3	38181853	38182108	MYD88	693_selected_genes
chr3	38182222	38182364	MYD88	693_selected_genes
chr3	38182597	38182802	MYD88	693_selected_genes
chr14	23882037	23882105	MYH7	693_selected_genes
chr14	23882942	23883127	MYH7	693_selected_genes
chr14	23883190	23883336	MYH7	693_selected_genes
chr14	23884178	23884504	MYH7	693_selected_genes
chr14	23884564	23884740	MYH7	693_selected_genes
chr14	23884812	23885066	MYH7	693_selected_genes
chr14	23885187	23885546	MYH7	693_selected_genes
chr14	23886051	23886226	MYH7	693_selected_genes
chr14	23886336	23886552	MYH7	693_selected_genes
chr14	23886686	23886920	MYH7	693_selected_genes
chr14	23887393	23887640	MYH7	693_selected_genes
chr14	23888360	23888529	MYH7	693_selected_genes
chr14	23888666	23888843	MYH7	693_selected_genes
chr14	23889028	23889468	MYH7	693_selected_genes
chr14	23890141	23890282	MYH7	693_selected_genes
chr14	23891363	23891559	MYH7	693_selected_genes
chr14	23892730	23892957	MYH7	693_selected_genes
chr14	23893090	23893383	MYH7	693_selected_genes
chr14	23893952	23894258	MYH7	693_selected_genes
chr14	23894465	23894652	MYH7	693_selected_genes
chr14	23894878	23895052	MYH7	693_selected_genes
chr14	23895147	23895315	MYH7	693_selected_genes
chr14	23895960	23896098	MYH7	693_selected_genes
chr14	23896423	23896541	MYH7	693_selected_genes
chr14	23896768	23897128	MYH7	693_selected_genes
chr14	23897683	23897904	MYH7	693_selected_genes
chr14	23898138	23898338	MYH7	693_selected_genes
chr14	23898412	23898581	MYH7	693_selected_genes
chr14	23898958	23899147	MYH7	693_selected_genes
chr14	23899743	23899897	MYH7	693_selected_genes
chr14	23900084	23900233	MYH7	693_selected_genes
chr14	23900601	23900715	MYH7	693_selected_genes
chr14	23900768	23900911	MYH7	693_selected_genes
chr14	23900944	23901103	MYH7	693_selected_genes
chr14	23901662	23901740	MYH7	693_selected_genes
chr14	23901822	23902029	MYH7	693_selected_genes
chr14	23902267	23902461	MYH7	693_selected_genes
chr14	23902715	23902966	MYH7	693_selected_genes
chr22	36678688	36678856	MYH9	693_selected_genes
chr22	36680113	36680336	MYH9	693_selected_genes
chr22	36680423	36680582	MYH9	693_selected_genes
chr22	36681141	36681400	MYH9	693_selected_genes
chr22	36681678	36681852	MYH9	693_selected_genes
chr22	36681885	36682024	MYH9	693_selected_genes
chr22	36682738	36682917	MYH9	693_selected_genes
chr22	36684272	36684484	MYH9	693_selected_genes
chr22	36684747	36685010	MYH9	693_selected_genes
chr22	36685105	36685368	MYH9	693_selected_genes

chr22	36688006	36688305	MYH9	693_selected_genes
chr22	36689349	36689552	MYH9	693_selected_genes
chr22	36689779	36689934	MYH9	693_selected_genes
chr22	36690112	36690369	MYH9	693_selected_genes
chr22	36690952	36691147	MYH9	693_selected_genes
chr22	36691525	36691788	MYH9	693_selected_genes
chr22	36692863	36693085	MYH9	693_selected_genes
chr22	36694939	36695113	MYH9	693_selected_genes
chr22	36696147	36696335	MYH9	693_selected_genes
chr22	36696871	36697128	MYH9	693_selected_genes
chr22	36697554	36697736	MYH9	693_selected_genes
chr22	36698588	36698747	MYH9	693_selected_genes
chr22	36700015	36700226	MYH9	693_selected_genes
chr22	36701053	36701173	MYH9	693_selected_genes
chr22	36701950	36702122	MYH9	693_selected_genes
chr22	36702434	36702678	MYH9	693_selected_genes
chr22	36705301	36705466	MYH9	693_selected_genes
chr22	36708068	36708292	MYH9	693_selected_genes
chr22	36710164	36710388	MYH9	693_selected_genes
chr22	36712536	36712739	MYH9	693_selected_genes
chr22	36714226	36714395	MYH9	693_selected_genes
chr22	36715559	36715705	MYH9	693_selected_genes
chr22	36716239	36716433	MYH9	693_selected_genes
chr22	36716817	36716966	MYH9	693_selected_genes
chr22	36717777	36717891	MYH9	693_selected_genes
chr22	36718448	36718591	MYH9	693_selected_genes
chr22	36721098	36721193	MYH9	693_selected_genes
chr22	36722587	36722731	MYH9	693_selected_genes
chr22	36723480	36723558	MYH9	693_selected_genes
chr22	36737389	36737596	MYH9	693_selected_genes
chr22	36744923	36745306	MYH9	693_selected_genes
chr12	111348855	111349004	MYL2	693_selected_genes
chr12	111350874	111350973	MYL2	693_selected_genes
chr12	111351024	111351153	MYL2	693_selected_genes
chr12	111351964	111352153	MYL2	693_selected_genes
chr12	111353493	111353619	MYL2	693_selected_genes
chr12	111356882	111357022	MYL2	693_selected_genes
chr12	111358305	111358358	MYL2	693_selected_genes
chr3	46899708	46899787	MYL3	693_selected_genes
chr3	46899848	46899976	MYL3	693_selected_genes
chr3	46900939	46901163	MYL3	693_selected_genes
chr3	46902140	46902340	MYL3	693_selected_genes
chr3	46902424	46902502	MYL3	693_selected_genes
chr3	46904726	46904905	MYL3	693_selected_genes
chr3	123332926	123333221	MYLK	693_selected_genes
chr3	123337460	123337642	MYLK	693_selected_genes
chr3	123339028	123339208	MYLK	693_selected_genes
chr3	123345639	123345813	MYLK	693_selected_genes
chr3	123348295	123348498	MYLK	693_selected_genes
chr3	123356892	123357066	MYLK	693_selected_genes
chr3	123359108	123359376	MYLK	693_selected_genes
chr3	123366045	123366299	MYLK	693_selected_genes
chr3	123367792	123367936	MYLK	693_selected_genes
chr3	123367983	123368066	MYLK	693_selected_genes
chr3	123375947	123376300	MYLK	693_selected_genes
chr3	123382926	123383130	MYLK	693_selected_genes
chr3	123385040	123385218	MYLK	693_selected_genes
chr3	123385958	123386059	MYLK	693_selected_genes
chr3	123401045	123401182	MYLK	693_selected_genes
chr3	123411556	123411723	MYLK	693_selected_genes
chr3	123418841	123419877	MYLK	693_selected_genes
chr3	123420259	123420381	MYLK	693_selected_genes
chr3	123426575	123426875	MYLK	693_selected_genes
chr3	123427519	123427767	MYLK	693_selected_genes
chr3	123428577	123428765	MYLK	693_selected_genes
chr3	123440949	123441152	MYLK	693_selected_genes
chr3	123444765	123444950	MYLK	693_selected_genes
chr3	123451717	123451974	MYLK	693_selected_genes
chr3	123452508	123453094	MYLK	693_selected_genes
chr3	123454219	123454288	MYLK	693_selected_genes
chr3	123456199	123456415	MYLK	693_selected_genes
chr3	123457718	123457934	MYLK	693_selected_genes
chr3	123458774	123458873	MYLK	693_selected_genes
chr3	123471152	123471410	MYLK	693_selected_genes
chr3	123512498	123512713	MYLK	693_selected_genes
chr11	17741304	17741984	MYOD1	693_selected_genes
chr11	17742423	17742552	MYOD1	693_selected_genes
chr11	17742776	17743080	MYOD1	693_selected_genes
chr12	78225216	78225509	NAV3	693_selected_genes
chr12	78334073	78334241	NAV3	693_selected_genes
chr12	78334835	78334938	NAV3	693_selected_genes
chr12	78359983	78360106	NAV3	693_selected_genes
chr12	78362273	78362507	NAV3	693_selected_genes
chr12	78388557	78388676	NAV3	693_selected_genes

chr12	78392091	78392281	NAV3	693_selected_genes
chr12	78400173	78401250	NAV3	693_selected_genes
chr12	78415501	78415667	NAV3	693_selected_genes
chr12	78443747	78443906	NAV3	693_selected_genes
chr12	78444518	78444952	NAV3	693_selected_genes
chr12	78452750	78452920	NAV3	693_selected_genes
chr12	78510526	78510709	NAV3	693_selected_genes
chr12	78511781	78512102	NAV3	693_selected_genes
chr12	78512991	78513750	NAV3	693_selected_genes
chr12	78515694	78516233	NAV3	693_selected_genes
chr12	78520921	78521013	NAV3	693_selected_genes
chr12	78522460	78522671	NAV3	693_selected_genes
chr12	78530931	78531170	NAV3	693_selected_genes
chr12	78534036	78534139	NAV3	693_selected_genes
chr12	78537148	78537222	NAV3	693_selected_genes
chr12	78540123	78540197	NAV3	693_selected_genes
chr12	78542596	78542724	NAV3	693_selected_genes
chr12	78552957	78553091	NAV3	693_selected_genes
chr12	78562509	78562671	NAV3	693_selected_genes
chr12	78569060	78569232	NAV3	693_selected_genes
chr12	78570193	78570264	NAV3	693_selected_genes
chr12	78570895	78571118	NAV3	693_selected_genes
chr12	78571374	78571490	NAV3	693_selected_genes
chr12	78573283	78573487	NAV3	693_selected_genes
chr12	78574625	78574850	NAV3	693_selected_genes
chr12	78579355	78579503	NAV3	693_selected_genes
chr12	78582002	78582148	NAV3	693_selected_genes
chr12	78582363	78582582	NAV3	693_selected_genes
chr12	78583738	78584024	NAV3	693_selected_genes
chr12	78591001	78591206	NAV3	693_selected_genes
chr12	78592359	78592481	NAV3	693_selected_genes
chr12	78592830	78593000	NAV3	693_selected_genes
chr12	78593089	78593336	NAV3	693_selected_genes
chr12	78594227	78594396	NAV3	693_selected_genes
chr12	78598689	78598943	NAV3	693_selected_genes
chr12	78604152	78604322	NAV3	693_selected_genes
chr8	90947784	90947865	NBN	693_selected_genes
chr8	90949228	90949328	NBN	693_selected_genes
chr8	90955455	90955619	NBN	693_selected_genes
chr8	90958342	90958548	NBN	693_selected_genes
chr8	90960026	90960145	NBN	693_selected_genes
chr8	90965446	90965944	NBN	693_selected_genes
chr8	90967485	90967808	NBN	693_selected_genes
chr8	90970927	90971107	NBN	693_selected_genes
chr8	90976612	90976760	NBN	693_selected_genes
chr8	90982566	90982810	NBN	693_selected_genes
chr8	90983375	90983543	NBN	693_selected_genes
chr8	90990422	90990576	NBN	693_selected_genes
chr8	90992936	90993146	NBN	693_selected_genes
chr8	90993577	90993776	NBN	693_selected_genes
chr8	90994199	90994255	NBN	693_selected_genes
chr8	90994924	90995108	NBN	693_selected_genes
chr8	90996727	90996814	NBN	693_selected_genes
chr20	46250966	46251099	NCOA3	693_selected_genes
chr20	46252629	46252852	NCOA3	693_selected_genes
chr20	46254099	46254250	NCOA3	693_selected_genes
chr20	46255720	46255945	NCOA3	693_selected_genes
chr20	46256279	46256518	NCOA3	693_selected_genes
chr20	46256640	46256792	NCOA3	693_selected_genes
chr20	46262214	46262405	NCOA3	693_selected_genes
chr20	46262736	46262964	NCOA3	693_selected_genes
chr20	46264040	46264482	NCOA3	693_selected_genes
chr20	46264609	46265531	NCOA3	693_selected_genes
chr20	46266366	46266552	NCOA3	693_selected_genes
chr20	46267726	46267971	NCOA3	693_selected_genes
chr20	46268295	46268591	NCOA3	693_selected_genes
chr20	46268643	46268820	NCOA3	693_selected_genes
chr20	46270931	46271153	NCOA3	693_selected_genes
chr20	46275791	46276135	NCOA3	693_selected_genes
chr20	46277723	46277878	NCOA3	693_selected_genes
chr20	46279700	46280045	NCOA3	693_selected_genes
chr20	46281124	46281349	NCOA3	693_selected_genes
chr20	46281649	46281841	NCOA3	693_selected_genes
chr20	46282124	46282186	NCOA3	693_selected_genes
chr17	15935584	15935822	NCOR1	693_selected_genes
chr17	15938053	15938283	NCOR1	693_selected_genes
chr17	15942581	15942993	NCOR1	693_selected_genes
chr17	15943729	15943833	NCOR1	693_selected_genes
chr17	15950239	15950432	NCOR1	693_selected_genes
chr17	15952133	15952327	NCOR1	693_selected_genes
chr17	15960802	15961076	NCOR1	693_selected_genes
chr17	15961195	15961403	NCOR1	693_selected_genes
chr17	15961759	15961938	NCOR1	693_selected_genes
chr17	15964689	15965233	NCOR1	693_selected_genes

chr17	15965393	15965609	NCOR1	693_selected_genes
chr17	15967356	15967526	NCOR1	693_selected_genes
chr17	15968158	15968358	NCOR1	693_selected_genes
chr17	15968773	15969033	NCOR1	693_selected_genes
chr17	15971182	15971460	NCOR1	693_selected_genes
chr17	15973453	15973864	NCOR1	693_selected_genes
chr17	15974697	15975004	NCOR1	693_selected_genes
chr17	15975433	15975567	NCOR1	693_selected_genes
chr17	15976717	15976908	NCOR1	693_selected_genes
chr17	15978822	15979041	NCOR1	693_selected_genes
chr17	15983252	15983403	NCOR1	693_selected_genes
chr17	15983696	15983848	NCOR1	693_selected_genes
chr17	15983895	15984066	NCOR1	693_selected_genes
chr17	15989570	15989781	NCOR1	693_selected_genes
chr17	15990460	15990684	NCOR1	693_selected_genes
chr17	15995143	15995397	NCOR1	693_selected_genes
chr17	16001655	16001835	NCOR1	693_selected_genes
chr17	16004538	16005144	NCOR1	693_selected_genes
chr17	16012074	16012251	NCOR1	693_selected_genes
chr17	16021176	16021366	NCOR1	693_selected_genes
chr17	16022711	16022824	NCOR1	693_selected_genes
chr17	16024340	16024608	NCOR1	693_selected_genes
chr17	16029370	16029548	NCOR1	693_selected_genes
chr17	16040599	16040751	NCOR1	693_selected_genes
chr17	16041446	16041551	NCOR1	693_selected_genes
chr17	16042296	16042525	NCOR1	693_selected_genes
chr17	16046894	16047035	NCOR1	693_selected_genes
chr17	16049664	16049887	NCOR1	693_selected_genes
chr17	16052739	16052856	NCOR1	693_selected_genes
chr17	16054878	16054955	NCOR1	693_selected_genes
chr17	16055234	16055337	NCOR1	693_selected_genes
chr17	16056627	16056734	NCOR1	693_selected_genes
chr17	16062048	16062212	NCOR1	693_selected_genes
chr17	16068267	16068500	NCOR1	693_selected_genes
chr17	16075091	16075359	NCOR1	693_selected_genes
chr17	16089842	16090026	NCOR1	693_selected_genes
chr17	16097750	16097908	NCOR1	693_selected_genes
chr12	124809922	124810154	NCOR2	693_selected_genes
chr12	124810700	124810941	NCOR2	693_selected_genes
chr12	124811929	124812204	NCOR2	693_selected_genes
chr12	124813604	124813728	NCOR2	693_selected_genes
chr12	124815365	124815469	NCOR2	693_selected_genes
chr12	124816839	124817038	NCOR2	693_selected_genes
chr12	124817650	124817850	NCOR2	693_selected_genes
chr12	124818944	124819217	NCOR2	693_selected_genes
chr12	124819655	124819851	NCOR2	693_selected_genes
chr12	124820003	124820203	NCOR2	693_selected_genes
chr12	124821273	124821751	NCOR2	693_selected_genes
chr12	124824526	124824775	NCOR2	693_selected_genes
chr12	124824814	124825014	NCOR2	693_selected_genes
chr12	124825122	124825322	NCOR2	693_selected_genes
chr12	124826343	124826645	NCOR2	693_selected_genes
chr12	124827525	124827797	NCOR2	693_selected_genes
chr12	124829117	124829522	NCOR2	693_selected_genes
chr12	124831084	124831421	NCOR2	693_selected_genes
chr12	124832345	124832482	NCOR2	693_selected_genes
chr12	124832694	124832885	NCOR2	693_selected_genes
chr12	124835107	124835308	NCOR2	693_selected_genes
chr12	124838613	124838767	NCOR2	693_selected_genes
chr12	124838998	124839153	NCOR2	693_selected_genes
chr12	124839357	124839510	NCOR2	693_selected_genes
chr12	124839952	124840142	NCOR2	693_selected_genes
chr12	124841162	124841377	NCOR2	693_selected_genes
chr12	124846646	124846868	NCOR2	693_selected_genes
chr12	124848199	124848370	NCOR2	693_selected_genes
chr12	124856542	124857181	NCOR2	693_selected_genes
chr12	124858933	124859034	NCOR2	693_selected_genes
chr12	124862757	124862955	NCOR2	693_selected_genes
chr12	124870265	124870458	NCOR2	693_selected_genes
chr12	124873205	124873301	NCOR2	693_selected_genes
chr12	124882639	124882752	NCOR2	693_selected_genes
chr12	124885021	124885244	NCOR2	693_selected_genes
chr12	124886924	124887132	NCOR2	693_selected_genes
chr12	124904477	124904626	NCOR2	693_selected_genes
chr12	124907021	124907126	NCOR2	693_selected_genes
chr12	124911142	124911371	NCOR2	693_selected_genes
chr12	124914133	124914277	NCOR2	693_selected_genes
chr12	124915131	124915358	NCOR2	693_selected_genes
chr12	124922450	124922567	NCOR2	693_selected_genes
chr12	124934335	124934438	NCOR2	693_selected_genes
chr12	124941626	124941733	NCOR2	693_selected_genes
chr12	124950693	124950857	NCOR2	693_selected_genes
chr12	124957472	124957702	NCOR2	693_selected_genes
chr12	124968116	124968345	NCOR2	693_selected_genes

chr12	124970961	124971139	NCOR2	693_selected_genes
chr12	124979667	124979822	NCOR2	693_selected_genes
chr18	55711867	55711965	NEDD4L	693_selected_genes
chr18	55816742	55816816	NEDD4L	693_selected_genes
chr18	55832994	55833118	NEDD4L	693_selected_genes
chr18	55912633	55912765	NEDD4L	693_selected_genes
chr18	55916105	55916194	NEDD4L	693_selected_genes
chr18	55919207	55919311	NEDD4L	693_selected_genes
chr18	55983188	55983289	NEDD4L	693_selected_genes
chr18	55989631	55989743	NEDD4L	693_selected_genes
chr18	55990438	55990591	NEDD4L	693_selected_genes
chr18	55992202	55992419	NEDD4L	693_selected_genes
chr18	55996201	55996384	NEDD4L	693_selected_genes
chr18	55997944	55998171	NEDD4L	693_selected_genes
chr18	56001024	56001149	NEDD4L	693_selected_genes
chr18	56002684	56002794	NEDD4L	693_selected_genes
chr18	56008244	56008426	NEDD4L	693_selected_genes
chr18	56008884	56009054	NEDD4L	693_selected_genes
chr18	56010112	56010360	NEDD4L	693_selected_genes
chr18	56016743	56016871	NEDD4L	693_selected_genes
chr18	56018197	56018302	NEDD4L	693_selected_genes
chr18	56024400	56024509	NEDD4L	693_selected_genes
chr18	56024663	56024716	NEDD4L	693_selected_genes
chr18	56031474	56031590	NEDD4L	693_selected_genes
chr18	56033205	56033485	NEDD4L	693_selected_genes
chr18	56034952	56035124	NEDD4L	693_selected_genes
chr18	56037603	56037724	NEDD4L	693_selected_genes
chr18	56040380	56040526	NEDD4L	693_selected_genes
chr18	56050452	56050576	NEDD4L	693_selected_genes
chr18	56052732	56052843	NEDD4L	693_selected_genes
chr18	56054645	56054755	NEDD4L	693_selected_genes
chr18	56056291	56056449	NEDD4L	693_selected_genes
chr18	56057852	56057999	NEDD4L	693_selected_genes
chr18	56058693	56058816	NEDD4L	693_selected_genes
chr18	56063373	56063526	NEDD4L	693_selected_genes
chr17	29422200	29422412	NF1	693_selected_genes
chr17	29482975	29483169	NF1	693_selected_genes
chr17	29486002	29486136	NF1	693_selected_genes
chr17	29490178	29490419	NF1	693_selected_genes
chr17	29496883	29497040	NF1	693_selected_genes
chr17	29508414	29508532	NF1	693_selected_genes
chr17	29508702	29508828	NF1	693_selected_genes
chr17	29509500	29509708	NF1	693_selected_genes
chr17	29527414	29527638	NF1	693_selected_genes
chr17	29528029	29528202	NF1	693_selected_genes
chr17	29528403	29528528	NF1	693_selected_genes
chr17	29533232	29533414	NF1	693_selected_genes
chr17	29541443	29541628	NF1	693_selected_genes
chr17	29545997	29546161	NF1	693_selected_genes
chr17	29548842	29549033	NF1	693_selected_genes
chr17	29550436	29550610	NF1	693_selected_genes
chr17	29552087	29552293	NF1	693_selected_genes
chr17	29553427	29553727	NF1	693_selected_genes
chr17	29554210	29554334	NF1	693_selected_genes
chr17	29554515	29554649	NF1	693_selected_genes
chr17	29556017	29556508	NF1	693_selected_genes
chr17	29556827	29557017	NF1	693_selected_genes
chr17	29557252	29557425	NF1	693_selected_genes
chr17	29557834	29557968	NF1	693_selected_genes
chr17	29559065	29559232	NF1	693_selected_genes
chr17	29559692	29559924	NF1	693_selected_genes
chr17	29559994	29560256	NF1	693_selected_genes
chr17	29562603	29562815	NF1	693_selected_genes
chr17	29562910	29563064	NF1	693_selected_genes
chr17	29575976	29576162	NF1	693_selected_genes
chr17	29579930	29580043	NF1	693_selected_genes
chr17	29584679	29584762	NF1	693_selected_genes
chr17	29585336	29585545	NF1	693_selected_genes
chr17	29586024	29586172	NF1	693_selected_genes
chr17	29587361	29587558	NF1	693_selected_genes
chr17	29588703	29588900	NF1	693_selected_genes
chr17	29592221	29592382	NF1	693_selected_genes
chr17	29626502	29626649	NF1	693_selected_genes
chr17	29652812	29653295	NF1	693_selected_genes
chr17	29654491	29654882	NF1	693_selected_genes
chr17	29657288	29657541	NF1	693_selected_genes
chr17	29661656	29661773	NF1	693_selected_genes
chr17	29661830	29662074	NF1	693_selected_genes
chr17	29663325	29663516	NF1	693_selected_genes
chr17	29663627	29663957	NF1	693_selected_genes
chr17	29664360	29664625	NF1	693_selected_genes
chr17	29664809	29664923	NF1	693_selected_genes
chr17	29664994	29665182	NF1	693_selected_genes
chr17	29665696	29665848	NF1	693_selected_genes

chr17	29667497	29667688	NF1	693_selected_genes
chr17	29670001	29670178	NF1	693_selected_genes
chr17	29676112	29676294	NF1	693_selected_genes
chr17	29677175	29677361	NF1	693_selected_genes
chr17	29679249	29679457	NF1	693_selected_genes
chr17	29683452	29683625	NF1	693_selected_genes
chr17	29683952	29684133	NF1	693_selected_genes
chr17	29684261	29684412	NF1	693_selected_genes
chr17	29685472	29685665	NF1	693_selected_genes
chr17	29685961	29686058	NF1	693_selected_genes
chr17	29687479	29687754	NF1	693_selected_genes
chr17	29694217	29694321	NF1	693_selected_genes
chr17	29701005	29701198	NF1	693_selected_genes
chr17	29705880	29705974	NF1	693_selected_genes
chr22	29999962	30000126	NF2	693_selected_genes
chr22	30032714	30032890	NF2	693_selected_genes
chr22	30035053	30035226	NF2	693_selected_genes
chr22	30038165	30038299	NF2	693_selected_genes
chr22	30050620	30050739	NF2	693_selected_genes
chr22	30051557	30051690	NF2	693_selected_genes
chr22	30054152	30054278	NF2	693_selected_genes
chr22	30057168	30057353	NF2	693_selected_genes
chr22	30060953	30061078	NF2	693_selected_genes
chr22	30064296	30064460	NF2	693_selected_genes
chr22	30067789	30067962	NF2	693_selected_genes
chr22	30069232	30069500	NF2	693_selected_genes
chr22	30070799	30070955	NF2	693_selected_genes
chr22	30074159	30074337	NF2	693_selected_genes
chr22	30077402	30077615	NF2	693_selected_genes
chr22	30078983	30079069	NF2	693_selected_genes
chr22	30090715	30090816	NF2	693_selected_genes
chr2	178092608	178092706	NFE2L2	693_selected_genes
chr2	178095487	178096761	NFE2L2	693_selected_genes
chr2	178097094	178097336	NFE2L2	693_selected_genes
chr2	178097880	178098092	NFE2L2	693_selected_genes
chr2	178098707	178099024	NFE2L2	693_selected_genes
chr2	178129234	178129329	NFE2L2	693_selected_genes
chr2	178175666	178175758	NFE2L2	693_selected_genes
chr4	103432064	103432131	NFKB1	693_selected_genes
chr4	103446643	103446739	NFKB1	693_selected_genes
chr4	103450967	103451096	NFKB1	693_selected_genes
chr4	103454976	103455067	NFKB1	693_selected_genes
chr4	103458989	103459138	NFKB1	693_selected_genes
chr4	103488118	103488317	NFKB1	693_selected_genes
chr4	103498007	103498221	NFKB1	693_selected_genes
chr4	103499984	103500221	NFKB1	693_selected_genes
chr4	103501666	103501821	NFKB1	693_selected_genes
chr4	103503997	103504139	NFKB1	693_selected_genes
chr4	103505813	103506002	NFKB1	693_selected_genes
chr4	103514556	103514750	NFKB1	693_selected_genes
chr4	103516023	103516163	NFKB1	693_selected_genes
chr4	103517269	103517514	NFKB1	693_selected_genes
chr4	103518651	103518843	NFKB1	693_selected_genes
chr4	103522026	103522191	NFKB1	693_selected_genes
chr4	103527627	103527879	NFKB1	693_selected_genes
chr4	103528281	103528501	NFKB1	693_selected_genes
chr4	103528780	103528933	NFKB1	693_selected_genes
chr4	103531706	103531881	NFKB1	693_selected_genes
chr4	103533175	103533292	NFKB1	693_selected_genes
chr4	103533565	103533788	NFKB1	693_selected_genes
chr4	103534556	103534763	NFKB1	693_selected_genes
chr4	103537565	103537776	NFKB1	693_selected_genes
chr10	104155691	104155762	NFKB2	693_selected_genes
chr10	104155984	104156116	NFKB2	693_selected_genes
chr10	104156180	104156271	NFKB2	693_selected_genes
chr10	104156456	104156605	NFKB2	693_selected_genes
chr10	104156635	104156837	NFKB2	693_selected_genes
chr10	104157033	104157190	NFKB2	693_selected_genes
chr10	104157258	104157467	NFKB2	693_selected_genes
chr10	104157712	104157867	NFKB2	693_selected_genes
chr10	104157943	104158079	NFKB2	693_selected_genes
chr10	104158116	104158305	NFKB2	693_selected_genes
chr10	104158470	104158646	NFKB2	693_selected_genes
chr10	104159019	104159279	NFKB2	693_selected_genes
chr10	104159308	104159500	NFKB2	693_selected_genes
chr10	104159811	104159976	NFKB2	693_selected_genes
chr10	104160009	104160273	NFKB2	693_selected_genes
chr10	104160386	104160606	NFKB2	693_selected_genes
chr10	104160678	104160831	NFKB2	693_selected_genes
chr10	104160911	104161113	NFKB2	693_selected_genes
chr10	104161180	104161300	NFKB2	693_selected_genes
chr10	104161476	104161699	NFKB2	693_selected_genes
chr10	104161779	104161941	NFKB2	693_selected_genes
chr10	104161983	104162158	NFKB2	693_selected_genes

chr14	35871193	35871291	NFKBIA	693_selected_genes
chr14	35871574	35871894	NFKBIA	693_selected_genes
chr14	35871951	35872090	NFKBIA	693_selected_genes
chr14	35872330	35872591	NFKBIA	693_selected_genes
chr14	35872870	35873029	NFKBIA	693_selected_genes
chr14	35873598	35873875	NFKBIA	693_selected_genes
chr14	51190155	51190415	NIN	693_selected_genes
chr14	51192564	51192809	NIN	693_selected_genes
chr14	51194381	51194494	NIN	693_selected_genes
chr14	51196215	51196466	NIN	693_selected_genes
chr14	51197615	51197726	NIN	693_selected_genes
chr14	51202208	51202360	NIN	693_selected_genes
chr14	51204832	51205029	NIN	693_selected_genes
chr14	51206000	51206230	NIN	693_selected_genes
chr14	51208274	51208471	NIN	693_selected_genes
chr14	51210108	51210272	NIN	693_selected_genes
chr14	51210935	51211108	NIN	693_selected_genes
chr14	51214684	51214848	NIN	693_selected_genes
chr14	51219210	51219476	NIN	693_selected_genes
chr14	51221255	51221375	NIN	693_selected_genes
chr14	51221434	51221610	NIN	693_selected_genes
chr14	51223184	51225373	NIN	693_selected_genes
chr14	51226549	51227102	NIN	693_selected_genes
chr14	51228482	51228654	NIN	693_selected_genes
chr14	51230518	51230707	NIN	693_selected_genes
chr14	51232999	51233139	NIN	693_selected_genes
chr14	51233472	51233633	NIN	693_selected_genes
chr14	51237080	51237305	NIN	693_selected_genes
chr14	51237544	51237735	NIN	693_selected_genes
chr14	51238024	51238211	NIN	693_selected_genes
chr14	51238993	51239211	NIN	693_selected_genes
chr14	51239641	51239838	NIN	693_selected_genes
chr14	51243641	51243882	NIN	693_selected_genes
chr14	51245457	51245547	NIN	693_selected_genes
chr14	51259191	51259256	NIN	693_selected_genes
chr14	51259404	51259624	NIN	693_selected_genes
chr14	51273429	51273561	NIN	693_selected_genes
chr14	51288566	51288799	NIN	693_selected_genes
chr14	51289872	51290047	NIN	693_selected_genes
chr14	51297156	51297260	NIN	693_selected_genes
chr14	51297698	51297851	NIN	693_selected_genes
chr5	36953773	36953887	NIPBL	693_selected_genes
chr5	36955548	36955764	NIPBL	693_selected_genes
chr5	36958180	36958358	NIPBL	693_selected_genes
chr5	36961560	36961710	NIPBL	693_selected_genes
chr5	36962199	36962401	NIPBL	693_selected_genes
chr5	36970952	36971163	NIPBL	693_selected_genes
chr5	36972021	36972168	NIPBL	693_selected_genes
chr5	36975852	36976529	NIPBL	693_selected_genes
chr5	36984752	36986428	NIPBL	693_selected_genes
chr5	36995698	36995931	NIPBL	693_selected_genes
chr5	37000449	37000697	NIPBL	693_selected_genes
chr5	37000893	37001015	NIPBL	693_selected_genes
chr5	37001065	37001205	NIPBL	693_selected_genes
chr5	37002738	37002892	NIPBL	693_selected_genes
chr5	37003337	37003474	NIPBL	693_selected_genes
chr5	37006433	37006715	NIPBL	693_selected_genes
chr5	37007399	37007601	NIPBL	693_selected_genes
chr5	37008084	37008215	NIPBL	693_selected_genes
chr5	37008699	37008850	NIPBL	693_selected_genes
chr5	37010163	37010352	NIPBL	693_selected_genes
chr5	37014759	37014892	NIPBL	693_selected_genes
chr5	37016114	37016297	NIPBL	693_selected_genes
chr5	37017095	37017289	NIPBL	693_selected_genes
chr5	37019387	37019527	NIPBL	693_selected_genes
chr5	37020535	37020800	NIPBL	693_selected_genes
chr5	37020851	37021004	NIPBL	693_selected_genes
chr5	37022127	37022276	NIPBL	693_selected_genes
chr5	37022320	37022517	NIPBL	693_selected_genes
chr5	37024661	37024846	NIPBL	693_selected_genes
chr5	37026305	37026454	NIPBL	693_selected_genes
chr5	37027435	37027539	NIPBL	693_selected_genes
chr5	37036455	37036614	NIPBL	693_selected_genes
chr5	37038678	37038865	NIPBL	693_selected_genes
chr5	37044423	37044614	NIPBL	693_selected_genes
chr5	37044712	37044856	NIPBL	693_selected_genes
chr5	37045519	37045724	NIPBL	693_selected_genes
chr5	37046185	37046326	NIPBL	693_selected_genes
chr5	37048578	37048802	NIPBL	693_selected_genes
chr5	37049187	37049428	NIPBL	693_selected_genes
chr5	37051855	37052013	NIPBL	693_selected_genes
chr5	37052442	37052693	NIPBL	693_selected_genes
chr5	37057262	37057459	NIPBL	693_selected_genes
chr5	37058967	37059292	NIPBL	693_selected_genes

chr5	37060920	37061145	NIPBL	693_selected_genes
chr5	37063866	37064150	NIPBL	693_selected_genes
chr5	37064603	37065019	NIPBL	693_selected_genes
chr14	36986457	36987250	NKX2-1	693_selected_genes
chr14	36988164	36988600	NKX2-1	693_selected_genes
chr14	36988761	36988842	NKX2-1	693_selected_genes
chr14	36989232	36989359	NKX2-1	693_selected_genes
chr9	139390497	139392035	NOTCH1	693_selected_genes
chr9	139393325	139393473	NOTCH1	693_selected_genes
chr9	139393538	139393736	NOTCH1	693_selected_genes
chr9	139394978	139395324	NOTCH1	693_selected_genes
chr9	139396174	139396390	NOTCH1	693_selected_genes
chr9	139396427	139396565	NOTCH1	693_selected_genes
chr9	139396698	139396965	NOTCH1	693_selected_genes
chr9	139397608	139397807	NOTCH1	693_selected_genes
chr9	139399099	139399581	NOTCH1	693_selected_genes
chr9	139399736	139400358	NOTCH1	693_selected_genes
chr9	139400953	139401116	NOTCH1	693_selected_genes
chr9	139401142	139401450	NOTCH1	693_selected_genes
chr9	139401731	139401914	NOTCH1	693_selected_genes
chr9	139402381	139402616	NOTCH1	693_selected_genes
chr9	139402658	139402862	NOTCH1	693_selected_genes
chr9	139403296	139403548	NOTCH1	693_selected_genes
chr9	139404159	139404438	NOTCH1	693_selected_genes
chr9	139405079	139405282	NOTCH1	693_selected_genes
chr9	139405578	139405748	NOTCH1	693_selected_genes
chr9	139407447	139407611	NOTCH1	693_selected_genes
chr9	139407818	139408014	NOTCH1	693_selected_genes
chr9	139408936	139409179	NOTCH1	693_selected_genes
chr9	139409716	139409877	NOTCH1	693_selected_genes
chr9	139409909	139410193	NOTCH1	693_selected_genes
chr9	139410407	139410571	NOTCH1	693_selected_genes
chr9	139411698	139411862	NOTCH1	693_selected_genes
chr9	139412178	139412414	NOTCH1	693_selected_genes
chr9	139412563	139412769	NOTCH1	693_selected_genes
chr9	139413017	139413301	NOTCH1	693_selected_genes
chr9	139413869	139414042	NOTCH1	693_selected_genes
chr9	139417276	139417665	NOTCH1	693_selected_genes
chr9	139418143	139418456	NOTCH1	693_selected_genes
chr9	139438450	139438579	NOTCH1	693_selected_genes
chr9	139440152	139440263	NOTCH1	693_selected_genes
chr1	120457903	120459342	NOTCH2	693_selected_genes
chr1	120460262	120460410	NOTCH2	693_selected_genes
chr1	120461003	120461201	NOTCH2	693_selected_genes
chr1	120461909	120462261	NOTCH2	693_selected_genes
chr1	120462826	120463045	NOTCH2	693_selected_genes
chr1	120464310	120464457	NOTCH2	693_selected_genes
chr1	120464833	120465094	NOTCH2	693_selected_genes
chr1	120465233	120465426	NOTCH2	693_selected_genes
chr1	120466234	120466632	NOTCH2	693_selected_genes
chr1	120467902	120468458	NOTCH2	693_selected_genes
chr1	120469096	120469259	NOTCH2	693_selected_genes
chr1	120471573	120471860	NOTCH2	693_selected_genes
chr1	120478016	120478252	NOTCH2	693_selected_genes
chr1	120479879	120480114	NOTCH2	693_selected_genes
chr1	120480454	120480658	NOTCH2	693_selected_genes
chr1	120483152	120483404	NOTCH2	693_selected_genes
chr1	120484123	120484402	NOTCH2	693_selected_genes
chr1	120491011	120491214	NOTCH2	693_selected_genes
chr1	120491604	120491774	NOTCH2	693_selected_genes
chr1	120493321	120493485	NOTCH2	693_selected_genes
chr1	120496140	120496336	NOTCH2	693_selected_genes
chr1	120497637	120497880	NOTCH2	693_selected_genes
chr1	120501989	120502150	NOTCH2	693_selected_genes
chr1	120506171	120506455	NOTCH2	693_selected_genes
chr1	120508050	120508214	NOTCH2	693_selected_genes
chr1	120508973	120509137	NOTCH2	693_selected_genes
chr1	120510030	120510269	NOTCH2	693_selected_genes
chr1	120510674	120510880	NOTCH2	693_selected_genes
chr1	120512108	120512392	NOTCH2	693_selected_genes
chr1	120529557	120529730	NOTCH2	693_selected_genes
chr1	120539270	120539394	NOTCH2	693_selected_genes
chr1	120539594	120539980	NOTCH2	693_selected_genes
chr1	120547926	120548236	NOTCH2	693_selected_genes
chr1	120572503	120572635	NOTCH2	693_selected_genes
chr1	120611922	120612045	NOTCH2	693_selected_genes
chr19	15271447	15272550	NOTCH3	693_selected_genes
chr19	15273145	15273398	NOTCH3	693_selected_genes
chr19	15276153	15276351	NOTCH3	693_selected_genes
chr19	15276572	15276927	NOTCH3	693_selected_genes
chr19	15278034	15278247	NOTCH3	693_selected_genes
chr19	15280871	15281006	NOTCH3	693_selected_genes
chr19	15281116	15281389	NOTCH3	693_selected_genes
chr19	15281456	15281661	NOTCH3	693_selected_genes

chr19	15284853	15285236	NOTCH3	693_selected_genes
chr19	15288310	15288926	NOTCH3	693_selected_genes
chr19	15289608	15289777	NOTCH3	693_selected_genes
chr19	15289810	15290118	NOTCH3	693_selected_genes
chr19	15290149	15290332	NOTCH3	693_selected_genes
chr19	15290857	15291092	NOTCH3	693_selected_genes
chr19	15291466	15291664	NOTCH3	693_selected_genes
chr19	15291746	15291998	NOTCH3	693_selected_genes
chr19	15292361	15292637	NOTCH3	693_selected_genes
chr19	15295080	15295286	NOTCH3	693_selected_genes
chr19	15295691	15295855	NOTCH3	693_selected_genes
chr19	15296042	15296244	NOTCH3	693_selected_genes
chr19	15296272	15296515	NOTCH3	693_selected_genes
chr19	15297663	15297824	NOTCH3	693_selected_genes
chr19	15297890	15298174	NOTCH3	693_selected_genes
chr19	15298666	15298830	NOTCH3	693_selected_genes
chr19	15299020	15299184	NOTCH3	693_selected_genes
chr19	15299774	15300010	NOTCH3	693_selected_genes
chr19	15300058	15300264	NOTCH3	693_selected_genes
chr19	15302209	15302493	NOTCH3	693_selected_genes
chr19	15302530	15302703	NOTCH3	693_selected_genes
chr19	15302745	15303134	NOTCH3	693_selected_genes
chr19	15303162	15303355	NOTCH3	693_selected_genes
chr19	15308285	15308414	NOTCH3	693_selected_genes
chr19	15311573	15311741	NOTCH3	693_selected_genes
chr6	32163188	32163952	NOTCH4	693_selected_genes
chr6	32164075	32164223	NOTCH4	693_selected_genes
chr6	32164676	32164874	NOTCH4	693_selected_genes
chr6	32165050	32165396	NOTCH4	693_selected_genes
chr6	32166172	32166361	NOTCH4	693_selected_genes
chr6	32166400	32166532	NOTCH4	693_selected_genes
chr6	32166677	32166947	NOTCH4	693_selected_genes
chr6	32168582	32168808	NOTCH4	693_selected_genes
chr6	32168868	32169302	NOTCH4	693_selected_genes
chr6	32169827	32170401	NOTCH4	693_selected_genes
chr6	32171521	32171684	NOTCH4	693_selected_genes
chr6	32171888	32172191	NOTCH4	693_selected_genes
chr6	32178503	32178738	NOTCH4	693_selected_genes
chr6	32180225	32180429	NOTCH4	693_selected_genes
chr6	32180575	32180713	NOTCH4	693_selected_genes
chr6	32180886	32181054	NOTCH4	693_selected_genes
chr6	32181439	32181642	NOTCH4	693_selected_genes
chr6	32181861	32182057	NOTCH4	693_selected_genes
chr6	32182977	32183187	NOTCH4	693_selected_genes
chr6	32184696	32184869	NOTCH4	693_selected_genes
chr6	32184904	32185068	NOTCH4	693_selected_genes
chr6	32185746	32185910	NOTCH4	693_selected_genes
chr6	32187343	32187588	NOTCH4	693_selected_genes
chr6	32187880	32188086	NOTCH4	693_selected_genes
chr6	32188156	32188443	NOTCH4	693_selected_genes
chr6	32188507	32188680	NOTCH4	693_selected_genes
chr6	32188729	32189127	NOTCH4	693_selected_genes
chr6	32190262	32190608	NOTCH4	693_selected_genes
chr6	32190756	32190888	NOTCH4	693_selected_genes
chr6	32191607	32191730	NOTCH4	693_selected_genes
chr5	170814927	170815035	NPM1	693_selected_genes
chr5	170817029	170817159	NPM1	693_selected_genes
chr5	170818283	170818453	NPM1	693_selected_genes
chr5	170818684	170818828	NPM1	693_selected_genes
chr5	170819688	170819845	NPM1	693_selected_genes
chr5	170819892	170820007	NPM1	693_selected_genes
chr5	170827131	170827239	NPM1	693_selected_genes
chr5	170827817	170827954	NPM1	693_selected_genes
chr5	170832280	170832432	NPM1	693_selected_genes
chr5	170833375	170833434	NPM1	693_selected_genes
chr5	170834678	170834803	NPM1	693_selected_genes
chr5	170837505	170837594	NPM1	693_selected_genes
chr16	69740887	69741135	NQO1	693_selected_genes
chr16	69744853	69745209	NQO1	693_selected_genes
chr16	69746905	69747057	NQO1	693_selected_genes
chr16	69748841	69749005	NQO1	693_selected_genes
chr16	69752000	69752181	NQO1	693_selected_genes
chr16	69752247	69752462	NQO1	693_selected_genes
chr16	69760310	69760367	NQO1	693_selected_genes
chr2	157182230	157182537	NR4A2	693_selected_genes
chr2	157182636	157182865	NR4A2	693_selected_genes
chr2	157183204	157183457	NR4A2	693_selected_genes
chr2	157184337	157184551	NR4A2	693_selected_genes
chr2	157184890	157185070	NR4A2	693_selected_genes
chr2	157185809	157186725	NR4A2	693_selected_genes
chr2	157188966	157189047	NR4A2	693_selected_genes
chr1	115251130	115251300	NRAS	693_selected_genes
chr1	115252164	115252374	NRAS	693_selected_genes
chr1	115256395	115256624	NRAS	693_selected_genes

chr1	115258645	115258806	NRAS	693_selected_genes
chr5	176562079	176563056	NSD1	693_selected_genes
chr5	176563848	176563982	NSD1	693_selected_genes
chr5	176618859	176619045	NSD1	693_selected_genes
chr5	176631095	176631318	NSD1	693_selected_genes
chr5	176636611	176639221	NSD1	693_selected_genes
chr5	176656740	176656900	NSD1	693_selected_genes
chr5	176662796	176662971	NSD1	693_selected_genes
chr5	176665212	176665533	NSD1	693_selected_genes
chr5	176666731	176666891	NSD1	693_selected_genes
chr5	176671170	176671296	NSD1	693_selected_genes
chr5	176673653	176673822	NSD1	693_selected_genes
chr5	176675156	176675350	NSD1	693_selected_genes
chr5	176678705	176678879	NSD1	693_selected_genes
chr5	176683926	176684177	NSD1	693_selected_genes
chr5	176686964	176687194	NSD1	693_selected_genes
chr5	176694537	176694744	NSD1	693_selected_genes
chr5	176696577	176696833	NSD1	693_selected_genes
chr5	176700647	176700810	NSD1	693_selected_genes
chr5	176707540	176707860	NSD1	693_selected_genes
chr5	176709440	176709607	NSD1	693_selected_genes
chr5	176710762	176710954	NSD1	693_selected_genes
chr5	176715794	176715951	NSD1	693_selected_genes
chr5	176718929	176719184	NSD1	693_selected_genes
chr5	176720807	176722485	NSD1	693_selected_genes
chr12	96052836	96053023	NTN4	693_selected_genes
chr12	96059560	96059781	NTN4	693_selected_genes
chr12	96063828	96063947	NTN4	693_selected_genes
chr12	96076457	96076623	NTN4	693_selected_genes
chr12	96077248	96077512	NTN4	693_selected_genes
chr12	96104193	96104432	NTN4	693_selected_genes
chr12	96106964	96107141	NTN4	693_selected_genes
chr12	96131618	96131947	NTN4	693_selected_genes
chr12	96180691	96181271	NTN4	693_selected_genes
chr12	96184007	96184112	NTN4	693_selected_genes
chr1	156785596	156785655	NTRK1	693_selected_genes
chr1	156811847	156812010	NTRK1	693_selected_genes
chr1	156830701	156830963	NTRK1	693_selected_genes
chr1	156834120	156834245	NTRK1	693_selected_genes
chr1	156834494	156834616	NTRK1	693_selected_genes
chr1	156836676	156836795	NTRK1	693_selected_genes
chr1	156837870	156838066	NTRK1	693_selected_genes
chr1	156838271	156838464	NTRK1	693_selected_genes
chr1	156841389	156841572	NTRK1	693_selected_genes
chr1	156843399	156843776	NTRK1	693_selected_genes
chr1	156844149	156844217	NTRK1	693_selected_genes
chr1	156844337	156844443	NTRK1	693_selected_genes
chr1	156844672	156844825	NTRK1	693_selected_genes
chr1	156845286	156845483	NTRK1	693_selected_genes
chr1	156845837	156846027	NTRK1	693_selected_genes
chr1	156846166	156846389	NTRK1	693_selected_genes
chr1	156848888	156849179	NTRK1	693_selected_genes
chr1	156849765	156849974	NTRK1	693_selected_genes
chr1	156851223	156851459	NTRK1	693_selected_genes
chr9	87285638	87285900	NTRK2	693_selected_genes
chr9	87317048	87317173	NTRK2	693_selected_genes
chr9	87317237	87317359	NTRK2	693_selected_genes
chr9	87322733	87322852	NTRK2	693_selected_genes
chr9	87325526	87325731	NTRK2	693_selected_genes
chr9	87338462	87338649	NTRK2	693_selected_genes
chr9	87339113	87339296	NTRK2	693_selected_genes
chr9	87342543	87342899	NTRK2	693_selected_genes
chr9	87356781	87356867	NTRK2	693_selected_genes
chr9	87359862	87360013	NTRK2	693_selected_genes
chr9	87366875	87367025	NTRK2	693_selected_genes
chr9	87425431	87425519	NTRK2	693_selected_genes
chr9	87475929	87476027	NTRK2	693_selected_genes
chr9	87482132	87482371	NTRK2	693_selected_genes
chr9	87486678	87486757	NTRK2	693_selected_genes
chr9	87549051	87549232	NTRK2	693_selected_genes
chr9	87563351	87563574	NTRK2	693_selected_genes
chr9	87570172	87570457	NTRK2	693_selected_genes
chr9	87635095	87635304	NTRK2	693_selected_genes
chr9	87636141	87636377	NTRK2	693_selected_genes
chr15	88420140	88420376	NTRK3	693_selected_genes
chr15	88423475	88423684	NTRK3	693_selected_genes
chr15	88428899	88428991	NTRK3	693_selected_genes
chr15	88459744	88459816	NTRK3	693_selected_genes
chr15	88468775	88468920	NTRK3	693_selected_genes
chr15	88472387	88472690	NTRK3	693_selected_genes
chr15	88476217	88476440	NTRK3	693_selected_genes
chr15	88483828	88484009	NTRK3	693_selected_genes
chr15	88522550	88522719	NTRK3	693_selected_genes
chr15	88524431	88524616	NTRK3	693_selected_genes

chr15	88576062	88576301	NTRK3	693_selected_genes
chr15	88615756	88615855	NTRK3	693_selected_genes
chr15	88669476	88669629	NTRK3	693_selected_genes
chr15	88670367	88670482	NTRK3	693_selected_genes
chr15	88671916	88671990	NTRK3	693_selected_genes
chr15	88678306	88678653	NTRK3	693_selected_genes
chr15	88679104	88679296	NTRK3	693_selected_genes
chr15	88679672	88679865	NTRK3	693_selected_genes
chr15	88680609	88680817	NTRK3	693_selected_genes
chr15	88690540	88690659	NTRK3	693_selected_genes
chr15	88726623	88726745	NTRK3	693_selected_genes
chr15	88727430	88727555	NTRK3	693_selected_genes
chr15	88799111	88799409	NTRK3	693_selected_genes
chr11	71714547	71714609	NUMA1	693_selected_genes
chr11	71714907	71715170	NUMA1	693_selected_genes
chr11	71715245	71715412	NUMA1	693_selected_genes
chr11	71715660	71715887	NUMA1	693_selected_genes
chr11	71716253	71716440	NUMA1	693_selected_genes
chr11	71717055	71717334	NUMA1	693_selected_genes
chr11	71718209	71718506	NUMA1	693_selected_genes
chr11	71719112	71719216	NUMA1	693_selected_genes
chr11	71719632	71719916	NUMA1	693_selected_genes
chr11	71719987	71720256	NUMA1	693_selected_genes
chr11	71720288	71720458	NUMA1	693_selected_genes
chr11	71721806	71721925	NUMA1	693_selected_genes
chr11	71723421	71723513	NUMA1	693_selected_genes
chr11	71723915	71727331	NUMA1	693_selected_genes
chr11	71727428	71727601	NUMA1	693_selected_genes
chr11	71728707	71728898	NUMA1	693_selected_genes
chr11	71729203	71729371	NUMA1	693_selected_genes
chr11	71729416	71729584	NUMA1	693_selected_genes
chr11	71729843	71730051	NUMA1	693_selected_genes
chr11	71730514	71730688	NUMA1	693_selected_genes
chr11	71732224	71732362	NUMA1	693_selected_genes
chr11	71733359	71733490	NUMA1	693_selected_genes
chr11	71734085	71734218	NUMA1	693_selected_genes
chr11	71735294	71735424	NUMA1	693_selected_genes
chr11	71740225	71740361	NUMA1	693_selected_genes
chr11	71746922	71747014	NUMA1	693_selected_genes
chr16	56782134	56782363	NUP93	693_selected_genes
chr16	56792424	56792592	NUP93	693_selected_genes
chr16	56832362	56832475	NUP93	693_selected_genes
chr16	56839390	56839578	NUP93	693_selected_genes
chr16	56852550	56852675	NUP93	693_selected_genes
chr16	56855390	56855530	NUP93	693_selected_genes
chr16	56857593	56857783	NUP93	693_selected_genes
chr16	56862863	56863046	NUP93	693_selected_genes
chr16	56864414	56864622	NUP93	693_selected_genes
chr16	56865728	56865944	NUP93	693_selected_genes
chr16	56866181	56866325	NUP93	693_selected_genes
chr16	56867101	56867343	NUP93	693_selected_genes
chr16	56868014	56868191	NUP93	693_selected_genes
chr16	56868256	56868379	NUP93	693_selected_genes
chr16	56868620	56868715	NUP93	693_selected_genes
chr16	56870487	56870654	NUP93	693_selected_genes
chr16	56871494	56871663	NUP93	693_selected_genes
chr16	56872838	56873030	NUP93	693_selected_genes
chr16	56873407	56873658	NUP93	693_selected_genes
chr16	56875591	56875770	NUP93	693_selected_genes
chr16	56876464	56876632	NUP93	693_selected_genes
chr16	56878385	56878611	NUP93	693_selected_genes
chr11	3692586	3692671	NUP98	693_selected_genes
chr11	3697363	3697631	NUP98	693_selected_genes
chr11	3697713	3697875	NUP98	693_selected_genes
chr11	3697892	3697954	NUP98	693_selected_genes
chr11	3700644	3700702	NUP98	693_selected_genes
chr11	3700758	3700963	NUP98	693_selected_genes
chr11	3704404	3704696	NUP98	693_selected_genes
chr11	3707177	3707449	NUP98	693_selected_genes
chr11	3712551	3712744	NUP98	693_selected_genes
chr11	3714436	3714630	NUP98	693_selected_genes
chr11	3716653	3716861	NUP98	693_selected_genes
chr11	3720286	3720603	NUP98	693_selected_genes
chr11	3721814	3722094	NUP98	693_selected_genes
chr11	3723663	3724147	NUP98	693_selected_genes
chr11	3726404	3726611	NUP98	693_selected_genes
chr11	3727649	3727882	NUP98	693_selected_genes
chr11	3733747	3733983	NUP98	693_selected_genes
chr11	3735022	3735250	NUP98	693_selected_genes
chr11	3740616	3740805	NUP98	693_selected_genes
chr11	3741916	3742080	NUP98	693_selected_genes
chr11	3744361	3744710	NUP98	693_selected_genes
chr11	3746307	3746474	NUP98	693_selected_genes
chr11	3752594	3752833	NUP98	693_selected_genes

chr11	3756395	3756579	NUP98	693_selected_genes
chr11	3765713	3765904	NUP98	693_selected_genes
chr11	3774520	3774663	NUP98	693_selected_genes
chr11	3781660	3781881	NUP98	693_selected_genes
chr11	3784106	3784294	NUP98	693_selected_genes
chr11	3789785	3789999	NUP98	693_selected_genes
chr11	3792952	3793183	NUP98	693_selected_genes
chr11	3794836	3794994	NUP98	693_selected_genes
chr11	3797086	3797276	NUP98	693_selected_genes
chr11	3800077	3800304	NUP98	693_selected_genes
chr11	3800360	3800512	NUP98	693_selected_genes
chr11	3803246	3803372	NUP98	693_selected_genes
chr15	34635808	34635928	NUTM1	693_selected_genes
chr15	34638117	34638261	NUTM1	693_selected_genes
chr15	34640144	34640903	NUTM1	693_selected_genes
chr15	34642879	34643058	NUTM1	693_selected_genes
chr15	34645911	34646098	NUTM1	693_selected_genes
chr15	34646621	34646958	NUTM1	693_selected_genes
chr15	34647196	34647363	NUTM1	693_selected_genes
chr15	34647663	34649717	NUTM1	693_selected_genes
chr11	59210616	59211614	OR5A1	693_selected_genes
chrX	38211924	38212051	OTC	693_selected_genes
chrX	38226518	38226707	OTC	693_selected_genes
chrX	38229023	38229155	OTC	693_selected_genes
chrX	38240569	38240707	OTC	693_selected_genes
chrX	38260502	38260706	OTC	693_selected_genes
chrX	38262845	38263018	OTC	693_selected_genes
chrX	38267969	38268073	OTC	693_selected_genes
chrX	38268103	38268303	OTC	693_selected_genes
chrX	38271089	38271277	OTC	693_selected_genes
chrX	38280250	38280360	OTC	693_selected_genes
chr8	101716500	101716643	PABPC1	693_selected_genes
chr8	101717128	101717309	PABPC1	693_selected_genes
chr8	101717791	101717926	PABPC1	693_selected_genes
chr8	101718853	101719058	PABPC1	693_selected_genes
chr8	101719089	101719250	PABPC1	693_selected_genes
chr8	101721335	101721476	PABPC1	693_selected_genes
chr8	101721661	101721984	PABPC1	693_selected_genes
chr8	101724564	101724710	PABPC1	693_selected_genes
chr8	101724854	101725042	PABPC1	693_selected_genes
chr8	101725289	101725434	PABPC1	693_selected_genes
chr8	101727664	101727854	PABPC1	693_selected_genes
chr8	101729975	101730143	PABPC1	693_selected_genes
chr8	101730289	101730533	PABPC1	693_selected_genes
chr8	101733593	101733836	PABPC1	693_selected_genes
chr11	77034156	77034224	PAK1	693_selected_genes
chr11	77034293	77034430	PAK1	693_selected_genes
chr11	77036347	77036446	PAK1	693_selected_genes
chr11	77040314	77040382	PAK1	693_selected_genes
chr11	77043749	77043937	PAK1	693_selected_genes
chr11	77047105	77047352	PAK1	693_selected_genes
chr11	77048343	77048493	PAK1	693_selected_genes
chr11	77051665	77051833	PAK1	693_selected_genes
chr11	77054838	77055001	PAK1	693_selected_genes
chr11	77058054	77058142	PAK1	693_selected_genes
chr11	77060258	77060357	PAK1	693_selected_genes
chr11	77064555	77064669	PAK1	693_selected_genes
chr11	77066687	77066912	PAK1	693_selected_genes
chr11	77069917	77070087	PAK1	693_selected_genes
chr11	77085347	77085435	PAK1	693_selected_genes
chr11	77090260	77090458	PAK1	693_selected_genes
chr11	77090913	77091064	PAK1	693_selected_genes
chr11	77103350	77103590	PAK1	693_selected_genes
chrX	110366306	110366531	PAK3	693_selected_genes
chrX	110385298	110385449	PAK3	693_selected_genes
chrX	110388062	110388175	PAK3	693_selected_genes
chrX	110389723	110389818	PAK3	693_selected_genes
chrX	110390939	110391143	PAK3	693_selected_genes
chrX	110395612	110395700	PAK3	693_selected_genes
chrX	110406117	110406299	PAK3	693_selected_genes
chrX	110406764	110406980	PAK3	693_selected_genes
chrX	110416220	110416334	PAK3	693_selected_genes
chrX	110435329	110435428	PAK3	693_selected_genes
chrX	110435708	110435871	PAK3	693_selected_genes
chrX	110437503	110437671	PAK3	693_selected_genes
chrX	110439044	110439194	PAK3	693_selected_genes
chrX	110439646	110439893	PAK3	693_selected_genes
chrX	110459623	110459811	PAK3	693_selected_genes
chrX	110463560	110463700	PAK3	693_selected_genes
chr20	9520083	9520289	PAK7	693_selected_genes
chr20	9523207	9523392	PAK7	693_selected_genes
chr20	9524990	9525166	PAK7	693_selected_genes
chr20	9538229	9538406	PAK7	693_selected_genes
chr20	9543512	9543696	PAK7	693_selected_genes

chr20	9546514	9547056	PAK7	693_selected_genes
chr20	9560766	9561602	PAK7	693_selected_genes
chr20	9624747	9625001	PAK7	693_selected_genes
chr16	23614754	23615015	PALB2	693_selected_genes
chr16	23619159	23619358	PALB2	693_selected_genes
chr16	23625299	23625437	PALB2	693_selected_genes
chr16	23632657	23632824	PALB2	693_selected_genes
chr16	23634264	23634476	PALB2	693_selected_genes
chr16	23635304	23635440	PALB2	693_selected_genes
chr16	23637531	23637743	PALB2	693_selected_genes
chr16	23640499	23640621	PALB2	693_selected_genes
chr16	23640935	23641815	PALB2	693_selected_genes
chr16	23646157	23647680	PALB2	693_selected_genes
chr16	23649145	23649298	PALB2	693_selected_genes
chr16	23649365	23649475	PALB2	693_selected_genes
chr16	23649852	23649938	PALB2	693_selected_genes
chr16	23651547	23651651	PALB2	693_selected_genes
chr16	23652405	23652503	PALB2	693_selected_genes
chr6	161771105	161771268	PARK2	693_selected_genes
chr6	161781094	161781262	PARK2	693_selected_genes
chr6	161807800	161807934	PARK2	693_selected_genes
chr6	161966420	161966494	PARK2	693_selected_genes
chr6	161969860	161970060	PARK2	693_selected_genes
chr6	161990361	161990473	PARK2	693_selected_genes
chr6	162206778	162206965	PARK2	693_selected_genes
chr6	162394308	162394474	PARK2	693_selected_genes
chr6	162475097	162475231	PARK2	693_selected_genes
chr6	162622137	162622309	PARK2	693_selected_genes
chr6	162683531	162683822	PARK2	693_selected_genes
chr6	162864316	162864530	PARK2	693_selected_genes
chr6	163148668	163148725	PARK2	693_selected_genes
chr1	226549135	226549267	PARP1	693_selected_genes
chr1	226549644	226549809	PARP1	693_selected_genes
chr1	226550774	226550886	PARP1	693_selected_genes
chr1	226551618	226551796	PARP1	693_selected_genes
chr1	226552677	226552880	PARP1	693_selected_genes
chr1	226553629	226553778	PARP1	693_selected_genes
chr1	226555155	226555334	PARP1	693_selected_genes
chr1	226555874	226556047	PARP1	693_selected_genes
chr1	226558109	226558243	PARP1	693_selected_genes
chr1	226561901	226562080	PARP1	693_selected_genes
chr1	226564783	226565029	PARP1	693_selected_genes
chr1	226566817	226567000	PARP1	693_selected_genes
chr1	226567248	226567367	PARP1	693_selected_genes
chr1	226567597	226567890	PARP1	693_selected_genes
chr1	226568743	226568934	PARP1	693_selected_genes
chr1	226570711	226570909	PARP1	693_selected_genes
chr1	226573179	226573406	PARP1	693_selected_genes
chr1	226574001	226574168	PARP1	693_selected_genes
chr1	226576331	226576481	PARP1	693_selected_genes
chr1	226578085	226578350	PARP1	693_selected_genes
chr1	226579874	226580040	PARP1	693_selected_genes
chr1	226580615	226580706	PARP1	693_selected_genes
chr1	226589707	226590105	PARP1	693_selected_genes
chr1	226595485	226595655	PARP1	693_selected_genes
chr14	20811775	20811871	PARP2	693_selected_genes
chr14	20813065	20813310	PARP2	693_selected_genes
chr14	20813527	20813648	PARP2	693_selected_genes
chr14	20814996	20815097	PARP2	693_selected_genes
chr14	20818659	20818806	PARP2	693_selected_genes
chr14	20819180	20819306	PARP2	693_selected_genes
chr14	20820378	20820531	PARP2	693_selected_genes
chr14	20822218	20822431	PARP2	693_selected_genes
chr14	20822942	20823131	PARP2	693_selected_genes
chr14	20823885	20823999	PARP2	693_selected_genes
chr14	20824030	20824215	PARP2	693_selected_genes
chr14	20824440	20824618	PARP2	693_selected_genes
chr14	20824723	20824873	PARP2	693_selected_genes
chr14	20825184	20825333	PARP2	693_selected_genes
chr14	20825529	20825708	PARP2	693_selected_genes
chr14	20825771	20825981	PARP2	693_selected_genes
chr3	51976680	51976749	PARP3	693_selected_genes
chr3	51977339	51977579	PARP3	693_selected_genes
chr3	51978079	51978258	PARP3	693_selected_genes
chr3	51978380	51978619	PARP3	693_selected_genes
chr3	51978768	51978951	PARP3	693_selected_genes
chr3	51978988	51979265	PARP3	693_selected_genes
chr3	51979485	51979685	PARP3	693_selected_genes
chr3	51979843	51979980	PARP3	693_selected_genes
chr3	51980156	51980384	PARP3	693_selected_genes
chr3	51981730	51981936	PARP3	693_selected_genes
chr3	51982301	51982521	PARP3	693_selected_genes
chr13	24995231	24995477	PARP4	693_selected_genes
chr13	25000578	25000761	PARP4	693_selected_genes

chr13	25005489	25005639	PARP4	693_selected_genes
chr13	25008507	25009637	PARP4	693_selected_genes
chr13	25015958	25016131	PARP4	693_selected_genes
chr13	25016702	25016848	PARP4	693_selected_genes
chr13	25017762	25017893	PARP4	693_selected_genes
chr13	25020793	25020924	PARP4	693_selected_genes
chr13	25021128	25021349	PARP4	693_selected_genes
chr13	25023830	25023980	PARP4	693_selected_genes
chr13	25026518	25026756	PARP4	693_selected_genes
chr13	25027699	25027817	PARP4	693_selected_genes
chr13	25029129	25029375	PARP4	693_selected_genes
chr13	25030453	25030641	PARP4	693_selected_genes
chr13	25033156	25033285	PARP4	693_selected_genes
chr13	25033376	25033473	PARP4	693_selected_genes
chr13	25034084	25034299	PARP4	693_selected_genes
chr13	25043136	25043273	PARP4	693_selected_genes
chr13	25044006	25044188	PARP4	693_selected_genes
chr13	25049584	25049759	PARP4	693_selected_genes
chr13	25051813	25052020	PARP4	693_selected_genes
chr13	25052205	25052439	PARP4	693_selected_genes
chr13	25058765	25058911	PARP4	693_selected_genes
chr13	25060280	25060468	PARP4	693_selected_genes
chr13	25064780	25064991	PARP4	693_selected_genes
chr13	25066533	25066757	PARP4	693_selected_genes
chr13	25067708	25067896	PARP4	693_selected_genes
chr13	25068685	25068885	PARP4	693_selected_genes
chr13	25072228	25072392	PARP4	693_selected_genes
chr13	25073413	25073539	PARP4	693_selected_genes
chr13	25074428	25074545	PARP4	693_selected_genes
chr13	25075745	25075997	PARP4	693_selected_genes
chr13	25077757	25077939	PARP4	693_selected_genes
chr9	36840500	36840658	PAX5	693_selected_genes
chr9	36846814	36846951	PAX5	693_selected_genes
chr9	36881975	36882127	PAX5	693_selected_genes
chr9	36923326	36923506	PAX5	693_selected_genes
chr9	36930859	36930966	PAX5	693_selected_genes
chr9	36966520	36966746	PAX5	693_selected_genes
chr9	37002619	37002798	PAX5	693_selected_genes
chr9	37006444	37006559	PAX5	693_selected_genes
chr9	37014968	37015216	PAX5	693_selected_genes
chr9	37020607	37020823	PAX5	693_selected_genes
chr9	37033957	37034053	PAX5	693_selected_genes
chr2	113976084	113976211	PAX8	693_selected_genes
chr2	113977643	113977780	PAX8	693_selected_genes
chr2	113984650	113984858	PAX8	693_selected_genes
chr2	113992945	113993184	PAX8	693_selected_genes
chr2	113994152	113994323	PAX8	693_selected_genes
chr2	113999102	113999381	PAX8	693_selected_genes
chr2	113999559	113999732	PAX8	693_selected_genes
chr2	114000241	114000380	PAX8	693_selected_genes
chr2	114001978	114002226	PAX8	693_selected_genes
chr2	114004305	114004521	PAX8	693_selected_genes
chr2	114035921	114035996	PAX8	693_selected_genes
chr3	52582053	52582276	PBRM1	693_selected_genes
chr3	52584411	52584678	PBRM1	693_selected_genes
chr3	52584737	52584858	PBRM1	693_selected_genes
chr3	52588714	52588920	PBRM1	693_selected_genes
chr3	52592239	52592454	PBRM1	693_selected_genes
chr3	52595757	52596009	PBRM1	693_selected_genes
chr3	52597273	52597534	PBRM1	693_selected_genes
chr3	52598040	52598274	PBRM1	693_selected_genes
chr3	52610531	52610739	PBRM1	693_selected_genes
chr3	52613044	52613240	PBRM1	693_selected_genes
chr3	52620415	52620729	PBRM1	693_selected_genes
chr3	52621343	52621551	PBRM1	693_selected_genes
chr3	52623060	52623296	PBRM1	693_selected_genes
chr3	52637511	52637773	PBRM1	693_selected_genes
chr3	52643303	52643996	PBRM1	693_selected_genes
chr3	52649341	52649497	PBRM1	693_selected_genes
chr3	52651252	52651579	PBRM1	693_selected_genes
chr3	52658887	52658982	PBRM1	693_selected_genes
chr3	52661263	52661411	PBRM1	693_selected_genes
chr3	52662884	52663076	PBRM1	693_selected_genes
chr3	52668592	52668856	PBRM1	693_selected_genes
chr3	52675944	52676086	PBRM1	693_selected_genes
chr3	52677238	52677384	PBRM1	693_selected_genes
chr3	52678694	52678830	PBRM1	693_selected_genes
chr3	52682334	52682483	PBRM1	693_selected_genes
chr3	52685732	52685851	PBRM1	693_selected_genes
chr3	52692189	52692356	PBRM1	693_selected_genes
chr3	52696123	52696317	PBRM1	693_selected_genes
chr3	52702488	52702686	PBRM1	693_selected_genes
chr3	52712490	52712638	PBRM1	693_selected_genes
chr3	52713564	52713752	PBRM1	693_selected_genes

chr2	70314850	70315971	PCBP1	693_selected_genes
chr1	55505485	55505742	PCSK9	693_selected_genes
chr1	55509490	55509732	PCSK9	693_selected_genes
chr1	55512170	55512344	PCSK9	693_selected_genes
chr1	55516839	55516984	PCSK9	693_selected_genes
chr1	55517925	55518109	PCSK9	693_selected_genes
chr1	55518297	55518489	PCSK9	693_selected_genes
chr1	55521640	55521887	PCSK9	693_selected_genes
chr1	55522978	55523212	PCSK9	693_selected_genes
chr1	55523683	55523907	PCSK9	693_selected_genes
chr1	55524146	55524345	PCSK9	693_selected_genes
chr1	55525133	55525361	PCSK9	693_selected_genes
chr1	55527022	55527254	PCSK9	693_selected_genes
chr1	55529016	55529282	PCSK9	693_selected_genes
chr2	242793184	242793474	PDCD1	693_selected_genes
chr2	242794075	242794160	PDCD1	693_selected_genes
chr2	242794324	242794530	PDCD1	693_selected_genes
chr2	242794747	242795157	PDCD1	693_selected_genes
chr2	242800889	242801015	PDCD1	693_selected_genes
chr4	55106194	55106307	PDGFRA	693_selected_genes
chr4	55124898	55125009	PDGFRA	693_selected_genes
chr4	55127236	55127604	PDGFRA	693_selected_genes
chr4	55129808	55130148	PDGFRA	693_selected_genes
chr4	55131060	55131241	PDGFRA	693_selected_genes
chr4	55133430	55133652	PDGFRA	693_selected_genes
chr4	55133693	55133933	PDGFRA	693_selected_genes
chr4	55136774	55136940	PDGFRA	693_selected_genes
chr4	55138535	55138712	PDGFRA	693_selected_genes
chr4	55139678	55139922	PDGFRA	693_selected_genes
chr4	55140672	55140817	PDGFRA	693_selected_genes
chr4	55140982	55141165	PDGFRA	693_selected_genes
chr4	55143529	55143684	PDGFRA	693_selected_genes
chr4	55144037	55144198	PDGFRA	693_selected_genes
chr4	55144503	55144707	PDGFRA	693_selected_genes
chr4	55144960	55145086	PDGFRA	693_selected_genes
chr4	55146457	55146674	PDGFRA	693_selected_genes
chr4	55151512	55151678	PDGFRA	693_selected_genes
chr4	55151982	55152155	PDGFRA	693_selected_genes
chr4	55153571	55153733	PDGFRA	693_selected_genes
chr4	55154940	55155090	PDGFRA	693_selected_genes
chr4	55155150	55155306	PDGFRA	693_selected_genes
chr4	55156454	55156746	PDGFRA	693_selected_genes
chr4	55161266	55161464	PDGFRA	693_selected_genes
chr5	149495300	149495534	PDGFRB	693_selected_genes
chr5	149497155	149497438	PDGFRB	693_selected_genes
chr5	149498284	149498440	PDGFRB	693_selected_genes
chr5	149499004	149499154	PDGFRB	693_selected_genes
chr5	149499549	149499711	PDGFRB	693_selected_genes
chr5	149500425	149500598	PDGFRB	693_selected_genes
chr5	149500741	149500910	PDGFRB	693_selected_genes
chr5	149501417	149501628	PDGFRB	693_selected_genes
chr5	149502579	149502789	PDGFRB	693_selected_genes
chr5	149503787	149503948	PDGFRB	693_selected_genes
chr5	149504264	149504419	PDGFRB	693_selected_genes
chr5	149504982	149505165	PDGFRB	693_selected_genes
chr5	149506057	149506202	PDGFRB	693_selected_genes
chr5	149509294	149509556	PDGFRB	693_selected_genes
chr5	149510076	149510250	PDGFRB	693_selected_genes
chr5	149511516	149511682	PDGFRB	693_selected_genes
chr5	149512287	149512530	PDGFRB	693_selected_genes
chr5	149513123	149513348	PDGFRB	693_selected_genes
chr5	149513418	149513596	PDGFRB	693_selected_genes
chr5	149514253	149514604	PDGFRB	693_selected_genes
chr5	149515092	149515466	PDGFRB	693_selected_genes
chr5	149516545	149516635	PDGFRB	693_selected_genes
chr2	173420853	173421099	PDK1	693_selected_genes
chr2	173423410	173423602	PDK1	693_selected_genes
chr2	173426922	173427044	PDK1	693_selected_genes
chr2	173427443	173427553	PDK1	693_selected_genes
chr2	173428754	173428811	PDK1	693_selected_genes
chr2	173429205	173429440	PDK1	693_selected_genes
chr2	173429680	173429826	PDK1	693_selected_genes
chr2	173431558	173431686	PDK1	693_selected_genes
chr2	173433443	173433570	PDK1	693_selected_genes
chr2	173435428	173435577	PDK1	693_selected_genes
chr2	173450980	173451141	PDK1	693_selected_genes
chr2	173457637	173457801	PDK1	693_selected_genes
chr2	173460531	173460722	PDK1	693_selected_genes
chr16	2588088	2588162	PDPK1	693_selected_genes
chr16	2588477	2588788	PDPK1	693_selected_genes
chr16	2607678	2607989	PDPK1	693_selected_genes
chr16	2611455	2611548	PDPK1	693_selected_genes
chr16	2611746	2611934	PDPK1	693_selected_genes
chr16	2615528	2615723	PDPK1	693_selected_genes

chr16	2616331	2616483	PDPK1	693_selected_genes
chr16	2627400	2627526	PDPK1	693_selected_genes
chr16	2631270	2631389	PDPK1	693_selected_genes
chr16	2631582	2631729	PDPK1	693_selected_genes
chr16	2633387	2633611	PDPK1	693_selected_genes
chr16	2636651	2636919	PDPK1	693_selected_genes
chr16	2645768	2645876	PDPK1	693_selected_genes
chr16	2647098	2647301	PDPK1	693_selected_genes
chr16	2647626	2647793	PDPK1	693_selected_genes
chrX	133511622	133511810	PHF6	693_selected_genes
chrX	133512009	133512161	PHF6	693_selected_genes
chrX	133527505	133527689	PHF6	693_selected_genes
chrX	133527913	133528007	PHF6	693_selected_genes
chrX	133547492	133547712	PHF6	693_selected_genes
chrX	133547827	133548021	PHF6	693_selected_genes
chrX	133549020	133549277	PHF6	693_selected_genes
chrX	133551173	133551361	PHF6	693_selected_genes
chrX	133559205	133559385	PHF6	693_selected_genes
chr4	41747798	41748364	PHOX2B	693_selected_genes
chr4	41749340	41749578	PHOX2B	693_selected_genes
chr4	41749639	41749749	PHOX2B	693_selected_genes
chr4	41750361	41750652	PHOX2B	693_selected_genes
chr1	204393954	204394193	PIK3C2B	693_selected_genes
chr1	204394715	204394894	PIK3C2B	693_selected_genes
chr1	204396750	204396907	PIK3C2B	693_selected_genes
chr1	204397241	204397373	PIK3C2B	693_selected_genes
chr1	204399023	204399191	PIK3C2B	693_selected_genes
chr1	204400771	204400946	PIK3C2B	693_selected_genes
chr1	204401302	204401554	PIK3C2B	693_selected_genes
chr1	204402418	204402578	PIK3C2B	693_selected_genes
chr1	204402895	204403102	PIK3C2B	693_selected_genes
chr1	204403541	204403761	PIK3C2B	693_selected_genes
chr1	204408037	204408224	PIK3C2B	693_selected_genes
chr1	204409294	204409474	PIK3C2B	693_selected_genes
chr1	204410573	204410716	PIK3C2B	693_selected_genes
chr1	204411628	204411786	PIK3C2B	693_selected_genes
chr1	204412519	204412750	PIK3C2B	693_selected_genes
chr1	204413170	204413315	PIK3C2B	693_selected_genes
chr1	204413433	204413577	PIK3C2B	693_selected_genes
chr1	204415058	204415297	PIK3C2B	693_selected_genes
chr1	204416538	204416731	PIK3C2B	693_selected_genes
chr1	204418287	204418449	PIK3C2B	693_selected_genes
chr1	204418952	204419170	PIK3C2B	693_selected_genes
chr1	204423771	204423944	PIK3C2B	693_selected_genes
chr1	204424958	204425204	PIK3C2B	693_selected_genes
chr1	204426139	204426223	PIK3C2B	693_selected_genes
chr1	204426830	204427027	PIK3C2B	693_selected_genes
chr1	204428980	204429094	PIK3C2B	693_selected_genes
chr1	204429426	204429556	PIK3C2B	693_selected_genes
chr1	204429652	204429814	PIK3C2B	693_selected_genes
chr1	204433114	204433285	PIK3C2B	693_selected_genes
chr1	204433552	204433757	PIK3C2B	693_selected_genes
chr1	204434321	204434472	PIK3C2B	693_selected_genes
chr1	204435873	204436190	PIK3C2B	693_selected_genes
chr1	204437972	204438955	PIK3C2B	693_selected_genes
chr12	18434990	18435718	PIK3C2G	693_selected_genes
chr12	18439755	18439888	PIK3C2G	693_selected_genes
chr12	18443763	18443971	PIK3C2G	693_selected_genes
chr12	18446809	18446974	PIK3C2G	693_selected_genes
chr12	18466870	18467023	PIK3C2G	693_selected_genes
chr12	18473870	18473991	PIK3C2G	693_selected_genes
chr12	18477943	18478057	PIK3C2G	693_selected_genes
chr12	18491334	18491507	PIK3C2G	693_selected_genes
chr12	18496235	18496319	PIK3C2G	693_selected_genes
chr12	18499549	18499795	PIK3C2G	693_selected_genes
chr12	18515672	18515849	PIK3C2G	693_selected_genes
chr12	18524088	18524270	PIK3C2G	693_selected_genes
chr12	18534674	18534839	PIK3C2G	693_selected_genes
chr12	18544030	18544211	PIK3C2G	693_selected_genes
chr12	18552567	18552806	PIK3C2G	693_selected_genes
chr12	18573849	18573993	PIK3C2G	693_selected_genes
chr12	18576853	18576998	PIK3C2G	693_selected_genes
chr12	18641357	18641588	PIK3C2G	693_selected_genes
chr12	18644359	18644517	PIK3C2G	693_selected_genes
chr12	18648970	18649113	PIK3C2G	693_selected_genes
chr12	18650527	18650707	PIK3C2G	693_selected_genes
chr12	18656189	18656376	PIK3C2G	693_selected_genes
chr12	18658200	18658420	PIK3C2G	693_selected_genes
chr12	18691064	18691271	PIK3C2G	693_selected_genes
chr12	18699231	18699391	PIK3C2G	693_selected_genes
chr12	18715611	18715851	PIK3C2G	693_selected_genes
chr12	18716285	18716457	PIK3C2G	693_selected_genes
chr12	18719857	18720016	PIK3C2G	693_selected_genes
chr12	18747402	18747528	PIK3C2G	693_selected_genes

chr12	18762443	18762588	PIK3C2G	693_selected_genes
chr12	18793337	18793513	PIK3C2G	693_selected_genes
chr12	18800784	18800987	PIK3C2G	693_selected_genes
chr18	39535231	39535349	PIK3C3	693_selected_genes
chr18	39535357	39535434	PIK3C3	693_selected_genes
chr18	39537509	39537748	PIK3C3	693_selected_genes
chr18	39542428	39542622	PIK3C3	693_selected_genes
chr18	39550265	39550520	PIK3C3	693_selected_genes
chr18	39564818	39564877	PIK3C3	693_selected_genes
chr18	39567750	39567887	PIK3C3	693_selected_genes
chr18	39570397	39570543	PIK3C3	693_selected_genes
chr18	39573208	39573330	PIK3C3	693_selected_genes
chr18	39575828	39575983	PIK3C3	693_selected_genes
chr18	39576576	39576719	PIK3C3	693_selected_genes
chr18	39584294	39584530	PIK3C3	693_selected_genes
chr18	39593380	39593585	PIK3C3	693_selected_genes
chr18	39595414	39595555	PIK3C3	693_selected_genes
chr18	39600576	39600694	PIK3C3	693_selected_genes
chr18	39607381	39607537	PIK3C3	693_selected_genes
chr18	39609263	39609430	PIK3C3	693_selected_genes
chr18	39613764	39613946	PIK3C3	693_selected_genes
chr18	39617630	39617809	PIK3C3	693_selected_genes
chr18	39618719	39618839	PIK3C3	693_selected_genes
chr18	39620615	39620730	PIK3C3	693_selected_genes
chr18	39623671	39623806	PIK3C3	693_selected_genes
chr18	39629469	39629605	PIK3C3	693_selected_genes
chr18	39629806	39629864	PIK3C3	693_selected_genes
chr18	39637821	39638040	PIK3C3	693_selected_genes
chr18	39639807	39639883	PIK3C3	693_selected_genes
chr18	39644678	39644819	PIK3C3	693_selected_genes
chr18	39647326	39647502	PIK3C3	693_selected_genes
chr18	39661061	39661126	PIK3C3	693_selected_genes
chr3	178916588	178916992	PIK3CA	693_selected_genes
chr3	178917452	178917712	PIK3CA	693_selected_genes
chr3	178919052	178919353	PIK3CA	693_selected_genes
chr3	178921306	178921602	PIK3CA	693_selected_genes
chr3	178922265	178922401	PIK3CA	693_selected_genes
chr3	178927357	178927513	PIK3CA	693_selected_genes
chr3	178927948	178928151	PIK3CA	693_selected_genes
chr3	178928193	178928378	PIK3CA	693_selected_genes
chr3	178935972	178936147	PIK3CA	693_selected_genes
chr3	178936958	178937090	PIK3CA	693_selected_genes
chr3	178937333	178937548	PIK3CA	693_selected_genes
chr3	178937711	178937865	PIK3CA	693_selected_genes
chr3	178938748	178938970	PIK3CA	693_selected_genes
chr3	178941843	178942000	PIK3CA	693_selected_genes
chr3	178942462	178942634	PIK3CA	693_selected_genes
chr3	178943724	178943853	PIK3CA	693_selected_genes
chr3	178947034	178947255	PIK3CA	693_selected_genes
chr3	178947766	178947934	PIK3CA	693_selected_genes
chr3	178947987	178948189	PIK3CA	693_selected_genes
chr3	178951856	178952177	PIK3CA	693_selected_genes
chr3	138374205	138374393	PIK3CB	693_selected_genes
chr3	138374958	138375141	PIK3CB	693_selected_genes
chr3	138376506	138376702	PIK3CB	693_selected_genes
chr3	138382722	138382896	PIK3CB	693_selected_genes
chr3	138383852	138384070	PIK3CB	693_selected_genes
chr3	138400783	138400927	PIK3CB	693_selected_genes
chr3	138402494	138402654	PIK3CB	693_selected_genes
chr3	138403441	138403670	PIK3CB	693_selected_genes
chr3	138407691	138407841	PIK3CB	693_selected_genes
chr3	138409816	138410010	PIK3CB	693_selected_genes
chr3	138413602	138413774	PIK3CB	693_selected_genes
chr3	138417723	138417962	PIK3CB	693_selected_genes
chr3	138423259	138423360	PIK3CB	693_selected_genes
chr3	138425975	138426156	PIK3CB	693_selected_genes
chr3	138431024	138431172	PIK3CB	693_selected_genes
chr3	138433284	138433586	PIK3CB	693_selected_genes
chr3	138452177	138452305	PIK3CB	693_selected_genes
chr3	138453450	138453671	PIK3CB	693_selected_genes
chr3	138456523	138456753	PIK3CB	693_selected_genes
chr3	138461374	138461648	PIK3CB	693_selected_genes
chr3	138474570	138474846	PIK3CB	693_selected_genes
chr3	138477989	138478210	PIK3CB	693_selected_genes
chr1	9770488	9770679	PIK3CD	693_selected_genes
chr1	9775573	9775852	PIK3CD	693_selected_genes
chr1	9775881	9776161	PIK3CD	693_selected_genes
chr1	9776472	9776702	PIK3CD	693_selected_genes
chr1	9776991	9777191	PIK3CD	693_selected_genes
chr1	9777569	9777709	PIK3CD	693_selected_genes
chr1	9778170	9778241	PIK3CD	693_selected_genes
chr1	9778726	9778998	PIK3CD	693_selected_genes
chr1	9779953	9780100	PIK3CD	693_selected_genes
chr1	9780144	9780325	PIK3CD	693_selected_genes

chr1	9780466	9780744	PIK3CD	693_selected_genes
chr1	9780774	9780992	PIK3CD	693_selected_genes
chr1	9781159	9781331	PIK3CD	693_selected_genes
chr1	9781476	9781674	PIK3CD	693_selected_genes
chr1	9781793	9781943	PIK3CD	693_selected_genes
chr1	9782007	9782236	PIK3CD	693_selected_genes
chr1	9782276	9782439	PIK3CD	693_selected_genes
chr1	9782560	9782689	PIK3CD	693_selected_genes
chr1	9783157	9783375	PIK3CD	693_selected_genes
chr1	9784001	9784175	PIK3CD	693_selected_genes
chr1	9784308	9784504	PIK3CD	693_selected_genes
chr1	9784836	9785019	PIK3CD	693_selected_genes
chr1	9786941	9787129	PIK3CD	693_selected_genes
chr7	106507981	106510026	PIK3CG	693_selected_genes
chr7	106512956	106513072	PIK3CG	693_selected_genes
chr7	106513132	106513408	PIK3CG	693_selected_genes
chr7	106515119	106515273	PIK3CG	693_selected_genes
chr7	106519938	106520135	PIK3CG	693_selected_genes
chr7	106522536	106522677	PIK3CG	693_selected_genes
chr7	106523452	106523633	PIK3CG	693_selected_genes
chr7	106524574	106524736	PIK3CG	693_selected_genes
chr7	106526554	106526762	PIK3CG	693_selected_genes
chr7	106545528	106545857	PIK3CG	693_selected_genes
chr5	67522478	67522862	PIK3R1	693_selected_genes
chr5	67535700	67535790	PIK3R1	693_selected_genes
chr5	67569192	67569335	PIK3R1	693_selected_genes
chr5	67569741	67569866	PIK3R1	693_selected_genes
chr5	67575404	67575586	PIK3R1	693_selected_genes
chr5	67576330	67576582	PIK3R1	693_selected_genes
chr5	67576729	67576859	PIK3R1	693_selected_genes
chr5	67584538	67584604	PIK3R1	693_selected_genes
chr5	67586531	67586687	PIK3R1	693_selected_genes
chr5	67588061	67588214	PIK3R1	693_selected_genes
chr5	67588288	67588376	PIK3R1	693_selected_genes
chr5	67588903	67589052	PIK3R1	693_selected_genes
chr5	67589105	67589336	PIK3R1	693_selected_genes
chr5	67589511	67589687	PIK3R1	693_selected_genes
chr5	67590338	67590531	PIK3R1	693_selected_genes
chr5	67590950	67591177	PIK3R1	693_selected_genes
chr5	67591222	67591341	PIK3R1	693_selected_genes
chr5	67591949	67592194	PIK3R1	693_selected_genes
chr5	67593214	67593454	PIK3R1	693_selected_genes
chr19	18266664	18267036	PIK3R2	693_selected_genes
chr19	18271255	18271398	PIK3R2	693_selected_genes
chr19	18271703	18271804	PIK3R2	693_selected_genes
chr19	18271838	18272020	PIK3R2	693_selected_genes
chr19	18272063	18272330	PIK3R2	693_selected_genes
chr19	18272750	18272886	PIK3R2	693_selected_genes
chr19	18272986	18273145	PIK3R2	693_selected_genes
chr19	18273192	18273341	PIK3R2	693_selected_genes
chr19	18273751	18273982	PIK3R2	693_selected_genes
chr19	18274047	18274223	PIK3R2	693_selected_genes
chr19	18276895	18277137	PIK3R2	693_selected_genes
chr19	18277914	18278141	PIK3R2	693_selected_genes
chr19	18279259	18279381	PIK3R2	693_selected_genes
chr19	18279510	18279731	PIK3R2	693_selected_genes
chr19	18279871	18280129	PIK3R2	693_selected_genes
chr6	37138053	37138458	PIM1	693_selected_genes
chr6	37138523	37138680	PIM1	693_selected_genes
chr6	37138731	37138832	PIM1	693_selected_genes
chr6	37138875	37139292	PIM1	693_selected_genes
chr6	37140746	37140973	PIM1	693_selected_genes
chr6	37141684	37141892	PIM1	693_selected_genes
chr1	151170468	151170618	PIP5K1A	693_selected_genes
chr1	151171447	151171617	PIP5K1A	693_selected_genes
chr1	151188412	151188501	PIP5K1A	693_selected_genes
chr1	151196695	151196780	PIP5K1A	693_selected_genes
chr1	151196821	151196907	PIP5K1A	693_selected_genes
chr1	151199770	151199901	PIP5K1A	693_selected_genes
chr1	151204121	151204302	PIP5K1A	693_selected_genes
chr1	151204698	151204866	PIP5K1A	693_selected_genes
chr1	151205001	151205204	PIP5K1A	693_selected_genes
chr1	151206647	151206997	PIP5K1A	693_selected_genes
chr1	151209008	151209264	PIP5K1A	693_selected_genes
chr1	151210632	151210766	PIP5K1A	693_selected_genes
chr1	151211580	151211679	PIP5K1A	693_selected_genes
chr1	151212405	151212540	PIP5K1A	693_selected_genes
chr1	151214573	151214770	PIP5K1A	693_selected_genes
chr1	151214888	151215068	PIP5K1A	693_selected_genes
chr1	151219370	151219466	PIP5K1A	693_selected_genes
chr1	151220313	151220448	PIP5K1A	693_selected_genes
chr12	32945332	32945451	PKP2	693_selected_genes
chr12	32945552	32945690	PKP2	693_selected_genes
chr12	32949017	32949257	PKP2	693_selected_genes

chr12	32955311	32955515	PKP2	693_selected_genes
chr12	32974264	32974488	PKP2	693_selected_genes
chr12	32975375	32975590	PKP2	693_selected_genes
chr12	32976953	32977121	PKP2	693_selected_genes
chr12	32993936	32994164	PKP2	693_selected_genes
chr12	32996090	32996272	PKP2	693_selected_genes
chr12	33003674	33003932	PKP2	693_selected_genes
chr12	33021835	33022021	PKP2	693_selected_genes
chr12	33030754	33031502	PKP2	693_selected_genes
chr12	33031828	33031991	PKP2	693_selected_genes
chr12	33049417	33049690	PKP2	693_selected_genes
chr20	39766256	39766523	PLCG1	693_selected_genes
chr20	39788220	39788423	PLCG1	693_selected_genes
chr20	39788484	39788628	PLCG1	693_selected_genes
chr20	39788720	39788818	PLCG1	693_selected_genes
chr20	39791066	39791201	PLCG1	693_selected_genes
chr20	39791256	39791390	PLCG1	693_selected_genes
chr20	39791572	39791657	PLCG1	693_selected_genes
chr20	39791817	39791940	PLCG1	693_selected_genes
chr20	39791992	39792144	PLCG1	693_selected_genes
chr20	39792329	39792498	PLCG1	693_selected_genes
chr20	39792535	39792671	PLCG1	693_selected_genes
chr20	39792695	39792866	PLCG1	693_selected_genes
chr20	39793547	39793766	PLCG1	693_selected_genes
chr20	39793859	39794032	PLCG1	693_selected_genes
chr20	39794064	39794216	PLCG1	693_selected_genes
chr20	39794253	39794491	PLCG1	693_selected_genes
chr20	39794808	39795059	PLCG1	693_selected_genes
chr20	39795090	39795260	PLCG1	693_selected_genes
chr20	39795293	39795524	PLCG1	693_selected_genes
chr20	39796466	39796594	PLCG1	693_selected_genes
chr20	39797381	39797535	PLCG1	693_selected_genes
chr20	39797693	39797840	PLCG1	693_selected_genes
chr20	39798071	39798191	PLCG1	693_selected_genes
chr20	39798726	39798934	PLCG1	693_selected_genes
chr20	39800807	39800954	PLCG1	693_selected_genes
chr20	39801035	39801310	PLCG1	693_selected_genes
chr20	39801343	39801542	PLCG1	693_selected_genes
chr20	39802034	39802199	PLCG1	693_selected_genes
chr20	39802266	39802478	PLCG1	693_selected_genes
chr20	39802537	39802676	PLCG1	693_selected_genes
chr20	39802738	39802976	PLCG1	693_selected_genes
chr20	39803081	39803174	PLCG1	693_selected_genes
chr20	39809118	39809183	PLCG1	693_selected_genes
chr16	81819569	81819812	PLCG2	693_selected_genes
chr16	81888023	81888217	PLCG2	693_selected_genes
chr16	81891842	81891986	PLCG2	693_selected_genes
chr16	81892695	81892793	PLCG2	693_selected_genes
chr16	81902793	81902928	PLCG2	693_selected_genes
chr16	81904431	81904565	PLCG2	693_selected_genes
chr16	81914489	81914583	PLCG2	693_selected_genes
chr16	81916848	81916971	PLCG2	693_selected_genes
chr16	81922751	81922903	PLCG2	693_selected_genes
chr16	81925051	81925220	PLCG2	693_selected_genes
chr16	81927288	81927424	PLCG2	693_selected_genes
chr16	81929386	81929557	PLCG2	693_selected_genes
chr16	81931455	81931539	PLCG2	693_selected_genes
chr16	81934191	81934410	PLCG2	693_selected_genes
chr16	81938982	81939137	PLCG2	693_selected_genes
chr16	81941264	81941404	PLCG2	693_selected_genes
chr16	81941995	81942221	PLCG2	693_selected_genes
chr16	81944099	81944350	PLCG2	693_selected_genes
chr16	81946176	81946346	PLCG2	693_selected_genes
chr16	81953063	81953294	PLCG2	693_selected_genes
chr16	81954777	81954899	PLCG2	693_selected_genes
chr16	81957064	81957224	PLCG2	693_selected_genes
chr16	81960661	81960808	PLCG2	693_selected_genes
chr16	81962137	81962254	PLCG2	693_selected_genes
chr16	81965076	81965284	PLCG2	693_selected_genes
chr16	81968008	81968161	PLCG2	693_selected_genes
chr16	81969748	81970008	PLCG2	693_selected_genes
chr16	81971337	81971533	PLCG2	693_selected_genes
chr16	81972380	81972545	PLCG2	693_selected_genes
chr16	81973471	81973689	PLCG2	693_selected_genes
chr16	81979754	81979893	PLCG2	693_selected_genes
chr16	81990274	81990509	PLCG2	693_selected_genes
chr16	81991535	81991628	PLCG2	693_selected_genes
chr5	57750384	57750626	PLK2	693_selected_genes
chr5	57750712	57750873	PLK2	693_selected_genes
chr5	57751086	57751266	PLK2	693_selected_genes
chr5	57751340	57751631	PLK2	693_selected_genes
chr5	57751827	57752007	PLK2	693_selected_genes
chr5	57752293	57752441	PLK2	693_selected_genes
chr5	57752746	57752944	PLK2	693_selected_genes

chr5	57752982	57753231	PLK2	693_selected_genes
chr5	57753289	57753435	PLK2	693_selected_genes
chr5	57753885	57754023	PLK2	693_selected_genes
chr5	57754200	57754380	PLK2	693_selected_genes
chr5	57754526	57754693	PLK2	693_selected_genes
chr5	57754786	57754944	PLK2	693_selected_genes
chr5	57755491	57755811	PLK2	693_selected_genes
chr18	57567384	57567492	PMAIP1	693_selected_genes
chr18	57567634	57567835	PMAIP1	693_selected_genes
chr18	57569853	57570105	PMAIP1	693_selected_genes
chr15	74287128	74287307	PML	693_selected_genes
chr15	74290319	74290842	PML	693_selected_genes
chr15	74315143	74315774	PML	693_selected_genes
chr15	74317172	74317293	PML	693_selected_genes
chr15	74324887	74325081	PML	693_selected_genes
chr15	74325471	74325974	PML	693_selected_genes
chr15	74326793	74327022	PML	693_selected_genes
chr15	74327447	74328317	PML	693_selected_genes
chr15	74328322	74328426	PML	693_selected_genes
chr15	74335304	74335546	PML	693_selected_genes
chr15	74336536	74337374	PML	693_selected_genes
chr2	190656510	190656692	PMS1	693_selected_genes
chr2	190660469	190660702	PMS1	693_selected_genes
chr2	190670352	190670633	PMS1	693_selected_genes
chr2	190671123	190671253	PMS1	693_selected_genes
chr2	190682717	190682932	PMS1	693_selected_genes
chr2	190708664	190708831	PMS1	693_selected_genes
chr2	190717355	190717528	PMS1	693_selected_genes
chr2	190718639	190718833	PMS1	693_selected_genes
chr2	190718939	190719879	PMS1	693_selected_genes
chr2	190720529	190720622	PMS1	693_selected_genes
chr2	190722185	190722255	PMS1	693_selected_genes
chr2	190728443	190728979	PMS1	693_selected_genes
chr2	190732499	190732680	PMS1	693_selected_genes
chr2	190738196	190738407	PMS1	693_selected_genes
chr2	190741972	190742187	PMS1	693_selected_genes
chr7	6013004	6013198	PMS2	693_selected_genes
chr7	6017193	6017413	PMS2	693_selected_genes
chr7	6018201	6018352	PMS2	693_selected_genes
chr7	6022429	6022647	PMS2	693_selected_genes
chr7	6026364	6027276	PMS2	693_selected_genes
chr7	6029405	6029611	PMS2	693_selected_genes
chr7	6031578	6031713	PMS2	693_selected_genes
chr7	6035139	6035289	PMS2	693_selected_genes
chr7	6036931	6037079	PMS2	693_selected_genes
chr7	6038713	6038931	PMS2	693_selected_genes
chr7	6042058	6042292	PMS2	693_selected_genes
chr7	6043295	6043448	PMS2	693_selected_genes
chr7	6043577	6043714	PMS2	693_selected_genes
chr7	6045497	6045687	PMS2	693_selected_genes
chr7	6048602	6048675	PMS2	693_selected_genes
chr6	89790588	89791178	PNRC1	693_selected_genes
chr6	89793446	89793940	PNRC1	693_selected_genes
chr19	50902083	50902335	POLD1	693_selected_genes
chr19	50902602	50902766	POLD1	693_selected_genes
chr19	50905009	50905206	POLD1	693_selected_genes
chr19	50905230	50905424	POLD1	693_selected_genes
chr19	50905436	50905655	POLD1	693_selected_genes
chr19	50905685	50905817	POLD1	693_selected_genes
chr19	50905843	50906023	POLD1	693_selected_genes
chr19	50906284	50906501	POLD1	693_selected_genes
chr19	50906724	50906879	POLD1	693_selected_genes
chr19	50909413	50909604	POLD1	693_selected_genes
chr19	50909638	50909799	POLD1	693_selected_genes
chr19	50910214	50910456	POLD1	693_selected_genes
chr19	50910558	50910697	POLD1	693_selected_genes
chr19	50911938	50912183	POLD1	693_selected_genes
chr19	50912353	50912517	POLD1	693_selected_genes
chr19	50912750	50912948	POLD1	693_selected_genes
chr19	50916657	50916803	POLD1	693_selected_genes
chr19	50916973	50917161	POLD1	693_selected_genes
chr19	50918046	50918272	POLD1	693_selected_genes
chr19	50918669	50918884	POLD1	693_selected_genes
chr19	50918955	50919108	POLD1	693_selected_genes
chr19	50919627	50919810	POLD1	693_selected_genes
chr19	50919841	50920005	POLD1	693_selected_genes
chr19	50920276	50920379	POLD1	693_selected_genes
chr19	50920403	50920551	POLD1	693_selected_genes
chr19	50921073	50921229	POLD1	693_selected_genes
chr12	133201257	133201421	POLE	693_selected_genes
chr12	133201465	133201605	POLE	693_selected_genes
chr12	133202205	133202381	POLE	693_selected_genes
chr12	133202677	133202928	POLE	693_selected_genes
chr12	133208875	133209119	POLE	693_selected_genes

chr12	133209224	133209406	POLE	693_selected_genes
chr12	133210746	133210989	POLE	693_selected_genes
chr12	133212452	133212635	POLE	693_selected_genes
chr12	133214574	133214750	POLE	693_selected_genes
chr12	133215685	133215909	POLE	693_selected_genes
chr12	133218207	133218462	POLE	693_selected_genes
chr12	133218737	133219008	POLE	693_selected_genes
chr12	133219066	133219340	POLE	693_selected_genes
chr12	133219380	133219607	POLE	693_selected_genes
chr12	133219784	133219941	POLE	693_selected_genes
chr12	133219967	133220171	POLE	693_selected_genes
chr12	133220397	133220588	POLE	693_selected_genes
chr12	133225487	133225683	POLE	693_selected_genes
chr12	133225866	133226126	POLE	693_selected_genes
chr12	133226237	133226500	POLE	693_selected_genes
chr12	133233696	133233869	POLE	693_selected_genes
chr12	133233909	133234040	POLE	693_selected_genes
chr12	133234428	133234581	POLE	693_selected_genes
chr12	133235855	133236120	POLE	693_selected_genes
chr12	133237529	133237775	POLE	693_selected_genes
chr12	133238087	133238295	POLE	693_selected_genes
chr12	133240564	133240759	POLE	693_selected_genes
chr12	133240930	133241073	POLE	693_selected_genes
chr12	133241862	133242061	POLE	693_selected_genes
chr12	133244063	133244259	POLE	693_selected_genes
chr12	133244916	133245113	POLE	693_selected_genes
chr12	133245195	133245348	POLE	693_selected_genes
chr12	133245371	133245550	POLE	693_selected_genes
chr12	133248775	133248933	POLE	693_selected_genes
chr12	133249187	133249450	POLE	693_selected_genes
chr12	133249724	133249888	POLE	693_selected_genes
chr12	133250135	133250318	POLE	693_selected_genes
chr12	133251958	133252128	POLE	693_selected_genes
chr12	133252295	133252431	POLE	693_selected_genes
chr12	133252654	133252815	POLE	693_selected_genes
chr12	133253106	133253264	POLE	693_selected_genes
chr12	133253923	133254054	POLE	693_selected_genes
chr12	133254138	133254330	POLE	693_selected_genes
chr12	133256057	133256262	POLE	693_selected_genes
chr12	133256514	133256657	POLE	693_selected_genes
chr12	133256738	133256833	POLE	693_selected_genes
chr12	133257167	133257298	POLE	693_selected_genes
chr12	133257698	133257890	POLE	693_selected_genes
chr12	133263814	133263926	POLE	693_selected_genes
chr3	121151125	121151289	POLQ	693_selected_genes
chr3	121151739	121151905	POLQ	693_selected_genes
chr3	121154943	121155147	POLQ	693_selected_genes
chr3	121158813	121158988	POLQ	693_selected_genes
chr3	121168136	121168298	POLQ	693_selected_genes
chr3	121178871	121179106	POLQ	693_selected_genes
chr3	121186340	121186512	POLQ	693_selected_genes
chr3	121187126	121187303	POLQ	693_selected_genes
chr3	121190811	121191036	POLQ	693_selected_genes
chr3	121192171	121192359	POLQ	693_selected_genes
chr3	121195361	121195605	POLQ	693_selected_genes
chr3	121200393	121200684	POLQ	693_selected_genes
chr3	121202207	121202454	POLQ	693_selected_genes
chr3	121203862	121204056	POLQ	693_selected_genes
chr3	121206123	121209280	POLQ	693_selected_genes
chr3	121212299	121212593	POLQ	693_selected_genes
chr3	121215629	121215804	POLQ	693_selected_genes
chr3	121217298	121217542	POLQ	693_selected_genes
chr3	121228382	121228575	POLQ	693_selected_genes
chr3	121228860	121229115	POLQ	693_selected_genes
chr3	121230708	121230901	POLQ	693_selected_genes
chr3	121238692	121238955	POLQ	693_selected_genes
chr3	121240824	121241021	POLQ	693_selected_genes
chr3	121248466	121248664	POLQ	693_selected_genes
chr3	121251811	121252081	POLQ	693_selected_genes
chr3	121255921	121256080	POLQ	693_selected_genes
chr3	121258254	121258461	POLQ	693_selected_genes
chr3	121260170	121260351	POLQ	693_selected_genes
chr3	121263548	121263778	POLQ	693_selected_genes
chr3	121264536	121264749	POLQ	693_selected_genes
chr12	106751632	106751754	POLR3B	693_selected_genes
chr12	106757622	106757705	POLR3B	693_selected_genes
chr12	106760268	106760375	POLR3B	693_selected_genes
chr12	106760410	106760525	POLR3B	693_selected_genes
chr12	106763027	106763153	POLR3B	693_selected_genes
chr12	106763335	106763486	POLR3B	693_selected_genes
chr12	106770111	106770253	POLR3B	693_selected_genes
chr12	106772019	106772187	POLR3B	693_selected_genes
chr12	106773783	106773942	POLR3B	693_selected_genes
chr12	106786783	106786956	POLR3B	693_selected_genes

chr12	106799609	106799779	POLR3B	693_selected_genes
chr12	106804578	106804763	POLR3B	693_selected_genes
chr12	106820949	106821161	POLR3B	693_selected_genes
chr12	106824025	106824276	POLR3B	693_selected_genes
chr12	106826070	106826283	POLR3B	693_selected_genes
chr12	106827471	106827675	POLR3B	693_selected_genes
chr12	106830809	106830934	POLR3B	693_selected_genes
chr12	106831433	106831582	POLR3B	693_selected_genes
chr12	106838215	106838393	POLR3B	693_selected_genes
chr12	106848254	106848514	POLR3B	693_selected_genes
chr12	106850890	106851099	POLR3B	693_selected_genes
chr12	106853003	106853171	POLR3B	693_selected_genes
chr12	106857230	106857423	POLR3B	693_selected_genes
chr12	106889807	106889961	POLR3B	693_selected_genes
chr12	106890504	106890721	POLR3B	693_selected_genes
chr12	106895075	106895239	POLR3B	693_selected_genes
chr12	106897833	106898057	POLR3B	693_selected_genes
chr12	106903172	106903352	POLR3B	693_selected_genes
chr19	42595383	42596117	POU2F2	693_selected_genes
chr19	42596191	42596447	POU2F2	693_selected_genes
chr19	42597893	42598072	POU2F2	693_selected_genes
chr19	42599412	42599639	POU2F2	693_selected_genes
chr19	42599671	42599870	POU2F2	693_selected_genes
chr19	42599914	42600106	POU2F2	693_selected_genes
chr19	42600208	42600420	POU2F2	693_selected_genes
chr19	42603653	42603795	POU2F2	693_selected_genes
chr19	42603842	42603998	POU2F2	693_selected_genes
chr19	42614832	42614933	POU2F2	693_selected_genes
chr19	42620835	42621064	POU2F2	693_selected_genes
chr19	42621376	42621609	POU2F2	693_selected_genes
chr19	42626219	42626359	POU2F2	693_selected_genes
chr19	42626470	42626555	POU2F2	693_selected_genes
chr19	42626637	42626757	POU2F2	693_selected_genes
chr19	42636510	42636588	POU2F2	693_selected_genes
chr17	58677750	58678272	PPM1D	693_selected_genes
chr17	58682041	58682134	PPM1D	693_selected_genes
chr17	58700856	58701135	PPM1D	693_selected_genes
chr17	58711188	58711363	PPM1D	693_selected_genes
chr17	58725227	58725468	PPM1D	693_selected_genes
chr17	58733934	58734227	PPM1D	693_selected_genes
chr17	58734267	58734350	PPM1D	693_selected_genes
chr17	58740330	58740938	PPM1D	693_selected_genes
chr19	52693324	52693572	PPP2R1A	693_selected_genes
chr19	52694272	52694445	PPP2R1A	693_selected_genes
chr19	52703501	52703590	PPP2R1A	693_selected_genes
chr19	52705171	52705312	PPP2R1A	693_selected_genes
chr19	52709190	52709341	PPP2R1A	693_selected_genes
chr19	52714487	52714770	PPP2R1A	693_selected_genes
chr19	52715913	52716111	PPP2R1A	693_selected_genes
chr19	52716182	52716388	PPP2R1A	693_selected_genes
chr19	52719006	52719171	PPP2R1A	693_selected_genes
chr19	52719231	52719352	PPP2R1A	693_selected_genes
chr19	52719756	52719941	PPP2R1A	693_selected_genes
chr19	52722918	52723142	PPP2R1A	693_selected_genes
chr19	52723416	52723527	PPP2R1A	693_selected_genes
chr19	52724206	52724411	PPP2R1A	693_selected_genes
chr19	52725326	52725519	PPP2R1A	693_selected_genes
chr19	52728944	52729097	PPP2R1A	693_selected_genes
chr19	52729192	52729259	PPP2R1A	693_selected_genes
chr9	127911926	127912225	PPP6C	693_selected_genes
chr9	127915786	127916046	PPP6C	693_selected_genes
chr9	127916159	127916289	PPP6C	693_selected_genes
chr9	127920494	127920686	PPP6C	693_selected_genes
chr9	127923094	127923210	PPP6C	693_selected_genes
chr9	127933338	127933484	PPP6C	693_selected_genes
chr9	127934162	127934251	PPP6C	693_selected_genes
chr9	127951172	127951251	PPP6C	693_selected_genes
chr9	127951815	127952022	PPP6C	693_selected_genes
chr6	106534403	106534495	PRDM1	693_selected_genes
chr6	106536050	106536349	PRDM1	693_selected_genes
chr6	106543464	106543634	PRDM1	693_selected_genes
chr6	106546523	106546621	PRDM1	693_selected_genes
chr6	106546935	106546994	PRDM1	693_selected_genes
chr6	106547149	106547452	PRDM1	693_selected_genes
chr6	106552674	106553833	PRDM1	693_selected_genes
chr6	106554220	106554399	PRDM1	693_selected_genes
chr6	106554760	106555386	PRDM1	693_selected_genes
chr7	151254261	151254343	PRKAG2	693_selected_genes
chr7	151257584	151257728	PRKAG2	693_selected_genes
chr7	151261138	151261335	PRKAG2	693_selected_genes
chr7	151262406	151262494	PRKAG2	693_selected_genes
chr7	151262780	151262996	PRKAG2	693_selected_genes
chr7	151263633	151263724	PRKAG2	693_selected_genes
chr7	151265776	151265953	PRKAG2	693_selected_genes

chr7	151267231	151267336	PRKAG2	693_selected_genes
chr7	151269724	151269820	PRKAG2	693_selected_genes
chr7	151271951	151272060	PRKAG2	693_selected_genes
chr7	151273431	151273563	PRKAG2	693_selected_genes
chr7	151292405	151292565	PRKAG2	693_selected_genes
chr7	151328701	151328879	PRKAG2	693_selected_genes
chr7	151329129	151329249	PRKAG2	693_selected_genes
chr7	151372480	151372748	PRKAG2	693_selected_genes
chr7	151432960	151433104	PRKAG2	693_selected_genes
chr7	151478212	151478542	PRKAG2	693_selected_genes
chr7	151483530	151483652	PRKAG2	693_selected_genes
chr7	151573566	151573730	PRKAG2	693_selected_genes
chr17	66511515	66511742	PRKAR1A	693_selected_genes
chr17	66518871	66519092	PRKAR1A	693_selected_genes
chr17	66519829	66519982	PRKAR1A	693_selected_genes
chr17	66520131	66520244	PRKAR1A	693_selected_genes
chr17	66521027	66521124	PRKAR1A	693_selected_genes
chr17	66521869	66522078	PRKAR1A	693_selected_genes
chr17	66523955	66524066	PRKAR1A	693_selected_genes
chr17	66524746	66524810	PRKAR1A	693_selected_genes
chr17	66524985	66525158	PRKAR1A	693_selected_genes
chr17	66526035	66526244	PRKAR1A	693_selected_genes
chr17	66526392	66526615	PRKAR1A	693_selected_genes
chr17	66547199	66547290	PRKAR1A	693_selected_genes
chr8	48686708	48686963	PRKDC	693_selected_genes
chr8	48689379	48689569	PRKDC	693_selected_genes
chr8	48690221	48690460	PRKDC	693_selected_genes
chr8	48690994	48691246	PRKDC	693_selected_genes
chr8	48691263	48691385	PRKDC	693_selected_genes
chr8	48691539	48691679	PRKDC	693_selected_genes
chr8	48694697	48694840	PRKDC	693_selected_genes
chr8	48694913	48695184	PRKDC	693_selected_genes
chr8	48696277	48696395	PRKDC	693_selected_genes
chr8	48697648	48697903	PRKDC	693_selected_genes
chr8	48701441	48701635	PRKDC	693_selected_genes
chr8	48701686	48701824	PRKDC	693_selected_genes
chr8	48706825	48707087	PRKDC	693_selected_genes
chr8	48710772	48710983	PRKDC	693_selected_genes
chr8	48711745	48711976	PRKDC	693_selected_genes
chr8	48713328	48713572	PRKDC	693_selected_genes
chr8	48715841	48716066	PRKDC	693_selected_genes
chr8	48719672	48719912	PRKDC	693_selected_genes
chr8	48729985	48730147	PRKDC	693_selected_genes
chr8	48731937	48732096	PRKDC	693_selected_genes
chr8	48733254	48733529	PRKDC	693_selected_genes
chr8	48734139	48734378	PRKDC	693_selected_genes
chr8	48736393	48736582	PRKDC	693_selected_genes
chr8	48739191	48739447	PRKDC	693_selected_genes
chr8	48740703	48740933	PRKDC	693_selected_genes
chr8	48743140	48743322	PRKDC	693_selected_genes
chr8	48744349	48744512	PRKDC	693_selected_genes
chr8	48746731	48746982	PRKDC	693_selected_genes
chr8	48748873	48749113	PRKDC	693_selected_genes
chr8	48749747	48750005	PRKDC	693_selected_genes
chr8	48751683	48751832	PRKDC	693_selected_genes
chr8	48752551	48752775	PRKDC	693_selected_genes
chr8	48761689	48761889	PRKDC	693_selected_genes
chr8	48761914	48762089	PRKDC	693_selected_genes
chr8	48765208	48765370	PRKDC	693_selected_genes
chr8	48766618	48766800	PRKDC	693_selected_genes
chr8	48767757	48767959	PRKDC	693_selected_genes
chr8	48769691	48769885	PRKDC	693_selected_genes
chr8	48771051	48771221	PRKDC	693_selected_genes
chr8	48771384	48771572	PRKDC	693_selected_genes
chr8	48772146	48772345	PRKDC	693_selected_genes
chr8	48773434	48773557	PRKDC	693_selected_genes
chr8	48774597	48774713	PRKDC	693_selected_genes
chr8	48774908	48775127	PRKDC	693_selected_genes
chr8	48775934	48776163	PRKDC	693_selected_genes
chr8	48777091	48777349	PRKDC	693_selected_genes
chr8	48790259	48790437	PRKDC	693_selected_genes
chr8	48792026	48792244	PRKDC	693_selected_genes
chr8	48793951	48794106	PRKDC	693_selected_genes
chr8	48794447	48794683	PRKDC	693_selected_genes
chr8	48798479	48798733	PRKDC	693_selected_genes
chr8	48800082	48800291	PRKDC	693_selected_genes
chr8	48801053	48801236	PRKDC	693_selected_genes
chr8	48801549	48801808	PRKDC	693_selected_genes
chr8	48802792	48803066	PRKDC	693_selected_genes
chr8	48805674	48805972	PRKDC	693_selected_genes
chr8	48809695	48809879	PRKDC	693_selected_genes
chr8	48811004	48811154	PRKDC	693_selected_genes
chr8	48812907	48813052	PRKDC	693_selected_genes
chr8	48815103	48815380	PRKDC	693_selected_genes

chr8	48817403	48817561	PRKDC	693_selected_genes
chr8	48824944	48825147	PRKDC	693_selected_genes
chr8	48826435	48826649	PRKDC	693_selected_genes
chr8	48827862	48828003	PRKDC	693_selected_genes
chr8	48830811	48830968	PRKDC	693_selected_genes
chr8	48839728	48839938	PRKDC	693_selected_genes
chr8	48840305	48840475	PRKDC	693_selected_genes
chr8	48841626	48841763	PRKDC	693_selected_genes
chr8	48842387	48842597	PRKDC	693_selected_genes
chr8	48843206	48843372	PRKDC	693_selected_genes
chr8	48845554	48845757	PRKDC	693_selected_genes
chr8	48846499	48846675	PRKDC	693_selected_genes
chr8	48847543	48847643	PRKDC	693_selected_genes
chr8	48848266	48848485	PRKDC	693_selected_genes
chr8	48848887	48849102	PRKDC	693_selected_genes
chr8	48852085	48852282	PRKDC	693_selected_genes
chr8	48855743	48855951	PRKDC	693_selected_genes
chr8	48856387	48856468	PRKDC	693_selected_genes
chr8	48856508	48856614	PRKDC	693_selected_genes
chr8	48866154	48866304	PRKDC	693_selected_genes
chr8	48866341	48866504	PRKDC	693_selected_genes
chr8	48866872	48867031	PRKDC	693_selected_genes
chr8	48868408	48868533	PRKDC	693_selected_genes
chr8	48869705	48869848	PRKDC	693_selected_genes
chr8	48869889	48870016	PRKDC	693_selected_genes
chr8	48872507	48872711	PRKDC	693_selected_genes
chr17	1553927	1554275	PRPF8	693_selected_genes
chr17	1554376	1554629	PRPF8	693_selected_genes
chr17	1554682	1554872	PRPF8	693_selected_genes
chr17	1554916	1555107	PRPF8	693_selected_genes
chr17	1556810	1557002	PRPF8	693_selected_genes
chr17	1557045	1557335	PRPF8	693_selected_genes
chr17	1558618	1558862	PRPF8	693_selected_genes
chr17	1559660	1559884	PRPF8	693_selected_genes
chr17	1559916	1560080	PRPF8	693_selected_genes
chr17	1561521	1561700	PRPF8	693_selected_genes
chr17	1561794	1562082	PRPF8	693_selected_genes
chr17	1562625	1562867	PRPF8	693_selected_genes
chr17	1563109	1563320	PRPF8	693_selected_genes
chr17	1563700	1563897	PRPF8	693_selected_genes
chr17	1563966	1564146	PRPF8	693_selected_genes
chr17	1564261	1564481	PRPF8	693_selected_genes
chr17	1564539	1564725	PRPF8	693_selected_genes
chr17	1564879	1565109	PRPF8	693_selected_genes
chr17	1565174	1565472	PRPF8	693_selected_genes
chr17	1576349	1576516	PRPF8	693_selected_genes
chr17	1576625	1576886	PRPF8	693_selected_genes
chr17	1577014	1577211	PRPF8	693_selected_genes
chr17	1577710	1577999	PRPF8	693_selected_genes
chr17	1578420	1578658	PRPF8	693_selected_genes
chr17	1578888	1579131	PRPF8	693_selected_genes
chr17	1579196	1579373	PRPF8	693_selected_genes
chr17	1579475	1579689	PRPF8	693_selected_genes
chr17	1579773	1580030	PRPF8	693_selected_genes
chr17	1580244	1580491	PRPF8	693_selected_genes
chr17	1580833	1581013	PRPF8	693_selected_genes
chr17	1581786	1581971	PRPF8	693_selected_genes
chr17	1582030	1582200	PRPF8	693_selected_genes
chr17	1582285	1582525	PRPF8	693_selected_genes
chr17	1582559	1582729	PRPF8	693_selected_genes
chr17	1582877	1583118	PRPF8	693_selected_genes
chr17	1583994	1584150	PRPF8	693_selected_genes
chr17	1584197	1584373	PRPF8	693_selected_genes
chr17	1584746	1585009	PRPF8	693_selected_genes
chr17	1585088	1585357	PRPF8	693_selected_genes
chr17	1585397	1585612	PRPF8	693_selected_genes
chr17	1586801	1587020	PRPF8	693_selected_genes
chr17	1587740	1587890	PRPF8	693_selected_genes
chr16	31143304	31143681	PRSS8	693_selected_genes
chr16	31143724	31143941	PRSS8	693_selected_genes
chr16	31143977	31144299	PRSS8	693_selected_genes
chr16	31144521	31144734	PRSS8	693_selected_genes
chr16	31146449	31146517	PRSS8	693_selected_genes
chr16	31146709	31146844	PRSS8	693_selected_genes
chr19	40899847	40903902	PRX	693_selected_genes
chr19	40904438	40904748	PRX	693_selected_genes
chr19	40909587	40909794	PRX	693_selected_genes
chr19	40913787	40913864	PRX	693_selected_genes
chr9	15465492	15465603	PSIP1	693_selected_genes
chr9	15466720	15466882	PSIP1	693_selected_genes
chr9	15468602	15468866	PSIP1	693_selected_genes
chr9	15468929	15469081	PSIP1	693_selected_genes
chr9	15469238	15469359	PSIP1	693_selected_genes
chr9	15469910	15470016	PSIP1	693_selected_genes

chr9	15471070	15471254	PSIP1	693_selected_genes
chr9	15471271	15471334	PSIP1	693_selected_genes
chr9	15472604	15472773	PSIP1	693_selected_genes
chr9	15473981	15474260	PSIP1	693_selected_genes
chr9	15478449	15478575	PSIP1	693_selected_genes
chr9	15479563	15479710	PSIP1	693_selected_genes
chr9	15485978	15486091	PSIP1	693_selected_genes
chr9	15486799	15486954	PSIP1	693_selected_genes
chr9	15489958	15490147	PSIP1	693_selected_genes
chr9	15506533	15506660	PSIP1	693_selected_genes
chr9	15510089	15510211	PSIP1	693_selected_genes
chr9	98209168	98209758	PTCH1	693_selected_genes
chr9	98211325	98211630	PTCH1	693_selected_genes
chr9	98212097	98212247	PTCH1	693_selected_genes
chr9	98215734	98215927	PTCH1	693_selected_genes
chr9	98218532	98218720	PTCH1	693_selected_genes
chr9	98220269	98220600	PTCH1	693_selected_genes
chr9	98221856	98222090	PTCH1	693_selected_genes
chr9	98224112	98224305	PTCH1	693_selected_genes
chr9	98229372	98229732	PTCH1	693_selected_genes
chr9	98231007	98231460	PTCH1	693_selected_genes
chr9	98232069	98232238	PTCH1	693_selected_genes
chr9	98236304	98236450	PTCH1	693_selected_genes
chr9	98238290	98238466	PTCH1	693_selected_genes
chr9	98239015	98239164	PTCH1	693_selected_genes
chr9	98239803	98240009	PTCH1	693_selected_genes
chr9	98240311	98240493	PTCH1	693_selected_genes
chr9	98241256	98241454	PTCH1	693_selected_genes
chr9	98242225	98242397	PTCH1	693_selected_genes
chr9	98242646	98242895	PTCH1	693_selected_genes
chr9	98244201	98244347	PTCH1	693_selected_genes
chr9	98244390	98244545	PTCH1	693_selected_genes
chr9	98247941	98248181	PTCH1	693_selected_genes
chr9	98268663	98268906	PTCH1	693_selected_genes
chr9	98270417	98270668	PTCH1	693_selected_genes
chr9	98278725	98278778	PTCH1	693_selected_genes
chr9	98278879	98279127	PTCH1	693_selected_genes
chr10	89623681	89624330	PTEN	693_selected_genes
chr10	89653756	89653891	PTEN	693_selected_genes
chr10	89685244	89685339	PTEN	693_selected_genes
chr10	89690777	89690871	PTEN	693_selected_genes
chr10	89692744	89693033	PTEN	693_selected_genes
chr10	89711849	89712041	PTEN	693_selected_genes
chr10	89717584	89717801	PTEN	693_selected_genes
chr10	89720625	89720900	PTEN	693_selected_genes
chr10	89725018	89725254	PTEN	693_selected_genes
chr1	186643459	186643919	PTGS2	693_selected_genes
chr1	186644355	186644553	PTGS2	693_selected_genes
chr1	186645004	186645341	PTGS2	693_selected_genes
chr1	186645573	186645870	PTGS2	693_selected_genes
chr1	186645939	186646073	PTGS2	693_selected_genes
chr1	186646755	186646987	PTGS2	693_selected_genes
chr1	186647367	186647561	PTGS2	693_selected_genes
chr1	186648164	186648358	PTGS2	693_selected_genes
chr1	186648428	186648595	PTGS2	693_selected_genes
chr1	186649345	186649447	PTGS2	693_selected_genes
chr12	112856890	112856954	PTPN11	693_selected_genes
chr12	112884054	112884227	PTPN11	693_selected_genes
chr12	112888096	112888341	PTPN11	693_selected_genes
chr12	112890973	112891216	PTPN11	693_selected_genes
chr12	112892342	112892509	PTPN11	693_selected_genes
chr12	112893728	112893892	PTPN11	693_selected_genes
chr12	112895069	112895155	PTPN11	693_selected_genes
chr12	112910722	112910869	PTPN11	693_selected_genes
chr12	112915429	112915559	PTPN11	693_selected_genes
chr12	112915635	112915844	PTPN11	693_selected_genes
chr12	112919852	112920034	PTPN11	693_selected_genes
chr12	112924253	112924462	PTPN11	693_selected_genes
chr12	112926221	112926339	PTPN11	693_selected_genes
chr12	112926802	112927004	PTPN11	693_selected_genes
chr12	112939922	112940085	PTPN11	693_selected_genes
chr12	112942473	112942593	PTPN11	693_selected_genes
chr1	198608373	198608496	PTPRC	693_selected_genes
chr1	198661450	198661538	PTPRC	693_selected_genes
chr1	198663121	198663335	PTPRC	693_selected_genes
chr1	198665815	198666063	PTPRC	693_selected_genes
chr1	198668667	198668858	PTPRC	693_selected_genes
chr1	198671490	198671684	PTPRC	693_selected_genes
chr1	198672401	198672526	PTPRC	693_selected_genes
chr1	198673575	198673652	PTPRC	693_selected_genes
chr1	198675837	198676106	PTPRC	693_selected_genes
chr1	198677236	198677415	PTPRC	693_selected_genes
chr1	198678790	198678978	PTPRC	693_selected_genes
chr1	198682056	198682226	PTPRC	693_selected_genes

chr1	198685785	198685994	PTPRC	693_selected_genes
chr1	198687197	198687456	PTPRC	693_selected_genes
chr1	198691519	198691630	PTPRC	693_selected_genes
chr1	198697443	198697602	PTPRC	693_selected_genes
chr1	198698240	198698325	PTPRC	693_selected_genes
chr1	198700720	198700880	PTPRC	693_selected_genes
chr1	198701403	198701544	PTPRC	693_selected_genes
chr1	198701583	198701710	PTPRC	693_selected_genes
chr1	198703299	198703386	PTPRC	693_selected_genes
chr1	198703431	198703579	PTPRC	693_selected_genes
chr1	198704230	198704406	PTPRC	693_selected_genes
chr1	198710972	198711180	PTPRC	693_selected_genes
chr1	198711335	198711521	PTPRC	693_selected_genes
chr1	198713157	198713357	PTPRC	693_selected_genes
chr1	198717212	198717353	PTPRC	693_selected_genes
chr1	198718519	198718703	PTPRC	693_selected_genes
chr1	198719595	198719780	PTPRC	693_selected_genes
chr1	198721352	198721525	PTPRC	693_selected_genes
chr1	198721697	198721926	PTPRC	693_selected_genes
chr1	198723372	198723558	PTPRC	693_selected_genes
chr1	198725009	198725335	PTPRC	693_selected_genes
chr9	8317848	8317967	PTPRD	693_selected_genes
chr9	8319805	8319991	PTPRD	693_selected_genes
chr9	8331556	8331761	PTPRD	693_selected_genes
chr9	8338896	8339072	PTPRD	693_selected_genes
chr9	8340317	8340494	PTPRD	693_selected_genes
chr9	8341064	8341293	PTPRD	693_selected_genes
chr9	8341667	8342003	PTPRD	693_selected_genes
chr9	8375910	8376115	PTPRD	693_selected_genes
chr9	8376581	8376751	PTPRD	693_selected_genes
chr9	8389206	8389432	PTPRD	693_selected_genes
chr9	8404511	8404685	PTPRD	693_selected_genes
chr9	8436566	8436714	PTPRD	693_selected_genes
chr9	8437172	8437264	PTPRD	693_selected_genes
chr9	8449699	8449870	PTPRD	693_selected_genes
chr9	8454554	8454619	PTPRD	693_selected_genes
chr9	8460251	8460305	PTPRD	693_selected_genes
chr9	8460385	8460596	PTPRD	693_selected_genes
chr9	8465440	8465700	PTPRD	693_selected_genes
chr9	8470969	8471110	PTPRD	693_selected_genes
chr9	8484093	8484403	PTPRD	693_selected_genes
chr9	8485201	8485352	PTPRD	693_selected_genes
chr9	8485736	8486374	PTPRD	693_selected_genes
chr9	8492836	8493004	PTPRD	693_selected_genes
chr9	8497216	8497293	PTPRD	693_selected_genes
chr9	8499621	8499865	PTPRD	693_selected_genes
chr9	8500728	8501084	PTPRD	693_selected_genes
chr9	8504235	8504430	PTPRD	693_selected_genes
chr9	8507275	8507459	PTPRD	693_selected_genes
chr9	8517822	8518454	PTPRD	693_selected_genes
chr9	8521251	8521571	PTPRD	693_selected_genes
chr9	8523487	8523549	PTPRD	693_selected_genes
chr9	8524753	8524815	PTPRD	693_selected_genes
chr9	8524899	8525071	PTPRD	693_selected_genes
chr9	8526601	8526669	PTPRD	693_selected_genes
chr9	8527319	8527378	PTPRD	693_selected_genes
chr9	8528399	8528517	PTPRD	693_selected_genes
chr9	8528555	8528804	PTPRD	693_selected_genes
chr9	8633291	8633483	PTPRD	693_selected_genes
chr9	8636673	8636869	PTPRD	693_selected_genes
chr9	8733754	8733868	PTPRD	693_selected_genes
chr19	5206759	5206878	PTPRS	693_selected_genes
chr19	5207907	5208093	PTPRS	693_selected_genes
chr19	5208222	5208427	PTPRS	693_selected_genes
chr19	5210454	5210630	PTPRS	693_selected_genes
chr19	5210664	5210841	PTPRS	693_selected_genes
chr19	5211575	5211804	PTPRS	693_selected_genes
chr19	5211950	5212286	PTPRS	693_selected_genes
chr19	5212322	5212527	PTPRS	693_selected_genes
chr19	5214346	5214516	PTPRS	693_selected_genes
chr19	5214546	5214772	PTPRS	693_selected_genes
chr19	5215274	5215448	PTPRS	693_selected_genes
chr19	5215483	5215631	PTPRS	693_selected_genes
chr19	5216705	5216803	PTPRS	693_selected_genes
chr19	5218405	5218570	PTPRS	693_selected_genes
chr19	5218772	5218834	PTPRS	693_selected_genes
chr19	5219284	5219503	PTPRS	693_selected_genes
chr19	5219924	5220190	PTPRS	693_selected_genes
chr19	5220245	5220389	PTPRS	693_selected_genes
chr19	5220985	5221289	PTPRS	693_selected_genes
chr19	5222108	5222256	PTPRS	693_selected_genes
chr19	5222674	5223333	PTPRS	693_selected_genes
chr19	5225712	5225880	PTPRS	693_selected_genes
chr19	5229301	5229378	PTPRS	693_selected_genes

chr19	5229476	5229720	PTPRS	693_selected_genes
chr19	5231295	5231651	PTPRS	693_selected_genes
chr19	5238904	5239099	PTPRS	693_selected_genes
chr19	5240184	5240368	PTPRS	693_selected_genes
chr19	5243886	5244518	PTPRS	693_selected_genes
chr19	5245761	5246081	PTPRS	693_selected_genes
chr19	5256093	5256155	PTPRS	693_selected_genes
chr19	5258002	5258190	PTPRS	693_selected_genes
chr19	5260790	5260858	PTPRS	693_selected_genes
chr19	5262949	5263016	PTPRS	693_selected_genes
chr19	5264993	5265232	PTPRS	693_selected_genes
chr19	5273304	5273362	PTPRS	693_selected_genes
chr19	5273427	5273619	PTPRS	693_selected_genes
chr19	5274184	5274380	PTPRS	693_selected_genes
chr19	5286035	5286176	PTPRS	693_selected_genes
chr20	40709493	40709597	PTPRT	693_selected_genes
chr20	40710496	40710682	PTPRT	693_selected_genes
chr20	40713296	40713510	PTPRT	693_selected_genes
chr20	40714342	40714518	PTPRT	693_selected_genes
chr20	40727035	40727217	PTPRT	693_selected_genes
chr20	40730738	40730962	PTPRT	693_selected_genes
chr20	40733183	40733383	PTPRT	693_selected_genes
chr20	40735400	40735586	PTPRT	693_selected_genes
chr20	40738947	40739152	PTPRT	693_selected_genes
chr20	40743813	40743980	PTPRT	693_selected_genes
chr20	40744653	40744763	PTPRT	693_selected_genes
chr20	40747017	40747165	PTPRT	693_selected_genes
chr20	40748549	40748636	PTPRT	693_selected_genes
chr20	40757368	40757495	PTPRT	693_selected_genes
chr20	40770529	40770667	PTPRT	693_selected_genes
chr20	40789966	40790207	PTPRT	693_selected_genes
chr20	40827854	40828062	PTPRT	693_selected_genes
chr20	40864843	40864923	PTPRT	693_selected_genes
chr20	40877301	40877487	PTPRT	693_selected_genes
chr20	40899011	40899118	PTPRT	693_selected_genes
chr20	40911103	40911190	PTPRT	693_selected_genes
chr20	40944337	40944661	PTPRT	693_selected_genes
chr20	40979242	40979395	PTPRT	693_selected_genes
chr20	40980698	40980950	PTPRT	693_selected_genes
chr20	41076834	41076994	PTPRT	693_selected_genes
chr20	41100880	41101227	PTPRT	693_selected_genes
chr20	41306480	41306824	PTPRT	693_selected_genes
chr20	41385076	41385301	PTPRT	693_selected_genes
chr20	41400049	41400215	PTPRT	693_selected_genes
chr20	41408832	41408964	PTPRT	693_selected_genes
chr20	41419809	41420131	PTPRT	693_selected_genes
chr20	41514421	41514597	PTPRT	693_selected_genes
chr20	41818260	41818398	PTPRT	693_selected_genes
chr6	163836200	163836392	QKI	693_selected_genes
chr6	163876285	163876478	QKI	693_selected_genes
chr6	163878387	163878443	QKI	693_selected_genes
chr6	163899786	163899953	QKI	693_selected_genes
chr6	163955988	163956182	QKI	693_selected_genes
chr6	163982988	163983126	QKI	693_selected_genes
chr6	163984426	163984802	QKI	693_selected_genes
chr6	163985673	163985767	QKI	693_selected_genes
chr6	163986952	163987028	QKI	693_selected_genes
chr6	163987727	163987852	QKI	693_selected_genes
chr6	163990444	163990541	QKI	693_selected_genes
chr6	163991700	163991767	QKI	693_selected_genes
chr7	6414341	6414426	RAC1	693_selected_genes
chr7	6426817	6426939	RAC1	693_selected_genes
chr7	6431529	6431697	RAC1	693_selected_genes
chr7	6438267	6438374	RAC1	693_selected_genes
chr7	6439731	6439844	RAC1	693_selected_genes
chr7	6441473	6441683	RAC1	693_selected_genes
chr7	6441921	6442102	RAC1	693_selected_genes
chr8	117859713	117859955	RAD21	693_selected_genes
chr8	117861159	117861293	RAD21	693_selected_genes
chr8	117862831	117863031	RAD21	693_selected_genes
chr8	117864161	117864360	RAD21	693_selected_genes
chr8	117864762	117864972	RAD21	693_selected_genes
chr8	117866458	117866732	RAD21	693_selected_genes
chr8	117868379	117868552	RAD21	693_selected_genes
chr8	117868859	117869035	RAD21	693_selected_genes
chr8	117869480	117869737	RAD21	693_selected_genes
chr8	117870565	117870722	RAD21	693_selected_genes
chr8	117874054	117874204	RAD21	693_selected_genes
chr8	117875343	117875523	RAD21	693_selected_genes
chr8	117878799	117878993	RAD21	693_selected_genes
chr5	131892991	131893170	RAD50	693_selected_genes
chr5	131894950	131895084	RAD50	693_selected_genes
chr5	131911443	131911645	RAD50	693_selected_genes
chr5	131914983	131915219	RAD50	693_selected_genes

chr5	131915528	131915783	RAD50	693_selected_genes
chr5	131923228	131923407	RAD50	693_selected_genes
chr5	131923590	131923806	RAD50	693_selected_genes
chr5	131924353	131924597	RAD50	693_selected_genes
chr5	131925297	131925554	RAD50	693_selected_genes
chr5	131926890	131927123	RAD50	693_selected_genes
chr5	131927543	131927751	RAD50	693_selected_genes
chr5	131928430	131928667	RAD50	693_selected_genes
chr5	131930535	131930761	RAD50	693_selected_genes
chr5	131931239	131931527	RAD50	693_selected_genes
chr5	131938966	131939206	RAD50	693_selected_genes
chr5	131939586	131939763	RAD50	693_selected_genes
chr5	131940472	131940716	RAD50	693_selected_genes
chr5	131944281	131944442	RAD50	693_selected_genes
chr5	131944783	131944926	RAD50	693_selected_genes
chr5	131944949	131945113	RAD50	693_selected_genes
chr5	131951669	131951847	RAD50	693_selected_genes
chr5	131953736	131954011	RAD50	693_selected_genes
chr5	131972781	131972917	RAD50	693_selected_genes
chr5	131973747	131973940	RAD50	693_selected_genes
chr5	131976338	131976522	RAD50	693_selected_genes
chr5	131977844	131978081	RAD50	693_selected_genes
chr5	131978671	131978806	RAD50	693_selected_genes
chr15	40990931	40991068	RAD51	693_selected_genes
chr15	40993236	40993424	RAD51	693_selected_genes
chr15	40993978	40994149	RAD51	693_selected_genes
chr15	40998349	40998517	RAD51	693_selected_genes
chr15	41001197	41001339	RAD51	693_selected_genes
chr15	41010977	41011122	RAD51	693_selected_genes
chr15	41020883	41021047	RAD51	693_selected_genes
chr15	41021677	41021857	RAD51	693_selected_genes
chr15	41022025	41022197	RAD51	693_selected_genes
chr15	41023227	41023401	RAD51	693_selected_genes
chr14	68290235	68290369	RAD51B	693_selected_genes
chr14	68292155	68292319	RAD51B	693_selected_genes
chr14	68301771	68301938	RAD51B	693_selected_genes
chr14	68331479	68331541	RAD51B	693_selected_genes
chr14	68331694	68331881	RAD51B	693_selected_genes
chr14	68352560	68352730	RAD51B	693_selected_genes
chr14	68353712	68353946	RAD51B	693_selected_genes
chr14	68758575	68758722	RAD51B	693_selected_genes
chr14	68878115	68878269	RAD51B	693_selected_genes
chr14	68934863	68934992	RAD51B	693_selected_genes
chr14	68937214	68937275	RAD51B	693_selected_genes
chr14	68944339	68944406	RAD51B	693_selected_genes
chr14	68963815	68963882	RAD51B	693_selected_genes
chr14	69061176	69061345	RAD51B	693_selected_genes
chr14	69077697	69077989	RAD51B	693_selected_genes
chr14	69117472	69117587	RAD51B	693_selected_genes
chr17	56769979	56770188	RAD51C	693_selected_genes
chr17	56772266	56772579	RAD51C	693_selected_genes
chr17	56774028	56774245	RAD51C	693_selected_genes
chr17	56780531	56780715	RAD51C	693_selected_genes
chr17	56783824	56783994	RAD51C	693_selected_genes
chr17	56787194	56787376	RAD51C	693_selected_genes
chr17	56798081	56798198	RAD51C	693_selected_genes
chr17	56801375	56801486	RAD51C	693_selected_genes
chr17	56809816	56809945	RAD51C	693_selected_genes
chr17	56811453	56811608	RAD51C	693_selected_genes
chr17	33427946	33428080	RAD51D	693_selected_genes
chr17	33428194	33428409	RAD51D	693_selected_genes
chr17	33430247	33430368	RAD51D	693_selected_genes
chr17	33430447	33430588	RAD51D	693_selected_genes
chr17	33433180	33433293	RAD51D	693_selected_genes
chr17	33433379	33433525	RAD51D	693_selected_genes
chr17	33433981	33434166	RAD51D	693_selected_genes
chr17	33434359	33434491	RAD51D	693_selected_genes
chr17	33443852	33444081	RAD51D	693_selected_genes
chr17	33445494	33445663	RAD51D	693_selected_genes
chr17	33446104	33446216	RAD51D	693_selected_genes
chr17	33446525	33446657	RAD51D	693_selected_genes
chr17	33446693	33446831	RAD51D	693_selected_genes
chr12	1022531	1022643	RAD52	693_selected_genes
chr12	1023034	1023312	RAD52	693_selected_genes
chr12	1023571	1023723	RAD52	693_selected_genes
chr12	1025484	1025726	RAD52	693_selected_genes
chr12	1025779	1026011	RAD52	693_selected_genes
chr12	1034590	1034716	RAD52	693_selected_genes
chr12	1035936	1036137	RAD52	693_selected_genes
chr12	1036285	1036454	RAD52	693_selected_genes
chr12	1038904	1039077	RAD52	693_selected_genes
chr12	1039114	1039335	RAD52	693_selected_genes
chr12	1040360	1040512	RAD52	693_selected_genes
chr12	1042115	1042249	RAD52	693_selected_genes

chr1	46714052	46714105	RAD54L	693_selected_genes
chr1	46714158	46714295	RAD54L	693_selected_genes
chr1	46715646	46715816	RAD54L	693_selected_genes
chr1	46724332	46724443	RAD54L	693_selected_genes
chr1	46725610	46725796	RAD54L	693_selected_genes
chr1	46726188	46726308	RAD54L	693_selected_genes
chr1	46726373	46726712	RAD54L	693_selected_genes
chr1	46726907	46727082	RAD54L	693_selected_genes
chr1	46733105	46733306	RAD54L	693_selected_genes
chr1	46736305	46736482	RAD54L	693_selected_genes
chr1	46738112	46738237	RAD54L	693_selected_genes
chr1	46738318	46738499	RAD54L	693_selected_genes
chr1	46739001	46739162	RAD54L	693_selected_genes
chr1	46739270	46739444	RAD54L	693_selected_genes
chr1	46739784	46739913	RAD54L	693_selected_genes
chr1	46740184	46740414	RAD54L	693_selected_genes
chr1	46743463	46743677	RAD54L	693_selected_genes
chr1	46743718	46743979	RAD54L	693_selected_genes
chr3	12625987	12626181	RAF1	693_selected_genes
chr3	12626320	12626505	RAF1	693_selected_genes
chr3	12626595	12626777	RAF1	693_selected_genes
chr3	12627154	12627323	RAF1	693_selected_genes
chr3	12629064	12629161	RAF1	693_selected_genes
chr3	12632271	12632498	RAF1	693_selected_genes
chr3	12633181	12633316	RAF1	693_selected_genes
chr3	12641164	12641332	RAF1	693_selected_genes
chr3	12641625	12641803	RAF1	693_selected_genes
chr3	12641861	12641939	RAF1	693_selected_genes
chr3	12644951	12645061	RAF1	693_selected_genes
chr3	12645609	12645813	RAF1	693_selected_genes
chr3	12647674	12647823	RAF1	693_selected_genes
chr3	12650239	12650447	RAF1	693_selected_genes
chr3	12650706	12650859	RAF1	693_selected_genes
chr3	12653423	12653586	RAF1	693_selected_genes
chr3	12659988	12660245	RAF1	693_selected_genes
chr2	109336037	109336159	RANBP2	693_selected_genes
chr2	109345562	109345680	RANBP2	693_selected_genes
chr2	109347204	109347366	RANBP2	693_selected_genes
chr2	109347752	109347956	RANBP2	693_selected_genes
chr2	109351962	109352243	RANBP2	693_selected_genes
chr2	109352534	109352730	RANBP2	693_selected_genes
chr2	109356919	109357162	RANBP2	693_selected_genes
chr2	109363141	109363279	RANBP2	693_selected_genes
chr2	109365350	109365610	RANBP2	693_selected_genes
chr2	109367694	109367926	RANBP2	693_selected_genes
chr2	109367958	109368184	RANBP2	693_selected_genes
chr2	109368301	109368475	RANBP2	693_selected_genes
chr2	109369428	109369640	RANBP2	693_selected_genes
chr2	109369856	109370044	RANBP2	693_selected_genes
chr2	109370255	109370452	RANBP2	693_selected_genes
chr2	109371335	109371565	RANBP2	693_selected_genes
chr2	109371606	109371740	RANBP2	693_selected_genes
chr2	109374843	109375029	RANBP2	693_selected_genes
chr2	109378531	109378676	RANBP2	693_selected_genes
chr2	109379667	109384869	RANBP2	693_selected_genes
chr2	109388131	109388352	RANBP2	693_selected_genes
chr2	109388919	109389062	RANBP2	693_selected_genes
chr2	109389298	109389527	RANBP2	693_selected_genes
chr2	109392162	109392417	RANBP2	693_selected_genes
chr2	109393560	109393712	RANBP2	693_selected_genes
chr2	109397699	109397910	RANBP2	693_selected_genes
chr2	109398558	109398882	RANBP2	693_selected_genes
chr2	109398958	109399343	RANBP2	693_selected_genes
chr2	109400026	109400382	RANBP2	693_selected_genes
chr17	38487445	38487673	RARA	693_selected_genes
chr17	38497616	38497892	RARA	693_selected_genes
chr17	38498931	38499144	RARA	693_selected_genes
chr17	38504542	38504741	RARA	693_selected_genes
chr17	38506010	38506202	RARA	693_selected_genes
chr17	38507628	38507808	RARA	693_selected_genes
chr17	38508136	38508347	RARA	693_selected_genes
chr17	38508557	38508784	RARA	693_selected_genes
chr17	38510528	38510783	RARA	693_selected_genes
chr17	38511489	38511698	RARA	693_selected_genes
chr17	38512235	38512503	RARA	693_selected_genes
chr5	86564243	86564832	RASA1	693_selected_genes
chr5	86564839	86564897	RASA1	693_selected_genes
chr5	86565041	86565129	RASA1	693_selected_genes
chr5	86626734	86626825	RASA1	693_selected_genes
chr5	86627139	86627342	RASA1	693_selected_genes
chr5	86628298	86628484	RASA1	693_selected_genes
chr5	86629058	86629179	RASA1	693_selected_genes
chr5	86633765	86633933	RASA1	693_selected_genes
chr5	86637081	86637163	RASA1	693_selected_genes

chr5	86642463	86642566	RASA1	693_selected_genes
chr5	86645005	86645206	RASA1	693_selected_genes
chr5	86648948	86649077	RASA1	693_selected_genes
chr5	86658342	86658513	RASA1	693_selected_genes
chr5	86659139	86659346	RASA1	693_selected_genes
chr5	86662142	86662202	RASA1	693_selected_genes
chr5	86665604	86665742	RASA1	693_selected_genes
chr5	86667909	86668037	RASA1	693_selected_genes
chr5	86669954	86670162	RASA1	693_selected_genes
chr5	86670631	86670758	RASA1	693_selected_genes
chr5	86672184	86672407	RASA1	693_selected_genes
chr5	86672672	86672882	RASA1	693_selected_genes
chr5	86674187	86674380	RASA1	693_selected_genes
chr5	86675526	86675692	RASA1	693_selected_genes
chr5	86676300	86676437	RASA1	693_selected_genes
chr5	86679504	86679622	RASA1	693_selected_genes
chr5	86681092	86681231	RASA1	693_selected_genes
chr5	86682617	86682745	RASA1	693_selected_genes
chr5	86685184	86685369	RASA1	693_selected_genes
chr5	86686591	86686725	RASA1	693_selected_genes
chr13	48878023	48878210	RB1	693_selected_genes
chr13	48881390	48881567	RB1	693_selected_genes
chr13	48916709	48916875	RB1	693_selected_genes
chr13	48919190	48919360	RB1	693_selected_genes
chr13	48921935	48922024	RB1	693_selected_genes
chr13	48923066	48923184	RB1	693_selected_genes
chr13	48934127	48934288	RB1	693_selected_genes
chr13	48936925	48937118	RB1	693_selected_genes
chr13	48939004	48939132	RB1	693_selected_genes
chr13	48941604	48941764	RB1	693_selected_genes
chr13	48942637	48942765	RB1	693_selected_genes
chr13	48947515	48947653	RB1	693_selected_genes
chr13	48951028	48951195	RB1	693_selected_genes
chr13	48953704	48953811	RB1	693_selected_genes
chr13	48954163	48954245	RB1	693_selected_genes
chr13	48954275	48954402	RB1	693_selected_genes
chr13	48955357	48955604	RB1	693_selected_genes
chr13	49027103	49027272	RB1	693_selected_genes
chr13	49030314	49030510	RB1	693_selected_genes
chr13	49033798	49033994	RB1	693_selected_genes
chr13	49037841	49037996	RB1	693_selected_genes
chr13	49039108	49039272	RB1	693_selected_genes
chr13	49039315	49039529	RB1	693_selected_genes
chr13	49047470	49047551	RB1	693_selected_genes
chr13	49050811	49051004	RB1	693_selected_genes
chr13	49051465	49051565	RB1	693_selected_genes
chr13	49054108	49054232	RB1	693_selected_genes
chrX	47004820	47004940	RBM10	693_selected_genes
chrX	47006730	47006922	RBM10	693_selected_genes
chrX	47028688	47028922	RBM10	693_selected_genes
chrX	47030401	47030682	RBM10	693_selected_genes
chrX	47032501	47032621	RBM10	693_selected_genes
chrX	47034392	47034516	RBM10	693_selected_genes
chrX	47035873	47036010	RBM10	693_selected_genes
chrX	47038476	47038587	RBM10	693_selected_genes
chrX	47038692	47038919	RBM10	693_selected_genes
chrX	47039253	47039464	RBM10	693_selected_genes
chrX	47039585	47039733	RBM10	693_selected_genes
chrX	47039792	47039930	RBM10	693_selected_genes
chrX	47040588	47040825	RBM10	693_selected_genes
chrX	47040880	47041070	RBM10	693_selected_genes
chrX	47041122	47041290	RBM10	693_selected_genes
chrX	47041324	47041466	RBM10	693_selected_genes
chrX	47041535	47041750	RBM10	693_selected_genes
chrX	47044428	47044628	RBM10	693_selected_genes
chrX	47044675	47044791	RBM10	693_selected_genes
chrX	47044815	47045054	RBM10	693_selected_genes
chrX	47045089	47045214	RBM10	693_selected_genes
chrX	47045438	47045595	RBM10	693_selected_genes
chrX	47045631	47045811	RBM10	693_selected_genes
chrX	47045847	47046023	RBM10	693_selected_genes
chrX	135954428	135954529	RBMX	693_selected_genes
chrX	135956275	135956636	RBMX	693_selected_genes
chrX	135957193	135957352	RBMX	693_selected_genes
chrX	135957391	135957567	RBMX	693_selected_genes
chrX	135957604	135957769	RBMX	693_selected_genes
chrX	135958636	135958839	RBMX	693_selected_genes
chrX	135960048	135960270	RBMX	693_selected_genes
chrX	135961150	135961342	RBMX	693_selected_genes
chrX	135961452	135961615	RBMX	693_selected_genes
chr8	145736788	145736963	RECQL4	693_selected_genes
chr8	145737038	145737197	RECQL4	693_selected_genes
chr8	145737268	145737475	RECQL4	693_selected_genes
chr8	145737501	145737732	RECQL4	693_selected_genes

chr8	145737749	145737969	RECQL4	693_selected_genes
chr8	145737999	145738179	RECQL4	693_selected_genes
chr8	145738204	145738546	RECQL4	693_selected_genes
chr8	145738575	145738889	RECQL4	693_selected_genes
chr8	145738929	145739121	RECQL4	693_selected_genes
chr8	145739286	145739516	RECQL4	693_selected_genes
chr8	145739547	145739771	RECQL4	693_selected_genes
chr8	145739800	145739934	RECQL4	693_selected_genes
chr8	145740294	145740481	RECQL4	693_selected_genes
chr8	145740508	145740651	RECQL4	693_selected_genes
chr8	145740684	145740866	RECQL4	693_selected_genes
chr8	145741122	145741299	RECQL4	693_selected_genes
chr8	145741346	145742173	RECQL4	693_selected_genes
chr8	145742408	145742599	RECQL4	693_selected_genes
chr8	145742772	145743044	RECQL4	693_selected_genes
chr8	145743059	145743193	RECQL4	693_selected_genes
chr2	61108950	61109010	REL	693_selected_genes
chr2	61118792	61118985	REL	693_selected_genes
chr2	61121506	61121705	REL	693_selected_genes
chr2	61128101	61128243	REL	693_selected_genes
chr2	61143986	61144177	REL	693_selected_genes
chr2	61145300	61145455	REL	693_selected_genes
chr2	61145503	61145766	REL	693_selected_genes
chr2	61147150	61147269	REL	693_selected_genes
chr2	61147492	61147638	REL	693_selected_genes
chr2	61147683	61147802	REL	693_selected_genes
chr2	61148872	61149695	REL	693_selected_genes
chr10	43572681	43572804	RET	693_selected_genes
chr10	43595881	43596195	RET	693_selected_genes
chr10	43597764	43598102	RET	693_selected_genes
chr10	43600374	43600666	RET	693_selected_genes
chr10	43601798	43602044	RET	693_selected_genes
chr10	43604453	43604703	RET	693_selected_genes
chr10	43606629	43606938	RET	693_selected_genes
chr10	43607521	43607697	RET	693_selected_genes
chr10	43608275	43608436	RET	693_selected_genes
chr10	43608978	43609148	RET	693_selected_genes
chr10	43609902	43610209	RET	693_selected_genes
chr10	43612006	43612204	RET	693_selected_genes
chr10	43613795	43613953	RET	693_selected_genes
chr10	43614953	43615218	RET	693_selected_genes
chr10	43615503	43615676	RET	693_selected_genes
chr10	43617368	43617489	RET	693_selected_genes
chr10	43619093	43619281	RET	693_selected_genes
chr10	43620305	43620455	RET	693_selected_genes
chr10	43621997	43622227	RET	693_selected_genes
chr10	43623534	43623742	RET	693_selected_genes
chr1	175914263	175914331	RFWD2	693_selected_genes
chr1	175916305	175916400	RFWD2	693_selected_genes
chr1	175956053	175956264	RFWD2	693_selected_genes
chr1	175957398	175957573	RFWD2	693_selected_genes
chr1	175958472	175958640	RFWD2	693_selected_genes
chr1	175996682	175996849	RFWD2	693_selected_genes
chr1	176012296	176012428	RFWD2	693_selected_genes
chr1	176012820	176012979	RFWD2	693_selected_genes
chr1	176015291	176015485	RFWD2	693_selected_genes
chr1	176050262	176050448	RFWD2	693_selected_genes
chr1	176054886	176055051	RFWD2	693_selected_genes
chr1	176085734	176085842	RFWD2	693_selected_genes
chr1	176102957	176103061	RFWD2	693_selected_genes
chr1	176104120	176104247	RFWD2	693_selected_genes
chr1	176105598	176105708	RFWD2	693_selected_genes
chr1	176118116	176118235	RFWD2	693_selected_genes
chr1	176131979	176132149	RFWD2	693_selected_genes
chr1	176132925	176133052	RFWD2	693_selected_genes
chr1	176137794	176137909	RFWD2	693_selected_genes
chr1	176145020	176145168	RFWD2	693_selected_genes
chr1	176153743	176153853	RFWD2	693_selected_genes
chr1	176175682	176176139	RFWD2	693_selected_genes
chr17	74467689	74468159	RHBDF2	693_selected_genes
chr17	74468191	74468277	RHBDF2	693_selected_genes
chr17	74468742	74468946	RHBDF2	693_selected_genes
chr17	74469061	74469212	RHBDF2	693_selected_genes
chr17	74469308	74469434	RHBDF2	693_selected_genes
chr17	74469704	74469849	RHBDF2	693_selected_genes
chr17	74469895	74470009	RHBDF2	693_selected_genes
chr17	74470089	74470249	RHBDF2	693_selected_genes
chr17	74470429	74470641	RHBDF2	693_selected_genes
chr17	74470786	74470911	RHBDF2	693_selected_genes
chr17	74471086	74471248	RHBDF2	693_selected_genes
chr17	74472886	74473131	RHBDF2	693_selected_genes
chr17	74473236	74473405	RHBDF2	693_selected_genes
chr17	74473713	74473892	RHBDF2	693_selected_genes
chr17	74474862	74475116	RHBDF2	693_selected_genes

chr17	74475138	74475384	RHBDF2	693_selected_genes
chr17	74475789	74476048	RHBDF2	693_selected_genes
chr17	74477431	74477631	RHBDF2	693_selected_genes
chr7	151164179	151164322	RHEB	693_selected_genes
chr7	151167631	151167763	RHEB	693_selected_genes
chr7	151168454	151168552	RHEB	693_selected_genes
chr7	151168609	151168716	RHEB	693_selected_genes
chr7	151174393	151174526	RHEB	693_selected_genes
chr7	151181797	151181915	RHEB	693_selected_genes
chr7	151188003	151188125	RHEB	693_selected_genes
chr7	151195149	151195291	RHEB	693_selected_genes
chr7	151216520	151216622	RHEB	693_selected_genes
chr3	49397616	49397840	RHOA	693_selected_genes
chr3	49398335	49398524	RHOA	693_selected_genes
chr3	49399903	49400084	RHOA	693_selected_genes
chr3	49405835	49406006	RHOA	693_selected_genes
chr3	49410647	49410800	RHOA	693_selected_genes
chr3	49412841	49413047	RHOA	693_selected_genes
chr5	38942380	38942505	RICTOR	693_selected_genes
chr5	38942909	38943098	RICTOR	693_selected_genes
chr5	38944522	38944696	RICTOR	693_selected_genes
chr5	38944989	38945195	RICTOR	693_selected_genes
chr5	38945567	38945851	RICTOR	693_selected_genes
chr5	38946544	38946679	RICTOR	693_selected_genes
chr5	38947340	38947568	RICTOR	693_selected_genes
chr5	38949432	38949554	RICTOR	693_selected_genes
chr5	38949788	38950847	RICTOR	693_selected_genes
chr5	38952272	38952552	RICTOR	693_selected_genes
chr5	38953061	38953218	RICTOR	693_selected_genes
chr5	38953537	38953680	RICTOR	693_selected_genes
chr5	38954850	38954988	RICTOR	693_selected_genes
chr5	38955671	38955831	RICTOR	693_selected_genes
chr5	38957728	38957857	RICTOR	693_selected_genes
chr5	38958519	38958646	RICTOR	693_selected_genes
chr5	38958743	38958958	RICTOR	693_selected_genes
chr5	38959271	38959448	RICTOR	693_selected_genes
chr5	38959855	38960105	RICTOR	693_selected_genes
chr5	38960474	38960660	RICTOR	693_selected_genes
chr5	38962391	38962488	RICTOR	693_selected_genes
chr5	38962561	38962713	RICTOR	693_selected_genes
chr5	38962952	38963168	RICTOR	693_selected_genes
chr5	38964868	38965019	RICTOR	693_selected_genes
chr5	38966717	38966848	RICTOR	693_selected_genes
chr5	38967237	38967354	RICTOR	693_selected_genes
chr5	38967413	38967554	RICTOR	693_selected_genes
chr5	38968019	38968157	RICTOR	693_selected_genes
chr5	38971953	38972086	RICTOR	693_selected_genes
chr5	38975613	38975731	RICTOR	693_selected_genes
chr5	38978659	38978777	RICTOR	693_selected_genes
chr5	38981943	38982163	RICTOR	693_selected_genes
chr5	38991025	38991202	RICTOR	693_selected_genes
chr5	38996895	38997009	RICTOR	693_selected_genes
chr5	39002611	39002793	RICTOR	693_selected_genes
chr5	39003634	39003749	RICTOR	693_selected_genes
chr5	39021115	39021263	RICTOR	693_selected_genes
chr5	39074187	39074285	RICTOR	693_selected_genes
chr5	39074405	39074504	RICTOR	693_selected_genes
chr1	155870153	155870434	RIT1	693_selected_genes
chr1	155874076	155874318	RIT1	693_selected_genes
chr1	155874368	155874426	RIT1	693_selected_genes
chr1	155874496	155874620	RIT1	693_selected_genes
chr1	155880215	155880322	RIT1	693_selected_genes
chr1	155880421	155880620	RIT1	693_selected_genes
chr1	155880643	155880701	RIT1	693_selected_genes
chr17	56432278	56432372	RNF43	693_selected_genes
chr17	56434501	56436209	RNF43	693_selected_genes
chr17	56437484	56437637	RNF43	693_selected_genes
chr17	56438118	56438330	RNF43	693_selected_genes
chr17	56439879	56440034	RNF43	693_selected_genes
chr17	56440610	56440792	RNF43	693_selected_genes
chr17	56440861	56440986	RNF43	693_selected_genes
chr17	56448246	56448419	RNF43	693_selected_genes
chr17	56492661	56492963	RNF43	693_selected_genes
chr6	117609629	117609990	ROS1	693_selected_genes
chr6	117622111	117622325	ROS1	693_selected_genes
chr6	117629931	117630116	ROS1	693_selected_genes
chr6	117631218	117631469	ROS1	693_selected_genes
chr6	117632157	117632305	ROS1	693_selected_genes
chr6	117638280	117638460	ROS1	693_selected_genes
chr6	117639325	117639440	ROS1	693_selected_genes
chr6	117641005	117641218	ROS1	693_selected_genes
chr6	117642396	117642582	ROS1	693_selected_genes
chr6	117645469	117645603	ROS1	693_selected_genes
chr6	117647361	117647602	ROS1	693_selected_genes

chr6	117649944	117650001	ROS1	693_selected_genes
chr6	117650466	117650634	ROS1	693_selected_genes
chr6	117658309	117658528	ROS1	693_selected_genes
chr6	117662272	117662499	ROS1	693_selected_genes
chr6	117662537	117662820	ROS1	693_selected_genes
chr6	117663537	117663732	ROS1	693_selected_genes
chr6	117665197	117665450	ROS1	693_selected_genes
chr6	117674127	117674357	ROS1	693_selected_genes
chr6	117677766	117678103	ROS1	693_selected_genes
chr6	117678941	117679197	ROS1	693_selected_genes
chr6	117680946	117681199	ROS1	693_selected_genes
chr6	117681479	117681593	ROS1	693_selected_genes
chr6	117683740	117684053	ROS1	693_selected_genes
chr6	117686197	117686392	ROS1	693_selected_genes
chr6	117686718	117686929	ROS1	693_selected_genes
chr6	117687213	117687478	ROS1	693_selected_genes
chr6	117700196	117700347	ROS1	693_selected_genes
chr6	117704454	117704696	ROS1	693_selected_genes
chr6	117706820	117707049	ROS1	693_selected_genes
chr6	117708026	117708187	ROS1	693_selected_genes
chr6	117708917	117709222	ROS1	693_selected_genes
chr6	117710487	117711034	ROS1	693_selected_genes
chr6	117714361	117714509	ROS1	693_selected_genes
chr6	117715299	117715534	ROS1	693_selected_genes
chr6	117715753	117715926	ROS1	693_selected_genes
chr6	117717325	117717452	ROS1	693_selected_genes
chr6	117718052	117718304	ROS1	693_selected_genes
chr6	117724276	117724465	ROS1	693_selected_genes
chr6	117725417	117725616	ROS1	693_selected_genes
chr6	117730719	117730830	ROS1	693_selected_genes
chr6	117735656	117735733	ROS1	693_selected_genes
chr6	117737395	117737505	ROS1	693_selected_genes
chr6	117739599	117739694	ROS1	693_selected_genes
chr6	117746671	117746844	ROS1	693_selected_genes
chr17	1733362	1733445	RPA1	693_selected_genes
chr17	1746071	1746172	RPA1	693_selected_genes
chr17	1747188	1747317	RPA1	693_selected_genes
chr17	1747846	1748005	RPA1	693_selected_genes
chr17	1756369	1756508	RPA1	693_selected_genes
chr17	1775702	1775845	RPA1	693_selected_genes
chr17	1778929	1779112	RPA1	693_selected_genes
chr17	1780480	1780633	RPA1	693_selected_genes
chr17	1782261	1782380	RPA1	693_selected_genes
chr17	1782483	1782726	RPA1	693_selected_genes
chr17	1782828	1783018	RPA1	693_selected_genes
chr17	1783811	1784010	RPA1	693_selected_genes
chr17	1787080	1787263	RPA1	693_selected_genes
chr17	1791943	1792170	RPA1	693_selected_genes
chr17	1795101	1795259	RPA1	693_selected_genes
chr17	1798277	1798414	RPA1	693_selected_genes
chr17	1800339	1800494	RPA1	693_selected_genes
chrX	38128853	38129110	RPGR	693_selected_genes
chrX	38132613	38132755	RPGR	693_selected_genes
chrX	38134316	38134424	RPGR	693_selected_genes
chrX	38135814	38136050	RPGR	693_selected_genes
chrX	38138397	38138483	RPGR	693_selected_genes
chrX	38144767	38147319	RPGR	693_selected_genes
chrX	38150186	38150302	RPGR	693_selected_genes
chrX	38150620	38150762	RPGR	693_selected_genes
chrX	38156511	38156730	RPGR	693_selected_genes
chrX	38157597	38157725	RPGR	693_selected_genes
chrX	38158183	38158419	RPGR	693_selected_genes
chrX	38160474	38160649	RPGR	693_selected_genes
chrX	38163862	38164068	RPGR	693_selected_genes
chrX	38169842	38170051	RPGR	693_selected_genes
chrX	38176543	38176743	RPGR	693_selected_genes
chrX	38178056	38178265	RPGR	693_selected_genes
chrX	38180254	38180367	RPGR	693_selected_genes
chrX	38182080	38182223	RPGR	693_selected_genes
chrX	38182626	38182802	RPGR	693_selected_genes
chrX	38186567	38186645	RPGR	693_selected_genes
chr1	6241502	6241571	RPL22	693_selected_genes
chr1	6246706	6246901	RPL22	693_selected_genes
chr1	6252961	6253139	RPL22	693_selected_genes
chr1	6257686	6257841	RPL22	693_selected_genes
chr1	6259596	6259658	RPL22	693_selected_genes
chr1	93297646	93297699	RPL5	693_selected_genes
chr1	93298920	93299040	RPL5	693_selected_genes
chr1	93299076	93299242	RPL5	693_selected_genes
chr1	93299327	93299380	RPL5	693_selected_genes
chr1	93300310	93300495	RPL5	693_selected_genes
chr1	93301721	93301978	RPL5	693_selected_genes
chr1	93302987	93303215	RPL5	693_selected_genes
chr1	93306082	93306221	RPL5	693_selected_genes

chr1	93307297	93307447	RPL5	693_selected_genes
chr19	1438399	1438686	RPS15	693_selected_genes
chr19	1438780	1438916	RPS15	693_selected_genes
chr19	1439941	1440486	RPS15	693_selected_genes
chr11	64126682	64126787	RPS6KA4	693_selected_genes
chr11	64126824	64126946	RPS6KA4	693_selected_genes
chr11	64127609	64127878	RPS6KA4	693_selected_genes
chr11	64127923	64128089	RPS6KA4	693_selected_genes
chr11	64128580	64128738	RPS6KA4	693_selected_genes
chr11	64128915	64129046	RPS6KA4	693_selected_genes
chr11	64129088	64129242	RPS6KA4	693_selected_genes
chr11	64129298	64129499	RPS6KA4	693_selected_genes
chr11	64132747	64132962	RPS6KA4	693_selected_genes
chr11	64135578	64135757	RPS6KA4	693_selected_genes
chr11	64135914	64136098	RPS6KA4	693_selected_genes
chr11	64136150	64136294	RPS6KA4	693_selected_genes
chr11	64136892	64137116	RPS6KA4	693_selected_genes
chr11	64137145	64137390	RPS6KA4	693_selected_genes
chr11	64137671	64137881	RPS6KA4	693_selected_genes
chr11	64138009	64138223	RPS6KA4	693_selected_genes
chr11	64138729	64138977	RPS6KA4	693_selected_genes
chr11	67195991	67196119	RPS6KB2	693_selected_genes
chr11	67196358	67196518	RPS6KB2	693_selected_genes
chr11	67196565	67196736	RPS6KB2	693_selected_genes
chr11	67196972	67197091	RPS6KB2	693_selected_genes
chr11	67198813	67199011	RPS6KB2	693_selected_genes
chr11	67199798	67199856	RPS6KB2	693_selected_genes
chr11	67200045	67200153	RPS6KB2	693_selected_genes
chr11	67200182	67200333	RPS6KB2	693_selected_genes
chr11	67200397	67200538	RPS6KB2	693_selected_genes
chr11	67200571	67200712	RPS6KB2	693_selected_genes
chr11	67200785	67200943	RPS6KB2	693_selected_genes
chr11	67201440	67201553	RPS6KB2	693_selected_genes
chr11	67201643	67201771	RPS6KB2	693_selected_genes
chr11	67201822	67201980	RPS6KB2	693_selected_genes
chr11	67202027	67202190	RPS6KB2	693_selected_genes
chr11	67202434	67202665	RPS6KB2	693_selected_genes
chr17	78519404	78519616	RPTOR	693_selected_genes
chr17	78599465	78599618	RPTOR	693_selected_genes
chr17	78617502	78617635	RPTOR	693_selected_genes
chr17	78681615	78681824	RPTOR	693_selected_genes
chr17	78704334	78704531	RPTOR	693_selected_genes
chr17	78727784	78728010	RPTOR	693_selected_genes
chr17	78765224	78765334	RPTOR	693_selected_genes
chr17	78795975	78796126	RPTOR	693_selected_genes
chr17	78796853	78797052	RPTOR	693_selected_genes
chr17	78811696	78811822	RPTOR	693_selected_genes
chr17	78820247	78820399	RPTOR	693_selected_genes
chr17	78829238	78829372	RPTOR	693_selected_genes
chr17	78831564	78831761	RPTOR	693_selected_genes
chr17	78854189	78854314	RPTOR	693_selected_genes
chr17	78857193	78857309	RPTOR	693_selected_genes
chr17	78857555	78857797	RPTOR	693_selected_genes
chr17	78858782	78858973	RPTOR	693_selected_genes
chr17	78865494	78865662	RPTOR	693_selected_genes
chr17	78866503	78866694	RPTOR	693_selected_genes
chr17	78867481	78867690	RPTOR	693_selected_genes
chr17	78882585	78882754	RPTOR	693_selected_genes
chr17	78896498	78896652	RPTOR	693_selected_genes
chr17	78897264	78897918	RPTOR	693_selected_genes
chr17	78899144	78899305	RPTOR	693_selected_genes
chr17	78914270	78914426	RPTOR	693_selected_genes
chr17	78919441	78919606	RPTOR	693_selected_genes
chr17	78921001	78921176	RPTOR	693_selected_genes
chr17	78923217	78923372	RPTOR	693_selected_genes
chr17	78931398	78931555	RPTOR	693_selected_genes
chr17	78933852	78934030	RPTOR	693_selected_genes
chr17	78935168	78935305	RPTOR	693_selected_genes
chr17	78936235	78936402	RPTOR	693_selected_genes
chr17	78936702	78936882	RPTOR	693_selected_genes
chr17	78938036	78938155	RPTOR	693_selected_genes
chr21	36164406	36164932	RUNX1	693_selected_genes
chr21	36171572	36171784	RUNX1	693_selected_genes
chr21	36193939	36194018	RUNX1	693_selected_genes
chr21	36206681	36206923	RUNX1	693_selected_genes
chr21	36228684	36228769	RUNX1	693_selected_genes
chr21	36231745	36231900	RUNX1	693_selected_genes
chr21	36252828	36253035	RUNX1	693_selected_genes
chr21	36259114	36259434	RUNX1	693_selected_genes
chr21	36261948	36262059	RUNX1	693_selected_genes
chr21	36265196	36265285	RUNX1	693_selected_genes
chr21	36421113	36421221	RUNX1	693_selected_genes
chr8	92972444	92972770	RUNX1T1	693_selected_genes
chr8	92982860	92983101	RUNX1T1	693_selected_genes

chr8	92988107	92988226	RUNX1T1	693_selected_genes
chr8	92992129	92992184	RUNX1T1	693_selected_genes
chr8	92998326	92998578	RUNX1T1	693_selected_genes
chr8	92999089	92999225	RUNX1T1	693_selected_genes
chr8	93003841	93004142	RUNX1T1	693_selected_genes
chr8	93006793	93006876	RUNX1T1	693_selected_genes
chr8	93015532	93015633	RUNX1T1	693_selected_genes
chr8	93017314	93017550	RUNX1T1	693_selected_genes
chr8	93023204	93023344	RUNX1T1	693_selected_genes
chr8	93026781	93027073	RUNX1T1	693_selected_genes
chr8	93029428	93029684	RUNX1T1	693_selected_genes
chr8	93074748	93074962	RUNX1T1	693_selected_genes
chr8	93088167	93088305	RUNX1T1	693_selected_genes
chr8	93107230	93107424	RUNX1T1	693_selected_genes
chr8	93107549	93107720	RUNX1T1	693_selected_genes
chr8	93115059	93115137	RUNX1T1	693_selected_genes
chr1	25228587	25229182	RUNX3	693_selected_genes
chr1	25233724	25233933	RUNX3	693_selected_genes
chr1	25245705	25245860	RUNX3	693_selected_genes
chr1	25254039	25254246	RUNX3	693_selected_genes
chr1	25255505	25255558	RUNX3	693_selected_genes
chr1	25256052	25256384	RUNX3	693_selected_genes
chr1	25290979	25291087	RUNX3	693_selected_genes
chr9	137218452	137218530	RXRA	693_selected_genes
chr9	137293452	137293753	RXRA	693_selected_genes
chr9	137299969	137300170	RXRA	693_selected_genes
chr9	137300760	137300990	RXRA	693_selected_genes
chr9	137308978	137309198	RXRA	693_selected_genes
chr9	137313496	137313676	RXRA	693_selected_genes
chr9	137320928	137321111	RXRA	693_selected_genes
chr9	137323725	137323867	RXRA	693_selected_genes
chr9	137325922	137326078	RXRA	693_selected_genes
chr9	137328287	137328485	RXRA	693_selected_genes
chr3	72427510	72427813	RYBP	693_selected_genes
chr3	72428165	72428314	RYBP	693_selected_genes
chr3	72428376	72428603	RYBP	693_selected_genes
chr3	72495621	72496094	RYBP	693_selected_genes
chr19	38924444	38924539	RYR1	693_selected_genes
chr19	38931359	38931529	RYR1	693_selected_genes
chr19	38932963	38933118	RYR1	693_selected_genes
chr19	38934172	38934297	RYR1	693_selected_genes
chr19	38934332	38934461	RYR1	693_selected_genes
chr19	38934763	38934926	RYR1	693_selected_genes
chr19	38935198	38935342	RYR1	693_selected_genes
chr19	38937086	38937230	RYR1	693_selected_genes
chr19	38937308	38937433	RYR1	693_selected_genes
chr19	38938969	38939176	RYR1	693_selected_genes
chr19	38939263	38939478	RYR1	693_selected_genes
chr19	38942378	38942550	RYR1	693_selected_genes
chr19	38943433	38943679	RYR1	693_selected_genes
chr19	38945849	38946035	RYR1	693_selected_genes
chr19	38946065	38946211	RYR1	693_selected_genes
chr19	38946247	38946416	RYR1	693_selected_genes
chr19	38948111	38948295	RYR1	693_selected_genes
chr19	38948665	38948957	RYR1	693_selected_genes
chr19	38949760	38950003	RYR1	693_selected_genes
chr19	38950989	38951256	RYR1	693_selected_genes
chr19	38954037	38954192	RYR1	693_selected_genes
chr19	38954361	38954515	RYR1	693_selected_genes
chr19	38955253	38955387	RYR1	693_selected_genes
chr19	38956705	38957063	RYR1	693_selected_genes
chr19	38958224	38958477	RYR1	693_selected_genes
chr19	38959580	38959805	RYR1	693_selected_genes
chr19	38959919	38960178	RYR1	693_selected_genes
chr19	38963991	38964436	RYR1	693_selected_genes
chr19	38965932	38966115	RYR1	693_selected_genes
chr19	38968324	38968535	RYR1	693_selected_genes
chr19	38969049	38969265	RYR1	693_selected_genes
chr19	38973641	38973778	RYR1	693_selected_genes
chr19	38973904	38974181	RYR1	693_selected_genes
chr19	38976204	38976867	RYR1	693_selected_genes
chr19	38979791	38980108	RYR1	693_selected_genes
chr19	38980690	38980941	RYR1	693_selected_genes
chr19	38981235	38981397	RYR1	693_selected_genes
chr19	38983104	38983301	RYR1	693_selected_genes
chr19	38984966	38985290	RYR1	693_selected_genes
chr19	38986829	38986994	RYR1	693_selected_genes
chr19	38987023	38987206	RYR1	693_selected_genes
chr19	38987474	38987619	RYR1	693_selected_genes
chr19	38989722	38989908	RYR1	693_selected_genes
chr19	38990249	38990486	RYR1	693_selected_genes
chr19	38990522	38990681	RYR1	693_selected_genes
chr19	38991220	38991391	RYR1	693_selected_genes
chr19	38991435	38991655	RYR1	693_selected_genes

chr19	38993121	38993392	RYR1	693_selected_genes
chr19	38993494	38993635	RYR1	693_selected_genes
chr19	38994834	38995025	RYR1	693_selected_genes
chr19	38995362	38995576	RYR1	693_selected_genes
chr19	38995617	38995746	RYR1	693_selected_genes
chr19	38995923	38996063	RYR1	693_selected_genes
chr19	38996420	38996611	RYR1	693_selected_genes
chr19	38996917	38997042	RYR1	693_selected_genes
chr19	38997085	38997211	RYR1	693_selected_genes
chr19	38997443	38997617	RYR1	693_selected_genes
chr19	38998326	38998492	RYR1	693_selected_genes
chr19	39001112	39001230	RYR1	693_selected_genes
chr19	39001274	39001446	RYR1	693_selected_genes
chr19	39002175	39002275	RYR1	693_selected_genes
chr19	39002686	39002797	RYR1	693_selected_genes
chr19	39002859	39003148	RYR1	693_selected_genes
chr19	39005640	39005772	RYR1	693_selected_genes
chr19	39006701	39006882	RYR1	693_selected_genes
chr19	39007973	39008356	RYR1	693_selected_genes
chr19	39009828	39010119	RYR1	693_selected_genes
chr19	39013642	39013780	RYR1	693_selected_genes
chr19	39013831	39013974	RYR1	693_selected_genes
chr19	39014529	39014594	RYR1	693_selected_genes
chr19	39015946	39016167	RYR1	693_selected_genes
chr19	39017607	39017717	RYR1	693_selected_genes
chr19	39018261	39018449	RYR1	693_selected_genes
chr19	39018704	39018859	RYR1	693_selected_genes
chr19	39018920	39019083	RYR1	693_selected_genes
chr19	39019213	39019360	RYR1	693_selected_genes
chr19	39019565	39019722	RYR1	693_selected_genes
chr19	39023104	39023206	RYR1	693_selected_genes
chr19	39023285	39023401	RYR1	693_selected_genes
chr19	39025334	39025484	RYR1	693_selected_genes
chr19	39025755	39025885	RYR1	693_selected_genes
chr19	39025930	39026057	RYR1	693_selected_genes
chr19	39026611	39026735	RYR1	693_selected_genes
chr19	39027364	39027432	RYR1	693_selected_genes
chr19	39028491	39028625	RYR1	693_selected_genes
chr19	39029173	39029338	RYR1	693_selected_genes
chr19	39033961	39034100	RYR1	693_selected_genes
chr19	39034146	39034325	RYR1	693_selected_genes
chr19	39034385	39034540	RYR1	693_selected_genes
chr19	39037059	39037191	RYR1	693_selected_genes
chr19	39038847	39039085	RYR1	693_selected_genes
chr19	39051727	39052119	RYR1	693_selected_genes
chr19	39055573	39056436	RYR1	693_selected_genes
chr19	39057525	39057652	RYR1	693_selected_genes
chr19	39058387	39058582	RYR1	693_selected_genes
chr19	39061221	39061358	RYR1	693_selected_genes
chr19	39062633	39062935	RYR1	693_selected_genes
chr19	39063791	39063972	RYR1	693_selected_genes
chr19	39066533	39066626	RYR1	693_selected_genes
chr19	39068532	39068713	RYR1	693_selected_genes
chr19	39068758	39068869	RYR1	693_selected_genes
chr19	39070596	39070793	RYR1	693_selected_genes
chr19	39070984	39071169	RYR1	693_selected_genes
chr19	39075557	39075764	RYR1	693_selected_genes
chr19	39076552	39076667	RYR1	693_selected_genes
chr19	39076705	39076856	RYR1	693_selected_genes
chr19	39077139	39077241	RYR1	693_selected_genes
chr19	39077939	39078085	RYR1	693_selected_genes
chr13	23903530	23903648	SACS	693_selected_genes
chr13	23904249	23915854	SACS	693_selected_genes
chr13	23927898	23928040	SACS	693_selected_genes
chr13	23928632	23930171	SACS	693_selected_genes
chr13	23932448	23932645	SACS	693_selected_genes
chr13	23939279	23939441	SACS	693_selected_genes
chr13	23942515	23942651	SACS	693_selected_genes
chr13	23945191	23945329	SACS	693_selected_genes
chr13	23949232	23949433	SACS	693_selected_genes
chr13	23985333	23985403	SACS	693_selected_genes
chr14	51100845	51100917	SAV1	693_selected_genes
chr14	51101875	51102127	SAV1	693_selected_genes
chr14	51107442	51107636	SAV1	693_selected_genes
chr14	51111436	51111757	SAV1	693_selected_genes
chr14	51131871	51132362	SAV1	693_selected_genes
chr14	51134074	51134243	SAV1	693_selected_genes
chr14	51134566	51134710	SAV1	693_selected_genes
chr7	66453332	66453511	SBDS	693_selected_genes
chr7	66456098	66456313	SBDS	693_selected_genes
chr7	66458178	66458429	SBDS	693_selected_genes
chr7	66459173	66459353	SBDS	693_selected_genes
chr7	66460247	66460429	SBDS	693_selected_genes
chr3	38888159	38889258	SCN11A	693_selected_genes

chr3	38891938	38892267	SCN11A	693_selected_genes
chr3	38904660	38904815	SCN11A	693_selected_genes
chr3	38908786	38908974	SCN11A	693_selected_genes
chr3	38912156	38912260	SCN11A	693_selected_genes
chr3	38912910	38913224	SCN11A	693_selected_genes
chr3	38913658	38913810	SCN11A	693_selected_genes
chr3	38921415	38921639	SCN11A	693_selected_genes
chr3	38924698	38924903	SCN11A	693_selected_genes
chr3	38926753	38926918	SCN11A	693_selected_genes
chr3	38927590	38927754	SCN11A	693_selected_genes
chr3	38935998	38936480	SCN11A	693_selected_genes
chr3	38938310	38938741	SCN11A	693_selected_genes
chr3	38941359	38941589	SCN11A	693_selected_genes
chr3	38945330	38945619	SCN11A	693_selected_genes
chr3	38946657	38946837	SCN11A	693_selected_genes
chr3	38949414	38949638	SCN11A	693_selected_genes
chr3	38950462	38950710	SCN11A	693_selected_genes
chr3	38951531	38951723	SCN11A	693_selected_genes
chr3	38961400	38961517	SCN11A	693_selected_genes
chr3	38962541	38962771	SCN11A	693_selected_genes
chr3	38966880	38967025	SCN11A	693_selected_genes
chr3	38968268	38968447	SCN11A	693_selected_genes
chr3	38986876	38987028	SCN11A	693_selected_genes
chr3	38988252	38988423	SCN11A	693_selected_genes
chr3	38991561	38991878	SCN11A	693_selected_genes
chr3	38591786	38593074	SCN5A	693_selected_genes
chr3	38595744	38596065	SCN5A	693_selected_genes
chr3	38597121	38597276	SCN5A	693_selected_genes
chr3	38597906	38598094	SCN5A	693_selected_genes
chr3	38598696	38598800	SCN5A	693_selected_genes
chr3	38601612	38601944	SCN5A	693_selected_genes
chr3	38603880	38604053	SCN5A	693_selected_genes
chr3	38607874	38608098	SCN5A	693_selected_genes
chr3	38616762	38616967	SCN5A	693_selected_genes
chr3	38618126	38618297	SCN5A	693_selected_genes
chr3	38620799	38621011	SCN5A	693_selected_genes
chr3	38622396	38622887	SCN5A	693_selected_genes
chr3	38627156	38627557	SCN5A	693_selected_genes
chr3	38628865	38629089	SCN5A	693_selected_genes
chr3	38639194	38639483	SCN5A	693_selected_genes
chr3	38640383	38640566	SCN5A	693_selected_genes
chr3	38645177	38645599	SCN5A	693_selected_genes
chr3	38646194	38646424	SCN5A	693_selected_genes
chr3	38647416	38647664	SCN5A	693_selected_genes
chr3	38648134	38648326	SCN5A	693_selected_genes
chr3	38649616	38649730	SCN5A	693_selected_genes
chr3	38651199	38651480	SCN5A	693_selected_genes
chr3	38655208	38655350	SCN5A	693_selected_genes
chr3	38655440	38655582	SCN5A	693_selected_genes
chr3	38662308	38662487	SCN5A	693_selected_genes
chr3	38663865	38664005	SCN5A	693_selected_genes
chr3	38671776	38671945	SCN5A	693_selected_genes
chr3	38674500	38674823	SCN5A	693_selected_genes
chr5	218445	218558	SDHA	693_selected_genes
chr5	220329	220477	SDHA	693_selected_genes
chr5	223571	223708	SDHA	693_selected_genes
chr5	224449	224661	SDHA	693_selected_genes
chr5	225508	225702	SDHA	693_selected_genes
chr5	225972	226187	SDHA	693_selected_genes
chr5	228274	228473	SDHA	693_selected_genes
chr5	230965	231140	SDHA	693_selected_genes
chr5	233566	233785	SDHA	693_selected_genes
chr5	235233	235479	SDHA	693_selected_genes
chr5	236517	236739	SDHA	693_selected_genes
chr5	240447	240616	SDHA	693_selected_genes
chr5	251081	251243	SDHA	693_selected_genes
chr5	251426	251608	SDHA	693_selected_genes
chr5	254447	254646	SDHA	693_selected_genes
chr5	256423	256560	SDHA	693_selected_genes
chr11	61197593	61197679	SDHAF2	693_selected_genes
chr11	61204207	61204419	SDHAF2	693_selected_genes
chr11	61205071	61205345	SDHAF2	693_selected_genes
chr11	61205450	61205666	SDHAF2	693_selected_genes
chr11	61205691	61205823	SDHAF2	693_selected_genes
chr11	61213387	61213568	SDHAF2	693_selected_genes
chr1	17345350	17345478	SDHB	693_selected_genes
chr1	17349077	17349250	SDHB	693_selected_genes
chr1	17350442	17350594	SDHB	693_selected_genes
chr1	17354218	17354385	SDHB	693_selected_genes
chr1	17355069	17355256	SDHB	693_selected_genes
chr1	17359529	17359665	SDHB	693_selected_genes
chr1	17371230	17371408	SDHB	693_selected_genes
chr1	17380417	17380539	SDHB	693_selected_genes
chr1	161284170	161284240	SDHC	693_selected_genes

chr1	161293378	161293485	SDHC	693_selected_genes
chr1	161298160	161298312	SDHC	693_selected_genes
chr1	161309326	161309401	SDHC	693_selected_genes
chr1	161310358	161310470	SDHC	693_selected_genes
chr1	161326441	161326655	SDHC	693_selected_genes
chr1	161332093	161332355	SDHC	693_selected_genes
chr11	111957606	111957708	SDHD	693_selected_genes
chr11	111958555	111958722	SDHD	693_selected_genes
chr11	111959565	111959760	SDHD	693_selected_genes
chr11	111961777	111961828	SDHD	693_selected_genes
chr11	111963778	111963946	SDHD	693_selected_genes
chr11	111965503	111965719	SDHD	693_selected_genes
chr11	111978243	111978315	SDHD	693_selected_genes
chr11	111989989	111990040	SDHD	693_selected_genes
chr11	112041116	112041275	SDHD	693_selected_genes
chr18	42281286	42281822	SETBP1	693_selected_genes
chr18	42449169	42449273	SETBP1	693_selected_genes
chr18	42456504	42456743	SETBP1	693_selected_genes
chr18	42529820	42533330	SETBP1	693_selected_genes
chr18	42618424	42618645	SETBP1	693_selected_genes
chr18	42643018	42643688	SETBP1	693_selected_genes
chr3	47058557	47058769	SETD2	693_selected_genes
chr3	47059102	47059254	SETD2	693_selected_genes
chr3	47061224	47061355	SETD2	693_selected_genes
chr3	47079130	47079292	SETD2	693_selected_genes
chr3	47084025	47084215	SETD2	693_selected_genes
chr3	47087951	47088136	SETD2	693_selected_genes
chr3	47098285	47099005	SETD2	693_selected_genes
chr3	47103627	47103861	SETD2	693_selected_genes
chr3	47106078	47106145	SETD2	693_selected_genes
chr3	47108534	47108633	SETD2	693_selected_genes
chr3	47125184	47125897	SETD2	693_selected_genes
chr3	47127659	47127829	SETD2	693_selected_genes
chr3	47129577	47129762	SETD2	693_selected_genes
chr3	47139419	47139596	SETD2	693_selected_genes
chr3	47142922	47143070	SETD2	693_selected_genes
chr3	47144810	47144938	SETD2	693_selected_genes
chr3	47147461	47147635	SETD2	693_selected_genes
chr3	47155340	47155519	SETD2	693_selected_genes
chr3	47158087	47158269	SETD2	693_selected_genes
chr3	47161217	47161295	SETD2	693_selected_genes
chr3	47161646	47166063	SETD2	693_selected_genes
chr3	47168112	47168178	SETD2	693_selected_genes
chr3	47205318	47205439	SETD2	693_selected_genes
chr1	150899228	150899309	SETDB1	693_selected_genes
chr1	150900154	150900475	SETDB1	693_selected_genes
chr1	150902417	150902619	SETDB1	693_selected_genes
chr1	150912390	150912475	SETDB1	693_selected_genes
chr1	150913779	150913929	SETDB1	693_selected_genes
chr1	150915013	150915189	SETDB1	693_selected_genes
chr1	150915302	150915554	SETDB1	693_selected_genes
chr1	150916370	150916494	SETDB1	693_selected_genes
chr1	150917368	150917663	SETDB1	693_selected_genes
chr1	150919336	150919513	SETDB1	693_selected_genes
chr1	150921572	150921779	SETDB1	693_selected_genes
chr1	150921817	150922026	SETDB1	693_selected_genes
chr1	150922908	150923591	SETDB1	693_selected_genes
chr1	150923815	150923982	SETDB1	693_selected_genes
chr1	150931628	150931848	SETDB1	693_selected_genes
chr1	150933013	150933692	SETDB1	693_selected_genes
chr1	150934580	150934659	SETDB1	693_selected_genes
chr1	150935037	150935220	SETDB1	693_selected_genes
chr1	150935424	150935640	SETDB1	693_selected_genes
chr1	150935980	150936352	SETDB1	693_selected_genes
chr1	150936445	150936584	SETDB1	693_selected_genes
chr1	150936697	150936865	SETDB1	693_selected_genes
chr2	198257001	198257210	SF3B1	693_selected_genes
chr2	198257670	198257937	SF3B1	693_selected_genes
chr2	198260754	198261077	SF3B1	693_selected_genes
chr2	198262683	198262865	SF3B1	693_selected_genes
chr2	198263159	198263330	SF3B1	693_selected_genes
chr2	198263667	198263777	SF3B1	693_selected_genes
chr2	198264753	198264915	SF3B1	693_selected_genes
chr2	198264950	198265183	SF3B1	693_selected_genes
chr2	198265413	198265685	SF3B1	693_selected_genes
chr2	198266098	198266274	SF3B1	693_selected_genes
chr2	198266440	198266637	SF3B1	693_selected_genes
chr2	198266683	198266879	SF3B1	693_selected_genes
chr2	198267254	198267575	SF3B1	693_selected_genes
chr2	198267647	198267784	SF3B1	693_selected_genes
chr2	198268283	198268513	SF3B1	693_selected_genes
chr2	198269774	198269926	SF3B1	693_selected_genes
chr2	198269973	198270221	SF3B1	693_selected_genes
chr2	198272696	198272868	SF3B1	693_selected_genes

chr2	198273067	198273330	SF3B1	693_selected_genes
chr2	198274468	198274756	SF3B1	693_selected_genes
chr2	198281439	198281660	SF3B1	693_selected_genes
chr2	198283207	198283337	SF3B1	693_selected_genes
chr2	198283630	198283700	SF3B1	693_selected_genes
chr2	198285076	198285291	SF3B1	693_selected_genes
chr2	198285727	198285882	SF3B1	693_selected_genes
chr2	198288506	198288723	SF3B1	693_selected_genes
chr2	198299670	198299748	SF3B1	693_selected_genes
chr6	134491380	134491598	SGK1	693_selected_genes
chr6	134491938	134492078	SGK1	693_selected_genes
chr6	134492135	134492341	SGK1	693_selected_genes
chr6	134492749	134492895	SGK1	693_selected_genes
chr6	134493305	134493479	SGK1	693_selected_genes
chr6	134493774	134493937	SGK1	693_selected_genes
chr6	134494135	134494317	SGK1	693_selected_genes
chr6	134494386	134494520	SGK1	693_selected_genes
chr6	134494574	134494729	SGK1	693_selected_genes
chr6	134495117	134495243	SGK1	693_selected_genes
chr6	134495623	134495795	SGK1	693_selected_genes
chr6	134495844	134495970	SGK1	693_selected_genes
chr6	134496656	134496824	SGK1	693_selected_genes
chr6	134498775	134498985	SGK1	693_selected_genes
chr6	134528468	134528594	SGK1	693_selected_genes
chr6	134536167	134536256	SGK1	693_selected_genes
chr6	134582868	134583311	SGK1	693_selected_genes
chr6	134638504	134638623	SGK1	693_selected_genes
chr12	111855924	111856706	SH2B3	693_selected_genes
chr12	111872680	111872856	SH2B3	693_selected_genes
chr12	111882517	111882677	SH2B3	693_selected_genes
chr12	111884531	111884683	SH2B3	693_selected_genes
chr12	111884720	111884862	SH2B3	693_selected_genes
chr12	111884903	111885048	SH2B3	693_selected_genes
chr12	111885108	111885373	SH2B3	693_selected_genes
chr12	111885434	111885656	SH2B3	693_selected_genes
chr12	111885761	111886131	SH2B3	693_selected_genes
chrX	123480467	123480654	SH2D1A	693_selected_genes
chrX	123499585	123499699	SH2D1A	693_selected_genes
chrX	123504000	123504195	SH2D1A	693_selected_genes
chrX	123505175	123505266	SH2D1A	693_selected_genes
chr3	72799409	72800012	SHQ1	693_selected_genes
chr3	72842041	72842212	SHQ1	693_selected_genes
chr3	72861796	72861970	SHQ1	693_selected_genes
chr3	72864475	72864579	SHQ1	693_selected_genes
chr3	72866355	72866560	SHQ1	693_selected_genes
chr3	72873549	72873727	SHQ1	693_selected_genes
chr3	72881494	72881657	SHQ1	693_selected_genes
chr3	72890170	72890375	SHQ1	693_selected_genes
chr3	72891405	72891578	SHQ1	693_selected_genes
chr3	72893484	72893629	SHQ1	693_selected_genes
chr3	72895340	72895449	SHQ1	693_selected_genes
chr3	72897323	72897516	SHQ1	693_selected_genes
chr15	75664294	75664575	SIN3A	693_selected_genes
chr15	75667980	75668238	SIN3A	693_selected_genes
chr15	75672944	75673089	SIN3A	693_selected_genes
chr15	75673928	75674071	SIN3A	693_selected_genes
chr15	75676579	75676803	SIN3A	693_selected_genes
chr15	75681967	75682187	SIN3A	693_selected_genes
chr15	75684557	75685181	SIN3A	693_selected_genes
chr15	75686995	75687229	SIN3A	693_selected_genes
chr15	75688573	75688862	SIN3A	693_selected_genes
chr15	75692355	75692522	SIN3A	693_selected_genes
chr15	75693045	75693306	SIN3A	693_selected_genes
chr15	75694167	75694336	SIN3A	693_selected_genes
chr15	75699370	75699510	SIN3A	693_selected_genes
chr15	75702151	75702357	SIN3A	693_selected_genes
chr15	75702449	75702652	SIN3A	693_selected_genes
chr15	75703807	75704109	SIN3A	693_selected_genes
chr15	75705078	75705411	SIN3A	693_selected_genes
chr15	75706520	75706677	SIN3A	693_selected_genes
chr15	75714857	75715189	SIN3A	693_selected_genes
chr15	75722502	75722741	SIN3A	693_selected_genes
chr21	46916223	46916318	SLC19A1	693_selected_genes
chr21	46918156	46918551	SLC19A1	693_selected_genes
chr21	46934864	46935091	SLC19A1	693_selected_genes
chr21	46935546	46936079	SLC19A1	693_selected_genes
chr21	46945705	46945897	SLC19A1	693_selected_genes
chr21	46950658	46950910	SLC19A1	693_selected_genes
chr21	46951277	46952087	SLC19A1	693_selected_genes
chr21	46954426	46954545	SLC19A1	693_selected_genes
chr21	46957659	46957898	SLC19A1	693_selected_genes
chr6	160638438	160638555	SLC22A2	693_selected_genes
chr6	160645711	160645861	SLC22A2	693_selected_genes
chr6	160662480	160662643	SLC22A2	693_selected_genes

chr6	160663300	160663459	SLC22A2	693_selected_genes
chr6	160664578	160664843	SLC22A2	693_selected_genes
chr6	160666441	160666602	SLC22A2	693_selected_genes
chr6	160668190	160668355	SLC22A2	693_selected_genes
chr6	160670222	160670441	SLC22A2	693_selected_genes
chr6	160671554	160671759	SLC22A2	693_selected_genes
chr6	160677620	160677774	SLC22A2	693_selected_genes
chr6	160679350	160679814	SLC22A2	693_selected_genes
chr7	107406274	107406348	SLC26A3	693_selected_genes
chr7	107407998	107408114	SLC26A3	693_selected_genes
chr7	107408185	107408378	SLC26A3	693_selected_genes
chr7	107412473	107412578	SLC26A3	693_selected_genes
chr7	107414339	107414623	SLC26A3	693_selected_genes
chr7	107415196	107415342	SLC26A3	693_selected_genes
chr7	107416871	107417014	SLC26A3	693_selected_genes
chr7	107417056	107417176	SLC26A3	693_selected_genes
chr7	107418594	107418751	SLC26A3	693_selected_genes
chr7	107420087	107420233	SLC26A3	693_selected_genes
chr7	107423216	107423344	SLC26A3	693_selected_genes
chr7	107423399	107423563	SLC26A3	693_selected_genes
chr7	107423624	107423822	SLC26A3	693_selected_genes
chr7	107427246	107427379	SLC26A3	693_selected_genes
chr7	107427776	107427979	SLC26A3	693_selected_genes
chr7	107429943	107430158	SLC26A3	693_selected_genes
chr7	107431467	107431705	SLC26A3	693_selected_genes
chr7	107432249	107432410	SLC26A3	693_selected_genes
chr7	107434161	107434351	SLC26A3	693_selected_genes
chr7	107434798	107434979	SLC26A3	693_selected_genes
chr12	20968647	20968781	SLCO1B3	693_selected_genes
chr12	21007936	21008128	SLCO1B3	693_selected_genes
chr12	21011347	21011530	SLCO1B3	693_selected_genes
chr12	21013925	21014097	SLCO1B3	693_selected_genes
chr12	21015320	21015517	SLCO1B3	693_selected_genes
chr12	21015664	21015813	SLCO1B3	693_selected_genes
chr12	21028143	21028436	SLCO1B3	693_selected_genes
chr12	21030680	21030895	SLCO1B3	693_selected_genes
chr12	21032344	21032590	SLCO1B3	693_selected_genes
chr12	21033763	21033979	SLCO1B3	693_selected_genes
chr12	21036326	21036561	SLCO1B3	693_selected_genes
chr12	21051344	21051459	SLCO1B3	693_selected_genes
chr12	21054258	21054426	SLCO1B3	693_selected_genes
chr12	21054808	21054961	SLCO1B3	693_selected_genes
chr12	21068912	21069206	SLCO1B3	693_selected_genes
chr12	21220227	21220342	SLCO1B3	693_selected_genes
chr12	21229360	21229528	SLCO1B3	693_selected_genes
chr12	21242816	21243065	SLCO1B3	693_selected_genes
chr18	45368172	45368346	SMAD2	693_selected_genes
chr18	45371579	45371880	SMAD2	693_selected_genes
chr18	45372008	45372196	SMAD2	693_selected_genes
chr18	45374820	45375083	SMAD2	693_selected_genes
chr18	45377619	45377723	SMAD2	693_selected_genes
chr18	45391404	45391529	SMAD2	693_selected_genes
chr18	45394668	45394853	SMAD2	693_selected_genes
chr18	45395588	45395832	SMAD2	693_selected_genes
chr18	45396818	45396960	SMAD2	693_selected_genes
chr18	45422866	45423152	SMAD2	693_selected_genes
chr15	67358467	67358723	SMAD3	693_selected_genes
chr15	67391186	67391387	SMAD3	693_selected_genes
chr15	67430339	67430463	SMAD3	693_selected_genes
chr15	67457207	67457451	SMAD3	693_selected_genes
chr15	67457565	67457747	SMAD3	693_selected_genes
chr15	67459091	67459216	SMAD3	693_selected_genes
chr15	67462866	67462967	SMAD3	693_selected_genes
chr15	67473553	67473816	SMAD3	693_selected_genes
chr15	67477039	67477227	SMAD3	693_selected_genes
chr15	67479332	67479493	SMAD3	693_selected_genes
chr15	67479677	67479872	SMAD3	693_selected_genes
chr15	67482725	67482899	SMAD3	693_selected_genes
chr18	48573391	48573690	SMAD4	693_selected_genes
chr18	48575030	48575255	SMAD4	693_selected_genes
chr18	48575639	48575719	SMAD4	693_selected_genes
chr18	48577698	48577810	SMAD4	693_selected_genes
chr18	48578979	48579047	SMAD4	693_selected_genes
chr18	48581125	48581388	SMAD4	693_selected_genes
chr18	48584469	48584639	SMAD4	693_selected_genes
chr18	48584684	48584851	SMAD4	693_selected_genes
chr18	48586210	48586311	SMAD4	693_selected_genes
chr18	48591767	48592001	SMAD4	693_selected_genes
chr18	48593363	48593582	SMAD4	693_selected_genes
chr18	48602982	48603206	SMAD4	693_selected_genes
chr18	48604600	48604862	SMAD4	693_selected_genes
chrX	128581113	128581235	SMARCA1	693_selected_genes
chrX	128582260	128582445	SMARCA1	693_selected_genes
chrX	128599471	128599734	SMARCA1	693_selected_genes

chrX	128599795	128599964	SMARCA1	693_selected_genes
chrX	128602724	128602907	SMARCA1	693_selected_genes
chrX	128605155	128605328	SMARCA1	693_selected_genes
chrX	128614652	128614816	SMARCA1	693_selected_genes
chrX	128615026	128615187	SMARCA1	693_selected_genes
chrX	128620969	128621139	SMARCA1	693_selected_genes
chrX	128622888	128623087	SMARCA1	693_selected_genes
chrX	128624011	128624194	SMARCA1	693_selected_genes
chrX	128625892	128626095	SMARCA1	693_selected_genes
chrX	128626991	128627077	SMARCA1	693_selected_genes
chrX	128630701	128630873	SMARCA1	693_selected_genes
chrX	128631796	128632073	SMARCA1	693_selected_genes
chrX	128633683	128633843	SMARCA1	693_selected_genes
chrX	128638685	128638804	SMARCA1	693_selected_genes
chrX	128640031	128640213	SMARCA1	693_selected_genes
chrX	128641892	128642098	SMARCA1	693_selected_genes
chrX	128645755	128645985	SMARCA1	693_selected_genes
chrX	128649638	128649789	SMARCA1	693_selected_genes
chrX	128649845	128649996	SMARCA1	693_selected_genes
chrX	128650282	128650499	SMARCA1	693_selected_genes
chrX	128652312	128652449	SMARCA1	693_selected_genes
chrX	128657148	128657372	SMARCA1	693_selected_genes
chr19	11094802	11095074	SMARCA4	693_selected_genes
chr19	11095923	11096106	SMARCA4	693_selected_genes
chr19	11096839	11097294	SMARCA4	693_selected_genes
chr19	11097555	11097704	SMARCA4	693_selected_genes
chr19	11098316	11098625	SMARCA4	693_selected_genes
chr19	11099967	11100144	SMARCA4	693_selected_genes
chr19	11101800	11102024	SMARCA4	693_selected_genes
chr19	11105478	11105702	SMARCA4	693_selected_genes
chr19	11106863	11107081	SMARCA4	693_selected_genes
chr19	11107144	11107245	SMARCA4	693_selected_genes
chr19	11113679	11113860	SMARCA4	693_selected_genes
chr19	11113990	11114098	SMARCA4	693_selected_genes
chr19	11118552	11118724	SMARCA4	693_selected_genes
chr19	11121031	11121232	SMARCA4	693_selected_genes
chr19	11123599	11123813	SMARCA4	693_selected_genes
chr19	11129607	11129724	SMARCA4	693_selected_genes
chr19	11130241	11130402	SMARCA4	693_selected_genes
chr19	11132375	11132668	SMARCA4	693_selected_genes
chr19	11134168	11134332	SMARCA4	693_selected_genes
chr19	11134981	11135139	SMARCA4	693_selected_genes
chr19	11136072	11136209	SMARCA4	693_selected_genes
chr19	11136950	11137047	SMARCA4	693_selected_genes
chr19	11138434	11138651	SMARCA4	693_selected_genes
chr19	11141380	11141594	SMARCA4	693_selected_genes
chr19	11143940	11144218	SMARCA4	693_selected_genes
chr19	11144417	11144566	SMARCA4	693_selected_genes
chr19	11144773	11144901	SMARCA4	693_selected_genes
chr19	11145564	11145833	SMARCA4	693_selected_genes
chr19	11150108	11150254	SMARCA4	693_selected_genes
chr19	11151948	11152261	SMARCA4	693_selected_genes
chr19	11168905	11169064	SMARCA4	693_selected_genes
chr19	11169438	11169590	SMARCA4	693_selected_genes
chr19	11170403	11170586	SMARCA4	693_selected_genes
chr19	11170695	11170888	SMARCA4	693_selected_genes
chr19	11172434	11172517	SMARCA4	693_selected_genes
chr19	11175843	11175902	SMARCA4	693_selected_genes
chr22	24129331	24129474	SMARCB1	693_selected_genes
chr22	24133917	24134106	SMARCB1	693_selected_genes
chr22	24135720	24135900	SMARCB1	693_selected_genes
chr22	24143105	24143347	SMARCB1	693_selected_genes
chr22	24145456	24145634	SMARCB1	693_selected_genes
chr22	24158931	24159148	SMARCB1	693_selected_genes
chr22	24167386	24167627	SMARCB1	693_selected_genes
chr22	24175733	24175915	SMARCB1	693_selected_genes
chr22	24176302	24176392	SMARCB1	693_selected_genes
chr12	50479127	50479354	SMARCD1	693_selected_genes
chr12	50479918	50480156	SMARCD1	693_selected_genes
chr12	50480378	50480471	SMARCD1	693_selected_genes
chr12	50480513	50480686	SMARCD1	693_selected_genes
chr12	50481120	50481293	SMARCD1	693_selected_genes
chr12	50481996	50482098	SMARCD1	693_selected_genes
chr12	50482278	50482445	SMARCD1	693_selected_genes
chr12	50483641	50483793	SMARCD1	693_selected_genes
chr12	50483998	50484210	SMARCD1	693_selected_genes
chr12	50484250	50484398	SMARCD1	693_selected_genes
chr12	50488194	50488380	SMARCD1	693_selected_genes
chr12	50490607	50490780	SMARCD1	693_selected_genes
chr12	50492471	50492623	SMARCD1	693_selected_genes
chr12	50492704	50492808	SMARCD1	693_selected_genes
chrX	53406998	53407132	SMC1A	693_selected_genes
chrX	53407515	53407676	SMC1A	693_selected_genes
chrX	53407913	53408033	SMC1A	693_selected_genes

chrX	53409127	53409329	SMC1A	693_selected_genes
chrX	53409401	53409606	SMC1A	693_selected_genes
chrX	53409992	53410199	SMC1A	693_selected_genes
chrX	53421672	53421833	SMC1A	693_selected_genes
chrX	53423121	53423325	SMC1A	693_selected_genes
chrX	53423366	53423562	SMC1A	693_selected_genes
chrX	53426485	53426677	SMC1A	693_selected_genes
chrX	53430472	53430629	SMC1A	693_selected_genes
chrX	53430683	53430850	SMC1A	693_selected_genes
chrX	53431861	53432106	SMC1A	693_selected_genes
chrX	53432151	53432348	SMC1A	693_selected_genes
chrX	53432399	53432629	SMC1A	693_selected_genes
chrX	53432677	53432913	SMC1A	693_selected_genes
chrX	53435967	53436225	SMC1A	693_selected_genes
chrX	53436326	53436459	SMC1A	693_selected_genes
chrX	53438685	53438876	SMC1A	693_selected_genes
chrX	53438919	53439228	SMC1A	693_selected_genes
chrX	53439824	53440113	SMC1A	693_selected_genes
chrX	53440156	53440410	SMC1A	693_selected_genes
chrX	53441681	53441844	SMC1A	693_selected_genes
chrX	53441904	53442143	SMC1A	693_selected_genes
chrX	53448819	53448992	SMC1A	693_selected_genes
chrX	53449415	53449574	SMC1A	693_selected_genes
chr10	112327549	112327614	SMC3	693_selected_genes
chr10	112328670	112328796	SMC3	693_selected_genes
chr10	112333439	112333528	SMC3	693_selected_genes
chr10	112335068	112335186	SMC3	693_selected_genes
chr10	112337153	112337275	SMC3	693_selected_genes
chr10	112337567	112337697	SMC3	693_selected_genes
chr10	112338360	112338489	SMC3	693_selected_genes
chr10	112340636	112340804	SMC3	693_selected_genes
chr10	112341655	112341881	SMC3	693_selected_genes
chr10	112342294	112342425	SMC3	693_selected_genes
chr10	112343116	112343331	SMC3	693_selected_genes
chr10	112343573	112343745	SMC3	693_selected_genes
chr10	112343915	112344179	SMC3	693_selected_genes
chr10	112349337	112349491	SMC3	693_selected_genes
chr10	112349624	112349774	SMC3	693_selected_genes
chr10	112350144	112350355	SMC3	693_selected_genes
chr10	112350723	112350915	SMC3	693_selected_genes
chr10	112352805	112353006	SMC3	693_selected_genes
chr10	112356130	112356333	SMC3	693_selected_genes
chr10	112357871	112358073	SMC3	693_selected_genes
chr10	112359386	112359595	SMC3	693_selected_genes
chr10	112360171	112360329	SMC3	693_selected_genes
chr10	112360754	112360913	SMC3	693_selected_genes
chr10	112361369	112361667	SMC3	693_selected_genes
chr10	112361698	112361961	SMC3	693_selected_genes
chr10	112362206	112362448	SMC3	693_selected_genes
chr10	112362557	112362785	SMC3	693_selected_genes
chr10	112362916	112363073	SMC3	693_selected_genes
chr10	112363963	112364085	SMC3	693_selected_genes
chr7	128828967	128829348	SMO	693_selected_genes
chr7	128843199	128843455	SMO	693_selected_genes
chr7	128845018	128845278	SMO	693_selected_genes
chr7	128845425	128845648	SMO	693_selected_genes
chr7	128845965	128846235	SMO	693_selected_genes
chr7	128846279	128846453	SMO	693_selected_genes
chr7	128848574	128848717	SMO	693_selected_genes
chr7	128849104	128849263	SMO	693_selected_genes
chr7	128850178	128850414	SMO	693_selected_genes
chr7	128850780	128850979	SMO	693_selected_genes
chr7	128851198	128851271	SMO	693_selected_genes
chr7	128851451	128851636	SMO	693_selected_genes
chr7	128851839	128852317	SMO	693_selected_genes
chr5	121726804	121726911	SNCAIP	693_selected_genes
chr5	121736655	121736850	SNCAIP	693_selected_genes
chr5	121739462	121739585	SNCAIP	693_selected_genes
chr5	121758537	121759459	SNCAIP	693_selected_genes
chr5	121761021	121761251	SNCAIP	693_selected_genes
chr5	121767638	121767802	SNCAIP	693_selected_genes
chr5	121776298	121776474	SNCAIP	693_selected_genes
chr5	121779240	121779405	SNCAIP	693_selected_genes
chr5	121779755	121779894	SNCAIP	693_selected_genes
chr5	121780232	121780452	SNCAIP	693_selected_genes
chr5	121785514	121785657	SNCAIP	693_selected_genes
chr5	121786202	121787324	SNCAIP	693_selected_genes
chr5	121788594	121788715	SNCAIP	693_selected_genes
chr5	121799160	121799295	SNCAIP	693_selected_genes
chr16	11348674	11349360	SOCS1	693_selected_genes
chr6	160103499	160103695	SOD2	693_selected_genes
chr6	160105860	160106090	SOD2	693_selected_genes
chr6	160109132	160109299	SOD2	693_selected_genes
chr6	160113470	160113920	SOD2	693_selected_genes

chr6	160114151	160114287	SOD2	693_selected_genes
chr6	160147858	160148000	SOD2	693_selected_genes
chr6	160148272	160148414	SOD2	693_selected_genes
chr2	39212939	39213481	SOS1	693_selected_genes
chr2	39214588	39214757	SOS1	693_selected_genes
chr2	39216385	39216480	SOS1	693_selected_genes
chr2	39222238	39222553	SOS1	693_selected_genes
chr2	39224037	39224204	SOS1	693_selected_genes
chr2	39224368	39224591	SOS1	693_selected_genes
chr2	39233527	39233695	SOS1	693_selected_genes
chr2	39234146	39234359	SOS1	693_selected_genes
chr2	39237699	39237869	SOS1	693_selected_genes
chr2	39239241	39239514	SOS1	693_selected_genes
chr2	39240575	39240729	SOS1	693_selected_genes
chr2	39240982	39241155	SOS1	693_selected_genes
chr2	39241880	39242012	SOS1	693_selected_genes
chr2	39249685	39250391	SOS1	693_selected_genes
chr2	39251125	39251303	SOS1	693_selected_genes
chr2	39261932	39262024	SOS1	693_selected_genes
chr2	39262327	39262476	SOS1	693_selected_genes
chr2	39262505	39262666	SOS1	693_selected_genes
chr2	39278259	39278453	SOS1	693_selected_genes
chr2	39281729	39281989	SOS1	693_selected_genes
chr2	39283817	39284032	SOS1	693_selected_genes
chr2	39285788	39285970	SOS1	693_selected_genes
chr2	39294743	39294919	SOS1	693_selected_genes
chr2	39347451	39347588	SOS1	693_selected_genes
chr22	38367223	38367330	SOX10	693_selected_genes
chr22	38369476	38370230	SOX10	693_selected_genes
chr22	38373848	38374167	SOX10	693_selected_genes
chr22	38379338	38379816	SOX10	693_selected_genes
chr8	55370673	55371030	SOX17	693_selected_genes
chr8	55371592	55372580	SOX17	693_selected_genes
chr3	181430123	181431127	SOX2	693_selected_genes
chr17	70117507	70117988	SOX9	693_selected_genes
chr17	70118834	70119138	SOX9	693_selected_genes
chr17	70119658	70120553	SOX9	693_selected_genes
chr1	16174537	16174670	SPEN	693_selected_genes
chr1	16199285	16199656	SPEN	693_selected_genes
chr1	16200584	16200915	SPEN	693_selected_genes
chr1	16202671	16203198	SPEN	693_selected_genes
chr1	16235790	16236001	SPEN	693_selected_genes
chr1	16237570	16237821	SPEN	693_selected_genes
chr1	16242597	16242799	SPEN	693_selected_genes
chr1	16245395	16245571	SPEN	693_selected_genes
chr1	16245873	16246037	SPEN	693_selected_genes
chr1	16247339	16247503	SPEN	693_selected_genes
chr1	16248718	16248869	SPEN	693_selected_genes
chr1	16254560	16262786	SPEN	693_selected_genes
chr1	16263632	16264165	SPEN	693_selected_genes
chr1	16264281	16264526	SPEN	693_selected_genes
chr1	16265187	16265396	SPEN	693_selected_genes
chr1	16265765	16265947	SPEN	693_selected_genes
chr17	47677714	47677909	SPOP	693_selected_genes
chr17	47679201	47679394	SPOP	693_selected_genes
chr17	47684586	47684759	SPOP	693_selected_genes
chr17	47685210	47685316	SPOP	693_selected_genes
chr17	47688616	47688844	SPOP	693_selected_genes
chr17	47695882	47695950	SPOP	693_selected_genes
chr17	47696317	47696495	SPOP	693_selected_genes
chr17	47696570	47696772	SPOP	693_selected_genes
chr17	47698006	47698057	SPOP	693_selected_genes
chr17	47699282	47699454	SPOP	693_selected_genes
chr17	47699563	47699670	SPOP	693_selected_genes
chr17	47700069	47700197	SPOP	693_selected_genes
chr1	158581028	158581204	SPTA1	693_selected_genes
chr1	158582581	158582776	SPTA1	693_selected_genes
chr1	158583485	158583682	SPTA1	693_selected_genes
chr1	158584017	158584121	SPTA1	693_selected_genes
chr1	158584980	158585218	SPTA1	693_selected_genes
chr1	158587301	158587403	SPTA1	693_selected_genes
chr1	158587803	158587871	SPTA1	693_selected_genes
chr1	158588986	158589149	SPTA1	693_selected_genes
chr1	158589934	158590281	SPTA1	693_selected_genes
chr1	158592747	158593007	SPTA1	693_selected_genes
chr1	158595910	158596037	SPTA1	693_selected_genes
chr1	158596603	158596822	SPTA1	693_selected_genes
chr1	158597389	158597538	SPTA1	693_selected_genes
chr1	158604307	158604490	SPTA1	693_selected_genes
chr1	158605677	158605849	SPTA1	693_selected_genes
chr1	158606405	158606576	SPTA1	693_selected_genes
chr1	158607797	158608056	SPTA1	693_selected_genes
chr1	158609346	158609501	SPTA1	693_selected_genes
chr1	158609634	158609822	SPTA1	693_selected_genes

chr1	158612175	158612357	SPTA1	693_selected_genes
chr1	158612578	158612791	SPTA1	693_selected_genes
chr1	158613086	158613240	SPTA1	693_selected_genes
chr1	158614017	158614211	SPTA1	693_selected_genes
chr1	158614952	158615200	SPTA1	693_selected_genes
chr1	158615259	158615409	SPTA1	693_selected_genes
chr1	158617303	158617535	SPTA1	693_selected_genes
chr1	158618273	158618468	SPTA1	693_selected_genes
chr1	158619620	158619762	SPTA1	693_selected_genes
chr1	158621131	158621283	SPTA1	693_selected_genes
chr1	158622231	158622468	SPTA1	693_selected_genes
chr1	158623038	158623240	SPTA1	693_selected_genes
chr1	158624375	158624563	SPTA1	693_selected_genes
chr1	158626328	158626471	SPTA1	693_selected_genes
chr1	158627241	158627509	SPTA1	693_selected_genes
chr1	158631051	158631224	SPTA1	693_selected_genes
chr1	158632466	158632760	SPTA1	693_selected_genes
chr1	158636080	158636312	SPTA1	693_selected_genes
chr1	158637622	158637877	SPTA1	693_selected_genes
chr1	158639172	158639378	SPTA1	693_selected_genes
chr1	158639473	158639601	SPTA1	693_selected_genes
chr1	158641107	158641268	SPTA1	693_selected_genes
chr1	158641823	158642011	SPTA1	693_selected_genes
chr1	158644093	158644245	SPTA1	693_selected_genes
chr1	158644304	158644490	SPTA1	693_selected_genes
chr1	158645905	158646110	SPTA1	693_selected_genes
chr1	158647454	158647649	SPTA1	693_selected_genes
chr1	158648165	158648349	SPTA1	693_selected_genes
chr1	158650347	158650544	SPTA1	693_selected_genes
chr1	158651291	158651482	SPTA1	693_selected_genes
chr1	158653135	158653311	SPTA1	693_selected_genes
chr1	158654872	158655162	SPTA1	693_selected_genes
chr1	158656258	158656332	SPTA1	693_selected_genes
chr9	131328994	131329281	SPTAN1	693_selected_genes
chr9	131331025	131331201	SPTAN1	693_selected_genes
chr9	131336928	131337119	SPTAN1	693_selected_genes
chr9	131337452	131337649	SPTAN1	693_selected_genes
chr9	131339076	131339260	SPTAN1	693_selected_genes
chr9	131339382	131339577	SPTAN1	693_selected_genes
chr9	131339605	131339810	SPTAN1	693_selected_genes
chr9	131340363	131340549	SPTAN1	693_selected_genes
chr9	131341890	131342042	SPTAN1	693_selected_genes
chr9	131343175	131343363	SPTAN1	693_selected_genes
chr9	131344035	131344196	SPTAN1	693_selected_genes
chr9	131344732	131344860	SPTAN1	693_selected_genes
chr9	131344947	131345153	SPTAN1	693_selected_genes
chr9	131345330	131345585	SPTAN1	693_selected_genes
chr9	131346041	131346273	SPTAN1	693_selected_genes
chr9	131346535	131346829	SPTAN1	693_selected_genes
chr9	131346974	131347147	SPTAN1	693_selected_genes
chr9	131348001	131348269	SPTAN1	693_selected_genes
chr9	131349859	131350002	SPTAN1	693_selected_genes
chr9	131351062	131351247	SPTAN1	693_selected_genes
chr9	131353730	131353929	SPTAN1	693_selected_genes
chr9	131355236	131355346	SPTAN1	693_selected_genes
chr9	131356428	131356677	SPTAN1	693_selected_genes
chr9	131360653	131360808	SPTAN1	693_selected_genes
chr9	131361216	131361290	SPTAN1	693_selected_genes
chr9	131362333	131362419	SPTAN1	693_selected_genes
chr9	131365796	131365894	SPTAN1	693_selected_genes
chr9	131366579	131366721	SPTAN1	693_selected_genes
chr9	131367287	131367482	SPTAN1	693_selected_genes
chr9	131367549	131367781	SPTAN1	693_selected_genes
chr9	131369857	131370007	SPTAN1	693_selected_genes
chr9	131370105	131370353	SPTAN1	693_selected_genes
chr9	131370383	131370580	SPTAN1	693_selected_genes
chr9	131371127	131371281	SPTAN1	693_selected_genes
chr9	131371375	131371634	SPTAN1	693_selected_genes
chr9	131371904	131371969	SPTAN1	693_selected_genes
chr9	131373967	131374149	SPTAN1	693_selected_genes
chr9	131374362	131374550	SPTAN1	693_selected_genes
chr9	131375634	131375789	SPTAN1	693_selected_genes
chr9	131377885	131378144	SPTAN1	693_selected_genes
chr9	131379893	131380064	SPTAN1	693_selected_genes
chr9	131380240	131380412	SPTAN1	693_selected_genes
chr9	131381124	131381307	SPTAN1	693_selected_genes
chr9	131383411	131383560	SPTAN1	693_selected_genes
chr9	131386581	131386791	SPTAN1	693_selected_genes
chr9	131387356	131387483	SPTAN1	693_selected_genes
chr9	131388022	131388282	SPTAN1	693_selected_genes
chr9	131388644	131388991	SPTAN1	693_selected_genes
chr9	131389639	131389820	SPTAN1	693_selected_genes
chr9	131390178	131390246	SPTAN1	693_selected_genes
chr9	131392574	131392679	SPTAN1	693_selected_genes

chr9	131394380	131394627	SPTAN1	693_selected_genes
chr9	131394684	131394788	SPTAN1	693_selected_genes
chr9	131394825	131395022	SPTAN1	693_selected_genes
chr9	131395061	131395259	SPTAN1	693_selected_genes
chr9	131395462	131395638	SPTAN1	693_selected_genes
chr20	36012531	36012831	SRC	693_selected_genes
chr20	36014452	36014602	SRC	693_selected_genes
chr20	36014837	36014905	SRC	693_selected_genes
chr20	36022272	36022421	SRC	693_selected_genes
chr20	36022551	36022705	SRC	693_selected_genes
chr20	36024539	36024739	SRC	693_selected_genes
chr20	36026076	36026282	SRC	693_selected_genes
chr20	36028492	36028722	SRC	693_selected_genes
chr20	36029979	36030106	SRC	693_selected_genes
chr20	36030812	36031016	SRC	693_selected_genes
chr20	36031126	36031308	SRC	693_selected_genes
chr20	36031548	36031807	SRC	693_selected_genes
chr17	74732217	74732571	SRSF2	693_selected_genes
chr17	74732855	74733267	SRSF2	693_selected_genes
chrX	123156452	123156546	STAG2	693_selected_genes
chrX	123159664	123159793	STAG2	693_selected_genes
chrX	123160389	123160493	STAG2	693_selected_genes
chrX	123164785	123165000	STAG2	693_selected_genes
chrX	123171351	123171498	STAG2	693_selected_genes
chrX	123176393	123176520	STAG2	693_selected_genes
chrX	123178988	123179243	STAG2	693_selected_genes
chrX	123181178	123181380	STAG2	693_selected_genes
chrX	123182829	123182953	STAG2	693_selected_genes
chrX	123184010	123184184	STAG2	693_selected_genes
chrX	123184945	123185094	STAG2	693_selected_genes
chrX	123185139	123185269	STAG2	693_selected_genes
chrX	123189952	123190110	STAG2	693_selected_genes
chrX	123191690	123191852	STAG2	693_selected_genes
chrX	123195048	123195216	STAG2	693_selected_genes
chrX	123195595	123195749	STAG2	693_selected_genes
chrX	123196726	123196869	STAG2	693_selected_genes
chrX	123196940	123197080	STAG2	693_selected_genes
chrX	123197672	123197926	STAG2	693_selected_genes
chrX	123199700	123199821	STAG2	693_selected_genes
chrX	123199999	123200137	STAG2	693_selected_genes
chrX	123200180	123200311	STAG2	693_selected_genes
chrX	123202388	123202531	STAG2	693_selected_genes
chrX	123204973	123205198	STAG2	693_selected_genes
chrX	123210156	123210346	STAG2	693_selected_genes
chrX	123211781	123211933	STAG2	693_selected_genes
chrX	123215204	123215403	STAG2	693_selected_genes
chrX	123217245	123217424	STAG2	693_selected_genes
chrX	123220371	123220645	STAG2	693_selected_genes
chrX	123224399	123224639	STAG2	693_selected_genes
chrX	123224678	123224839	STAG2	693_selected_genes
chrX	123227842	123228019	STAG2	693_selected_genes
chrX	123229196	123229324	STAG2	693_selected_genes
chrX	123234398	123234472	STAG2	693_selected_genes
chr17	40467737	40467843	STAT3	693_selected_genes
chr17	40468781	40468944	STAT3	693_selected_genes
chr17	40469174	40469267	STAT3	693_selected_genes
chr17	40474274	40474537	STAT3	693_selected_genes
chr17	40474996	40475186	STAT3	693_selected_genes
chr17	40475252	40475397	STAT3	693_selected_genes
chr17	40475565	40475668	STAT3	693_selected_genes
chr17	40476703	40476889	STAT3	693_selected_genes
chr17	40476955	40477104	STAT3	693_selected_genes
chr17	40478108	40478242	STAT3	693_selected_genes
chr17	40481402	40481500	STAT3	693_selected_genes
chr17	40481546	40481690	STAT3	693_selected_genes
chr17	40481739	40481819	STAT3	693_selected_genes
chr17	40483464	40483574	STAT3	693_selected_genes
chr17	40485665	40485808	STAT3	693_selected_genes
chr17	40485883	40486092	STAT3	693_selected_genes
chr17	40489427	40489629	STAT3	693_selected_genes
chr17	40489755	40489900	STAT3	693_selected_genes
chr17	40490723	40490855	STAT3	693_selected_genes
chr17	40491306	40491452	STAT3	693_selected_genes
chr17	40497551	40497700	STAT3	693_selected_genes
chr17	40498561	40498756	STAT3	693_selected_genes
chr17	40500381	40500560	STAT3	693_selected_genes
chr17	40526051	40526106	STAT3	693_selected_genes
chr2	191894540	191894617	STAT4	693_selected_genes
chr2	191895672	191895831	STAT4	693_selected_genes
chr2	191896150	191896267	STAT4	693_selected_genes
chr2	191897658	191897900	STAT4	693_selected_genes
chr2	191898190	191898377	STAT4	693_selected_genes
chr2	191898611	191898756	STAT4	693_selected_genes
chr2	191899248	191899348	STAT4	693_selected_genes

chr2	191900864	191901050	STAT4	693_selected_genes
chr2	191903899	191904048	STAT4	693_selected_genes
chr2	191905765	191905899	STAT4	693_selected_genes
chr2	191919190	191919285	STAT4	693_selected_genes
chr2	191922718	191922862	STAT4	693_selected_genes
chr2	191922902	191922970	STAT4	693_selected_genes
chr2	191923410	191923520	STAT4	693_selected_genes
chr2	191926429	191926572	STAT4	693_selected_genes
chr2	191927462	191927671	STAT4	693_selected_genes
chr2	191929507	191929709	STAT4	693_selected_genes
chr2	191931130	191931266	STAT4	693_selected_genes
chr2	191934393	191934522	STAT4	693_selected_genes
chr2	191937798	191937941	STAT4	693_selected_genes
chr2	191940927	191941076	STAT4	693_selected_genes
chr2	191996520	191996651	STAT4	693_selected_genes
chr2	192011313	192011508	STAT4	693_selected_genes
chr2	192012776	192012954	STAT4	693_selected_genes
chr19	1206887	1207227	STK11	693_selected_genes
chr19	1216241	1216307	STK11	693_selected_genes
chr19	1218390	1218524	STK11	693_selected_genes
chr19	1219297	1219437	STK11	693_selected_genes
chr19	1220346	1220529	STK11	693_selected_genes
chr19	1220554	1220741	STK11	693_selected_genes
chr19	1221186	1221364	STK11	693_selected_genes
chr19	1221922	1222030	STK11	693_selected_genes
chr19	1222958	1223196	STK11	693_selected_genes
chr19	1226427	1226687	STK11	693_selected_genes
chr19	1227566	1227881	STK11	693_selected_genes
chr1	36807330	36807599	STK40	693_selected_genes
chr1	36808774	36809074	STK40	693_selected_genes
chr1	36809435	36809605	STK40	693_selected_genes
chr1	36809695	36809890	STK40	693_selected_genes
chr1	36814275	36814441	STK40	693_selected_genes
chr1	36819939	36820042	STK40	693_selected_genes
chr1	36820781	36821059	STK40	693_selected_genes
chr1	36823814	36824008	STK40	693_selected_genes
chr1	36824312	36824463	STK40	693_selected_genes
chr1	36826796	36826958	STK40	693_selected_genes
chr10	104263884	104264116	SUFU	693_selected_genes
chr10	104268900	104269085	SUFU	693_selected_genes
chr10	104309701	104309888	SUFU	693_selected_genes
chr10	104352313	104352506	SUFU	693_selected_genes
chr10	104353367	104353503	SUFU	693_selected_genes
chr10	104353724	104353847	SUFU	693_selected_genes
chr10	104356871	104357075	SUFU	693_selected_genes
chr10	104359164	104359326	SUFU	693_selected_genes
chr10	104374999	104375184	SUFU	693_selected_genes
chr10	104377021	104377360	SUFU	693_selected_genes
chr10	104378797	104378853	SUFU	693_selected_genes
chr10	104386906	104387025	SUFU	693_selected_genes
chr10	104389797	104389937	SUFU	693_selected_genes
chr16	28617116	28617279	SULT1A1	693_selected_genes
chr16	28617351	28617582	SULT1A1	693_selected_genes
chr16	28618056	28618201	SULT1A1	693_selected_genes
chr16	28618246	28618423	SULT1A1	693_selected_genes
chr16	28619586	28619734	SULT1A1	693_selected_genes
chr16	28619773	28619949	SULT1A1	693_selected_genes
chr16	28620003	28620205	SULT1A1	693_selected_genes
chr16	28621454	28621537	SULT1A1	693_selected_genes
chr16	28631358	28631479	SULT1A1	693_selected_genes
chr16	28634426	28634543	SULT1A1	693_selected_genes
chr17	30264240	30264564	SUZ12	693_selected_genes
chr17	30267279	30267376	SUZ12	693_selected_genes
chr17	30267415	30267530	SUZ12	693_selected_genes
chr17	30274610	30274729	SUZ12	693_selected_genes
chr17	30293140	30293240	SUZ12	693_selected_genes
chr17	30300139	30300275	SUZ12	693_selected_genes
chr17	30302475	30302757	SUZ12	693_selected_genes
chr17	30303514	30303658	SUZ12	693_selected_genes
chr17	30309992	30310148	SUZ12	693_selected_genes
chr17	30315313	30315541	SUZ12	693_selected_genes
chr17	30320235	30320377	SUZ12	693_selected_genes
chr17	30320858	30321052	SUZ12	693_selected_genes
chr17	30321557	30321765	SUZ12	693_selected_genes
chr17	30322557	30322806	SUZ12	693_selected_genes
chr17	30323791	30323921	SUZ12	693_selected_genes
chr17	30325651	30326047	SUZ12	693_selected_genes
chr9	93606155	93606622	SYK	693_selected_genes
chr9	93607690	93607901	SYK	693_selected_genes
chr9	93624462	93624651	SYK	693_selected_genes
chr9	93626845	93626974	SYK	693_selected_genes
chr9	93627304	93627404	SYK	693_selected_genes
chr9	93629387	93629506	SYK	693_selected_genes
chr9	93636460	93636598	SYK	693_selected_genes

chr9	93636928	93637156	SYK	693_selected_genes
chr9	93639827	93640087	SYK	693_selected_genes
chr9	93641020	93641260	SYK	693_selected_genes
chr9	93650005	93650196	SYK	693_selected_genes
chr9	93650771	93650934	SYK	693_selected_genes
chr9	93657784	93657907	SYK	693_selected_genes
chr6	152443545	152443836	SYNE1	693_selected_genes
chr6	152451829	152451938	SYNE1	693_selected_genes
chr6	152453231	152453374	SYNE1	693_selected_genes
chr6	152454385	152454648	SYNE1	693_selected_genes
chr6	152456213	152456392	SYNE1	693_selected_genes
chr6	152457727	152457978	SYNE1	693_selected_genes
chr6	152461059	152461321	SYNE1	693_selected_genes
chr6	152462312	152462489	SYNE1	693_selected_genes
chr6	152464732	152464925	SYNE1	693_selected_genes
chr6	152466596	152466715	SYNE1	693_selected_genes
chr6	152469154	152469538	SYNE1	693_selected_genes
chr6	152470586	152470828	SYNE1	693_selected_genes
chr6	152472662	152472850	SYNE1	693_selected_genes
chr6	152473068	152473301	SYNE1	693_selected_genes
chr6	152476001	152476202	SYNE1	693_selected_genes
chr6	152477019	152477257	SYNE1	693_selected_genes
chr6	152485272	152485485	SYNE1	693_selected_genes
chr6	152489222	152489364	SYNE1	693_selected_genes
chr6	152497503	152497720	SYNE1	693_selected_genes
chr6	152501245	152501454	SYNE1	693_selected_genes
chr6	152510361	152510567	SYNE1	693_selected_genes
chr6	152522933	152523109	SYNE1	693_selected_genes
chr6	152527277	152527522	SYNE1	693_selected_genes
chr6	152529081	152529366	SYNE1	693_selected_genes
chr6	152532603	152532748	SYNE1	693_selected_genes
chr6	152534721	152534919	SYNE1	693_selected_genes
chr6	152536015	152536220	SYNE1	693_selected_genes
chr6	152539366	152539563	SYNE1	693_selected_genes
chr6	152540112	152540345	SYNE1	693_selected_genes
chr6	152541951	152542206	SYNE1	693_selected_genes
chr6	152542535	152542719	SYNE1	693_selected_genes
chr6	152545603	152545824	SYNE1	693_selected_genes
chr6	152546830	152547036	SYNE1	693_selected_genes
chr6	152551656	152551862	SYNE1	693_selected_genes
chr6	152552500	152552727	SYNE1	693_selected_genes
chr6	152553225	152553425	SYNE1	693_selected_genes
chr6	152554890	152555123	SYNE1	693_selected_genes
chr6	152555777	152555960	SYNE1	693_selected_genes
chr6	152557216	152557463	SYNE1	693_selected_genes
chr6	152557926	152558108	SYNE1	693_selected_genes
chr6	152560642	152560866	SYNE1	693_selected_genes
chr6	152563349	152563600	SYNE1	693_selected_genes
chr6	152565646	152565816	SYNE1	693_selected_genes
chr6	152570270	152570422	SYNE1	693_selected_genes
chr6	152575989	152576249	SYNE1	693_selected_genes
chr6	152576700	152576906	SYNE1	693_selected_genes
chr6	152577743	152577925	SYNE1	693_selected_genes
chr6	152583141	152583348	SYNE1	693_selected_genes
chr6	152589165	152589325	SYNE1	693_selected_genes
chr6	152590264	152590446	SYNE1	693_selected_genes
chr6	152599198	152599440	SYNE1	693_selected_genes
chr6	152602916	152603140	SYNE1	693_selected_genes
chr6	152605087	152605332	SYNE1	693_selected_genes
chr6	152614697	152614909	SYNE1	693_selected_genes
chr6	152615069	152615287	SYNE1	693_selected_genes
chr6	152621750	152621941	SYNE1	693_selected_genes
chr6	152622978	152623235	SYNE1	693_selected_genes
chr6	152629598	152629792	SYNE1	693_selected_genes
chr6	152630940	152631177	SYNE1	693_selected_genes
chr6	152631505	152631678	SYNE1	693_selected_genes
chr6	152631797	152632033	SYNE1	693_selected_genes
chr6	152637958	152638146	SYNE1	693_selected_genes
chr6	152639190	152639423	SYNE1	693_selected_genes
chr6	152639972	152640175	SYNE1	693_selected_genes
chr6	152642347	152642550	SYNE1	693_selected_genes
chr6	152642830	152643046	SYNE1	693_selected_genes
chr6	152644587	152644897	SYNE1	693_selected_genes
chr6	152646193	152646462	SYNE1	693_selected_genes
chr6	152647067	152647262	SYNE1	693_selected_genes
chr6	152647405	152647793	SYNE1	693_selected_genes
chr6	152650839	152653050	SYNE1	693_selected_genes
chr6	152655117	152655433	SYNE1	693_selected_genes
chr6	152657950	152658177	SYNE1	693_selected_genes
chr6	152660350	152660526	SYNE1	693_selected_genes
chr6	152665190	152665387	SYNE1	693_selected_genes
chr6	152668168	152668395	SYNE1	693_selected_genes
chr6	152671277	152671495	SYNE1	693_selected_genes
chr6	152671727	152671930	SYNE1	693_selected_genes

chr6	152673136	152673513	SYNE1	693_selected_genes
chr6	152674372	152674593	SYNE1	693_selected_genes
chr6	152674698	152674904	SYNE1	693_selected_genes
chr6	152675768	152676136	SYNE1	693_selected_genes
chr6	152679482	152679697	SYNE1	693_selected_genes
chr6	152680424	152680618	SYNE1	693_selected_genes
chr6	152683279	152683483	SYNE1	693_selected_genes
chr6	152684835	152684934	SYNE1	693_selected_genes
chr6	152685956	152686179	SYNE1	693_selected_genes
chr6	152688114	152688542	SYNE1	693_selected_genes
chr6	152690081	152690287	SYNE1	693_selected_genes
chr6	152690580	152690774	SYNE1	693_selected_genes
chr6	152694146	152694379	SYNE1	693_selected_genes
chr6	152697490	152697718	SYNE1	693_selected_genes
chr6	152697885	152698072	SYNE1	693_selected_genes
chr6	152702115	152702522	SYNE1	693_selected_genes
chr6	152706783	152706998	SYNE1	693_selected_genes
chr6	152708181	152708541	SYNE1	693_selected_genes
chr6	152711389	152711612	SYNE1	693_selected_genes
chr6	152712386	152712728	SYNE1	693_selected_genes
chr6	152716625	152716831	SYNE1	693_selected_genes
chr6	152717884	152718143	SYNE1	693_selected_genes
chr6	152719728	152719894	SYNE1	693_selected_genes
chr6	152720725	152720983	SYNE1	693_selected_genes
chr6	152722247	152722501	SYNE1	693_selected_genes
chr6	152725322	152725474	SYNE1	693_selected_genes
chr6	152728123	152728356	SYNE1	693_selected_genes
chr6	152730177	152730386	SYNE1	693_selected_genes
chr6	152730668	152730869	SYNE1	693_selected_genes
chr6	152734461	152734691	SYNE1	693_selected_genes
chr6	152737496	152738175	SYNE1	693_selected_genes
chr6	152740678	152740882	SYNE1	693_selected_genes
chr6	152746490	152746707	SYNE1	693_selected_genes
chr6	152748724	152748976	SYNE1	693_selected_genes
chr6	152749314	152749552	SYNE1	693_selected_genes
chr6	152751221	152751370	SYNE1	693_selected_genes
chr6	152751591	152751869	SYNE1	693_selected_genes
chr6	152754904	152755105	SYNE1	693_selected_genes
chr6	152757028	152757261	SYNE1	693_selected_genes
chr6	152762239	152762430	SYNE1	693_selected_genes
chr6	152763184	152763405	SYNE1	693_selected_genes
chr6	152765520	152765738	SYNE1	693_selected_genes
chr6	152768567	152768782	SYNE1	693_selected_genes
chr6	152770642	152770801	SYNE1	693_selected_genes
chr6	152771734	152771993	SYNE1	693_selected_genes
chr6	152772156	152772365	SYNE1	693_selected_genes
chr6	152774584	152774880	SYNE1	693_selected_genes
chr6	152776535	152776794	SYNE1	693_selected_genes
chr6	152776995	152777204	SYNE1	693_selected_genes
chr6	152777777	152777962	SYNE1	693_selected_genes
chr6	152779866	152780090	SYNE1	693_selected_genes
chr6	152782706	152782900	SYNE1	693_selected_genes
chr6	152783832	152784050	SYNE1	693_selected_genes
chr6	152784462	152784677	SYNE1	693_selected_genes
chr6	152786367	152786620	SYNE1	693_selected_genes
chr6	152787091	152787238	SYNE1	693_selected_genes
chr6	152792706	152792925	SYNE1	693_selected_genes
chr6	152793410	152793573	SYNE1	693_selected_genes
chr6	152793680	152793781	SYNE1	693_selected_genes
chr6	152801870	152801941	SYNE1	693_selected_genes
chr6	152804194	152804409	SYNE1	693_selected_genes
chr6	152805943	152806132	SYNE1	693_selected_genes
chr6	152809505	152809663	SYNE1	693_selected_genes
chr6	152819851	152819952	SYNE1	693_selected_genes
chr6	152823742	152823902	SYNE1	693_selected_genes
chr6	152826310	152826557	SYNE1	693_selected_genes
chr6	152831302	152831531	SYNE1	693_selected_genes
chr6	152832120	152832263	SYNE1	693_selected_genes
chr6	152832680	152832751	SYNE1	693_selected_genes
chr6	152841568	152841702	SYNE1	693_selected_genes
chr6	152847189	152847335	SYNE1	693_selected_genes
chr6	152861069	152861181	SYNE1	693_selected_genes
chr6	152949374	152949491	SYNE1	693_selected_genes
chrX	70586139	70586369	TAF1	693_selected_genes
chrX	70587323	70587488	TAF1	693_selected_genes
chrX	70587878	70588045	TAF1	693_selected_genes
chrX	70594991	70595161	TAF1	693_selected_genes
chrX	70596774	70597066	TAF1	693_selected_genes
chrX	70597427	70597696	TAF1	693_selected_genes
chrX	70598059	70598328	TAF1	693_selected_genes
chrX	70598648	70598906	TAF1	693_selected_genes
chrX	70601567	70601794	TAF1	693_selected_genes
chrX	70602360	70602538	TAF1	693_selected_genes
chrX	70602585	70602743	TAF1	693_selected_genes

chrX	70602815	70603039	TAF1	693_selected_genes
chrX	70603786	70604010	TAF1	693_selected_genes
chrX	70604769	70604924	TAF1	693_selected_genes
chrX	70607085	70607336	TAF1	693_selected_genes
chrX	70608061	70608253	TAF1	693_selected_genes
chrX	70608562	70608743	TAF1	693_selected_genes
chrX	70609409	70609540	TAF1	693_selected_genes
chrX	70612393	70612593	TAF1	693_selected_genes
chrX	70612699	70612869	TAF1	693_selected_genes
chrX	70613125	70613351	TAF1	693_selected_genes
chrX	70613891	70614120	TAF1	693_selected_genes
chrX	70617077	70617341	TAF1	693_selected_genes
chrX	70618396	70618612	TAF1	693_selected_genes
chrX	70621352	70621614	TAF1	693_selected_genes
chrX	70626462	70626621	TAF1	693_selected_genes
chrX	70627398	70627547	TAF1	693_selected_genes
chrX	70627798	70628026	TAF1	693_selected_genes
chrX	70636442	70636566	TAF1	693_selected_genes
chrX	70641133	70641251	TAF1	693_selected_genes
chrX	70642941	70643114	TAF1	693_selected_genes
chrX	70643798	70643941	TAF1	693_selected_genes
chrX	70643978	70644113	TAF1	693_selected_genes
chrX	70673994	70674112	TAF1	693_selected_genes
chrX	70674565	70674736	TAF1	693_selected_genes
chrX	70674831	70674887	TAF1	693_selected_genes
chrX	70678063	70678241	TAF1	693_selected_genes
chrX	70678974	70679126	TAF1	693_selected_genes
chrX	70679376	70679583	TAF1	693_selected_genes
chrX	70680450	70680678	TAF1	693_selected_genes
chrX	70683648	70683921	TAF1	693_selected_genes
chrX	70748366	70748600	TAF1	693_selected_genes
chrX	70749537	70749598	TAF1	693_selected_genes
chr6	32813330	32813587	TAP1	693_selected_genes
chr6	32814819	32815006	TAP1	693_selected_genes
chr6	32815264	32815477	TAP1	693_selected_genes
chr6	32815670	32815894	TAP1	693_selected_genes
chr6	32816403	32816642	TAP1	693_selected_genes
chr6	32816741	32816920	TAP1	693_selected_genes
chr6	32818071	32818319	TAP1	693_selected_genes
chr6	32818695	32818951	TAP1	693_selected_genes
chr6	32819860	32820041	TAP1	693_selected_genes
chr6	32820139	32820304	TAP1	693_selected_genes
chr6	32820790	32821618	TAP1	693_selected_genes
chr3	176743260	176743337	TBL1XR1	693_selected_genes
chr3	176744135	176744287	TBL1XR1	693_selected_genes
chr3	176750733	176750949	TBL1XR1	693_selected_genes
chr3	176751960	176752138	TBL1XR1	693_selected_genes
chr3	176755860	176755985	TBL1XR1	693_selected_genes
chr3	176756075	176756247	TBL1XR1	693_selected_genes
chr3	176763891	176764002	TBL1XR1	693_selected_genes
chr3	176765062	176765210	TBL1XR1	693_selected_genes
chr3	176765248	176765362	TBL1XR1	693_selected_genes
chr3	176767759	176767951	TBL1XR1	693_selected_genes
chr3	176768240	176768423	TBL1XR1	693_selected_genes
chr3	176769266	176769539	TBL1XR1	693_selected_genes
chr3	176771535	176771731	TBL1XR1	693_selected_genes
chr3	176782682	176782790	TBL1XR1	693_selected_genes
chr12	115109620	115110132	TBX3	693_selected_genes
chr12	115111944	115112665	TBX3	693_selected_genes
chr12	115114092	115114300	TBX3	693_selected_genes
chr12	115115359	115115486	TBX3	693_selected_genes
chr12	115117284	115117481	TBX3	693_selected_genes
chr12	115117692	115117802	TBX3	693_selected_genes
chr12	115118658	115118976	TBX3	693_selected_genes
chr12	115120591	115121030	TBX3	693_selected_genes
chr15	57212086	57212211	TCF12	693_selected_genes
chr15	57213198	57213321	TCF12	693_selected_genes
chr15	57355922	57356046	TCF12	693_selected_genes
chr15	57364825	57364937	TCF12	693_selected_genes
chr15	57383961	57384114	TCF12	693_selected_genes
chr15	57458574	57458689	TCF12	693_selected_genes
chr15	57484330	57484516	TCF12	693_selected_genes
chr15	57489945	57490048	TCF12	693_selected_genes
chr15	57511694	57511813	TCF12	693_selected_genes
chr15	57523324	57523480	TCF12	693_selected_genes
chr15	57524463	57524653	TCF12	693_selected_genes
chr15	57524884	57525079	TCF12	693_selected_genes
chr15	57526215	57526330	TCF12	693_selected_genes
chr15	57535644	57535773	TCF12	693_selected_genes
chr15	57540238	57540304	TCF12	693_selected_genes
chr15	57543522	57543646	TCF12	693_selected_genes
chr15	57544593	57544715	TCF12	693_selected_genes
chr15	57545434	57545691	TCF12	693_selected_genes
chr15	57554266	57554431	TCF12	693_selected_genes

chr15	57555284	57555497	TCF12	693_selected_genes
chr15	57565202	57565485	TCF12	693_selected_genes
chr15	57574615	57574810	TCF12	693_selected_genes
chr19	1611680	1611873	TCF3	693_selected_genes
chr19	1612180	1612457	TCF3	693_selected_genes
chr19	1615258	1615845	TCF3	693_selected_genes
chr19	1619083	1619258	TCF3	693_selected_genes
chr19	1619289	1619498	TCF3	693_selected_genes
chr19	1619753	1619877	TCF3	693_selected_genes
chr19	1620941	1621071	TCF3	693_selected_genes
chr19	1621106	1621215	TCF3	693_selected_genes
chr19	1621811	1621994	TCF3	693_selected_genes
chr19	1622027	1622247	TCF3	693_selected_genes
chr19	1622286	1622439	TCF3	693_selected_genes
chr19	1623921	1624024	TCF3	693_selected_genes
chr19	1625549	1625732	TCF3	693_selected_genes
chr19	1627332	1627450	TCF3	693_selected_genes
chr19	1631920	1632140	TCF3	693_selected_genes
chr19	1632305	1632429	TCF3	693_selected_genes
chr19	1646328	1646451	TCF3	693_selected_genes
chr19	1650150	1650272	TCF3	693_selected_genes
chr10	114710490	114710729	TCF7L2	693_selected_genes
chr10	114710940	114711057	TCF7L2	693_selected_genes
chr10	114711216	114711391	TCF7L2	693_selected_genes
chr10	114724289	114724408	TCF7L2	693_selected_genes
chr10	114799758	114799910	TCF7L2	693_selected_genes
chr10	114849130	114849324	TCF7L2	693_selected_genes
chr10	114900917	114901100	TCF7L2	693_selected_genes
chr10	114903656	114903809	TCF7L2	693_selected_genes
chr10	114905744	114905885	TCF7L2	693_selected_genes
chr10	114910716	114910907	TCF7L2	693_selected_genes
chr10	114911458	114911668	TCF7L2	693_selected_genes
chr10	114912066	114912224	TCF7L2	693_selected_genes
chr10	114917754	114917853	TCF7L2	693_selected_genes
chr10	114918400	114918501	TCF7L2	693_selected_genes
chr10	114919653	114919776	TCF7L2	693_selected_genes
chr10	114920352	114920475	TCF7L2	693_selected_genes
chr10	114921312	114921369	TCF7L2	693_selected_genes
chr10	114925288	114925756	TCF7L2	693_selected_genes
chr5	1253817	1253971	TERT	693_selected_genes
chr5	1254457	1254645	TERT	693_selected_genes
chr5	1255376	1255551	TERT	693_selected_genes
chr5	1258687	1258799	TERT	693_selected_genes
chr5	1260563	1260740	TERT	693_selected_genes
chr5	1264493	1264732	TERT	693_selected_genes
chr5	1266553	1266675	TERT	693_selected_genes
chr5	1268609	1268773	TERT	693_selected_genes
chr5	1271208	1271344	TERT	693_selected_genes
chr5	1272274	1272420	TERT	693_selected_genes
chr5	1278730	1278936	TERT	693_selected_genes
chr5	1279380	1279610	TERT	693_selected_genes
chr5	1280247	1280478	TERT	693_selected_genes
chr5	1282518	1282764	TERT	693_selected_genes
chr5	1293402	1294806	TERT	693_selected_genes
chr5	1294860	1295129	TERT	693_selected_genes
chr10	70332070	70334034	TET1	693_selected_genes
chr10	70360712	70360816	TET1	693_selected_genes
chr10	70404429	70406787	TET1	693_selected_genes
chr10	70411577	70411718	TET1	693_selected_genes
chr10	70412232	70412376	TET1	693_selected_genes
chr10	70426776	70427038	TET1	693_selected_genes
chr10	70432626	70432827	TET1	693_selected_genes
chr10	70441130	70441270	TET1	693_selected_genes
chr10	70442567	70442755	TET1	693_selected_genes
chr10	70446087	70446489	TET1	693_selected_genes
chr10	70450539	70451596	TET1	693_selected_genes
chr4	106111601	106111668	TET2	693_selected_genes
chr4	106155028	106158622	TET2	693_selected_genes
chr4	106162470	106162615	TET2	693_selected_genes
chr4	106163965	106164109	TET2	693_selected_genes
chr4	106164701	106164960	TET2	693_selected_genes
chr4	106180750	106180951	TET2	693_selected_genes
chr4	106182890	106183030	TET2	693_selected_genes
chr4	106190741	106190929	TET2	693_selected_genes
chr4	106193695	106194100	TET2	693_selected_genes
chr4	106196179	106197701	TET2	693_selected_genes
chr13	114240127	114240189	TFDP1	693_selected_genes
chr13	114265285	114265402	TFDP1	693_selected_genes
chr13	114277469	114277626	TFDP1	693_selected_genes
chr13	114285912	114286084	TFDP1	693_selected_genes
chr13	114287409	114287627	TFDP1	693_selected_genes
chr13	114288179	114288373	TFDP1	693_selected_genes
chr13	114288823	114288942	TFDP1	693_selected_genes
chr13	114290266	114290468	TFDP1	693_selected_genes

chr13	114290823	114291040	TFDP1	693_selected_genes
chr13	114291909	114292236	TFDP1	693_selected_genes
chr13	114294409	114294607	TFDP1	693_selected_genes
chr9	101867462	101867609	TGFBR1	693_selected_genes
chr9	101890190	101890309	TGFBR1	693_selected_genes
chr9	101891092	101891407	TGFBR1	693_selected_genes
chr9	101894753	101895046	TGFBR1	693_selected_genes
chr9	101900115	101900396	TGFBR1	693_selected_genes
chr9	101904792	101905010	TGFBR1	693_selected_genes
chr9	101906988	101907195	TGFBR1	693_selected_genes
chr9	101908741	101908916	TGFBR1	693_selected_genes
chr9	101909910	101910091	TGFBR1	693_selected_genes
chr9	101911436	101911612	TGFBR1	693_selected_genes
chr3	30648350	30648494	TGFBR2	693_selected_genes
chr3	30664665	30664790	TGFBR2	693_selected_genes
chr3	30686213	30686432	TGFBR2	693_selected_genes
chr3	30691736	30691977	TGFBR2	693_selected_genes
chr3	30713104	30713954	TGFBR2	693_selected_genes
chr3	30715571	30715763	TGFBR2	693_selected_genes
chr3	30729850	30730028	TGFBR2	693_selected_genes
chr3	30732886	30733116	TGFBR2	693_selected_genes
chr18	3447712	3447820	TGIF1	693_selected_genes
chr18	3449604	3449679	TGIF1	693_selected_genes
chr18	3450462	3450528	TGIF1	693_selected_genes
chr18	3451952	3452405	TGIF1	693_selected_genes
chr18	3456326	3456759	TGIF1	693_selected_genes
chr18	3457337	3457963	TGIF1	693_selected_genes
chr3	156395461	156396428	TIPARP	693_selected_genes
chr3	156398929	156399003	TIPARP	693_selected_genes
chr3	156411783	156412002	TIPARP	693_selected_genes
chr3	156413628	156413839	TIPARP	693_selected_genes
chr3	156421187	156421516	TIPARP	693_selected_genes
chr3	156422447	156422945	TIPARP	693_selected_genes
chr9	71789263	71789373	TJP2	693_selected_genes
chr9	71820052	71820255	TJP2	693_selected_genes
chr9	71827438	71827542	TJP2	693_selected_genes
chr9	71831229	71831404	TJP2	693_selected_genes
chr9	71833147	71833333	TJP2	693_selected_genes
chr9	71835777	71836437	TJP2	693_selected_genes
chr9	71840194	71840348	TJP2	693_selected_genes
chr9	71840912	71841116	TJP2	693_selected_genes
chr9	71842655	71842814	TJP2	693_selected_genes
chr9	71842871	71843055	TJP2	693_selected_genes
chr9	71844074	71844191	TJP2	693_selected_genes
chr9	71844972	71845173	TJP2	693_selected_genes
chr9	71849329	71849488	TJP2	693_selected_genes
chr9	71850918	71851179	TJP2	693_selected_genes
chr9	71851839	71852077	TJP2	693_selected_genes
chr9	71852768	71852914	TJP2	693_selected_genes
chr9	71853600	71853730	TJP2	693_selected_genes
chr9	71854827	71855088	TJP2	693_selected_genes
chr9	71861580	71861731	TJP2	693_selected_genes
chr9	71862902	71863267	TJP2	693_selected_genes
chr9	71864265	71864426	TJP2	693_selected_genes
chr9	71865925	71866305	TJP2	693_selected_genes
chr9	71867705	71867841	TJP2	693_selected_genes
chr9	71869099	71869315	TJP2	693_selected_genes
chr9	120466725	120466868	TLR4	693_selected_genes
chr9	120470815	120471032	TLR4	693_selected_genes
chr9	120474641	120476951	TLR4	693_selected_genes
chr2	96919520	96919878	TMEM127	693_selected_genes
chr2	96920545	96920760	TMEM127	693_selected_genes
chr2	96930850	96931144	TMEM127	693_selected_genes
chr3	14166668	14166730	TMEM43	693_selected_genes
chr3	14170329	14170568	TMEM43	693_selected_genes
chr3	14170886	14171086	TMEM43	693_selected_genes
chr3	14172296	14172481	TMEM43	693_selected_genes
chr3	14173054	14173199	TMEM43	693_selected_genes
chr3	14174020	14174120	TMEM43	693_selected_genes
chr3	14174340	14174460	TMEM43	693_selected_genes
chr3	14175213	14175334	TMEM43	693_selected_genes
chr3	14176244	14176416	TMEM43	693_selected_genes
chr3	14176632	14176757	TMEM43	693_selected_genes
chr3	14177281	14177433	TMEM43	693_selected_genes
chr3	14180654	14180822	TMEM43	693_selected_genes
chr3	14183067	14183320	TMEM43	693_selected_genes
chr21	42838043	42838105	TMPRSS2	693_selected_genes
chr21	42839635	42839838	TMPRSS2	693_selected_genes
chr21	42840297	42840490	TMPRSS2	693_selected_genes
chr21	42842549	42842695	TMPRSS2	693_selected_genes
chr21	42843707	42843933	TMPRSS2	693_selected_genes
chr21	42845226	42845448	TMPRSS2	693_selected_genes
chr21	42848478	42848572	TMPRSS2	693_selected_genes
chr21	42851073	42851234	TMPRSS2	693_selected_genes

chr21	42852377	42852554	TMPRSS2	693_selected_genes
chr21	42860295	42860465	TMPRSS2	693_selected_genes
chr21	42861408	42861545	TMPRSS2	693_selected_genes
chr21	42866257	42866530	TMPRSS2	693_selected_genes
chr21	42870020	42870141	TMPRSS2	693_selected_genes
chr21	42879851	42879956	TMPRSS2	693_selected_genes
chr6	31543493	31543729	TNF	693_selected_genes
chr6	31544285	31544381	TNF	693_selected_genes
chr6	31544518	31544616	TNF	693_selected_genes
chr6	31544867	31545339	TNF	693_selected_genes
chr6	138192339	138192684	TNFAIP3	693_selected_genes
chr6	138195956	138196197	TNFAIP3	693_selected_genes
chr6	138196799	138196997	TNFAIP3	693_selected_genes
chr6	138197107	138197328	TNFAIP3	693_selected_genes
chr6	138198187	138198418	TNFAIP3	693_selected_genes
chr6	138199543	138200513	TNFAIP3	693_selected_genes
chr6	138201182	138201414	TNFAIP3	693_selected_genes
chr6	138202146	138202481	TNFAIP3	693_selected_genes
chr1	2488078	2488197	TNFRSF14	693_selected_genes
chr1	2489139	2489298	TNFRSF14	693_selected_genes
chr1	2489756	2489932	TNFRSF14	693_selected_genes
chr1	2491236	2491442	TNFRSF14	693_selected_genes
chr1	2492037	2492178	TNFRSF14	693_selected_genes
chr1	2492907	2492988	TNFRSF14	693_selected_genes
chr1	2493086	2493279	TNFRSF14	693_selected_genes
chr1	2494278	2494360	TNFRSF14	693_selected_genes
chr1	2494561	2494737	TNFRSF14	693_selected_genes
chr19	55663176	55663310	TNNI3	693_selected_genes
chr19	55665372	55665599	TNNI3	693_selected_genes
chr19	55666083	55666223	TNNI3	693_selected_genes
chr19	55667543	55667763	TNNI3	693_selected_genes
chr19	55667945	55668070	TNNI3	693_selected_genes
chr19	55668392	55668527	TNNI3	693_selected_genes
chr19	55668638	55668701	TNNI3	693_selected_genes
chr19	55668910	55668982	TNNI3	693_selected_genes
chr1	201328312	201328408	TNNT2	693_selected_genes
chr1	201328725	201328816	TNNT2	693_selected_genes
chr1	201330381	201330522	TNNT2	693_selected_genes
chr1	201331015	201331187	TNNT2	693_selected_genes
chr1	201331212	201331268	TNNT2	693_selected_genes
chr1	201331488	201331547	TNNT2	693_selected_genes
chr1	201332397	201332559	TNNT2	693_selected_genes
chr1	201333400	201333528	TNNT2	693_selected_genes
chr1	201334293	201334460	TNNT2	693_selected_genes
chr1	201334712	201334823	TNNT2	693_selected_genes
chr1	201335940	201336024	TNNT2	693_selected_genes
chr1	201336873	201336959	TNNT2	693_selected_genes
chr1	201337264	201337380	TNNT2	693_selected_genes
chr1	201337520	201337581	TNNT2	693_selected_genes
chr1	201338435	201338495	TNNT2	693_selected_genes
chr1	201338918	201338999	TNNT2	693_selected_genes
chr1	201341129	201341198	TNNT2	693_selected_genes
chr1	201341247	201341308	TNNT2	693_selected_genes
chr1	201342186	201342246	TNNT2	693_selected_genes
chr1	201342299	201342407	TNNT2	693_selected_genes
chr5	72112539	72112604	TNPO1	693_selected_genes
chr5	72144186	72144350	TNPO1	693_selected_genes
chr5	72147045	72147171	TNPO1	693_selected_genes
chr5	72151575	72151775	TNPO1	693_selected_genes
chr5	72157609	72157766	TNPO1	693_selected_genes
chr5	72161397	72161581	TNPO1	693_selected_genes
chr5	72168440	72168572	TNPO1	693_selected_genes
chr5	72171416	72171589	TNPO1	693_selected_genes
chr5	72173029	72173198	TNPO1	693_selected_genes
chr5	72178268	72178379	TNPO1	693_selected_genes
chr5	72178865	72179084	TNPO1	693_selected_genes
chr5	72182871	72183074	TNPO1	693_selected_genes
chr5	72183879	72184155	TNPO1	693_selected_genes
chr5	72185587	72185809	TNPO1	693_selected_genes
chr5	72187611	72187748	TNPO1	693_selected_genes
chr5	72188940	72189098	TNPO1	693_selected_genes
chr5	72189178	72189387	TNPO1	693_selected_genes
chr5	72189417	72189555	TNPO1	693_selected_genes
chr5	72192259	72192408	TNPO1	693_selected_genes
chr5	72192857	72193003	TNPO1	693_selected_genes
chr5	72195807	72195933	TNPO1	693_selected_genes
chr5	72196775	72196925	TNPO1	693_selected_genes
chr5	72199510	72199635	TNPO1	693_selected_genes
chr5	72201104	72201262	TNPO1	693_selected_genes
chr5	72204661	72204792	TNPO1	693_selected_genes
chr22	35695896	35695998	TOM1	693_selected_genes
chr22	35713844	35713979	TOM1	693_selected_genes
chr22	35717709	35717832	TOM1	693_selected_genes
chr22	35717926	35718055	TOM1	693_selected_genes

chr22	35718966	35719195	TOM1	693_selected_genes
chr22	35719463	35719648	TOM1	693_selected_genes
chr22	35719735	35719932	TOM1	693_selected_genes
chr22	35723238	35723405	TOM1	693_selected_genes
chr22	35726314	35726498	TOM1	693_selected_genes
chr22	35727263	35727413	TOM1	693_selected_genes
chr22	35728948	35729032	TOM1	693_selected_genes
chr22	35729371	35729515	TOM1	693_selected_genes
chr22	35730295	35730466	TOM1	693_selected_genes
chr22	35734680	35734806	TOM1	693_selected_genes
chr22	35741692	35741802	TOM1	693_selected_genes
chr22	35742897	35742990	TOM1	693_selected_genes
chr22	35743022	35743227	TOM1	693_selected_genes
chr20	39657682	39657765	TOP1	693_selected_genes
chr20	39658045	39658120	TOP1	693_selected_genes
chr20	39690008	39690155	TOP1	693_selected_genes
chr20	39704785	39704959	TOP1	693_selected_genes
chr20	39706196	39706302	TOP1	693_selected_genes
chr20	39708699	39708845	TOP1	693_selected_genes
chr20	39709779	39709905	TOP1	693_selected_genes
chr20	39713076	39713233	TOP1	693_selected_genes
chr20	39721086	39721252	TOP1	693_selected_genes
chr20	39725834	39726006	TOP1	693_selected_genes
chr20	39726829	39727002	TOP1	693_selected_genes
chr20	39728670	39728908	TOP1	693_selected_genes
chr20	39729823	39730018	TOP1	693_selected_genes
chr20	39741396	39741590	TOP1	693_selected_genes
chr20	39742584	39742820	TOP1	693_selected_genes
chr20	39743985	39744104	TOP1	693_selected_genes
chr20	39744892	39745057	TOP1	693_selected_genes
chr20	39746783	39746961	TOP1	693_selected_genes
chr20	39750310	39750455	TOP1	693_selected_genes
chr20	39750620	39750820	TOP1	693_selected_genes
chr20	39751809	39751962	TOP1	693_selected_genes
chr17	38545745	38545924	TOP2A	693_selected_genes
chr17	38546191	38546441	TOP2A	693_selected_genes
chr17	38547732	38547917	TOP2A	693_selected_genes
chr17	38548294	38548388	TOP2A	693_selected_genes
chr17	38548444	38548618	TOP2A	693_selected_genes
chr17	38548811	38549014	TOP2A	693_selected_genes
chr17	38551675	38551816	TOP2A	693_selected_genes
chr17	38552509	38552742	TOP2A	693_selected_genes
chr17	38554784	38554918	TOP2A	693_selected_genes
chr17	38554999	38555214	TOP2A	693_selected_genes
chr17	38555266	38555408	TOP2A	693_selected_genes
chr17	38556098	38556344	TOP2A	693_selected_genes
chr17	38556435	38556686	TOP2A	693_selected_genes
chr17	38556755	38556940	TOP2A	693_selected_genes
chr17	38557076	38557358	TOP2A	693_selected_genes
chr17	38559132	38559331	TOP2A	693_selected_genes
chr17	38560378	38560550	TOP2A	693_selected_genes
chr17	38560603	38560768	TOP2A	693_selected_genes
chr17	38561017	38561160	TOP2A	693_selected_genes
chr17	38562610	38562770	TOP2A	693_selected_genes
chr17	38562810	38562966	TOP2A	693_selected_genes
chr17	38563058	38563219	TOP2A	693_selected_genes
chr17	38563775	38563951	TOP2A	693_selected_genes
chr17	38564193	38564401	TOP2A	693_selected_genes
chr17	38564718	38564907	TOP2A	693_selected_genes
chr17	38567335	38567523	TOP2A	693_selected_genes
chr17	38567580	38567732	TOP2A	693_selected_genes
chr17	38567871	38568095	TOP2A	693_selected_genes
chr17	38568985	38569248	TOP2A	693_selected_genes
chr17	38569421	38569569	TOP2A	693_selected_genes
chr17	38569706	38569902	TOP2A	693_selected_genes
chr17	38572231	38572345	TOP2A	693_selected_genes
chr17	38572648	38572789	TOP2A	693_selected_genes
chr17	38572966	38573172	TOP2A	693_selected_genes
chr17	38573997	38574068	TOP2A	693_selected_genes
chr17	7565231	7565357	TP53	693_selected_genes
chr17	7569498	7569587	TP53	693_selected_genes
chr17	7572901	7573033	TP53	693_selected_genes
chr17	7573901	7574058	TP53	693_selected_genes
chr17	7576511	7576682	TP53	693_selected_genes
chr17	7576827	7576951	TP53	693_selected_genes
chr17	7576993	7577180	TP53	693_selected_genes
chr17	7577473	7577633	TP53	693_selected_genes
chr17	7578151	7578314	TP53	693_selected_genes
chr17	7578345	7578579	TP53	693_selected_genes
chr17	7579286	7579615	TP53	693_selected_genes
chr17	7579674	7579746	TP53	693_selected_genes
chr17	7579813	7579937	TP53	693_selected_genes
chr15	43699555	43699793	TP53BP1	693_selected_genes
chr15	43700115	43700311	TP53BP1	693_selected_genes

chr15	43701069	43701319	TP53BP1	693_selected_genes
chr15	43701819	43701964	TP53BP1	693_selected_genes
chr15	43704844	43704990	TP53BP1	693_selected_genes
chr15	43705291	43705557	TP53BP1	693_selected_genes
chr15	43706275	43706339	TP53BP1	693_selected_genes
chr15	43707766	43708032	TP53BP1	693_selected_genes
chr15	43708397	43708639	TP53BP1	693_selected_genes
chr15	43712477	43712958	TP53BP1	693_selected_genes
chr15	43713197	43713397	TP53BP1	693_selected_genes
chr15	43713966	43714349	TP53BP1	693_selected_genes
chr15	43720188	43720391	TP53BP1	693_selected_genes
chr15	43724366	43724900	TP53BP1	693_selected_genes
chr15	43730496	43730639	TP53BP1	693_selected_genes
chr15	43733698	43733806	TP53BP1	693_selected_genes
chr15	43738559	43738813	TP53BP1	693_selected_genes
chr15	43739070	43739137	TP53BP1	693_selected_genes
chr15	43739530	43739708	TP53BP1	693_selected_genes
chr15	43748064	43749441	TP53BP1	693_selected_genes
chr15	43762030	43762289	TP53BP1	693_selected_genes
chr15	43766845	43766990	TP53BP1	693_selected_genes
chr15	43767737	43767917	TP53BP1	693_selected_genes
chr15	43769765	43769982	TP53BP1	693_selected_genes
chr15	43771569	43771749	TP53BP1	693_selected_genes
chr15	43772031	43772240	TP53BP1	693_selected_genes
chr15	43773067	43773245	TP53BP1	693_selected_genes
chr15	43783841	43783976	TP53BP1	693_selected_genes
chr15	43784174	43784318	TP53BP1	693_selected_genes
chr15	43784456	43784691	TP53BP1	693_selected_genes
chr15	43785209	43785266	TP53BP1	693_selected_genes
chr3	189349279	189349391	TP63	693_selected_genes
chr3	189455503	189455682	TP63	693_selected_genes
chr3	189456405	189456588	TP63	693_selected_genes
chr3	189507564	189507656	TP63	693_selected_genes
chr3	189526035	189526340	TP63	693_selected_genes
chr3	189561960	189562226	TP63	693_selected_genes
chr3	189581995	189582232	TP63	693_selected_genes
chr3	189584445	189584611	TP63	693_selected_genes
chr3	189585596	189585756	TP63	693_selected_genes
chr3	189586343	189586530	TP63	693_selected_genes
chr3	189587087	189587220	TP63	693_selected_genes
chr3	189590622	189590809	TP63	693_selected_genes
chr3	189597827	189597992	TP63	693_selected_genes
chr3	189604157	189604365	TP63	693_selected_genes
chr3	189607103	189607298	TP63	693_selected_genes
chr3	189608552	189608696	TP63	693_selected_genes
chr3	189611969	189612316	TP63	693_selected_genes
chr6	18130873	18131036	TPMT	693_selected_genes
chr6	18132338	18132433	TPMT	693_selected_genes
chr6	18134009	18134145	TPMT	693_selected_genes
chr6	18139168	18139293	TPMT	693_selected_genes
chr6	18139870	18139973	TPMT	693_selected_genes
chr6	18143801	18143984	TPMT	693_selected_genes
chr6	18148028	18148171	TPMT	693_selected_genes
chr6	18149193	18149383	TPMT	693_selected_genes
chr20	30345254	30345410	TPX2	693_selected_genes
chr20	30347834	30348007	TPX2	693_selected_genes
chr20	30354333	30354510	TPX2	693_selected_genes
chr20	30358120	30358299	TPX2	693_selected_genes
chr20	30359337	30359510	TPX2	693_selected_genes
chr20	30363644	30363816	TPX2	693_selected_genes
chr20	30365264	30365466	TPX2	693_selected_genes
chr20	30366590	30366812	TPX2	693_selected_genes
chr20	30368716	30368874	TPX2	693_selected_genes
chr20	30370026	30370218	TPX2	693_selected_genes
chr20	30371482	30371749	TPX2	693_selected_genes
chr20	30380512	30380658	TPX2	693_selected_genes
chr20	30381625	30381852	TPX2	693_selected_genes
chr20	30382179	30382376	TPX2	693_selected_genes
chr20	30385181	30385343	TPX2	693_selected_genes
chr20	30386142	30386380	TPX2	693_selected_genes
chr20	30388747	30388908	TPX2	693_selected_genes
chr14	103336513	103336808	TRAF3	693_selected_genes
chr14	103338228	103338330	TRAF3	693_selected_genes
chr14	103341935	103342090	TRAF3	693_selected_genes
chr14	103342669	103342887	TRAF3	693_selected_genes
chr14	103352500	103352631	TRAF3	693_selected_genes
chr14	103355871	103355996	TRAF3	693_selected_genes
chr14	103357636	103357779	TRAF3	693_selected_genes
chr14	103361384	103361494	TRAF3	693_selected_genes
chr14	103363572	103363763	TRAF3	693_selected_genes
chr14	103369566	103369791	TRAF3	693_selected_genes
chr14	103371524	103372146	TRAF3	693_selected_genes
chr16	2213896	2214027	TRAF7	693_selected_genes
chr16	2215832	2215962	TRAF7	693_selected_genes

chr16	2218052	2218194	TRAF7	693_selected_genes
chr16	2220589	2220756	TRAF7	693_selected_genes
chr16	2221239	2221382	TRAF7	693_selected_genes
chr16	2221547	2221631	TRAF7	693_selected_genes
chr16	2222166	2222400	TRAF7	693_selected_genes
chr16	2222440	2222625	TRAF7	693_selected_genes
chr16	2223157	2223425	TRAF7	693_selected_genes
chr16	2223456	2223580	TRAF7	693_selected_genes
chr16	2223763	2223862	TRAF7	693_selected_genes
chr16	2223896	2224074	TRAF7	693_selected_genes
chr16	2224226	2224359	TRAF7	693_selected_genes
chr16	2225086	2225176	TRAF7	693_selected_genes
chr16	2225276	2225443	TRAF7	693_selected_genes
chr16	2225475	2225648	TRAF7	693_selected_genes
chr16	2225809	2225979	TRAF7	693_selected_genes
chr16	2226024	2226206	TRAF7	693_selected_genes
chr16	2226240	2226410	TRAF7	693_selected_genes
chr16	2226535	2226600	TRAF7	693_selected_genes
chr5	14143809	14144016	TRIO	693_selected_genes
chr5	14184033	14184093	TRIO	693_selected_genes
chr5	14270908	14271033	TRIO	693_selected_genes
chr5	14280405	14280570	TRIO	693_selected_genes
chr5	14286954	14287197	TRIO	693_selected_genes
chr5	14290799	14291362	TRIO	693_selected_genes
chr5	14293095	14293268	TRIO	693_selected_genes
chr5	14297155	14297397	TRIO	693_selected_genes
chr5	14304544	14304726	TRIO	693_selected_genes
chr5	14316596	14316877	TRIO	693_selected_genes
chr5	14330861	14331034	TRIO	693_selected_genes
chr5	14336619	14336861	TRIO	693_selected_genes
chr5	14358261	14358481	TRIO	693_selected_genes
chr5	14359440	14359665	TRIO	693_selected_genes
chr5	14363815	14364061	TRIO	693_selected_genes
chr5	14364733	14364950	TRIO	693_selected_genes
chr5	14366943	14367113	TRIO	693_selected_genes
chr5	14368791	14369033	TRIO	693_selected_genes
chr5	14369457	14369657	TRIO	693_selected_genes
chr5	14374312	14374477	TRIO	693_selected_genes
chr5	14378095	14378261	TRIO	693_selected_genes
chr5	14381213	14381386	TRIO	693_selected_genes
chr5	14387521	14387766	TRIO	693_selected_genes
chr5	14387815	14387981	TRIO	693_selected_genes
chr5	14388696	14388813	TRIO	693_selected_genes
chr5	14389372	14389532	TRIO	693_selected_genes
chr5	14390314	14390434	TRIO	693_selected_genes
chr5	14390984	14391124	TRIO	693_selected_genes
chr5	14394121	14394264	TRIO	693_selected_genes
chr5	14397126	14397288	TRIO	693_selected_genes
chr5	14398963	14399264	TRIO	693_selected_genes
chr5	14401046	14401198	TRIO	693_selected_genes
chr5	14405931	14406124	TRIO	693_selected_genes
chr5	14406656	14406806	TRIO	693_selected_genes
chr5	14419861	14420155	TRIO	693_selected_genes
chr5	14461102	14461445	TRIO	693_selected_genes
chr5	14462838	14463059	TRIO	693_selected_genes
chr5	14465628	14465774	TRIO	693_selected_genes
chr5	14471401	14471600	TRIO	693_selected_genes
chr5	14472675	14472792	TRIO	693_selected_genes
chr5	14474077	14474231	TRIO	693_selected_genes
chr5	14476977	14477097	TRIO	693_selected_genes
chr5	14479344	14479484	TRIO	693_selected_genes
chr5	14480002	14480145	TRIO	693_selected_genes
chr5	14481317	14481418	TRIO	693_selected_genes
chr5	14481624	14481752	TRIO	693_selected_genes
chr5	14482665	14482907	TRIO	693_selected_genes
chr5	14485152	14485380	TRIO	693_selected_genes
chr5	14487547	14488394	TRIO	693_selected_genes
chr5	14489024	14489203	TRIO	693_selected_genes
chr5	14490902	14490954	TRIO	693_selected_genes
chr5	14492650	14492948	TRIO	693_selected_genes
chr5	14496962	14497151	TRIO	693_selected_genes
chr5	14497930	14498008	TRIO	693_selected_genes
chr5	14498172	14498385	TRIO	693_selected_genes
chr5	14498602	14498774	TRIO	693_selected_genes
chr5	14502662	14502791	TRIO	693_selected_genes
chr5	14504476	14504727	TRIO	693_selected_genes
chr5	14507205	14507394	TRIO	693_selected_genes
chr5	14507963	14508556	TRIO	693_selected_genes
chr5	14531827	14531908	TRIO	693_selected_genes
chr7	98478748	98478898	TRRAP	693_selected_genes
chr7	98479572	98479672	TRRAP	693_selected_genes
chr7	98487932	98488093	TRRAP	693_selected_genes
chr7	98490021	98490176	TRRAP	693_selected_genes
chr7	98491395	98491529	TRRAP	693_selected_genes

chr7	98493361	98493468	TRRAP	693_selected_genes
chr7	98495338	98495514	TRRAP	693_selected_genes
chr7	98497019	98497147	TRRAP	693_selected_genes
chr7	98497276	98497415	TRRAP	693_selected_genes
chr7	98498221	98498368	TRRAP	693_selected_genes
chr7	98500976	98501165	TRRAP	693_selected_genes
chr7	98503774	98503903	TRRAP	693_selected_genes
chr7	98506325	98506610	TRRAP	693_selected_genes
chr7	98507653	98508067	TRRAP	693_selected_genes
chr7	98508107	98508255	TRRAP	693_selected_genes
chr7	98508674	98508919	TRRAP	693_selected_genes
chr7	98509619	98509861	TRRAP	693_selected_genes
chr7	98513320	98513536	TRRAP	693_selected_genes
chr7	98515020	98515327	TRRAP	693_selected_genes
chr7	98519350	98519601	TRRAP	693_selected_genes
chr7	98522709	98522911	TRRAP	693_selected_genes
chr7	98524764	98525014	TRRAP	693_selected_genes
chr7	98527586	98527854	TRRAP	693_selected_genes
chr7	98528230	98528478	TRRAP	693_selected_genes
chr7	98529002	98529313	TRRAP	693_selected_genes
chr7	98530838	98531050	TRRAP	693_selected_genes
chr7	98533176	98533323	TRRAP	693_selected_genes
chr7	98534753	98534925	TRRAP	693_selected_genes
chr7	98535247	98535468	TRRAP	693_selected_genes
chr7	98540546	98540665	TRRAP	693_selected_genes
chr7	98543344	98543448	TRRAP	693_selected_genes
chr7	98543527	98543598	TRRAP	693_selected_genes
chr7	98545818	98545988	TRRAP	693_selected_genes
chr7	98546163	98546333	TRRAP	693_selected_genes
chr7	98547014	98547229	TRRAP	693_selected_genes
chr7	98547257	98547489	TRRAP	693_selected_genes
chr7	98547661	98547910	TRRAP	693_selected_genes
chr7	98548473	98548652	TRRAP	693_selected_genes
chr7	98550764	98551081	TRRAP	693_selected_genes
chr7	98552695	98552952	TRRAP	693_selected_genes
chr7	98553743	98553952	TRRAP	693_selected_genes
chr7	98553996	98554181	TRRAP	693_selected_genes
chr7	98555578	98555739	TRRAP	693_selected_genes
chr7	98556941	98557138	TRRAP	693_selected_genes
chr7	98558858	98559122	TRRAP	693_selected_genes
chr7	98559899	98560075	TRRAP	693_selected_genes
chr7	98562226	98562423	TRRAP	693_selected_genes
chr7	98563290	98563543	TRRAP	693_selected_genes
chr7	98564638	98564810	TRRAP	693_selected_genes
chr7	98565082	98565346	TRRAP	693_selected_genes
chr7	98567709	98567939	TRRAP	693_selected_genes
chr7	98569396	98569593	TRRAP	693_selected_genes
chr7	98573746	98573916	TRRAP	693_selected_genes
chr7	98574080	98574418	TRRAP	693_selected_genes
chr7	98574536	98574724	TRRAP	693_selected_genes
chr7	98575808	98575971	TRRAP	693_selected_genes
chr7	98576366	98576552	TRRAP	693_selected_genes
chr7	98579366	98579608	TRRAP	693_selected_genes
chr7	98580861	98581140	TRRAP	693_selected_genes
chr7	98581690	98582006	TRRAP	693_selected_genes
chr7	98582541	98582692	TRRAP	693_selected_genes
chr7	98586362	98586614	TRRAP	693_selected_genes
chr7	98588052	98588267	TRRAP	693_selected_genes
chr7	98589734	98589875	TRRAP	693_selected_genes
chr7	98591135	98591385	TRRAP	693_selected_genes
chr7	98592184	98592496	TRRAP	693_selected_genes
chr7	98601787	98602063	TRRAP	693_selected_genes
chr7	98602728	98602996	TRRAP	693_selected_genes
chr7	98605974	98606209	TRRAP	693_selected_genes
chr7	98608649	98608903	TRRAP	693_selected_genes
chr7	98608938	98609183	TRRAP	693_selected_genes
chr7	98609668	98610003	TRRAP	693_selected_genes
chr9	135771596	135772166	TSC1	693_selected_genes
chr9	135772545	135772757	TSC1	693_selected_genes
chr9	135772784	135773022	TSC1	693_selected_genes
chr9	135776076	135776249	TSC1	693_selected_genes
chr9	135776950	135777111	TSC1	693_selected_genes
chr9	135777966	135778199	TSC1	693_selected_genes
chr9	135779012	135779229	TSC1	693_selected_genes
chr9	135779772	135779866	TSC1	693_selected_genes
chr9	135780942	135781551	TSC1	693_selected_genes
chr9	135782092	135782247	TSC1	693_selected_genes
chr9	135782662	135782782	TSC1	693_selected_genes
chr9	135785932	135786104	TSC1	693_selected_genes
chr9	135786363	135786525	TSC1	693_selected_genes
chr9	135786742	135786980	TSC1	693_selected_genes
chr9	135787643	135787869	TSC1	693_selected_genes
chr9	135796724	135796848	TSC1	693_selected_genes
chr9	135797180	135797385	TSC1	693_selected_genes

chr9	135798709	135798904	TSC1	693_selected_genes
chr9	135800948	135801151	TSC1	693_selected_genes
chr9	135802562	135802716	TSC1	693_selected_genes
chr9	135804128	135804284	TSC1	693_selected_genes
chr16	2098245	2098299	TSC2	693_selected_genes
chr16	2098562	2098779	TSC2	693_selected_genes
chr16	2100375	2100512	TSC2	693_selected_genes
chr16	2103317	2103568	TSC2	693_selected_genes
chr16	2104271	2104466	TSC2	693_selected_genes
chr16	2105377	2105546	TSC2	693_selected_genes
chr16	2106136	2106270	TSC2	693_selected_genes
chr16	2106619	2106795	TSC2	693_selected_genes
chr16	2107080	2107204	TSC2	693_selected_genes
chr16	2108722	2108899	TSC2	693_selected_genes
chr16	2110645	2110839	TSC2	693_selected_genes
chr16	2111846	2112034	TSC2	693_selected_genes
chr16	2112472	2112626	TSC2	693_selected_genes
chr16	2112947	2113079	TSC2	693_selected_genes
chr16	2114247	2114453	TSC2	693_selected_genes
chr16	2115494	2115661	TSC2	693_selected_genes
chr16	2120431	2120604	TSC2	693_selected_genes
chr16	2121485	2121642	TSC2	693_selected_genes
chr16	2121759	2121960	TSC2	693_selected_genes
chr16	2122216	2122389	TSC2	693_selected_genes
chr16	2122824	2123009	TSC2	693_selected_genes
chr16	2124175	2124415	TSC2	693_selected_genes
chr16	2125774	2125918	TSC2	693_selected_genes
chr16	2126043	2126280	TSC2	693_selected_genes
chr16	2126466	2126611	TSC2	693_selected_genes
chr16	2127573	2127752	TSC2	693_selected_genes
chr16	2129007	2129222	TSC2	693_selected_genes
chr16	2129251	2129454	TSC2	693_selected_genes
chr16	2129532	2129695	TSC2	693_selected_genes
chr16	2130140	2130403	TSC2	693_selected_genes
chr16	2131570	2131824	TSC2	693_selected_genes
chr16	2132411	2132530	TSC2	693_selected_genes
chr16	2133165	2133280	TSC2	693_selected_genes
chr16	2133670	2133842	TSC2	693_selected_genes
chr16	2134203	2134741	TSC2	693_selected_genes
chr16	2134926	2135052	TSC2	693_selected_genes
chr16	2135205	2135348	TSC2	693_selected_genes
chr16	2136168	2136405	TSC2	693_selected_genes
chr16	2136707	2136897	TSC2	693_selected_genes
chr16	2137838	2137967	TSC2	693_selected_genes
chr16	2138023	2138165	TSC2	693_selected_genes
chr16	2138202	2138351	TSC2	693_selected_genes
chr16	2138421	2138636	TSC2	693_selected_genes
chr14	81421999	81422219	TSHR	693_selected_genes
chr14	81528466	81528588	TSHR	693_selected_genes
chr14	81534572	81534697	TSHR	693_selected_genes
chr14	81554223	81554397	TSHR	693_selected_genes
chr14	81557387	81557512	TSHR	693_selected_genes
chr14	81558849	81558977	TSHR	693_selected_genes
chr14	81562957	81563076	TSHR	693_selected_genes
chr14	81574693	81574825	TSHR	693_selected_genes
chr14	81574867	81575050	TSHR	693_selected_genes
chr14	81605997	81606236	TSHR	693_selected_genes
chr14	81609258	81610722	TSHR	693_selected_genes
chr6	80715535	80715724	TTK	693_selected_genes
chr6	80715796	80715917	TTK	693_selected_genes
chr6	80717500	80717773	TTK	693_selected_genes
chr6	80718077	80718234	TTK	693_selected_genes
chr6	80720505	80720699	TTK	693_selected_genes
chr6	80721125	80721290	TTK	693_selected_genes
chr6	80721420	80721543	TTK	693_selected_genes
chr6	80721577	80721722	TTK	693_selected_genes
chr6	80722970	80723108	TTK	693_selected_genes
chr6	80724154	80724328	TTK	693_selected_genes
chr6	80732015	80732214	TTK	693_selected_genes
chr6	80736069	80736256	TTK	693_selected_genes
chr6	80737576	80737753	TTK	693_selected_genes
chr6	80741158	80741301	TTK	693_selected_genes
chr6	80744676	80744884	TTK	693_selected_genes
chr6	80744957	80745159	TTK	693_selected_genes
chr6	80746166	80746341	TTK	693_selected_genes
chr6	80747658	80747789	TTK	693_selected_genes
chr6	80749387	80749614	TTK	693_selected_genes
chr6	80749887	80750022	TTK	693_selected_genes
chr6	80750297	80750445	TTK	693_selected_genes
chr6	80751810	80751944	TTK	693_selected_genes
chr1	145438777	145439077	TXNIP	693_selected_genes
chr1	145439482	145439690	TXNIP	693_selected_genes
chr1	145439752	145439950	TXNIP	693_selected_genes
chr1	145440012	145440165	TXNIP	693_selected_genes

chr1	145440243	145440550	TXNIP	693_selected_genes
chr1	145440606	145440813	TXNIP	693_selected_genes
chr1	145440876	145441078	TXNIP	693_selected_genes
chr1	145441157	145441243	TXNIP	693_selected_genes
chr18	657717	657972	TYMS	693_selected_genes
chr18	659615	659739	TYMS	693_selected_genes
chr18	662120	662345	TYMS	693_selected_genes
chr18	669046	669198	TYMS	693_selected_genes
chr18	670666	670892	TYMS	693_selected_genes
chr18	671354	671476	TYMS	693_selected_genes
chr18	672834	673022	TYMS	693_selected_genes
chr21	44513186	44513384	U2AF1	693_selected_genes
chr21	44514555	44514698	U2AF1	693_selected_genes
chr21	44514739	44514923	U2AF1	693_selected_genes
chr21	44515522	44515671	U2AF1	693_selected_genes
chr21	44515778	44515878	U2AF1	693_selected_genes
chr21	44520537	44520654	U2AF1	693_selected_genes
chr21	44521450	44521567	U2AF1	693_selected_genes
chr21	44524399	44524537	U2AF1	693_selected_genes
chr21	44527535	44527629	U2AF1	693_selected_genes
chr8	103266504	103266767	UBR5	693_selected_genes
chr8	103269834	103269970	UBR5	693_selected_genes
chr8	103271187	103271382	UBR5	693_selected_genes
chr8	103273348	103273512	UBR5	693_selected_genes
chr8	103274117	103274323	UBR5	693_selected_genes
chr8	103276676	103276811	UBR5	693_selected_genes
chr8	103277302	103277531	UBR5	693_selected_genes
chr8	103279149	103279260	UBR5	693_selected_genes
chr8	103281180	103281361	UBR5	693_selected_genes
chr8	103282241	103282436	UBR5	693_selected_genes
chr8	103283336	103283498	UBR5	693_selected_genes
chr8	103284731	103285025	UBR5	693_selected_genes
chr8	103287723	103287872	UBR5	693_selected_genes
chr8	103287910	103288081	UBR5	693_selected_genes
chr8	103289174	103289445	UBR5	693_selected_genes
chr8	103291028	103291192	UBR5	693_selected_genes
chr8	103291238	103291461	UBR5	693_selected_genes
chr8	103292596	103292720	UBR5	693_selected_genes
chr8	103293491	103293772	UBR5	693_selected_genes
chr8	103297329	103297591	UBR5	693_selected_genes
chr8	103297715	103298034	UBR5	693_selected_genes
chr8	103298562	103298870	UBR5	693_selected_genes
chr8	103299635	103299817	UBR5	693_selected_genes
chr8	103300357	103300519	UBR5	693_selected_genes
chr8	103301655	103301829	UBR5	693_selected_genes
chr8	103305807	103306097	UBR5	693_selected_genes
chr8	103306157	103306368	UBR5	693_selected_genes
chr8	103307184	103307364	UBR5	693_selected_genes
chr8	103307406	103307583	UBR5	693_selected_genes
chr8	103307616	103307785	UBR5	693_selected_genes
chr8	103307838	103308035	UBR5	693_selected_genes
chr8	103309095	103309237	UBR5	693_selected_genes
chr8	103309660	103309859	UBR5	693_selected_genes
chr8	103310594	103310742	UBR5	693_selected_genes
chr8	103311050	103311224	UBR5	693_selected_genes
chr8	103311654	103311800	UBR5	693_selected_genes
chr8	103312202	103312404	UBR5	693_selected_genes
chr8	103316245	103316422	UBR5	693_selected_genes
chr8	103317287	103317548	UBR5	693_selected_genes
chr8	103323501	103323749	UBR5	693_selected_genes
chr8	103323937	103324142	UBR5	693_selected_genes
chr8	103324336	103324498	UBR5	693_selected_genes
chr8	103324544	103324738	UBR5	693_selected_genes
chr8	103326006	103326169	UBR5	693_selected_genes
chr8	103326946	103327106	UBR5	693_selected_genes
chr8	103335513	103335748	UBR5	693_selected_genes
chr8	103338748	103338916	UBR5	693_selected_genes
chr8	103339944	103340149	UBR5	693_selected_genes
chr8	103341292	103341453	UBR5	693_selected_genes
chr8	103341485	103341652	UBR5	693_selected_genes
chr8	103354675	103354937	UBR5	693_selected_genes
chr8	103357598	103357798	UBR5	693_selected_genes
chr8	103358438	103358648	UBR5	693_selected_genes
chr8	103359105	103359345	UBR5	693_selected_genes
chr8	103372273	103372443	UBR5	693_selected_genes
chr8	103372791	103372918	UBR5	693_selected_genes
chr8	103373327	103373459	UBR5	693_selected_genes
chr8	103373784	103373879	UBR5	693_selected_genes
chr8	103424375	103424487	UBR5	693_selected_genes
chr2	234526328	234527233	UGT1A1	693_selected_genes
chr2	234580555	234581460	UGT1A1	693_selected_genes
chr2	234621612	234622529	UGT1A1	693_selected_genes
chr2	234637747	234638664	UGT1A1	693_selected_genes
chr2	234668908	234669822	UGT1A1	693_selected_genes

chr2	234675654	234675836	UGT1A1	693_selected_genes
chr2	234676469	234676607	UGT1A1	693_selected_genes
chr2	234676840	234677110	UGT1A1	693_selected_genes
chr2	234678151	234678232	UGT1A1	693_selected_genes
chr2	234680882	234681230	UGT1A1	693_selected_genes
chrX	118968815	118969015	UPF3B	693_selected_genes
chrX	118971694	118972039	UPF3B	693_selected_genes
chrX	118972304	118972515	UPF3B	693_selected_genes
chrX	118974583	118974672	UPF3B	693_selected_genes
chrX	118975013	118975246	UPF3B	693_selected_genes
chrX	118975672	118975766	UPF3B	693_selected_genes
chrX	118977128	118977289	UPF3B	693_selected_genes
chrX	118979135	118979284	UPF3B	693_selected_genes
chrX	118985432	118985589	UPF3B	693_selected_genes
chrX	118985704	118985861	UPF3B	693_selected_genes
chrX	118986710	118986916	UPF3B	693_selected_genes
chrX	40982856	40983002	USP9X	693_selected_genes
chrX	40988227	40988423	USP9X	693_selected_genes
chrX	40990684	40990814	USP9X	693_selected_genes
chrX	40993952	40994115	USP9X	693_selected_genes
chrX	40996031	40996300	USP9X	693_selected_genes
chrX	40999883	41000049	USP9X	693_selected_genes
chrX	41000193	41000495	USP9X	693_selected_genes
chrX	41000520	41000709	USP9X	693_selected_genes
chrX	41002518	41002721	USP9X	693_selected_genes
chrX	41003749	41003904	USP9X	693_selected_genes
chrX	41007596	41007853	USP9X	693_selected_genes
chrX	41010148	41010335	USP9X	693_selected_genes
chrX	41012175	41012359	USP9X	693_selected_genes
chrX	41022017	41022155	USP9X	693_selected_genes
chrX	41025099	41025492	USP9X	693_selected_genes
chrX	41026709	41026855	USP9X	693_selected_genes
chrX	41027234	41027496	USP9X	693_selected_genes
chrX	41029222	41029513	USP9X	693_selected_genes
chrX	41029697	41029897	USP9X	693_selected_genes
chrX	41030885	41030965	USP9X	693_selected_genes
chrX	41031065	41031236	USP9X	693_selected_genes
chrX	41043225	41043406	USP9X	693_selected_genes
chrX	41043624	41043953	USP9X	693_selected_genes
chrX	41045744	41045920	USP9X	693_selected_genes
chrX	41047219	41047395	USP9X	693_selected_genes
chrX	41048536	41048753	USP9X	693_selected_genes
chrX	41055132	41055283	USP9X	693_selected_genes
chrX	41055478	41055637	USP9X	693_selected_genes
chrX	41055819	41056016	USP9X	693_selected_genes
chrX	41056591	41056788	USP9X	693_selected_genes
chrX	41057755	41058028	USP9X	693_selected_genes
chrX	41060287	41060558	USP9X	693_selected_genes
chrX	41064530	41064771	USP9X	693_selected_genes
chrX	41069736	41069960	USP9X	693_selected_genes
chrX	41073795	41073987	USP9X	693_selected_genes
chrX	41075126	41075930	USP9X	693_selected_genes
chrX	41076447	41076621	USP9X	693_selected_genes
chrX	41077599	41077875	USP9X	693_selected_genes
chrX	41078329	41078509	USP9X	693_selected_genes
chrX	41082444	41082680	USP9X	693_selected_genes
chrX	41083969	41084240	USP9X	693_selected_genes
chrX	41084276	41084415	USP9X	693_selected_genes
chrX	41088480	41088687	USP9X	693_selected_genes
chrX	41088794	41089105	USP9X	693_selected_genes
chrX	41089728	41089874	USP9X	693_selected_genes
chrX	41091614	41091802	USP9X	693_selected_genes
chr3	10183506	10183896	VHL	693_selected_genes
chr3	10188172	10188345	VHL	693_selected_genes
chr3	10191445	10191674	VHL	693_selected_genes
chr1	117690254	117690429	VTCN1	693_selected_genes
chr1	117695687	117696016	VTCN1	693_selected_genes
chr1	117699170	117699568	VTCN1	693_selected_genes
chr1	117712703	117712818	VTCN1	693_selected_genes
chr1	117715783	117715874	VTCN1	693_selected_genes
chr1	117753420	117753502	VTCN1	693_selected_genes
chr13	27216382	27216565	WASF3	693_selected_genes
chr13	27239139	27239324	WASF3	693_selected_genes
chr13	27241628	27241832	WASF3	693_selected_genes
chr13	27245983	27246151	WASF3	693_selected_genes
chr13	27250660	27250886	WASF3	693_selected_genes
chr13	27254146	27254363	WASF3	693_selected_genes
chr13	27255165	27255482	WASF3	693_selected_genes
chr13	27256718	27257136	WASF3	693_selected_genes
chr13	27259799	27260007	WASF3	693_selected_genes
chr4	1902356	1903003	WHSC1	693_selected_genes
chr4	1905917	1906130	WHSC1	693_selected_genes
chr4	1913792	1913904	WHSC1	693_selected_genes
chr4	1918572	1918789	WHSC1	693_selected_genes

chr4	1919842	1920375	WHSC1	693_selected_genes
chr4	1932327	1932522	WHSC1	693_selected_genes
chr4	1936845	1937014	WHSC1	693_selected_genes
chr4	1940152	1940284	WHSC1	693_selected_genes
chr4	1941355	1941539	WHSC1	693_selected_genes
chr4	1944040	1944153	WHSC1	693_selected_genes
chr4	1952773	1952955	WHSC1	693_selected_genes
chr4	1953809	1953983	WHSC1	693_selected_genes
chr4	1954844	1955276	WHSC1	693_selected_genes
chr4	1956862	1957092	WHSC1	693_selected_genes
chr4	1957394	1957601	WHSC1	693_selected_genes
chr4	1957684	1957940	WHSC1	693_selected_genes
chr4	1959634	1959788	WHSC1	693_selected_genes
chr4	1961172	1961492	WHSC1	693_selected_genes
chr4	1962736	1962903	WHSC1	693_selected_genes
chr4	1976564	1976756	WHSC1	693_selected_genes
chr4	1976995	1977152	WHSC1	693_selected_genes
chr4	1978176	1978431	WHSC1	693_selected_genes
chr4	1980339	1980661	WHSC1	693_selected_genes
chr8	38133133	38133425	WHSC1L1	693_selected_genes
chr8	38133788	38134043	WHSC1L1	693_selected_genes
chr8	38135798	38135955	WHSC1L1	693_selected_genes
chr8	38137032	38137224	WHSC1L1	693_selected_genes
chr8	38138959	38139126	WHSC1L1	693_selected_genes
chr8	38145979	38146299	WHSC1L1	693_selected_genes
chr8	38146885	38147048	WHSC1L1	693_selected_genes
chr8	38147967	38148220	WHSC1L1	693_selected_genes
chr8	38153288	38153495	WHSC1L1	693_selected_genes
chr8	38156936	38157133	WHSC1L1	693_selected_genes
chr8	38162079	38162300	WHSC1L1	693_selected_genes
chr8	38162740	38162988	WHSC1L1	693_selected_genes
chr8	38172139	38172316	WHSC1L1	693_selected_genes
chr8	38172908	38173087	WHSC1L1	693_selected_genes
chr8	38173404	38173585	WHSC1L1	693_selected_genes
chr8	38175448	38175581	WHSC1L1	693_selected_genes
chr8	38176387	38176483	WHSC1L1	693_selected_genes
chr8	38178537	38178715	WHSC1L1	693_selected_genes
chr8	38184222	38184399	WHSC1L1	693_selected_genes
chr8	38186870	38187436	WHSC1L1	693_selected_genes
chr8	38188923	38189128	WHSC1L1	693_selected_genes
chr8	38194797	38195010	WHSC1L1	693_selected_genes
chr8	38196028	38196150	WHSC1L1	693_selected_genes
chr8	38204989	38205714	WHSC1L1	693_selected_genes
chr6	112375481	112375633	WISP3	693_selected_genes
chr6	112381190	112381303	WISP3	693_selected_genes
chr6	112382168	112382522	WISP3	693_selected_genes
chr6	112385932	112386225	WISP3	693_selected_genes
chr6	112388146	112388260	WISP3	693_selected_genes
chr6	112389382	112389626	WISP3	693_selected_genes
chr6	112390516	112390848	WISP3	693_selected_genes
chr12	862706	863515	WNK1	693_selected_genes
chr12	922782	923005	WNK1	693_selected_genes
chr12	936182	936482	WNK1	693_selected_genes
chr12	939143	939351	WNK1	693_selected_genes
chr12	960833	960973	WNK1	693_selected_genes
chr12	966301	966440	WNK1	693_selected_genes
chr12	968385	968655	WNK1	693_selected_genes
chr12	970153	970534	WNK1	693_selected_genes
chr12	971221	971461	WNK1	693_selected_genes
chr12	974250	974555	WNK1	693_selected_genes
chr12	976970	978325	WNK1	693_selected_genes
chr12	980405	980539	WNK1	693_selected_genes
chr12	987352	987552	WNK1	693_selected_genes
chr12	988713	989222	WNK1	693_selected_genes
chr12	989861	990190	WNK1	693_selected_genes
chr12	990832	990980	WNK1	693_selected_genes
chr12	991051	991264	WNK1	693_selected_genes
chr12	992083	992250	WNK1	693_selected_genes
chr12	992535	992759	WNK1	693_selected_genes
chr12	992914	993022	WNK1	693_selected_genes
chr12	993261	993434	WNK1	693_selected_genes
chr12	993789	995275	WNK1	693_selected_genes
chr12	996361	996495	WNK1	693_selected_genes
chr12	998280	998414	WNK1	693_selected_genes
chr12	999593	999704	WNK1	693_selected_genes
chr12	1003702	1003826	WNK1	693_selected_genes
chr12	1005211	1005923	WNK1	693_selected_genes
chr12	1006619	1006872	WNK1	693_selected_genes
chr12	1009616	1009861	WNK1	693_selected_genes
chr12	1010701	1010844	WNK1	693_selected_genes
chr12	1013593	1013685	WNK1	693_selected_genes
chr12	1016987	1017225	WNK1	693_selected_genes
chr12	1017615	1017983	WNK1	693_selected_genes
chr8	30915938	30916084	WRN	693_selected_genes

chr8	30916643	30916806	WRN	693_selected_genes
chr8	30921779	30921975	WRN	693_selected_genes
chr8	30922405	30922604	WRN	693_selected_genes
chr8	30924523	30924723	WRN	693_selected_genes
chr8	30925748	30925868	WRN	693_selected_genes
chr8	30933663	30933828	WRN	693_selected_genes
chr8	30938357	30938837	WRN	693_selected_genes
chr8	30941189	30941320	WRN	693_selected_genes
chr8	30942656	30942787	WRN	693_selected_genes
chr8	30945266	30945461	WRN	693_selected_genes
chr8	30946380	30946506	WRN	693_selected_genes
chr8	30947955	30948073	WRN	693_selected_genes
chr8	30948324	30948483	WRN	693_selected_genes
chr8	30949320	30949439	WRN	693_selected_genes
chr8	30954258	30954391	WRN	693_selected_genes
chr8	30958339	30958496	WRN	693_selected_genes
chr8	30969105	30969340	WRN	693_selected_genes
chr8	30973844	30974069	WRN	693_selected_genes
chr8	30977733	30977965	WRN	693_selected_genes
chr8	30982012	30982164	WRN	693_selected_genes
chr8	30982398	30982541	WRN	693_selected_genes
chr8	30989855	30990047	WRN	693_selected_genes
chr8	30998920	30999141	WRN	693_selected_genes
chr8	30999171	30999316	WRN	693_selected_genes
chr8	31000116	31000242	WRN	693_selected_genes
chr8	31001040	31001164	WRN	693_selected_genes
chr8	31004543	31004669	WRN	693_selected_genes
chr8	31004854	31005017	WRN	693_selected_genes
chr8	31007831	31007996	WRN	693_selected_genes
chr8	31012114	31012296	WRN	693_selected_genes
chr8	31014858	31015071	WRN	693_selected_genes
chr8	31024512	31024771	WRN	693_selected_genes
chr8	31030485	31030643	WRN	693_selected_genes
chr11	32410578	32410750	WT1	693_selected_genes
chr11	32413492	32413635	WT1	693_selected_genes
chr11	32414186	32414326	WT1	693_selected_genes
chr11	32417777	32417978	WT1	693_selected_genes
chr11	32421468	32421615	WT1	693_selected_genes
chr11	32438010	32438111	WT1	693_selected_genes
chr11	32439093	32439225	WT1	693_selected_genes
chr11	32449476	32449629	WT1	693_selected_genes
chr11	32450017	32450190	WT1	693_selected_genes
chr11	32452050	32452110	WT1	693_selected_genes
chr11	32456220	32456916	WT1	693_selected_genes
chrX	123019487	123020414	XIAP	693_selected_genes
chrX	123022443	123022593	XIAP	693_selected_genes
chrX	123025062	123025191	XIAP	693_selected_genes
chrX	123026555	123026648	XIAP	693_selected_genes
chrX	123034317	123034568	XIAP	693_selected_genes
chrX	123040812	123041056	XIAP	693_selected_genes
chr9	100437695	100437894	XPA	693_selected_genes
chr9	100444565	100444737	XPA	693_selected_genes
chr9	100447179	100447347	XPA	693_selected_genes
chr9	100449352	100449568	XPA	693_selected_genes
chr9	100451790	100451946	XPA	693_selected_genes
chr9	100455905	100456066	XPA	693_selected_genes
chr9	100459377	100459599	XPA	693_selected_genes
chr3	14187415	14187684	XPC	693_selected_genes
chr3	14188764	14188904	XPC	693_selected_genes
chr3	14189382	14189526	XPC	693_selected_genes
chr3	14190036	14190256	XPC	693_selected_genes
chr3	14190288	14190473	XPC	693_selected_genes
chr3	14193809	14193941	XPC	693_selected_genes
chr3	14197809	14198020	XPC	693_selected_genes
chr3	14199485	14200417	XPC	693_selected_genes
chr3	14201215	14201355	XPC	693_selected_genes
chr3	14206287	14206458	XPC	693_selected_genes
chr3	14206902	14207110	XPC	693_selected_genes
chr3	14208643	14208778	XPC	693_selected_genes
chr3	14209731	14209905	XPC	693_selected_genes
chr3	14211912	14212075	XPC	693_selected_genes
chr3	14214341	14214587	XPC	693_selected_genes
chr3	14219940	14220093	XPC	693_selected_genes
chr2	61705929	61706126	XPO1	693_selected_genes
chr2	61708294	61708441	XPO1	693_selected_genes
chr2	61709489	61709699	XPO1	693_selected_genes
chr2	61710066	61710251	XPO1	693_selected_genes
chr2	61711046	61711265	XPO1	693_selected_genes
chr2	61712877	61713122	XPO1	693_selected_genes
chr2	61715274	61715431	XPO1	693_selected_genes
chr2	61715697	61715931	XPO1	693_selected_genes
chr2	61717751	61717936	XPO1	693_selected_genes
chr2	61719144	61719358	XPO1	693_selected_genes
chr2	61719434	61719641	XPO1	693_selected_genes

chr2	61719676	61719908	XPO1	693_selected_genes
chr2	61720024	61720213	XPO1	693_selected_genes
chr2	61721003	61721251	XPO1	693_selected_genes
chr2	61722564	61722773	XPO1	693_selected_genes
chr2	61723988	61724167	XPO1	693_selected_genes
chr2	61725782	61725952	XPO1	693_selected_genes
chr2	61725974	61726073	XPO1	693_selected_genes
chr2	61726822	61727054	XPO1	693_selected_genes
chr2	61729105	61729200	XPO1	693_selected_genes
chr2	61729358	61729470	XPO1	693_selected_genes
chr2	61749720	61749843	XPO1	693_selected_genes
chr2	61753508	61753681	XPO1	693_selected_genes
chr2	61760881	61761057	XPO1	693_selected_genes
chr7	152345701	152346473	XRCC2	693_selected_genes
chr7	152357760	152357892	XRCC2	693_selected_genes
chr7	152373100	152373189	XRCC2	693_selected_genes
chr14	104165109	104165379	XRCC3	693_selected_genes
chr14	104165444	104165541	XRCC3	693_selected_genes
chr14	104165675	104165938	XRCC3	693_selected_genes
chr14	104169484	104169689	XRCC3	693_selected_genes
chr14	104172640	104172737	XRCC3	693_selected_genes
chr14	104173314	104173577	XRCC3	693_selected_genes
chr14	104174833	104175021	XRCC3	693_selected_genes
chr14	104177344	104177449	XRCC3	693_selected_genes
chr11	101981554	101981925	YAP1	693_selected_genes
chr11	101984849	101985150	YAP1	693_selected_genes
chr11	102033161	102033327	YAP1	693_selected_genes
chr11	102056723	102056887	YAP1	693_selected_genes
chr11	102076598	102076842	YAP1	693_selected_genes
chr11	102080222	102080320	YAP1	693_selected_genes
chr11	102094327	102094508	YAP1	693_selected_genes
chr11	102098174	102098337	YAP1	693_selected_genes
chr11	102100407	102100696	YAP1	693_selected_genes
chr18	724398	724657	YES1	693_selected_genes
chr18	732808	732990	YES1	693_selected_genes
chr18	736782	736986	YES1	693_selected_genes
chr18	739709	739836	YES1	693_selected_genes
chr18	742892	743122	YES1	693_selected_genes
chr18	743234	743440	YES1	693_selected_genes
chr18	745682	745882	YES1	693_selected_genes
chr18	745922	746076	YES1	693_selected_genes
chr18	747894	748043	YES1	693_selected_genes
chr18	751679	751829	YES1	693_selected_genes
chr18	756531	756860	YES1	693_selected_genes
chr18	775164	775221	YES1	693_selected_genes
chr16	72821037	72822772	ZFHX3	693_selected_genes
chr16	72827128	72832638	ZFHX3	693_selected_genes
chr16	72833900	72834053	ZFHX3	693_selected_genes
chr16	72845450	72845701	ZFHX3	693_selected_genes
chr16	72845778	72845962	ZFHX3	693_selected_genes
chr16	72863652	72863783	ZFHX3	693_selected_genes
chr16	72923604	72923886	ZFHX3	693_selected_genes
chr16	72984342	72984889	ZFHX3	693_selected_genes
chr16	72991300	72994069	ZFHX3	693_selected_genes
chr14	69256224	69257234	ZFP36L1	693_selected_genes
chr14	69257674	69257929	ZFP36L1	693_selected_genes
chr14	69259573	69259812	ZFP36L1	693_selected_genes
chr14	69262485	69263036	ZFP36L1	693_selected_genes
chr2	43451432	43452916	ZFP36L2	693_selected_genes
chr2	43453378	43453479	ZFP36L2	693_selected_genes
chr13	20567187	20568084	ZMYM2	693_selected_genes
chr13	20576964	20577300	ZMYM2	693_selected_genes
chr13	20579188	20579404	ZMYM2	693_selected_genes
chr13	20580488	20580751	ZMYM2	693_selected_genes
chr13	20593661	20593849	ZMYM2	693_selected_genes
chr13	20600726	20600927	ZMYM2	693_selected_genes
chr13	20601317	20601483	ZMYM2	693_selected_genes
chr13	20605433	20605607	ZMYM2	693_selected_genes
chr13	20608368	20608569	ZMYM2	693_selected_genes
chr13	20610851	20611076	ZMYM2	693_selected_genes
chr13	20625547	20625763	ZMYM2	693_selected_genes
chr13	20626391	20626476	ZMYM2	693_selected_genes
chr13	20632689	20632869	ZMYM2	693_selected_genes
chr13	20632963	20633078	ZMYM2	693_selected_genes
chr13	20633561	20633727	ZMYM2	693_selected_genes
chr13	20635167	20635389	ZMYM2	693_selected_genes
chr13	20636960	20637136	ZMYM2	693_selected_genes
chr13	20638565	20638710	ZMYM2	693_selected_genes
chr13	20640965	20641184	ZMYM2	693_selected_genes
chr13	20641353	20641555	ZMYM2	693_selected_genes
chr13	20656130	20656295	ZMYM2	693_selected_genes
chr13	20656895	20657197	ZMYM2	693_selected_genes
chr13	20657770	20657941	ZMYM2	693_selected_genes
chr13	20659936	20660179	ZMYM2	693_selected_genes

chrX	70460740	70460983	ZMYM3	693_selected_genes
chrX	70461051	70461219	ZMYM3	693_selected_genes
chrX	70461994	70462299	ZMYM3	693_selected_genes
chrX	70462794	70462959	ZMYM3	693_selected_genes
chrX	70463653	70463855	ZMYM3	693_selected_genes
chrX	70464126	70464345	ZMYM3	693_selected_genes
chrX	70464614	70464768	ZMYM3	693_selected_genes
chrX	70465163	70465360	ZMYM3	693_selected_genes
chrX	70465492	70465717	ZMYM3	693_selected_genes
chrX	70465810	70465973	ZMYM3	693_selected_genes
chrX	70466177	70466387	ZMYM3	693_selected_genes
chrX	70466419	70466567	ZMYM3	693_selected_genes
chrX	70467169	70467385	ZMYM3	693_selected_genes
chrX	70467558	70467781	ZMYM3	693_selected_genes
chrX	70467986	70468187	ZMYM3	693_selected_genes
chrX	70468262	70468399	ZMYM3	693_selected_genes
chrX	70468510	70468676	ZMYM3	693_selected_genes
chrX	70468843	70469044	ZMYM3	693_selected_genes
chrX	70469267	70469554	ZMYM3	693_selected_genes
chrX	70469850	70470078	ZMYM3	693_selected_genes
chrX	70470256	70470601	ZMYM3	693_selected_genes
chrX	70471002	70471125	ZMYM3	693_selected_genes
chrX	70471382	70471476	ZMYM3	693_selected_genes
chrX	70472413	70473130	ZMYM3	693_selected_genes
chr20	52185629	52185828	ZNF217	693_selected_genes
chr20	52188257	52188417	ZNF217	693_selected_genes
chr20	52192240	52193844	ZNF217	693_selected_genes
chr20	52194847	52195014	ZNF217	693_selected_genes
chr20	52197974	52199390	ZNF217	693_selected_genes
chr8	37553472	37553765	ZNF703	693_selected_genes
chr8	37554637	37556217	ZNF703	693_selected_genes
chr17	80787992	80788778	ZNF750	693_selected_genes
chr17	80788869	80790355	ZNF750	693_selected_genes
chr19	58361812	58361954	ZNF814	693_selected_genes
chr19	58363687	58363781	ZNF814	693_selected_genes
chr19	58370242	58370376	ZNF814	693_selected_genes
chr19	58376212	58376312	ZNF814	693_selected_genes
chr19	58379809	58379972	ZNF814	693_selected_genes
chr19	58384164	58386619	ZNF814	693_selected_genes
chr19	58388232	58388435	ZNF814	693_selected_genes
chr19	58397225	58397362	ZNF814	693_selected_genes
chr19	58400109	58400195	ZNF814	693_selected_genes
chrX	15808593	15808684	ZRSR2	693_selected_genes
chrX	15809031	15809161	ZRSR2	693_selected_genes
chrX	15817969	15818101	ZRSR2	693_selected_genes
chrX	15819371	15819662	ZRSR2	693_selected_genes
chrX	15821785	15821944	ZRSR2	693_selected_genes
chrX	15822208	15822345	ZRSR2	693_selected_genes
chrX	15826330	15826419	ZRSR2	693_selected_genes
chrX	15827297	15827466	ZRSR2	693_selected_genes
chrX	15833774	15834038	ZRSR2	693_selected_genes
chrX	15836684	15836790	ZRSR2	693_selected_genes
chrX	15838304	15838464	ZRSR2	693_selected_genes
chrX	15840828	15841390	ZRSR2	693_selected_genes
chr16	77281845	77281987	ADAMTS18	693_selected_genes
chr16	77317827	77317993	ADAMTS18	693_selected_genes
chr16	77323135	77323333	ADAMTS18	693_selected_genes
chr16	77325137	77325400	ADAMTS18	693_selected_genes
chr16	77326947	77327180	ADAMTS18	693_selected_genes
chr16	77328794	77329049	ADAMTS18	693_selected_genes
chr16	77331160	77331337	ADAMTS18	693_selected_genes
chr16	77334134	77334326	ADAMTS18	693_selected_genes
chr16	77353720	77354015	ADAMTS18	693_selected_genes
chr16	77354950	77355124	ADAMTS18	693_selected_genes
chr16	77356207	77356388	ADAMTS18	693_selected_genes
chr16	77359737	77359960	ADAMTS18	693_selected_genes
chr16	77369627	77369826	ADAMTS18	693_selected_genes
chr16	77375575	77375721	ADAMTS18	693_selected_genes
chr16	77387604	77387808	ADAMTS18	693_selected_genes
chr16	77389811	77389999	ADAMTS18	693_selected_genes
chr16	77393189	77393345	ADAMTS18	693_selected_genes
chr16	77395976	77396186	ADAMTS18	693_selected_genes
chr16	77397673	77397807	ADAMTS18	693_selected_genes
chr16	77398059	77398303	ADAMTS18	693_selected_genes
chr16	77401308	77401645	ADAMTS18	693_selected_genes
chr16	77465166	77465533	ADAMTS18	693_selected_genes
chr16	77468289	77468427	ADAMTS18	693_selected_genes
chr16	77468477	77468617	ADAMTS18	693_selected_genes
chr19	13049468	13049609	CALR	693_selected_genes
chr19	13049922	13050074	CALR	693_selected_genes
chr19	13050216	13050470	CALR	693_selected_genes
chr19	13050841	13050986	CALR	693_selected_genes
chr19	13051031	13051291	CALR	693_selected_genes
chr19	13051329	13051493	CALR	693_selected_genes

chr19	13051532	13051726	CALR	693_selected_genes
chr19	13054325	13054468	CALR	693_selected_genes
chr19	13054501	13054820	CALR	693_selected_genes
chr5	98192058	98192454	CHD1	693_selected_genes
chr5	98193858	98194119	CHD1	693_selected_genes
chr5	98194642	98194744	CHD1	693_selected_genes
chr5	98195650	98195797	CHD1	693_selected_genes
chr5	98199086	98199315	CHD1	693_selected_genes
chr5	98204173	98204364	CHD1	693_selected_genes
chr5	98205432	98205611	CHD1	693_selected_genes
chr5	98206365	98206532	CHD1	693_selected_genes
chr5	98207729	98207930	CHD1	693_selected_genes
chr5	98208095	98208284	CHD1	693_selected_genes
chr5	98209271	98209452	CHD1	693_selected_genes
chr5	98210750	98210842	CHD1	693_selected_genes
chr5	98212076	98212287	CHD1	693_selected_genes
chr5	98215230	98215457	CHD1	693_selected_genes
chr5	98216754	98216900	CHD1	693_selected_genes
chr5	98216957	98217104	CHD1	693_selected_genes
chr5	98217653	98217852	CHD1	693_selected_genes
chr5	98218766	98218966	CHD1	693_selected_genes
chr5	98221256	98221378	CHD1	693_selected_genes
chr5	98223766	98223969	CHD1	693_selected_genes
chr5	98224754	98224967	CHD1	693_selected_genes
chr5	98228203	98228442	CHD1	693_selected_genes
chr5	98229094	98229335	CHD1	693_selected_genes
chr5	98230275	98230415	CHD1	693_selected_genes
chr5	98231904	98232171	CHD1	693_selected_genes
chr5	98232871	98233049	CHD1	693_selected_genes
chr5	98233934	98234163	CHD1	693_selected_genes
chr5	98234342	98234493	CHD1	693_selected_genes
chr5	98235158	98235434	CHD1	693_selected_genes
chr5	98236489	98236811	CHD1	693_selected_genes
chr5	98236864	98237064	CHD1	693_selected_genes
chr5	98238578	98238693	CHD1	693_selected_genes
chr5	98239470	98239637	CHD1	693_selected_genes
chr5	98240575	98240827	CHD1	693_selected_genes
chr5	98262012	98262115	CHD1	693_selected_genes
chr11	128564128	128564196	FLI1	693_selected_genes
chr11	128627984	128628246	FLI1	693_selected_genes
chr11	128634520	128634589	FLI1	693_selected_genes
chr11	128634659	128634741	FLI1	693_selected_genes
chr11	128637987	128638192	FLI1	693_selected_genes
chr11	128642651	128642905	FLI1	693_selected_genes
chr11	128651827	128651943	FLI1	693_selected_genes
chr11	128675235	128675351	FLI1	693_selected_genes
chr11	128677049	128677159	FLI1	693_selected_genes
chr11	128679026	128679124	FLI1	693_selected_genes
chr11	128680328	128680908	FLI1	693_selected_genes
chr2	176994466	176994527	HOXD8	693_selected_genes
chr2	176995069	176995696	HOXD8	693_selected_genes
chr2	176996019	176996365	HOXD8	693_selected_genes
chrX	107975775	107979599	IRS4	693_selected_genes
chr1	11734806	11734898	MAD2L2	693_selected_genes
chr1	11735113	11735256	MAD2L2	693_selected_genes
chr1	11735687	11735813	MAD2L2	693_selected_genes
chr1	11735901	11736222	MAD2L2	693_selected_genes
chr1	11736840	11737030	MAD2L2	693_selected_genes
chr1	11737487	11737696	MAD2L2	693_selected_genes
chr1	11740384	11740683	MAD2L2	693_selected_genes
chr8	23538708	23539177	NKX3-1	693_selected_genes
chr8	23540091	23540427	NKX3-1	693_selected_genes
chr8	68864604	68864795	PREX2	693_selected_genes
chr8	68930055	68930177	PREX2	693_selected_genes
chr8	68931758	68931931	PREX2	693_selected_genes
chr8	68934245	68934400	PREX2	693_selected_genes
chr8	68939431	68939583	PREX2	693_selected_genes
chr8	68942706	68942918	PREX2	693_selected_genes
chr8	68950368	68950552	PREX2	693_selected_genes
chr8	68956696	68956850	PREX2	693_selected_genes
chr8	68965306	68965506	PREX2	693_selected_genes
chr8	68968039	68968234	PREX2	693_selected_genes
chr8	68972888	68973039	PREX2	693_selected_genes
chr8	68981242	68981396	PREX2	693_selected_genes
chr8	68982044	68982144	PREX2	693_selected_genes
chr8	68984704	68984830	PREX2	693_selected_genes
chr8	68989606	68989729	PREX2	693_selected_genes
chr8	68992652	68992845	PREX2	693_selected_genes
chr8	68992955	68993098	PREX2	693_selected_genes
chr8	68995449	68995648	PREX2	693_selected_genes
chr8	68999933	69000069	PREX2	693_selected_genes
chr8	69002788	69002975	PREX2	693_selected_genes
chr8	69005814	69005982	PREX2	693_selected_genes
chr8	69009226	69009461	PREX2	693_selected_genes

chr8	69011891	69012103	PREX2	693_selected_genes
chr8	69017347	69017622	PREX2	693_selected_genes
chr8	69020318	69020591	PREX2	693_selected_genes
chr8	69021625	69021883	PREX2	693_selected_genes
chr8	69023141	69023195	PREX2	693_selected_genes
chr8	69027962	69028192	PREX2	693_selected_genes
chr8	69030759	69030904	PREX2	693_selected_genes
chr8	69031641	69031774	PREX2	693_selected_genes
chr8	69032405	69032546	PREX2	693_selected_genes
chr8	69033130	69033309	PREX2	693_selected_genes
chr8	69039587	69039679	PREX2	693_selected_genes
chr8	69046268	69046536	PREX2	693_selected_genes
chr8	69050624	69050777	PREX2	693_selected_genes
chr8	69058418	69058612	PREX2	693_selected_genes
chr8	69069531	69069696	PREX2	693_selected_genes
chr8	69103931	69104048	PREX2	693_selected_genes
chr8	69104544	69104785	PREX2	693_selected_genes
chr8	69129825	69129978	PREX2	693_selected_genes
chr8	69132385	69132546	PREX2	693_selected_genes
chr8	69136768	69136886	PREX2	693_selected_genes
chr8	69143542	69143638	PREX2	693_selected_genes
chr16	3632317	3632719	SLX4	693_selected_genes
chr16	3633072	3633536	SLX4	693_selected_genes
chr16	3634744	3634897	SLX4	693_selected_genes
chr16	3638977	3641336	SLX4	693_selected_genes
chr16	3642674	3642891	SLX4	693_selected_genes
chr16	3644428	3644625	SLX4	693_selected_genes
chr16	3645580	3645719	SLX4	693_selected_genes
chr16	3646128	3646419	SLX4	693_selected_genes
chr16	3647354	3647721	SLX4	693_selected_genes
chr16	3647772	3648025	SLX4	693_selected_genes
chr16	3650954	3651217	SLX4	693_selected_genes
chr16	3652093	3652333	SLX4	693_selected_genes
chr16	3656449	3656724	SLX4	693_selected_genes
chr16	3658405	3658990	SLX4	693_selected_genes
chr2	139307761	139307889	SPOPL	693_selected_genes
chr2	139308027	139308199	SPOPL	693_selected_genes
chr2	139308447	139308649	SPOPL	693_selected_genes
chr2	139310098	139310276	SPOPL	693_selected_genes
chr2	139311983	139312126	SPOPL	693_selected_genes
chr2	139316566	139316794	SPOPL	693_selected_genes
chr2	139316826	139316932	SPOPL	693_selected_genes
chr2	139318349	139318522	SPOPL	693_selected_genes
chr2	139322252	139322445	SPOPL	693_selected_genes
chr2	139322484	139322588	SPOPL	693_selected_genes
chr2	139326480	139326675	SPOPL	693_selected_genes
chr6	31939748	31940018	STK19	693_selected_genes
chr6	31940053	31940313	STK19	693_selected_genes
chr6	31940372	31940559	STK19	693_selected_genes
chr6	31946654	31946812	STK19	693_selected_genes
chr6	31947165	31947355	STK19	693_selected_genes
chr6	31948202	31948350	STK19	693_selected_genes
chr6	31948405	31948603	STK19	693_selected_genes
chr6	31948755	31948851	STK19	693_selected_genes
chr6	31981492	31981589	STK19	693_selected_genes
chr7	23749879	23749979	STK31	693_selected_genes
chr7	23751692	23751789	STK31	693_selected_genes
chr7	23751827	23751930	STK31	693_selected_genes
chr7	23757074	23757223	STK31	693_selected_genes
chr7	23766834	23766959	STK31	693_selected_genes
chr7	23768682	23768893	STK31	693_selected_genes
chr7	23775131	23775540	STK31	693_selected_genes
chr7	23776497	23776722	STK31	693_selected_genes
chr7	23792310	23792476	STK31	693_selected_genes
chr7	23793908	23794118	STK31	693_selected_genes
chr7	23802394	23802567	STK31	693_selected_genes
chr7	23808588	23808818	STK31	693_selected_genes
chr7	23809233	23809400	STK31	693_selected_genes
chr7	23810598	23810768	STK31	693_selected_genes
chr7	23811740	23811922	STK31	693_selected_genes
chr7	23821012	23821164	STK31	693_selected_genes
chr7	23823176	23823307	STK31	693_selected_genes
chr7	23825071	23825247	STK31	693_selected_genes
chr7	23826101	23826277	STK31	693_selected_genes
chr7	23826431	23826568	STK31	693_selected_genes
chr7	23827573	23827773	STK31	693_selected_genes
chr7	23830417	23830590	STK31	693_selected_genes
chr7	23854737	23854856	STK31	693_selected_genes
chr7	23871729	23872010	STK31	693_selected_genes
chr19	59055477	59055603	TRIM28	693_selected_genes
chr19	59056099	59056489	TRIM28	693_selected_genes
chr19	59056766	59056929	TRIM28	693_selected_genes
chr19	59057105	59057288	TRIM28	693_selected_genes
chr19	59058717	59058903	TRIM28	693_selected_genes

chr19	59058938	59059105	TRIM28	693_selected_genes
chr19	59059133	59059298	TRIM28	693_selected_genes
chr19	59059375	59059572	TRIM28	693_selected_genes
chr19	59059635	59059800	TRIM28	693_selected_genes
chr19	59059827	59059984	TRIM28	693_selected_genes
chr19	59060081	59060168	TRIM28	693_selected_genes
chr19	59060203	59060302	TRIM28	693_selected_genes
chr19	59060329	59060632	TRIM28	693_selected_genes
chr19	59060672	59061042	TRIM28	693_selected_genes
chr19	59061078	59061252	TRIM28	693_selected_genes
chr19	59061290	59061427	TRIM28	693_selected_genes
chr19	59061488	59061676	TRIM28	693_selected_genes
chr19	59061718	59061945	TRIM28	693_selected_genes
chr1	202300937	202301113	UBE2T	693_selected_genes
chr1	202302112	202302246	UBE2T	693_selected_genes
chr1	202302339	202302488	UBE2T	693_selected_genes
chr1	202302552	202302708	UBE2T	693_selected_genes
chr1	202304091	202304211	UBE2T	693_selected_genes
chr1	202304744	202304907	UBE2T	693_selected_genes
chrY	6734088	6734144	AMELY	CNV_SNPs
chrY	6736052	6736528	AMELY	CNV_SNPs
chrY	6736747	6736842	AMELY	CNV_SNPs
chrY	6736883	6736975	AMELY	CNV_SNPs
chrY	6738021	6738119	AMELY	CNV_SNPs
chrY	6740570	6740674	AMELY	CNV_SNPs
chrY	25276401	25276528	DAZ1	CNV_SNPs
chrY	25281331	25281445	DAZ1	CNV_SNPs
chrY	25285968	25286053	DAZ1	CNV_SNPs
chrY	25286747	25286869	DAZ1	CNV_SNPs
chrY	25289132	25289254	DAZ1	CNV_SNPs
chrY	25298918	25299040	DAZ1	CNV_SNPs
chrY	25301295	25301417	DAZ1	CNV_SNPs
chrY	25303691	25303813	DAZ1	CNV_SNPs
chrY	25306074	25306196	DAZ1	CNV_SNPs
chrY	25308470	25308592	DAZ1	CNV_SNPs
chrY	25310861	25310983	DAZ1	CNV_SNPs
chrY	25313306	25313428	DAZ1	CNV_SNPs
chrY	25314778	25314968	DAZ1	CNV_SNPs
chrY	25315007	25315121	DAZ1	CNV_SNPs
chrY	25315507	25315609	DAZ1	CNV_SNPs
chrY	25316119	25316261	DAZ1	CNV_SNPs
chrY	25316485	25316682	DAZ1	CNV_SNPs
chrY	25325618	25325808	DAZ1	CNV_SNPs
chrY	25325847	25325961	DAZ1	CNV_SNPs
chrY	25326347	25326449	DAZ1	CNV_SNPs
chrY	25326959	25327101	DAZ1	CNV_SNPs
chrY	25327325	25327522	DAZ1	CNV_SNPs
chrY	25336466	25336656	DAZ1	CNV_SNPs
chrY	25336695	25336809	DAZ1	CNV_SNPs
chrY	25337195	25337297	DAZ1	CNV_SNPs
chrY	25337807	25337949	DAZ1	CNV_SNPs
chrY	25338173	25338370	DAZ1	CNV_SNPs
chrY	25344919	25344972	DAZ1	CNV_SNPs
chrY	21867855	21868256	KDM5D	CNV_SNPs
chrY	21868301	21868551	KDM5D	CNV_SNPs
chrY	21868654	21868774	KDM5D	CNV_SNPs
chrY	21869007	21869657	KDM5D	CNV_SNPs
chrY	21869794	21869982	KDM5D	CNV_SNPs
chrY	21870104	21870334	KDM5D	CNV_SNPs
chrY	21870735	21870924	KDM5D	CNV_SNPs
chrY	21871311	21871720	KDM5D	CNV_SNPs
chrY	21872236	21872383	KDM5D	CNV_SNPs
chrY	21877212	21877410	KDM5D	CNV_SNPs
chrY	21877475	21877650	KDM5D	CNV_SNPs
chrY	21877683	21877915	KDM5D	CNV_SNPs
chrY	21878139	21878384	KDM5D	CNV_SNPs
chrY	21878456	21878626	KDM5D	CNV_SNPs
chrY	21882732	21882945	KDM5D	CNV_SNPs
chrY	21882990	21883222	KDM5D	CNV_SNPs
chrY	21885201	21885344	KDM5D	CNV_SNPs
chrY	21893632	21893841	KDM5D	CNV_SNPs
chrY	21893906	21894076	KDM5D	CNV_SNPs
chrY	21894444	21894653	KDM5D	CNV_SNPs
chrY	21897212	21897408	KDM5D	CNV_SNPs
chrY	21897481	21897661	KDM5D	CNV_SNPs
chrY	21901388	21901573	KDM5D	CNV_SNPs
chrY	21903178	21903399	KDM5D	CNV_SNPs
chrY	21903595	21903768	KDM5D	CNV_SNPs
chrY	21905022	21905150	KDM5D	CNV_SNPs
chrY	21906245	21906445	KDM5D	CNV_SNPs
chrY	7142328	7142544	PRKY	CNV_SNPs
chrY	7171952	7172171	PRKY	CNV_SNPs
chrY	7193921	7194235	PRKY	CNV_SNPs
chrY	7209130	7209300	PRKY	CNV_SNPs

chrY	7224150	7224296	PRKY	CNV_SNPs
chrY	7235371	7235440	PRKY	CNV_SNPs
chrY	22918024	22918077	RPS4Y2	CNV_SNPs
chrY	22918638	22918766	RPS4Y2	CNV_SNPs
chrY	22921728	22921959	RPS4Y2	CNV_SNPs
chrY	22923144	22923292	RPS4Y2	CNV_SNPs
chrY	22930665	22930887	RPS4Y2	CNV_SNPs
chrY	22941369	22941577	RPS4Y2	CNV_SNPs
chrY	22942791	22942943	RPS4Y2	CNV_SNPs
chr17	45356535	45356586	rs1000232	CNV_SNPs
chr4	120259187	120259238	rs10004114	CNV_SNPs
chr4	62707627	62707678	rs10021694	CNV_SNPs
chr15	38456835	38456886	rs1003179	CNV_SNPs
chr5	112675998	112676049	rs10067046	CNV_SNPs
chr5	154504876	154504927	rs10078699	CNV_SNPs
chr17	64938316	64938367	rs1008365	CNV_SNPs
chr8	11268051	11268102	rs10094320	CNV_SNPs
chr8	98381999	98382050	rs10097828	CNV_SNPs
chr8	18464904	18464955	rs10102929	CNV_SNPs
chr8	38858405	38858456	rs10106085	CNV_SNPs
chr9	84386686	84386737	rs1010745	CNV_SNPs
chr9	32890562	32890613	rs10123304	CNV_SNPs
chr14	50551008	50551059	rs10140137	CNV_SNPs
chr14	81376464	81376515	rs10142753	CNV_SNPs
chr14	22395224	22395275	rs10150202	CNV_SNPs
chr4	165697014	165697065	rs10155303	CNV_SNPs
chr10	15009342	15009393	rs10159658	CNV_SNPs
chr15	26216344	26216395	rs10162861	CNV_SNPs
chr2	4901077	4901128	rs10174397	CNV_SNPs
chr2	46128432	46128483	rs10187908	CNV_SNPs
chrX	132127819	132127870	rs1018794	CNV_SNPs
chr2	152156232	152156283	rs10203366	CNV_SNPs
chr7	82956863	82956914	rs10226944	CNV_SNPs
chr7	82948568	82948619	rs10230672	CNV_SNPs
chr7	85608081	85608132	rs10231971	CNV_SNPs
chr7	103122619	103122670	rs10233848	CNV_SNPs
chr7	22987474	22987525	rs10253228	CNV_SNPs
chr9	87315091	87315142	rs1025743	CNV_SNPs
chr18	31418105	31418156	rs1026257	CNV_SNPs
chr14	98829979	98830030	rs1026504	CNV_SNPs
chrX	15172163	15172214	rs1031914	CNV_SNPs
chr15	99861592	99861643	rs1033035	CNV_SNPs
chr7	97793183	97793234	rs1039057	CNV_SNPs
chr6	134758287	134758338	rs1040636	CNV_SNPs
chr19	36833772	36833823	rs10418925	CNV_SNPs
chr3	156952168	156952219	rs10451916	CNV_SNPs
chr14	75322301	75322352	rs10483863	CNV_SNPs
chr7	52528962	52529013	rs10488329	CNV_SNPs
chr1	157550040	157550091	rs10489673	CNV_SNPs
chr8	4166400	4166451	rs10503249	CNV_SNPs
chr8	18100729	18100780	rs10503610	CNV_SNPs
chr9	107040687	107040738	rs10512324	CNV_SNPs
chr9	119905343	119905394	rs10513286	CNV_SNPs
chr4	105135417	105135468	rs10516507	CNV_SNPs
chr15	88126103	88126154	rs10520660	CNV_SNPs
chr10	3097186	3097237	rs10732921	CNV_SNPs
chr11	35242881	35242932	rs10734436	CNV_SNPs
chr10	68050495	68050546	rs10740230	CNV_SNPs
chr11	18586096	18586147	rs10741747	CNV_SNPs
chr12	53130318	53130369	rs10747645	CNV_SNPs
chr10	122302216	122302267	rs10749377	CNV_SNPs
chr11	110097785	110097836	rs10749957	CNV_SNPs
chr1	175869528	175869579	rs10753110	CNV_SNPs
chr2	204836572	204836623	rs1075547	CNV_SNPs
chr12	18398210	18398261	rs10770329	CNV_SNPs
chr12	18407048	18407099	rs10770338	CNV_SNPs
chr12	13685790	13685841	rs10772690	CNV_SNPs
chr17	13964718	13964769	rs10775377	CNV_SNPs
chr18	26620793	26620844	rs10775524	CNV_SNPs
chr12	106214267	106214318	rs10778450	CNV_SNPs
chr1	96258899	96258950	rs10783020	CNV_SNPs
chr12	55215288	55215339	rs10783669	CNV_SNPs
chr12	60263200	60263251	rs10784009	CNV_SNPs
chr11	121779441	121779492	rs1078402	CNV_SNPs
chr1	152330919	152330970	rs10788835	CNV_SNPs
chr1	20541344	20541395	rs10799615	CNV_SNPs
chr1	169431337	169431388	rs10800447	CNV_SNPs
chr1	188746948	188746999	rs10800595	CNV_SNPs
chr1	240683463	240683514	rs10802883	CNV_SNPs
chr2	208230117	208230168	rs10804160	CNV_SNPs
chr7	1672514	1672565	rs10807969	CNV_SNPs
chr9	18995124	18995175	rs10811085	CNV_SNPs
chr9	28966272	28966323	rs10812896	CNV_SNPs
chr9	4110921	4110972	rs10814848	CNV_SNPs

chr9	126974778	126974829	rs10818929	CNV_SNPs
chr10	34717570	34717621	rs10827368	CNV_SNPs
chr11	15511564	15511615	rs10832446	CNV_SNPs
chr11	23122578	23122629	rs10833939	CNV_SNPs
chr12	20290641	20290692	rs10841451	CNV_SNPs
chr12	11664152	11664203	rs10845372	CNV_SNPs
chr12	4947306	4947357	rs10849154	CNV_SNPs
chr12	93919271	93919322	rs10859505	CNV_SNPs
chr12	93919415	93919466	rs10859508	CNV_SNPs
chr12	107194750	107194801	rs10861667	CNV_SNPs
chr2	25100712	25100763	rs10865315	CNV_SNPs
chr3	36109749	36109800	rs10865869	CNV_SNPs
chr14	38345823	38345874	rs10872905	CNV_SNPs
chr14	48351596	48351647	rs10873015	CNV_SNPs
chr12	68307496	68307547	rs10878697	CNV_SNPs
chr12	46516103	46516154	rs10880923	CNV_SNPs
chr11	111273660	111273711	rs10891259	CNV_SNPs
chr11	131904835	131904886	rs10894489	CNV_SNPs
chr11	62789105	62789156	rs10897315	CNV_SNPs
chr11	74401737	74401788	rs10899035	CNV_SNPs
chr10	128270673	128270724	rs10901638	CNV_SNPs
chr10	1510021	1510072	rs10903464	CNV_SNPs
chr9	92495050	92495101	rs10908943	CNV_SNPs
chr1	184327230	184327281	rs10911580	CNV_SNPs
chr1	169022877	169022928	rs10919044	CNV_SNPs
chr1	245977970	245978021	rs10924340	CNV_SNPs
chr9	35208119	35208170	rs10972381	CNV_SNPs
chr11	39894347	39894398	rs11035554	CNV_SNPs
chr12	117838903	117838954	rs11068469	CNV_SNPs
chr15	63766650	63766701	rs11071752	CNV_SNPs
chr15	95792247	95792298	rs11073424	CNV_SNPs
chr16	89877949	89878000	rs11076631	CNV_SNPs
chr17	68921014	68921065	rs11077530	CNV_SNPs
chr17	80782983	80783034	rs11078015	CNV_SNPs
chr17	58206113	58206164	rs11079395	CNV_SNPs
chr17	50802481	50802532	rs11080015	CNV_SNPs
chr18	30089452	30089503	rs11081772	CNV_SNPs
chr18	46385265	46385316	rs11082713	CNV_SNPs
chr21	17798934	17798985	rs11088585	CNV_SNPs
chrX	92181881	92181932	rs11091840	CNV_SNPs
chr2	21058238	21058289	rs11096687	CNV_SNPs
chr9	138663770	138663821	rs11103182	CNV_SNPs
chr9	138829683	138829734	rs11103233	CNV_SNPs
chr12	91237894	91237945	rs11105839	CNV_SNPs
chr2	79227624	79227675	rs11126691	CNV_SNPs
chr3	17744184	17744235	rs11128848	CNV_SNPs
chr3	29047647	29047698	rs11129341	CNV_SNPs
chr3	29250670	29250721	rs11129353	CNV_SNPs
chr3	43176994	43177045	rs11129993	CNV_SNPs
chr16	79109909	79109960	rs11150134	CNV_SNPs
chr18	71915647	71915698	rs11151937	CNV_SNPs
chr14	21337240	21337291	rs11156730	CNV_SNPs
chr1	75413596	75413647	rs11162256	CNV_SNPs
chr1	82939511	82939562	rs11163482	CNV_SNPs
chr12	62755605	62755656	rs11174442	CNV_SNPs
chr10	98403520	98403571	rs11188870	CNV_SNPs
chr1	66372880	66372931	rs11208766	CNV_SNPs
chr11	113282269	113282320	rs1124493	CNV_SNPs
chr15	99856823	99856874	rs11247079	CNV_SNPs
chr8	11587208	11587259	rs11250159	CNV_SNPs
chr10	29965785	29965836	rs1148210	CNV_SNPs
chr3	25569539	25569590	rs1153600	CNV_SNPs
chr1	209632537	209632588	rs1156784	CNV_SNPs
chr1	162831291	162831342	rs11588419	CNV_SNPs
chr10	49977720	49977771	rs11598212	CNV_SNPs
chr11	69069512	69069563	rs11601693	CNV_SNPs
chr12	97487805	97487856	rs11612664	CNV_SNPs
chr15	36872472	36872523	rs11638468	CNV_SNPs
chr15	98674831	98674882	rs11639134	CNV_SNPs
chr17	8383173	8383224	rs11651129	CNV_SNPs
chr6	25795551	25795602	rs1165155	CNV_SNPs
chr18	74387273	74387324	rs11660099	CNV_SNPs
chr18	53631016	53631067	rs11663406	CNV_SNPs
chr19	15890491	15890542	rs11669449	CNV_SNPs
chr2	100801172	100801223	rs11677053	CNV_SNPs
chr2	55687592	55687643	rs11694644	CNV_SNPs
chr20	35478116	35478167	rs11697412	CNV_SNPs
chr20	59927979	59928030	rs11698886	CNV_SNPs
chr3	156800358	156800409	rs11712937	CNV_SNPs
chr4	136777663	136777714	rs11723866	CNV_SNPs
chr4	6113533	6113584	rs11731332	CNV_SNPs
chr4	134635916	134635967	rs11735128	CNV_SNPs
chr5	150777971	150778022	rs11740475	CNV_SNPs
chr5	178996409	178996460	rs11743261	CNV_SNPs

chr5	52665273	52665324	rs11747505	CNV_SNPs
chr6	12518662	12518713	rs11753185	CNV_SNPs
chr7	139338107	139338158	rs11761839	CNV_SNPs
chr7	43288458	43288509	rs11771692	CNV_SNPs
chr8	10470889	10470940	rs11773913	CNV_SNPs
chr8	11096127	11096178	rs11777002	CNV_SNPs
chr7	18182729	18182780	rs1178359	CNV_SNPs
chr7	18171003	18171054	rs1178370	CNV_SNPs
chr10	110982677	110982728	rs11815325	CNV_SNPs
chr14	59556179	59556230	rs11850859	CNV_SNPs
chr15	78164680	78164731	rs11857176	CNV_SNPs
chr16	79167662	79167713	rs11862384	CNV_SNPs
chr2	193562182	193562233	rs11887586	CNV_SNPs
chr2	106021462	106021513	rs11888592	CNV_SNPs
chr2	125301476	125301527	rs11890408	CNV_SNPs
chr2	164133542	164133593	rs11891934	CNV_SNPs
chr2	7541743	7541794	rs11897624	CNV_SNPs
chr2	238791977	238792028	rs11898345	CNV_SNPs
chr2	28539851	28539902	rs11902458	CNV_SNPs
chr3	72323145	72323196	rs11922110	CNV_SNPs
chr4	28400357	28400408	rs11938446	CNV_SNPs
chr4	173704156	173704207	rs11938551	CNV_SNPs
chr5	150229775	150229826	rs11949556	CNV_SNPs
chr5	101265839	101265890	rs11952711	CNV_SNPs
chr5	40457433	40457484	rs11955354	CNV_SNPs
chr5	8453597	8453648	rs11960788	CNV_SNPs
chr8	120994368	120994419	rs11984766	CNV_SNPs
chr8	3176613	3176664	rs11991912	CNV_SNPs
chr8	66183840	66183891	rs11998282	CNV_SNPs
chr1	14192646	14192697	rs1203659	CNV_SNPs
chr11	110697279	110697330	rs120434	CNV_SNPs
chr1	35013560	35013611	rs12136079	CNV_SNPs
chr4	124344691	124344742	rs12233855	CNV_SNPs
chr1	230355412	230355463	rs12403323	CNV_SNPs
chr1	227596254	227596305	rs12408981	CNV_SNPs
chr15	94591006	94591057	rs12437942	CNV_SNPs
chr15	93545908	93545959	rs12438635	CNV_SNPs
chr15	54532114	54532165	rs12441013	CNV_SNPs
chr15	97476056	97476107	rs12442455	CNV_SNPs
chr2	133317863	133317914	rs12463443	CNV_SNPs
chr2	83593547	83593598	rs12464446	CNV_SNPs
chr2	79525007	79525058	rs12465102	CNV_SNPs
chr2	216768813	216768864	rs12465204	CNV_SNPs
chr2	76787898	76787949	rs12477610	CNV_SNPs
chr3	26806082	26806133	rs12493081	CNV_SNPs
chr3	94429146	94429197	rs12497253	CNV_SNPs
chr3	76716768	76716819	rs12497518	CNV_SNPs
chr4	67840288	67840339	rs12500111	CNV_SNPs
chr4	130125280	130125331	rs12502722	CNV_SNPs
chr4	115456085	115456136	rs12503437	CNV_SNPs
chr5	4089175	4089226	rs12513797	CNV_SNPs
chr5	29954937	29954988	rs12516563	CNV_SNPs
chr5	14009059	14009110	rs12517769	CNV_SNPs
chr6	41311866	41311917	rs12530182	CNV_SNPs
chr7	158450939	158450990	rs12533939	CNV_SNPs
chr13	106236072	106236123	rs1253820	CNV_SNPs
chr8	5498385	5498436	rs12541274	CNV_SNPs
chr8	143486672	143486723	rs12543849	CNV_SNPs
chr8	13745562	13745613	rs12544387	CNV_SNPs
chr8	90282785	90282836	rs12547059	CNV_SNPs
chr8	125874933	125874984	rs12549299	CNV_SNPs
chr9	20776717	20776768	rs12551126	CNV_SNPs
chr15	26933202	26933253	rs12593415	CNV_SNPs
chr17	63314165	63314216	rs12603034	CNV_SNPs
chr18	31705588	31705639	rs12605566	CNV_SNPs
chr19	34115522	34115573	rs12610868	CNV_SNPs
chr2	226701382	226701433	rs12612024	CNV_SNPs
chr2	185198399	185198450	rs12616371	CNV_SNPs
chr4	29722971	29723022	rs12641943	CNV_SNPs
chr4	67191513	67191564	rs12643470	CNV_SNPs
chr4	155454261	155454312	rs12649647	CNV_SNPs
chr5	154936186	154936237	rs12655584	CNV_SNPs
chr5	4577816	4577867	rs12657445	CNV_SNPs
chr5	72671712	72671763	rs12659148	CNV_SNPs
chr7	67073991	67074042	rs12667043	CNV_SNPs
chr7	154594045	154594096	rs12667380	CNV_SNPs
chr7	2020969	2021020	rs12668848	CNV_SNPs
chr8	29496736	29496787	rs12676327	CNV_SNPs
chr8	3489730	3489781	rs12678397	CNV_SNPs
chr9	129469513	129469564	rs12684797	CNV_SNPs
chrX	39945599	39945650	rs12687359	CNV_SNPs
chr14	20347538	20347589	rs1319954	CNV_SNPs
chr13	43603845	43603896	rs1323862	CNV_SNPs
chr22	45134171	45134222	rs132410	CNV_SNPs

chr1	102237792	102237843	rs1327583	CNV_SNPs
chr13	68930959	68931010	rs1329680	CNV_SNPs
chr9	122204778	122204829	rs1331624	CNV_SNPs
chr22	25770764	25770815	rs133216	CNV_SNPs
chr9	31112831	31112882	rs1335143	CNV_SNPs
chr1	232455377	232455428	rs1338519	CNV_SNPs
chr10	15950123	15950174	rs1339474	CNV_SNPs
chr10	9959759	9959810	rs1339673	CNV_SNPs
chr13	104378156	104378207	rs1341438	CNV_SNPs
chr10	108316036	108316087	rs1341675	CNV_SNPs
chr1	118346193	118346244	rs1342813	CNV_SNPs
chr8	132408483	132408534	rs1346820	CNV_SNPs
chr11	43266808	43266859	rs1353461	CNV_SNPs
chr2	50178125	50178176	rs1363046	CNV_SNPs
chr18	28787498	28787549	rs1365294	CNV_SNPs
chr10	60277523	60277574	rs1365740	CNV_SNPs
chr4	156646934	156646985	rs1384623	CNV_SNPs
chr3	112547575	112547626	rs1387024	CNV_SNPs
chr11	7206537	7206588	rs1395907	CNV_SNPs
chr2	142098136	142098187	rs1402473	CNV_SNPs
chr2	154688702	154688753	rs1406084	CNV_SNPs
chr10	109599363	109599414	rs1411289	CNV_SNPs
chr9	28385646	28385697	rs1412227	CNV_SNPs
chr1	221345716	221345767	rs1417922	CNV_SNPs
chr7	97097061	97097112	rs1420175	CNV_SNPs
chr3	34786430	34786481	rs1420518	CNV_SNPs
chr19	53812497	53812548	rs1428642	CNV_SNPs
chr2	141599192	141599243	rs1429369	CNV_SNPs
chr2	222202836	222202887	rs1430230	CNV_SNPs
chr4	65604590	65604641	rs1430526	CNV_SNPs
chr14	87813005	87813056	rs1430564	CNV_SNPs
chr2	82017274	82017325	rs1432154	CNV_SNPs
chr2	79422939	79422990	rs1434199	CNV_SNPs
chr2	158739558	158739609	rs1441136	CNV_SNPs
chr12	61873061	61873112	rs1453703	CNV_SNPs
chr3	36515690	36515741	rs1456078	CNV_SNPs
chr1	234272861	234272912	rs1458590	CNV_SNPs
chr4	118610357	118610408	rs1459551	CNV_SNPs
chr3	118605443	118605494	rs1464413	CNV_SNPs
chr1	219421902	219421953	rs1474372	CNV_SNPs
chr3	22112034	22112085	rs1479996	CNV_SNPs
chrX	97019175	97019226	rs1481156	CNV_SNPs
chr8	120071872	120071923	rs1485315	CNV_SNPs
chr12	55427285	55427336	rs1488049	CNV_SNPs
chr3	2001776	2001827	rs1488745	CNV_SNPs
chr11	27795512	27795563	rs1491868	CNV_SNPs
chr7	24164598	24164649	rs1494188	CNV_SNPs
chr7	97826206	97826257	rs1495525	CNV_SNPs
chr11	88359605	88359656	rs1504081	CNV_SNPs
chr2	6851243	6851294	rs1505696	CNV_SNPs
chr12	43131105	43131156	rs1517662	CNV_SNPs
chr5	126841094	126841145	rs151796	CNV_SNPs
chr4	157519070	157519121	rs1521696	CNV_SNPs
chr7	16603634	16603685	rs1524358	CNV_SNPs
chr12	77781845	77781896	rs1527062	CNV_SNPs
chr2	183485132	183485183	rs1527879	CNV_SNPs
chr16	31691886	31691937	rs1528317	CNV_SNPs
chr1	118223958	118224009	rs1529968	CNV_SNPs
chr2	120048703	120048754	rs1530562	CNV_SNPs
chr7	121090833	121090884	rs1534520	CNV_SNPs
chr6	76598878	76598929	rs1535501	CNV_SNPs
chr6	72558554	72558605	rs1536766	CNV_SNPs
chr11	20575761	20575812	rs1540186	CNV_SNPs
chr6	32877147	32877198	rs154989	CNV_SNPs
chr7	155835530	155835581	rs1551634	CNV_SNPs
chr21	41918932	41918983	rs1554936	CNV_SNPs
chr3	1449846	1449897	rs155864	CNV_SNPs
chr5	139517022	139517073	rs156095	CNV_SNPs
chr6	162170013	162170064	rs1569836	CNV_SNPs
chr6	90009285	90009336	rs1570932	CNV_SNPs
chr16	52085567	52085618	rs1582317	CNV_SNPs
chr5	58205554	58205605	rs159734	CNV_SNPs
chr10	18471935	18471986	rs1624525	CNV_SNPs
chr7	87600900	87600951	rs1637497	CNV_SNPs
chr15	45468600	45468651	rs1648306	CNV_SNPs
chr22	21135737	21135788	rs165583	CNV_SNPs
chr8	565206	565257	rs1669694	CNV_SNPs
chr12	104975183	104975234	rs167510	CNV_SNPs
chr10	29108029	29108080	rs1691921	CNV_SNPs
chrY	19370890	19370941	rs16980598	CNV_SNPs
chrX	32172558	32172609	rs1718052	CNV_SNPs
chr1	7520103	7520154	rs1725235	CNV_SNPs
chr2	34654103	34654154	rs17260872	CNV_SNPs
chrX	93434409	93434460	rs1736839	CNV_SNPs

chrX	73434921	73434972	rs174211	CNV_SNPs
chr14	104540829	104540880	rs1744266	CNV_SNPs
chr20	36971093	36971144	rs1780615	CNV_SNPs
chr11	131050868	131050919	rs1793609	CNV_SNPs
chr3	194238364	194238415	rs1807193	CNV_SNPs
chr15	87255722	87255773	rs1814089	CNV_SNPs
chr10	119379061	119379112	rs181652	CNV_SNPs
chr12	65379347	65379398	rs1822929	CNV_SNPs
chr2	14527990	14528041	rs1829065	CNV_SNPs
chr11	127795820	127795871	rs1834311	CNV_SNPs
chr2	39842114	39842165	rs1861242	CNV_SNPs
chr3	187304043	187304094	rs1863384	CNV_SNPs
chr2	240678176	240678227	rs1866136	CNV_SNPs
chr13	93770307	93770358	rs1867437	CNV_SNPs
chr5	33817313	33817364	rs1867718	CNV_SNPs
chr22	25494718	25494769	rs1883278	CNV_SNPs
chr6	39338409	39338460	rs1885615	CNV_SNPs
chr13	43549995	43550046	rs1886152	CNV_SNPs
chr13	99566409	99566460	rs1886551	CNV_SNPs
chr9	27687133	27687184	rs1888171	CNV_SNPs
chr2	193538794	193538845	rs1898917	CNV_SNPs
chrX	25314696	25314747	rs1904925	CNV_SNPs
chr3	61187171	61187222	rs1916800	CNV_SNPs
chr4	28143831	28143882	rs1918319	CNV_SNPs
chr7	78902658	78902709	rs1918939	CNV_SNPs
chr13	46833124	46833175	rs1926005	CNV_SNPs
chr6	82610162	82610213	rs1931656	CNV_SNPs
chr11	132534844	132534895	rs1939508	CNV_SNPs
chr11	121939950	121940001	rs1944463	CNV_SNPs
chr6	82467081	82467132	rs194609	CNV_SNPs
chr8	87558257	87558308	rs1946940	CNV_SNPs
chr10	53238089	53238140	rs1947060	CNV_SNPs
chr14	49557420	49557471	rs1950605	CNV_SNPs
chr14	27035086	27035137	rs1951073	CNV_SNPs
chr14	83401732	83401783	rs1959730	CNV_SNPs
chr10	90918688	90918739	rs1977501	CNV_SNPs
chr19	17193525	17193576	rs1979260	CNV_SNPs
chr16	2781817	2781868	rs1981539	CNV_SNPs
chr12	81163991	81164042	rs1982909	CNV_SNPs
chr1	214977339	214977390	rs1993032	CNV_SNPs
chr7	23293720	23293771	rs199347	CNV_SNPs
chr15	71950445	71950496	rs1995337	CNV_SNPs
chr4	157615557	157615608	rs1996770	CNV_SNPs
chrX	93324954	93325005	rs1999923	CNV_SNPs
chr20	51970935	51970986	rs200630	CNV_SNPs
chr20	21116276	21116327	rs2024937	CNV_SNPs
chr15	63054413	63054464	rs2030040	CNV_SNPs
chr2	191869137	191869188	rs2030171	CNV_SNPs
chr2	17675709	17675760	rs2030328	CNV_SNPs
chr5	44273647	44273698	rs2034347	CNV_SNPs
chr16	82113445	82113496	rs2042429	CNV_SNPs
chr2	134775318	134775369	rs2046475	CNV_SNPs
chr18	29691581	29691632	rs2048245	CNV_SNPs
chr7	124270548	124270599	rs2049438	CNV_SNPs
chr1	158507158	158507209	rs2051068	CNV_SNPs
chr5	147496281	147496332	rs2052532	CNV_SNPs
chr13	106826352	106826403	rs2053530	CNV_SNPs
chr5	135406632	135406683	rs2058043	CNV_SNPs
chrX	49070787	49070838	rs2071317	CNV_SNPs
chr6	46910389	46910440	rs2077055	CNV_SNPs
chr17	10552462	10552513	rs2097657	CNV_SNPs
chr10	43052954	43053005	rs210219	CNV_SNPs
chr18	10151862	10151913	rs2110540	CNV_SNPs
chr3	188183906	188183957	rs2114239	CNV_SNPs
chr3	60459175	60459226	rs2121863	CNV_SNPs
chr3	6610042	6610093	rs2129907	CNV_SNPs
chr17	6847047	6847098	rs2135845	CNV_SNPs
chr7	144662648	144662699	rs2140849	CNV_SNPs
chr1	35468403	35468454	rs2151554	CNV_SNPs
chr4	15499225	15499276	rs2160043	CNV_SNPs
chr9	125767514	125767565	rs2167128	CNV_SNPs
chr12	62290630	62290681	rs2168454	CNV_SNPs
chr5	137431475	137431526	rs217256	CNV_SNPs
chr10	68821546	68821597	rs2182161	CNV_SNPs
chr7	70859465	70859516	rs2189930	CNV_SNPs
chr7	91179865	91179916	rs2192804	CNV_SNPs
chr2	57016366	57016417	rs2193624	CNV_SNPs
chr10	10923405	10923456	rs2208952	CNV_SNPs
chr14	61884620	61884671	rs2209388	CNV_SNPs
chr14	57141577	57141628	rs2210034	CNV_SNPs
chr7	153622356	153622407	rs2215195	CNV_SNPs
chr2	225069342	225069393	rs2219088	CNV_SNPs
chrX	145961863	145961914	rs2225832	CNV_SNPs
chr1	4849358	4849409	rs2235438	CNV_SNPs

chr16	1601261	1601312	rs2235646	CNV_SNPs
chr22	19053380	19053431	rs2238749	CNV_SNPs
chr2	217736105	217736156	rs2241191	CNV_SNPs
chr11	8852213	8852264	rs2243566	CNV_SNPs
chr7	50739429	50739480	rs2244353	CNV_SNPs
chr10	56851370	56851421	rs2246693	CNV_SNPs
chr17	986716	986767	rs2257468	CNV_SNPs
chr17	953430	953481	rs2257540	CNV_SNPs
chr20	22964773	22964824	rs2257766	CNV_SNPs
chr11	110665888	110665939	rs226111	CNV_SNPs
chrX	132467697	132467748	rs2266802	CNV_SNPs
chr22	44445633	44445684	rs2267600	CNV_SNPs
chr3	39453031	39453082	rs2269349	CNV_SNPs
chr1	172415425	172415476	rs2269615	CNV_SNPs
chr5	161322863	161322914	rs2279020	CNV_SNPs
chr11	2583115	2583166	rs2283170	CNV_SNPs
chr19	58288143	58288194	rs2285615	CNV_SNPs
chr17	74681409	74681460	rs2286593	CNV_SNPs
chr17	30696266	30696317	rs2286645	CNV_SNPs
chr18	43015506	43015557	rs2287434	CNV_SNPs
chr18	42288451	42288502	rs2290932	CNV_SNPs
chr2	179632684	179632735	rs2291313	CNV_SNPs
chr9	421954	422005	rs2297081	CNV_SNPs
chr5	21760576	21760627	rs2303748	CNV_SNPs
chr13	67591701	67591752	rs2325020	CNV_SNPs
chr7	45127256	45127307	rs2331207	CNV_SNPs
chr21	28399156	28399207	rs233621	CNV_SNPs
chr22	29413975	29414026	rs2347790	CNV_SNPs
chr14	50455061	50455112	rs2355654	CNV_SNPs
chr7	150486057	150486108	rs2373852	CNV_SNPs
chr2	141641903	141641954	rs2380943	CNV_SNPs
chr6	100315001	100315052	rs2397660	CNV_SNPs
chr12	21591895	21591946	rs2417982	CNV_SNPs
chr10	111500874	111500925	rs2419304	CNV_SNPs
chr2	60170849	60170900	rs2419407	CNV_SNPs
chr12	22968352	22968403	rs2433651	CNV_SNPs
chrX	91420480	91420531	rs2437447	CNV_SNPs
chr8	6327248	6327299	rs2442496	CNV_SNPs
chr13	54073443	54073494	rs2488375	CNV_SNPs
chr6	40588269	40588320	rs2494971	CNV_SNPs
chr10	36854013	36854064	rs2503069	CNV_SNPs
chr10	38372638	38372689	rs2505257	CNV_SNPs
chr7	86544641	86544692	rs2519713	CNV_SNPs
chr5	142329119	142329170	rs252234	CNV_SNPs
chr2	159560022	159560073	rs2528611	CNV_SNPs
chr6	31053231	31053282	rs2535310	CNV_SNPs
chr5	53875455	53875506	rs2548632	CNV_SNPs
chr11	35053791	35053842	rs2553794	CNV_SNPs
chr13	33833784	33833835	rs2555605	CNV_SNPs
chr5	162712711	162712762	rs2560373	CNV_SNPs
chr2	85408525	85408576	rs2568201	CNV_SNPs
chr18	55466570	55466621	rs2571219	CNV_SNPs
chr8	59790184	59790235	rs2594953	CNV_SNPs
chr3	12955956	12956007	rs2596902	CNV_SNPs
chr11	68758483	68758534	rs2602822	CNV_SNPs
chr12	78288785	78288836	rs2619062	CNV_SNPs
chr17	4540251	4540302	rs2619118	CNV_SNPs
chr11	19498834	19498885	rs2632032	CNV_SNPs
chr8	131862793	131862844	rs263240	CNV_SNPs
chr7	56749181	56749232	rs2634103	CNV_SNPs
chr3	87348040	87348091	rs2646218	CNV_SNPs
chr12	106589632	106589683	rs2649712	CNV_SNPs
chr5	79172110	79172161	rs265005	CNV_SNPs
chr11	99322295	99322346	rs2656176	CNV_SNPs
chr6	122329241	122329292	rs2679665	CNV_SNPs
chr8	103858282	103858333	rs2679748	CNV_SNPs
chr3	24205225	24205276	rs2683544	CNV_SNPs
chr6	122329944	122329995	rs2684222	CNV_SNPs
chr9	114912591	114912642	rs2691669	CNV_SNPs
chr10	71539555	71539606	rs2704476	CNV_SNPs
chr1	70742037	70742088	rs270487	CNV_SNPs
chr18	68060697	68060748	rs2711419	CNV_SNPs
chr7	111820064	111820115	rs2729544	CNV_SNPs
chr7	41461174	41461225	rs273172	CNV_SNPs
chr19	51805292	51805343	rs273628	CNV_SNPs
chr6	93108665	93108716	rs2757027	CNV_SNPs
chr1	157599891	157599942	rs2758629	CNV_SNPs
chr14	85173201	85173252	rs2765909	CNV_SNPs
chr10	38542562	38542613	rs2800485	CNV_SNPs
chr6	34617118	34617169	rs2814992	CNV_SNPs
chr6	39190261	39190312	rs2815118	CNV_SNPs
chr9	113560093	113560144	rs2821143	CNV_SNPs
chr21	20195176	20195227	rs2825168	CNV_SNPs
chr6	118629839	118629890	rs283044	CNV_SNPs

chr21	31384271	31384322	rs2832533	CNV_SNPs
chr21	37557448	37557499	rs2835302	CNV_SNPs
chr18	10451669	10451720	rs2847348	CNV_SNPs
chr8	97963785	97963836	rs2853252	CNV_SNPs
chr4	187367513	187367564	rs2889190	CNV_SNPs
chr7	64721520	64721571	rs2900628	CNV_SNPs
chr7	81076806	81076857	rs2906231	CNV_SNPs
chr12	3001318	3001369	rs2907603	CNV_SNPs
chr17	11396351	11396402	rs2908977	CNV_SNPs
chr7	2640352	2640403	rs2917728	CNV_SNPs
chr18	19020924	19020975	rs291783	CNV_SNPs
chr18	9924591	9924642	rs29193	CNV_SNPs
chr1	202188615	202188666	rs2924105	CNV_SNPs
chr1	113234430	113234481	rs2932532	CNV_SNPs
chr5	14869889	14869940	rs2934813	CNV_SNPs
chr8	111298517	111298568	rs2948998	CNV_SNPs
chr15	74566999	74567050	rs2959019	CNV_SNPs
chr5	173763330	173763381	rs2964101	CNV_SNPs
chr5	5492692	5492743	rs2964131	CNV_SNPs
chr8	6710331	6710382	rs2977794	CNV_SNPs
chr5	172573741	172573792	rs29798	CNV_SNPs
chr1	59564347	59564398	rs2989871	CNV_SNPs
chr14	51605975	51606026	rs2999357	CNV_SNPs
chr2	17986657	17986708	rs300152	CNV_SNPs
chr16	27356641	27356692	rs3024560	CNV_SNPs
chr1	99589398	99589449	rs303387	CNV_SNPs
chr7	154799067	154799118	rs306288	CNV_SNPs
chr16	55329575	55329626	rs31042	CNV_SNPs
chr6	29230657	29230708	rs3130827	CNV_SNPs
chr1	83024666	83024717	rs318416	CNV_SNPs
chr17	31030690	31030741	rs321176	CNV_SNPs
chr7	80802084	80802135	rs327677	CNV_SNPs
chr5	133908352	133908403	rs329321	CNV_SNPs
chr3	159601737	159601788	rs33053	CNV_SNPs
chr5	68390258	68390309	rs337259	CNV_SNPs
chr1	226064719	226064770	rs360093	CNV_SNPs
chr8	53901260	53901311	rs360956	CNV_SNPs
chr7	103275777	103275828	rs362646	CNV_SNPs
chr7	103214914	103214965	rs362813	CNV_SNPs
chr15	62266224	62266275	rs3743294	CNV_SNPs
chr4	25133643	25133694	rs3756208	CNV_SNPs
chr2	173914749	173914800	rs3769210	CNV_SNPs
chr2	216826580	216826631	rs3770530	CNV_SNPs
chr7	2166488	2166539	rs3778984	CNV_SNPs
chr12	68617193	68617244	rs3782553	CNV_SNPs
chr14	61799745	61799796	rs3783816	CNV_SNPs
chr15	88610431	88610482	rs3784429	CNV_SNPs
chr15	48633066	48633117	rs3784621	CNV_SNPs
chr1	66042052	66042103	rs3790428	CNV_SNPs
chr6	41460261	41460312	rs3800282	CNV_SNPs
chr1	120199163	120199214	rs380155	CNV_SNPs
chr8	12586235	12586286	rs3802268	CNV_SNPs
chr13	111119051	111119102	rs3803234	CNV_SNPs
chr7	123382264	123382315	rs3807634	CNV_SNPs
chrX	71788844	71788895	rs3817725	CNV_SNPs
chr6	25092513	25092564	rs3818270	CNV_SNPs
chr14	73128957	73129008	rs3854	CNV_SNPs
chr6	153412450	153412501	rs3910736	CNV_SNPs
chr10	49842629	49842680	rs3910910	CNV_SNPs
chr3	180017398	180017449	rs3914871	CNV_SNPs
chrX	25648131	25648182	rs3922856	CNV_SNPs
chr4	183806878	183806929	rs3924787	CNV_SNPs
chr19	28780436	28780487	rs3960412	CNV_SNPs
chr6	5106873	5106924	rs402541	CNV_SNPs
chr16	8382854	8382905	rs40644	CNV_SNPs
chr6	136294957	136295008	rs4072488	CNV_SNPs
chr7	32894477	32894528	rs4101647	CNV_SNPs
chr5	75927162	75927213	rs410262	CNV_SNPs
chr21	37954696	37954747	rs412510	CNV_SNPs
chr4	105409628	105409679	rs4129288	CNV_SNPs
chr13	109078673	109078724	rs4144092	CNV_SNPs
chr8	128343853	128343904	rs420101	CNV_SNPs
chr7	19616411	19616462	rs4236296	CNV_SNPs
chr7	107730723	107730774	rs423686	CNV_SNPs
chr22	26790859	26790910	rs4239922	CNV_SNPs
chr4	190262460	190262511	rs4241875	CNV_SNPs
chr5	2979684	2979735	rs4242095	CNV_SNPs
chr16	79980399	79980450	rs4258627	CNV_SNPs
chr4	48356307	48356358	rs429969	CNV_SNPs
chr21	43995897	43995948	rs430807	CNV_SNPs
chr4	166310695	166310746	rs4308333	CNV_SNPs
chr4	28204079	28204130	rs4318636	CNV_SNPs
chr7	3372223	3372274	rs4327753	CNV_SNPs
chr14	64619824	64619875	rs4329843	CNV_SNPs

chr18	44240500	44240551	rs435202	CNV_SNPs
chr16	58529589	58529640	rs4356470	CNV_SNPs
chr10	117888412	117888463	rs4370849	CNV_SNPs
chr3	38547995	38548046	rs4371464	CNV_SNPs
chr15	71892189	71892240	rs4398066	CNV_SNPs
chr15	63995397	63995448	rs4411464	CNV_SNPs
chr10	121932807	121932858	rs4427485	CNV_SNPs
chr4	1402624	1402675	rs4441710	CNV_SNPs
chr13	102276743	102276794	rs4451831	CNV_SNPs
chr8	84050866	84050917	rs4481591	CNV_SNPs
chr5	77168064	77168115	rs4490567	CNV_SNPs
chr13	25694314	25694365	rs4491357	CNV_SNPs
chr11	21635256	21635307	rs4495891	CNV_SNPs
chr9	10094872	10094923	rs450818	CNV_SNPs
chr10	10136313	10136364	rs4509669	CNV_SNPs
chr9	75047035	75047086	rs4526421	CNV_SNPs
chr9	13731175	13731226	rs4536526	CNV_SNPs
chr4	176935608	176935659	rs4536875	CNV_SNPs
chr21	27340275	27340326	rs454312	CNV_SNPs
chr18	34882906	34882957	rs4567809	CNV_SNPs
chr17	77453337	77453388	rs4572435	CNV_SNPs
chr4	28746066	28746117	rs4591585	CNV_SNPs
chr3	32670331	32670382	rs4608730	CNV_SNPs
chr6	98417121	98417172	rs4610575	CNV_SNPs
chr17	114643	114694	rs4617924	CNV_SNPs
chr8	24533167	24533218	rs4625043	CNV_SNPs
chr16	26320113	26320164	rs4643323	CNV_SNPs
chr21	31252285	31252336	rs464500	CNV_SNPs
chr1	22587852	22587903	rs4655036	CNV_SNPs
chr1	170835736	170835787	rs4656809	CNV_SNPs
chr2	66352769	66352820	rs4671714	CNV_SNPs
chr2	58496250	58496301	rs4672233	CNV_SNPs
chr2	223361127	223361178	rs4673024	CNV_SNPs
chr2	203723963	203724014	rs4673238	CNV_SNPs
chr3	75139661	75139712	rs4677451	CNV_SNPs
chr3	33432281	33432332	rs4678951	CNV_SNPs
chr3	154880344	154880395	rs4679739	CNV_SNPs
chr5	58088324	58088375	rs4700308	CNV_SNPs
chr6	45556043	45556094	rs4711819	CNV_SNPs
chr6	17627564	17627615	rs4716167	CNV_SNPs
chr7	151430683	151430734	rs4726088	CNV_SNPs
chr7	135681492	135681543	rs4732165	CNV_SNPs
chr8	139766452	139766503	rs4736186	CNV_SNPs
chr8	70250381	70250432	rs4737959	CNV_SNPs
chr8	86485280	86485331	rs4740054	CNV_SNPs
chr9	3206132	3206183	rs4741818	CNV_SNPs
chr10	6176140	6176191	rs4747886	CNV_SNPs
chr10	133191802	133191853	rs4751376	CNV_SNPs
chr11	44368355	44368406	rs4755815	CNV_SNPs
chr11	39878796	39878847	rs4756547	CNV_SNPs
chr8	109235832	109235883	rs475865	CNV_SNPs
chr16	3498925	3498976	rs4786416	CNV_SNPs
chr17	72939420	72939471	rs4789110	CNV_SNPs
chr17	77280312	77280363	rs4789882	CNV_SNPs
chr17	31819572	31819623	rs4795799	CNV_SNPs
chr18	2548075	2548126	rs4797070	CNV_SNPs
chr19	33906097	33906148	rs4805885	CNV_SNPs
chr20	55178055	55178106	rs4811719	CNV_SNPs
chr22	41461810	41461861	rs4821995	CNV_SNPs
chrX	122696537	122696588	rs4825872	CNV_SNPs
chr3	48515053	48515104	rs4858795	CNV_SNPs
chr13	75909170	75909221	rs485988	CNV_SNPs
chr4	65763566	65763617	rs4860174	CNV_SNPs
chr16	78785537	78785588	rs4888839	CNV_SNPs
chr16	31011157	31011208	rs4889606	CNV_SNPs
chrX	3085438	3085489	rs4892924	CNV_SNPs
chr3	104282508	104282559	rs4895085	CNV_SNPs
chr14	104146335	104146386	rs4900589	CNV_SNPs
chr14	52640538	52640589	rs4901224	CNV_SNPs
chr5	88365752	88365803	rs4916667	CNV_SNPs
chr11	110644506	110644557	rs4937385	CNV_SNPs
chr13	41032433	41032484	rs4941979	CNV_SNPs
chr11	76059203	76059254	rs4945074	CNV_SNPs
chr10	45386020	45386071	rs4948924	CNV_SNPs
chr1	30794934	30794985	rs4949254	CNV_SNPs
chr1	147282420	147282471	rs4950346	CNV_SNPs
chr2	44561506	44561557	rs4953086	CNV_SNPs
chr2	47257839	47257890	rs4953445	CNV_SNPs
chr5	151755990	151756041	rs4958301	CNV_SNPs
chr6	6269272	6269323	rs4960181	CNV_SNPs
chr8	141840855	141840906	rs4961234	CNV_SNPs
chr9	16206734	16206785	rs4961670	CNV_SNPs
chr10	127191096	127191147	rs4962305	CNV_SNPs
chr12	27330490	27330541	rs4964049	CNV_SNPs

chr15	99226122	99226173	rs4966012	CNV_SNPs
chr5	134389663	134389714	rs4976264	CNV_SNPs
chr9	113137374	113137425	rs4978425	CNV_SNPs
chr8	56063780	56063831	rs5008177	CNV_SNPs
chr16	7032428	7032479	rs5015420	CNV_SNPs
chr9	3253587	3253638	rs527697	CNV_SNPs
chr18	7023568	7023619	rs529821	CNV_SNPs
chr11	83269165	83269216	rs533209	CNV_SNPs
chr11	100850176	100850227	rs533571	CNV_SNPs
chr1	185520574	185520625	rs534817	CNV_SNPs
chr18	2224716	2224767	rs564201	CNV_SNPs
chr1	190734025	190734076	rs567695	CNV_SNPs
chr22	30937281	30937332	rs5749122	CNV_SNPs
chr8	16704073	16704124	rs576762	CNV_SNPs
chr6	163528922	163528973	rs578974	CNV_SNPs
chr6	163517009	163517060	rs579172	CNV_SNPs
chr11	30098624	30098675	rs588231	CNV_SNPs
chrX	140519341	140519392	rs5907921	CNV_SNPs
chrX	145042078	145042129	rs5919974	CNV_SNPs
chrX	22516314	22516365	rs5925700	CNV_SNPs
chrX	125318915	125318966	rs5933384	CNV_SNPs
chrX	13078749	13078800	rs5935490	CNV_SNPs
chrX	69254734	69254785	rs5936531	CNV_SNPs
chr1	182696091	182696142	rs595289	CNV_SNPs
chr11	30254524	30254575	rs595496	CNV_SNPs
chrX	8703385	8703436	rs5978265	CNV_SNPs
chrX	26693479	26693530	rs5986635	CNV_SNPs
chr22	33828970	33829021	rs5998941	CNV_SNPs
chr22	45714911	45714962	rs6006984	CNV_SNPs
chr20	58196398	58196449	rs6015534	CNV_SNPs
chr12	52921253	52921304	rs602017	CNV_SNPs
chr20	345838	345889	rs6051490	CNV_SNPs
chr20	7600987	7601038	rs6055187	CNV_SNPs
chr20	60426087	60426138	rs6061867	CNV_SNPs
chr20	41956894	41956945	rs6073049	CNV_SNPs
chr20	1421906	1421957	rs6079138	CNV_SNPs
chr20	16142307	16142358	rs6080154	CNV_SNPs
chr20	6952545	6952596	rs6085786	CNV_SNPs
chr20	48451103	48451154	rs6095681	CNV_SNPs
chr20	58054422	58054473	rs6100460	CNV_SNPs
chr1	234802241	234802292	rs610329	CNV_SNPs
chr20	2135879	2135930	rs6113024	CNV_SNPs
chr8	36092395	36092446	rs611313	CNV_SNPs
chr20	39924604	39924655	rs6129804	CNV_SNPs
chr20	15770357	15770408	rs6135517	CNV_SNPs
chr20	19414921	19414972	rs6136727	CNV_SNPs
chr1	117273508	117273559	rs614516	CNV_SNPs
chr12	50412885	50412936	rs615382	CNV_SNPs
chr13	103337331	103337382	rs630706	CNV_SNPs
chr18	66080177	66080228	rs636909	CNV_SNPs
chr20	19506703	19506754	rs642189	CNV_SNPs
chr1	4955836	4955887	rs6426446	CNV_SNPs
chr2	241362974	241363025	rs6437337	CNV_SNPs
chr3	95454442	95454493	rs6438074	CNV_SNPs
chr3	123714956	123715007	rs6438822	CNV_SNPs
chr4	46047380	46047431	rs6447494	CNV_SNPs
chr11	128571231	128571282	rs644818	CNV_SNPs
chr5	34605299	34605350	rs6451151	CNV_SNPs
chr6	68164932	68164983	rs6455233	CNV_SNPs
chr7	12252715	12252766	rs6460897	CNV_SNPs
chr7	16584444	16584495	rs6461264	CNV_SNPs
chr7	4798507	4798558	rs6463033	CNV_SNPs
chr7	46780588	46780639	rs6463347	CNV_SNPs
chr8	121520030	121520081	rs6469932	CNV_SNPs
chr9	20781953	20782004	rs6475473	CNV_SNPs
chr11	13127867	13127918	rs6486088	CNV_SNPs
chr13	99178250	99178301	rs6491430	CNV_SNPs
chr15	39564267	39564318	rs6492866	CNV_SNPs
chr2	67673904	67673955	rs650129	CNV_SNPs
chr17	31889928	31889979	rs6505356	CNV_SNPs
chrX	106444183	106444234	rs6523931	CNV_SNPs
chr4	134134283	134134334	rs6534936	CNV_SNPs
chrX	147332654	147332705	rs6540406	CNV_SNPs
chr2	86184460	86184511	rs6547651	CNV_SNPs
chr10	2158541	2158592	rs6560798	CNV_SNPs
chr13	34299207	34299258	rs6562039	CNV_SNPs
chr18	59254112	59254163	rs6567221	CNV_SNPs
chr14	57255575	57255626	rs6573105	CNV_SNPs
chr14	57732997	57733048	rs6573142	CNV_SNPs
chr14	80952082	80952133	rs6574581	CNV_SNPs
chr14	20893193	20893244	rs6575809	CNV_SNPs
chr10	103249010	103249061	rs6584424	CNV_SNPs
chr21	43174410	43174461	rs6586235	CNV_SNPs
chr11	86333408	86333459	rs6592302	CNV_SNPs

chr16	115046	115097	rs6600227	CNV_SNPs
chrX	149924712	149924763	rs6627327	CNV_SNPs
chrX	451116	451167	rs6645103	CNV_SNPs
chrY	401116	401167	rs6645103	CNV_SNPs
chr1	108079919	108079970	rs666003	CNV_SNPs
chr1	36411711	36411762	rs6665591	CNV_SNPs
chr1	116333708	116333759	rs6669078	CNV_SNPs
chr1	71762177	71762228	rs6695355	CNV_SNPs
chr1	53811916	53811967	rs6698595	CNV_SNPs
chr2	20616399	20616450	rs6710671	CNV_SNPs
chr2	99373694	99373745	rs6712597	CNV_SNPs
chr2	133919401	133919452	rs6716343	CNV_SNPs
chr2	222085587	222085638	rs6719473	CNV_SNPs
chr2	108623296	108623347	rs6720783	CNV_SNPs
chr2	125424586	125424637	rs6750530	CNV_SNPs
chr2	5429234	5429285	rs6759683	CNV_SNPs
chr3	63597356	63597407	rs6774940	CNV_SNPs
chr3	23146656	23146707	rs6783710	CNV_SNPs
chr3	55235599	55235650	rs6785046	CNV_SNPs
chr3	174257048	174257099	rs6800681	CNV_SNPs
chr3	116506445	116506496	rs6806089	CNV_SNPs
chr3	75164833	75164884	rs6806344	CNV_SNPs
chr6	65096771	65096822	rs681665	CNV_SNPs
chr4	145225972	145226023	rs6821055	CNV_SNPs
chr4	114757188	114757239	rs6823304	CNV_SNPs
chr4	41333435	41333486	rs6830887	CNV_SNPs
chr4	78271594	78271645	rs6831412	CNV_SNPs
chr4	94653668	94653719	rs6837139	CNV_SNPs
chr4	142367714	142367765	rs6837853	CNV_SNPs
chr4	190093220	190093271	rs6848521	CNV_SNPs
chr4	24030735	24030786	rs6858568	CNV_SNPs
chr5	111983015	111983066	rs6860330	CNV_SNPs
chr5	96383726	96383777	rs6861399	CNV_SNPs
chr5	127196040	127196091	rs6864687	CNV_SNPs
chr5	30031889	30031940	rs6885929	CNV_SNPs
chr5	116415218	116415269	rs6889147	CNV_SNPs
chr3	16403740	16403791	rs690343	CNV_SNPs
chr6	166653937	166653988	rs6909545	CNV_SNPs
chr6	137723518	137723569	rs6912317	CNV_SNPs
chr6	148266727	148266778	rs6919109	CNV_SNPs
chr6	121788705	121788756	rs6919748	CNV_SNPs
chr6	164341194	164341245	rs6921880	CNV_SNPs
chr6	3797831	3797882	rs6925590	CNV_SNPs
chr6	106051712	106051763	rs6933379	CNV_SNPs
chr7	37220906	37220957	rs6946785	CNV_SNPs
chr7	78867384	78867435	rs6947782	CNV_SNPs
chr7	47802482	47802533	rs6953684	CNV_SNPs
chr7	53722854	53722905	rs6964140	CNV_SNPs
chr8	102984253	102984304	rs6982161	CNV_SNPs
chr8	18916964	18917015	rs6982849	CNV_SNPs
chr8	65193111	65193162	rs6982872	CNV_SNPs
chr8	38736063	38736114	rs6984966	CNV_SNPs
chr8	142098951	142099002	rs6990155	CNV_SNPs
chr12	63294833	63294884	rs699576	CNV_SNPs
chr8	31397722	31397773	rs7006706	CNV_SNPs
chr7	83542147	83542198	rs701286	CNV_SNPs
chr8	131098136	131098187	rs7015970	CNV_SNPs
chr8	2387950	2388001	rs7017910	CNV_SNPs
chr9	100968572	100968623	rs7029585	CNV_SNPs
chr9	116791135	116791186	rs7030167	CNV_SNPs
chr9	102136151	102136202	rs7030379	CNV_SNPs
chr9	79231313	79231364	rs7031280	CNV_SNPs
chr9	5754962	5755013	rs7038447	CNV_SNPs
chrX	115503892	115503943	rs7057233	CNV_SNPs
chr10	3280638	3280689	rs7071984	CNV_SNPs
chr10	16762497	16762548	rs7084555	CNV_SNPs
chr10	88866103	88866154	rs7087038	CNV_SNPs
chr10	8596750	8596801	rs7093997	CNV_SNPs
chr10	134199041	134199092	rs7095623	CNV_SNPs
chr10	16154496	16154547	rs7097856	CNV_SNPs
chr11	47286264	47286315	rs7120118	CNV_SNPs
chr11	133495530	133495581	rs7127862	CNV_SNPs
chr1	99780258	99780309	rs712878	CNV_SNPs
chr11	126528471	126528522	rs7129619	CNV_SNPs
chr12	66943149	66943200	rs7134781	CNV_SNPs
chr22	22535111	22535162	rs713644	CNV_SNPs
chr14	25438224	25438275	rs7141943	CNV_SNPs
chr2	212698692	212698743	rs714393	CNV_SNPs
chr14	33107146	33107197	rs7152377	CNV_SNPs
chr13	32507400	32507451	rs715332	CNV_SNPs
chr15	95085048	95085099	rs7161761	CNV_SNPs
chr13	75629001	75629052	rs716655	CNV_SNPs
chr15	59502471	59502522	rs7173371	CNV_SNPs
chr16	61579557	61579608	rs7184753	CNV_SNPs

chr7	11691270	11691321	rs719914	CNV_SNPs
chr16	85880399	85880450	rs7205084	CNV_SNPs
chr21	41326551	41326602	rs720566	CNV_SNPs
chr17	21809368	21809419	rs7215861	CNV_SNPs
chr12	27578886	27578937	rs722135	CNV_SNPs
chr18	54917206	54917257	rs7231097	CNV_SNPs
chr18	51274810	51274861	rs7241627	CNV_SNPs
chr19	56752015	56752066	rs7247429	CNV_SNPs
chr19	39538566	39538617	rs7250431	CNV_SNPs
chr19	51231373	51231424	rs7252682	CNV_SNPs
chr10	95374443	95374494	rs725888	CNV_SNPs
chr19	13519277	13519328	rs7260408	CNV_SNPs
chr20	19602016	19602067	rs728173	CNV_SNPs
chr12	17903318	17903369	rs7297243	CNV_SNPs
chr12	16394822	16394873	rs7298878	CNV_SNPs
chr12	28073146	28073197	rs7309492	CNV_SNPs
chr12	108602944	108602995	rs7313402	CNV_SNPs
chr12	131945103	131945154	rs7314445	CNV_SNPs
chr21	42462162	42462213	rs733428	CNV_SNPs
chr8	91433891	91433942	rs734545	CNV_SNPs
chr1	180810207	180810258	rs7349119	CNV_SNPs
chr2	110053183	110053234	rs7370446	CNV_SNPs
chr22	27561474	27561525	rs738156	CNV_SNPs
chr6	169644006	169644057	rs7382429	CNV_SNPs
chr22	49026427	49026478	rs738686	CNV_SNPs
chr6	25606652	25606703	rs742131	CNV_SNPs
chr10	105517136	105517187	rs743241	CNV_SNPs
chr4	21650342	21650393	rs7437388	CNV_SNPs
chr2	72126394	72126445	rs744027	CNV_SNPs
chr5	161253425	161253476	rs7443990	CNV_SNPs
chr3	150742651	150742702	rs744876	CNV_SNPs
chr5	178268106	178268157	rs7448848	CNV_SNPs
chr11	66677340	66677391	rs746018	CNV_SNPs
chr8	131907750	131907801	rs7463591	CNV_SNPs
chr3	6422859	6422910	rs746493	CNV_SNPs
chr2	4717063	4717114	rs747888	CNV_SNPs
chr11	102020044	102020095	rs7483889	CNV_SNPs
chr3	55487291	55487342	rs751193	CNV_SNPs
chr1	219991315	219991366	rs7525210	CNV_SNPs
chr10	86028553	86028604	rs753795	CNV_SNPs
chr1	171393761	171393812	rs7540116	CNV_SNPs
chr20	21177872	21177923	rs754332	CNV_SNPs
chr1	189243465	189243516	rs7545468	CNV_SNPs
chr1	165044012	165044063	rs7554201	CNV_SNPs
chr9	136548773	136548824	rs756691	CNV_SNPs
chr2	119592951	119593002	rs7567463	CNV_SNPs
chr17	69889160	69889211	rs757996	CNV_SNPs
chr2	135069446	135069497	rs7587559	CNV_SNPs
chr2	54660516	54660567	rs7597093	CNV_SNPs
chr2	169205256	169205307	rs7597374	CNV_SNPs
chr2	170845387	170845438	rs7597437	CNV_SNPs
chr2	106678580	106678631	rs7598657	CNV_SNPs
chr2	41764879	41764930	rs7604226	CNV_SNPs
chr2	38467232	38467283	rs7608771	CNV_SNPs
chr3	177899327	177899378	rs7620308	CNV_SNPs
chr3	106127688	106127739	rs7620623	CNV_SNPs
chr3	23317516	23317567	rs7630344	CNV_SNPs
chr17	8693617	8693668	rs764605	CNV_SNPs
chr3	158104680	158104731	rs7648196	CNV_SNPs
chr4	75662489	75662540	rs7656912	CNV_SNPs
chr4	181797570	181797621	rs7659727	CNV_SNPs
chr14	31375296	31375347	rs766146	CNV_SNPs
chr18	72515813	72515864	rs768759	CNV_SNPs
chr5	59018007	59018058	rs7701748	CNV_SNPs
chr5	75111371	75111422	rs7709242	CNV_SNPs
chr5	26678567	26678618	rs7711007	CNV_SNPs
chr5	31061549	31061600	rs7720376	CNV_SNPs
chr5	136191598	136191649	rs7724913	CNV_SNPs
chr12	77814005	77814056	rs772515	CNV_SNPs
chr5	31652739	31652790	rs7727036	CNV_SNPs
chr5	36368228	36368279	rs7728579	CNV_SNPs
chr5	21824669	21824720	rs7730813	CNV_SNPs
chr6	125258111	125258162	rs7762582	CNV_SNPs
chr1	106053777	106053828	rs777107	CNV_SNPs
chr6	153023158	153023209	rs7774405	CNV_SNPs
chr7	71297490	71297541	rs7777622	CNV_SNPs
chr7	78021610	78021661	rs7790008	CNV_SNPs
chr7	78319702	78319753	rs7791589	CNV_SNPs
chr3	9040968	9041019	rs780930	CNV_SNPs
chr8	121504093	121504144	rs7814311	CNV_SNPs
chr17	3958315	3958366	rs781845	CNV_SNPs
chr8	109735804	109735855	rs7820557	CNV_SNPs
chr12	58814942	58814993	rs782097	CNV_SNPs
chr8	104088093	104088144	rs7833175	CNV_SNPs

chr8	30349888	30349939	rs7840384	CNV_SNPs
chr9	75156031	75156082	rs7857300	CNV_SNPs
chr9	75794663	75794714	rs7875086	CNV_SNPs
chr18	38529945	38529996	rs788433	CNV_SNPs
chr10	117807855	117807906	rs7907882	CNV_SNPs
chr9	12518560	12518611	rs791673	CNV_SNPs
chr10	53793390	53793441	rs7918280	CNV_SNPs
chr11	105610179	105610230	rs7928594	CNV_SNPs
chr11	81185305	81185356	rs7937418	CNV_SNPs
chr11	130716131	130716182	rs7943757	CNV_SNPs
chr11	42138488	42138539	rs7949634	CNV_SNPs
chr12	122974227	122974278	rs7955513	CNV_SNPs
chr12	109960936	109960987	rs7956788	CNV_SNPs
chr12	40763947	40763998	rs7968048	CNV_SNPs
chr13	29678894	29678945	rs7981238	CNV_SNPs
chr13	55384620	55384671	rs7990641	CNV_SNPs
chr13	42178306	42178357	rs7995811	CNV_SNPs
chr13	87971310	87971361	rs7999126	CNV_SNPs
chr13	90822886	90822937	rs8002817	CNV_SNPs
chr14	36558051	36558102	rs8008989	CNV_SNPs
chr14	80471850	80471901	rs8015905	CNV_SNPs
chr14	105906047	105906098	rs8021311	CNV_SNPs
chr15	55966616	55966667	rs8025445	CNV_SNPs
chr3	59967647	59967698	rs802774	CNV_SNPs
chr15	48073742	48073793	rs8036299	CNV_SNPs
chr16	80160719	80160770	rs8057500	CNV_SNPs
chr18	61199924	61199975	rs8082959	CNV_SNPs
chr18	55179116	55179167	rs8089950	CNV_SNPs
chr18	73601981	73602032	rs8096468	CNV_SNPs
chr19	31706385	31706436	rs8109968	CNV_SNPs
chr21	39625753	39625804	rs8129186	CNV_SNPs
chr14	77427443	77427494	rs8181996	CNV_SNPs
chr10	26580010	26580061	rs8190780	CNV_SNPs
chr1	109490431	109490482	rs839543	CNV_SNPs
chr1	7434518	7434569	rs845215	CNV_SNPs
chr7	12463083	12463134	rs847901	CNV_SNPs
chr14	46730873	46730924	rs858891	CNV_SNPs
chr1	30535088	30535139	rs869357	CNV_SNPs
chr19	31301860	31301911	rs879835	CNV_SNPs
chr1	15295993	15296044	rs880922	CNV_SNPs
chr11	12264114	12264165	rs880963	CNV_SNPs
chr17	53367274	53367325	rs884303	CNV_SNPs
chr5	153690447	153690498	rs889029	CNV_SNPs
chr19	18758229	18758280	rs889362	CNV_SNPs
chr6	119108337	119108388	rs902780	CNV_SNPs
chr4	184234561	184234612	rs907360	CNV_SNPs
chr20	52776815	52776866	rs912505	CNV_SNPs
chr22	26388118	26388169	rs916424	CNV_SNPs
chr13	32747096	32747147	rs916732	CNV_SNPs
chr5	97303808	97303859	rs918595	CNV_SNPs
chr2	43951896	43951947	rs919694	CNV_SNPs
chr5	148140671	148140722	rs919723	CNV_SNPs
chr15	77033908	77033959	rs920712	CNV_SNPs
chr19	28788892	28788943	rs921931	CNV_SNPs
chr6	31358917	31358968	rs9266791	CNV_SNPs
chr3	98267263	98267314	rs9289628	CNV_SNPs
chrX	125499310	125499361	rs929019	CNV_SNPs
chr5	28498957	28499008	rs9292261	CNV_SNPs
chr6	45769392	45769443	rs9296469	CNV_SNPs
chr17	10379650	10379701	rs9303259	CNV_SNPs
chr2	36353088	36353139	rs931996	CNV_SNPs
chr6	100302383	100302434	rs9321767	CNV_SNPs
chr6	155699594	155699645	rs9322521	CNV_SNPs
chr11	123605911	123605962	rs9326264	CNV_SNPs
chr6	82852518	82852569	rs9341911	CNV_SNPs
chr6	167630413	167630464	rs9348239	CNV_SNPs
chr6	19728977	19729028	rs9356708	CNV_SNPs
chr6	170798865	170798916	rs9366232	CNV_SNPs
chr6	139292657	139292708	rs9376390	CNV_SNPs
chr6	97729927	97729978	rs9387216	CNV_SNPs
chr6	119748233	119748284	rs9401144	CNV_SNPs
chr9	82832743	82832794	rs9410830	CNV_SNPs
chr6	79720841	79720892	rs9443638	CNV_SNPs
chr6	158411619	158411670	rs9459057	CNV_SNPs
chr20	41759515	41759566	rs947206	CNV_SNPs
chr18	13910704	13910755	rs948330	CNV_SNPs
chr3	16770070	16770121	rs951491	CNV_SNPs
chr13	96897230	96897281	rs9525166	CNV_SNPs
chr8	81288899	81288950	rs952557	CNV_SNPs
chr13	46408011	46408062	rs9526114	CNV_SNPs
chr13	80561410	80561461	rs9531007	CNV_SNPs
chr8	66631946	66631997	rs953593	CNV_SNPs
chr13	34348506	34348557	rs9538560	CNV_SNPs
chr13	21004712	21004763	rs953958	CNV_SNPs

chr2	189541662	189541713	rs955813	CNV_SNPs
chr13	76833658	76833709	rs9573824	CNV_SNPs
chr13	92901888	92901939	rs9584005	CNV_SNPs
chr6	116470535	116470586	rs958738	CNV_SNPs
chr12	92820726	92820777	rs959897	CNV_SNPs
chr4	12321643	12321694	rs961996	CNV_SNPs
chr22	49370808	49370859	rs9628100	CNV_SNPs
chr16	89250856	89250907	rs9635539	CNV_SNPs
chr7	78924663	78924714	rs9648980	CNV_SNPs
chr2	178848461	178848512	rs966433	CNV_SNPs
chrX	86830819	86830870	rs968971	CNV_SNPs
chr2	49394679	49394730	rs969230	CNV_SNPs
chr11	8589622	8589673	rs9736791	CNV_SNPs
chr3	112353212	112353263	rs981181	CNV_SNPs
chr3	41443241	41443292	rs9824480	CNV_SNPs
chr3	28650212	28650263	rs9839541	CNV_SNPs
chr3	28746004	28746055	rs9840640	CNV_SNPs
chr3	162769352	162769403	rs9841791	CNV_SNPs
chr3	164564553	164564604	rs9842109	CNV_SNPs
chr3	59789150	59789201	rs9846213	CNV_SNPs
chr3	25408425	25408476	rs9853460	CNV_SNPs
chr3	134069635	134069686	rs9853621	CNV_SNPs
chr3	75010962	75011013	rs9857154	CNV_SNPs
chr3	155848926	155848977	rs9862153	CNV_SNPs
chr3	190212680	190212731	rs9864293	CNV_SNPs
chr17	55816806	55816857	rs9906591	CNV_SNPs
chr16	79008542	79008593	rs9927800	CNV_SNPs
chr16	1987032	1987083	rs9928312	CNV_SNPs
chr15	54609441	54609492	rs994244	CNV_SNPs
chr18	70571212	70571263	rs9955288	CNV_SNPs
chrX	5699368	5699419	rs996407	CNV_SNPs
chr4	67138117	67138168	rs9968294	CNV_SNPs
chr4	175047613	175047664	rs9992579	CNV_SNPs
chrY	2655004	2655669	SRY	CNV_SNPs
chrY	9175093	9175647	TSPY4	CNV_SNPs
chrY	9176204	9176332	TSPY4	CNV_SNPs
chrY	9176410	9176572	TSPY4	CNV_SNPs
chrY	9176623	9176819	TSPY4	CNV_SNPs
chrY	9176864	9177007	TSPY4	CNV_SNPs
chrY	9177651	9177724	TSPY4	CNV_SNPs
chrY	9195426	9195713	TSPY4	CNV_SNPs
chrY	9215705	9216240	TSPY4	CNV_SNPs
chrY	9216797	9216925	TSPY4	CNV_SNPs
chrY	9217003	9217165	TSPY4	CNV_SNPs
chrY	9217216	9217412	TSPY4	CNV_SNPs
chrY	9217468	9217600	TSPY4	CNV_SNPs
chrY	9218244	9218317	TSPY4	CNV_SNPs
chrY	9236050	9236586	TSPY4	CNV_SNPs
chrY	9237143	9237271	TSPY4	CNV_SNPs
chrY	9237349	9237511	TSPY4	CNV_SNPs
chrY	9237562	9237758	TSPY4	CNV_SNPs
chrY	9237814	9237946	TSPY4	CNV_SNPs
chrY	9238590	9238663	TSPY4	CNV_SNPs
chrY	9365728	9366045	TSPY4	CNV_SNPs
chrY	9366602	9366730	TSPY4	CNV_SNPs
chrY	9366808	9366970	TSPY4	CNV_SNPs
chrY	9367021	9367217	TSPY4	CNV_SNPs
chrY	9367273	9367405	TSPY4	CNV_SNPs
chrY	9368049	9368122	TSPY4	CNV_SNPs
chrY	14821355	14821501	USP9Y	CNV_SNPs
chrY	14832496	14832695	USP9Y	CNV_SNPs
chrY	14834015	14834145	USP9Y	CNV_SNPs
chrY	14837020	14837183	USP9Y	CNV_SNPs
chrY	14838482	14838751	USP9Y	CNV_SNPs
chrY	14847520	14847686	USP9Y	CNV_SNPs
chrY	14847906	14848208	USP9Y	CNV_SNPs
chrY	14848319	14848508	USP9Y	CNV_SNPs
chrY	14850065	14850268	USP9Y	CNV_SNPs
chrY	14851433	14851588	USP9Y	CNV_SNPs
chrY	14869096	14869353	USP9Y	CNV_SNPs
chrY	14870410	14870597	USP9Y	CNV_SNPs
chrY	14872388	14872572	USP9Y	CNV_SNPs
chrY	14882976	14883114	USP9Y	CNV_SNPs
chrY	14885491	14885884	USP9Y	CNV_SNPs
chrY	14887379	14887525	USP9Y	CNV_SNPs
chrY	14888557	14888819	USP9Y	CNV_SNPs
chrY	14889927	14890218	USP9Y	CNV_SNPs
chrY	14890514	14890714	USP9Y	CNV_SNPs
chrY	14891434	14891605	USP9Y	CNV_SNPs
chrY	14898111	14898292	USP9Y	CNV_SNPs
chrY	14898429	14898758	USP9Y	CNV_SNPs
chrY	14902314	14902490	USP9Y	CNV_SNPs
chrY	14903406	14903582	USP9Y	CNV_SNPs
chrY	14904939	14905159	USP9Y	CNV_SNPs

chrY	14922088	14922247	USP9Y	CNV_SNPs
chrY	14922581	14922778	USP9Y	CNV_SNPs
chrY	14923544	14923741	USP9Y	CNV_SNPs
chrY	14924739	14925012	USP9Y	CNV_SNPs
chrY	14928033	14928304	USP9Y	CNV_SNPs
chrY	14930329	14930570	USP9Y	CNV_SNPs
chrY	14945588	14945812	USP9Y	CNV_SNPs
chrY	14949811	14950003	USP9Y	CNV_SNPs
chrY	14951764	14952565	USP9Y	CNV_SNPs
chrY	14952910	14953084	USP9Y	CNV_SNPs
chrY	14954140	14954416	USP9Y	CNV_SNPs
chrY	14954963	14955143	USP9Y	CNV_SNPs
chrY	14958232	14958468	USP9Y	CNV_SNPs
chrY	14958832	14959103	USP9Y	CNV_SNPs
chrY	14959138	14959277	USP9Y	CNV_SNPs
chrY	14968239	14968446	USP9Y	CNV_SNPs
chrY	14968532	14968795	USP9Y	CNV_SNPs
chrY	14969465	14969611	USP9Y	CNV_SNPs
chrY	14971178	14971366	USP9Y	CNV_SNPs
chrY	2821952	2822063	ZFY	CNV_SNPs
chrY	2829089	2829712	ZFY	CNV_SNPs
chrY	2843110	2843310	ZFY	CNV_SNPs
chrY	2843526	2843720	ZFY	CNV_SNPs
chrY	2844685	2844888	ZFY	CNV_SNPs
chrY	2845955	2846146	ZFY	CNV_SNPs
chrY	2846825	2848059	ZFY	CNV_SNPs
chr10	123258030	123258033	FGFR2	281_hotspots
chr10	123274768	123274771	FGFR2	281_hotspots
chr10	123279675	123279678	FGFR2	281_hotspots
chr10	89692903	89692906	PTEN	281_hotspots
chr10	89692921	89692924	PTEN	281_hotspots
chr10	89717671	89717674	PTEN	281_hotspots
chr10	96798738	96798741	CYP2C8	281_hotspots
chr11	108117797	108117800	ATM	281_hotspots
chr11	108218043	108218046	ATM	281_hotspots
chr11	533872	533875	HRAS	281_hotspots
chr11	534283	534286	HRAS	281_hotspots
chr11	534286	534289	HRAS	281_hotspots
chr11	69456210	69456213	CCND1	281_hotspots
chr11	69466020	69466023	CCND1	281_hotspots
chr11	71949085	71949088	INPPL1	281_hotspots
chr12	112888197	112888200	PTPN11	281_hotspots
chr12	112888209	112888212	PTPN11	281_hotspots
chr12	112926886	112926889	PTPN11	281_hotspots
chr12	112926907	112926910	PTPN11	281_hotspots
chr12	133250286	133250289	POLE	281_hotspots
chr12	133253182	133253185	POLE	281_hotspots
chr12	25378559	25378562	KRAS	281_hotspots
chr12	25378646	25378649	KRAS	281_hotspots
chr12	25380274	25380277	KRAS	281_hotspots
chr12	25380277	25380280	KRAS	281_hotspots
chr12	25398279	25398282	KRAS	281_hotspots
chr12	25398282	25398285	KRAS	281_hotspots
chr12	46230639	46230642	ARID2	281_hotspots
chr12	52385714	52385717	ACVR1B	281_hotspots
chr12	56477629	56477632	ERBB3	281_hotspots
chr12	56478853	56478856	ERBB3	281_hotspots
chr12	56482340	56482343	ERBB3	281_hotspots
chr12	58145428	58145431	CDK4	281_hotspots
chr12	58145434	58145437	CDK4	281_hotspots
chr13	111372024	111372027	ING1	281_hotspots
chr13	28592639	28592642	FLT3	281_hotspots
chr13	73649904	73649907	KLF5	281_hotspots
chr14	105246548	105246551	AKT1	281_hotspots
chr14	38061460	38061463	FOXA1	281_hotspots
chr14	65544745	65544748	MAX	281_hotspots
chr14	65560512	65560515	MAX	281_hotspots
chr14	95557627	95557630	DICER1	281_hotspots
chr15	45003744	45003747	B2M	281_hotspots
chr15	66727440	66727443	MAP2K1	281_hotspots
chr15	66727452	66727455	MAP2K1	281_hotspots
chr15	66729161	66729164	MAP2K1	281_hotspots
chr15	90631836	90631839	IDH2	281_hotspots
chr15	90631932	90631935	IDH2	281_hotspots
chr16	3788615	3788618	CREBBP	281_hotspots
chr16	56782198	56782201	NUP93	281_hotspots
chr16	56782201	56782204	NUP93	281_hotspots
chr16	68772217	68772220	CDH1	281_hotspots
chr17	37868206	37868209	ERBB2	281_hotspots
chr17	37879656	37879659	ERBB2	281_hotspots
chr17	37880218	37880221	ERBB2	281_hotspots
chr17	37880260	37880263	ERBB2	281_hotspots
chr17	37880999	37881002	ERBB2	281_hotspots
chr17	37881331	37881334	ERBB2	281_hotspots

chr17	38510601	38510604	RARA	281_hotspots
chr17	40481574	40481577	STAT3	281_hotspots
chr17	47696423	47696426	SPOP	281_hotspots
chr17	47696429	47696432	SPOP	281_hotspots
chr17	47696641	47696644	SPOP	281_hotspots
chr17	47696686	47696689	SPOP	281_hotspots
chr17	7576852	7576855	TP53	281_hotspots
chr17	7577079	7577082	TP53	281_hotspots
chr17	7577091	7577094	TP53	281_hotspots
chr17	7577094	7577097	TP53	281_hotspots
chr17	7577097	7577100	TP53	281_hotspots
chr17	7577103	7577106	TP53	281_hotspots
chr17	7577112	7577115	TP53	281_hotspots
chr17	7577115	7577118	TP53	281_hotspots
chr17	7577118	7577121	TP53	281_hotspots
chr17	7577121	7577124	TP53	281_hotspots
chr17	7577127	7577130	TP53	281_hotspots
chr17	7577139	7577142	TP53	281_hotspots
chr17	7577515	7577518	TP53	281_hotspots
chr17	7577533	7577536	TP53	281_hotspots
chr17	7577536	7577539	TP53	281_hotspots
chr17	7577545	7577548	TP53	281_hotspots
chr17	7577548	7577551	TP53	281_hotspots
chr17	7577554	7577557	TP53	281_hotspots
chr17	7577557	7577560	TP53	281_hotspots
chr17	7577563	7577566	TP53	281_hotspots
chr17	7577566	7577569	TP53	281_hotspots
chr17	7577569	7577572	TP53	281_hotspots
chr17	7577572	7577575	TP53	281_hotspots
chr17	7577578	7577581	TP53	281_hotspots
chr17	7577605	7577608	TP53	281_hotspots
chr17	7578188	7578191	TP53	281_hotspots
chr17	7578200	7578203	TP53	281_hotspots
chr17	7578203	7578206	TP53	281_hotspots
chr17	7578206	7578209	TP53	281_hotspots
chr17	7578209	7578212	TP53	281_hotspots
chr17	7578233	7578236	TP53	281_hotspots
chr17	7578260	7578263	TP53	281_hotspots
chr17	7578263	7578266	TP53	281_hotspots
chr17	7578266	7578269	TP53	281_hotspots
chr17	7578269	7578272	TP53	281_hotspots
chr17	7578370	7578371	TP53	281_hotspots
chr17	7578392	7578395	TP53	281_hotspots
chr17	7578401	7578404	TP53	281_hotspots
chr17	7578404	7578407	TP53	281_hotspots
chr17	7578410	7578413	TP53	281_hotspots
chr17	7578440	7578443	TP53	281_hotspots
chr17	7578476	7578479	TP53	281_hotspots
chr17	7578497	7578500	TP53	281_hotspots
chr17	7578506	7578509	TP53	281_hotspots
chr17	7578524	7578527	TP53	281_hotspots
chr17	7578533	7578536	TP53	281_hotspots
chr17	7578551	7578554	TP53	281_hotspots
chr17	7579311	7579314	TP53	281_hotspots
chr17	7579353	7579356	TP53	281_hotspots
chr17	7579587	7579590	TP53	281_hotspots
chr17	7796793	7796796	CHD3	281_hotspots
chr18	45368209	45368212	SMAD2	281_hotspots
chr18	48591887	48591890	SMAD4	281_hotspots
chr18	48591917	48591920	SMAD4	281_hotspots
chr18	48593404	48593407	SMAD4	281_hotspots
chr18	48604786	48604789	SMAD4	281_hotspots
chr19	11144112	11144115	SMARCA4	281_hotspots
chr19	3118940	3118943	GNA11	281_hotspots
chr19	4117548	4117551	MAP2K2	281_hotspots
chr19	52715969	52715972	PPP2R1A	281_hotspots
chr19	52715981	52715984	PPP2R1A	281_hotspots
chr1	11169374	11169377	MTOR	281_hotspots
chr1	11184571	11184574	MTOR	281_hotspots
chr1	11217228	11217231	MTOR	281_hotspots
chr1	115256527	115256530	NRAS	281_hotspots
chr1	115258742	115258745	NRAS	281_hotspots
chr1	115258745	115258748	NRAS	281_hotspots
chr1	120612005	120612008	NOTCH2	281_hotspots
chr1	158606544	158606547	SPTA1	281_hotspots
chr1	209879199	209879202	HSD11B1	281_hotspots
chr1	226252133	226252136	H3F3A	281_hotspots
chr1	226252154	226252157	H3F3A	281_hotspots
chr1	226567665	226567668	PARP1	281_hotspots
chr1	27087502	27087505	ARID1A	281_hotspots
chr1	27100206	27100208	ARID1A	281_hotspots
chr1	27106353	27106356	ARID1A	281_hotspots
chr1	47395873	47395876	CYP4A11	281_hotspots
chr20	20033085	20033088	CRNKL1	281_hotspots

chr20	39832809	39832812	ZHX3	281_hotspots
chr20	57484419	57484422	GNAS	281_hotspots
chr21	44514775	44514778	U2AF1	281_hotspots
chr21	44524454	44524457	U2AF1	281_hotspots
chr21	44524484	44524487	U2AF1	281_hotspots
chr22	22127161	22127164	MAPK1	281_hotspots
chr22	41565528	41565531	EP300	281_hotspots
chr22	41566473	41566476	EP300	281_hotspots
chr2	16082315	16082318	MYCN	281_hotspots
chr2	178098798	178098801	NFE2L2	281_hotspots
chr2	178098801	178098804	NFE2L2	281_hotspots
chr2	178098804	178098807	NFE2L2	281_hotspots
chr2	178098807	178098810	NFE2L2	281_hotspots
chr2	178098942	178098945	NFE2L2	281_hotspots
chr2	178098951	178098954	NFE2L2	281_hotspots
chr2	178098954	178098957	NFE2L2	281_hotspots
chr2	178098957	178098960	NFE2L2	281_hotspots
chr2	178098966	178098969	NFE2L2	281_hotspots
chr2	198265474	198265477	SF3B1	281_hotspots
chr2	198266609	198266612	SF3B1	281_hotspots
chr2	198266831	198266834	SF3B1	281_hotspots
chr2	198267358	198267361	SF3B1	281_hotspots
chr2	198267478	198267481	SF3B1	281_hotspots
chr2	198267481	198267484	SF3B1	281_hotspots
chr2	209113110	209113113	IDH1	281_hotspots
chr2	25457240	25457243	DNMT3A	281_hotspots
chr2	29432662	29432665	ALK	281_hotspots
chr2	29443694	29443697	ALK	281_hotspots
chr2	39281775	39281778	SOS1	281_hotspots
chr2	61719469	61719472	XPO1	281_hotspots
chr2	70315178	70315181	PCBP1	281_hotspots
chr3	10183762	10183765	VHL	281_hotspots
chr3	10188319	10188320	VHL	281_hotspots
chr3	12645697	12645700	RAF1	281_hotspots
chr3	138374242	138374245	PIK3CB	281_hotspots
chr3	178916724	178916727	PIK3CA	281_hotspots
chr3	178916853	178916856	PIK3CA	281_hotspots
chr3	178916874	178916877	PIK3CA	281_hotspots
chr3	178916928	178916931	PIK3CA	281_hotspots
chr3	178916943	178916946	PIK3CA	281_hotspots
chr3	178916964	178916965	PIK3CA	281_hotspots
chr3	178921547	178921550	PIK3CA	281_hotspots
chr3	178921550	178921553	PIK3CA	281_hotspots
chr3	178922362	178922365	PIK3CA	281_hotspots
chr3	178927979	178927982	PIK3CA	281_hotspots
chr3	178928078	178928081	PIK3CA	281_hotspots
chr3	178936081	178936084	PIK3CA	281_hotspots
chr3	178936090	178936093	PIK3CA	281_hotspots
chr3	178936093	178936096	PIK3CA	281_hotspots
chr3	178938933	178938936	PIK3CA	281_hotspots
chr3	178952005	178952008	PIK3CA	281_hotspots
chr3	178952071	178952074	PIK3CA	281_hotspots
chr3	178952074	178952077	PIK3CA	281_hotspots
chr3	178952083	178952086	PIK3CA	281_hotspots
chr3	30732968	30732971	TGFBR2	281_hotspots
chr3	41266096	41266099	CTNNB1	281_hotspots
chr3	41266099	41266102	CTNNB1	281_hotspots
chr3	41266102	41266105	CTNNB1	281_hotspots
chr3	41266108	41266111	CTNNB1	281_hotspots
chr3	41266111	41266114	CTNNB1	281_hotspots
chr3	41266123	41266126	CTNNB1	281_hotspots
chr3	41266135	41266138	CTNNB1	281_hotspots
chr3	41268764	41268767	CTNNB1	281_hotspots
chr3	41274908	41274911	CTNNB1	281_hotspots
chr3	49412896	49412899	RHOA	281_hotspots
chr3	49412902	49412905	RHOA	281_hotspots
chr3	49413007	49413010	RHOA	281_hotspots
chr4	153244182	153244185	FBXW7	281_hotspots
chr4	153247286	153247289	FBXW7	281_hotspots
chr4	153247364	153247367	FBXW7	281_hotspots
chr4	153249382	153249385	FBXW7	281_hotspots
chr4	153251904	153251907	FBXW7	281_hotspots
chr4	1803563	1803566	FGFR3	281_hotspots
chr4	1803566	1803569	FGFR3	281_hotspots
chr4	1806103	1806106	FGFR3	281_hotspots
chr4	55594220	55594223	KIT	281_hotspots
chr5	112116591	112116594	APC	281_hotspots
chr5	112128142	112128145	APC	281_hotspots
chr5	112128190	112128193	APC	281_hotspots
chr5	112162890	112162893	APC	281_hotspots
chr5	112164615	112164618	APC	281_hotspots
chr5	112173916	112173919	APC	281_hotspots
chr5	112174093	112174096	APC	281_hotspots
chr5	112174630	112174633	APC	281_hotspots

chr5	112175302	112175305	APC	281_hotspots
chr5	112175422	112175425	APC	281_hotspots
chr5	112175506	112175509	APC	281_hotspots
chr5	112175512	112175515	APC	281_hotspots
chr5	112175575	112175578	APC	281_hotspots
chr5	112175638	112175641	APC	281_hotspots
chr5	35876390	35876393	IL7R	281_hotspots
chr5	67588950	67588953	PIK3R1	281_hotspots
chr5	67589137	67589140	PIK3R1	281_hotspots
chr5	67591096	67591099	PIK3R1	281_hotspots
chr5	67591105	67591108	PIK3R1	281_hotspots
chr5	67591150	67591152	PIK3R1	281_hotspots
chr7	105177175	105177178	RINT1	281_hotspots
chr7	116412042	116412043	MET	281_hotspots
chr7	140453131	140453134	BRAF	281_hotspots
chr7	140453134	140453137	BRAF	281_hotspots
chr7	140453143	140453146	BRAF	281_hotspots
chr7	140453146	140453149	BRAF	281_hotspots
chr7	140453152	140453155	BRAF	281_hotspots
chr7	140453986	140453987	BRAF	281_hotspots
chr7	140481400	140481403	BRAF	281_hotspots
chr7	140481409	140481412	BRAF	281_hotspots
chr7	148508725	148508728	EZH2	281_hotspots
chr7	151188047	151188050	RHEB	281_hotspots
chr7	55211078	55211081	EGFR	281_hotspots
chr7	55221820	55221823	EGFR	281_hotspots
chr7	55233041	55233044	EGFR	281_hotspots
chr7	55241706	55241709	EGFR	281_hotspots
chr7	55259513	55259516	EGFR	281_hotspots
chr7	55259522	55259525	EGFR	281_hotspots
chr7	6426891	6426894	RAC1	281_hotspots
chr7	6441972	6441975	RAC1	281_hotspots
chr7	77885497	77885500	MAGI2	281_hotspots
chr8	128750724	128750727	MYC	281_hotspots
chr8	128750988	128750991	MYC	281_hotspots
chr8	38272305	38272308	FGFR1	281_hotspots
chr8	38274848	38274851	FGFR1	281_hotspots
chr9	127911966	127911969	PPP6C	281_hotspots
chr9	137328349	137328352	RXRA	281_hotspots
chr9	16436466	16436469	BNC2	281_hotspots
chr9	21970967	21970970	CDKN2A	281_hotspots
chr9	21971015	21971018	CDKN2A	281_hotspots
chr9	21971027	21971030	CDKN2A	281_hotspots
chr9	21971093	21971096	CDKN2A	281_hotspots
chr9	21971108	21971111	CDKN2A	281_hotspots
chr9	21971117	21971120	CDKN2A	281_hotspots
chr9	21971183	21971186	CDKN2A	281_hotspots
chr9	21971204	21971207	CDKN2A	281_hotspots
chr9	21974676	21974679	CDKN2A	281_hotspots
chr9	78848415	78848418	PCSK5	281_hotspots
chr9	80537109	80537112	GNAQ	281_hotspots
chrX	44922801	44922804	KDM6A	281_hotspots
chrX	70349257	70349260	MED12	281_hotspots
